# Pegcetacoplan Treatment and Consensus Features of Geographic Atrophy Over 24 Months

**DOI:** 10.1001/jamaophthalmol.2024.1269

**Published:** 2024-05-09

**Authors:** Dun Jack Fu, Pallavi Bagga, Gunjan Naik, Sophie Glinton, Livia Faes, Bart Liefers, Rosana Lima, Georgina Wignall, Pearse A. Keane, Estelle Ioannidou, Ana Paula Ribeiro Reis, Alex McKeown, Lukas Scheibler, Praveen J. Patel, Ismail Moghul, Nikolas Pontikos, Konstantinos Balaskas

**Affiliations:** 1 National Institute for Health Research Biomedical Research Centre at Moorfields Eye Hospital and University College London Institute of Ophthalmology, London, United Kingdom; 2 Department of Ophthalmology, Erasmus University Medical Center, Rotterdam, the Netherlands; 3 Apellis Pharmaceuticals, Waltham, Massachusetts

## Abstract

**Importance:**

Despite widespread availability and consensus on its advantages for detailed imaging of geographic atrophy (GA), spectral-domain optical coherence tomography (SD-OCT) might benefit from automated quantitative OCT analyses in GA diagnosis, monitoring, and reporting of its landmark clinical trials.

**Objective:**

To analyze the association between pegcetacoplan and consensus GA SD-OCT end points.

**Design, Setting, and Participants:**

This was a post hoc analysis of 11 614 SD-OCT volumes from 936 of the 1258 participants in 2 parallel phase 3 studies, the Study to Compare the Efficacy and Safety of Intravitreal APL-2 Therapy With Sham Injections in Patients With Geographic Atrophy (GA) Secondary to Age-Related Macular Degeneration (OAKS) and Study to Compare the Efficacy and Safety of Intravitreal APL-2 Therapy With Sham Injections in Patients With Geographic Atrophy (GA) Secondary to Age-Related Macular Degeneration (DERBY). OAKS and DERBY were 24-month, multicenter, randomized, double-masked, sham-controlled studies conducted from August 2018 to July 2020 among adults with GA with total area 2.5 to 17.5 mm^2^ on fundus autofluorescence imaging (if multifocal, at least 1 lesion ≥1.25 mm^2^). This analysis was conducted from September to December 2023.

**Interventions:**

Study participants received pegcetacoplan, 15 mg per 0.1-mL intravitreal injection, monthly or every other month, or sham injection monthly or every other month.

**Main Outcomes and Measures:**

The primary end point was the least squares mean change from baseline in area of retinal pigment epithelium and outer retinal atrophy in each of the 3 treatment arms (pegcetacoplan monthly, pegcetacoplan every other month, and pooled sham [sham monthly and sham every other month]) at 24 months. Feature-specific area analysis was conducted by Early Treatment Diabetic Retinopathy Study (ETDRS) regions of interest (ie, foveal, parafoveal, and perifoveal).

**Results:**

Among 936 participants, the mean (SD) age was 78.5 (7.22) years, and 570 participants (60.9%) were female. Pegcetacoplan, but not sham treatment, was associated with reduced growth rates of SD-OCT biomarkers for GA for up to 24 months. Reductions vs sham in least squares mean (SE) change from baseline of retinal pigment epithelium and outer retinal atrophy area were detectable at every time point from 3 through 24 months (least squares mean difference vs pooled sham at month 24, pegcetacoplan monthly: −0.86 mm^2^; 95% CI, −1.15 to −0.57; *P* < .001; pegcetacoplan every other month: −0.69 mm^2^; 95% CI, −0.98 to −0.39; *P* < .001). This association was more pronounced with more frequent dosing (pegcetacoplan monthly vs pegcetacoplan every other month at month 24: −0.17 mm^2^; 95% CI, −0.43 to 0.08; *P* = .17). Stronger associations were observed in the parafoveal and perifoveal regions for both pegcetacoplan monthly and pegcetacoplan every other month.

**Conclusions and Relevance:**

These findings offer additional insight into the potential effects of pegcetacoplan on the development of GA, including potential effects on the retinal pigment epithelium and photoreceptors.

**Trial Registration:**

ClinicalTrials.gov Identifiers: NCT03525600 and NCT03525613

## Key Points

**Question:**

What is the association between intravitreal pegcetacoplan and consensus spectral domain optical coherence tomography features of geographic atrophy?

**Findings:**

In this secondary analysis of 2 randomized clinical trials, stronger associations were observed in the parafoveal and perifoveal macular regions; significant foveal involvement at baseline limited associations observed in the foveal region.

**Meaning:**

These results provide evidence to suggest that pegcetacoplan may delay atrophy of both retinal pigment epithelium and photoreceptors.

## Teaser

This secondary analysis of 2 randomized clinical trials evaluates the association between pegcetacoplan and geographic atrophy on spectral-domain optical coherence tomography.

## Introduction

Age-related macular degeneration (AMD) is a major cause of blindness worldwide,^1^ and almost 90% of people with AMD have the nonneovascular subtype of AMD,^2^ the defining end point lesion of which is geographic atrophy (GA). Pathological features include irreversible degeneration of macular photoreceptors, retinal pigment epithelium (RPE), the Bruch membrane, and choriocapillaris with corresponding scotomas.^3^ Reducing GA lesion enlargement rate is thus an important therapeutic goal, especially if it is associated with functional benefits over 1 or 2 or many years. Change in GA lesion area over time is the anatomical clinical trial end point for progression assessment.^4,5^ GA lesions can be detected using different ophthalmic imaging modalities, among which short-wavelength fundus autofluorescence (FAF) is the most commonly used in clinical trials. Short-wavelength FAF reveals autofluorescent molecules in the retina, most notably those contained in lipofuscin and melanolipofuscin, long-lasting inclusion bodies in RPE cell bodies.^6,7^ RPE atrophy is a dominant feature of GA, visualized in FAF images as dark areas due to replacement of intact RPE with scattered highly pigmented cells in a bed of atrophy.^8,9,10,11^ Pathological features of other retinal layers in GA are not as readily accessible with FAF.^12,13^ FAF is thus primarily a binary grading system for whether RPE atrophy, a major feature of GA, is present or absent. Yet interpretation of FAF images is subject to variability. For example, hypoautofluorescence can correspond to RPE loss, as would be expected in GA; however, it does not reflect the variable integrity of photoreceptors.^14^ Additionally, precisely defining lesion boundaries can be challenging for FAF images,^10,15,16^ especially in the foveal region, where blue light absorption by luteal pigment naturally reduces the FAF signal.^17^

To better detect and differentiate changes of individual retinal layers during GA development and progression, spectral-domain optical coherence tomography (SD-OCT) has been put forth as a reference standard for GA.^12,18^ As one of the most frequently used diagnostic imaging procedures throughout medicine,^19^ SD-OCT is more widespread in routine clinical practice than FAF and provides cross-sectional visualization of the retinal layers, RPE, and often also the choroid in high resolution.^20^ Consensus SD-OCT features have been suggested as future trial end points for GA by the National Eye Institute and US Food and Drug Administration (FDA).^21^

The Consensus of Atrophy Meetings (CAM) group, an international consortium of experts in AMD and retinal imaging, has defined GA disease progression based on SD-OCT structural markers.^2,12^ However, few GA studies to date have used the CAM-defined end points, as manual segmentation of OCT volume scans is time consuming, labor intensive, and limited by interrater variability.^22,23,24^ These barriers might be mitigated by automated image segmentation algorithms. We recently developed a deep-learning based platform that uses CAM-defined OCT features to detect and quantify GA and its components (ie, quantitative OCT biomarkers), with a grading performance comparable to human specialist graders on an external validation dataset.^25,26^ Despite its widespread availability and advantages for detailed diagnosis and monitoring of GA, SD-OCT has yet to follow its own precedent in neovascular AMD, where it is the primary imaging modality used to inform both routine clinical practice and anatomic outcomes in trials.^27^

A complement protein C3 inhibitor, pegcetacoplan, has been shown to inhibit growth of GA in phase 2^28^ and phase 3 clinical trials, although no benefit on visual acuity prespecified secondary outcomes was identified.^28^ It is the first drug approved by the FDA for GA. GA lesion area in the pivotal studies leading to pegcetacoplan approval by the FDA was assessed conventionally by FAF manual segmentation. In this post hoc analysis, our deep-learning automated quantitative OCT analytical platform was used to assess the association between intravitreal pegcetacoplan and GA features, on SD-OCT volume scans from the phase 3 Study to Compare the Efficacy and Safety of Intravitreal APL-2 Therapy With Sham Injections in Patients With Geographic Atrophy (GA) Secondary to Age-Related Macular Degeneration (DERBY; NCT03525600) and Study to Compare the Efficacy and Safety of Intravitreal APL-2 Therapy With Sham Injections in Patients With Geographic Atrophy (GA) Secondary to Age-Related Macular Degeneration (OAKS; NCT03525613) (protocols in Supplement 1) over a 24-month follow-up period. Furthermore, change in visual function was weakly associated with SD-OCT GA features but not pegcetacoplan treatment compared to sham.

## Methods

### Study Design and Cohort Selection

This post hoc analysis included 11 614 SD-OCT volumes from 936 participants enrolled in the 24-month, phase 3, multicenter, randomized, double-masked, sham-controlled OAKS and DERBY studies assessing the efficacy of intravitreal pegcetacoplan was in eyes with GA secondary to AMD (eMethods in Supplement 2) and conducted from August 2018 to July 2020.^28^ Study protocols were approved by institutional review boards or ethics committees at each site. Both studies adhered to the Declaration of Helsinki. All participants provided written informed consent that permitted deidentified post hoc image analysis research. The Consolidated Standards of Reporting Trials (CONSORT) reporting guideline has been adhered to, and a CONSORT diagram is provided in eFigure 1 in Supplement 2. This analysis was conducted from September to December 2023. Race data were gathered and reported via electronic health record at participating sites and reported to assess the diversity of the patient cohort and identify potential underrepresented patient populations for future research.

### Image Analysis Workflow

**Figure 1.**
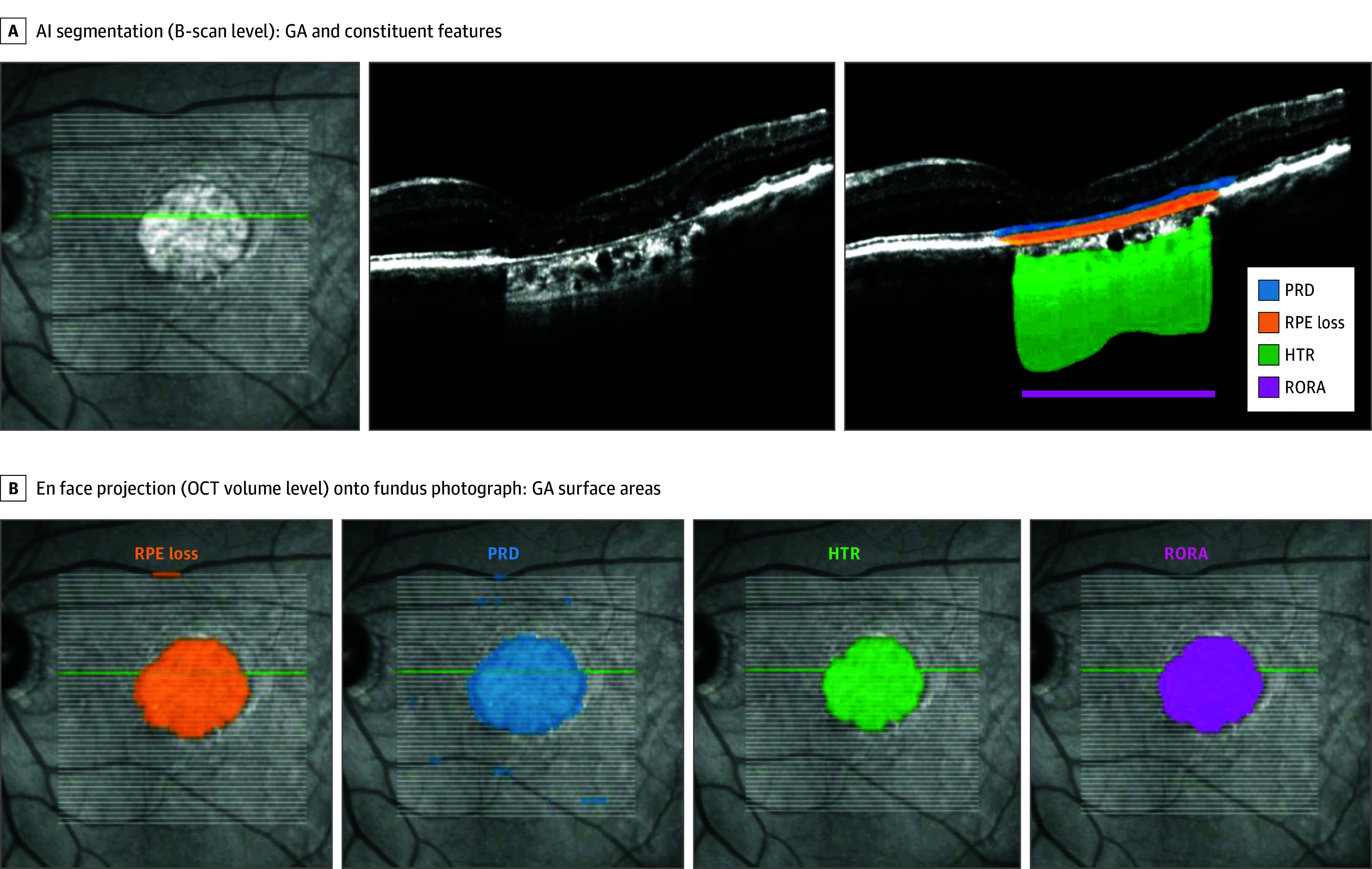
Segmentation of Geographic Atrophy (GA) Features From Spectral-Domain Optical Coherence Tomography (SD-OCT) Imaging For each SD-OCT volume, all B-scans were segmented for photoreceptor degeneration (PRD), retinal pigment epithelium (RPE) loss, hypertransmission (HTR), and RPE and outer retinal atrophy (RORA). RORA was taken to be overlapping regions of the 3 former features—that is, co-occurrence as per A-scan. Exemplar segmentation of a single B-scan and its axis along en face fundus photograph shown. Resultant feature probability maps from total volume segmentations collectively presented by projection onto en face fundus photograph. AI indicates artificial intelligence.

GA and its constituent features were automatically segmented from all SD-OCT volumes at the Artificial Intelligence Lab of the Moorfields Ophthalmic Reading Centre as previously described (Figure 1 and eMethods in Supplement 2).^25,29^ Photoreceptor degeneration (PRD) in isolation was defined as PRD without overlapping RPE loss or hypertransmission and RPE loss and outer retinal atrophy (RORA) as regions of overlapping RPE loss, PRD, and hypertransmission. RORA can thus be considered as a continuous variable that encompasses both incomplete RORA and complete RORA.^12,30^ Areas of each feature could thus be summarized by retinal regions divided up into the Early Treatment Diabetic Retinopathy Study (ETDRS) grid of 3 concentric rings: the foveal region (circle of 1-mm diameter centered on the foveal center); the inner ETDRS annulus of 3-mm outer diameter and 1-mm inner diameter; and the outer perifoveal ETDRS annulus with 6-mm diameter outer diameter and 3-mm inner diameter.^31,32^ The foveal center was defined as the deepest point of the foveal pit (eFigure 1 and eMethods in Supplement 2).

### Study Outcomes

The primary end point was the least squares mean change from baseline in area of RORA between each of the 3 treatment arms (15 mg per 0.1-mL intravitreal injection pegcetacoplan monthly, every other month, and pooled sham [sham monthly and sham every other month]) at 24 months. Secondary end points included changes in best-corrected visual acuity (BCVA; measured in ETDRS score letters) and areas of RORA, RPE loss, hypertransmission, PRD, and PRD in isolation at 12, 18, and 24 months postbaseline. Subanalyses were carried out to evaluate agreement across the ETDRS regions.

### Statistical Analysis

Analyses were performed on the modified intent-to-treat population, defined as all participants who received at least 1 injection of pegcetacoplan or sham and had a baseline and at least 1 postbaseline value of GA lesion area as measured by SD-OCT in the study eye (eMethods in Supplement 2). Statistical analyses were performed by Apellis Pharmaceuticals biostatistics team with SAS software version 9.4 (SAS Institute). Data visualization was carried out using R version 4.2.3 (R Foundation). Two-tailed *P* values <.05 were considered significant.

## Results

### Cohort Characteristics at Baseline

Of the 1258 participants with GA enrolled in the OAKS and DERBY trials, 936 (456 from OAKS, 480 from DERBY) were taken forward to analysis (eFigure 2 in Supplement 2). The overall mean (SD) age was 78.5 (7.22) years; 570 participants (60.9%) were female; and 860 participants were White (92.1% vs not reported, 61 [6.5%] and other 13 [1.5%], including Asian, Black or African American, and multiple races, consolidated owing to small numbers) (Table 1). Baseline BCVA was similar across all treatment groups.

**Table 1.** Demographic and Clinical Characteristics at Baseline

Characteristic	No. (%)				
	Pegcetacoplan monthly (n = 310)	Pegcetacoplan EOM (n = 309)	Sham injection monthly (n = 157)	Sham injection EOM (n = 160)	Pooled sham (n = 317)
Age, y					
Mean (SD)	78.5 (7.00)	78.7 (7.58)	78.5 (7.34)	78.3 (6.89)	78.4 (7.11)
Median (IQR; range)	79.0 (74.0-83.0; 60-95)	79.0 (73.0-84.0; 60-100)	79.0 (74.0-84.0; 60-94)	79.0 (74.0-83.0; 61-96)	79.0 (74.0-83.0; 60-96)
Age group, y					
<65	8 (2.6)	12 (3.9)	7 (4.5)	7 (4.4)	14 (4.4)
≥65-<75	80 (25.8)	76 (24.6)	38 (24.2)	36 (22.5)	74 (23.3)
≥75-<85	158 (51.0)	144 (46.6)	75 (47.8)	88 (55.0)	163 (51.4)
≥85	64 (20.6)	77 (24.9)	37 (23.6)	29 (18.1)	66 (20.8)
Sex					
Female	189 (61.0)	177 (57.3)	94 (59.9)	110 (68.8)	204 (64.4)
Male	121 (39.0)	132 (42.7)	63 (40.1)	50 (31.3)	113 (35.6)
Race^a^					
White	284 (91.6)	283 (91.6)	146 (93.0)	149 (93.1)	295 (93.1)
Not reported	22 (7.1)	21 (6.8)	8 (5.1)	10 (6.3)	18 (5.7)
Other^b^	4 (1.3)	5 (1.6)	3 (1.9)	1 (0.6)	4 (1.3)
BCVA, ETDRS letter score (approximate Snellen equivalent)					
Mean	59.9 (20/63)	59.3 (20/63)	57.8 (20/63)	59.9 (20/63)	58.9 (20/63)
SD	16.25	15.95	17.34	16.14	16.75
Median	62.0 (20/63)	62.0 (20/63)	60.0 (20/63)	60.0 (20/63)	60.0 (20/63)
IQR	50.0-72.0 (20/100-20/40)	48.0-72.0 (20/100-20/40)	40.0-73.0 (20/160-20/40)	50.0-73.0 (20/100-20/40)	45.0-73.0 (20/125-20/40)
Range	24-87 (20/320-20/20)	24-93 (20/320-20/15)	26-86 (20/320-20/20)	24-87 (20/320-20/20)	24-87 (20/320-20/20)
GA lesion location					
Foveal center involvement	191 (61.6)	192 (62.1)	110 (70.1)	100 (62.5)	210 (66.2)
No foveal center involvement	119 (38.4)	117 (37.9)	47 (29.9)	60 (37.5)	107 (33.8)
GA lesion size by FAF, mm^2^					
Mean (SD)	8.23 (3.94)	8.28 (3.97)	8.00 (3.99)	8.41 (4.02)	8.21 (4.00)
Median (IQR; range)	7.39 (4.84-11.38; 2.26-18.11)	7.55 (5.04-10.99; 2.61-17.70)	7.44 (4.59-10.18; 2.59-16.85)	7.79 (5.02-11.34; 2.64-17.77)	7.62 (4.73-10.62; 2.58-17.77)
RORA, mm^2^					
Mean (SD)	7.22 (3.47)	7.30 (3.34)	7.02 (3.44)	7.25 (3.48)	7.13 (3.45)
Median (IQR; range)	6.48 (4.45-9.61; 1.68-17.98)	6.58 (4.60-9.60; 1.66-16.86)	6.48 (4.25-9.06; 2.00-16.34)	6.67 (4.20-9.51; 2.21-16.23)	6.61 (4.23-9.32; 2.00-16.34)
RPE loss, mm^2^					
Mean (SD)	7.82 (3.62)	7.80 (3.47)	7.48 (3.52)	7.75 (3.63)	7.62 (3.57)
Median (IQR; range)	7.12 (4.96-10.12; 1.96-18.59)	7.07 (4.94-10.09; 1.66-19.90)	6.96 (4.573-9.35; 2.32-17.71)	7.03 (4.59-10.22; 2.30-16.74)	7.02 (4.57-9.71; 2.32-17.71)
Hypertransmission, mm^2^					
Mean (SD)	8.57 (3.84)	8.61 (3.65)	8.25 (3.74)	8.54 (3.78)	8.40 (3.76)
Median (IQR; range)	8.04 (5.45-11.37; 2.05-20.88)	8.28 (5.55-11.25; 1.92-18.33)	7.56 (5.41-10.38; 2.52-19.73)	8.21 (5.35-10.91; 2.84-17.28)	7.95 (5.38-10.57; 2.52-19.73)
Photoreceptor degeneration, mm^2^					
Mean (SD)	13.60 (5.52)	13.39 (5.32)	12.82 (5.08)	13.60 (5.28)	13.22 (5.19)
Median (IQR; range)	12.96 (9.17-17.70; 3.41-26.67)	12.46 (9.18-17.45; 2.87-27.98)	12.41 (8.67-16.09; 3.36-26.22)	13.47 (9.85-16.87; 3.95-27.32)	12.85 (9.21-16.69; 3.36-27.32)
Photoreceptor degeneration in isolation, mm^2^					
Mean (SD)	4.74 (3.11)	4.55 (2.92)	4.42 (2.80)	4.81 (2.66)	4.62 (2.73)
Median (IQR; range)	3.96 (4.46-9.62; 1.68-17.98)	3.69 (4.60-9.60; 1.66-16.87)	3.58 (4.25-9.06; 2.00-16.34)	4.75 (4.20-9.51; 2.21-16.23)	4.05 (4.23-9.33; 2.00-16.34)

Abbreviations: BCVA, best-corrected visual acuity; EOM, every other month; ETDRS, Early Treatment Diabetic Retinopathy Study; GA, geographic atrophy; FAF, fundus autofluorescence; RORA, RPE and outer retinal atrophy; RPE, retinal pigment epithelium.

^a^ Race data were collected via electronic health record at participating sites and reported to assess the diversity of the patient cohort and identify potential underrepresented patient populations for future research.

^b^ Other included Asian, Black or African American, and multiple races, consolidated owing to small numbers.

Total GA lesion areas and location were calculated from segmentations of same-day FAF and SD-OCT imaging (Table 1 and eFigure 3 in Supplement 2). Areas of all SD-OCT GA features were observed to be similar across each of the treatment arms when considering the entire ETDRS grid; the 1-mm diameter ETDRS foveal region (0.79 mm^2^); the inner, perifoveal ETDRS annulus (6.23 mm^2^), and the outer perifoveal ETDRS annulus (21.2 mm^2^) (eFigure 3 in Supplement 2). With FAF, the overall number of eyes with detected GA involvement of the foveal center was 593 of 936 (63.4%), ranging from 191 of 310 (61.6%) in the pegcetacoplan monthly group to 110 of 157 (70.1%) in the sham monthly group (Table 1). Yet at the SD-OCT level, GA features were found to involve the foveal center for most study eyes at baseline: RORA (644 of 936 eyes [69%]), RPE loss (659 of 936 eyes [70%]), and PRD (829 of 936 eyes [89%]). Moreover, these features were detected within the foveal region of 1-mm diameters for most study eyes: RORA (897 of 936 eyes [96%]), RPE loss (904 of 936 [97%]), and PRD (936 of 936 eyes [100%]); with a mean occupancy of 57% RORA, 60% RPE-loss, 67% hypertransmission; and 86% PRD.

### Assessment of GA Lesion Growth Rate Over Time Using SD-OCT

**Figure 2.**
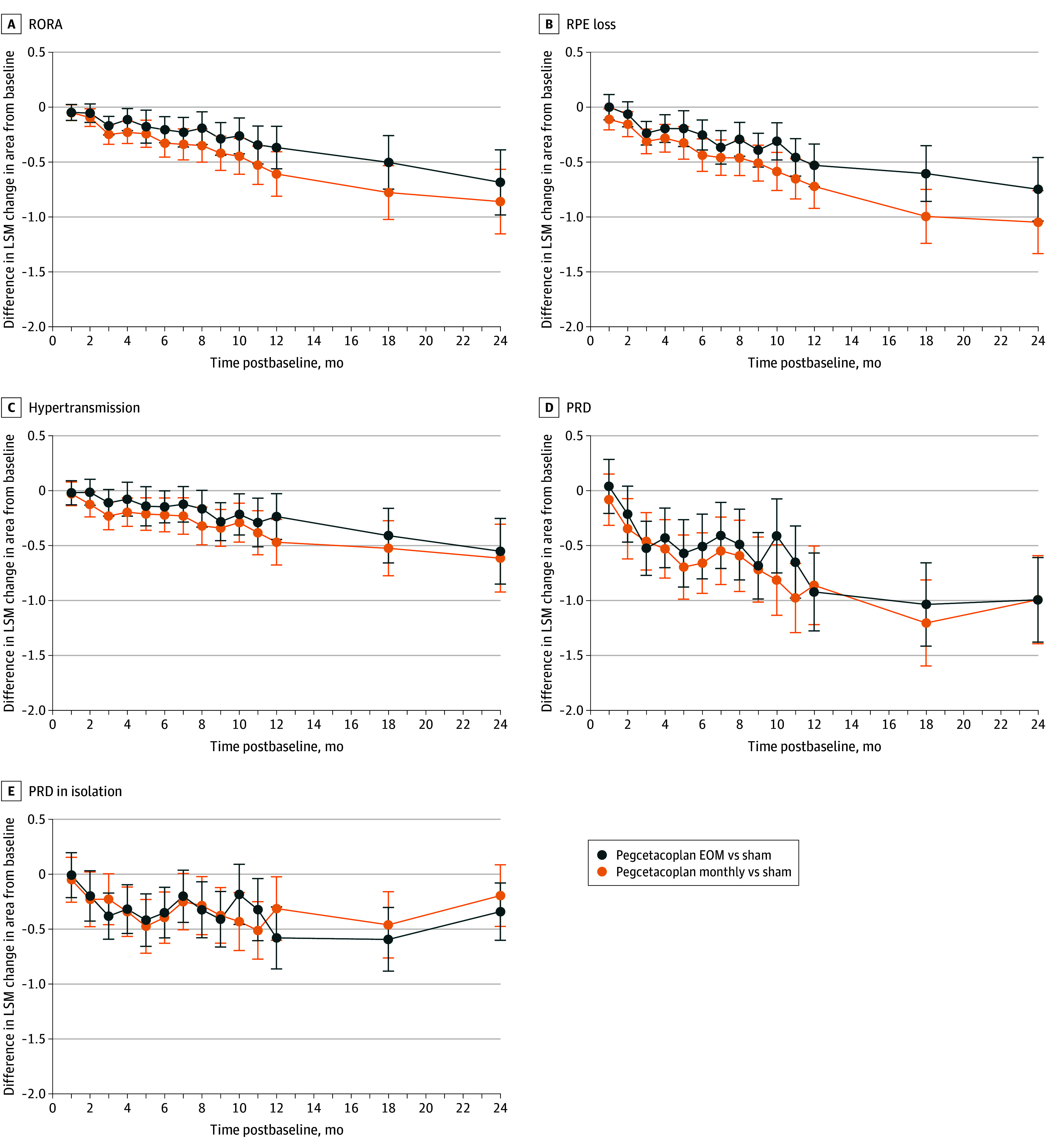
Association Between Pegcetacoplan and Geographic Atrophy (GA) Features at the Spectral-Domain Optical Coherence Tomography (SD-OCT) Level The figure shows differences between treatment groups in least square mean (LSM) mean change in area of GA features from baseline. SD-OCT GA features considered were retinal pigment epithelium (RPE) loss and outer retinal atrophy (RORA), RPE loss, hypertransmission, photoreceptor degeneration (PRD), and PRD in isolation in study eyes. Changes within total Early Treatment Diabetic Retinopathy Study regions were considered. Graphs show LSMs and SEs (error bars) by treatment group and time postbaseline, which were estimated from a mixed-effects model with a random intercept at the level of the participant that included the following as cross-level interactions: treatment, presence of choroidal neovascularization in the fellow eye (yes or no), baseline GA lesion area (<7.5 mm^2^ or ≥7.5 mm^2^), baseline SD-OCT GA feature, analysis visit, treatment × analysis visit, and baseline SD-OCT GA feature × analysis visit. EOM indicates every other month.

Within the circular 6-mm diameter ETDRS grid, pegcetacoplan was associated with reduced growth rates of SD-OCT biomarkers of GA for up to 24 months. In comparison to the pooled sham group, a reduction in the least squares mean change of RORA area from baseline was detectable at month 3 in study eyes receiving pegcetacoplan every other month (−0.17 mm^2^; 95% CI, −0.25 to −0.08; *P* < .001), and at every time point up to month 24 least squares mean difference vs pooled sham at month 24, pegcetacoplan monthly: −0.86 mm^2^; 95% CI, −1.15 to −0.57; *P* < .001; pegcetacoplan every other month: −0.69 mm^2^; 95% CI, −0.98 to −0.39; *P* < .001) (Table 2, Figure 2, and eFigure 4 in Supplement 2). This association was more pronounced with pegcetacoplan monthly than with pegcetacoplan every other month dosing (−0.17 mm^2^; 95% CI, −0.43 to 0.08; *P* = .17). A reduction in RORA growth was observed between study eyes receiving pegcetacoplan monthly and the pooled sham group and was apparent from month 2.

**Table 2.** Analysis of Change in Area From Baseline at 12, 18, and 24 Months Postbaseline Within the Circular, 6-mm Diameter Early Treatment Diabetic Retinopathy Study Grid

Variable	PM	*P* value		PEOM	*P* value (PEOM vs pooled sham)	Pooled sham
		PM vs pooled sham	PM vs PEOM			
Change in area of RORA from baseline through month 24 with MMRM, mm^2^						
No. of individuals included in the model	298	NA	NA	299	NA	307
Change in RORA at month 12, mm^2^						
LSM (SE)	1.18 (0.07)	<.001	.006	1.42 (0.06)	<.001	1.79 (0.08)
Difference (95% CI) in LSM (vs pooled sham)	−0.61 (−0.81 to −0.41)			−0.37 (−0.56 to −0.18)		NA
Percentage difference (vs pooled sham)	−34.0			−20.6		NA
Difference (95% CI) in LSM (vs PEOM)	−0.24 (−0.41 to −0.07)			NA		NA
Percentage difference (vs PEOM)	−16.9			NA		NA
Change in RORA at month 18, mm^2^						
LSM (SE)	1.83 (0.08)	<.001	.009	2.11 (0.08)	<.001	2.61 (0.10)
Difference (95% CI) in LSM (vs pooled sham)	−0.78 (−1.02 to −0.53)			−0.50 (−0.75 to −0.26)		NA
Percentage difference (vs pooled sham)	−29.8			−19.3		NA
Difference (95% CI) in LSM (vs PEOM)	−0.27 (−0.48 to −0.07)			NA		NA
Percentage difference (vs PEOM)	−13.0			NA		NA
Change in RORA at month 24, mm^2^						
LSM (SE)	2.62 (0.09)	<.001	.17	2.79 (0.09)	<.001	3.48 (0.12)
Difference (95% CI) in LSM (vs pooled sham)	−0.86 (−1.15 to −0.57)			−0.69 (−0.98 to −0.39)		NA
Percentage difference (vs pooled sham)	−24.7			−19.7		NA
Difference (95% CI) in LSM (vs PEOM)	−0.17 (−0.43 to 0.08)			NA		NA
Percentage difference (vs PEOM)	−6.3			NA		NA
RPE loss						
No. of individuals included in the model	298	NA	NA	299	NA	307
Change in RPE loss at month 12, mm^2^						
LSM (SE)	1.18 (0.07)	<.001	.04	1.38 (0.07)	<.001	1.91 (0.08)
Difference (95% CI) in LSM (vs pooled sham)	−0.72 (−0.92 to −0.52)			−0.53 (−0.72 to −0.34)		NA
Percentage difference (vs pooled sham)	−37.9			−27.8		NA
Difference (95% CI) in LSM (vs PEOM)	−0.19 (−0.38 to −0.01)			NA		NA
Percentage difference (vs PEOM)	−13.9			NA		NA
Change in RPE loss at month 18, mm^2^						
LSM (SE)	1.74 (0.08)	<.001	.001	2.13 (0.09)	<.001	2.73 (0.10)
Difference (95% CI) in LSM (vs pooled sham)	−0.99 (−1.24 to −0.75)			−0.61 (−0.86 to −0.35)		NA
Percentage difference (vs pooled sham)	−36.4			−22.1		NA
Difference (95% CI) in LSM (vs PEOM)	−0.39 (−0.63 to −0.15)			NA		NA
Percentage difference (vs PEOM)	−18.3			NA		NA
Change in RPE loss at month 24, mm^2^						
LSM (SE)	2.48 (0.10)	<.001	.03	2.78 (0.10)	<.001	3.53 (0.11)
Difference (95% CI) in LSM (vs pooled sham)	−1.05 (−1.33 to −0.76)			−0.75 (−1.04 to −0.46)		NA
Percentage difference (vs pooled sham)	−29.7			−21.2		NA
Difference (95% CI) in LSM (vs PEOM)	−0.30 (−0.57 to −0.03)			NA		NA
Percentage difference (vs PEOM)	−10.8			NA		NA
Hypertransmission						
No. of individuals included in the model	298	NA	NA	299	NA	307
Change in hypertransmission at month 12, mm^2^						
LSM (SE)	1.24 (0.07)	<.001	.02	1.47 (0.07)	.02	1.71 (0.08)
Difference (95% CI) in LSM (vs pooled sham)	−0.47 (−0.68 to −0.26)			−0.24 (−0.45 to −0.03)		NA
Percentage difference (vs pooled sham)	−27.5			−13.9		NA
Difference (95% CI) in LSM (vs PEOM)	−0.23 (−0.44 to −0.03)			NA		NA
Percentage difference (vs PEOM)	−15.8			NA		NA
Change in hypertransmission at month 18, mm^2^						
LSM (SE)	2.05 (0.09)	<.001	.34	2.17 (0.09)	<.001	2.58 (0.10)
Difference (95% CI) in LSM (vs pooled sham)	−0.53 (−0.78 to −0.28)			−0.41 (−0.66 to −0.16)		NA
Percentage difference (vs pooled sham)	−20.4			−15.9		NA
Difference (95% CI) in LSM (vs PEOM)	−0.12 (−0.35 to 0.12)			NA		NA
Percentage difference (vs PEOM)	−5.3			NA		NA
Change in hypertransmission at month 24, mm^2^						
LSM (SE)	2.84 (0.11)	<.001	.67	2.90 (0.10)	<.001	3.46 (0.12)
Difference (95% CI) in LSM (vs pooled sham)	−0.62 (−0.92 to −0.31)			−0.55 (−0.85 to −0.25)		NA
Percentage difference (vs pooled sham)	−17.8			−16.0		NA
Difference (95% CI) in LSM (vs PEOM)	−0.06 (−0.35 to 0.22)			NA		NA
Percentage difference (vs PEOM)	−2.2			NA		NA
PRD						
No. of individuals included in the model	298	NA	NA	299	NA	307
Change in PRD at month 12, mm^2^						
LSM (SE)	0.99 (0.12)	<.001	.72	0.93 (0.12)	<.001	1.86 (0.14)
Difference (95% CI) in LSM (vs pooled sham)	−0.86 (−1.22 to −0.50)			−0.92 (−1.28 to −0.57)		NA
Percentage difference (vs pooled sham)	−46.5			−49.7		NA
Difference (95% CI) in LSM (vs PEOM)	0.06 (−0.27 to 0.39)			NA		NA
Percentage difference (vs PEOM)	6.5			NA		NA
Change in PRD at month 18, mm^2^						
LSM (SE)	1.47 (0.14)	<.001	.36	1.64 (0.13)	<.001	2.67 (0.15)
Difference (95% CI) in LSM (vs pooled sham)	−1.21 (−1.60 to −0.81)			−1.04 (−1.42 to −0.66)		NA
Percentage difference (vs pooled sham)	−45.1			−38.8		NA
Difference (95% CI) in LSM (vs PEOM)	−0.17 (−0.53 to 0.19)			NA		NA
Percentage difference (vs PEOM)	−10.3			NA		NA
Change in PRD at month 24, mm^2^						
LSM (SE)	2.24 (0.15)	<.001	.99	2.23 (0.14)	<.001	3.23 (0.15)
Difference (95% CI) in LSM (vs pooled sham)	−0.99 (−1.39 to −0.59)			−0.99 (−1.38 to −0.61)		NA
Percentage difference (vs pooled sham)	−30.8			−30.8		NA
Difference (95% CI) in LSM (vs PEOM)	0.00 (−0.38 to 0.38)			NA		NA
Percentage difference (vs PEOM)	0.0			NA		NA
PRD in isolation						
No. of individuals included in the model	298	NA	NA	299	NA	307
Change in PRD in isolation at month 12, mm^2^						
LSM (SE)	−0.30 (0.10)	.003	0.5	NA	<.001	0.02 (0.11)
Difference (95% CI) in LSM (vs pooled sham)	−0.31 (−0.60 to −0.02)			−0.58 (−0.86 to −0.30)		NA
Percentage difference (vs pooled sham)	−2056.3			−3808.2		NA
Difference (95% CI) in LSM (vs PEOM)	0.27 (−0.00 to 0.53)			NA		NA
Percentage difference (vs PEOM)	−47.2			NA		NA
Change in PRD in isolation at month 18, mm^2^						
LSM (SE)	−0.49 (0.10)	.002	.32	−0.63 (0.09)	<.001	−0.03 (0.12)
Difference (95% CI) in LSM (vs pooled sham)	−0.46 (−0.76 to −0.16)			−0.59 (−0.88 to −0.30)		NA
Percentage difference (vs pooled sham)	1443.9			1859.7		NA
Difference (95% CI) in LSM (vs PEOM)	0.13 (−0.13 to 0.39)			NA		NA
Percentage difference (vs PEOM)	−21.2			NA		NA
Change in PRD in isolation at month 24, mm^2^						
LSM (SE)	−0.53 (0.10)	.17	.28	−0.68 (0.09)	.01	−0.34 (0.10)
Difference (95% CI) in LSM (vs pooled sham)	−0.19 (−0.47 to 0.09)			−0.34 (−0.60 to −0.08)		NA
Percentage difference (vs pooled sham)	57.9			101.4		NA
Difference (95% CI) in LSM (vs PEOM)	0.15 (−0.12 to 0.41)			NA		NA
Percentage difference (vs PEOM)	−21.6			NA		NA

Abbreviations: LSM, least squares mean; MMRM, mixed model for repeated measures; NA, not applicable; PEOM, pegcetacoplan every other month; PM, pegcetacoplan monthly; PRD, photoreceptor degeneration; RORA, RPE and outer retinal atrophy; RPE, retinal pigment epithelium.

For the constituent features of GA, RPE loss, hypertransmission, and PRD, slower growth rates vs pooled sham were observed in study eyes receiving pegcetacoplan monthly between baseline and month 24 (RPE loss least squares mean difference, −1.05 mm^2^; 95% CI, −1.33 to −0.76; *P* < .001; hypertransmission least squares mean difference, −0.62 mm^2^; 95% CI, −0.92 to −0.31; *P* < .001; PRD least squares mean difference,−0.99 mm^2^; 95% CI, −1.39 to −0.59; *P* < .001) (Figure 2 and Table 2). A smaller least squares mean difference compared to sham was observed in the pegcetacoplan every other month group at month 24 for RPE loss and hypertransmission. PRD in isolation represents an earlier stage in GA development and has been shown^26^ to be prognostic for GA progression (eFigure 6 in Supplement 2). Negative growth (ie, reduction) of PRD in isolation was observed with the pegcetacoplan monthly and pegcetacoplan every other month groups at every time point following baseline up to 24 postbaseline (mean [SE] pegcetacoplan monthly −0.53 [0.10] vs pegcetacoplan every other month, −0.68 [0.09] mm^2^). Yet PRD in isolation was observed to grow in the pooled sham group at every time point following baseline up until month 18.

Growth rate of RORA (pegcetacoplan monthly, 34% [−0.61 mm^2^; 95% CI, −0.81 to −0.41; *P* < .001]; pegcetacoplan every other month, 28% [−0.53 mm^2^; 95% CI, −0.72 to −0.34; *P* < .001]) and RPE loss (pegcetacoplan monthly, 38% [−0.72 mm^2^; 95% CI, −0.92 to −0.52; *P* < .001]; pegcetacoplan every other month, 28% [−0.53 mm^2^; 95% CI, −0.72 to −0.34; *P* < .001]) were reduced in comparison with pooled sham group at 12 months. With FAF, pegcetacoplan monthly and pegcetacoplan every other month were associated with reduced GA lesion growth compared with sham treatment by 21% (–0.41 mm^2^; 95% CI, –0.64 to –0.18; *P* < .001) and 16% (–0.32 mm^2^; 95% CI, –0.54 to –0.09; *P* = .006), respectively, vs pooled sham at 12 months in OAKS and by 12% (–0.23 mm^2^; 95% CI, –0.47 to 0.01; *P* = .06) and 11% (–0.21 mm^2^; 95% CI, –0.44 to 0.03; *P* = .09), respectively, in DERBY.^28^

### Pegcetacoplan Results by ETDRS Region

GA lesions tend to initially form in the parafoveal or perifoveal regions.^33^ Scotomas in the parafoveal and perifoveal regions can decrease low-luminance visual acuity and contrast sensitivity, so visual function abnormalities can develop before the foveal region is affected by GA.^34^ As subregions of the macula (foveal, parafoveal, and perifoveal regions) contribute differently toward visual function, we assessed pegcetacoplan treatment by ETDRS subregion (eFigures 4 and 5 and eTable 1 in Supplement 2). In the perifoveal region, differences compared to the pooled sham group were observed in least squares mean area growth vs baseline of RORA, RPE loss, hypertransmission, and PRD for both the pegcetacoplan monthly and the pegcetacoplan every other month groups at month 24. The area growth of PRD in isolation was reduced in both groups, but only detectable in the pegcetacoplan every other month group at month 24 (least squares mean difference, −0.36 mm^2^; 95% CI, −0.61 to −0.11; *P* = .005 vs sham). Similarly, there was a reduction compared to the sham group in expansion of RORA and RPE loss in the parafoveal region for the pegcetacoplan monthly (RORA least squares mean difference, −0.22 mm^2^; 95% CI, −0.36 to −0.09; *P* = .001; RPE loss least squares mean difference, −0.25 mm^2^; 95% CI, −0.38 to −0.12; *P* < .001) and pegcetacoplan every other month (RORA least squares mean difference, −0.17 mm^2^; 95% CI, −0.31 to −0.03; *P* = .02; RPE loss least squares mean difference, −0.18 mm^2^; 95% CI, −0.32 to −0.04; *P* = .01) at month 24. When considering the foveal region (1-mm diameter; 0.79 mm^2^), a difference was only observed in RPE loss at month 24 in the pegcetacoplan monthly group. Observed data showed a similar trend via sensitivity analyses (eFigure 7 and eTable 4 in Supplement 2). While FAF imaging suggested involvement of the central foveal point for 63% (593 of 936) of the cohort (Table 1), PRD of the central foveal point was detected for 89% (829 of 936) of cases and more than 95% of study eyes (899 of 936) already had RORA occupying the foveal region at baseline.

### Association With BCVA

**Figure 3.**
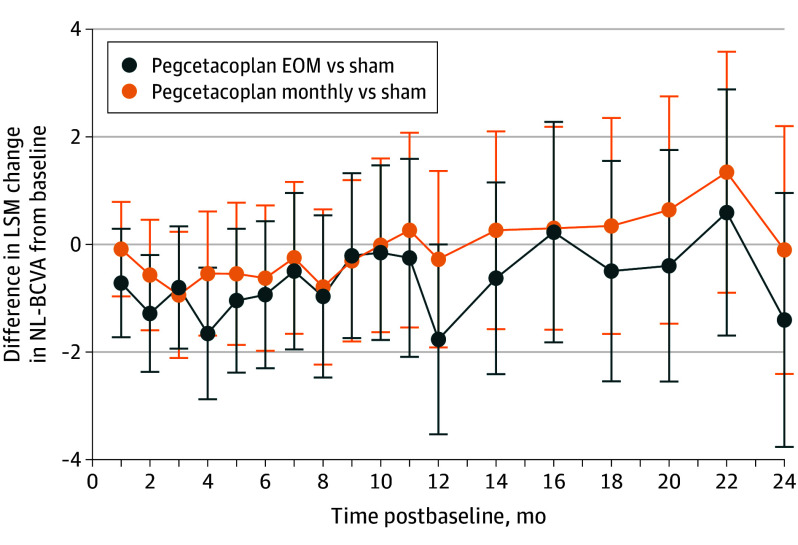
Least-Squares Mean (LSM) Change in Normal-Luminance Best-Corrected Visual Acuity (NL-BCVA) Score The figure shows differences between treatment groups in LSM change in NL-BCVA in Early Treatment Diabetic Retinopathy Study letter score from baseline. The graph shows LSMs and SEs (error bars) by treatment group and time postbaseline, which were estimated from a mixed-effects model with a random intercept at the level of the participant that included the following as cross-level interactions: treatment, baseline geographic atrophy lesion area (<7.5 mm^2^ or ≥7.5 mm^2^); baseline BCVA score; analysis visit; baseline presence of choroidal neovascularization in the fellow eye (yes or no); analysis visit × treatment; baseline BCVA score × analysis visit. EOM indicates every other month.

When compared to pooled sham, pegcetacoplan was not associated with BCVA change from baseline to months 12, 18, and 24 (Figure 3 and eTable 2 in Supplement 2). For each SD-OCT feature of GA, change from baseline to month 24 within the 6-mm diameter ETDRS region showed weak associations with change in BCVA across each of the treatment groups (eTable 3 in Supplement 2).

## Discussion

This secondary analysis of 2 randomized clinical trials used automatically quantified SD-OCT features to offer additional insight into the protective association between pegcetacoplan and GA area growth, demonstrating an association between pegcetacoplan and delayed atrophy of both RPE and photoreceptors. SD-OCT findings were consistent with the FAF-based end point analysis for the phase 3 OAKS and DERBY trials.

RPE cells have autofluorescent characteristics that FAF imaging detects and exploits to signify RPE atrophy.^6,18,35,36,37,38,39,40^ It is therefore interesting that at 12 and 24 months, our analyses of RPE loss for the combined cohort showed consistently greater reductions in RPE loss than what was observed for GA area growth with FAF. Photoreceptor loss commonly preceded RPE loss.^41,42^ It may represent an early retinal change in GA development and can predict progression.^41,42^ In line with previous reports,^14^ FAF did not accurately capture growth rates of PRD and thus PRD in isolation. Our analysis therefore offers novel insights into the associations of pegcetacoplan in early GA stages where photoreceptors are affected in addition to subsequent atrophy of RPE. We observed that, apart from PRD in isolation, all features of GA with SD-OCT showed a mean positive growth rate over the observation period of 24 months, with pegcetacoplan monthly and pegcetacoplan every other month showing slower average growths in this time than sham (eFigures 4 and 5 in Supplement 2; Table 2). With sham, PRD in isolation is stable over the first 18 months, indicating that the rate of PRD was similar to RPE loss. A negative growth rate observed from 18 months onward suggests RPE loss was occurring faster than PRD. Pegcetacoplan treatment groups showed negative growth for PRD in isolation from month 1. Based on the model that PRD preceded RPE loss, we propose pegcetacoplan may be protective of both photoreceptors and RPE, and that protection of photoreceptors can be observed more readily than with RPE.

Our detailed analyses of SD-OCT GA features per ETDRS region offers unique insights into the extent and maturity of GA the population included in the pegcetacoplan phase 3 program. Involvement of the central foveal point, based on FAF (63%), was found to be lower compared to when based on SD-OCT quantification (>95%). PRD of the central foveal point was detected in 89% of cases. The extent of disease and foveal involvement may therefore cause ceiling effects for the study interventions. This is corroborated by the mean change in area from baseline over time per ETDRS region for the consensus GA features. In the perifoveal and parafoveal regions, a clear reduction in GA area growth rate compared to sham was observed with pegcetacoplan monthly and pegcetacoplan every other month, as would be expected from the results for overall GA growth reported for FAF and for the total ETDRS region. However, associations observed in the foveal region were minimal, which may be surprising considering a common perception that the fovea is initially spared in GA.^33^ In the context of our baseline analysis of the fovea, however, the association between treatment and SD-OCT GA features within the foveal region may have been limited by high foveal region and foveal center involvement of those same features at baseline. To fully assess pegcetacoplan effects on GA growth in each of the macula regions, studies are needed that include participants with a diverse range in foveal region occupancy of GA features. This may impact the observed treatment effects on clinical outcomes that measure foveal health, such as BCVA.

### Limitations

This study has limitations. Our methodology permits tracking of macular lesions over time for personalized GA monitoring, as well as lesion registration for interpatient modeling and research. However, there are extramacular regions—and thus extramacular pathology—not captured by the SD-OCT. As a post hoc analysis, direct causal relationships cannot be determined and therefore the findings are limited to exploration of the association between treatment and changes on SD-OCT.

## Conclusions

This study demonstrates the potential importance of SD-OCT imaging for assessing growth and response to treatment of GA. Assessment of pegcetacoplan effects on GA growth with FAF does not necessarily consider prognostic structural changes, such as high incidence of PRD in the foveal region. Using automatically segmented longitudinal quantitative OCT biomarkers, we offer additional insight into the protective association of pegcetacoplan treatment against GA area growth. Importantly, our data show that SD-OCT features were associated with visual outcome and that pegcetacoplan was associated with a delay in atrophy of both the RPE and the photoreceptors. However, an association between pegcetacoplan treatment with BCVA was not demonstrated.

## Notes

Supplement 1. Trial protocols

Supplement 2. eFigure 1. Segmentation of ETDRS regions from SD-OCT imaging eFigure 2. CONSORT flow diagram eFigure 3. SD-OCT biomarkers of GA at baseline eFigure 4. Difference in least squares mean growth of GA features substratified by the ETDRS region eFigure 5. Least squares mean change in GA features eFigure 6. Example of participants with slow GA growth (top) and with fast GA growth (bottom) over treatment period eFigure 7. Sensitivity analyses eTable 1. Analysis of change in area from baseline at 12, 18, and 24 months post baseline by ETDRS region eTable 2. Least squares mean change in best-corrected visual acuity from baseline at 12, 18, and 24 months post-baseline eTable 3. Spearman Correlation of Change from Baseline between GA SD-OCT feature and BCVA at Month 24 eTable 4. Change in area from baseline at 12, 18, and 24 months post baseline – Sensitivity analyses eMethods

Supplement 3. Data sharing statement
