# Supplementary material for: Pegcetacoplan Treatment and Consensus Features of Geographic Atrophy Over 24 Months
Source: JAMA Ophthalmol. 2024 May 9;142(6):548–58. doi: 10.1001/jamaophthalmol.2024.1269 (PMC11082756; doi:10.1001/jamaophthalmol.2024.1269)
Supplement: Supplement 1. — Trial protocols [file jamaophthalmol-e241269-s001.pdf]

PEGCETACOPLAN (APL-2)

PROTOCOL APL2-303

A PHASE 3, MULTI-CENTER, RANDOMIZED, DOUBLE-MASKED, SHAM-CONTROLLED STUDY TO COMPARE THE EFFICACY AND SAFETY OF INTRAVITREAL PEGCETACOPLAN THERAPY WITH SHAM INJECTIONS IN PATIENTS WITH GEOGRAPHIC ATROPHY (GA) SECONDARY TO AGE-RELATED MACULAR DEGENERATION (AMD)

US IND No.: 124784

EudraCT No.: 2018-001436-22

Phase: 3

Version: Amendment 5

Date: 12 August 2020

Confidentiality Statement

This document is confidential. It contains proprietary information of Apellis Pharmaceuticals, Inc. Any viewing or disclosure of such information that is not authorized in writing by Apellis Pharmaceuticals, Inc, is strictly prohibited. Such information may be used solely for the purpose of reviewing or performing this study.

INVESTIGATOR AGREEMENT

**Long Title:**	A Phase 3, Multi-Center, Randomized, Double-Masked, Sham-Controlled Study to Compare the Efficacy and Safety of Intravitreal Pegcetacoplan Therapy with Sham Injections in Patients with Geographic Atrophy (GA) Secondary to Age-Related Macular Degeneration (AMD)
**Short Title:**	Derby
**Protocol Number, Version, and Date:**	APL2-303 / Amendment 5 / 12 August 2020
**Study Phase:**	Phase 3
**Sponsor Name and Address:**	Apellis Pharmaceuticals, Inc<br>100 5th Ave<br>Waltham, MA 02451<br>USA
**Investigational Test Article:**	Pegcetacoplan intravitreal injection (also known as APL-2)
**US IND Number:**	124784
**EudraCT Number:**	2018-001436-22
**Indication Studied:**	Geographic atrophy secondary to age-related macular degeneration
**Investigator Agreement:**	I have read the clinical study described herein, recognize its confidentiality, and agree to conduct the described trial in compliance with Good Clinical Practice (GCP), the ethical principles contained within the Declaration of Helsinki, this protocol, and all applicable regulatory requirements.

Principal Investigator:

Name: ____________________________________

Signature: ________________________________

Date: ______/______/______ (DD/MMM/YYYY)

Pegcetacoplan
APL2-303 Protocol Amendment 5 12 August 2020

SPONSOR INFORMATION

**Sponsor**	Apellis Pharmaceuticals, Inc<br>100 5th Ave<br>Waltham, MA 02451<br>USA
**Sponsor Representative:**	PPD<br>PPD<br>PPD<br>PPD

Signature: PPD

Date: PPD

PROTOCOL AMENDMENT SUMMARY OF CHANGES

Amendment 5: Summary of Changes From the Previous Version

Amendment Date: 12 August 2020

Updates to the protocol implemented in this amendment are provided in the table below.

Description of Change	Section(s) Affected by Change
Nonsubstantial changes that did not impact content of the document have been made for clarity.	Entire document
Updated company address	Investigator Agreement and Sponsor Information
Changed APL-2 to International Nonproprietary Name: pegcetacoplan; updated related abbreviations and protocol title	Entire document
Added the descriptor "maximum" when referencing reading speed	Synopsis, Section 5.1.2, Section 5.1.5, Section 12.7.2, Section 12.7.3
Removed the 6-month follow-up period and updated the study length from 30 months to 24 months and made it a 30- or 60-day follow-up period based on treatment group because that length of time is deemed sufficient to evaluate the safety of pegcetacoplan based on its half-life in the vitreous. Additionally, there is an option for subjects to enroll in a separate study during which longer-term safety and efficacy will be collected.	Synopsis, Section 8, Section 8.1.4.2, Section 11.8 (previously Section 11.10)
Updated the text regarding the sample size to both simplify and reduce redundancies	Synopsis, Section 12.5
Previous Section 4.1.3.1 Pharmacokinetics moved to Section 4.1.3.2 to follow Section 4.1.3.1 Toxicology	Section 4.1.3.1 and Section 4.1.3.2
Updated the formulation information	Section 4.1.2 and Section 7.3.3.1
Revised number of injections for each treatment arm as the injections at Month 24 will no longer be administered	Section 7.3.1 (Table 1), Section 8
Updated the text regarding endophthalmitis for clarity	Section 7.5.3
Updated study schema	Section 8
Revised Study Procedures based on the removal of the Month 24 injection and the 6-month follow-up period	Section 8
Revised the language regarding genotyping samples to indicate it these samples will be collected for those subjects who consent to the analysis	Section 9.7
Updated the language within the Adverse Events definition section to be consistent with the new Apellis standard language	Section 11.1
Updated the language within the Recording Adverse Events section to be consistent with the new Apellis standard language	Section 11.2
Removed the section on Treatment and Follow-Up of Adverse Events as the information contained within it is now included in other revised sections to align with new Apellis standard language	Section 11.3

Updated the language within the Reporting Adverse Events section to be consistent with the new Apellis standard language.	Section 11.3 (previously Section 11.4)
Updated the language within the Serious Adverse Events section to be consistent with the new Apellis standard language	Section 11.4 (previously Section 11.5)
Removed the section on Unexpected Adverse Events or Unexpected Suspected Adverse Reactions as the information contained within it is now included in other revised sections to align with new Apellis standard language	previously Section 11.6
Updated the language within the Pregnancy section to be consistent with the new Apellis standard language	Section 11.6 (previously Section 11.8)
Removed APL-2 investigator's brochure from references	Section 15
Updated Footnotes G and M	Appendix A, Appendix B, Appendix C, Appendix D, and Appendix K
Deleted follow-up visits from Schedules of Activities	Appendix B and Appendix D and Tables B and D of Appendix K
Deleted pegcetacoplan and sham administration and postinjection assessment from Visit 20 of the Schedule of Activities for monthly and every-other-month treatment groups Month 13 to Month 30	Appendix B and Appendix D and Tables B and D of Appendix K

1. TABLE OF CONTENTS, LIST OF TABLES, AND LIST OF FIGURES

TABLE OF CONTENTS

INVESTIGATOR AGREEMENT.....2
SPONSOR INFORMATION.....3
PROTOCOL AMENDMENT SUMMARY OF CHANGES.....4
1. TABLE OF CONTENTS, LIST OF TABLES, AND LIST OF FIGURES.....6
2. SYNOPSIS.....12
3. ABBREVIATIONS.....20
4. INTRODUCTION.....22
4.1. Background.....22
4.1.1. Age-Related Macular Degeneration.....22
4.1.2. Pegcetacoplan.....22
4.1.3. Nonclinical Data.....23
CCI [redacted]
4.1.4. Clinical Data.....26
4.1.5. Rationale.....27
4.1.5.1. Rationale for Pegcetacoplan for Treatment of Geographic Atrophy.....27
4.1.6. Dose Selection.....27
4.2. Risk/Benefit.....28
5. STUDY OBJECTIVES.....29
5.1. Study Objectives.....29
5.1.1. Primary Objective.....29
5.1.2. Key Secondary Objectives.....29
5.1.3. Secondary Objectives.....29
5.1.4. Safety Objectives.....29
5.1.5. Exploratory Objectives.....30
6. PATIENT POPULATION.....31
6.1. Patient Selection.....31
6.1.1. Inclusion Criteria.....31
6.1.2. Exclusion Criteria.....32

6.2. Women of Childbearing Potential 33
6.3. Women of Non-Childbearing Potential 33
6.4. Approved Methods of Contraception 33
6.5. Discontinuation of Subjects 34
7. TREATMENT OF SUBJECTS 35
7.1. Allocation to Treatment 35
7.2. Masking and Minimization of Bias 35
7.2.1. Unmasking 35
7.3. Dosage and Administration 36
7.3.1. Dose Levels and Treatment Arms 36
7.3.2. Treatment Administration 36
7.3.3. Drug Supplies 37
7.3.3.1. Identity of Investigational Product 37
7.3.3.2. Storage 37
7.3.3.3. Accountability 37
7.3.4. Intravitreal Pegcetacoplan Administration 37
7.4. Sham Injection Administration 38
7.5. Concomitant Therapies 38
7.5.1. Treatment of New Exudation Related to Active Choroidal Neovascularization in the Study Eye and/or Fellow Eye 38
7.5.2. Prohibited Therapies 39
7.5.3. Endophthalmitis Treatment 39
8. STUDY PROCEDURES 40
8.1. Study Visit Schedule 41
8.1.1. Screening Period—Within 28 Days Prior to Randomization/Treatment (Day –28 to Day –1) 41
8.1.1.1. Visit 1—All Subjects 41
8.1.2. Randomization/Initial Treatment—Day 1—Within 28 Days of Screening 41
8.1.2.1. Visit 2—All Groups 41
8.1.3. PK and Complement Sample- Day 7 (Select Sites) 42
8.1.3.1. Visit 2A—All Groups (Select Sites) 42
8.1.4. Treatment Phase—24 Months 42
8.1.4.1. Months 1-12 42

8.1.4.2. Months 13-24 43
8.1.5. Early Termination Visit 44
8.1.6. Unscheduled Visits 44
9. ASSESSMENTS 45
9.1. Informed Consent 45
9.2. Demographic Information/Medical/Surgical History 45
9.3. Ocular History/Ocular Procedures 45
9.4. Vital Signs 45
9.5. Physical Examination 45
9.6. Laboratory Analysis of Blood and Urine 45
9.7. Genotyping Samples 46
9.8. Urine Pregnancy Test 46
9.9. Patient-Reported Outcomes 47
9.9.1. The National Eye Institute Visual Functioning Questionnaire 25-Item Version 47
9.9.2. The Functional Reading Independence Index 47
9.10. Best Corrected Visual Acuity and Low Luminance Best Corrected Visual Acuity 47
9.11. Minnesota Low-Vision Reading Test or Radner Reading Charts (in Select Countries) 48
9.12. Home-Based Functional Digital Applications (Optional, in Select Sites) 48
9.13. Complete Ophthalmic Exam 48
9.14. Ocular Imaging 48
9.15. Postinjection Assessment 49
9.16. Blood Volume for Study Assessments 50
9.17. Samples for Clinical Repository (Optional, Select Sites) 50
9.17.1. Approval by the Institutional Review Board or Ethics Committee 51
9.17.2. Sample Collection 51
9.17.3. Confidentiality and Data Ownership 51
9.17.4. Consent to Participate in the Clinical Repository 52
9.17.5. Withdrawal from the Clinical Repository 52
9.17.6. Monitoring and Oversight 52
10. SAFETY EVALUATIONS 53

10.1. Data Monitoring Committee 53
11. ADVERSE EVENTS 54
11.1. Definition 54
11.2. Recording Adverse Events 54
11.3. Reporting Adverse Events 55
11.3.1. Relationship of Events to Study Treatment 55
11.3.2. Severity of Events 56
11.4. Serious Adverse Events 56
11.5. Treatment and Follow-up of Adverse Events 56
11.6. Pregnancy 57
11.7. Disease Progression 57
11.8. Withdrawal 57
12. DATA MANAGEMENT AND STATISTICAL CONSIDERATIONS 58
12.1. Data Collection 58
12.2. Clinical Data Management 58
12.3. Statistical Analysis Process 58
12.4. Planned Interim Analysis and Data Safety Monitoring Committee 58
12.5. Sample Size Calculation and Power Considerations 59
12.6. Statistical Analysis Sets 59
12.7. Efficacy Analyses 60
12.7.1. Primary Efficacy Analysis 60
12.7.2. Secondary Efficacy Analysis 61
12.7.3. Exploratory Efficacy Analysis 62
12.8. Safety Analyses 62
12.8.1. Anti-Therapeutic Antibodies 63
12.8.2. Death 63
12.8.3. Ocular Assessments 63
12.9. Pharmacokinetic Analyses 63
12.10. Handling of Missing Data 63
12.11. Visit Windows 63
13. ETHICS 64
13.1. Ethical Conduct of the Study 64
13.2. Institutional Review Board/Ethic Committee 64

13.3. Subject Information and Consent 64
14. ADMINISTRATIVE CONSIDERATIONS 65
14.1. Direct Access to Source Data/Documents 65
14.2. Quality Control and Quality Assurance 65
14.3. Monitoring 65
14.4. Data Handling and Record Keeping 65
14.5. Protocol Amendments 66
14.6. Report Format 66
14.7. Finance and Insurance 66
14.8. Publication Policy 66
14.9. ClinicalTrials.gov 67
14.10. Termination of Study 67
15. REFERENCES 68
16. APPENDICES 70
APPENDIX A: VISIT SCHEDULE—Monthly Group—Screening, Day 1 Through Month 12 70
APPENDIX B: VISIT SCHEDULE—Monthly Group—Month 13 to Month 24 74
APPENDIX C: VISIT SCHEDULE—Every-Other-Month Group—Screening, Day 1 Through Month 12 77
APPENDIX D: VISIT SCHEDULE—Every-Other-Month Group—Month 13 to Month 24 81
APPENDIX E: NATIONAL EYE INSTITUTE VISUAL FUNCTIONING QUESTIONNAIRE 25-ITEM VERSION 84
APPENDIX F: FUNCTIONAL READING INDEPENDENCE INDEX 101
APPENDIX G: MINNESOTA LOW-VISION READING TEST AND RADNER READING CARDS 102
APPENDIX H: GRADING SCALE FOR ASSESSMENT OF ANTERIOR CHAMBER FLARE OR CELLS 103
APPENDIX I: GRADING SCALE FOR ASSESSMENT OF VITREOUS CELLS 104
APPENDIX J: AMENDMENT HISTORY 105
APPENDIX K: PROTOCOL CHANGES TO BE FOLLOWED DURING COVID-19 RESTRICTIONS 117

LIST OF TABLES

Table 1: Treatment Arms With Approximate Number of Subjects....36
Table 2: Laboratory Sampling and Analysis....46
Table 3: Total Study Blood Volume....50
Table 4: Power to Detect a Difference Among 3 Groups With an Equal Size of 200 Subjects....59

LIST OF FIGURES

Figure 1: Study Schema....40

2. SYNOPSIS

Study Title

A Phase 3, Multi-Center, Randomized, Double-Masked, Sham-Controlled Study to Compare the Efficacy and Safety of Intravitreal Pegcetacoplan Therapy with Sham Injections in Patients with Geographic Atrophy (GA) Secondary to Age-Related Macular Degeneration (AMD)

Protocol Number, Version, and Date

APL2-303/ Amendment 5 / 12 August 2020

Investigational Product, Dose, and Route of Administration

- Pegcetacoplan (also known as APL-2)15 mg/0.1 mL
- Intravitreal (IVT) Injection

Study Arms

Arm	Abbreviation	Randomization
Pegcetacoplan 15 mg/0.1 mL monthly for 24 months	PM	2
Pegcetacoplan 15 mg/0.1 mL every other month for 24 months	PEOM	2
Sham monthly for 24 months	SM	1
Sham every other month for 24 months	SEOM	1

Study Phase and Type

Phase 3, multicenter, randomized, double-masked, sham-injection controlled.

Number of Subjects and Sites

- Approximately 600 subjects
- Approximately 100 sites

Objectives

Primary

To evaluate the efficacy of pegcetacoplan compared to sham injection in patients with geographic atrophy (GA) secondary to age-related macular degeneration (AMD) assessed by change in the total area of GA lesions from baseline as measured by fundus autofluorescence (FAF).

Key Secondary

To evaluate the efficacy of pegcetacoplan compared to sham injection in patients with GA secondary to AMD with respect to:

- Monocular maximum reading speed (study eye), as assessed by Minnesota Low-Vision Reading Test (MNREAD) or Radner Reading Charts (in select countries)
- Functional Reading Independence Index score
- Normal luminance best corrected visual acuity score in the study eye

Secondary

- To evaluate the efficacy of pegcetacoplan compared to sham injection in patients with GA secondary to AMD with respect to:
    - Low luminance best corrected visual acuity score in the study eye
    - Low luminance deficit in the study eye
    - Total area of GA lesion(s) in the study eye
    - Monocular critical print size (study eye), as assessed by MNREAD or Radner Reading Charts (in select countries)
    - National Eye Institute Visual Functioning Questionnaire 25-Item Version (NEI VFQ-25) distance activity subscale score (in select countries)
- To evaluate the pharmacokinetics of pegcetacoplan as assessed by systemic plasma concentration of pegcetacoplan (in select sites)

Safety

- To evaluate the safety and tolerability of pegcetacoplan compared to sham injection in patients with GA secondary to AMD as indicated by:
    - Incidence and severity of ocular and systemic treatment-emergent adverse events
    - Incidence of anti-therapeutic antibodies directed against pegcetacoplan
    - Incidence of new active choroidal neovascularization (CNV) in the study eye

Exploratory

- To evaluate the efficacy of pegcetacoplan compared to sham injection in patients with GA secondary to AMD as indicated by:
    - NEI VFQ-25 composite score
    - NEI VFQ-25 near activity subscale score (in select countries)
    - Comparison between study eye and fellow eye in change in GA lesion size
    - To evaluate the maximum binocular reading speed as assessed by MNREAD or Radner Reading Charts (in select countries)
    - To evaluate the binocular critical print size as assessed by MNREAD or Radner Reading Charts (in select countries)
- To evaluate the relationship between genetic polymorphisms associated with AMD with GA progression and response to pegcetacoplan
- To evaluate the incidence of new onset of subclinical CNV in the study eye
- To assess sensitivity and specificity of a digital reading speed application to detect disease progression/regression (optional, select sites)
- To assess sensitivity and specificity of a digital visual function application to detect disease progression/regression (optional, select sites)

Inclusion Criteria

The study eye must meet all inclusion criteria. If both eyes meet the inclusion criteria, the eye with the worst visual acuity at the screening visit will be designated as the study eye. If both eyes have the same visual acuity, the right eye will be selected as the study eye.

Ocular- specific inclusion criteria apply to the **study eye** only, unless otherwise specified.

1. Age ≥60 years.
2. Normal luminance best corrected visual acuity of 24 letters or better using Early Treatment Diabetic Retinopathy Study (ETDRS) charts (approximately 20/320 Snellen equivalent).
3. Clinical diagnosis of GA of the macula secondary to AMD as determined by the investigator and confirmed by the reading center.
4. The GA lesion must meet the following criteria as determined by the central reading center's assessment of FAF imaging at screening:
   a. Total GA area must be ≥2.5 and ≤17.5 mm^2 (1 and 7 disk areas respectively)
   b. If GA is multifocal, at least 1 focal lesion must be ≥1.25 mm^2 (0.5 disk areas), with the overall aggregate area of GA as specified above in 4a.
   c. The entire GA lesion must be completely visualized on the macula centered image and must be able to be imaged in its entirety and not contiguous with any areas of peripapillary atrophy.
   d. Presence of any pattern of hyperautofluorescence in the junctional zone of GA. Absence of hyperautofluorescence (ie, pattern = none) is exclusionary.[1]
5. Adequate clarity of ocular media, adequate pupillary dilation, and fixation to permit the collection of good quality images as determined by the investigator.
6. Female subjects must be:
   a. Women of non-childbearing potential, or
   b. Women of childbearing potential with a negative serum pregnancy test at screening and must agree to use protocol-defined methods of contraception for the duration of the study and refrain from breastfeeding for the duration of the study.
7. Males with female partners of childbearing potential must agree to use protocol-defined methods of contraception and agree to refrain from donating sperm for the duration of the study.
8. Willing and able to give informed consent and to comply with the study procedures and assessments.

Exclusion Criteria

Ocular specific exclusion criteria apply to the **study eye** only, unless otherwise specified.

1. GA secondary to a condition other than AMD such as Stargardt disease, cone rod dystrophy, or toxic maculopathies like plaquenil maculopathy in either eye.
2. Spherical equivalent of the refractive error demonstrating >6 diopters of myopia or an axial length >26 mm.
3. Any history or active CNV, associated with AMD or any other cause, including any evidence of retinal pigment epithelium rips or evidence of neovascularization anywhere based on spectral domain optical coherence tomography imaging and/or fluorescein angiography as assessed by the reading center.
4. Presence of an active ocular disease that in the opinion of the investigator compromises or confounds visual function, including but not limited to, uveitis, other macular diseases (eg, clinically significant

epiretinal membrane, full thickness macular hole or uncontrolled glaucoma/ocular hypertension). Benign conditions in the opinion of the investigator such as peripheral retina dystrophy are not exclusionary.

5. Intraocular surgery (including lens replacement surgery) within 3 months prior to randomization.
6. History of laser therapy in the macular region.
7. Aphakia or absence of the posterior capsule. Note: Yttrium aluminum garnet laser posterior capsulotomy for posterior capsule opacification done at least 60 days prior to screening is not exclusionary.
8. Any ocular condition other than GA secondary to AMD that may require surgery or medical intervention during the study period or, in the opinion of the investigator, could compromise visual function during the study period.
9. Any contraindication to IVT injection including current ocular or periocular infection.
10. History of prior IVT injection.
11. Prior participation in another interventional clinical study for IVT therapies in either eye (including subjects receiving sham).
12. Prior participation in another interventional clinical study for GA in either eye including investigational oral medication and placebo.
13. Participation in any systemic experimental treatment or any other systemic investigational new drug within 6 weeks or 5 half-lives of the active ingredient (whichever is longer) prior to the start of study treatment. Note: clinical trials solely involving observation, over-the-counter vitamins, supplements, or diets are not exclusionary.
14. Medical or psychiatric conditions that, in the opinion of the investigator, make consistent follow-up over the 24-month treatment period unlikely, or would make the subject an unsafe study candidate.
15. Any screening laboratory value (hematology, serum chemistry or urinalysis) that in the opinion of the investigator is clinically significant and not suitable for study participation.
16. Known hypersensitivity to fluorescein sodium for injection or hypersensitivity to pegcetacoplan or any of the excipients in pegcetacoplan solution.

Study Design

This is a 24-month, Phase 3, multicenter, randomized, double-masked, sham-injection controlled study to assess the efficacy and safety of multiple IVT injections of pegcetacoplan in subjects with GA secondary to AMD.

The study will randomize approximately 600 subjects across approximately 100 multinational sites. Subjects will be screened within 28 days before receiving pegcetacoplan or sham injection. Upon entry into the study, subjects will be assigned a screening number. Subjects who meet all inclusion and none of the exclusion criteria will return to the clinic for randomization and treatment on Visit 2 (Day 1). At this visit, subjects will be randomized 2:2:1:1 to receive pegcetacoplan monthly (PM), pegcetacoplan every other month (PEOM), sham injection monthly (SM), or sham injection every other month (SEOM), respectively. Randomization will be stratified according to GA lesion area at screening (<7.5 mm^2; ≥ 7.5 mm^2), and presence of CNV in the fellow eye.

All subjects will be assessed monthly during the first 12 months regardless of treatment regimen. From Month 12 to Month 24, subjects will follow the outlined visit schedule (Appendix A to Appendix D) based on treatment assignment (ie, subjects in the monthly groups will be assessed monthly while subjects in the every-other-month [EOM] group will be assessed EOM). The last visit in the study will be at Month 24, approximately 30 days

(monthly treatment group) or 60 days (EOM treatment group) after the last visit at which investigational product is administered. At the end of the 24-month study period, subjects will have the option to enroll into a separate open-label study.

Subjects who discontinue study treatment, can continue participation in the study and should be encouraged to return to the clinical site for as many follow-up visits as they can (with the exception of pegcetacoplan/sham administration). Subjects who wish to fully withdraw from the study before Month 24, should be encouraged to complete the early termination visit.

Endpoints and Statistical Analysis:

Endpoints

Primary Efficacy Endpoint

- Change from baseline to Month 12 in total area of GA lesion(s) in the study eye (in mm^2) based on FAF.

Key Secondary Efficacy Endpoints

- Change from baseline in monocular maximum reading speed (study eye), as assessed by MNREAD or Radner Reading Charts at Month 24 (in select countries)
- Change from baseline in Functional Reading Independence Index score, at Month 24.
- Change from baseline in normal luminance best corrected visual acuity score at Month 24 as assessed by ETDRS chart.

Secondary Efficacy Endpoints

- Change from baseline in low luminance best corrected visual acuity score at Month 12 and Month 24 as assessed by ETDRS chart.
- Change from baseline in low luminance deficit at Month 12 and Month 24.
- Change from baseline at each planned assessment in the total area of GA lesion(s) in the study eye (in mm^2) as assessed by FAF.
- Change from baseline in monocular critical print size (study eye), as assessed by MNREAD or Radner Reading Charts, at Month 12 and Month 24 (in select countries).
- Change from baseline in the NEI VFQ-25 distance activity subscale score at Month 12 and Month 24 (in select sites).
- Systemic plasma concentration of pegcetacoplan over time (in select sites).

Exploratory Endpoints

- Change from baseline in NEI VFQ-25 composite score at Month 12 and Month 24.
- Change from baseline in NEI VFQ-25 near activity subscale score at Month 12 and Month 24 (in select countries).
- Comparison between study eye and fellow eye in change in GA lesion size from baseline to Month 12 and Month 24.
- Binocular maximum reading speed as assessed by MNREAD or Radner Reading Charts over time (in select countries).

- Binocular critical print size as assessed by MNREAD or Radner Reading Charts over time (in select countries).
- Relationship between genetic polymorphisms associated with AMD with GA progression and response to pegcetacoplan.
- Incidence of new onset of subclinical CNV in the study eye.
- Assess sensitivity and specificity of a digital reading speed application to detect disease progression/regression (optional, select sites).
- Assess sensitivity and specificity of a digital visual function application to detect disease progression/regression (optional, select sites).

Safety Endpoints

- Incidence and severity of ocular and systemic treatment-emergent adverse events.
- Incidence of anti-therapeutic antibodies directed against pegcetacoplan.
- Incidence of new active CNV in the study eye.

Analysis of Primary Efficacy Endpoint

The primary efficacy endpoint is the change from baseline to Month 12 in the total area of GA lesion(s) in eyes injected with pegcetacoplan, either PM or PEOM, or sham injections. GA lesion area (mm^2) as measured by a quantified central reading center based on FAF images. The primary analysis will be the comparison of pegcetacoplan, either PM or PEOM versus the combined 2 sham arms (the 2 sham arms will be combined into a single ‘control’ group).

The null and alternative hypotheses for the primary efficacy analysis are:

H_0: $\mu_S = \mu_{PM}$ vs H_A: $\mu_S \neq \mu_{PM}$, and

H_0: $\mu_S = \mu_{PEOM}$ vs H_A: $\mu_S \neq \mu_{PEOM}$

Note: Here μ indicates each group’s respective mean change from baseline to Month 12 in GA lesion area for the comparison of the primary endpoint.

A mixed effect model for longitudinal data will be used to analyze the change from baseline in GA lesion area. The model will include treatment, and presence of CNV in the fellow eye as fixed effects; baseline GA lesion area (at screening), time (in months) as a factor; as well as the time × treatment interaction term. All available data up to 12 months will be included in the model for the primary analysis. The mean change from baseline to 12 months will be estimated from the model (ie, LS mean) and compared between each of the pegcetacoplan arms to the sham control. For other time point of interest, LS mean change from baseline will be estimated and compared between treatments. Unstructured variance covariance will be used,

For the analysis of final study data, a similar model including data up to 24 months will be used and LS means at time points of interest will be estimated and compared between treatment.

This study is expected to have an approximately 5 data monitoring committee data reviews. Allocating an alpha level of 0.0001 for each data monitoring committee data review, the alpha level remains for the efficacy analysis at 0.0495 to maintain an overall study alpha of 0.05.

The hypothesis testing strategy for the primary and secondary efficacy endpoints will be based on the Gate-keeping multiple testing procedures controlling for the study wide type I error strongly at 2-sided 0.0495 as follows:

Step 1. The mean GA lesion growth at 12 months will be compared between the PM group and the Control at the α level of 0.0495. If the null hypotheses of no difference between groups in this step is rejected, the testing proceeds to **Step 2** and **Step 3**. If it's not rejected, the testing procedure stops at this step.

Step 2. The mean GA lesion growth at 12 months will be compared between the PEOM group and the Control at the **α1** level. If the null hypotheses of no difference between groups in this step is rejected, the **α1** level will be passed down to **Step 3**. The actual value of **α1** will be specified in the SAP and it will be defined to ensure an adequate power of at least 80% for the comparison in this step.

Step 3. The mean GA lesion growth at 24 months will be compared between the PM group and the Control at the α level of **0.0495** if the null hypotheses are rejected at both **Step 1** and **Step 2**; or at the α level of **(0.0495 – α1)** if **Step 2** testing does not reject the null hypothesis. If the null hypothesis at this step is rejected, the α level used at this step will be passed down to the next step of testing. If it's not rejected, the testing procedure stops at this step.

Step 4. The prioritization and alpha allocation for the remaining secondary endpoints will be specified in the SAP.

The following sensitivity and supportive analyses will be performed to evaluate the robustness of the results from the primary analysis method:

- Analyses will be repeated using the modified intent-to-treat and per-protocol sets
- Primary and secondary endpoints will also be summarized with no pooling of the 2 sham arms. The comparison for pegcetacoplan and sham injection within each dose regimen (ie, PM vs SM and PEOM vs SEOM) will be conducted
- Multiple imputation methods and other sensitivity analyses will be explored, and details will be provided in the SAP

Analysis for Key Secondary and Secondary Endpoint(s)

The key secondary and secondary endpoints will be analyzed in the same fashion as the primary endpoint using mixed effect model. The binary secondary endpoints will be analyzed using Cochran-Mantel- Haenszel test stratified by the randomization stratification factors. Hypothesis testing for 3 key secondary endpoints will be performed in the order specified. The SAP will provide more details on multiplicity adjustment and the approach for alpha spending among the endpoints.

Sample Size Justification

Subjects will be randomized in a 2:2:1:1 ratio to receive treatment with PM, PEOM, SM, or SEOM. The annual growth rate in GA lesion area is expected to have a mean of 1.47, 1.70 and 2.13 mm^2/year for PM, PEOM, and sham-pooled groups, respectively, as estimated from the results of a Phase 2 trial for pegcetacoplan. The standard deviation of the lesion growth is estimated to be 1.50 mm^2 based on the same Phase 2 trial data or 1.25 mm^2 based on natural history data.[26] With a sample size of 200 subjects in each group (ie, a total enrollment of 600 subjects) and the observed annual growth rate in GA lesion area from Phase 2 trial for pegcetacoplan, the study will have 99.2% power to show the difference between PM and sham group and 81.5% power to show the difference between PEOM and sham group using the common standard deviation of 1.5 mm^2 and two-sided alpha of 0.05. The approximation is calculated using PROC POWER one-way analysis of variance, SAS 9.4. The study power is likely larger when utilizing the longitudinal data to model the primary endpoint. The actual study power may also vary

based on the distribution of the stratification factors (ie, lesion area at screening, presence of CNV in fellow eye), and site enrollment.

3. ABBREVIATIONS

Abbreviation	Term
AE	adverse event
AMD	age-related macular degeneration
CH50	classical pathway of complement functional test
CNV	choroidal neovascularization
C_{max}	Maximum concentration occurring at T_{max}
CRF (eCRF)	case report form (electronic CRF) (used interchangeably)
DMC	data monitoring committee
EOM	every other month
ETDRS	Early Treatment Diabetic Retinopathy Study
FAF	fundus autofluorescence
FFA/FA	fundus fluorescein angiography or fluorescein angiography
FRI	Functional Reading Independence Index
GA	geographic atrophy
GCP	Good Clinical Practice
GLP	Good Laboratory Practice
IB	investigator’s brochure
ICF	informed consent form
ICH	International Council for Harmonisation
IEC	independent ethics committee
IOP	intraocular pressure
IP	investigational product
IRB	institutional review board
ITT	intent-to-treat
IV	intravenous
IVT	intravitreal
LL-BCVA	low luminance best corrected visual acuity
MedDRA	Medical Dictionary for Regulatory Activities

MNREAD	Minnesota Low-Vision Reading Test
MOP	manual of procedures
NEI VFQ-25	National Eye Institute Visual Functioning Questionnaire 25-Item Version
NIR	near infrared reflectance
NOEL	no-observable-effect level
NL-BCVA	normal luminance best corrected visual acuity
OCT	optical coherence tomography
OCT-A	optical coherence tomography angiography
PEG	polyethylene glycol
PEG40	polyethylene glycol (40-kDa nominal molecular weight)
PEOM	pegcetacoplan every other month
PI	principal investigator
PK	pharmacokinetics
PM	pegcetacoplan monthly
PP	per-protocol
RPE	retinal pigment epithelium
SAE	serious adverse event
SC	subcutaneous
SC5b-9	soluble terminal complement complex (ie, soluble analog of membrane attack complex)
SD-OCT	spectral domain optical coherence tomography
SEOM	sham every other month
SM	sham monthly
TEAE	treatment-emergent adverse event
T_{max}	time of maximum observed concentration sampled during a dosing interval
$t_{1/2}$	terminal elimination half-life
VEGF	vascular endothelial growth factor
WOCBP	women of childbearing potential

4. INTRODUCTION

4.1. Background

This study is being conducted as part of a series of studies for the clinical development of pegcetacoplan for advanced age-related macular degeneration (AMD) (neovascular AMD and geographic atrophy [GA]). The trial will be conducted in compliance with this protocol, Good Clinical Practice (GCP), and applicable regulatory requirements. The subject population will comprise adult male and female subjects with GA secondary to AMD.

4.1.1. Age-Related Macular Degeneration

Age-related macular degeneration is the leading cause of severe vision loss in people over the age of 65 in the United States and other Western countries.[2] In the United States, about 1.75 million people have the advanced forms of AMD.[3] The early signs of AMD (drusen and pigmentary changes) are common in individuals over age 65 and precede the late stage forms, which are visually devastating. The late stage forms of AMD are classified into either macular neovascularization (neovascular, wet, or exudative AMD) or GA.

Geographic atrophy is a disease characterized by thinning and loss of the retinal pigment epithelium (RPE) and concurrent atrophy of photoreceptors and choriocapillaris.[4,5,6] Clinically, GA is characterized by gradually expanding atrophy leaving islands of dead retinal cells in the back of the eye. Although GA can result in significant visual function deficits in reading, night vision, and dark adaptation, and produce dense, irreversible scotomas in the visual field, the initial decline in visual acuity may be relatively limited if the fovea is spared. When the fovea is involved, GA quickly causes blindness.

Genetic susceptibility has become increasingly recognized as a risk factor and important contributor to AMD. More than 19 genetic polymorphisms have been demonstrated to influence AMD risk, with as many as 5 of these encoded by genes that modulate the complement system. Inflammatory processes, especially those mediated by complement are thought to play a key role in AMD.[5] It is thought that these may contribute to loss of choriocapillaris, photoreceptors, and RPE cells.

GA is responsible for approximately 20% of all cases of legal blindness in North America (ie, best corrected visual acuity 20/200 or worse) with increasing incidence and prevalence owing to a higher life expectancy.[4] While there is treatment for exudative AMD with anti–vascular endothelial growth factor (VEGF) therapies, no approved therapy exists for GA which is usually bilateral and relentlessly progressive. It represents a significant unmet need as it leads to significant visual impairment and affects more than 5 million people worldwide.[13]

An overview of available information regarding pegcetacoplan follows below. Further details can be found in the pegcetacoplan investigator’s brochure (IB).[1]

4.1.2. Pegcetacoplan

Pegcetacoplan is a PEGylated cyclic peptide inhibitor of complement C3. Pegcetacoplan is formed by 2 identical pentadecapeptides (combining a bioactive cyclic tridecapeptide C3–inhibiting moiety and a 2-amino acid linker) covalently coupled to each end of a linear

40-kDa polyethylene glycol (PEG) chain (PEG40). There are 2 peptide moieties per molecule of pegcetacoplan.

The peptide portion of the drug binds to complement C3 and is a broad inhibitor of the complement cascade, a biological process that is part of innate immunity and is involved in multiple inflammatory processes. The PEGylation of the molecule imparts slower clearance from the vitreous humor following administration.

Pegcetacoplan intravitreal (IVT) injection 15 mg/0.1 mL will be provided as a 150 mg/mL sterile solution of pegcetacoplan in stoppered glass vials. Pegcetacoplan is a sterile, isotonic solution in acetate-buffer, pH 5.0, containing trehalose. The drug product is packaged in 2R clear Type I glass vials with 13-mm FluroTec-coated chlorobutyl grey stoppers and sealed with 13-mm aluminum/polypropylene flip-off type seals.

4.1.3. Nonclinical Data

This section is intended to briefly summarize information on the safety, tolerability, and pharmacokinetics (PK) of IVT, intravenous (IV), and subcutaneous (SC) injections of pegcetacoplan. For complete and detailed information, refer to the IB.

As pegcetacoplan is only pharmacologically active in primates, the pivotal IVT toxicological studies have been conducted in cynomolgus monkeys. The safety and tolerability of IVT-administered pegcetacoplan has been assessed in a Good Laboratory Practice (GLP)–compliant pivotal chronic (9-month) repeat-dose study in cynomolgus monkeys. In addition, 2 GLP-compliant 2-month ocular bridging studies (the first comparing drug substance from 2 different contract manufacturing organizations, the second assessing the safety and tolerability profiles of 3 different formulations to support the Phase 3 clinical study) have been conducted, in cynomolgus monkeys as well. Pegcetacoplan was observed to be minimally immunogenic in the 9-month study as evidenced by a lack of circulating antibodies in the majority of monkeys studied. The no-observable-effect level (NOEL) for chronic (9-month) IVT dosing was concluded to be >24.8 mg/eye. The results of this 9-month chronic study in cynomolgus monkeys support the safety of chronic IVT injections of pegcetacoplan at monthly or bimonthly intervals in humans.

Pharmacokinetic assessments included in the chronic (9-month) study revealed serum concentrations of pegcetacoplan that were approximately dose-proportional 24 hours after the first IVT dose. The serum terminal elimination half-life ($t_{1/2}$) of IVT-administered pegcetacoplan, determined in a separate PK assessing a 10 mg/eye dose (in 50 μL), was 10.4 days although the pegcetacoplan levels measured in the serum following infrequent IVT administration are orders of magnitude lower than pharmacological serum levels achieved with SC or IV administration.

Additional nonclinical studies in monkeys and rabbits, including assessments of other routes of administration (SC and IV), have further defined pegcetacoplan's safety profile. Pegcetacoplan has been shown to be generally tolerated through all 3 routes of administration assessed (IVT, SC, and IV), with no adverse effects observed in safety pharmacology studies (in vivo cardiopulmonary telemetry and in vitro hERG inhibition assay) and genotoxicity studies (Ames, in vitro aneugenicity and clastogenicity in TK6 cells, and in vivo clastogenicity in mouse micronuclei).

CCI

[redacted]

CCI

[redacted]

CCI [redacted]

4.1.4. Clinical Data

The initial safety and tolerability of pegcetacoplan following IVT administration in humans was tested in an open-label, single dose escalation, Phase I clinical study in patients with wet AMD under protocol POT-CP043014 (NCT02461771). The study was conducted in multiple ophthalmology clinical sites in the US and Australia. A single dose of pegcetacoplan was administered on Day 1 to patients suffering from wet AMD currently receiving anti-VEGF standard of care. Three escalating doses of pegcetacoplan administered IVT were studied. Three subjects received 4 mg; 3 subjects received 10 mg, and 7 subjects received 20 mg. No serious adverse events (SAEs) or drug-related adverse events (AEs) of concern were observed. It was concluded that administration of a single dose of pegcetacoplan IVT up to 20 mg is safe and well tolerated.

A Phase 2 study (Protocol POT-CP121614; NCT02503332) to assess the safety, tolerability, and evidence of activity of multiple IVT injections of pegcetacoplan in subjects with GA secondary to AMD has been completed. The study was conducted at multiple ophthalmology clinical sites in the US, Australia, and New Zealand. Subjects were randomized in a 2:1:2:1 manner to either receive pegcetacoplan IVT 15 mg monthly for 12 months; sham IVT monthly for 12 months; pegcetacoplan IVT 15 mg every other month (EOM) for 12 months; or sham IVT EOM for 12 months.

This study demonstrated a statistically significant slowing of disease progression at Month 12 at the prespecified alpha of 0.1. Pegcetacoplan administered monthly showed a 29% (*P*=.008) reduction in the rate of GA lesion growth compared to sham, and pegcetacoplan administered EOM showed a 20% (*P*=.067) reduction. Pegcetacoplan has been generally well tolerated. The most frequently reported AEs have been related to the injection procedure (IVT injection), which are commonly found in this type of study. An imbalance in new active choroidal neovascularization (CNV) in subjects treated with pegcetacoplan was observed and the risk of developing new exudation may be increased in subjects with a prior history of neovascular AMD in the fellow eye. One subject in the sham arm, 1/81 (1%) was observed to have developed new active study eye exudation compared with 25/165 (15%) subjects that received pegcetacoplan IVT injections, 26 subjects in total. Of the 25 pegcetacoplan treated subjects with new active CNV, 18 subjects received PM treatment and 7 subjects received pegcetacoplan every other month (PEOM) treatment.

Seventeen of the 25 (68%) subjects that developed new active study eye CNV had a prior history of neovascular AMD in the nonstudy fellow eye. No significant imbalance in history of neovascular AMD in the fellow eye was observed among the 3 arms to explain the imbalance in new exudation observed in study eyes. Visual acuity data did not demonstrate clear differences between subjects developing new study eye exudation compared with those that did not.

A Phase 1b study (Protocol APL2-103; NCT 03777332) to assess the safety of pegcetacoplan in subjects with GA secondary to AMD and low vision is currently ongoing. This study is being conducted at multiple ophthalmology clinical sites across the US. Subjects receive monthly treatment with IVT pegcetacoplan for 24 months.

4.1.5. Rationale

4.1.5.1. Rationale for Pegcetacoplan for Treatment of Geographic Atrophy

The rationale for the use of a complement inhibitor in patients with AMD is based on evidence from both human and animal studies. Human biochemical, genetic, and clinical lines of evidence indicate that the complement system plays a role in the etiology of AMD. Complement components including C3, the membrane attack complex, and complement factor H, are present in drusen and basal laminar deposits in eyes from patients with AMD.[7,8,9,10] Genetic variants of complement factor H,[8,11,12,13,14] C3,[15,16] complement factor I,[17] and other complement components[18,19,20] have been associated with altered risks for the development of both the neovascular and atrophic forms of AMD. Patients with AMD also have signs of systemic complement activation, exhibiting higher serum levels of complement factor B, C3a, C5a, SC5b-9 (soluble terminal complement complex), C3d, and Ba compared to age-matched controls.[21,22,23,24]

4.1.6. Dose Selection

A single dose of 15 mg/0.1 mL injection administered monthly or EOM for 24 months will be tested in this study (see Section 7.3.1). Pegcetacoplan was well tolerated in a panel of animal toxicology studies. A 9-month, repeat-dose GLP study in cynomolgus monkeys was conducted to evaluate the safety of IVT injections of pegcetacoplan at doses up to 24.8 mg/eye at a frequency of 1 injection every 4 weeks.

In this pivotal chronic toxicological study conducted in monkeys, the NOEL was established as >24.8 mg of pegcetacoplan delivered IVT every 4 weeks for 9 months using a 100 µL injection. The volume of the human vitreous is approximately 4 mL, which is approximately 2.7-fold larger than the mean vitreous volume of cynomolgus monkeys, 1.5 mL.[25] Based on the difference in vitreous volume between man and cynomolgus and the NOEL defined in nonhuman primates, the human equivalent dose was determined to be 67 mg/eye every 4 weeks. The dose (15 mg/injection) of pegcetacoplan that will be evaluated in this clinical study is expected to result in drug concentrations approximately 4.5-fold lower that the NOEL observed in cynomolgus monkeys.

To support appropriate assessments of dose response to pegcetacoplan, pegcetacoplan is administered in 2 different frequencies in the study: monthly and EOM.

Physician feedback injecting a 200 mg/mL solution in the Phase I (Study POT-CP043014; NCT02461771) trial confirmed that 150 mg/mL is the highest practical concentration that can be routinely administered, which set the dose of the Phase 2 trial to 15 mg (ie, 0.1 mL of a 150 mg/mL solution). The 15-mg dose given monthly was found to be the most efficacious in the Phase 2 trial. The 15-mg dose given EOM demonstrated slightly less efficacy but offers a reduced treatment burden for patients and physicians.

4.2. Risk/Benefit

The Phase I (Study POT-CP043014; NCT02461771) and Phase 2 (Study POT-CP121614; NCT02503332) studies provide supporting evidence of a positive benefit-risk profile for the use of pegcetacoplan in treating patients with AMD, specifically patients with GA. These preliminary results of an up to 28% reduction of GA progression rate support the evaluation of pegcetacoplan in a Phase 3 study.

The reported safety data from these studies demonstrated an acceptable safety and tolerability profile with no clinically significant safety concerns observed. A total of 178 patients have received at least 1 dose (15 mg/injection) of pegcetacoplan as part of these studies. In the Phase 2 study in patients with GA an imbalance in new exudation in subjects treated with pegcetacoplan was observed. Section 4.1.4 provides a summary of the study design and key results from both studies.

The safety monitoring practices employed by this protocol (complete ophthalmologic exam, IOP monitoring, optical coherence tomography (OCT), fluorescein angiography (FA), vital signs, hematology, serum chemistry, urinalysis, physical exam, vital signs, and AE questioning) are adequate to protect the subjects' safety. There are also risks associated with the ophthalmic procedures required for participants in this study. However, these are all standard procedures that are widely performed in ophthalmology.

In the days following any IVT injection, patients are at risk of developing sterile or infectious intraocular inflammation (eg, endophthalmitis). In recent studies conducted with pegcetacoplan IVT from a single manufacturer, events of transient moderate and severe intraocular inflammation have been observed. Other risks of IVT injection include traumatic cataract, retinal detachment, increased IOP, and hemorrhage.

The amount of blood (see Section 9.16) planned for collection from each subject over the 24 months of the study does not pose an undue risk in this patient population.

There is a potential health benefit for trial participants from receipt of study drug. If efficacious, pegcetacoplan is expected to alter the course of GA and slow its rate of progression.

5. STUDY OBJECTIVES

5.1. Study Objectives

5.1.1. Primary Objective

To evaluate the efficacy of pegcetacoplan compared to sham injection in patients with GA secondary to AMD assessed by change in the total area of GA lesions from baseline as measured by fundus autofluorescence (FAF).

5.1.2. Key Secondary Objectives

To evaluate the efficacy of pegcetacoplan compared to sham injection in patients with GA secondary to AMD with respect to:

- Monocular maximum reading speed (study eye), as assessed by the Minnesota Low-Vision Reading Test (MNREAD) or Radner Reading Charts (in select countries)
- Functional Reading Independence Index (FRI) score
- Normal luminance best corrected visual acuity (NL-BCVA) score in the study eye

5.1.3. Secondary Objectives

- To evaluate the efficacy of pegcetacoplan compared to sham injection in patients with GA secondary to AMD with respect to:
    - Low luminance best corrected visual acuity score (LL-BCVA) in the study eye
    - Low luminance deficit in the study eye
    - Total area of GA lesion(s) in the study eye
    - Monocular critical print size (study eye), as assessed by MNREAD or Radner Reading Charts (in select countries)
    - National Eye Institute Visual Functioning Questionnaire 25-Item Version (NEI VFQ-25) distance activity subscale score (in select countries)
- To evaluate the PK of pegcetacoplan as assessed by systemic plasma concentration of pegcetacoplan (in select sites)

5.1.4. Safety Objectives

- To evaluate the safety and tolerability of pegcetacoplan compared to sham injection in patients with GA secondary to AMD as indicated by:
    - Incidence and severity of ocular and systemic treatment-emergent adverse events (TEAEs)
    - Incidence of anti-therapeutic antibodies directed against pegcetacoplan
    - Incidence of new active CNV in the study eye

5.1.5. Exploratory Objectives

- To evaluate the efficacy of pegcetacoplan compared to sham injection in patients with GA secondary to AMD as indicated by:
    - NEI VFQ-25 composite score
    - NEI VFQ-25 near activity subscale score (in select countries)
    - Comparison between study eye and fellow eye in change in GA lesion size
    - To evaluate the binocular maximum reading speed as assessed by MNREAD or Radner Reading Charts (in select countries)
    - To evaluate the binocular critical print size as assessed by MNREAD or Radner Reading Charts (in select countries)
    - To evaluate the relationship between genetic polymorphisms associated with AMD with GA progression and response to pegcetacoplan
- To evaluate the incidence of new onset of subclinical CNV in the study eye
- To assess sensitivity and specificity of a digital reading speed application to detect disease progression/regression (optional, select sites)
- To assess sensitivity and specificity of a digital visual function application to detect disease progression/regression (optional, select sites).

6. PATIENT POPULATION

The study population includes approximately 600 subjects to be randomized at approximately 100 multinational sites. To participate in the study, subjects must be diagnosed with GA of the macula secondary to AMD in the study eye.

6.1. Patient Selection

6.1.1. Inclusion Criteria

The study eye must meet all inclusion criteria. If both eyes meet the inclusion criteria, the eye with the worst normal luminance visual acuity at the screening visit will be designated as the study eye. If both eyes have the same visual acuity, the right eye will be selected as the study eye.

Ocular-specific inclusion criteria apply to the **study eye** only, unless otherwise specified.

1. Age ≥60 years.
2. Normal luminance best corrected visual acuity of 24 letters or better using Early Treatment Diabetic Retinopathy Study (ETDRS) charts (approximately 20/320 Snellen equivalent).
3. Clinical diagnosis of GA of the macula secondary to AMD as determined by the investigator and confirmed by the reading center.
4. The GA lesion must meet the following criteria as determined by the central reading center's assessment of FAF imaging at screening:
   a. Total GA area must be ≥2.5 and ≤17.5 mm^2 (1 and 7 disk areas respectively).
   b. If GA is multifocal, at least 1 focal lesion must be ≥1.25 mm^2 (0.5 disk areas), with the overall aggregate area of GA as specified above in 4a.
   c. The entire GA lesion must be completely visualized on the macula centered image and must be able to be imaged in its entirety and not contiguous with any areas of peripapillary atrophy.
   d. Presence of any pattern of hyperautofluorescence in the junctional zone of GA. Absence of hyperautofluorescence (ie, pattern = none) is exclusionary.[1]
5. Adequate clarity of ocular media, adequate pupillary dilation, and fixation to permit the collection of good quality images as determined by the investigator
6. Female subjects must be:
   a. Women of non-childbearing potential, or
   b. Women of childbearing potential (WOCBP) with a negative serum pregnancy test at screening and must agree to use protocol-defined methods of contraception for the duration of the study and refrain from breastfeeding for the duration of the study.
7. Males with female partners of childbearing potential must agree to use protocol-defined methods of contraception and agree to refrain from donating sperm for the duration of the study.

8. Willing and able to give informed consent and to comply with the study procedures and assessments.

6.1.2. Exclusion Criteria

Ocular specific exclusion criteria apply to the **study eye** only, unless otherwise specified.

1. GA secondary to a condition other than AMD such as Stargardt disease, cone rod dystrophy, or toxic maculopathies like plaquenil maculopathy in either eye.
2. Spherical equivalent of the refractive error demonstrating >6 diopters of myopia or an axial length >26 mm.
3. Any history or active CNV, associated with AMD or any other cause, including any evidence of RPE rips or evidence of neovascularization anywhere based on SD-OCT imaging and/or FA as assessed by the reading center.
4. Presence of an active ocular disease that in the opinion of the investigator compromises or confounds visual function, including but not limited to, uveitis, other macular diseases (eg, clinically significant epiretinal membrane, full thickness macular hole or uncontrolled glaucoma/ocular hypertension). Benign conditions in the opinion of the investigator such as peripheral retina dystrophy are not exclusionary.
5. Intraocular surgery (including lens replacement surgery) within 3 months prior to randomization.
6. History of laser therapy in the macular region.
7. Aphakia or absence of the posterior capsule. Note: Yttrium aluminum garnet laser posterior capsulotomy for posterior capsule opacification done at least 60 days prior to screening is not exclusionary.
8. Any ocular condition other than GA secondary to AMD that may require surgery or medical intervention during the study period or, in the opinion of the investigator, could compromise visual function during the study period.
9. Any contraindication to IVT injection including current ocular or periocular infection.
10. History of prior IVT injection.
11. Prior participation in another interventional clinical study for IVT therapies in either eye (including subjects receiving sham).
12. Prior participation in another interventional clinical study for GA in either eye including investigational oral medication and placebo.
13. Participation in any systemic experimental treatment or any other systemic investigational new drug including within 6 weeks or 5 half-lives of the active ingredient (whichever is longer) prior to the start of study treatment. Note: clinical trials solely involving observation, over-the-counter vitamins, supplements, or diets are not exclusionary.
14. Medical or psychiatric conditions that, in the opinion of the investigator, make consistent follow-up over the 24-month treatment period unlikely, or would make the subject an unsafe study candidate.

15. Any screening laboratory value (hematology, serum chemistry or urinalysis) that in the opinion of the investigator is clinically significant and not suitable for study participation.
16. Known hypersensitivity to fluorescein sodium for injection or hypersensitivity to pegcetacoplan or any of the excipients in pegcetacoplan solution.

6.2. Women of Childbearing Potential

WOCBP are defined as premenopausal women physiologically capable of becoming pregnant.

6.3. Women of Non-Childbearing Potential

Women of non-childbearing potential are defined as women meeting any of the following criteria:

- Older than 45 years with amenorrhea for >2 years or older than 60 years with amenorrhea for >1 year. Both confirmed by follicle-stimulating hormone and luteinizing hormone levels.
- Has undergone hysterectomy.
- Has undergone bilateral oophorectomy.
- Has undergone bilateral salpingectomy.

6.4. Approved Methods of Contraception

Approved methods of contraception include:

- Combined (estrogen-and progestogen-containing) hormonal contraception associated with inhibition of ovulation:
    - Oral
    - Intravaginal
    - Transdermal
- Progestogen-only hormonal contraception associated with inhibition of ovulation:
    - Oral
    - Injectable
    - Implantable
- Intrauterine device (IUD)
- Intrauterine hormone-releasing system (IUS)
- Bilateral tubal occlusion
- Vasectomized partner *(provided that partner is the sole sexual partner of the WOCBP trial participant and that the vasectomized partner has received medical assessment of the surgical success)*

- Sexual abstinence *(defined as refraining from heterosexual intercourse during the entire period of risk associated with the study treatments). Sexual abstinence is only accepted when it is the preferred and usual lifestyle of the subject.*

Subjects must agree to use an approved method of contraception during the study and 90 days after their last dose of study drug.

6.5. Discontinuation of Subjects

A subject may withdraw from the study at any time for any reason without prejudice to his/her future medical care by the physician or at the institution. The investigator or sponsor may withdraw the subject at any time (eg, in the interest of subject safety). The investigator is encouraged to discuss withdrawal of a subject from treatment with the investigational product (IP) with the medical monitor when possible.

Subjects who discontinue treatment with the IP can continue participation in the study and should be encouraged to return to the clinical site for as many follow-up visits as they can. In the event that a subject terminates early from the study, all early termination procedures should be performed even if they are outside the allowed study window.

The reason for termination, date of stopping treatment with IP, all follow-up information and the total amount of IP administered must be recorded in the case report form (CRF) and source documents.

7. TREATMENT OF SUBJECTS

7.1. Allocation to Treatment

Each subject will be assigned a unique screening number after signing the informed consent. Subjects who complete the study screening assessments and meet all the eligibility criteria will be scheduled to enter the study and randomized on Day 1. As part of the screening process, the reading center will evaluate FAF, OCT, digital color fundus photography, near infrared reflectance (NIR), and FA to provide an objective assessment of subject eligibility. Subjects will be randomized 2:2:1:1 to receive treatment with pegcetacoplan monthly (PM), PEOM, sham injection monthly (SM), or sham injection every other month (SEOM), respectively. Table 1 presents the treatment arms along with the approximate number of subjects and injections per arm.

The randomization scheme will be generated and maintained by the sponsor, or designee. Subject randomization will be stratified by GA lesion area at screening (<7.5 mm^2; ≥ 7.5 mm^2) and presence of CNV in the fellow eye (yes; no). Further details on the randomization procedures will be described in the statistical analysis plan.

7.2. Masking and Minimization of Bias

The intent of masking is to limit the occurrence of conscious and unconscious bias in the conduct and interpretation of the clinical study. Bias could arise from the influence that the knowledge of a specific treatment assignment may have on the recruitment and allocation of subjects, their subsequent care, the assessment of endpoints, the handling of withdrawals, and so on. The essential aim of masking, therefore, is to prevent identification of the treatments by the subject and the masked assessors associated with the conduct of the study until all such opportunities for bias have passed.

This is a double-masked study. Designated masked study site staff (eg, assistant(s), visual acuity technicians, OCT technicians, photographers, technicians administering questionnaires, subjects, reading center personnel, the assigned evaluating physician(s), and the sponsor) will be masked to treatment assignment. However, the treating physician and any associated support staff involved in performing the IVT or sham injections will be unmasked to study treatment. These individuals are not allowed to discuss treatment and/or patient outcome with masked study staff, including the evaluating physician. The principal investigator must be masked to subjects' treatment assignment. To prevent bias in treatment assignment, eligible subjects will be randomized using a web-based randomization system. Documentation will be put in place to avoid unintentional unmasking during the study. All study roles will be clearly documented on the site delegation of authority log and once the roles have been designated and executed, these roles should not be switched during the conduct of the study. In unforeseen circumstances, a site can contact the sponsor to switch a study staff member from the masked role to the unmasked role but not vice versa.

7.2.1. Unmasking

In the event of a medical emergency where the knowledge of subject treatment by masked individuals (eg, the subject or his/her physician) is required, an individual investigator (or designee) will have the ability to unmask the treatment assignment for a specific subject and

share that information with the appropriate parties. All documentation indicating unmasking must be retained with the subject's source documentation in a secure manner. A data monitoring committee (DMC) will be set up to monitor patient safety and review data. The DMC will be provided unmasked safety data but will be masked to efficacy data unless this data is deemed medically necessary. Procedures for DMC unmasking will be documented in a DMC charter.

For regulatory reporting and if required by local regulations, the sponsor will unmask study treatment for all serious, unexpected adverse reactions that are considered to be related to study drug. Subjects who have had their treatment assignment unmasked secondary to a serious or unexpected AEs or medical emergency will no longer receive study treatment. However, they should continue to complete as many of the follow-up visits as possible.

The study unmasking for the primary analysis at 12 months will be limited to the analysis team and personnel only on an as-needed basis. All other personnel in the "masked" role will remain masked until the end of study. A document listing out the roles and responsibilities of the individuals participating in the unmasking analysis will be provided prior to the unmasking.

7.3. Dosage and Administration

7.3.1. Dose Levels and Treatment Arms

After randomization and during the treatment phase beginning at Day 1, all subjects will receive a single dose of 15 mg pegcetacoplan/0.1 mL or sham injection IVT either monthly or EOM depending on treatment designation as presented in Table 1 below.

Table 1: Treatment Arms With Approximate Number of Subjects

Treatment arms
Pegcetacoplan 15 mg/0.1 mL monthly for 24 months (n = approximately 200 subjects; 24 pegcetacoplan injections)
Pegcetacoplan 15 mg/0.1 mL every other month for 24 months (n = approximately 200 subjects; 12 pegcetacoplan injections)
Sham monthly for 24 months (n = approximately 100 subjects; 24 sham injections)
Sham every other month for 24 months (n = approximately 100 subjects; 12 sham injections)

7.3.2. Treatment Administration

Only qualified study staff and those delegated the responsibility of study drug administration on the delegation of authority log should perform this procedure. All staff should be appropriately trained on all procedures prior to performing the procedures. Sites should follow the Visit Schedule for order of procedures and assessments.

Administration of study treatment (pegcetacoplan or sham) can be done on a separate day from the assessment visit if both days fall within the visit window. If this occurs on the randomization visit, then the administration of pegcetacoplan or sham should be done within 3 days of randomization and after approval from the medical monitor. When study treatment administration is on a day other than a study visit, then the only assessment that must be done on the day of study treatment administration is the preinjection IOP.

If a subject falls outside the visit window for a dosing visit, the dose should be skipped and the subject should be scheduled on time for the next dosing visit.

7.3.3. Drug Supplies

7.3.3.1. Identity of Investigational Product

Pegcetacoplan will be supplied as a liquid solution in stoppered glass vials and should be stored according to the label. Specific instructions for preparing pegcetacoplan for the IVT injection procedure will be provided in the manual of procedures. Pegcetacoplan Intravitreal Injection 15 mg/0.1 mL (150 mg/mL) is a sterile, isotonic solution of pegcetacoplan in acetate-buffer, pH 5.0, containing trehalose. The drug product is packaged in 2R clear Type I glass vials with 13-mm FluroTec-coated chlorobutyl grey stoppers and sealed with 13-mm aluminum/polypropylene flip-off type seals.

Sham will be provided as empty stoppered glass vials and should be stored according to the label.

7.3.3.2. Storage

Vials should be automatically stored as per the instructions until ready for use. Each vial should only be used once. Vials should not be shaken and should be protected from sunlight.

7.3.3.3. Accountability

Pegcetacoplan drug product and sham vials will be provided to a designee at the study site and must be stored in a pharmacy or otherwise locked and secured, at the temperature specified on the label. The drug product supply is accessible only to those individuals authorized by the PI. The sponsor will supply sufficient quantities of pegcetacoplan drug product and sham to allow completion of this study. The site should only use the investigational medicinal product provided by the sponsor for use in the study.

Designated unmasked study staff will provide the study treatments to the subjects in accordance with their assigned subject numbers and the randomization schedule. During the study, the receipt of the drugs supplied at the clinical site and of study treatment dispensation for each subject will be documented in drug accountability records. These drug accountability records are to be kept separate from the patient medical records and other source documents.

All used vials should be retained by the clinical site until drug accountability monitoring is performed and then returned to the sponsor or designee or destroyed per sponsor instructions. At the conclusion of the study, any unused IP returned to the sponsor or designee, or destroyed per sponsor instructions, and this will be documented in the drug accountability records.

7.3.4. Intravitreal Pegcetacoplan Administration

Subjects receiving active treatment will be administered 0.1 mL IVT injection of pegcetacoplan according to their treatment designation using a thin wall needle. Detailed instructions on drug preparation, preinjection procedures, administration of pegcetacoplan, and postinjection procedures will be provided in the manual of procedures.

Clinic staff involved in the injection tray assembly, anesthetic preparation, and study drug preparation and administration will follow appropriate aseptic techniques to minimize the risk of potential AEs associated with IVT injections.

Administration of pegcetacoplan is only allowed if preinjection IOP ≤21 mm Hg. If necessary, antiglaucomatous medication can be given to lower the IOP. To minimize transient IOP elevation after IVT injection of pegcetacoplan, decompression of the eye **must** be performed before all pegcetacoplan injections. This is done by applying moderate pressure to the globe with cotton swabs for 30 to 60 seconds during anesthetic preparation.

In addition to the procedures outlined in the protocol and manual of procedures (MOP), adherence to specific institutional policies associated with IVT injections will be observed.

7.4. Sham Injection Administration

The procedure for sham injection will be the same as that used for IVT injection until the actual injection but no actual injection will occur. The injecting physician will only touch the study eye with the blunt end of the syringe. No needle or medication will be injected inside the eye. Detailed instructions on sham injection procedures and postinjection procedures will be provided in the MOP.

Subjects randomized to the monthly or EOM sham injection groups will receive SM or SEOM, respectively. The same assessments will be performed as for the subjects in the pegcetacoplan groups.

7.5. Concomitant Therapies

Any concomitant medications a participant is receiving at the start of the study, within 30 days prior to screening, or that are given for any reason during the study (except for routine medications given for ocular procedures required by the protocol, such as topical anesthetic) must be recorded in the source documents and CRF including start and stop date and time, dose, route, and indication. In addition, all invasive intraocular procedures from the previous 5 years must also be recorded in the source documents and CRF including start and stop dates. Surgical anesthetics, paramedical or alternative therapies (eg, acupuncture, herbal supplements) should also be recorded in the source documents and CRF within 30 days prior to screening.

Metoclopramide or other agents to prevent nausea induced by fluorescein injection may be administered at the discretion of the PI.

7.5.1. Treatment of New Exudation Related to Active Choroidal Neovascularization in the Study Eye and/or Fellow Eye

The suspected onset or presence of new exudation related to active CNV secondary to AMD in the study eye and/or fellow eye must be documented in the source documents and CRF. If the investigator suspects new exudation related to active CNV in the study eye based on fundus examination and/or OCT findings (eg, subretinal fluid, intraretinal fluid, cystoid macular edema, serous pigment epithelial detachment), a FA and optical coherence tomography angiography (OCT-A; select sites only) must also be captured following the imaging protocol procedure. All images, as outlined above, must be sent to the reading center. The reading center will provide a

report indicating whether or not evidence of active, exudative AMD is present or absent, based on the images sent for assessment.

The determination about initiation of anti-VEGF treatment for the exudation related to active CNV is the sole responsibility of the investigator. Treatment with anti-VEGF should start after the report is received by the site from the reading center, with the exception of cases with clear evidence of disease activity (eg; subretinal hemorrhage, extensive subretinal fluid and/or edema, and/or presence of subretinal hyperreflective material) that, in the opinion of the investigator, may have a detrimental visual impact if not treated immediately.

If it is determined that the subject requires anti-VEGF therapy, ranibizumab or aflibercept should be selected and administered by the injecting (unmasked) physician. Ranibizumab should be given monthly and aflibercept EOM after 3 monthly loading doses. The frequency of aflibercept can be changed to monthly if deemed necessary by the investigator, however the physician should refrain from using as-needed treatment (PRN) or treat and extend protocols. Every effort should be made to use the same anti-VEGF therapy for a subject during the course of the study and all treatments should be documented in the CRF.

If anti-VEGF therapy is administered in the study eye on the same day as an pegcetacoplan (or sham) injection, the anti-VEGF therapy shall be administered first and the pegcetacoplan or sham injection shall occur at least 30 minutes after the anti-VEGF injection and only if the IOP is ≤21 mm Hg. Antiglaucomatous medication can be given to lower the IOP to the appropriate range to allow for the pegcetacoplan injection.

In order to avoid potential unmasking, if anti-VEGF and pegcetacoplan are given at the same visit, the unmasked physician must perform both procedures. If the anti-VEGF therapy is given on a separate day from the pegcetacoplan administration, either the masked or the unmasked physician may perform this injection.

Treatment with anti-VEGF is allowed in the fellow eye. The treatments for the fellow eye can be administered according to the site's standard protocol for CNV treatment. Any treatments or therapies administered to the fellow eye within 5 years of screening and while on study should be recorded as a concomitant medication.

7.5.2. Prohibited Therapies

The PI should make a determination regarding patient continuation of therapies used to treat concomitant medical conditions. Therapies as noted in the inclusion/exclusion criteria are prohibited as specified.

7.5.3. Endophthalmitis Treatment

Endophthalmitis cases must be reported as SAEs. The decision to treat a participant for endophthalmitis or suspected endophthalmitis will be guided by the clinical judgment of the investigator and in accordance with local guidelines (as applicable). A culture sample should be performed prior to making a decision on treatment. The treatment method (pars plana vitrectomy vs IVT injection of antibiotics) and choice of antimicrobial agents are also at the discretion of the physician and should follow current standard practice patterns. The decision to use IVT steroids (eg, dexamethasone) for the treatment of endophthalmitis is also at the discretion of the physician.

8. STUDY PROCEDURES

A study schema is presented below in Figure 1. All randomized subjects will return every month to the clinical site for assessments and additional pegcetacoplan or sham injections according to their randomization scheme until Month 12. From Month 12 onwards, subjects will return to the clinical site based on their randomized treatment schedule (monthly or EOM) and will follow the treatment regimen and assessments outlined in the Visit Schedule until Month 24. In addition, subjects will be contacted via phone by masked study staff 4 (±2) days after the first 3 study treatments to collect safety information.

The end of this trial for each subject is defined as when the subject completes their Month 24 study visit, approximately 30 days (monthly treatment group) and approximately 60 days (EOM treatment group) after the last visit at which IP is administered. The period following last dose of IP is sufficient to evaluate the safety of pegcetacoplan based on its half-life in the vitreous; further details can be found in the pegcetacoplan IB. At the end of the 24-month study period, subjects will be offered entry to enroll into a separate open-label study.

Figure 1: Study Schema

Screening (28 ±2 days)	Enrollment/Randomization (Day 1)	Treatment Period (24 Months)

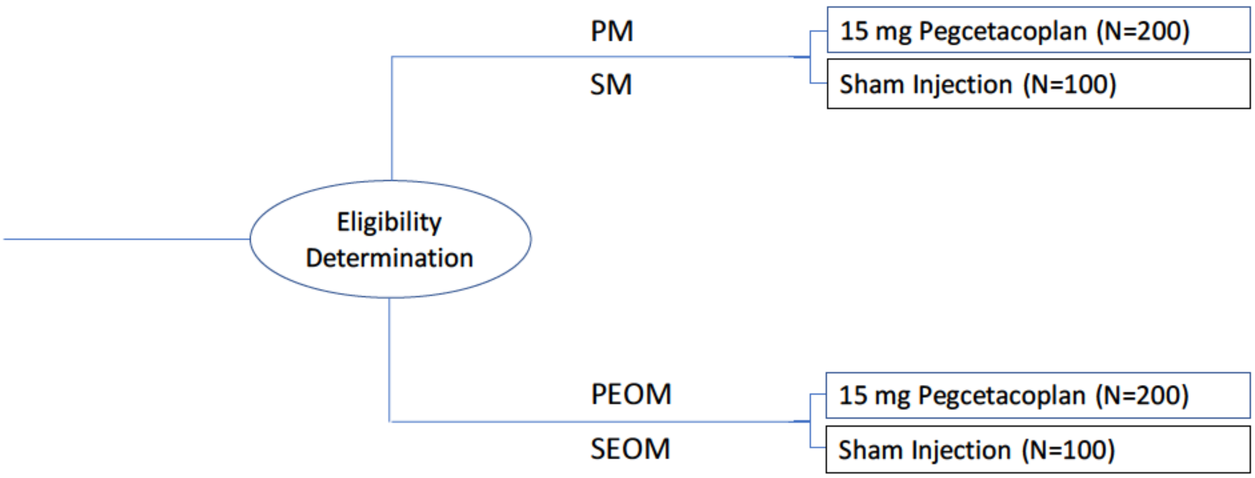

Abbreviations: PM = pegcetacoplan monthly; SM = sham monthly; PEOM = pegcetacoplan every other month; SEOM = sham every other month.

Subjects who discontinue study treatment should return to the clinic for study assessments as per the outlined visit schedule for their assigned treatment arm.

Safety will be assessed throughout the study by a number of evaluations including: monitoring of AEs, preinjection and postinjection monitoring, blood and urine samples will be collected, physical examination, vital signs, and follow-up phone calls will be performed. Blood samples will also be collected for the PK assessment of pegcetacoplan (select sites), anti-therapeutic antibodies, complement profiling (select sites), genotyping, and clinical repository (if the subject consents to this portion).

The planned length of participation in the study for each subject is approximately 25 months (from the beginning of the screening period through completion of the Month 24 visit). After

completion of the 24-month treatment period, subjects will be offered entry into a separate open-label study.

8.1. Study Visit Schedule

Below is a condensed description of the study visits and the procedures and examinations that will be performed. Please refer to the Visit Schedule in Appendix A to Appendix D for a detailed schedule of procedures/assessments for the monthly and EOM visit schedules. Additional safety assessments not listed in this section or the flow chart may be performed if considered necessary at the discretion of the investigator.

8.1.1. Screening Period—Within 28 Days Prior to Randomization/Treatment (Day –28 to Day –1)

8.1.1.1. Visit 1—All Subjects

Note: All ophthalmic procedures (including imaging) are to be performed on both eyes, except where specified.

Before any study-specific procedures are performed, the purpose and nature of the study should be explained and the patient should read, sign, and date the institutional review board/independent ethics committee (IRB/IEC)–approved informed consent form (ICF). The individual obtaining consent from the patient and a witness, if applicable, should also sign and date the ICF. Once the patient has signed the ICF, a screening number should be assigned to the patient. Demographic information, significant medical/surgical history within the previous 5 years, invasive ocular procedures within the previous 5 years, and concomitant medications used within 30 days prior to screening should be collected (including vitamins and all over-the-counter as well as prescription medications). Complete smoking/tobacco history should also be collected.

Subject eligibility should then be determined by reviewing the inclusion/exclusion criteria and the study eye should be selected. Prior to the administration of fluorescein, blood and urine should be collected for safety labs (including blood for human chorionic gonadotropin/follicle-stimulating hormone/luteinizing hormone, if applicable) and vital signs along with a physical examination including weight and height should be performed.

A complete ophthalmic exam including slitlamp exam of the cornea, iris, anterior chamber, aqueous reaction (cells and flare), dilated fundus exam of the vitreous and retina, and IOP measurement. Normal luminance best corrected visual acuity should be performed prior to dilating the eyes. Images should be captured as outlined in the Visit Schedule (FAF, NIR, digital color fundus photography, fundus fluorescein angiography [FFA]) and forwarded to the reading center for determination of eligibility if applicable.

8.1.2. Randomization/Initial Treatment—Day 1—Within 28 Days of Screening

8.1.2.1. Visit 2—All Groups

At this visit, all inclusion/exclusion criteria should be reviewed prior to randomization and dosing, including the determination of eligibility by the reading center. Subjects will be randomized using the interactive web response system. A complete ophthalmic exam including

slitlamp exam of the cornea, iris, anterior chamber, lens, and aqueous reaction (cells and flare), dilated fundus exam of the vitreous and retina, and IOP measurement will be performed and imaging collected as per the Visit Schedule.

All assessments should be performed on the same day. All study visits should be scheduled and projected based on the Day 1 visit date with the exception of Visit 2a which should be based on the Day 1 dose date.

Blood should be drawn for PK and complement profiling (select sites only) and anti-pegcetacoplan antibodies.

Prior to dilating the eyes, all functional tests should be performed (NL-BCVA, LL-BCVA, and MNREAD or Radner Reading Charts [in select countries]). Subjects at select sites will be trained on how to use the home-based digital applications for visual function and reading speed if the subject decides to participate in this portion. This training must occur after completion of all functional tests (NL-BCVA, LL-BCVA). Tests completed using the digital application at select sites (optional) should be completed prior to dilating the eyes but after completion of all functional tests and quality of life measures (NL-BCVA, LL-BCVA, MNREAD or Radner Reading Charts [in select countries], NEI VFQ-25, and FRI). The subject should be instructed to take the electronic device home and to complete the digital application weekly on the same day each week, if possible. The quality of life measures (NEI VFQ-25 and FRI) should be administered by the masked site staff.

Images should be performed including FAF, SD-OCT, OCT-A (select sites), endothelial cell count (select sites), and NIR and sent to the reading center for evaluation.

Study drug or sham injection should be performed by the unmasked physician as described in the MOP and the study eye should be monitored post injection as outlined in Section 9.15. A follow-up phone call should be scheduled with the subject 4 ± 2 days after randomization Day 1 to assess for any AEs.

8.1.3. PK and Complement Sample- Day 7 (Select Sites)

8.1.3.1. Visit 2A—All Groups (Select Sites)

Blood should be drawn for PK and complement profiling (select sites).

8.1.4. Treatment Phase—24 Months

8.1.4.1. Months 1-12

8.1.4.1.1. Visits 3-14 (Monthly and Every-Other-Month Groups)

During this phase, there will be clinic visits every month. Dosing and assessments will occur monthly in the monthly pegcetacoplan and sham injection treatment arms. Dosing will occur EOM in the EOM pegcetacoplan and sham injection treatment arms, however, the subjects will return monthly for assessments (with no dose given).

A complete ophthalmic exam including slitlamp exam of the cornea, iris, anterior chamber, lens, and aqueous reaction (cells and flare), dilated fundus exam of the vitreous and retina, and IOP measurement will be performed and imaging collected as per the Visit Schedule.

Blood will be drawn for safety labs, PK, and complement profile (select sites), Anti-pegcetacoplan antibodies, and genotyping (Month 2 only) prior to the administration of fluorescein as per the Visit Schedule. Samples will be collected for the clinical repository for those subjects that consent to this portion.

Prior to dilating the eyes, all functional tests will be performed (NL-BCVA, LL-BCVA, and MNREAD or Radner Reading Charts [in select countries]). Tests completed using the digital application at select sites (optional) will be completed prior to dilating the eyes but after completion of all functional tests and quality of life measures (NL-BCVA, LL-BCVA, MNREAD or Radner Reading Charts [in select countries], NEI VFQ-25, and FRI). At select sites the subject should be instructed to take the electronic device home and to complete the digital application weekly on the same day each week, if possible. The subject will be instructed to bring back the electronic device for the visits specified in the schedule of events. The quality of life measures (NEI VFQ-25 and FRI) will be administered by the masked site staff.

Imaging will be performed including FAF, FFA, SD-OCT, OCT-A (select sites), endothelial cell count (select sites), and NIR and sent to the reading center for evaluation per the visit schedule.

Study drug or sham injection should be performed by the unmasked physician as described in the MOP and the study eye should be monitored post injection as outlined in Section 9.15. A follow-up phone call should be scheduled with the subject as outlined in the schedule of events.

In the event that a subject is early terminated from the study, all early termination procedures should be performed even if they are outside the allowed study window.

If the subject would like to discontinue dosing but is amenable to continuing in the study, the site should make every effort to have the subject complete as many follow-up visits as possible.

8.1.4.2. Months 13-24

8.1.4.2.1. Visits 15-26 (Monthly Group) and 15-20 (Every-Other-Month Group)

During this phase, clinic visits will follow treatment designation (ie, the monthly subjects will return monthly for dosing and assessments and the EOM subjects will return EOM for dosing and assessments). At select sites, subjects will complete the home-based digital assessments weekly during this period.

A complete ophthalmic exam including slitlamp exam of the cornea, iris, anterior chamber, lens, and aqueous reaction (cells and flare), dilated fundus exam of the vitreous and retina, and IOP measurement will be performed and imaging collected per the Visit Schedule.

Blood should be drawn for safety labs, PK, and complement profile (select sites), anti-pegcetacoplan antibodies, and genotyping, prior to the administration of fluorescein as per the Visit Schedule. Samples will be collected for the clinical repository for those subjects that consented to this portion.

Prior to dilating the eyes, all functional tests should be performed (NL-BCVA, LL-BCVA, and MNREAD or Radner Reading Charts [in select countries]). The quality of life measures (NEI VFQ-25 and FRI) should be administered by the masked site staff.

Images should be performed including FAF, FFA, SD-OCT, OCT-A (select sites), endothelial cell count (select sites), and NIR and sent to the reading center for evaluation per the visit schedule.

IVT (or sham) injection should be performed by the unmasked physician as described in the MOP (noting no injections at Month 24) and the study eye should be monitored post injection as outlined in Section 9.15.

In the event that a subject is early terminated from the study, all early termination procedures should be performed even if they are outside the allowed study window.

If the subject would like to discontinue dosing but is amenable to continuing in the study, the site should make every effort to have the subject complete as many follow-up visits as possible.

The completion of the 24-month study period occurs approximately 30 days (monthly treatment group) and approximately 60 days (EOM treatment group) after the last visit at which IP is administered. The period following last dose of IP is sufficient to evaluate the safety of pegcetacoplan based on its half-life in the vitreous; further details can be found in the pegcetacoplan IB. At the end of the 24-month study period, subjects will have the option to enroll into a separate open-label study.

8.1.5. Early Termination Visit

A list of all assessments to be performed at the early termination visit can be found on the Visit Schedule. All subjects who end the study early for any reason must complete the early termination visit, however all efforts should be made to have the subject return for as many follow-up visits as possible even if dosing does not occur. All ophthalmic procedures are to be performed on **BOTH EYES**.

8.1.6. Unscheduled Visits

If a subject returns to the clinical site before their next scheduled visit for an assessment of an AEs or at the request of the physician, all safety assessments should be performed and any additional assessments as deemed medically necessary by the physician.

9. ASSESSMENTS

The following evaluations will be performed during the study as outlined in the Visit Schedule in Appendix A to Appendix D. Refer to the MOP for detailed descriptions of study-related procedures.

9.1. Informed Consent

Written informed consent for participation must be obtained before performing any study-specific assessments. Informed consent for all subjects should be maintained within the subject source documentation.

9.2. Demographic Information/Medical/Surgical History

Demographic information will be collected from all subjects including but not limited to date of birth, race/ethnicity (where locally permitted). All significant medical conditions and surgeries within the past 5 years should be captured for the subject including chronic and ongoing conditions. Any history or current use of tobacco is to be collected.

9.3. Ocular History/Ocular Procedures

Ocular history within the previous 5 years should be collected and recorded for all subjects. The history should include any significant previous ocular surgeries, procedures and/or medications or treatments used for these conditions.

9.4. Vital Signs

Vital signs consist of body temperature, respiratory rate, blood pressure (systolic and diastolic), and heart rate measurements.

On injection visits, vital signs will be measured prior to dosing. Vital signs should be taken with the patient in a seated position after resting for 5 minutes. Vital signs will be measured before venipuncture.

9.5. Physical Examination

A physical exam will be performed and should include but should not be limited to an evaluation of the eyes, ears, nose, throat, lymph nodes, head, and neurological function. A patient's height and weight should also be measured at screening. If any abnormalities are noted at screening, the PI, or designee, should determine the clinical significance of the finding and whether this will pose any safety risk to the subject. Any changes from baseline should be noted and the clinical significance assessed. Any new, clinically significant, findings should be documented as AEs.

9.6. Laboratory Analysis of Blood and Urine

Collection of blood and urine will occur at the study site and the samples will be shipped to a central laboratory for analysis. All samples (including urine) should be collected prior to treatment and FFA/OCT-A assessments (if applicable). Procedures for the collection and processing of blood and urine are provided in the laboratory manual.

Table 2 presents the laboratory and urine analysis that will be performed.

Table 2: Laboratory Sampling and Analysis

Hematology	Chemistry	Urinalysis	Other
Hemoglobin	Blood urea nitrogen	pH	C3, CH50, AH50
Hematocrit	Creatinine	Specific gravity	Genotyping
Red blood cell count	Bilirubin (total, direct and indirect)	Protein	Anti-pegcetacoplan antibodies
White blood cell count with differential	Albumin	Glucose	HCG[a]
Platelet count	Alkaline phosphatase	Ketones	FSH[b]
	Aspartate aminotransferase	Bilirubin	LH[b]
	Alanine aminotransferase	Blood	
	Creatine kinase	Nitrite	
	Glucose	Urobilinogen	
	Electrolytes (sodium, potassium, chloride, bicarbonate)	Leukocyte esterase	

Abbreviations: AH50 = alternative pathway of complement functional test; CH50 = classical pathway of complement functional test; FSH = follicle-stimulating hormone; HCG = human chorionic gonadotropin; LH = luteinizing hormone.

[a] Serum pregnancy test (ie, HCG) will be performed for females of childbearing potential at screening only.

[b] FSH and LH will be performed for postmenopausal females at screening only.

The principal investigator, or designee, must review the results of the screening Visit clinical laboratory tests (including any retest results) and confirm that these results do not show evidence of any medical condition that would make study participation inappropriate. The principal investigator, or designee, should also assess any changes from baseline at the follow-up visits and the final visit. Clinically significant laboratory values are to be recorded as an AEs.

9.7. Genotyping Samples

The genetic marker sample will be used to evaluate the relationship between genetic polymorphisms associated with AMD with disease progression and response to pegcetacoplan.

A whole-blood sample will be collected for genetic marker analysis. These samples will be collected only for those subjects who consent to this analysis.

These samples will be stored as per the clinical repository guidelines outlined in Section 9.17 if the subject consents to this portion of the study. These samples will be stored up to 15 years after the date of the final closure of the associated clinical database if the patient consented to the clinical repository.

9.8. Urine Pregnancy Test

Urine pregnancy test will be performed in WOCBP only as outlined in the Visit Schedule in Appendix A to Appendix D.

9.9. Patient-Reported Outcomes

Data will be collected via interview-administered questionnaires to assess patient-reported outcomes during scheduled visits as outlined in the Visit Schedule. The questionnaire should be administered by the masked site staff and should be performed prior to any other assessments being performed that day.

Questionnaire data will be used to assess subject-reported efficacy of pegcetacoplan and changes in quality of life over time. Questionnaires will be translated into the appropriate language for each country or region.

9.9.1. The National Eye Institute Visual Functioning Questionnaire 25-Item Version

The NEI-VFQ is an interviewer-administered questionnaire designed to assess patient-reported visual function (Appendix E). The NEI-VFQ is to be administered by the masked staff. It is a 25-item questionnaire with a composite score and covers 12 domains of functional health status and well-being (general health, general vision, ocular pain, near activities (select countries), distance activities (select countries), social functioning, mental health, role difficulties, dependency, driving, color vision, and peripheral vision). Scoring yields 12 subscales based on the 12 domains covered in the questionnaire. These scales are scored from 0 to 100 with higher scores indicating better visual function. The recall period is not specified; however, it is important that the patient uses the same recall period each time the questionnaire is administered throughout their participation in the study.

9.9.2. The Functional Reading Independence Index

The FRI will be interviewer-administered and is an individualized assessment of functional reading independence (Appendix F). The questionnaire has 7 items with 1 total index score. Higher levels on the scale represent higher functional reading independence. The recall period is 7 days.

9.10. Best Corrected Visual Acuity and Low Luminance Best Corrected Visual Acuity

Best corrected visual acuity (including LL-BCVA) will be measured at each visit as per the visit schedule by certified study staff. The study staff performing visual acuity should be masked to the treatment assignment. Best corrected visual acuity testing will be assessed on ETDRS chart starting at a distance of 4 m, performed by a certified visual acuity examiner, and should precede any examination requiring administration of eye drops to dilate the eye or any examination requiring contact with the eye.

The LL-BCVA acuity will be measured by placing a neutral density trial lens causing a reduction of 2.0 log units in luminance. The same requirements apply to measurement of low luminance visual acuity as described above for best corrected visual acuity. Low luminance deficit will be auto-calculated.

A Visual Acuity Specifications procedure manual and training materials will be provided to all sites. All examiners will require certification prior to performing this assessment as part of the study.

9.11. Minnesota Low-Vision Reading Test or Radner Reading Charts (in Select Countries)

The Minnesota Low-Vision Reading Test or Radner Reading Charts (Appendix G) should be administered first monocularly for both eyes and then binocularly. The manual of operations should be referenced for a list of versions that should be administered based on country. These tests should be administered prior to dilating the eyes.

9.12. Home-Based Functional Digital Applications (Optional, in Select Sites)

In addition to in-clinic assessments, visual function and reading speed will also be evaluated using applications on an electronic device. Subjects who decide to participate in this portion will be trained on how to use the digital applications at the Day 1 visit and will complete the assessments using the digital application in the clinic at Day 1; Months 1-3, Month 6, 12, 18, and 24. In-clinic assessments on the digital application should be completed after completion of all functional tests and quality of life measures prior to dilating the eyes (NL-BCVA, LL-BCVA, MNREAD or Radner Reading Charts [in select countries], NEI VFQ-25, and FRI).

Subjects will also take home the electronic device to complete visual function and reading speed assessments weekly beginning at Day 1. Subjects should be instructed to complete the assessments on the same day each week and at approximately the same time of day each week, if possible.

9.13. Complete Ophthalmic Exam

The complete ophthalmic exam will consist of the following:

- External examination of the eye and adnexa.
- Routine screening for eyelids/pupil responsiveness (including ptosis, abnormal pupil shape, unequal pupils, abnormal reaction to light, and afferent pupillary defect).
- Slitlamp examination [cornea, anterior chamber, iris, lens, aqueous reaction (cells and flare). Please see Appendix H for grading scales.
- Dilated fundus exam including evaluation of retina and vitreous (ie, posterior segment abnormalities, retinal hemorrhage/detachment, and vitreal hemorrhage density and vitreous cells). Vitreal hemorrhage density and vitreous cells grading scales are outlined in Appendix I.
- IOP measurement—A measurement of IOP will be conducted using either Tono-Pen or Goldmann applanation tonometer as outlined in the MOP. This should be performed prior to dilating the eyes and the same method should be used for all measurements in the same subject throughout the study.

9.14. Ocular Imaging

The following ocular images will be obtained and sent to the reading center as outlined in the visit schedule. A reading center manual along with training materials will be provided to all sites which will provide information on standardized procedures for the collection, storage, and

transmission of all images. Prior to any images being taken at the site, site personnel must be properly trained and certified and test images and systems and software must be certified and validated by the reading center. Only trained and certified site staff delegated the responsibility of image collection should perform this task. Ocular images obtained as part of this study are:

- digital color fundus photographs
- FA
- SD-OCT imaging
- FAF (Heidelberg Spectralis instrument)
- NIR
- Endothelial cell count (specular microscopy): select sites only
- OCT-A: select sites only

If a patient misses a visit during which ocular images should have been taken, the images should be collected at the next scheduled study visit.

In the event that a subject is suspected to have new active CNV in the study eye and/or the fellow eye, an SD-OCT and FFA using the protocol specified procedures should be performed and sent to the reading center to confirm the diagnosis (Section 7.5.1). In addition, in select sites, OCT-A should also be captured according to the study imaging protocol and sent to the reading center.

9.15. Postinjection Assessment

The study eye will be assessed after the IVT injection of pegcetacoplan or sham to ensure that the injection procedure and/or the study medication have not endangered the health of the eye. The initial postinjection assessments should be done within 5 minutes post injection and include a gross assessment of vision (light perception, hand motion). If the subject passes the gross vision test, he/she can be released from the clinic. If the subject does not pass the gross vision test, IOP must be measured at that time. Additional IOP measurement must be taken approximately every 30 minutes thereafter until IOP ≤30 mm Hg and the subject is able to be released from the clinic.

All subjects receiving an anti-VEGF (ranibizumab or aflibercept) and pegcetacoplan/sham injection on the same day, should have the IOP measured prior to and after the anti-VEGF (prepegcetacoplan). The second injection (pegcetacoplan/sham) can only be given if the IOP ≤21 mm Hg. Antiglaucomatous medication can be given to lower the IOP. The subject can only be released from the clinic if the IOP is ≤30 mm Hg.

Any subject who develops a significant and sustained raise in IOP (>30 mm Hg) after any injection, should be monitored according to the investigator's clinical judgment and may undergo additional procedures and measurements of IOP beyond those specified in the protocol as well as IOP lowering procedures. If any concern or immediate toxicity is noted, the subject will remain at the site and will be treated according to the physician's clinical judgment.

9.16. Blood Volume for Study Assessments

Table 3: Total Study Blood Volume

Assay	Number of time points	Approximate volume per time point * (mL)	Approximate sample volume over course of study (mL)—all groups
Pharmacokinetics[a]	7	4	28
Anti-pegcetacoplan antibodies	9	2	18
*Hematology	8	4	32
**Chemistry (incl. HCG/LH)	8	5	40
FSH/LH (postmenopausal women only)	-	5	-
Genotyping sample[a]	1	8	8
Complement profile (C3, CH50, and AH50)[a]	6	4	24
Clinical repository (select sites and only subjects that consent to clinical repository)	5	14	70
**Total blood volume**			**220[a]**

Abbreviations: AH50 = alternative pathway of complement functional test; CH50 = classical pathway of complement functional test; FSH = follicle-stimulating hormone; HCG = human chorionic gonadotropin; LH = luteinizing hormone.

[a] Represents the standard collection volume planned over the duration of the study, actual volume may vary by group and across sites.

* Volume will vary slightly between regions and analyzing labs based on the standard methodology.

** In some regions, may be included in the chemistry sample based on local methodology.

9.17. Samples for Clinical Repository (Optional, Select Sites)

Apellis intends to apply genomic research across the pegcetacoplan development program to explore how genomic variations may affect the clinical parameters associated with and response to pegcetacoplan. Select sites will be asked to collect additional whole-blood samples and derivatives thereof in a centrally administered facility for the long-term storage of human biologic specimens. The collection and analysis of these specimens will facilitate the rational design of new pharmaceutical agents and the development of diagnostic tests, which may allow for individualized drug therapy for subjects in the future. Specimens for the Genetic Biorepository will be collected from subjects who give specific consent to participate in this optional research only and this will only be done at a select group of sites.

Specimens will be used to achieve the following objectives:

- To study the association of biomarkers with efficacy, AEs, or disease progression
- To increase knowledge and understanding of disease biology

- To study drug response, including drug effects and the processes of drug absorption and disposition
- To develop biomarker or diagnostic assays and establish the performance characteristics of these assays

Future research may suggest other genes, gene categories, proteins, etc. as candidates for influencing not only response to pegcetacoplan but also susceptibility to AMD for which pegcetacoplan may be evaluated. Thus, this additional genomic research may involve the future study of additional unnamed genes or gene categories, but only as they relate to AMD disease susceptibility and drug action.

9.17.1. Approval by the Institutional Review Board or Ethics Committee

Collection and submission of biological samples to the clinical repository is contingent upon the review and approval of the exploratory research and the clinical repository portion of the ICF by each site's IRB or IEC and, if applicable, an appropriate regulatory body. If a site has not been granted approval for clinical repository sampling, this section of the protocol (Section 9.17) will not be applicable at that site.

9.17.2. Sample Collection

The following samples will be collected for research purposes, including but not limited to research on dynamic (noninherited) biomarkers related to AMD and related diseases, pegcetacoplan, and signaling pathways related to AMD and the complement pathway:

- Residual whole-blood clinical genotyping sample
- 14-mL whole-blood sample collected at the specified time points

For all samples, dates of consent and specimen collection should be recorded on the associated clinical repository page of the electronic CRF (eCRF). For sampling procedures, storage conditions, and shipment instructions, see the laboratory manual.

All collected specimens will be destroyed no later than 15 years after the date of final closure of the clinical database. The clinical repository storage period will be in accordance with the IRB/EC-approved ICF and applicable laws (eg, health authority requirements).

9.17.3. Confidentiality and Data Ownership

Patient medical information associated with clinical repository specimens is confidential and may only be disclosed to third parties as permitted by the ICF (or separate authorization for use and disclosure of personal health information) signed by the patient, unless required by law.

Data derived from clinical repository specimen analysis on individual subjects will generally not be provided to the subjects or to study investigators unless required by law. The aggregate results of any research conducted using clinical repository specimens will be available in accordance with the effective Apellis policy on study data publication.

Any inventions and resulting patents, improvements, and/or know-how originating from the use of the clinical repository data will become and remain the exclusive and unburdened property of Apellis, including the right to sell, license, or assign the invention to another entity.

9.17.4. Consent to Participate in the Clinical Repository

The ICF will contain a separate section or a separate ICF that will address participation in the clinical repository. The investigator or authorized designee will explain to each patient the objectives, methods, and potential hazards of participation in the clinical repository. Subjects will be told that they are free to refuse to participate and may withdraw their specimens at any time and for any reason during the storage period. Subjects who decline to participate in the clinical repository can still participate in this clinical study. The investigator should document whether the patient has given consent to participate by completing the clinical repository Research Sample Informed Consent eCRF. In the event of a clinical repository participant's death or loss of competence, the participant's specimens and data will continue to be used as part of the clinical repository research.

9.17.5. Withdrawal from the Clinical Repository

Subjects who give consent to provide clinical repository specimens have the right to withdraw their specimens from the clinical repository at any time for any reason. If a patient wishes to withdraw consent to the testing of his or her specimens, the investigator must inform the sponsor in writing of the patient's wishes using the clinical repository Patient Withdrawal Form and, if the study is ongoing, must enter the date of withdrawal on the Clinical Repository Research Sample Withdrawal of Informed Consent eCRF. The patient will be provided with instructions on how to withdraw consent after the study is closed. A patient's withdrawal from this Study does not, by itself, constitute withdrawal of specimens from the clinical repository. Likewise, a patient's withdrawal from the clinical repository does not constitute withdrawal from this portion of the study.

9.17.6. Monitoring and Oversight

Clinical Repository specimens will be tracked in a manner consistent with GCP by a quality-controlled, auditable, and appropriately validated laboratory information management system to ensure compliance with data confidentiality as well as adherence to authorized use of specimens as specified in this protocol and in the ICF. Apellis monitors and auditors will have direct access to appropriate parts of records relating to patient participation in the clinical repository for the purposes of verifying the data provided to Apellis. The site will permit monitoring, audits, IRB/EC review, and health authority inspections by providing direct access to source data and documents related to the clinical repository samples.

10. SAFETY EVALUATIONS

Any clinically significant abnormalities persisting at the end of the study/early withdrawal or end of the posttreatment phase will be followed by the investigator until resolution or until a clinically stable endpoint is reached. The study includes a number of evaluations to monitor safety including monitoring of AEs, postinjection monitoring, laboratory and urine sampling, physical examination, and vital signs.

10.1. Data Monitoring Committee

An external, independent DMC will be formed with the purpose of reviewing all data across the conduct of the study on an ongoing basis. The DMC will follow a charter that will outline the frequency of meetings and the roles and responsibilities of all members. The DMC will meet at the beginning of the study and approximately every 6 months thereafter and will perform a masked review of all relevant events on an ongoing basis. An ad hoc meeting of the DMC may be convened by the sponsor or the DMC chairperson at any time between the regularly scheduled DMC meetings and data reviews, if warranted by new safety information or for any other reason. The DMC will communicate their recommendations to the sponsor who will notify the appropriate health authorities according to local regulatory requirements.

11. ADVERSE EVENTS

11.1. Definition

An AE is any untoward medical occurrence associated with the use of a drug in humans, whether or not it is considered drug related. An AE can, therefore be any unfavorable and unintended sign, including a clinically significant abnormal laboratory finding, symptom, or disease temporally associated with the use of an IP, whether or not considered related to the IP.

Adverse events can be spontaneously reported by the subject and/or in response to an open question from the study personnel or revealed by observation and will be recorded during the study at the investigational site. All identified AEs must be recorded and described on the appropriate AE or SAE page of the eCRF.

Fluctuating or nonsignificant changes in laboratory values do not necessarily qualify for AE recording but are still collected and recorded via the appropriate eCRF form, if applicable. If these changes in laboratory values are linked to a diagnosis, this diagnosis should be reported as an AE, especially if the diagnosis constitutes an SAE or leads to discontinuation of administration of IP.

11.2. Recording Adverse Events

Adverse events and SAEs will be collected from the signing of the consent form until the last visit or early termination visit, 30 days after the last IP administration for the monthly treatment groups and 60 days after the last IP administration for the EOM treatment groups.

Any events that occur prior to dosing will be categorized as pretreatment events; events occurring after dosing will be recorded as TEAEs (start date of dosing and, therefore, categorization of the event will be dependent on randomization assignment).

For each AE, the investigator will evaluate and report the onset date (and time if applicable), resolution date (and time if applicable), intensity, causality, action taken, serious outcome, and whether or not it caused the subject to discontinue the study.

If possible, the outcome of any AE that caused permanent discontinuation or was present at the end of the study should be reported, particularly if the AE was considered by the investigator to be related to the IP. Subjects experiencing AEs that cause interruption or discontinuation of IP, or those experiencing AEs that are present at the last visit or early termination visit should receive follow-up as appropriate.

All SAEs must be reported to the sponsor/Apellis Safety via eCRF within 24 hours of becoming aware of the event, whether or not the event is deemed treatment-related. If the electronic data capture (EDC) system is not operational (or for paper-based study[ies]), the site must complete the paper SAE form and email to [redacted] immediately and also within 24 hours of becoming aware of the event. The reported information submitted as a paper SAE must be entered into the EDC system once it becomes operational.

Adverse events will be coded in accordance with the Medical Dictionary for Regulatory Activities (MedDRA). If known, the diagnosis of the underlying illness or disorder should be recorded, rather than its individual symptoms.

11.3. Reporting Adverse Events

The sponsor has the responsibility to inform concerned health authorities, ethic committees, and investigators about suspected unexpected serious adverse reactions (SUSARs) in line with GCP guidance and applicable regulatory requirements.

If required, specific SAEs should be reported to the concerned ethic committees in compliance with local requirements.

11.3.1. Relationship of Events to Study Treatment

All AEs that occur during this study will be recorded. The investigator will review each event and assess its relationship to study treatment (definitely related, possibly related, unlikely related, not related, unknown). The date and time of onset, time relationship to drug dosing, duration, and outcome (recovered/resolved, recovered/resolved with sequelae, recovering/resolving, not recovered/not resolved, fatal, or unknown) of each event will be noted.

The following definitions should be considered when evaluating the relationship of AEs and SAEs to the study treatment:

Definitely Related	• Event or laboratory test abnormality, with plausible time relationship to drug intake<br>• Cannot be explained by disease or other drugs<br>• Response to withdrawal plausible (pharmacologically, pathologically)<br>• Event definitive pharmacologically or phenomenologically (ie, an objective and specific medical disorder or a recognized pharmacological phenomenon)<br>• Rechallenge satisfactory, if necessary
Possibly Related	• Event or laboratory test abnormality, with reasonable time relationship to drug intake<br>• Could also be explained by disease or other drugs<br>• Information on drug withdrawal may be lacking or unclear
Unlikely Related	• Event or laboratory test abnormality, with a time to drug intake that makes a relationship improbable (but not impossible)<br>• Disease or other drugs provide plausible explanations
Not Related	• Event or laboratory test abnormality, is plausibly related to the participant's clinical state, underlying disease, or the study procedure/conditions<br>• Time relationship to drug intake makes a relationship unreasonable<br>• Other obvious causes for event or laboratory test abnormality exist
Unknown	• Report suggests an adverse event, however, cannot be judged at this time because information is insufficient or contradictory<br>• More data for proper assessment is needed, or additional data is under examination

11.3.2. Severity of Events

The following definitions should be considered when evaluating the severity of AEs and SAEs:

Mild	Asymptomatic or mild symptoms; clinical or diagnostic observations only; intervention not indicated.
Moderate	Minimal, local or noninvasive intervention indicated; limiting age-appropriate instrumental ADL [a].
Severe	Medically significant but not immediately life-threatening; hospitalization or prolongation of hospitalization indicated; disabling; limiting self-care ADL[b].<br>Note**:** An experience may be severe but may not be serious, eg, severe headache).

A semicolon indicates 'or' within the description of the grade.
Note: Activities of Daily Living (ADL)
[a] Instrumental ADL refer to preparing meals, shopping for groceries or clothes, using the telephone, managing money, etc.
[b] Self-care ADL refer to bathing, dressing and undressing, feeding self, using the toilet, taking medications, and not bedridden.

11.4. Serious Adverse Events

An SAE is any AE or suspected adverse reaction that, in the view of either the investigator or sponsor, results in any of the following outcomes: death; life-threatening; inpatient hospitalization or prolongation of existing hospitalization; a persistent or significant incapacity or substantial disruption of the ability to conduct normal life functions; or a congenital anomaly/birth defect.

Important medical events that may not result in death, be life-threatening*, or require hospitalization may be considered serious when, based upon appropriate medical judgment, they may jeopardize the subject and may require medical or surgical intervention to prevent one of the outcomes listed in the above definition.

Examples of such medical events include allergic bronchospasm requiring intensive treatment in an emergency room or at home; blood dyscrasias or convulsions that do not result in inpatient hospitalization; or the development of drug dependency or drug abuse.

**Life-threatening* is defined as an AE or suspected adverse reaction, which, in the view of either the Investigator or Sponsor places the subject at immediate risk of death as it occurred. It does not include an AE or suspected adverse reaction that, had it occurred in a more severe form, might have caused death.

Unexpected Adverse Event

An AE is considered "unexpected" if it is not listed in the Reference Safety Information section of the IB.

11.5. Treatment and Follow-up of Adverse Events

AEs (whether serious or nonserious), including clinically significant abnormal laboratory test values, will be evaluated by the investigator and treated and/or followed up until the symptoms or value(s) return to baseline or are clinically stable. Treatment of AEs will be performed by appropriately trained medical personnel, either at the clinical site or at a nearby hospital

emergency room. When appropriate, medical tests and/or examinations will be performed to document resolution of the event(s).

AEs continuing after completion of the study will be followed up by telephone or with visits per the discretion of the investigator. If possible, the outcome of any AE that caused discontinuation from the study or was present at the end of the study should be reported, particularly if the AE was considered by the investigator to be related to the study drug.

11.6. Pregnancy

Although pregnancy is not an AE, all pregnancies and suspected pregnancies (including a positive pregnancy test regardless of age or disease state) occurring with a female subject or the female partner of a male subject, must be followed to conclusion to determine their outcome and are considered immediately reportable events.

The pregnancy, suspected pregnancy, or positive pregnancy test must be reported to Apellis Safety within 24 hours of the investigator's awareness using the paper Pregnancy Report Form. The Pregnancy Report Form shall be signed and dated by the investigator and submitted via email to CCI [redacted].

The investigator must follow the subject until completion of the pregnancy and must report the outcome of the pregnancy (eg, delivery, termination, etc.) and neonatal status up to 12 months postdelivery. An abnormal outcome is defined as any pregnancy that results in the birth of a child with persistent or significant incapacity or substantial disruption of the ability to conduct normal life functions (formerly referred to as disabilities), congenital anomalies, or birth defects. In the event of an abnormal outcome, an SAE Report Form will be required.

11.7. Disease Progression

Normal progression or worsening of the medical condition under study (eg, vision loss due to the progression of GA in either eye), by itself, does not necessarily constitute an AEs unless the change can be reasonably attributed to an action of the test article and not only to its lack of efficacy. Disease progression that requires an intervention (eg, administration of IVT anti-VEGF agents for neovascular AMD) should be recorded as an AEs in the eCRF.

11.8. Withdrawal

Participants may choose to discontinue from treatment or to completely withdraw from this study for any reason at any time without penalty or prohibition from enrolling in other clinical protocols.

Participants wishing to withdraw from the study completely will be offered an early termination visit. This early termination visit will include the examinations outlined in Section 8.1.5.

Participants wishing to discontinue treatment but willing to continue with other study procedures, will return to the clinical site for follow-up visits, as per protocol, until Month 24.

Additional information on subject discontinuation is provided in Section 6.5.

12. DATA MANAGEMENT AND STATISTICAL CONSIDERATIONS

12.1. Data Collection

The investigators' authorized site personnel must enter the information required by the protocol on the CRF. A study monitor will visit each site in accordance with the monitoring plan and review the CRF data against the source data for completeness and accuracy. Discrepancies between source data and data entered on the CRF will be addressed by qualified site personnel. When a data discrepancy warrants correction, the correction will be made by authorized site personnel. Data collection procedures will be discussed with the site at the site initiation visit and/or at the investigator's meeting.

12.2. Clinical Data Management

Data are to be entered into a clinical database as specified in the contract research organization's data management plan. Quality control and data validation procedures are applied to ensure the validity and accuracy of the clinical database.

Data are to be reviewed and checked for omissions, errors, and values requiring further clarification using computerized and manual procedures. Data queries requiring clarification are to be communicated to the site for resolution. Only authorized personnel will make corrections to the clinical database, and all corrections are documented in an auditable manner.

12.3. Statistical Analysis Process

The study will be analyzed by the sponsor or its agent.

The statistical analysis plan (SAP) will provide the statistical methods and definitions for the analysis of the efficacy and safety data, as well as describe the approaches to be taken for summarizing other study information such as subject disposition, demographics and baseline characteristics, IP exposure, and prior and concomitant medications. The SAP will also include a description of how missing, unused, and spurious data will be addressed.

To preserve the integrity of the statistical analysis and study conclusions, the SAP will be finalized prior to database lock. All statistical analyses will be performed using SAS (SAS Institute, Cary, NC 27513).

12.4. Planned Interim Analysis and Data Safety Monitoring Committee

No interim analysis is planned for this study. During the study, patient safety will be monitored on a continuous basis by the medical monitor until the last patient completes his or her last scheduled study assessment.

An independent DMC will also be established to provide an ongoing, independent review and assessment of the safety data, and to safeguard the interests and safety of the participating patients in the study. The ongoing review of SAEs and other responsibilities of the DMC will be described in the DMC charter.

The overall Type I (alpha) error rate for the study will be 0.05. To accommodate DMC data reviews, the alpha available for efficacy hypothesis testing will be reduced by 0.0001 for each

DMC review. The study is expected to have 3-4 DMC data reviews prior to the primary analysis of 12-month data and one review afterward.

12.5. Sample Size Calculation and Power Considerations

Subjects will be randomized in a 2:2:1:1 ratio to receive treatment with PM, PEOM, SM, or SEOM. The annual growth rate in GA lesion area is expected to have a mean of 1.47, 1.70, and 2.13 mm^2/year for PM, PEOM, and sham-pooled groups, respectively, as estimated from the results of a Phase 2 trial for pegcetacoplan. The standard deviation of the lesion growth is estimated to be 1.50 mm^2 based on the same Phase 2 trial data or 1.25 mm^2 based on natural history data.[26] The following table provides an approximation of the study power for a sample size of 200 subjects in each group (ie, a total enrollment of 600 subjects) under different alpha values and standard deviations. The approximation is calculated using PROC POWER one-way analysis of variance, SAS 9.4. The study power is likely larger when utilizing the longitudinal data to model the primary endpoint. The actual study power may also vary based on the distribution of the stratification factors (ie, lesion area at screening, presence of CNV in fellow eye), and site enrollment.

Table 4: Power to Detect a Difference Among 3 Groups With an Equal Size of 200 Subjects

		Power for a true mean of 1.47, 1.70, and 2.13 mm^2/year for PM, PEOM, and sham, respectively		
**Common standard deviation (mm^2)**	**Alpha (2-sided)**	**PM vs sham**	**PEOM vs sham**	**Overall (among 3 groups)**
1.25	0.0495	>99.9%	92.9%	99.9%
1.25	0.0248	99.9%	88.2%	99.7%
1.40	0.0495	99.7%	86.5%	99.3%
1.40	0.0248	99.3%	79.4%	98.6%
1.50	0.0495	99.2%	81.5%	98.4%
1.50	0.0248	98.4%	73.1%	97.0%

Abbreviations: PM = pegcetacoplan monthly, PEOM = pegcetacoplan every other month.

12.6. Statistical Analysis Sets

The **safety set** will consist of all subjects who receive any amount of IP.

The **intent-to-treat (ITT) set** will include all randomized subjects. Subjects will be analyzed in the treatment arm assigned at randomization with the 2 sham treatment arms being combined into a single ‘control’ group.

The **modified ITT set** will include all randomized subjects who receive at least 1 injection of pegcetacoplan or sham and have baseline and at least 1 postbaseline value of GA lesion in the study eye as assessed by FAF.

The **per-protocol (PP) set** will include all ITT subjects who follow the protocol without any major deviation(s) that could impact the integrity of the data. A detailed description of the reasons for exclusion from the PP population will be included in the SAP.

12.7. Efficacy Analyses

The primary, secondary, and exploratory efficacy analyses will be performed using the ITT set and presented by treatment group.

12.7.1. Primary Efficacy Analysis

The primary efficacy endpoint is the change from baseline to Month 12 in the total area of GA lesion(s) in eyes injected with pegcetacoplan, either PM or PEOM, or sham injections. GA lesion area (mm^2) as measured by a quantified central reading center based on FAF images. The primary analysis will be the comparison of pegcetacoplan, either PM or PEOM versus the combined 2 sham arms (the 2 sham arms will be combined into a single 'control' group).

The null and alternative hypotheses for the primary efficacy analysis are:

H_0: $\mu_S = \mu_{PM}$ vs H_A: $\mu_S \neq \mu_{PM}$, and

H_0: $\mu_S = \mu_{PEOM}$ vs H_A: $\mu_S \neq \mu_{PEOM}$

Note: Here μ indicates each group's respective mean change from baseline to Month 12 in GA lesion area for the comparison of the primary endpoint.

A mixed effect model for longitudinal data will be used to analyze the change from baseline in GA lesion area. The model will include treatment, and presence of CNV in the fellow eye as fixed effects; baseline GA lesion area (at screening), time (in months) as a factor; as well as the time × treatment interaction term. All available data up to 12 months will be included in the model for the primary analysis. The mean change from baseline to 12 months will be estimated from the model (ie, LS mean) and compared between each of the pegcetacoplan arms to the sham control. For other time point of interest, LS mean change from baseline will be estimated and compared between treatments. Unstructured variance covariance will be used.

For the analysis of final study data, a similar model including data up to 24 months will be used and LS means at time points of interest will be estimated and compared between treatment.

As indicated in an earlier section, the study is expected to have an approximately 5 DMC data reviews. Allocating an alpha level of 0.0001 for each DMC data review, the alpha level remains for the efficacy analysis at 0.0495 to maintain an overall study alpha of 0.05.

The hypothesis testing strategy for the primary and secondary efficacy endpoints will be based on the Gate-keeping multiple testing procedures controlling for the study wide type I error strongly at 2-sided 0.0495 as follows:

Step 1. The mean GA lesion growth at 12 months will be compared between the PM group and the Control at the α level of 0.0495. If the null hypotheses of no difference between groups in this step is rejected, the testing proceeds to **Step 2** and **Step 3**. If it's not rejected, the testing procedure stops at this step.

Step 2. The mean GA lesion growth at 12 months will be compared between the PEOM group and the Control at the **α1** level. If the null hypotheses of no difference between groups in this step is rejected, the **α1** level will be passed down to **Step 3**. The actual value of **α1** will be specified in the SAP and it will be defined to ensure an adequate power of at least 80% for the comparison in this step.

Step 3. The mean GA lesion growth at 24 months will be compared between the PM group and the Control at the α level of **0.0495** if the null hypotheses are rejected at both **Step 1** and **Step 2**; or at the α level of **(0.0495 – α1)** if **Step 2** testing does not reject the null hypothesis. If the null hypothesis at this step is rejected, the α level used at this step will be passed down to the next step of testing. If it's not rejected, the testing procedure stops at this step.

Step 4. The prioritization and alpha allocation for the remaining secondary endpoints will be specified in the SAP.

The following sensitivity and supportive analyses will be performed to evaluate the robustness of the results from the primary analysis method:

- Analyses will be repeated using the modified ITT and PP sets
- Primary and secondary endpoints will also be summarized with no pooling of the 2 sham arms. The comparison for pegcetacoplan and sham injection within each dose regimen (ie, PM vs SM and PEOM vs SEOM) will be conducted
- Multiple imputation methods and other sensitivity analyses will be explored, and details will be provided in the SAP

12.7.2. Secondary Efficacy Analysis

The key secondary and secondary endpoints will be analyzed in the same fashion as the primary endpoint using mixed effect model. The binary secondary endpoints will be analyzed using Cochran-Mantel-Haenszel test stratified by the randomization stratification factors. Hypothesis testing for 3 key secondary endpoints will be performed in the order specified. The SAP will provide more details on multiplicity adjustment and the approach for alpha spending among the endpoints.

Key Secondary Efficacy Endpoints

- Change from baseline in monocular maximum reading speed (study eye), as assessed by MNREAD or Radner Reading Charts at Month 24 (in select countries).
- Change from baseline in FRI score, at Month 24.
- Change from baseline in NL-BCVA at Month 24 as assessed by ETDRS chart.

Secondary Efficacy Endpoints

- Change from baseline in LL-BCVA at Month 12 and Month 24 as assessed by ETDRS chart.
- Change from baseline in low luminance deficit at Month 12 and Month 24.
- Change from baseline at each planned assessment in the total area of GA lesion(s) in the study eye (in mm^2) as assessed by FAF.
- Change from baseline in monocular critical print size (study eye), as assessed by MNREAD or Radner Reading Charts, at Month 12 and Month 24 (in select countries).
- Change from baseline in the NEI VFQ-25 distance activity subscale score at Month 12 and Month 24 (in select countries).

- Systemic Plasma concentration of pegcetacoplan over time (select sites).

12.7.3. Exploratory Efficacy Analysis

Summary statistics will be provided for the following **exploratory endpoints:**

- Change from baseline in NEI VFQ-25 composite score at Month 12 and Month 24.
- Change from baseline in NEI VFQ-25 near activity subscale score at Month 12 and Month 24 (in select countries).
- Comparison between study eye and fellow eye in change in GA lesion size from baseline to Month 12 and Month 24.
- Binocular maximum reading speed as assessed by MNREAD or Radner Reading Charts over time (in select countries).
- Binocular critical print size as assessed by MNREAD or Radner Reading Charts over time (in select countries).
- Relationship between genetic polymorphisms associated with AMD with GA progression and response to pegcetacoplan.
- Incidence of new onset of subclinical CNV in the study eye.
- Assess sensitivity and specificity of a digital reading speed application to detect disease progression/regression (optional, select sites).
- Assess sensitivity and specificity of a digital visual function application to detect disease progression/regression (optional, select sites).

12.8. Safety Analyses

Adverse events will be collected from the time of the first study drug administration until a subject completes the study or discontinues prematurely. Treatment-emergent adverse events are defined as those AEs that develop or worsen after the first dose of study medication and up to 30 days beyond the last dose of study medication. The current version of MedDRA will be used to classify all AEs. Treatment- emergent AEs will be summarized by System Organ Class and Preferred Term, in accordance with the MedDRA coding dictionary. The number of subjects reporting each AE Preferred Term will be tabulated for all TEAEs and separately for those considered as related to study treatment by the investigator or designee. Number of subjects reporting SAEs will also be tabulated. Adverse event summaries will be presented for each treatment group separately.

Adverse events will be summarized by MedDRA coding terms, and separate tabulations also will be produced for related AEs (those considered by the investigator as definitively drug related), SAEs, and discontinuations due to AEs. Vital signs data and findings from physical and ophthalmologic examinations will be tabulated for changes over time on study. Laboratory parameters will be summarized for changes across study by using descriptive statistics. Separate summaries will be prepared for systemic (nonocular) and ocular AEs, with events in the study eye and nonstudy eye summarized separately

12.8.1. Anti-Therapeutic Antibodies

Data on anti-therapeutic antibodies directed against pegcetacoplan will be summarized by the number and percentage of subjects with confirmed positive anti-therapeutic antibodies.

12.8.2. Death

Patient deaths and primary cause of death will be summarized.

12.8.3. Ocular Assessments

Descriptive summaries will be generated for ocular assessments such as visual acuity and IOP.

12.9. Pharmacokinetic Analyses

Pharmacokinetic parameters will be determined from the plasma concentration-time data for

Pegcetacoplan by noncompartmental analysis. The PK endpoints will include but not be limited to the following PK parameters:

- AUC0-tau: Area under the curve for the defined interval between doses
- C_{max}: Maximum concentration occurring at T_{max}
- T_{max}: Time of maximum observed concentration sampled during a dosing interval

Summary statistics (number of observations, mean, standard deviation, coefficient of variation, median, maximum, minimum, and geometric mean) will be determined for all PK parameters by overall and by assessment time. Plasma concentrations at each nominal sampling time will also be summarized using descriptive statistics.

12.10. Handling of Missing Data

All efforts will be made to minimize missing data. A full description of the imputation methods will be provided in the SAP.

12.11. Visit Windows

Analysis visits will be derived with windows for the monthly visits to assess the primary endpoint. Baseline is defined as the date of randomization. If 2 or more treatment visits occur within a window, the closest visit to the target day will be used as that analysis visit; if 2 visits are equidistant from the scheduled analysis visit day, the later analysis visit will be used.

13. ETHICS

13.1. Ethical Conduct of the Study

This research will be carried out in accordance with the protocol, applicable regulations, the ethical principles set forth in the Declaration of Helsinki, and the International Council for Harmonisation (ICH) Harmonized Tripartite Guidance for GCP, E6, R1.

13.2. Institutional Review Board/Ethic Committee

The study protocol, any amendments to the protocol, ICF, the IB, and other study-specific information will be reviewed and approved by the IRB/IEC. The study will not be initiated until the IRB/IEC has approved the protocol or a modification thereof. All records pertaining to IRB/IEC submission and approval should be kept in the site's regulatory files and sponsor's trial master file).

The IRB/IEC must be constituted and operate in accordance with the principles and requirements described in ICH Guidance E6 and national and local regulations as deemed appropriate.

13.3. Subject Information and Consent

The principal investigator, or designee, is responsible for obtaining an informed consent. A written informed consent, in compliance with ICH Guidance E6, must be obtained from each subject at the screening visit, prior to performing any study-related procedures.

The purpose of the study, the procedures to be carried out, and the potential hazards will be described to the subjects in nontechnical terms. The subject will be given sufficient time to consider the study's implications before deciding to participate in the study. The subject and/or legal guardian will be required to sign and date an ICF and will be assured that they may withdraw from the study at any time without jeopardizing their medical care. The PI shall retain the original, signed informed consent for study participation in the subject's medical record and shall provide the subject and/or legal guardian with a copy of the signed consent.

If there are any changes/amendments to the approved protocol, which may directly affect the subject's decision to continue participation in the study, the ICF shall be amended to incorporate the changes to the protocol and the subject must re-sign the IRB/IEC approved amended ICF.

14. ADMINISTRATIVE CONSIDERATIONS

14.1. Direct Access to Source Data/Documents

The principal investigator, or designee, must maintain, at all times, the primary records (ie, source documents) of each subject's data for data verification. Examples of source documents are medical records, laboratory reports, study drug records, and printed CRF pages that are used as the source.

The investigator will permit trial-related monitoring, audits, and inspections by the sponsor and/or its designee, IRB/IEC, and the regulatory agencies at any time during the study. The investigator will ensure that the auditor is allowed direct access to the source data, medical records, eCRFs, and the site's regulatory file for the study and any other pertinent information.

14.2. Quality Control and Quality Assurance

This study is to be performed in full compliance with the protocol, GCP, and applicable regulatory requirements. The principal investigator, Sponsor, and/or its designee are responsible for ensuring that the study staff receive appropriate training on the protocol, study procedures, and any other relevant information.

Quality assurance and quality control systems are implemented and maintained using written Investigative site, sponsor and/or designee standard operating procedures to ensure that the study is conducted and data are generated, documented (recorded), and reported in compliance with the protocol, GCP, and the applicable regulatory requirement(s) and local laws, rules, regulations.

Quality control checks will be applied at each stage of data handling (eg, edit checks) to ensure that all data are reliable and have been processed correctly.

14.3. Monitoring

On-site monitoring will be performed by the sponsor's designee for the duration of the study. The monitor will ensure that the study is conducted, recorded, and reported in accordance with the protocol, standard operating procedures, GCP, and the applicable regulatory requirements. The monitor will verify the accuracy and completeness of the eCRF entries, source documents, and other study-related records against each other. The investigator, or designee, will provide direct access to source data/documents for study-related monitoring. It is important that the investigator and the investigator site staff are available at these visits. The monitor will record the date of each visit together with a summary of the status and progress of the study. Proposed actions will be documented in writing to the investigator.

14.4. Data Handling and Record Keeping

The investigator must maintain all documentation related to this study. All essential documents (as defined in the ICH Guideline E6 and applicable local regulations) and the data generated in connection with this study, together with the original copy of the final report, will be retained for at least 2 years after the last approval of a marketing application in an ICH region and until there are no pending or contemplated marketing applications in an ICH region or at least 2 years have elapsed since the formal discontinuation of clinical development of the IP. These documents

should be retained for a longer period if required by the applicable regulatory requirements or by an agreement with the sponsor.

It is the responsibility of the sponsor to inform the investigator/Institution as to when these documents no longer need to be retained.

14.5. Protocol Amendments

Any amendments to the study protocol deemed necessary as the study progresses will be discussed between sponsor and the investigator. The investigator will not implement any changes to the protocol without an agreement by the sponsor and prior review and documented approval/favorable opinion from the IRB/IEC of an amendment, except where necessary to eliminate immediate hazards to study subject or when the changes involve only logistical or administrative aspects of the study (eg, change in staff, telephone numbers).

Changes resulting in amendments will be made jointly between the sponsor and the investigator and must be confirmed in writing. Amendment(s) will be approved and signed off in the same way as the protocol.

14.6. Report Format

According to the ICH Harmonized Tripartite Guideline (Organization of the Common Technical Document for the Registration of Pharmaceuticals for Human Use M4 and the ICH M2 Expert Working Group), the final report will be written according to the ICH E3 Guideline (Structure and Content of Clinical Study Reports).

14.7. Finance and Insurance

Finance and insurance will be addressed in a clinical trial agreement between the sponsor and the investigator/institution.

14.8. Publication Policy

The data generated for this study are considered confidential information and are the property of the sponsor. All study information provided to the PI and site personnel by the sponsor shall not be published or disclosed to a third party without the prior written consent of the sponsor.

Apellis will endeavor to publish the results of all qualifying, applicable, and covered studies according to external guidelines in a timely manner regardless of whether the outcomes are perceived as positive, neutral, or negative. Additionally, Apellis adheres to external guidelines (eg, Good Publication Practices 2) when forming a publication steering committee, which is done for large, multicenter Phase 2-4 and certain other studies as determined by Apellis. The purpose of the publication steering committee is to act as a noncommercial body that advises or decides on dissemination of scientific study data in accordance with the scope of this policy.

All publications relating to Apellis products or projects must undergo appropriate technical and intellectual property review, with Apellis agreement to publish prior to release of information. The review is aimed at protecting the sponsor's proprietary information existing either at the commencement of the study or generated during the study. To the extent permitted by the publisher and copyright law, the investigator will own (or share with other authors) the copyright

on his/her publications. To the extent that the investigator has such sole, joint, or shared rights, the investigator grants the sponsor a perpetual, irrevocable, royalty-free license to make and distribute copies of such publications.

The term “publication” refers to any public disclosure including original research articles, review articles, oral presentations, abstracts and posters at medical congresses, journal supplements, letters to the editor, invited lectures, opinion pieces, book chapters, electronic postings on medical/scientific websites, or other disclosure of the study results, in printed, electronic, oral, or other form.

Subject to the terms of the paragraph below, the investigator shall have the right to publish the study results, and any background information provided by the sponsor that is necessary to include in any publication of study results, or necessary for other scholars to verify such study results. Notwithstanding the foregoing, no publication that incorporates the sponsor’s confidential information shall be submitted for publication without the sponsor’s prior written agreement to publish and shall be given to the sponsor for review at least 60 days prior to submission for publication. If requested in writing by Apellis, the institution and investigator shall withhold submission of such publication for up to an additional 60 days to allow for filing of a patent application.

If the study is part of a multicenter study, the first publication of the study results shall be made by the sponsor in conjunction with the sponsor’s presentation of a joint, multicenter publication of the compiled and analyzed study results. If such a multicenter publication is not submitted to a journal for publication by the sponsor within an 18-month period after conclusion, abandonment, or termination of the study at all sites, or after the sponsor confirms there shall be no multicenter study publication of the study results, an investigator may individually publish the study results from the specific site in accordance with this section. The investigator must, however, acknowledge in the publication the limitations of the single-site data being presented.

Unless otherwise required by the journal in which the publication appears, or the forum in which it is made, authorship will comply with the International Committee of Medical Journal Editors current standards. Participation as an investigator does not confer any rights to authorship of publications.

14.9. ClinicalTrials.gov

This study will be listed with ClinicalTrials.gov.

14.10. Termination of Study

The sponsor reserves the right to suspend or discontinue this study for administrative and/or safety reasons at any time. The investigator reserves the right to discontinue dosing subjects at any time for safety reasons.

15. REFERENCES

1. Holz FG, Bindewald-Wittich A, Fleckenstein M, et al. Progression of geographic atrophy and impact of fundus autofluorescence patterns in age-related macular degeneration. *American journal of ophthalmology.* 2007;143(3):463-472.

2. Rein DB, Wittenborn JS, Zhang X, et al. Forecasting age-related macular degeneration through the year 2050: the potential impact of new treatments. *Archives of ophthalmology.* 2009;127(4):533-540.

3. Friedman DS, O'Colmain BJ, Munoz B, et al. Prevalence of age-related macular degeneration in the United States. *Archives of ophthalmology.* 2004;122(4):564-572.

4. Holz FG, Strauss EC, Schmitz-Valckenberg S, van Lookeren Campagne M. Geographic atrophy: clinical features and potential therapeutic approaches. *Ophthalmology.* 2014;121(5):1079-1091.

5. Sunness JS, Bressler NM, Tian Y, Alexander J, Applegate CA. Measuring geographic atrophy in advanced age-related macular degeneration. *Investigative ophthalmology & visual science.* 1999;40(8):1761-1769.

6. Sunness JS, Gonzalez-Baron J, Applegate CA, et al. Enlargement of atrophy and visual acuity loss in the geographic atrophy form of age-related macular degeneration. *Ophthalmology.* 1999;106(9):1768-1779.

7. Crabb JW, Miyagi M, Gu X, et al. Drusen proteome analysis: an approach to the etiology of age-related macular degeneration. *Proceedings of the National Academy of Sciences of the United States of America.* 2002;99(23):14682-14687.

8. Hageman GS, Anderson DH, Johnson LV, et al. A common haplotype in the complement regulatory gene factor H (HF1/CFH) predisposes individuals to age-related macular degeneration. *Proceedings of the National Academy of Sciences of the United States of America.* 2005;102(20):7227-7232.

9. Lommatzsch A, Hermans P, Muller KD, Bornfeld N, Bird AC, Pauleikhoff D. Are low inflammatory reactions involved in exudative age-related macular degeneration? Morphological and immunhistochemical analysis of AMD associated with basal deposits. *Graefe's archive for clinical and experimental ophthalmology = Albrecht von Graefes Archiv fur klinische und experimentelle Ophthalmologie.* 2008;246(6):803-810.

10. Mullins RF, Russell SR, Anderson DH, Hageman GS. Drusen associated with aging and age-related macular degeneration contain proteins common to extracellular deposits associated with atherosclerosis, elastosis, amyloidosis, and dense deposit disease. *FASEB journal : official publication of the Federation of American Societies for Experimental Biology.* 2000;14(7):835-846.

11. Edwards AO, Ritter R, 3rd, Abel KJ, Manning A, Panhuysen C, Farrer LA. Complement factor H polymorphism and age-related macular degeneration. *Science.* 2005;308(5720):421-424.

12. Haines JL, Hauser MA, Schmidt S, et al. Complement factor H variant increases the risk of age-related macular degeneration. *Science.* 2005;308(5720):419-421.

13. Klein RJ, Zeiss C, Chew EY, et al. Complement factor H polymorphism in age-related macular degeneration. *Science.* 2005;308(5720):385-389.

14. Maller J, George S, Purcell S, et al. Common variation in three genes, including a noncoding variant in CFH, strongly influences risk of age-related macular degeneration. *Nature genetics.* 2006;38(9):1055-1059.

15. Seddon JM, Yu Y, Miller EC, et al. Rare variants in CFI, C3 and C9 are associated with high risk of advanced age-related macular degeneration. *Nature genetics.* 2013;45(11):1366-1370.

16. Yates JR, Sepp T, Matharu BK, et al. Complement C3 variant and the risk of age-related macular degeneration. *The New England journal of medicine.* 2007;357(6):553-561.

17. van de Ven JP, Nilsson SC, Tan PL, et al. A functional variant in the CFI gene confers a high risk of age-related macular degeneration. *Nature genetics.* 2013;45(7):813-817.

18. Fritsche LG, Chen W, Schu M, et al. Seven new loci associated with age-related macular degeneration. *Nature genetics.* 2013;45(4):433-439, 439e431-432.

19. Gold B, Merriam JE, Zernant J, et al. Variation in factor B (BF) and complement component 2 (C2) genes is associated with age-related macular degeneration. *Nature genetics.* 2006;38(4):458-462.

20. Horie-Inoue K, Inoue S. Genomic aspects of age-related macular degeneration. *Biochemical and biophysical research communications.* 2014;452(2):263-275.

21. Hecker LA, Edwards AO. Genetic control of complement activation in humans and age related macular degeneration. *Advances in experimental medicine and biology.* 2010;703:49-62.

22. Hecker LA, Edwards AO, Ryu E, et al. Genetic control of the alternative pathway of complement in humans and age-related macular degeneration. *Human molecular genetics.* 2010;19(1):209-215.

23. Machalinska A, Dziedziejko V, Mozolewska-Piotrowska K, Karczewicz D, Wiszniewska B, Machalinski B. Elevated plasma levels of C3a complement compound in the exudative form of age-related macular degeneration. *Ophthalmic research.* 2009;42(1):54-59.

24. Scholl HP, Charbel Issa P, Walier M, et al. Systemic complement activation in age-related macular degeneration. *PloS one.* 2008;3(7):e2593.

25. Leeds JM, Henry SP, Bistner S, Scherrill S, Williams K, Levin AA. Pharmacokinetics of an antisense oligonucleotide injected intravitreally in monkeys. *Drug metabolism and disposition: the biological fate of chemicals.* 1998;26(7):670-675.

26. Fleckenstein M, Mitchell P, Freund K.B. et al. The Progression of Geographic Atrophy Secondary to Age-Related Macular Degeneration. *Ophthalmology*. 2018; 125(3):369-390.

16. APPENDICES

APPENDIX A: VISIT SCHEDULE—Monthly Group—Screening, Day 1 Through Month 12

	Screening	Treatment														Early Term[A]
**Visit #**	**1**	**2**	**2A**	**3**	**4**	**5**	**6**	**7**	**8**	**9**	**10**	**11**	**12**	**13**	**14**	
**Day**	**–28 to –1**	**1**	**7**	**30**	**60**	**90**	**120**	**150**	**180**	**210**	**240**	**270**	**300**	**330**	**360**	
**Week**	**0**	**0**	**1**	**4**	**8**	**12**	**16**	**20**	**24**	**28**	**32**	**36**	**40**	**44**	**48**	
**Month**	**0**	**0**	**0**	**1**	**2**	**3**	**4**	**5**	**6**	**7**	**8**	**9**	**10**	**11**	**12**	
**Window (+ or – days)**	**2**	**0**	**1**	**8**	**8**	**8**	**8**	**8**	**8**	**8**	**8**	**8**	**8**	**8**	**8**	
Informed Consent/Assign Screening Number	x															
Demographic Data	x															
Inclusion/Exclusion Criteria [B]	x	x														
Medical/Surgical/Ocular History [C]	x															
Blood Draw—Safety Labs [D,E,F]	x	x			x				x						x	x
Urine Sample Collection [D, E,F]	x	x			x				x						x	x
Urine Pregnancy Test [D,E,F]		x		x	x	x	x	x	x	x	x	x	x	x	x	
Blood Draw—PK and Complement Profile (C3, CH50, AH50) [D, S]		x	x	x					x						x	x
Blood Draw—Genotyping (if applicable) [D]					x											
Blood Draw—Anti-Pegcetacoplan Ab [D]		x		x	x				x						x	x
Blood Draw for Clinical Repository (if applicable)[D,G]					x				x						x	x
Vital Signs [H]	x	x		x	x	x	x	x	x	x	x	x	x	x	x	x
Physical Examination [I]	x														x	x
BCVA [J]	x	x		x	x	x	x	x	x	x	x	x	x	x	x	x
LL-BCVA [J]		x		x	x	x	x	x	x	x	x	x	x	x	x	x
MNREAD or Radner Reading Charts (select countries) [J,K]		x							x						x	x
Slitlamp Examination	x	x		x	x	x	x	x	x	x	x	x	x	x	x	x

APPENDIX A: VISIT SCHEDULE—Monthly Group—Screening, Day 1 Through Month 12 (Continued)

	Screening	Treatment														Early Term[A]
**Visit #**	**1**	**2**	**2A**	**3**	**4**	**5**	**6**	**7**	**8**	**9**	**10**	**11**	**12**	**13**	**14**	
**Day**	**–28 to –1**	**1**	**7**	**30**	**60**	**90**	**120**	**150**	**180**	**210**	**240**	**270**	**300**	**330**	**360**	
**Week**	**0**	**0**	**1**	**4**	**8**	**12**	**16**	**20**	**24**	**28**	**32**	**36**	**40**	**44**	**48**	
**Month**	**0**	**0**	**0**	**1**	**2**	**3**	**4**	**5**	**6**	**7**	**8**	**9**	**10**	**11**	**12**	
**Window (+ or – days)**	**2**	**0**	**1**	**8**	**8**	**8**	**8**	**8**	**8**	**8**	**8**	**8**	**8**	**8**	**8**	
Endothelial Cell Count[S]		x							x						x	x
NEI VFQ-25 [L]		x							x						x	x
FRI [L]		x							x						x	x
Home-Based Digital Applications [L,M S]		x		x	x	x			x						x	
Dilated Indirect Ophthalmoscopy	x	x		x	x	x	x	x	x	x	x	x	x	x	x	x
IOP Measurement	x	x		x	x	x	x	x	x	x	x	x	x	x	x	x
SD-OCT [N]	x	x		x	x	x	x	x	x	x	x	x	x	x	x	x
FAF [N]	x	x			SE		SE		x		SE		SE		x	x
NIR [N]	x	x			SE		SE		x		SE		SE		x	x
DCFP [N]	x														x	x
FFA [N]	x														x	x
OCT-A[R S]		x[R]							x[R]						x[R]	x
Study Eye Determination	x															
Randomization		x														
Pegcetacoplan administration or Sham Injection[T]		x		x	x	x	x	x	x	x	x	x	x	x	x	
Postinjection Assessment [O]		x		x	x	x	x	x	x	x	x	x	x	x	x	
Follow-Up Call [P]		x		x	x	x										
Concomitant Medication/Concomitant Ocular Procedures [Q]	x	x	x	x	x	x	x	x	x	x	x	x	x	x	x	x
Adverse Events	x	x	x	x	x	x	x	x	x	x	x	x	x	x	x	x

APPENDIX A: VISIT SCHEDULE—Monthly Group—Screening, Day 1 Through Month 12 (Continued)

Abbreviations: AH50 = alternative pathway of complement functional test; BCVA = best corrected visual acuity; CH50 = classical pathway of complement functional test; CNV = choroidal neovascularization; DCFP = digital color fundus photography; eCRF = electronic case report form; FAF = fundus autofluorescence; FFA = fundus fluorescein angiography; FRI = Functional Reading Independence Index; IOP = intraocular pressure; LL-BCVA = low luminance best corrected visual acuity; MNREAD = Minnesota Low-Vision Reading Test; NEI VFG-25 = National Eye Institute Visual Functioning Questionnaire 25-Item Version; NIR = near infrared reflectance; OCT-A = optical coherence tomography angiography; PK = pharmacokinetics; SD-OCT = spectral domain optical coherence tomography; SE = study eye; Term = termination; VEGF = vascular endothelial growth factor.

Note: All ocular assessments are to be performed for both eyes unless annotated with 'SE' (study eye) in the above schedule. All assessments should be performed on the same day. All study visits should be scheduled and projected based on the Day 1 visit date with the exception of Visit 2a which should be based on the Day 1 dose date.

A. For subjects that discontinue the study early, the early termination assessments should be performed after a minimum of 30 days have passed from the last dosing visit. If a subject reports for a scheduled visit and decides to terminate early prior to dosing, the visit should be considered the early termination visit and all early termination procedures should be performed. At Month 24, all subjects should be offered entry into an open-label study.

B. At Day 1 (Visit 2), confirm subject eligibility through reviewing the inclusion/exclusion criteria and receive confirmation of eligibility from the reading center.

C. Significant medical/surgical history from the previous 5 years. Anti-VEGF treatments (fellow eye) and invasive ocular procedures performed within the past 5 years and while on study should also be recorded. Any history of tobacco use should be recorded.

D. Obtain prior to fluorescein angiography and before study drug administration.

E. At screening, serum pregnancy should be performed for women of childbearing potential. If positive, subject is not eligible to continue in the study.

F. Beginning at Day 1, perform the urine pregnancy test for women of childbearing potential at each treatment visit. If positive, perform a serum pregnancy test. If serum test is positive, study drug should not be administered and an early term visit should be completed.

G. Only subjects that sign the separate consent for the clinical repository and genotyping will have these samples collected. A 14-mL whole-blood sample will be collected at each of the specified visits.

H. Blood pressure, respiratory rate, heart rate, and temperature. On dosing days, vital signs should be taken predose.

I. Height and weight should be collected at screening.

J. Perform assessments prior to dilating the eyes.

K. In select countries, the MNREAD or Radner Reading Charts should be done during the study visit, prior to dilating the eyes. It should be performed monocularly first, then binocularly.

L. To be administered by the masked site staff prior to any other assessments performed on that day. In-clinic assessments on the digital application should be completed after completion of all functional tests and quality of life measures prior to dilating the eyes.

M. At select sites, and for those subjects who decide to participate, the digital applications will be completed on an electronic device and will consist of assessments for visual function and reading speed. Subjects will receive training at Day 1 on the use of the digital applications and the electronic device and will perform the tests using the application in the clinic at Month 1, Month 2, Month 3, Month 6, Month 12, Month 18, and Month 24. Beginning at Day 1, subjects will complete the functional assessments weekly at home. Subjects should be instructed to complete the assessments on the same day each week and at approximately the same time of day each week, if possible.

N. FAF and SD-OCT images, near infrared reflectance, fluorescein angiograms, and fundus photographs will be performed for the study eye only on days where 'SE' is specified and for both eyes at all other visits as specified in the above schedule and will be sent to the reading center for evaluation (consult the reading center manual for specifics on image capture, processing, and transmission). Images should be captured prior to dosing on dosing days. If a subject misses a study visit or images cannot be obtained at a specific visit, study staff should make every effort to obtain images at the next scheduled visit. If new active CNV is suspected, SD-OCT, FFA, and OCT-A (select sites) images must be collected and sent to the reading center for analysis.

O. Postinjection assessments should be performed within 5 minutes after dosing by the unmasked physician or study staff and should include a gross assessment of vision (finger-counting, hand motion, then light perception when applicable). If subject passes gross vision test, the subject may leave the site. If subject fails gross vision test, the tonometry should be performed. IOP should be ≤30 mm Hg in order for the subject to leave the site. If necessary, antiglaucomatous medication can be given to lower the IOP. If IOP is >30 mm Hg, assessments will continue every approximately 30 minutes from the previous measurement until the subject passes gross vision test and IOP is ≤30 mm Hg. Note: if the study eye is treated with a ranibizumab or aflibercept injection during the same visit as the study treatment (pegcetacoplan or sham), the treatment with ranibizumab or aflibercept must be performed first. The pre- and post- anti-VEGF IOP values must be measured and recorded on the eCRF.

APPENDIX A: VISIT SCHEDULE—Monthly Group—Screening, Day 1 Through Month 12 (Continued)

P. Starting at Day 1, study subjects will be contacted by study site staff within 4 ± 2 days after each study treatment visit (through Month 3) to collect any information on any safety concerns, decrease in vision, eye pain, unusual ocular events, or any new ocular symptoms in the study eye. If the physician determines that there are any safety concerns, a follow-up visit should be scheduled as soon as possible.
Q. Record concomitant medications (ie, prescription and over-the-counter medications) used by the patient within 30 days of screening and throughout the subject's participation in the study.
R. In addition to the time points indicated on the study schedule, OCT-A should be performed at the time of any suspected new active CNV. If new active CNV is confirmed in the study eye, OCT-A should be repeated every 2 months for the study eye.
S. At select sites only.
T. Administration of study treatment (pegcetacoplan or sham) can be done on a separate day from the assessment visit if both days fall within the visit window. If this occurs on the randomization visit, then the administration of pegcetacoplan or sham should be done within 3 days of randomization and after approval from the medical monitor. When study treatment administration is on a day other than a study visit, then the only assessment that must be done on the day of study treatment administration is the preinjection IOP.

APPENDIX B: VISIT SCHEDULE—Monthly Group—Month 13 to Month 24

	Treatment												Early Term [A]
**Visit #**	**15**	**16**	**17**	**18**	**19**	**20**	**21**	**22**	**23**	**24**	**25**	**26**	
**Day**	**390**	**420**	**450**	**480**	**510**	**540**	**570**	**600**	**630**	**660**	**690**	**720**	
**Week**	**52**	**56**	**60**	**64**	**68**	**72**	**76**	**80**	**84**	**88**	**92**	**96**	
**Month**	**13**	**14**	**15**	**16**	**17**	**18**	**19**	**20**	**21**	**22**	**23**	**24**	
**Window (+ or – days)**	**8**	**8**	**8**	**8**	**8**	**8**	**8**	**8**	**8**	**8**	**8**	**8**	
Informed Consent/Assign Screening Number													
Demographic Data													
Inclusion/Exclusion Criteria [B]													
Medical/Surgical/Ocular History [C]													
Blood Draw—Safety Labs [D,E,F]						x						x	x
Urine Sample Collection [D,E,F]						x						x	x
Urine Pregnancy Test [D,E,F]	x	x	x	x	x	x	x	x	x	x	x	x	
Blood Draw—PK and Complement Profile (C3, CH50, AH50)[D,S]												x	x
Blood Draw—Genotyping (if applicable) [D]													
Blood Draw—Anti-Pegcetacoplan Ab [D]		x				x						x	x
Blood Draw for Clinical Repository (if applicable) [D,G]												x	x
Vital Signs [H]	x	x	x	x	x	x	x	x	x	x	x	x	x
Physical Examination [I]												x	x
BCVA [J]	x	x	x	x	x	x	x	x	x	x	x	x	x
LL-BCVA [I]	x	x	x	x	x	x	x	x	x	x	x	x	x
MNREAD or Radner Reading Charts (select countries) [J,K]						x						x	x
Slitlamp Examination	x	x	x	x	x	x	x	x	x	x	x	x	x
Endothelial Cell Count[S]												X	X
NEI VFQ-25 [L]						x						x	x
FRI [L]						x						x	x
Dilated Indirect Ophthalmoscopy	x	x	x	x	x	x	x	x	x	x	x	x	x
Home-Based Digital Applications [L,M,S]						x						x	

APPENDIX B: VISIT SCHEDULE—Monthly Group—Month 13 to Month 24 (Continued)

	Treatment												Early Term [A]
**Visit #**	**15**	**16**	**17**	**18**	**19**	**20**	**21**	**22**	**23**	**24**	**25**	**26**	
**Day**	**390**	**420**	**450**	**480**	**510**	**540**	**570**	**600**	**630**	**660**	**690**	**720**	
**Week**	**52**	**56**	**60**	**64**	**68**	**72**	**76**	**80**	**84**	**88**	**92**	**96**	
**Month**	**13**	**14**	**15**	**16**	**17**	**18**	**19**	**20**	**21**	**22**	**23**	**24**	
**Window (+ or – days)**	**8**	**8**	**8**	**8**	**8**	**8**	**8**	**8**	**8**	**8**	**8**	**8**	
IOP Measurement	x	x	x	x	x	x	x	x	x	x	x	x	x
SD-OCT [N]	x	x	x	x	x	x	x	x	x	x	x	x	x
FAF [N]		SE		SE		x		SE		SE		x	x
NIR [N]		SE		SE		x		SE		SE		x	x
DCFP [N]												x	x
FFA [N]												x	x
OCT-A [R,S]						x [R]						x [R]	x
Study Eye Determination													
Randomization													
Pegcetacoplan administration or Sham Injection [T]	x	x	x	x	x	x	x	x	x	x	x		
Postinjection Assessment [O]	x	x	x	x	x	x	x	x	x	x	x		
Follow-Up Call [P]													
Concomitant Medication/Concomitant Ocular Procedures [Q]	x	x	x	x	x	x	x	x	x	x	x	x	x
Adverse Events	x	x	x	x	x	x	x	x	x	x	x	x	x

Abbreviations: AH50 = alternative pathway of complement functional test; BCVA = best corrected visual acuity; CH50 = classical pathway of complement functional test; CNV = choroidal neovascularization; DCFP = digital color fundus photography; eCRF = electronic case report form; FAF = fundus autofluorescence; FFA = fundus fluorescein angiography; FRI = Functional Reading Independence Index; IOP = intraocular pressure; LL-BCVA = low luminance best corrected visual acuity; MNREAD = Minnesota Low-Vision Reading Test; NEI VFG-25 = National Eye Institute Visual Functioning Questionnaire 25-Item Version; NIR = near infrared reflectance; OCT-A = optical coherence tomography angiography; PK = pharmacokinetics; SD-OCT = spectral domain optical coherence tomography; SE = study eye; Term = termination; VEGF = vascular endothelial growth factor.

Note: All ocular assessments are to be performed for both eyes unless annotated with 'SE' (study eye) in the above schedule. All assessments should be performed on the same day. All study visits should be scheduled and projected based on the Day 1 visit date with the exception of Visit 2a which should be based on the Day 1 dose date.

A. For subjects that discontinue the study early, the early termination assessments should be performed after a minimum of 30 days have passed from the last dosing visit. If a subject reports for a scheduled visit and decides to terminate early prior to dosing, the visit should be considered the early termination visit and all early termination procedures should be performed. At Month 24, all subjects should be offered entry into an open-label study.

B. At Day 1 (Visit 2), confirm subject eligibility through reviewing the inclusion/exclusion criteria and receive confirmation of eligibility from the reading center.

APPENDIX B: VISIT SCHEDULE—Monthly Group—Month 13 to Month 24 (Continued)

C. Significant medical/surgical history from the previous 5 years. Anti-VEGF treatments (fellow eye) and invasive ocular procedures performed within the past 5 years and while on study should also be recorded. Any history of tobacco use should be recorded.
D. Obtain prior to fluorescein angiography and before study drug administration.
E. At screening, serum pregnancy should be performed for women of childbearing potential. If positive, subject is not eligible to continue in the study.
F. Beginning at Day 1, perform the urine pregnancy test for women of childbearing potential at each treatment visit. If positive, perform a serum pregnancy test. If serum test is positive, study drug should not be administered and an early term visit should be completed.
G. Only subjects that sign the separate consent for the clinical repository and genotyping will have these samples collected. A 14-mL whole-blood sample will be collected at each of the specified visits.
H. Blood pressure, respiratory rate, heart rate, and temperature. On dosing days, vital signs should be taken predose.
I. Height and weight should be measured at screening.
J. Perform assessments prior to dilating the eyes.
K. In select countries, the MNREAD or Radner Reading Charts should be done during the study visit, prior to dilating the eyes. It should be performed monocularly first, then binocularly.
L. To be administered by the masked site staff prior to any other assessments performed on that day. In-clinic assessments on the digital application should be completed after completion of all functional tests and quality of life measures prior to dilating the eyes.
M. At select sites, and for those subjects who decide to participate, the digital applications will be completed on an electronic device and will consist of assessments for visual function and reading speed. Subjects will receive training at Day 1 on the use of the digital applications and the electronic device and will perform the tests using the application in the clinic at Month 1, Month 2, Month 3, Month 6, Month 12, Month 18, and Month 24. Beginning at Day 1, subjects will complete the functional assessments weekly at home. Subjects should be instructed to complete the assessments on the same day each week and at approximately the same time of day each week, if possible.
N. FAF and SD-OCT images, near infrared reflectance, fluorescein angiograms, and fundus photographs will be performed for the study eye only on days where 'SE' is specified and for both eyes at all other visits as specified in the above schedule and will be sent to the reading center for evaluation (consult the reading center manual for specifics on image capture, processing, and transmission). Images should be captured prior to dosing on dosing days. If a subject misses a study visit or images cannot be obtained at a specific visit, study staff should make every effort to obtain images at the next scheduled visit. If new active CNV is suspected, SD-OCT, FFA, and OCT-A (selected sites) images should be collected and sent to the reading center for analysis.
O. Postinjection assessments should be performed within 5 minutes after dosing by the unmasked physician or study staff and should include a gross assessment of vision (finger-counting, hand motion, then light perception when applicable). If subject passes gross vision test, the subject may leave the site. If subject fails gross vision test, the tonometry should be performed. IOP should be ≤30 mm Hg in order for the subject to leave the site. If necessary, antiglaucomatous medication can be given to lower the IOP. If IOP is >30 mm Hg, assessments will continue every approximately 30 minutes from the previous measurement until the subject passes gross vision test and IOP is ≤30 mm Hg. Note: if the study eye is treated with a ranibizumab or aflibercept injection during the same visit as the study treatment (pegcetacoplan or sham), the treatment with ranibizumab or aflibercept must be performed first. The pre- and post- anti-VEGF IOP values must be measured and recorded on the eCRF.
P. Starting at Day 1, study subjects will be contacted by study site staff within 4 ± 2 days after each study treatment visit (through Month 3) to collect any information on any safety concerns, decrease in vision, eye pain, unusual ocular events, or any new ocular symptoms in the study eye. If the physician determines that there are any safety concerns, a follow-up visit should be scheduled as soon as possible.
Q. Record concomitant medications (ie, prescription and over-the-counter medications) used by the patient within 30 days of screening and throughout the subject's participation in the study.
R. In addition to the time points indicated on the study schedule, OCT-A should be performed at the time of any suspected new active CNV. If new active CNV is confirmed in the study eye, OCT-A should be repeated every 2 months for the study eye.
S. At select sites only.
T. Administration of study treatment (pegcetacoplan or sham) can be done on a separate day from the assessment visit if both days fall within the visit window. If this occurs on the randomization visit, then the administration of pegcetacoplan or sham should be done within 3 days of randomization and after approval from the medical monitor. When study treatment administration is on a day other than a study visit, then the only assessment that must be done on the day of study treatment administration is the preinjection IOP.

APPENDIX C: VISIT SCHEDULE—Every-Other-Month Group—Screening, Day 1 Through Month 12

	Screening	Treatment														Early Term[A]
**Visit #**	1	2	2A	3	4	5	6	7	8	9	10	11	12	13	14	
**Day**	–28 to –1	1	7	30	60	90	120	150	180	210	240	270	300	330	360	
**Week**	0	0	1	4	8	12	16	20	24	28	32	36	40	44	48	
**Month**	0	0	0	1	2	3	4	5	6	7	8	9	10	11	12	
**Window (+ or – days)**	2	0	1	8	8	8	8	8	8	8	8	8	8	8	8	
Informed Consent/Assign Screening Number	x															
Demographic Data	x															
Inclusion/Exclusion Criteria [B]	x	x														
Medical/Surgical/Ocular History [C]	x															
Blood Draw—Safety Labs [D,E,F]	x	x			x				x						x	x
Urine Sample Collection [D,E,F]	x	x			x				x						x	x
Urine Pregnancy Test [D,E,F]		x			x		x		x		x		x		x	
Blood Draw—PK and Complement Profile (C3, CH50, AH50) [D,S]		x	x	x					x						x	x
Blood Draw—Genotyping (if applicable) [D]					x											
Blood Draw- Anti-Pegcetacoplan Ab [D]		x		x	x				x						x	x
Blood Draw for Clinical Repository (if applicable) [D,G]					x				x						x	x
Vital Signs [H]	x	x		x	x	x	x	x	x	x	x	x	x	x	x	x
Physical Examination [I]	x														x	x
BCVA [J]	x	x		x	x	x	x	x	x	x	x	x	x	x	x	x
LL-BCVA [J]		x		x	x	x	x	x	x	x	x	x	x	x	x	x
MNREAD or Radner Reading Charts (select countries) [J,K]		x							x						x	x
Slitlamp Examination	x	x		x	x	x	x	x	x	x	x	x	x	x	x	x
Endothelial Cell Count[S]		x							x						x	x
NEI VFQ-25 [L]		x							x						x	x
FRI [L]		x							x						x	x
Home-Based Digital Applications [L,M,S]		x		x	x	x			x						x	
Dilated Indirect Ophthalmoscopy	x	x		x	x	x	x	x	x	x	x	x	x	x	x	x

APPENDIX C: VISIT SCHEDULE—Every-Other-Month Group—Screening, Day 1 Through Month 12 (Continued)

	Screening	Treatment														Early Term[A]
Visit #	1	2	2A	3	4	5	6	7	8	9	10	11	12	13	14	
Day	–28 to –1	1	7	30	60	90	120	150	180	210	240	270	300	330	360	
Week	0	0	1	4	8	12	16	20	24	28	32	36	40	44	48	
Month	0	0	0	1	2	3	4	5	6	7	8	9	10	11	12	
Window (+ or – days)	2	0	1	8	8	8	8	8	8	8	8	8	8	8	8	
IOP Measurement	x	x		x	x	x	x	x	x	x	x	x	x	x	x	x
SD-OCT [N]	x	x		x	x	x	x	x	x	x	x	x	x	x	x	x
FAF [N]	x	x			SE		SE		x		SE		SE		x	x
NIR [N]	x	x			SE		SE		x		SE		SE		x	x
DCFP [N]	x														x	x
FFA [N]	x														x	x
OCT-A[R, S]		x[R]							x[R]						x[R]	x
Study Eye Determination	x															
Randomization		x														
Pegcetacoplan administration or Sham Injection[T]		x			x		x		x		x		x		x	x
Postinjection Assessment [O]		x			x		x		x		x		x		x	x
Follow-Up Call [P]		x			x		x									
Concomitant Medication/Concomitant Ocular Procedures [Q]	x	x	x	x	x	x	x	x	x	x	x	x	x	x	x	x
Adverse Events	x	x	x	x	x	x	x	x	x	x	x	x	x	x	x	x

Abbreviations: AH50 = alternative pathway of complement functional test; BCVA = best corrected visual acuity; CH50 = classical pathway of complement functional test; CNV = choroidal neovascularization; DCFP = digital color fundus photography; eCRF = electronic case report form; FAF = fundus autofluorescence; FFA = fundus fluorescein angiography; FRI = Functional Reading Independence Index; IOP = intraocular pressure; LL-BCVA = low luminance best corrected visual acuity; MNREAD = Minnesota Low-Vision Reading Test; NEI VFG-25 = National Eye Institute Visual Functioning Questionnaire 25-Item Version; NIR = near infrared reflectance; OCT-A = optical coherence tomography angiography; PK = pharmacokinetics; SD-OCT = spectral domain optical coherence tomography; SE = study eye; Term = termination; VEGF = vascular endothelial growth factor.

Note: All ocular assessments are to be performed for both eyes unless annotated with 'SE' (study eye) in the above schedule. All assessments should be performed on the same day. All study visits should be scheduled and projected based on the Day 1 visit date with the exception of Visit 2a which should be based on the Day 1 dose date.

A. For subjects that discontinue the study early, the early termination assessments should be performed after a minimum of 30 days have passed from the last dosing visit. If a subject reports for a scheduled visit and decides to terminate early prior to dosing, the visit should be considered the early termination visit and all early termination procedures should be performed At Month 24, all subjects should be offered entry into an open-label study.

APPENDIX C: VISIT SCHEDULE—Every-Other-Month Group—Screening, Day 1 Through Month 12 (Continued)

B. At Day 1 (Visit 2), confirm subject eligibility through reviewing the inclusion/exclusion criteria and receive confirmation of eligibility from the reading center.

C. Significant medical/surgical history from the previous 5 years. Anti-VEGF treatments (fellow eye) and invasive ocular procedures performed within the past 5 years and while on study should also be recorded. Any history of tobacco use should be recorded.
D. Obtain prior to fluorescein angiography and before study drug administration.
E. At screening, serum pregnancy should be performed for women of childbearing potential. If positive, subject is not eligible to continue in the study.
F. Beginning at Day 1, perform the urine pregnancy test for women of childbearing potential at each treatment visit. If positive, perform a serum pregnancy test. If serum test is positive, study drug should not be administered and an early term visit should be completed.
G. Only subjects that sign the separate consent for the clinical repository and genotyping will have these samples collected. A 14-mL whole-blood sample will be collected each of the specified visits.
H. Blood pressure, respiratory rate, heart rate, and temperature. On dosing days, vital signs should be taken predose.
I. Height and weight should be measured at screening.
J. Perform assessments prior to dilating the eyes.
K. In select countries, the MNREAD or Radner Reading Charts should be done during the study visit, prior to dilating the eyes. It should be performed monocularly first, then binocularly.
L. To be administered by the masked site staff prior to any other assessments performed on that day. In-clinic assessments on the digital application should be completed after completion of all functional tests and quality of life measures prior to dilating the eyes.
M. At select sites, and for those subjects who decide to participate, the digital application will be completed on an electronic device and will consist of assessments for visual function and reading speed. Subjects will receive training at Day 1 on the use of the digital applications and the electronic device and will perform the tests using the application in the clinic at Month 1, Month 2, Month 3, Month 6, Month 12, Month 18, and Month 24. Beginning at Day 1, subjects will complete the functional assessments weekly at home. Subjects should be instructed to complete the assessments on the same day each week and at approximately the same time of day each week, if possible.
N. FAF and SD-OCT images, near infrared reflectance, fluorescein angiograms, and fundus photographs will be performed for the study eye only on days where 'SE' is specified and for both eyes at all other visits as specified in the above schedule and will be sent to the reading center for evaluation (consult the reading center manual for specifics on image capture, processing, and transmission). Images should be captured prior to dosing on dosing days. If a subject misses a study visit or images cannot be obtained at a specific visit, study staff should make every effort to obtain images at the next scheduled visit. If new active CNV is suspected, SD-OCT, FFA, and OCT-A (select sites) images should be collected and sent to the reading center for analysis.
O. Postinjection assessments should be performed within 5 minutes after dosing by the unmasked physician or study staff and should include a gross assessment of vision (finger-counting, hand motion, then light perception when applicable). If subject passes gross vision test, the subject may leave the site. If subject fails gross vision test, the tonometry should be performed. IOP should be ≤30 mm Hg in order for the subject to leave the site. If necessary, antiglaucomatous medication can be given to lower the IOP. If IOP is >30 mm Hg, assessments will continue every approximately 30 minutes from the previous measurement until the subject passes gross vision test and IOP is ≤30 mm Hg. Note: if the study eye is treated with a ranibizumab or aflibercept injection during the same visit as the study treatment (pegcetacoplan or sham), the treatment with ranibizumab or aflibercept must be performed first. The pre- and post- anti-VEGF IOP values must be measured and recorded on the eCRF.
P. Starting at Day 1, study subjects will be contacted by study site staff within 4 ± 2 days after each study treatment visit (through Month 4) to collect any information on any safety concerns, decrease in vision, eye pain, unusual ocular events, or any new ocular symptoms in the study eye. If the physician determines that there are any safety concerns, a follow-up visit should be scheduled as soon as possible.
Q. Record concomitant medications (ie, prescription and over-the-counter medications) used by the patient within 30 days of screening and throughout the subject's participation in the study.
R. In addition to the time points indicated on the study schedule, OCT-A should be performed at the time of any suspected new active CNV. If new active CNV is confirmed in the study eye, OCT-A should be repeated every 2 months for the study eye.
S. At select sites only.

APPENDIX C: VISIT SCHEDULE—Every-Other-Month Group—Screening, Day 1 Through Month 12 (Continued)

T. Administration of study treatment (pegcetacoplan or sham) can be done on a separate day from the assessment visit if both days fall within the visit window. If this occurs on the randomization visit, then the administration of pegcetacoplan or sham should be done within 3 days of randomization and after approval from the medical monitor. When study treatment administration is on a day other than a study visit, then the only assessment that must be done on the day of study treatment administration is the preinjection IOP.

APPENDIX D: VISIT SCHEDULE—Every-Other-Month Group—Month 13 to Month 24

	Treatment												Early Term [A]
Visit #		15		16		17		18		19		20	
Day	390	420	450	480	510	540	570	600	630	660	690	720	
Week	52	56	60	64	68	72	76	80	84	88	92	96	
Month	13	14	15	16	17	18	19	20	21	22	23	24	
Window (+ or – days)		16		16		16		16		16		16	
Informed Consent/Assign Screening Number													
Demographic Data													
Inclusion/Exclusion Criteria [B]													
Medical/Surgical/Ocular History [C]													
Blood Draw—Safety Labs [D,E,F]						x						x	x
Urine Sample Collection [D, E,F]						x						x	x
Urine Pregnancy Test [D,E,F]		x		x		x		x		x		x	
Blood Draw—PK and Complement Profile (C3, CH50, AH50) [D,S]												x	x
Blood Draw—Anti-Pegcetacoplan Ab [D]		x				x						x	x
Blood Draw for Clinical Repository (if applicable) [D,G]												x	x
Vital Signs [H]		x		x		x		x		x		x	x
Physical Examination [I]												x	x
BCVA [J]		x		x		x		x		x		x	x
LL-BCVA [J]		x		x		x		x		x		x	x
MNREAD or Radner Reading Charts (select countries) [J,K]						x						x	x
Slitlamp Examination		x		x		x		x		x		x	x
Endothelial Cell Count[S]												x	x
NEI VFQ-25 [L]						x						x	x
FRI [L]						x						x	x
Home-Based Digital Applications (optional, select sites) [L,M,S]						x						x	

APPENDIX D: VISIT SCHEDULE—Every-Other-Month Group—Month 13 to Month 24 (Continued)

	Treatment												Early Term [A]
**Visit #**		**15**		**16**		**17**		**18**		**19**		**20**	
**Day**	**390**	**420**	**450**	**480**	**510**	**540**	**570**	**600**	**630**	**660**	**690**	**720**	
**Week**	**52**	**56**	**60**	**64**	**68**	**72**	**76**	**80**	**84**	**88**	**92**	**96**	
**Month**	**13**	**14**	**15**	**16**	**17**	**18**	**19**	**20**	**21**	**22**	**23**	**24**	
**Window (+ or – days)**		**16**		**16**		**16**		**16**		**16**		**16**	
Dilated Indirect Ophthalmoscopy		x		x		x		x		x		x	x
IOP Measurement		x		x		x		x		x		x	x
SD-OCT [N]		x		x		x		x		x		x	x
FAF [N]		SE		SE		x		SE		SE		x	x
NIR[N]		SE		SE		x		SE		SE		x	x
DCFP [N]												x	x
FFA [N]												x	x
OCT-A[R,S]						x[R]						x[R]	x
Study Eye Determination													
Pegcetacoplan administration or Sham Injection[T]		x		x		x		x		x			
Postinjection Assessment [O]		x		x		x		x		x			
Follow-Up Call [P]													
Concomitant Medication/Concomitant Ocular Procedures [Q]		x		x		x		x		x		x	x
Adverse Events		x		x		x		x		x		x	x

Abbreviations: AH50 = alternative pathway of complement functional test; BCVA = best corrected visual acuity; CH50 = classical pathway of complement functional test; CNV = choroidal neovascularization; DCFP = digital color fundus photography; eCRF = electronic case report form; FAF = fundus autofluorescence; FFA = fundus fluorescein angiography; FRI = Functional Reading Independence Index; IOP = intraocular pressure; LL-BCVA = low luminance best corrected visual acuity; MNREAD = Minnesota Low-Vision Reading Test; NEI VFG-25 = National Eye Institute Visual Functioning Questionnaire 25-Item Version; NIR = near infrared reflectance; OCT-A = optical coherence tomography angiography; PK = pharmacokinetics; SD-OCT = spectral domain optical coherence tomography; SE = study eye; Term = termination; VEGF = vascular endothelial growth factor.

Note: All ocular assessments are to be performed for both eyes unless annotated with 'SE' (study eye) in the above schedule. . All assessments should be performed on the same day. All study visits should be scheduled and projected based on the Day 1 visit date with the exception of Visit 2a which should be based on the Day 1 dose date.

A. For subjects that discontinue the study early, the early termination assessments should be performed after a minimum of 30 days have passed from the last dosing visit. If a subject reports for a scheduled visit and decides to terminate early prior to dosing, the visit should be considered the early termination visit and all early termination procedures should be performed. At Month 24, all subjects should be offered entry into an open-label study.

APPENDIX D: VISIT SCHEDULE—Every-Other-Month Group—Month 13 to Month 24 (Continued)

B. At Day 1 (Visit 2), confirm subject eligibility through reviewing the inclusion/exclusion criteria and receive confirmation of eligibility from the reading center.

C. Significant medical/surgical history from the previous 5 years. Anti-VEGF treatments (fellow eye) and invasive ocular procedures performed within the past 5 years and while on study should also be recorded. Any history of tobacco use should be recorded.

D. Obtain prior to fluorescein angiography and before study drug administration.

E. At screening, serum pregnancy should be performed for women of childbearing potential. If positive, subject is not eligible to continue in the study.

F. Beginning at Day 1, perform the urine pregnancy test for women of childbearing potential at each treatment visit. If positive, perform a serum pregnancy test. If serum test is positive, study drug should not be administered and an early term visit should be completed.

G. Only subjects that sign the separate consent for the clinical repository and genotyping will have these samples collected. A 14-mL whole-blood sample will be collected at each of the specified visits.

H. Blood pressure, respiratory rate, heart rate, and temperature. On dosing days, vital signs should be taken predose.

I. Height and weight should be measured at screening.

J. Perform assessments prior to dilating the eyes.

K. In select countries, the MNREAD or Radner Reading Charts should be done during the study visit, prior to dilating the eyes. It should be performed monocularly first, then binocularly.

L. To be administered by the masked site staff prior to any other assessments performed on that day. In-clinic assessments on the digital application should be completed after completion of all functional tests and quality of life measures prior to dilating the eyes.

M. At select sites, and for those subjects who decide to participate, the digital applications will be completed on an electronic device and will consist of assessments for visual function and reading speed. Subjects will receive training at Day 1 on the use of the digital applications and the electronic device and will perform the tests using the application in the clinic at Month 1, Month 2, Month 3, Month 6, Month 12, Month 18, and Month 24. Beginning at Day 1, subjects will complete the functional assessments weekly at home. Subjects should be instructed to complete the assessments on the same day each week and at approximately the same time of day each week, if possible.

N. FAF and SD-OCT images, near infrared reflectance, fluorescein angiograms, and fundus photographs will be performed for the study eye only on days where 'SE' is specified and for both eyes at all other visits as specified in the above schedule and will be sent to the reading center for evaluation (consult the reading center manual for specifics on image capture, processing, and transmission). Images should be captured prior to dosing on dosing days. If a subject misses a study visit or images cannot be obtained at a specific visit, study staff should make every effort to obtain images at the next scheduled visit. If new active CNV is suspected, SD-OCT, FFA, and OCT-A (select sites) images should be collected and sent to the reading center for analysis.

O. Postinjection assessments should be performed within 5 minutes after dosing by the unmasked physician or study staff and should include a gross assessment of vision (finger-counting, hand motion, then light perception when applicable). If subject passes gross vision test, the subject may leave the site. If subject fails gross vision test, the tonometry should be performed. IOP should be ≤30 mm Hg in order for the subject to leave the site. If necessary, antiglaucomatous medication can be given in order to lower IOP. If IOP is >30 mm Hg, assessments will continue every approximately 30 minutes from the previous measurement until the subject passes the gross vision test and IOP is ≤30 mm Hg. Note: if the study eye is treated with a ranibizumab or aflibercept injection during the same visit as the study treatment (pegcetacoplan or sham), the treatment with ranibizumab or aflibercept must be performed first. The pre- and post- anti-VEGF IOP values must be measured and recorded on the eCRF.

P. Starting at Day 1, study subjects will be contacted by study site staff within 4 ± 2 days after each study treatment visit (through Month 4) to collect any information on any safety concerns, decrease in vision, eye pain, unusual ocular events, or any new ocular symptoms in the study eye. If the investigator determines that there are any safety concerns, a follow-up visit should be scheduled as soon as possible.

Q. Record concomitant medications (ie, prescription and over-the-counter medications) used by the patient within 30 days of screening and throughout the subject's participation in the study.

R. In addition to the time points indicated on the study schedule, OCT-A should be performed at the time of any suspected new active CNV. If new active CNV is confirmed in the study eye, OCT-A should be repeated every 2 months for the study eye.

S. At select sites only.

T. Administration of study treatment (pegcetacoplan or sham) can be done on a separate day from the assessment visit if both days fall within the visit window. If this occurs on the randomization visit, then the administration of pegcetacoplan or sham should be done within 3 days of randomization and after approval from the medical monitor. When study treatment administration is on a day other than a study visit, then the only assessment that must be done on the day of study treatment administration is the preinjection IOP.

APPENDIX E: NATIONAL EYE INSTITUTE VISUAL FUNCTIONING QUESTIONNAIRE 25-ITEM VERSION

PB/IA

National Eye Institute Visual Functioning Questionnaire - 25 (VFQ-25)

version 2000

(INTERVIEWER ADMINISTERED FORMAT)

January 2000

RAND hereby grants permission to use the "National Eye Institute Visual Functioning Questionnaire 25 (VFQ-25) July 1996, in accordance with the following conditions which shall be assumed by all to have been agreed to as a consequence of accepting and using this document:

1. Changes to the NEI VFQ-25 - July 1996 may be made without the written permission of RAND. However, all such changes shall be clearly identified as having been made by the recipient.

2. The user of this NEI VFQ-25 - July 1996 accepts full responsibility, and agrees to hold RAND harmless, for the accuracy of any translations of the NEI VFQ-25 Test Version - July 1996 into another language and for any errors, omissions, misinterpretations, or consequences thereof.

3. The user of this NEI VFQ-25 - July 1996 accepts full responsibility, and agrees to hold RAND harmless, for any consequences resulting from the use of the NEI VFQ-25.

4. The user of the NEI VFQ-25 - July 1996 will provide a credit line when printing and distributing this document or in publications of results or analyses based on this instrument acknowledging that it was developed at RAND under the sponsorship of the National Eye Institute.

5. No further written permission is needed for use of this NEI VFQ-25 - July 1996.

7/29/96

© R 1996

Instructions:

I'm going to read you some statements about problems which involve your vision or feelings that you have about your vision condition. After each question I will read you a list of possible answers. Please choose the response that best describes your situation.

Please answer all the questions as if you were wearing your glasses or contact lenses (if any).

Please take as much time as you need to answer each question. All your answers are confidential. In order for this survey to improve our knowledge about vision problems and how they affect your quality of life, your answers must be as accurate as possible. Remember, if you wear glasses or contact lenses for a particular activity, please answer all of the following questions as though you were wearing them.

© R 1996

Visual Functioning Questionnaire - 25

PART 1 - GENERAL HEALTH AND VISION

1. **<u>In general,</u> would you say your overall <u>health</u> is*:**

(Circle One)

READ CATEGORIES:

Excellent	1
Very Good	2
Good................................	3
Fair..................................	4
Poor	5

2. **At the present time, would you say your eyesight using both eyes (with glasses or contact lenses, if you wear them) is <u>excellent</u>, <u>good</u>, <u>fair</u>, <u>poor</u>, or <u>very</u> <u>poor</u> or are you <u>completely blind</u>?**

(Circle One)

READ CATEGORIES:

Excellent	1
Good................................	2
Fair..................................	3
Poor	4
Very Poor	5
Completely Blind.............	6

*** Skip Question 1 when the VFQ-25 is administered at the same time as the SF-36 or RAND 36-Item Health Survey 1.0**

© R 1996

3. How much of the time do you worry about your eyesight?

(Circle One)

READ CATEGORIES:

None of the time........................	1
A little of the time.......................	2
Some of the time.........................	3
Most of the time	4
All of the time?...........................	5

4. How much pain or discomfort have you had in and around your eyes (for example, burning, itching, or aching)? Would you say it is:

(Circle One)

READ CATEGORIES:

None	1
Mild..................................	2
Moderate	3
Severe, or.........................	4
Very severe?.....................	5

PART 2 - DIFFICULTY WITH ACTIVITIES

The next questions are about how much difficulty, if any, you have doing certain activities wearing your glasses or contact lenses if you use them for that activity.

5. How much difficulty do you have reading ordinary print in newspapers? Would you say you have:
(READ CATEGORIES AS NEEDED)

(Circle One)

No difficulty at all..	1
A little difficulty..	2
Moderate difficulty...	3
Extreme difficulty..	4
Stopped doing this because of your eyesight	5
Stopped doing this for other reasons or not interested in doing this	6

© R 1996

6. How much difficulty do you have doing work or hobbies that require you to <u>see well up close</u>, such as cooking, sewing, fixing things around the house, or using hand tools? Would you say:
(READ CATEGORIES AS NEEDED)

(Circle One)

No difficulty at all.. 1
A little difficulty.. 2
Moderate difficulty.. 3
Extreme difficulty.. 4
Stopped doing this because of your eyesight.... 5
Stopped doing this for other reasons or not interested in doing this 6

7. Because of your eyesight, how much difficulty do you have <u>finding something on a crowded shelf</u>?
(READ CATEGORIES AS NEEDED)

(Circle One)

No difficulty at all.. 1
A little difficulty.. 2
Moderate difficulty.. 3
Extreme difficulty.. 4
Stopped doing this because of your eyesight.... 5
Stopped doing this for other reasons or not interested in doing this 6

8. How much difficulty do you have <u>reading street signs or the names of stores</u>?
(READ CATEGORIES AS NEEDED)

(Circle One)

No difficulty at all.. 1
A little difficulty.. 2
Moderate difficulty.. 3
Extreme difficulty.. 4
Stopped doing this because of your eyesight.... 5
Stopped doing this for other reasons or not interested in doing this 6

© R 1996

9. **Because of your eyesight, how much difficulty do you have going down steps, stairs, or curbs in dim light or at night?**
(READ CATEGORIES AS NEEDED)

(Circle One)

No difficulty at all.. 1
A little difficulty.. 2
Moderate difficulty.. 3
Extreme difficulty.. 4
Stopped doing this because of your eyesight.... 5
Stopped doing this for other reasons or not interested in doing this 6

10. **Because of your eyesight, how much difficulty do you have noticing objects off to the side while you are walking along?**
(READ CATEGORIES AS NEEDED)

(Circle One)

No difficulty at all.. 1
A little difficulty.. 2
Moderate difficulty.. 3
Extreme difficulty.. 4
Stopped doing this because of your eyesight.... 5
Stopped doing this for other reasons or not interested in doing this 6

11. **Because of your eyesight, how much difficulty do you have seeing how people react to things you say?**
(READ CATEGORIES AS NEEDED)

(Circle One)

No difficulty at all.. 1
A little difficulty.. 2
Moderate difficulty.. 3
Extreme difficulty.. 4
Stopped doing this because of your eyesight.... 5
Stopped doing this for other reasons or not interested in doing this 6

© R 1996

12. Because of your eyesight, how much difficulty do you have <u>picking out and matching your own clothes</u>?
(READ CATEGORIES AS NEEDED)

(Circle One)

No difficulty at all	1
A little difficulty	2
Moderate difficulty	3
Extreme difficulty	4
Stopped doing this because of your eyesight	5
Stopped doing this for other reasons or not interested in doing this	6

13. Because of your eyesight, how much difficulty do you have <u>visiting with people in their homes, at parties, or in restaurants</u> ?
(READ CATEGORIES AS NEEDED)

(Circle One)

No difficulty at all	1
A little difficulty	2
Moderate difficulty	3
Extreme difficulty	4
Stopped doing this because of your eyesight	5
Stopped doing this for other reasons or not interested in doing this	6

14. Because of your eyesight, how much difficulty do you have <u>going out to see movies, plays, or sports events</u>?
(READ CATEGORIES AS NEEDED)

(Circle One)

No difficulty at all	1
A little difficulty	2
Moderate difficulty	3
Extreme difficulty	4
Stopped doing this because of your eyesight	5
Stopped doing this for other reasons or not interested in doing this	6

© R 1996

15. Now, I'd like to ask about driving a car. Are you currently driving, at least once in a while?

(Circle One)

Yes .... 1 *Skip To Q 15c*

No 2

15a. IF NO, ASK: Have you never driven a car or have you given up driving?

(Circle One)

Never drove ...... 1 *Skip To Part 3, Q 17*

Gave up............ 2

15b. IF GAVE UP DRIVING: Was that mainly because of your eyesight, mainly for some other reason, or because of both your eyesight and other reasons?

(Circle One)

Mainly eyesight .... 1 *Skip To Part 3, Q 17*

Mainly other reasons .... 2 *Skip To Part 3, Q 17*

Both eyesight and other reasons ... 3 *Skip To Part 3, Q 17*

15c. IF CURRENTLY DRIVING: How much difficulty do you have driving during the daytime in familiar places? Would you say you have:

(Circle One)

No difficulty at all 1
A little difficulty 2
Moderate difficulty 3
Extreme difficulty 4

© R 1996

16. How much difficulty do you have driving at night? Would you say you have: (READ CATEGORIES AS NEEDED)

(Circle One)

No difficulty at all..................................	1
A little difficulty......................................	2
Moderate difficulty................................	3
Extreme difficulty..................................	4
Have you stopped doing this because of your eyesight................................	5
Have you stopped doing this for other reasons or are you not interested in doing this	6

16a. How much difficulty do you have driving in difficult conditions, such as in bad weather, during rush hour, on the freeway, or in city traffic? Would you say you have:
(READ CATEGORIES AS NEEDED)

(Circle One)

No difficulty at all..................................	1
A little difficulty......................................	2
Moderate difficulty................................	3
Extreme difficulty..................................	4
Have you stopped doing this because of your eyesight................................	5
Have you stopped doing this for other reasons or are you not interested in doing this	6

© R 1996

PART 3: RESPONSES TO VISION PROBLEMS

The next questions are about how things you do may be affected by your vision. For each one, I'd like you to tell me if this is true for you <u>all</u>, <u>most</u>, <u>some</u>, <u>a little</u>, or <u>none</u> of the time.

(Circle One On Each Line)

READ CATEGORIES:	All of the time	Most of the time	Some of the time	A little of the time	None of the time
17. <u>Do you accomplish less</u> than you would like because of your vision?	1	2	3	4	5
18. <u>Are you limited</u> in how long you can work or do other activities because of your vision?	1	2	3	4	5
19. How much does pain or discomfort <u>in or around your eyes</u>, for example, burning, itching, or aching, keep you from doing what you'd like to be doing? Would you say:	1	2	3	4	5

© R 1996

For each of the following statements, please tell me if it is definitely true, mostly true, mostly false, or definitely false for you or you are not sure.

(Circle One On Each Line)

	Definitely True	Mostly True	Not Sure	Mostly False	Definitely False
20. I stay home most of the time because of my eyesight.....	1	2	3	4	5
21. I feel frustrated a lot of the time because of my eyesight..............................	1	2	3	4	5
22. I have much less control over what I do, because of my eyesight.	1	2	3	4	5
23. Because of my eyesight, I have to rely too much on what other people tell me..	1	2	3	4	5
24. I need a lot of help from others because of my eyesight..............................	1	2	3	4	5
25. I worry about doing things that will embarrass myself or others, because of my eyesight..............................	1	2	3	4	5

That's the end of the interview. Thank you very much for your time and your help.

© R 1996

Appendix of Optional Additional Questions

SUBSCALE: GENERAL HEALTH

A1. How would you rate your overall health, on a scale where zero is as bad as death and 10 is best possible health?

(Circle One)

0	1	2	3	4	5	6	7	8	9	10
**Worst**										**Best**

SUBSCALE: GENERAL VISION

A2. How would you rate your eyesight now (with glasses or contact lens on, if you wear them), on a scale of from 0 to 10, where zero means the worst possible eyesight, as bad or worse than being blind, and 10 means the best possible eyesight?

(Circle One)

0	1	2	3	4	5	6	7	8	9	10
**Worst**										**Best**

SUBSCALE: NEAR VISION

A3. Wearing glasses, how much difficulty do you have reading the small print in a telephone book, on a medicine bottle, or on legal forms? Would you say:
(READ CATEGORIES AS NEEDED)

(Circle One)

No difficulty at all **1**
A little difficulty **2**
Moderate difficulty **3**
Extreme difficulty **4**
Stopped doing this because of your eyesight **5**
Stopped doing this for other reasons or not interested in doing this **6**

© R 1996

A4. Because of your eyesight, how much difficulty do you have figuring out whether bills you receive are accurate?
(READ CATEGORIES AS NEEDED)

(Circle One)

**No difficulty at all**	**1**
**A little difficulty**	**2**
**Moderate difficulty**	**3**
**Extreme difficulty**	**4**
**Stopped doing this because of your eyesight**	**5**
**Stopped doing this for other reasons or not interested in doing this**	**6**

A5. Because of your eyesight, how much difficulty do you have doing things like shaving, styling your hair, or putting on makeup?
(READ CATEGORIES AS NEEDED)

(Circle One)

**No difficulty at all**	**1**
**A little difficulty**	**2**
**Moderate difficulty**	**3**
**Extreme difficulty**	**4**
**Stopped doing this because of your eyesight**	**5**
**Stopped doing this for other reasons or not interested in doing this**	**6**

SUBSCALE: DISTANCE VISION

A6. Because of your eyesight, how much difficulty do you have recognizing people you know from across a room?
(READ CATEGORIES AS NEEDED)

(Circle One)

**No difficulty at all**	**1**
**A little difficulty**	**2**
**Moderate difficulty**	**3**
**Extreme difficulty**	**4**
**Stopped doing this because of your eyesight**	**5**
**Stopped doing this for other reasons or not interested in doing this**	**6**

© R 1996

A7. Because of your eyesight, how much difficulty do you have taking part in active sports or other outdoor activities that you enjoy (like golf, bowling, jogging, or walking)?
(READ CATEGORIES AS NEEDED)

(Circle One)

No difficulty at all.. 1
A little difficulty.. 2
Moderate difficulty.. 3
Extreme difficulty.. 4
Stopped doing this because of your eyesight.... 5
Stopped doing this for other reasons or not interested in doing this 6

A8. Because of your eyesight, how much difficulty do you have seeing and enjoying programs on TV?
(READ CATEGORIES AS NEEDED)

(Circle One)

No difficulty at all.. 1
A little difficulty.. 2
Moderate difficulty.. 3
Extreme difficulty.. 4
Stopped doing this because of your eyesight.... 5
Stopped doing this for other reasons or not interested in doing this 6

SUBSCALE: SOCIAL FUNCTION

A9. Because of your eyesight, how much difficulty do you have entertaining friends and family in your home?
(READ CATEGORIES AS NEEDED)

(Circle One)

No difficulty at all.. 1
A little difficulty.. 2
Moderate difficulty.. 3
Extreme difficulty.. 4
Stopped doing this because of your eyesight.... 5
Stopped doing this for other reasons or not interested in doing this 6

© R 1996

SUBSCALE: DRIVING

A10. [This items, "driving in difficult conditions", has been included as item 16a as part of the base set of 25 vision-targeted items.]

SUBSCALE: ROLE LIMITATIONS

A11. The next questions are about things you may do because of your vision. For each item, I'd like you to tell me if this is true for you all, most, some, a little, or none of the time.
(READ CATEGORIES AS NEEDED)

(Circle One On Each Line)

	All of the time	Most of the time	Some of the time	A little of the time	None of the time
a. Do you have more help from others because of your vision?	1	2	3	4	5
b. Are you limited in the kinds of things you can do because of your vision?	1	2	3	4	5

© R 1996

SUBSCALES: WELL-BEING/DISTRESS (#A12) and DEPENDENCY (#A13)

The next questions are about how you deal with your vision. For each statement, please tell me if it is definitely true, mostly true, mostly false, or definitely false for you or you don't know.

(Circle One On Each Line)

	Definitely True	Mostly True	Not Sure	Mostly False	Definitely False
**A12.** I am often irritable because of my eyesight.	1	2	3	4	5
**A13.** I don't go out of my home alone, because of my eyesight..............................	1	2	3	4	5

© R 1996

APPENDIX F: FUNCTIONAL READING INDEPENDENCE INDEX

Kimel M, Yu R, Leidy N. The Functional Reading Independence Index (FRI Index) - User Manual (Version 1.2). Evidera. 2015

CCI

[redacted]

CCI

[redacted]

CCI

[redacted]

CCI

[redacted]

CCI

[redacted]

CCI

[redacted]

CCI

[redacted]

CCI

[redacted]

CCI

[redacted]

CCI

[redacted]

CCI [redacted]

CCI

[redacted]

CCI

[redacted]

CCI

[redacted]

CCI

[redacted]

CCI

[redacted]

CCI

[redacted]

CCI

[redacted]

CCI

[redacted]

CCI

[redacted]

CCI

[redacted]

CCI

[redacted]

CCI

[redacted]

CCI

[redacted]

CCI

[redacted]

CCI

[redacted]

CCI

[redacted]

CCI

[redacted]

CCI

[redacted]

CCI

[redacted]

CCI

[redacted]

CCI

[redacted]

APPENDIX G: MINNESOTA LOW-VISION READING TEST AND RADNER READING CARDS

The manual of procedures should be referenced for a detailed table of countries and languages to determine which test will be administered. Equipment and testing procedures will also be described in detail in the manual of procedures for both tests.

MINNESOTA LOW-VISION READING TEST:

The Minnesota Low-Vision Reading Test (MNREAD) acuity cards are continuous-text reading-acuity cards used for measuring the reading acuity and speed of normal and low-vision patients. These charts were developed at the Minnesota Laboratory for Low-Vision Research, University of Minnesota, Minneapolis, Minnesota, USA.

The MNREAD Acuity Charts will be used to measure:

- Critical print size: the smallest print that the patient can read with maximum speed
- Maximum reading speed: the patient's reading speed when reading is not limited by print size

RADNER READING CARDS:

The Radner Reading Cards consist of sentence optotypes, which are optimized reading test items, standardized by construction and statistical selection. These cards allow for accurate and comparable measurements of reading acuity and reading speed and measurement of critical print size.

APPENDIX H: GRADING SCALE FOR ASSESSMENT OF ANTERIOR CHAMBER FLARE OR CELLS

AQUEOUS REACTION- FLARE

Grade	Description
0	No protein is visible in the anterior chamber when viewed by an experienced observer using slitlamp biomicroscopy; a small, bright, focal slit-beam of white light; and high magnification.
Trace	Trace amount of protein detectable in the anterior chamber. This protein is visible only with careful scrutiny by an experienced observer using slitlamp biomicroscopy; a small, bright, focal slit-beam of white light; and high magnification.
1+	Mild amount of protein detectable in the anterior chamber. This protein is immediately apparent to an experienced observer using slitlamp biomicroscopy and high magnification, but such protein is detected only with careful observation with the naked eye and a small, bright, focal slit-beam of white light. Note presence of any fibrin.
2-3+	Moderate amount of protein detectable in the anterior chamber. These grades are similar to 1+ but the opacity would be readily visible to the naked eye of an observer using any source of a focused beam of white light. This is a continuum of moderate opacification, with 2+ being less apparent than 3+. Note presence of any fibrin.
4+	A large (severe) amount of protein is detectable in the anterior chamber. Similar to 3+, but the density of the protein approaches that of the lens. Additionally, frank fibrin deposition is frequently seen in acute circumstances. It needs to be noted that because fibrin may persist for a period of time after partial or complete restoration of the blood-aqueous barrier, it is possible to have resorbing fibrin present with lower numeric assignations for flare (eg, 1+ flare with fibrin).

AQUEOUS REACTION- CELLS

Grade	Description
0	No cells are seen in any optical section when a large slitlamp beam is swept across the anterior chamber.
Trace	Rare (1-3) cells are observed when the slitlamp beam is swept across the anterior chamber. When the instrument is held stationary, not every optical section contains circulating cells.
1+	3-10 cells/optical section are seen when the slitlamp beam is swept across the anterior chamber. When the instrument is held stationary, every optical section contains circulating cells. Note stage of hypopyon, if applicable.
2+	10-25 cells are seen when the slitlamp beam is swept across the anterior chamber. When the instrument is held stationary, every optical section contains circulating cells. Note stage of hypopyon, if applicable.
3+	25-50 cells are seen when the slitlamp beam is swept across the anterior chamber. When the instrument is held stationary, every optical section contains circulating cells. Keratic precipitates or cellular deposits on the anterior lens capsule may be present. Note stage of hypopyon, if applicable.
4+	More than 50 cells are seen when the slitlamp beam is swept across the anterior chamber. When the instrument is held stationary, every optical section contains circulating cells or hypopyon is noted. As for the fibrin deposition, hypopyon may persist for a period of time after exudation of cells into the anterior chamber has diminished or ceased entirely, making it possible for have 1+ circulating cells in the anterior chamber with a resolving hypopyon.

APPENDIX I: GRADING SCALE FOR ASSESSMENT OF VITREOUS CELLS

Grade	Description	Cells in Retro-Illuminated Field
0	Clear	0
Trace	Few opacities	1-20
1	Scattered opacities	21-50
2	Moderate opacities	51-100
3	Many opacities	101-250
4	Dense opacities	≥251

APPENDIX J: AMENDMENT HISTORY

A summary of changes from previous amendments is provided below.

Protocol Versions		
**Summary of Change(s) Since Last Version of Approved Protocol**		
**Amendment 4**	**Amendment Date<br>27 April 2020**	**Global<br><br>Section(s) Affected by Change**
**Description of Change**		
Non-substantial changes that did not impact content of the document have been made for clarity.		Entire document
Information related to COVID-19-related changes added		Appendix K

Protocol Versions		
**Summary of Change(s) Since Last Version of Approved Protocol**		
**Amendment 3<br>Version 1.0**	**Amendment Date<br>14 February 2019**	**Global<br><br>Section(s) Affected by Change**
**Description of Change**		
Non-substantial changes that did not impact content of the document have been made for clarity.		Entire document
The study has been updated to note that complement profiling will be conducted in select sites only.		Entire document
It was noted that:<br>In addition, two GLP-compliant 2-month ocular bridging studies (the first comparing drug substance from two different CMOs, the second assessing the safety and tolerability profiles of three different formulations to support the Phase 3 clinical study) have been conducted, in cynomolgus monkeys as well.<br>The previous version of the protocol noted that 1 2-month ocular bridging study was completed and that data from a second study would be performed to assess the safety and tolerability profiles of three different formulations in order to support the Phase 3 formulation.		Section 4.1.3 Nonclinical Data
Updated to include Clinical Study APL2-103.		Section 4.1.4 Clinical Data
Updated to include the following statement:<br><br>In recent studies conducted with APL 2 IVT from a single manufacturer, events of transient moderate and severe intraocular inflammation have been observed.		Section 4.2 Risk/Benefit

Section revised to further define approved methods of contraception include:<br>1. Combined (estrogen-and progestogen-containing) hormonal contraception associated with inhibition of ovulation:<br>1. Oral<br>2. Intravaginal<br>3. Transdermal<br>2. Progestogen-only hormonal contraception associated with inhibition of ovulation:<br>1. Oral<br>2. Injectable<br>3. Implantable<br>3. Intrauterine device (IUD)<br>4. Intrauterine hormone-releasing system (IUS)<br>5. Bilateral tubal occlusion<br>6. Vasectomized partner (provided that partner is the sole sexual partner of the WOCBP trial participant and that the vasectomized partner has received medical assessment of the surgical success)<br>7. Sexual abstinence (defined as refraining from heterosexual intercourse during the entire period of risk associated with the study treatments). Sexual abstinence is only accepted when it is the preferred and usual lifestyle of the subject.<br><br>~~Approved methods of contraception include: oral contraceptives, intrauterine device, medically acceptable barrier methods (diaphragm or condom), implantable or injectable contraceptives (like DepoProvera) or removable birth control device (like NuvaRing or Ortho Evra patches); and/or surgical sterilization (at least 6 months before dosing). Subjects practicing abstinence and coitus interruptus (pull out method) must agree to use an approved method of contraception during the study and 90 days after their last dose of study drug.~~	Section 6.4 Approved Methods of Contraception
The following section was added:<br><br>A subject may withdraw from the study at any time for any reason without prejudice to his/her future medical care by the physician or at the institution. The investigator or sponsor may withdraw the subject at any time (n, in the interest of subject safety). The investigator is encouraged to discuss withdrawal of a subject from treatment with the investigational product with the medical monitor when possible. Subjects who discontinue treatment with the investigational product can continue participation in the study and should be encouraged to return to the clinical site for as many follow-up visits as they can. In the event that a subject terminates early from the study, all early termination procedures should be performed even if they are outside the allowed study window.<br>The reason for termination, date of stopping treatment with	Section 6.5 Discontinuation of Subjects

investigational product, all follow-up information and the total amount of investigational product administered must be recorded in the case report form (CRF) and source documents.	
The requirement to notify the sponsor prior to unmasking was removed:<br><br>In the event of a medical emergency where the knowledge of subject treatment by masked individuals (eg, the subject or his/her physician) is required, an individual Investigator (or designee) will have the ability to unmask the treatment assignment for a specific subject and share that information with the appropriate parties. ~~The Investigator (or designee) must endeavor to notify the Sponsor prior to unmasking a subject.~~	Section 7.2.1 Unmasking
Section was revised to allow study treatment administration to occur on a day separate from the assessment visit:<br><br>Only qualified study staff and those delegated the responsibility of study drug administration on the Delegation of Authority log should perform this procedure. All staff should be appropriately trained on all procedures prior to performing the procedures. Sites should follow the Visit Schedule for order of procedures and assessments.<br><br>Administration of study treatment (APL-2 or Sham) can be done on a separate day from the assessment visit if both days fall within the visit window. If this occurs on the randomization visit, then the administration of APL-2 or Sham should be done within 3 days of randomization and after approval from the medical monitor. When study treatment administration is on a day other than a study visit, then the only assessment that must be done on the day of study treatment administration is the pre-injection IOP.<br><br>If a subject falls outside the visit window for a dosing visit, the dose should be skipped and the subject should be scheduled on time for the next dosing visit.	Section 7.3.2 Treatment Administration
The description of sham injection was modified as there is no requirement for sham to be sterile:<br><br>Sham will be provided as ~~sterile~~ empty stoppered glass vials and should be stored according to the label.	Section 7.3.3.1 Identity of the Investigational Product
The procedure for sham injection was further described:<br><br>The procedure for sham injection will be the same as that used for IVT injection until the actual injection but no actual injection will occur. The injecting physician will only touch the study eye with the blunt end of the syringe. No needle or medication will be injected inside the eye.	Section 7.4 Sham Injection Administration

Detailed instructions on sham injection procedures and post-injection procedures will be provided in the manual of procedures.	
The end of the trial for each subject was defined:<br><br>The end of the trial for each subject is defined as when the subject either completes their Month 24 Visit and enrolls in the open-label extension study or, should a subject elect not to enter the open label extension study, when the subject completes their exit visit at Month 30.<br><br>Through the section it has also been noted that endothelial cell count assessment is for "select sites only". This was previously noted in the schedule of events but not consistently in the body of the protocol. In addition FAF and NAR are no longer "study eye only".	Section 8. Study Procedures
The following assessments were removed as they will no longer be conducted at the screening Visit 1:<br>~~1. Low luminance BCVA~~<br>~~2. Endothelial cell count~~<br>~~3. OCT-A~~<br>The following text was moved to Section 8.1.2 as training for home-based digital applications will now be conducted at Visit 2:<br>~~Prior to dilating the eyes, subjects at select sites will be trained on how to use the home-based digital applications for visual function and reading speed if the subject decides to participate in this portion. This training must occur after completion of all functional tests (NL-BCVA, LL-BCVA).~~	Section 8.1.1 Visit 1 All Subjects
It was clarified that:<br>All assessments should be performed on the same day. All study visits should be scheduled and projected based on the Day 1 visit date with the exception of Visit 2a which should be based on the Day 1 dose date.<br><br>The following text was added as home-based digital application training will now occur at Visit 2:<br>Subjects at select sites will be trained on how to use the home-based digital applications for visual function and reading speed if the subject decides to participate in this portion. This training must occur after completion of all functional tests (NL-BCVA, LL-BCVA).<br><br>The following assessments were added:<br>4. Endothelial cell count<br>5. OCT-A (select sites)<br><br>In addition FAF and NAR are no longer "study eye only".<br><br>The following text was removed as subject discontinuation is now covered in Section 6.5.<br>~~In the event that a subject is early terminated from the study, all early termination procedures should be performed even if they are outside the allowed study window.~~	Section 8.1.2 Randomization/Initial Treatment – Day – Within 28 Days of Screening

~~If the subject would like to discontinue dosing but is amenable to continuing in the study, the site should make every effort to have the subject complete as many follow-up visits as possible.~~	
Visit 2A for PK, complement profile (select sites) and Anti-APL-2 antibody sample collection was added.<br><br>Visit 2A- All Groups (select sites)<br>Blood should be drawn for PK and Complement profiling (select sites).	Section 8.1.3 PK and Complement Sample Day 7 (select sites)
It has been noted in the text which assessments will be conducted at Month 27 and Month 30.	Section 8.1.5 Follow-up Phase
Blood draw was modified to reflect revised PK sampling schedule.	Table 3
Instructions were revised to include the following:<br><br>All AEs encountered during the study will be monitored and reported in detail in the source documents and documented on the eCRF, from signing of the ICF until the Exit Visit. AEs should be recorded by maximum severity. AEs, especially those for which the relationship to ~~test drug~~ study treatment are considered by the Investigator to be possibly or definitely related, should be followed up until they have returned to the baseline status or stabilized. If a clear explanation is established, it should be recorded on the eCRF.	Section 11.2 Recording Adverse Events
Instructions modified as follows:<br>~~If any AEs are serious, special procedures will be followed. All SAEs will be reported to the Safety Monitor by the Investigator via fax or email within one calendar day of becoming aware of the event, whether or not the serious events are deemed drug-related. SAE reporting contact information will be provided separately and as included in the Safety Monitoring Plan. All SAEs must be reported to the applicable ethics committee by the Investigator in accordance with their regulations.~~<br><br>**Special procedures will be followed for reporting SAEs.** All SAEs will be reported to the Safety Monitor by the Investigator via eCRF or fax/email (if eCRF is not available) within **24 hours** of becoming aware of the event, whether or not the event is deemed treatment-related. If the EDC system is not operational, the site must complete the appropriate paper SAE form and fax/email to the number listed on the SAE form, also within 24 hours of becoming aware of the event. The reported information submitted as a paper SAE form must be entered into the EDC system once it becomes operational.<br>SAE reporting contact information will be provided separately and included in the Safety Monitoring Plan. All SAEs must be reported to the applicable ethics committee by the Investigator in accordance with their regulations.	Section 11.5 Serious Adverse Events
The following text was added:<br><br>A suspected adverse reaction means any AE for which there is a reasonable possibility that the drug caused the AE. Reasonable	Section 11.6 Unexpected Adverse Events or Unexpected Suspected Adverse Reactions

possibility means there is evidence (such as a temporal relationship) to suggest a causal relationship between the drug and the AE.	
The analysis description was modified as follows as this will be conducted at all sites. This is an error correction.<br>• Change from baseline at each planned assessment in the total area of GA lesion(s) in the study eye (in mm^2) as assessed by FAF ~~(in select sites)~~	Section 12.7.2 Secondary Efficacy Analysis
Section 12.11 was added to describe how visit windows will be used in the assessment of the primary endpoint:<br>Analysis visits will be derived with windows for the monthly visits to assess the primary endpoint. Baseline is defined as the date of randomization. If 2 or more treatment visits occur within a window, the closest visit to the target day will be used as that analysis visit; if 2 visits are equidistant from the scheduled analysis visit day, the later analysis visit will be used.	Section 12.11 Visit Windows
All study schedules were modified to align with the changes noted above, including the updated PK and complement profile (in select sites) sampling schedule, as well as the addition of Visit 2A.	Appendices A, B,C, D: Visit Schedules

**Protocol Versions**		
**Summary of Change(s) Since Last Version of Approved Protocol**		
**Amendment 2**<br>**Version 1.0**	**Amendment Date**<br>**08 August 2018**	**Global**
**Description of Change**		**Section(s) Affected by Change**
Exclusion criteria #4 and #10 were modified as follows for clarity and to exclude subjects with history of IVT injection in either eye:<br>4. Presence ~~in either eye~~ of an active ocular disease that in the opinion of the Investigator compromises or confounds visual function, including but not limited to, uveitis, other macular diseases (e.g. clinically significant epiretinal membrane [ERM], full thickness macular hole or uncontrolled glaucoma/ocular hypertension). Benign conditions in the opinion of the investigator such as peripheral retina dystrophy are not exclusionary.<br>10. History of prior intravitreal injection ~~in the study eye~~.<br>Exclusion criteria #13 was modified as follows for clarity:<br>13. Participation in any systemic experimental treatment or any other systemic investigational new drug including within 6 weeks or 5		Synopsis<br>Section 6.1.2 Exclusion Criteria

half-lives of the active ingredient (whichever is longer) prior to the start of study treatment. Note: clinical trials solely involving observation, over-the-counter vitamins, supplements, or diets are not exclusionary.	
The following exclusion criteria was implemented as Exclusion Criteria #12 (EC criteria following this were renumbered):<br>**12.** Prior participation in another interventional clinical study for geographic atrophy in either eye including investigational oral medication and placebo.	Synopsis<br>Section 6.1.2 Exclusion Criteria
Clarified that the following endpoint will not be limited to select sites only:<br>Change from baseline at each planned assessment in the total area of GA lesion(s) in the study eye (in mm^2) as assessed by FAF.	Synopsis<br>Section 12.7.2 Secondary Efficacy Endpoints
Noted that the NEI VFQ-25 distance activity and near activity subscale score endpoints will be conducted in select countries as these subscales are not available in all languages.	Synopsis<br>Section 5.1.3 Secondary Objectives<br>Section 5.1.5 Exploratory Objectives<br>Section 12.7.2 Secondary Efficacy Analysis<br>Section 12.7.3 Exploratory Efficacy Analysis<br>Section 9.9.1 The National Eye Institute Visual Functioning Questionnaire 25-Item Version (NEI VFQ)<br>Section 12.7.3 Exploratory Efficacy Analysis<br>Section 9.9.1 The National Eye Institute Visual Functioning Questionnaire 25-Item Version (NEI VFQ)
Contraception text was updated to align with the Investigator's Brochure and in studies with APL-2 in other indications:<br>Approved methods of contraception include: ~~hormonal contraceptives associated with inhibition of ovulation,~~ oral contraceptives, intrauterine device, ~~intrauterine hormone-releasing system; and/or bilateral tubal occlusion (at least 6 months before dosing)~~ medically acceptable barrier methods (diaphragm or condom), implantable or injectable contraceptives (like DepoProvera) or removable birth control device (like NuvaRing or Ortho Evra patches); and/or surgical sterilization (at least 6 months before dosing). Subjects practicing abstinence and coitus interruptus (pull out method) must agree to use an approved method of contraception during the study and ~~60~~ 90 days after their last dose of study drug.	Section 6.4 Approved Methods of Contraception
Masking language was modified to indicate that the PI is required to remain masked to subjects' treatment assignments, while the treating physician and any associated support staff involved in IP administration	Section 7.2 Masking and Minimization of Bias

will be unmasked.	
Guidance on the treatment of new exudation related to active choroidal neovascularization in the study eye and/or fellow eye was modified to clarify that the Reading Center will provide a report to indicate whether or not there is evidence of active, exudative AMD, and the Investigator will then determine if anti-VEGF treatment should be initiated. The Investigator should wait for the Reading Center report before making a decision regarding treatment, except in cases where there is clear evidence of disease activity that may have a detrimental visual impact if not treated immediately.<br><br>It was also clarified that either ranibizumab or aflibercept should be selected as the anti-VEGF therapy, and that the unmasked physician should administer anti-VEGF treatment if the treatment is administered on the same day as APL-2.<br><br>It was also noted that any treatments of therapies administered to the fellow eye within 5 years of screening should be recorded as a concomitant medication	Section 7.5.1 Treatment of New Exudation Related to Active Choroidal Neovascularization in the Study Eye and/or Fellow Eye<br>Visit Schedules A-D
The following instruction regarding electronic devices was deleted:<br>~~The subject should be instructed to take the electronic device home and to complete the digital application weekly on the same day each week, if possible.~~	Section 8.1.1 Screening Period
The following instruction regarding electronic devices was added:<br>The subject will be instructed to bring back the electronic device for the visits specified in the schedule of events.	Section 8.1.3.1 Months 1-12
Month 6 and Month 12 were added as times of in-clinic assessment.<br>It was noted that subjects will take electronic devices home at Day 1 instead of at Screening.	Section 9.12 Home-Based Functional Digital Applications (in select sites/countries)<br>Visit Schedules A-D.
Clarified that:<br>In the event that a subject is suspected to have new active CNV in the study eye and/or the fellow eye, an SD-OCT and FA using the protocol specified procedures should be performed and sent to the Reading Center to confirm the diagnosis. In addition, in select sites, OCT-A should also be captured according to the study imaging protocol and sent to the Reading Center.	Section 9.14 Ocular Imaging
It was clarified that if the subject does not pass the gross vision test, IOP must be measured at that time. Additional IOP measurement must be	Section 9.15 Post-Injection Assessment; Visit Schedule

taken approximately every 30 minutes thereafter until IOP ≤ 30 mmHg and the subject is able to be released from the clinic.<br><br>It was clarified that anti-VEGF treatment will be either ranibizumab or aflibercept, and that IOP should be measured before and after the anti-VEGF injection, but prior to administration of APL-2.	Footnote "O" (all Visit Schedules)
Blood volume for study assessments was updated based on the lab manual.	Section 9.16 Blood Volume for Study Assessments
It was clarified that ~~15~~14 mL whole blood sample will be collected at the specified timepoints for research samples.	Section 9.17.2 Sample Collection<br>Visit Schedules A-D
The screening urine pregnancy test was removed as a serum pregnancy test will be performed for screening.<br><br>Footnotes "M" (All Visit Schedules) was modified to indicate that "Beginning at Day 1 Screening, subjects will complete the functional assessments weekly at home."	Visit Schedules A and C
Week 24 blood draw for clinical repository was added (error correction).	Visit Schedule C
The full version of the NEI VFQ-25 has been included in Appendix E.	Appendix E.
An amendment history appendix was added to show changes enacted by previous amendments.	Appendix J
**Amendment 1 Amendment Date**<br>**Version 1.0 31 May 2018**	**Section(s) Affected by Change**
Non-substantial changes that did not impact content of the document have been made for clarity.	Entire document
The changes noted below were incorporated into the synopsis where applicable.	Synopsis
The following text was removed as the data is related to neovascular AMD and was determined not to be critical information for rational as APL-2 as a potential therapy for GA:<br><br>While there is not yet an anti-complement therapy approved for the treatment of neovascular AMD or GA, data from recent clinical studies support the hypothesis that complement inhibition might have an effect on the etiology of age-related macular degeneration. In the Phase I clinical study, ASAP (Potentia Pharmaceuticals, Inc), thinning of the retina occurred in two high dose patients after treatment with the C3 complement inhibitor APL-1 (a.k.a. POT-4 /AL-78898A) despite extensive pre-existing retinal damage and resistance to anti-VEGF	Section 4.1.5.1 Rationale for APL-2 for Treatment of Geographic Atrophy

therapy.	
Study objectives were expanded to describe the endpoints that will be utilized to meet them. Endpoint descriptions were moved to Section 12.	Section 5. STUDY OBJECTIVES AND ENDPOINTS
It was noted that exploratory objectives utilizing digital applications will be conducted in select sites/countries only as these applications will be utilized according to local regulation.	Section 5.1.3 Exploratory Objectives<br>Section 8.1 Study Visit Schedule<br>Section 9.13 Home-Based Digital Applications (in select sites/countries)
It was clarified that to participate in the study, subjects must be diagnosed with GA of the macula secondary to AMD in one or both eyes the study eye.	Section 6. Patient Population
It was noted that if both eyes have the same visual acuity score, the right eye will be selected as the study eye.	Section 6.1.1 Inclusion Criteria
Exclusion criteria 11 was updated as follows:<br>11. Prior participation in another interventional clinical study for intravitreal therapies in either eye (including subjects receiving sham).	Section 6.1.2 Exclusion Criteria
Contraception requirements were updated as follows based on requirements from ethics committees:<br>Approved methods of contraception include: oral hormonal contraceptives associated with inhibition of ovulation, intrauterine device, medically acceptable barrier methods (diaphragm or condom), implantable or injectable contraceptives or removable birth control device intrauterine hormone-releasing system; and/or sterilization bilateral tubal occlusion (at least 6 months before dosing). Subjects practicing abstinence and coitus interruptus (pull out method) must agree to use an approved method of contraception during the study and 60 days after their last dose of study drug.	Section 6.4 Approved Methods of Contraception
"Endothelial cell count" was removed as an assessment that will be provided to the Reading Center for objective assessment of subject eligibility.<br>"Optical Coherence Tomography Angiography (OCT-A, selected sites only)" was added as an assessment that will be provided to the Reading Center for objective assessment of subject eligibility.	Section 7.1 Allocation to Treatment<br>Section 8.1. Study Visit Schedule
"Country" was removed as a stratification factor.	Section 7.1 Allocation to Treatment

References to “DSMB” updated to “DMC”.	Section 7.2.1 Unmasking<br><br>Section 10.1 Data Monitoring Committee
Reference to preparing APL-2 for injection was removed from the paragraph describing sham.	Section 7.3.3.1 Identity of the Investigational Product
Text was updated to indicate that:<br><br>The suspected onset or presence of new active choroidal neovascularization (CNV) secondary to AMD in the study eye and/or fellow eye must be documented in the source document and CRF.	Section 7.5.1 Treatment of New Active Choroidal Neovascularization in the Study Eye and/or Fellow Eye<br><br>Section 9.15 Ocular Imaging
Optical Coherence Tomography Angiography (OCT-A) was added as a monitoring assessment for select sites to confirm the diagnosis of new active CNV.	Section 7.5.1 Treatment of New Active Choroidal Neovascularization in the Study Eye and/or Fellow Eye<br><br>Section 9.15 Ocular Imaging
Text corrected as follows:<br><br>The treatment method (pars plana vitrectomy vs. intravitreal injection of antibiotics vitreous tap) and choice of antimicrobial agents are also at the discretion of the physician and should follow current standard practice patterns.	Section 7.5.3 Endophthalmitis Treatment
References to “biobanking” have been replaced by references to the “genetic biorepository” and the “clinical repository”.	Section 8. STUDY DESIGN<br><br>Section 9.7 Genotyping Samples<br><br>Section 9.17 Blood Volume for Study Assessments
Noted that blood draws will be conducted for genotyping.	Section 8.1.3.2 Months 13-24
Text was modified to clarify that the study staff performing visual acuity should be masked to the treatment assignment only; the protocol previously stated that staff should also be blinded to the study eye.	Section 9.11 Best-corrected Visual Acuity and Low Luminance Best-corrected Visual Acuity
Blood volume for genetic biorepository was updated.	Section 9.17 Blood Volume for Study Assessments
The following samples were removed:<br><br>a. All residual serum and whole blood samples collected during the course of the study for PK analysis, genotyping and anti-APL-2 antibody formation<br><br>b. Residual serum PK sample<br><br>c. Residual serum Anti-Therapeutic Antibodies (ATA) sample	Section 9.18.2 Sample Collection

It was noted that the 15 mL whole blood sample will be collected at Month 2, baseline, Month 12 and Month 24 of the study	
It was clarified that the DMC will meet at the beginning of the study and every 6 months thereafter.	Section 10.1 Data Monitoring Committee
Reference to "possibly related" AEs was removed as there is no "possibly related" AE categorization for this study.<br><br>Resolution outcome possibilities were updated to accurately align with the SAE report forms that will be used for this study.	Section 11.2 Recording Adverse Events<br><br>Section 11.4.1 Relationship of Events to Study Treatment
Clarified that:<br><br>All SAEs will be reported to the Safety Monitor by the Investigator via fax or email within one working calendar day of becoming aware of the event, whether or not the serious events are deemed drug-related.	Section 11.5 Serious Adverse Events
Section reorganized, modified, and updated to depict protocol specifications for data management and statistical considerations more clearly, accurately, and thoroughly.	Section 12. DATA MANAGEMENT AND STATISTICAL CONSIDERATIONS
Footnote was updated to indicate that: **All assessments should be performed on the same day, with the exception of screening.**<br><br>Frequency of microperimetry and endothelial cell count was updated. Microperimetry footnote was updated to remove reference to the study eye as that assessment will now be conducted on both eyes.<br><br>The blood draw for the clinical repository was removed at baseline, Month 1, Month 8, Month 18, and Month 27.<br><br>OCT-A was added at screening, Month 6, Month 12, Month 18, and Month 24 for select sites. Footnote "S" was added to note that in addition to the time points indicated on the study schedule, OCT-A should be performed at the time of any suspected new active CNV. If new active CNV is confirmed in the study eye, OCT-A should be repeated every 2 months for the study eye.	All Study Schedules (Appendices: A, B, C, and D)

APPENDIX K: PROTOCOL CHANGES TO BE FOLLOWED DURING COVID-19 RESTRICTIONS

OVERVIEW

In response to the COVID-19 crisis, to ensure the safety of study subjects and Investigative Sites as well as proper conduct of the study, TEMPORARY changes to the protocol have been implemented. These changes should be followed only during COVID-19 restrictions and include extended IP administration windows, changes to masking rules, rescreening instructions, and a revised schedule of assessments.

Where feasible, sites could continue to follow the full schedule of assessments (based on their treatment group assignment).

EXTENDED IP ADMINISTRATION WINDOWS

In order to allow more flexibility to sites and subjects, and to potentially mitigate missed IP administration, an extended IP administration window can be followed. The extended window can ONLY be used in situations related to COVID-19 restrictions and after medical monitor approval. Footnote "T" of each COVID-19 assessment table below reflects these extended IP options.

Per protocol, the IP administration window is as follows:

- Monthly treatment group: +/– 8 days for the entire study duration
- EOM treatment group: +/– 8 days for the first study year and +/- 16 days for the second study year

During COVID-19 restrictions, the IP administration window can be extended to the following:

- Monthly treatment group: –8 days to +15 days. Note, interval for consecutive injections must be at least 14 days.
- EOM treatment group: –8 days to +30 days for the first study year and –16 days to +30 days for the second study year.

MASKING RULES

Due to current COVID-19 restrictions, clinical sites might encounter difficulties maintaining appropriate clinic staffing to satisfy the approved masking rules for the APL2-303 (Derby) study. Based on this, and in an attempt to minimize the amount of missed data and IP administrations, Apellis is implementing a temporary adjustment to the study masking rules.

This temporary change must be approved by the Apellis Medical Director **prior to** implementation and must be documented via a temporary and modified delegation of authority Log. Each masked assessment performed by an unmasked staff and vice versa (even with Apellis approval and following the below guidelines) should be documented.

The principal investigator (PI) is responsible for the overall oversight of the study site data and s/he will not be allowed to switch into an unmasked role. Every masked individual that performs IP administration and/or postinjection assessment (all unmasked assessment) as a temporary measure, will **permanently** be considered an unmasked individual and will not be able to perform masked assessments once these exemptions are lifted.

RESCREENING PROCEDURE

Prior to the implementation of these temporary changes, sites continuing to screen patients have been encouraged to complete the screening and baseline assessments in their entirety. However, if a subject was deemed a screen failure for not being able to meet the original screening window (Day –28 to Day –1 [+/- 2 days]) due to COVID-19 related restrictions, a rescreening visit is allowed and should be followed according to the 2 scenarios below.

Subjects Who Completed Screening and Were Considered Eligible by Reading Center and Investigator

Subjects who were screened prior to 30 March 2020 and completed all screening assessments (as described in the Schedule of Assessments Appendix A [every month treatment group] and Appendix C [every other month [EOM] treatment group]) and considered eligible by the reading center and investigator and are able to return to the clinic within 90 days of initial screening, will receive a new subject ID number and undergo an *abbreviated screening*, prior to randomization, that includes the following assessments:

- Informed consent/assign new screening number
- Normal luminance best corrected visual acuity assessment
- Slitlamp examination
- Dilated indirect ophthalmoscopy
- IOP measurement
- SD-OCT*
- Concomitant medication/concomitant ocular procedures collection
- AE collection

*SD-OCT images collected at this visit will not be used by the reading center to determine eligibility but should be used by the investigator to detect any potential new exclusion criterion.

If the investigator deems it necessary, additional assessments can be performed if there is a concern that the subject might now meet an exclusion criterion that was not the case during the original screening (eg, FFA to exclude the presence of CNV).

Subjects With Incomplete Screening Assessment

Subjects that signed the informed consent but were not able to complete all screening assessments due to COVID-19-related restrictions are not eligible for the abbreviated screening. These subjects can be rescreened but must follow the standard screening schedule of assessment (as described in the Schedule of Assessments Appendix A [every month treatment group] and Appendix C [EOM treatment group]). These subjects will also receive a new screening ID number.

MINIMUM SCHEDULE OF ASSESSMENT

Schedule of Assessments

Where feasible, sites could continue to follow the full schedule of assessments (based on their treatment group assignment). The minimum assessment tables, only to be followed during this

COVID-19 effort and if determined necessary to use based on the investigator's clinical judgment, are provided below to reduce the time required for each study visit. Subjects in the EOM treatment group do not need to be seen for the non-IP administration visits. Assessments not performed (even those that have been removed in the minimum assessment table) should be documented.

Subjects that are not able to come into the clinic for a study visit due to COVID-19-related restrictions, including visits for the EOM group that do not include IP administration, should be contacted via the phone for the collection of AEs (including SAEs) and concomitant medications. **All SAEs are still required to be reported to Apellis within 24 hours of site awareness, even if reported via phone call**. All communications via phone call should also be documented in the source documents and in the respective CRF page. In addition, these subjects should be instructed to self-monitor their vision at home and report any changes in vision or their overall health via phone call. The site must inform the Sponsor of any subjects lost to follow-up.

It is critical that local, country, and regional governance regarding COVID-19 is followed along with your best clinical judgment when managing this situation. All visits or assessments missed as a result of COVID-19 will be captured in the case report forms.

The Schedule of Assessments tables shown below supersede those sent on 30 March 2020. These changes were previously communicated to the sites via a memorandum.

APPENDIX A (TRACKED): COVID-19 VISIT SCHEDULE—Monthly Group—Screening, Day 1 Through Month 12

	Screening	Treatment														
**Visit #**	**1**	**2**	**2A**	**3**	**4**	**5**	**6**	**7**	**8**	**9**	**10**	**11**	**12**	**13**	**14**	**Early Term[A]**
**Day**	**–28 to –1**	**1**	**7**	**30**	**60**	**90**	**120**	**150**	**180**	**210**	**240**	**270**	**300**	**330**	**360**	
**Week**	**0**	**0**	**1**	**4**	**8**	**12**	**16**	**20**	**24**	**28**	**32**	**36**	**40**	**44**	**48**	
**Month**	**0**	**0**	**0**	**1**	**2**	**3**	**4**	**5**	**6**	**7**	**8**	**9**	**10**	**11**	**12**	
**Window (+ or – days)**	**2**	**0**	**1**	**8**	**8**	**8**	**8**	**8**	**8**	**8**	**8**	**8**	**8**	**8**	**8**	
Informed Consent/Assign Screening Number	x															
Demographic Data	x															
Inclusion/Exclusion Criteria [B]	x	x														
Medical/Surgical/Ocular History [C]	x															
Blood Draw—Safety Labs [D,E,F]	x	x			~~x~~				~~x~~						x	x
Urine Sample Collection [D, E,F]	x	x			~~x~~				~~x~~						x	x
Urine Pregnancy Test [D,E,F]		x		x	x	x	x	x	x	x	x	x	x	x	x	
Blood Draw—PK and Complement Profile (C3, CH50, AH50) [D, S]		x	x	x					x						x	x
Blood Draw—Genotyping (if applicable) [D]					~~x~~											
Blood Draw- Anti-Pegcetacoplan Ab [D]		x		~~x~~	~~x~~				~~x~~						x	x
Blood Draw for Clinical Repository (if applicable)[D,G]					~~x~~				~~x~~						~~x~~	~~x~~
Vital Signs [H]	x	x		~~x~~	~~x~~	~~x~~	~~x~~	~~x~~	x	~~x~~	~~x~~	~~x~~	~~x~~	~~x~~	x	x
Physical Examination [I]	x														x	x
BCVA [J]	x	x		x	x	x	x	x	x	x	x	x	x	x	x	x
LL-BCVA [J]		x		~~x~~	~~x~~	~~x~~	~~x~~	~~x~~	x	~~x~~	~~x~~	~~x~~	~~x~~	~~x~~	x	x
MNREAD or Radner Reading Charts (select countries) [J,K]		x							~~x~~						x	x
Slitlamp Examination	x	x		x	x	x	x	x	x	x	x	x	x	x	x	x
Endothelial Cell Count[S]		x							x						x	x
NEI VFQ-25 [L]		x							~~x~~						x	x

APPENDIX A (TRACKED): COVID-19 VISIT SCHEDULE—Monthly Group—Screening, Day 1 Through Month 12

	Screening	Treatment														Early Term[A]
**Visit #**	**1**	**2**	**2A**	**3**	**4**	**5**	**6**	**7**	**8**	**9**	**10**	**11**	**12**	**13**	**14**	
**Day**	**–28 to –1**	**1**	**7**	**30**	**60**	**90**	**120**	**150**	**180**	**210**	**240**	**270**	**300**	**330**	**360**	
**Week**	**0**	**0**	**1**	**4**	**8**	**12**	**16**	**20**	**24**	**28**	**32**	**36**	**40**	**44**	**48**	
**Month**	**0**	**0**	**0**	**1**	**2**	**3**	**4**	**5**	**6**	**7**	**8**	**9**	**10**	**11**	**12**	
**Window (+ or – days)**	**2**	**0**	**1**	**8**	**8**	**8**	**8**	**8**	**8**	**8**	**8**	**8**	**8**	**8**	**8**	
FRI [L]		x							~~x~~						x	x
Home-Based Digital Applications [L,M S]		x		~~x~~	~~x~~	~~x~~			~~x~~						x	
Dilated Indirect Ophthalmoscopy	x	x		x	x	x	x	x	x	x	x	x	x	x	x	x
IOP Measurement	x	x		x	x	x	x	x	x	x	x	x	x	x	x	x
SD-OCT [N]	x	x		~~x~~	~~x~~	~~x~~	~~x~~	~~x~~	x	~~x~~	~~x~~	~~x~~	~~x~~	~~x~~	x	x
FAF [N]	x	x			~~SE~~		~~SE~~		x		~~SE~~		~~SE~~		x	x
NIR [N]	x	x			~~SE~~		~~SE~~		x		~~SE~~		~~SE~~		x	x
DCFP [N]	x														x	x
FFA [N]	x														x	x
OCT-A [R S]		x [R]							~~x R~~						x [R]	x
Study Eye Determination	x															
Randomization		x														
Pegcetacoplan administration or Sham Injection[T]		x		x	x	x	x	x	x	x	x	x	x	x	x	
Postinjection Assessment [O]		x		x	x	x	x	x	x	x	x	x	x	x	x	
Follow-Up Call [P]		x		x	x	x										
Concomitant Medication/Concomitant Ocular Procedures [Q]	x	x	~~x~~	x	x	x	x	x	x	x	x	x	x	x	x	x
Adverse Events	x	x	~~x~~	x	x	x	x	x	x	x	x	x	x	x	x	x

APPENDIX A (TRACKED): COVID-19 VISIT SCHEDULE—Monthly Group—Screening, Day 1 Through Month 12

Abbreviations: AH50 = alternative pathway of complement functional test; BCVA = best corrected visual acuity; CH50 = classical pathway of complement functional test; CNV = choroidal neovascularization; DCFP = digital color fundus photography; eCRF = electronic case report form; FAF = fundus autofluorescence; FFA = fundus fluorescein angiography; FRI = Functional Reading Independence Index; IOP = intraocular pressure; LL-BCVA = low luminance best corrected visual acuity; MNREAD = Minnesota Low-Vision Reading Test; NEI VFG-25 = National Eye Institute Visual Functioning Questionnaire 25-Item Version; NIR = near infrared reflectance; OCT-A = optical coherence tomography angiography; PK = pharmacokinetics; SD-OCT = spectral domain optical coherence tomography; SE = study eye; Term = termination; VEGF = vascular endothelial growth factor.

Note: All ocular assessments are to be performed for both eyes unless annotated with 'SE' (study eye) in the above schedule. All assessments should be performed on the same day. All study visits should be scheduled and projected based on the Day 1 visit date with the exception of Visit 2a which should be based on the Day 1 dose date.

A. For subjects that discontinue the study early, the early termination assessments should be performed after a minimum of 30 days have passed from the last dosing visit. If a subject reports for a scheduled visit and decides to terminate early prior to dosing, the visit should be considered the early termination visit and all early termination procedures should be performed. At Month 24, all subjects should be offered entry into an open-label study.

B. At Day 1 (Visit 2), confirm subject eligibility through reviewing the inclusion/exclusion criteria and receive confirmation of eligibility from the reading center.

C. Significant medical/surgical history from the previous 5 years. Anti-VEGF treatments (fellow eye) and invasive ocular procedures performed within the past 5 years and while on study should also be recorded. Any history of tobacco use should be recorded.

D. Obtain prior to fluorescein angiography and before study drug administration.

E. At screening, serum pregnancy should be performed for women of childbearing potential. If positive, subject is not eligible to continue in the study.

F. Beginning at Day 1, perform the urine pregnancy test for women of childbearing potential at each treatment visit. If positive, perform a serum pregnancy test. If serum test is positive, study drug should not be administered and an early term visit should be completed.

G. Only subjects that sign the separate consent for the clinical repository and genotyping will have these samples collected. A 14-mL whole-blood sample will be collected at each of the specified visits.

H. Blood pressure, respiratory rate, heart rate, and temperature. On dosing days, vital signs should be taken predose.

I. Height and weight should be collected at screening.

J. Perform assessments prior to dilating the eyes.

K. In select countries, the MNREAD or Radner Reading Charts should be done during the study visit, prior to dilating the eyes. It should be performed monocularly first, then binocularly.

L. To be administered by the masked site staff prior to any other assessments performed on that day. In-clinic assessments on the digital application should be completed after completion of all functional tests and quality of life measures prior to dilating the eyes.

M. At select sites, and for those subjects who decide to participate, the digital applications will be completed on an electronic device and will consist of assessments for visual function and reading speed. Subjects will receive training at Day 1 on the use of the digital applications and the electronic device and will perform the tests using the application in the clinic at Month 1, Month 2, Month 3, Month 6, Month 12, Month 18, and Month 24. Beginning at Day 1, subjects will complete the functional assessments weekly at home. Subjects should be instructed to complete the assessments on the same day each week and at approximately the same time of day each week, if possible.

N. FAF and SD-OCT images, near infrared reflectance, fluorescein angiograms, and fundus photographs will be performed for the study eye only on days where 'SE' is specified and for both eyes at all other visits as specified in the above schedule and will be sent to the reading center for evaluation (consult the reading center manual for specifics on image capture, processing, and transmission). Images should be captured prior to dosing on dosing days. If a subject misses a study visit or images cannot be obtained at a specific visit, study staff should make every effort to obtain images at the next scheduled visit. If new active CNV is suspected, SD-OCT, FFA, and OCT-A (select sites) images must be collected and sent to the reading center for analysis.

O. Postinjection assessments should be performed within 5 minutes after dosing by the unmasked physician or study staff and should include a gross assessment of vision (finger-counting, hand motion, then light perception when applicable). If subject passes gross vision test, the subject may leave the site. If subject fails gross vision test, the tonometry should be performed. IOP should be ≤30 mm Hg in order for the subject to leave the site. If necessary, antiglaucomatous medication can be given to lower the IOP. If IOP is >30 mm Hg, assessments will continue every approximately 30 minutes from the previous measurement until the subject passes gross vision test and IOP is ≤30 mm Hg. Note: if the study eye is treated with a ranibizumab or aflibercept injection during the same visit as the study treatment (pegcetacoplan or sham), the treatment with ranibizumab or aflibercept must be performed first. The pre- and post- anti-VEGF IOP values must be measured and recorded on the eCRF.

APPENDIX A (TRACKED): COVID-19 VISIT SCHEDULE—Monthly Group—Screening, Day 1 Through Month 12

P. Starting at Day 1, study subjects will be contacted by study site staff within 4 ± 2 days after each study treatment visit (through Month 3) to collect any information on any safety concerns, decrease in vision, eye pain, unusual ocular events, or any new ocular symptoms in the study eye. If the physician determines that there are any safety concerns, a follow-up visit should be scheduled as soon as possible.

Q. Record concomitant medications (ie, prescription and over-the-counter medications) used by the patient within 30 days of screening and throughout the subject's participation in the study.

R. In addition to the time points indicated on the study schedule, OCT-A should be performed at the time of any suspected new active CNV. If new active CNV is confirmed in the study eye, OCT-A should be repeated every 2 months for the study eye.

S. At select sites only.

T. Administration of study treatment (pegcetacoplan or sham) can be done on a separate day from the assessment visit if both days fall within the visit window. If this occurs on the randomization visit, then the administration of pegcetacoplan or sham should be done within 3 days of randomization and after approval from the medical monitor. When study treatment administration is on a day other than a study visit, then the only assessment that must be done on the day of study treatment administration is the preinjection IOP. **During the COVID-19 pandemic, the following flexibility is allowed for IP administration: –8 days to +15 days after medical monitor approval. Note, interval for consecutive injections must be at least 14 days.**

APPENDIX B (TRACKED): COVID-19 VISIT SCHEDULE—Monthly Group—Month 13 to Month 24

	Treatment												Early Term [A]
**Visit #**	**15**	**16**	**17**	**18**	**19**	**20**	**21**	**22**	**23**	**24**	**25**	**26**	
**Day**	**390**	**420**	**450**	**480**	**510**	**540**	**570**	**600**	**630**	**660**	**690**	**720**	
**Week**	**52**	**56**	**60**	**64**	**68**	**72**	**76**	**80**	**84**	**88**	**92**	**96**	
**Month**	**13**	**14**	**15**	**16**	**17**	**18**	**19**	**20**	**21**	**22**	**23**	**24**	
**Window (+ or – days)**	**8**	**8**	**8**	**8**	**8**	**8**	**8**	**8**	**8**	**8**	**8**	**8**	
Informed Consent/Assign Screening Number													
Demographic Data													
Inclusion/Exclusion Criteria [B]													
Medical/Surgical/Ocular History [C]													
Blood Draw—Safety Labs [D,E,F]						~~x~~						x	x
Urine Sample Collection [D,E,F]						~~x~~						x	x
Urine Pregnancy Test [D,E,F]	x	x	x	x	x	x	x	x	x	x	x	x	
Blood Draw—PK and Complement Profile (C3, CH50, AH50) [D,S]												x	x
Blood Draw—Genotyping (if applicable) [D]													
Blood Draw—Anti-Pegcetacoplan Ab [D]		~~x~~				~~x~~						x	x
Blood Draw for Clinical Repository (if applicable)[D,G]												~~x~~	~~x~~
Vital Signs [H]	~~x~~	~~x~~	~~x~~	~~x~~	~~x~~	x	~~x~~	~~x~~	~~x~~	~~x~~	~~x~~	x	x
Physical Examination [I]												x	x
BCVA [J]	x	x	x	x	x	x	x	x	x	x	x	x	x
LL-BCVA [I]	~~x~~	~~x~~	~~x~~	~~x~~	~~x~~	x	~~x~~	~~x~~	~~x~~	~~x~~	~~x~~	x	x
MNREAD or Radner Reading Charts (select countries) [J,K]						~~x~~						x	x
Slitlamp Examination	x	x	x	x	x	x	x	x	x	x	x	x	x
Endothelial Cell Count[S]												x	x
NEI VFQ-25 [L]						~~x~~						x	x
FRI [L]						~~x~~						x	x
Dilated Indirect Ophthalmoscopy	x	x	x	x	x	x	x	x	x	x	x	x	x

APPENDIX B (TRACKED): COVID-19 VISIT SCHEDULE—Monthly Group—Month 13 to Month 24

	Treatment												Early Term [A]
**Visit #**	**15**	**16**	**17**	**18**	**19**	**20**	**21**	**22**	**23**	**24**	**25**	**26**	
**Day**	**390**	**420**	**450**	**480**	**510**	**540**	**570**	**600**	**630**	**660**	**690**	**720**	
**Week**	**52**	**56**	**60**	**64**	**68**	**72**	**76**	**80**	**84**	**88**	**92**	**96**	
**Month**	**13**	**14**	**15**	**16**	**17**	**18**	**19**	**20**	**21**	**22**	**23**	**24**	
**Window (+ or – days)**	**8**	**8**	**8**	**8**	**8**	**8**	**8**	**8**	**8**	**8**	**8**	**8**	
Home-Based Digital Applications [L,M,S]						~~x~~						~~x~~	
IOP Measurement	x	x	x	x	x	x	x	x	x	x	x	x	x
SD-OCT [N]	~~x~~	~~x~~	~~x~~	~~x~~	~~x~~	x	~~x~~	~~x~~	~~x~~	~~x~~	~~x~~	x	x
FAF [N]		~~SE~~		~~SE~~		x		~~SE~~		~~SE~~		x	x
NIR [N]		~~SE~~		~~SE~~		x		~~SE~~		~~SE~~		x	x
DCFP [N]												x	x
FFA [N]												x	x
OCT-A [R,S]						~~x [R]~~						x [R]	x
Study Eye Determination													
Randomization													
Pegcetacoplan administration or Sham Injection[T]	x	x	x	x	x	x	x	x	x	x	x		
Postinjection Assessment [O]	x	x	x	x	x	x	x	x	x	x	x		
Follow-Up Call [P]													
Concomitant Medication/Concomitant Ocular Procedures [Q]	x	x	x	x	x	x	x	x	x	x	x	x	x
Adverse Events	x	x	x	x	x	x	x	x	x	x	x	x	x

Abbreviations: AH50 = alternative pathway of complement functional test; BCVA = best corrected visual acuity; CH50 = classical pathway of complement functional test; CNV = choroidal neovascularization; DCFP = digital color fundus photography; eCRF = electronic case report form; FAF = fundus autofluorescence; FFA = fundus fluorescein angiography; FRI = Functional Reading Independence Index; IOP = intraocular pressure; LL-BCVA = low luminance best corrected visual acuity; MNREAD = Minnesota Low-Vision Reading Test; NEI VFG-25 = National Eye Institute Visual Functioning Questionnaire 25-Item Version; NIR = near infrared reflectance; OCT-A = optical coherence tomography angiography; PK = pharmacokinetics; SD-OCT = spectral domain optical coherence tomography; SE = study eye; Term = termination; VEGF = vascular endothelial growth factor.

Note: All ocular assessments are to be performed for both eyes unless annotated with 'SE' (study eye) in the above schedule. All assessments should be performed on the same day. All study visits should be scheduled and projected based on the Day 1 visit date.

APPENDIX B (TRACKED): COVID-19 VISIT SCHEDULE—Monthly Group—Month 13 to Month 24

A. For subjects that discontinue the study early, the early termination assessments should be performed after a minimum of 30 days have passed from the last dosing visit. If a subject reports for a scheduled visit and decides to terminate early prior to dosing, then the visit should be considered the early termination visit and all early termination procedures should be performed. At Month 24, all subjects should be offered entry into an open-label study.
B. At Day 1 (Visit 2), confirm subject eligibility through reviewing the inclusion/exclusion criteria and receive confirmation of eligibility from the reading center.
C. Significant medical/surgical history from the previous 5 years. Anti-VEGF treatments (fellow eye) and invasive ocular procedures performed within the past 5 years and while on study should also be recorded. Any history of tobacco use should be recorded.
D. Obtain prior to fluorescein angiography and before study drug administration.
E. At screening, serum pregnancy should be performed for women of childbearing potential. If positive, subject is not eligible to continue in the study.
F. Beginning at Day 1, perform the urine pregnancy test for women of childbearing potential at each treatment visit. If positive, perform a serum pregnancy test. If serum test is positive, study drug should not be administered and an early term visit should be completed.
G. Only subjects that sign the separate consent for the clinical repository and genotyping will have these samples collected. A 14-mL whole-blood sample will be collected at each of the specified visits.
H. Blood pressure, respiratory rate, heart rate, and temperature. On dosing days, vital signs should be taken predose.
I. Height and weight should be measured at screening.
J. Perform assessments prior to dilating the eyes.
K. In select countries, the MNREAD or Radner Reading Charts should be done during the study visit, prior to dilating the eyes. It should be performed monocularly first, then binocularly.
L. To be administered by the masked site staff prior to any other assessments performed on that day. In-clinic assessments on the digital application should be completed after completion of all functional tests and quality of life measures prior to dilating the eyes.
M. At select sites, and for those subjects who decide to participate, the digital applications will be completed on an electronic device and will consist of assessments for visual function and reading speed. Subjects will receive training at Day1 on the use of the digital applications and the electronic device and will perform the tests using the application in the clinic at Month 1, Month 2, Month 3, Month 6, Month 12, Month 18, and Month 24. Beginning at Day 1, subjects will complete the functional assessments weekly at home. Subjects should be instructed to complete the assessments on the same day each week and at approximately the same time of day each week, if possible.
N. FAF and SD-OCT images, near infrared reflectance, fluorescein angiograms, and fundus photographs will be performed for the study eye only on days where 'SE' is specified and for both eyes at all other visits as specified in the above schedule and will be sent to the reading center for evaluation (consult the reading center manual for specifics on image capture, processing, and transmission). Images should be captured prior to dosing on dosing days. If a subject misses a study visit or images cannot be obtained at a specific visit, study staff should make every effort to obtain images at the next scheduled visit. If new active CNV is suspected, SD-OCT, FFA, and OCT-A (selected sites) images should be collected and sent to the reading center for analysis.
O. Postinjection assessments should be performed within 5 minutes after dosing by the unmasked physician or study staff and should include a gross assessment of vision (finger-counting, hand motion, then light perception when applicable). If subject passes gross vision test, the subject may leave the site. If subject fails gross vision test, the tonometry should be performed. IOP should be ≤30 mm Hg in order for the subject to leave the site. If necessary, antiglaucomatous medication can be given to lower the IOP. If IOP is >30 mm Hg, assessments will continue every approximately 30 minutes from the previous measurement until the subject passes gross vision test and IOP is ≤30 mm Hg. Note: if the study eye is treated with a ranibizumab or aflibercept injection during the same visit as the study treatment (pegcetacoplan or sham), the treatment with ranibizumab or aflibercept must be performed first. The pre- and post- anti-VEGF IOP values must be measured and recorded on the eCRF.
P. Starting at Day 1, study subjects will be contacted by study site staff within 4 ± 2 days after each study treatment visit (through Month 3) to collect any information on any safety concerns, decrease in vision, eye pain, unusual ocular events, or any new ocular symptoms in the study eye. If the physician determines that there are any safety concerns, a follow-up visit should be scheduled as soon as possible.
Q. Record concomitant medications (ie, prescription and over-the-counter medications) used by the patient within 30 days of screening and throughout the subject's participation in the study.
R. In addition to the time points indicated on the study schedule, OCT-A should be performed at the time of any suspected new active CNV. If new active CNV is confirmed in the study eye, OCT-A should be repeated every 2 months for the study eye.
S. At select sites only.

APPENDIX B (TRACKED): COVID-19 VISIT SCHEDULE—Monthly Group—Month 13 to Month 24

T. Administration of pegcetacoplan or sham can be done on separate days from the assessment visit if both days fall within the visit window. Administration of study treatment (pegcetacoplan or sham) can be done on a separate day from the assessment visit if both days fall within the visit window. If this occurs on the randomization visit, then the administration of pegcetacoplan or sham should be done within 3 days of randomization and after approval from the medical monitor. When study treatment administration is on a day other than a study visit, then the only assessment that must be done on the day of study treatment administration is preinjection IOP. During the COVID-19 pandemic, the following flexibility is allowed for IP administration: –8 days to +15 days after medical monitor approval. Note, interval for consecutive injections must be at least 14 days.

APPENDIX C (TRACKED): COVID-19 VISIT SCHEDULE—Every-Other-Month Group—Screening, Day 1 Through Month 12

	Screening	Treatment														Early Term[A]
**Visit #**	**1**	**2**	**2A**	**3**	**4**	**5**	**6**	**7**	**8**	**9**	**10**	**11**	**12**	**13**	**14**	
**Day**	**–28 to –1**	**1**	**7**	**30**	**60**	**90**	**120**	**150**	**180**	**210**	**240**	**270**	**300**	**330**	**360**	
**Week**	**0**	**0**	**1**	**4**	**8**	**12**	**16**	**20**	**24**	**28**	**32**	**36**	**40**	**44**	**48**	
**Month**	**0**	**0**	**0**	**1**	**2**	**3**	**4**	**5**	**6**	**7**	**8**	**9**	**10**	**11**	**12**	
**Window (+ or – days)**	**2**	**0**	**1**	**8**	**8**	**8**	**8**	**8**	**8**	**8**	**8**	**8**	**8**	**8**	**8**	
Informed Consent/Assign Screening Number	x															
Demographic Data	x															
Inclusion/Exclusion Criteria [B]	x	x														
Medical/Surgical/Ocular History [C]	x															
Blood Draw—Safety Labs [D,E,F]	x	x			~~x~~				~~x~~						x	x
Urine Sample Collection [D,E,F]	x	x			~~x~~				~~x~~						x	x
Urine Pregnancy Test [D,E,F]		x			x		x		x		x		x		x	
Blood Draw—PK and Complement Profile (C3, CH50, AH50) [D,S]		x	x	x					x						x	x
Blood Draw—Genotyping (if applicable) [D]					~~x~~											
Blood Draw- Anti-Pegcetacoplan Ab [D]		x		~~x~~	~~x~~				~~x~~						x	x
Blood Draw for Clinical Repository (if applicable) [D,G]					~~x~~				~~x~~						~~x~~	x
Vital Signs [H]	x	x		~~x~~	~~x~~	~~x~~	~~x~~	~~x~~	x	~~x~~	~~x~~	~~x~~	~~x~~	~~x~~	x	x
Physical Examination [I]	x														x	x
BCVA [J]	x	x		~~x~~	x	~~x~~	x	~~x~~	x	~~x~~	x	~~x~~	x	~~x~~	x	x
LL-BCVA [J]		x		~~x~~	~~x~~	~~x~~	~~x~~	~~x~~	x	~~x~~	~~x~~	~~x~~	~~x~~	~~x~~	x	x
MNREAD or Radner Reading Charts (select countries) [J,K]		x							~~x~~						x	x
Slitlamp Examination	x	x		~~x~~	x	~~x~~	x	~~x~~	x	~~x~~	x	~~x~~	x	~~x~~	x	x
Endothelial Cell Count[S]		x							x						x	x
NEI VFQ-25 [L]		x							~~x~~						x	x
FRI [L]		x							~~x~~						x	x

APPENDIX C (TRACKED): COVID-19 VISIT SCHEDULE—Every-Other-Month Group—Screening, Day 1 Through Month 12

	Screening	Treatment														Early Term[A]
**Visit #**	**1**	**2**	**2A**	**3**	**4**	**5**	**6**	**7**	**8**	**9**	**10**	**11**	**12**	**13**	**14**	
**Day**	**–28 to –1**	**1**	**7**	**30**	**60**	**90**	**120**	**150**	**180**	**210**	**240**	**270**	**300**	**330**	**360**	
**Week**	**0**	**0**	**1**	**4**	**8**	**12**	**16**	**20**	**24**	**28**	**32**	**36**	**40**	**44**	**48**	
**Month**	**0**	**0**	**0**	**1**	**2**	**3**	**4**	**5**	**6**	**7**	**8**	**9**	**10**	**11**	**12**	
**Window (+ or – days)**	**2**	**0**	**1**	**8**	**8**	**8**	**8**	**8**	**8**	**8**	**8**	**8**	**8**	**8**	**8**	
Home-Based Digital Applications [L,M,S]		x		~~x~~	~~x~~	~~x~~			~~x~~						x	
Dilated Indirect Ophthalmoscopy	x	x		~~x~~	x	~~x~~	x	~~x~~	x	~~x~~	x	~~x~~	x	~~x~~	x	x
IOP Measurement	x	x		~~x~~	x	~~x~~	x	~~x~~	x	~~x~~	x	~~x~~	x	~~x~~	x	x
SD-OCT [N]	x	x		~~x~~	~~x~~	~~x~~	~~x~~	~~x~~	x	~~x~~	~~x~~	~~x~~	~~x~~	~~x~~	x	x
FAF [N]	x	x			~~SE~~		~~SE~~		x		~~SE~~		~~SE~~		x	x
NIR [N]	x	x			~~SE~~		~~SE~~		x		~~SE~~		~~SE~~		x	x
DCFP [N]	x														x	x
FFA [N]	x														x	x
OCT-A[R, S]		x[R]							~~x[R]~~						x[R]	x
Study Eye Determination	x															
Randomization		x														
Pegcetacoplan administration or Sham Injection[T]		x			x		x		x		x		x		x	
Postinjection Assessment [O]		x			x		x		x		x		x		x	
Follow-Up Call [P]		x			x		x									
Concomitant Medication/Concomitant Ocular Procedures [Q]	x	x	x	~~x~~	x	~~x~~	x	~~x~~	x	~~x~~	x	~~x~~	x	~~x~~	x	x
Adverse Events	x	x	x	~~x~~	x	~~x~~	x	~~x~~	x	~~x~~	x	~~x~~	x	~~x~~	x	x

Abbreviations: AH50 = alternative pathway of complement functional test; BCVA = best corrected visual acuity; CH50 = classical pathway of complement functional test; CNV = choroidal neovascularization; DCFP = digital color fundus photography; eCRF = electronic case report form; FAF = fundus autofluorescence; FFA = fundus fluorescein angiography; FRI = Functional Reading Independence Index; IOP = intraocular pressure; LL-BCVA = low luminance best corrected visual acuity; MNREAD = Minnesota Low-Vision Reading Test; NEI VFG-25 = National Eye Institute Visual Functioning Questionnaire 25-Item Version; NIR = near infrared reflectance; OCT-A = optical coherence tomography angiography; PK = pharmacokinetics; SD-OCT = spectral domain optical coherence tomography; SE = study eye; Term = termination; VEGF = vascular endothelial growth factor.

APPENDIX C (TRACKED): COVID-19 VISIT SCHEDULE—Every-Other-Month Group—Screening, Day 1 Through Month 12

Note: All ocular assessments are to be performed for both eyes unless annotated with 'SE' (study eye) in the above schedule. All assessments should be performed on the same day. All study visits should be scheduled and projected based on the Day 1 visit date with the exception of Visit 2a which should be based on the Day 1 dose date.

A. For subjects that discontinue the study early, the early termination assessments should be performed after a minimum of 30 days have passed from the last dosing visit. If a subject reports for a scheduled visit and decides to terminate early prior to dosing, the visit should be considered the early termination visit and all early termination procedures should be performed At Month 24, all subjects should be offered entry into an open-label study.

B. At Day 1 (Visit 2), confirm subject eligibility through reviewing the inclusion/exclusion criteria and receive confirmation of eligibility from the reading center.

C. Significant medical/surgical history from the previous 5 years. Anti-VEGF treatments (fellow eye) and invasive ocular procedures performed within the past 5 years and while on study should also be recorded. Any history of tobacco use should be recorded.

D. Obtain prior to fluorescein angiography and before study drug administration.

E. At screening, serum pregnancy should be performed for women of childbearing potential. If positive, subject is not eligible to continue in the study.

F. Beginning at Day 1, perform the urine pregnancy test for women of childbearing potential at each treatment visit. If positive, perform a serum pregnancy test. If serum test is positive, study drug should not be administered and an early term visit should be completed.

G. Only subjects that sign the separate consent for the clinical repository and genotyping will have these samples collected. A 14-mL whole-blood sample will be collected each of the specified visits.

H. Blood pressure, respiratory rate, heart rate, and temperature. On dosing days, vital signs should be taken predose.

I. Height and weight should be measured at screening.

J. Perform assessments prior to dilating the eyes.

K. In select countries, the MNREAD or Radner Reading Charts should be done during the study visit, prior to dilating the eyes. It should be performed monocularly first, then binocularly.

L. To be administered by the masked site staff prior to any other assessments performed on that day. In-clinic assessments on the digital application should be completed after completion of all functional tests and quality of life measures prior to dilating the eyes.

M. At select sites, and for those subjects who decide to participate, the digital application will be completed on an electronic device and will consist of assessments for visual function and reading speed. Subjects will receive training at Day 1 on the use of the digital applications and the electronic device and will perform the tests using the application in the clinic at Month 1, Month 2, Month 3, Month 6, Month 12, Month 18, and Month 24. Beginning at Day 1, subjects will complete the functional assessments weekly at home. Subjects should be instructed to complete the assessments on the same day each week and at approximately the same time of day each week, if possible.

N. FAF and SD-OCT images, near infrared reflectance, fluorescein angiograms, and fundus photographs will be performed for the study eye only on days where 'SE' is specified and for both eyes at all other visits as specified in the above schedule and will be sent to the reading center for evaluation (consult the reading center manual for specifics on image capture, processing, and transmission). Images should be captured prior to dosing on dosing days. If a subject misses a study visit or images cannot be obtained at a specific visit, study staff should make every effort to obtain images at the next scheduled visit. If new active CNV is suspected, SD-OCT, FFA, and OCT-A (select sites) images should be collected and sent to the reading center for analysis.

O. Post- injection assessments should be performed within 5 minutes after dosing by the unmasked physician or study staff and should include a gross assessment of vision (finger-counting, hand motion, then light perception when applicable). If subject passes gross vision test, the subject may leave the site. If subject fails gross vision test, the tonometry should be performed. IOP should be ≤30 mm Hg in order for the subject to leave the site. If necessary, antiglaucomatous medication can be given to lower the IOP. If IOP is >30 mm Hg, assessments will continue every approximately 30 minutes from the previous measurement until the subject passes gross vision test and IOP is ≤30 mm Hg. Note: if the study eye is treated with a ranibizumab or aflibercept injection during the same visit as the study treatment (pegcetacoplan or sham), the treatment with ranibizumab or aflibercept must be performed first. The pre- and post- anti-VEGF IOP values must be measured and recorded on the eCRF.

P. Starting at Day 1, study subjects will be contacted by study site staff within 4 ± 2 days after each study treatment visit (through Month 4) to collect any information on any safety concerns, decrease in vision, eye pain, unusual ocular events, or any new ocular symptoms in the study eye. If the physician determines that there are any safety concerns, a follow-up visit should be scheduled as soon as possible.

Q. Record concomitant medications (ie, prescription and over-the-counter medications) used by the patient within 30 days of screening and throughout the subject's participation in the study.

APPENDIX C (TRACKED): COVID-19 VISIT SCHEDULE—Every-Other-Month Group—Screening, Day 1 Through Month 12

R. In addition to the time points indicated on the study schedule, OCT-A should be performed at the time of any suspected new active CNV. If new active CNV is confirmed in the study eye, OCT-A should be repeated every 2 months for the study eye.
S. At select sites only.
T. Administration of study treatment (pegcetacoplan or sham) can be done on a separate day from the assessment visit if both days fall within the visit window. If this occurs on the randomization visit, then the administration of pegcetacoplan or sham should be done within 3 days of randomization and after approval from the medical monitor. When study treatment administration is on a day other than a study visit, then the only assessment that must be done on the day of study treatment administration is the preinjection IOP. During the COVID-19 pandemic, the following flexibility is allowed for IP administration: –8 days to +30 days after medical monitor approval.

APPENDIX D (TRACKED): COVID-19 VISIT SCHEDULE—Every-Other-Month Group—Month 13 to Month 24													
	**Treatment**												**Early Term** [A]
**Visit #**		**15**		**16**		**17**		**18**		**19**		**20**	
**Day**	**390**	**420**	**450**	**480**	**510**	**540**	**570**	**600**	**630**	**660**	**690**	**720**	
**Week**	**52**	**56**	**60**	**64**	**68**	**72**	**76**	**80**	**84**	**88**	**92**	**96**	
**Month**	**13**	**14**	**15**	**16**	**17**	**18**	**19**	**20**	**21**	**22**	**23**	**24**	
**Window (+ or – days)**		**16**		**16**		**16**		**16**		**16**		**16**	
Informed Consent/Assign Screening Number													
Demographic Data													
Inclusion/Exclusion Criteria [B]													
Medical/Surgical/Ocular History [C]													
Blood Draw—Safety Labs [D,E,F]						~~x~~						x	x
Urine Sample Collection [D, E,F]						~~x~~						x	x
Urine Pregnancy Test [D,E,F]		x		x		x		x		x		x	
Blood Draw—PK and Complement Profile (C3, CH50, AH50) [D,S]												x	x
Blood Draw—Anti-Pegcetacoplan Ab [D]		~~x~~				~~x~~						x	x
Blood Draw—Genotyping (if applicable) [D]													
Blood Draw for Clinical Repository (if applicable) [D,G]												~~x~~	~~x~~
Vital Signs [H]		~~x~~		~~x~~		x		~~x~~		~~x~~		x	x
Physical Examination [I]												x	x
BCVA [J]		x		x		x		x		x		x	x
LL-BCVA [J]		~~x~~		~~x~~		x		~~x~~		~~x~~		x	x
MNREAD or Radner Reading Charts (select countries) [J,K]						~~x~~						x	x
Slitlamp Examination		x		x		x		x		x		x	x
Endothelial Cell Count [S]												x	x
NEI VFQ-25 [L]						~~x~~						x	x
FRI [L]						~~x~~						x	x
Home-Based Digital Applications [L,M,S]						~~x~~						~~x~~	

APPENDIX D (TRACKED): COVID-19 VISIT SCHEDULE—Every-Other-Month Group—Month 13 to Month 24

	Treatment												Early Term [A]
**Visit #**		**15**		**16**		**17**		**18**		**19**		**20**	
**Day**	**390**	**420**	**450**	**480**	**510**	**540**	**570**	**600**	**630**	**660**	**690**	**720**	
**Week**	**52**	**56**	**60**	**64**	**68**	**72**	**76**	**80**	**84**	**88**	**92**	**96**	
**Month**	**13**	**14**	**15**	**16**	**17**	**18**	**19**	**20**	**21**	**22**	**23**	**24**	
**Window (+ or – days)**		**16**		**16**		**16**		**16**		**16**		**16**	
Dilated Indirect Ophthalmoscopy		x		x		x		x		x		x	x
IOP Measurement		x		x		x		x		x		x	x
SD-OCT [N]		~~x~~		~~x~~		x		~~x~~		~~x~~		x	x
FAF [N]		~~SE~~		~~SE~~		x		~~SE~~		~~SE~~		x	x
NIR [N]		~~SE~~		~~SE~~		x		~~SE~~		~~SE~~		x	x
DCFP [N]												x	x
FFA [N]												x	x
OCT-A [R,S]						~~x[R]~~						x[R]	x
Study Eye Determination													
Randomization													
Pegcetacoplan administration or Sham Injection[T]		x		x		x		x		x			
Postinjection Assessment [O]		x		x		x		x		x			
Follow-Up Call [P]													
Concomitant Medication/Concomitant Ocular Procedures [Q]		x		x		x		x		x		x	x
Adverse Events		x		x		x		x		x		x	x

Abbreviations: AH50 = alternative pathway of complement functional test; BCVA = best corrected visual acuity; CH50 = classical pathway of complement functional test; CNV = choroidal neovascularization; DCFP = digital color fundus photography; eCRF = electronic case report form; FAF = fundus autofluorescence; FFA = fundus fluorescein angiography; FRI = Functional Reading Independence Index; IOP = intraocular pressure; LL-BCVA = low luminance best corrected visual acuity; MNREAD = Minnesota Low-Vision Reading Test; NEI VFG-25 = National Eye Institute Visual Functioning Questionnaire 25-Item Version; NIR = near infrared reflectance; OCT-A = optical coherence tomography angiography; PK = pharmacokinetics; SD-OCT = spectral domain optical coherence tomography; SE = study eye; Term = termination; VEGF = vascular endothelial growth factor.

Note: All ocular assessments are to be performed for both eyes unless annotated with 'SE' (study eye) in the above schedule. All assessments should be performed on the same day. All study visits should be scheduled and projected based on the Day 1 visit date.

APPENDIX D (TRACKED): COVID-19 VISIT SCHEDULE—Every-Other-Month Group—Month 13 to Month 24

A. For subjects that discontinue the study early, the early termination assessments should be performed after a minimum of 30 days have passed from the last dosing visit. If a subject reports for a scheduled visit and decides to terminate early prior to dosing, then the visit should be considered the early termination visit and all early termination procedures should be performed. At Month 24, all subjects should be offered entry into an open label study.
B. At Day 1 (Visit 2), confirm subject eligibility through reviewing the inclusion/exclusion criteria and receive confirmation of eligibility from the reading center.
C. Significant medical/surgical history from the previous 5 years. Anti-VEGF treatments (fellow eye) and invasive ocular procedures performed within the past 5 years and while on study should be recorded. Any history of tobacco use should be recorded.
D. Obtain prior to fluorescein angiography and before study drug administration.
E. At screening, serum pregnancy should be performed for women of childbearing potential. If positive, subject is not eligible to continue in the study.
F. Beginning at Day 1, perform the urine pregnancy test for women of childbearing potential at each treatment visit. If positive, perform a serum pregnancy test. If serum test is positive, study drug should not be administered and an early term visit should be completed.
G. Only subjects that sign the separate consent for the clinical repository and genotyping will have these samples collected. A 14-mL whole-blood sample will be collected at the specified time points.
H. Blood pressure, respiratory rate, heart rate, and temperature. On dosing days, vital signs should be taken pre- dose.
I. Height and weight should be measured at screening.
J. Perform assessments prior to dilating the eyes.
K. In select countries, the MNREAD or Radner Reading Charts should be done during the study visit, prior to dilating the eyes. It should be performed monocularly first, then binocularly.
L. To be administered by the masked site staff prior to any other assessments performed on that day. In-clinic assessments on the digital application should be completed after completion of all functional tests and quality of life measures prior to dilating the eyes.
M. At select sites, and for those subjects who decide to participate, the digital applications will be completed on an electronic device and will consist of assessments for visual function and reading speed. Subjects will receive training at Day 1 on the use of the digital applications and the electronic device and will perform the tests using the application in the clinic at Month 1, Month 2, Month 3, Month 6, Month 12, Month 18, and Month 24. Beginning at Day 1, subjects will complete the functional assessments weekly at home. Subjects should be instructed to complete the assessments on the same day each week and at approximately the same time of day each week, if possible.
N. FAF and SD-OCT images, near infrared reflectance, fluorescein angiograms, and fundus photographs will be performed for the study eye only on days where 'SE' is specified and for both eyes at all other visits as specified in the above schedule and will be sent to the reading center for evaluation (consult the reading center manual for specifics on image capture, processing, and transmission). Images should be captured prior to dosing on dosing days. If a subject misses a study visit or images cannot be obtained at a specific visit, study staff should make every effort to obtain images at the next scheduled visit. If new active CNV is suspected, SD-OCT, FFA, and OCT-A (selected sites) images should be collected and sent to the reading center for analysis.
O. Postinjection assessments should be performed within 5 minutes after dosing by the unmasked physician or study staff and should include a gross assessment of vision (finger-counting, hand motion, then light perception when applicable). If subject passes gross vision test, the subject may leave the site. If subject fails gross vision test, the tonometry should be performed. IOP should be ≤30 mm Hg in order for the subject to leave the site. If necessary, antiglaucomatous medication can be given in order to lower IOP. If IOP is >30 mm Hg, assessments will continue every approximately 30 minutes from the previous measurement until the subject passes the gross vision test and IOP is ≤30 mm Hg. Note: if the study eye is treated with a ranibizumab or aflibercept injection during the same visit as the study treatment (pegcetacoplan or sham), the treatment with ranibizumab or aflibercept must be performed first. The pre- and post- anti-VEGF IOP values must be measured and recorded on the eCRF.
P. Starting at Day 1, study subjects will be contacted by study site staff within 4 ± 2 days after each study treatment visit (through Month 4) to collect any information on any safety concerns, decrease in vision, eye pain, unusual ocular events, or any new ocular symptoms in the study eye. If the investigator determines that there are any safety concerns, a follow-up visit should be scheduled as soon as possible.
Q. Record concomitant medications (ie, prescription and over-the-counter medications) used by the patient within 30 days of screening and throughout the subject's participation in the study.
R. In addition to the time points indicated on the study schedule, OCT-A should be performed at the time of any suspected new active CNV. If new active CNV is confirmed in the study eye, OCT-A should be repeated every 2 months for the study eye.
S. At select sites only.

APPENDIX D (TRACKED): COVID-19 VISIT SCHEDULE—Every-Other-Month Group—Month 13 to Month 24

T. Administration of pegcetacoplan or sham can be done on separate days from the assessment visit if both days fall within the visit window. Administration of study treatment (pegcetacoplan or sham) can be done on a separate day from the assessment visit if both days fall within the visit window. If this occurs on the randomization visit, then the administration of pegcetacoplan or sham should be done within 3 days of randomization and after approval from the medical monitor. When study treatment administration is on a day other than a study visit, then the only assessment that must be done on the day of study treatment administration is preinjection IOP. During the COVID-19 pandemic, the following flexibility is allowed for IP administration: –16 days to +30 days after medical monitor approval.

STATISTICAL ANALYSIS PLAN

APL2-303

A Phase III, Multi-Center, Randomized, Double-Masked, Sham-Controlled Study to Compare the Efficacy and Safety of Intravitreal APL-2 Therapy with Sham Injections in Patients with Geographic Atrophy (GA) Secondary to Age-Related Macular Degeneration (AMD)

PROTOCOL IDENTIFIER: DERBY

Study Sponsor:	Apellis Pharmaceuticals, Inc.<br>100 5th Ave<br>Waltham, MA 02451
Author:	Caleb Bliss
Protocol:	Amendment 5, Version 1.0, 12 August 2020
SAP Version #:	2.0
SAP Date:	10 August 2021
Status:	Final

REVISION HISTORY

Version	Issue Date	Summary of Changes
1.0	07/23/2021	New Document
2.0	08/10/2021	• Changed the thresholds for censoring COVID-19 impacted data in the supplementary analyses for the primary and key secondary endpoints.<br>• Added an additional covariance matrix to the MMRM models if the statistical modeling fails to converge with the first two covariance matrices<br>• Fixed an error in the sample code and text for maximum reading speed calculation.<br>• Fixed an error in Figure 2 with extraneous text boxes included.<br>• Provided further details on the analysis of suspected, confirmed, and exudative AMD's.

APPROVAL SIGNATURES

DocuSigned by:
Caleb Bliss
Signer Name: Caleb Bliss
Signing Reason: I approve this document
Signing Time: 10-Aug-2021 | 12:42 EDT
F3BA2A1310AF4B61B42694F4D40BCC5B

Caleb Bliss, PhD<br>Director, Biostatistics<br>Apellis Pharmaceuticals, Inc.	Date (dd-Mmm-yyyy)

DocuSigned by:
Jason Raines
Signer Name: Jason Raines
Signing Reason: I approve this document
Signing Time: 10-Aug-2021 | 12:44 EDT
DDF9F44C0B7943249C1956ECB8C87617

Jason Raines, MS, MPH<br>Vice President, Biometrics & Data Management<br>Apellis Pharmaceuticals, Inc.	Date (dd-Mmm-yyyy)

DocuSigned by:
Shisheng Li
Signer Name: Shisheng Li
Signing Reason: I approve this document
Signing Time: 10-Aug-2021 | 13:26 EDT
5A71B0B3EFF3483B9684D6926333A6C4

Shisheng Li, PhD<br>Director, Clinical Pharmacology<br>Apellis Pharmaceuticals, Inc.	Date (dd-Mmm-yyyy)

DocuSigned by:
Patrick Murphy
Signer Name: Patrick Murphy
Signing Reason: I approve this document
Signing Time: 10-Aug-2021 | 12:46 EDT
FFAFEE1C127040998DAC2D44E0D1736B

Patrick Murphy, MSc<br>Senior Director, Regulatory Affairs<br>Apellis Ireland Limited	Date (dd-Mmm-yyyy)

DocuSign Envelope ID: 4873FE2C-F62E-4C55-9B53-4046589BF803

DocuSigned by:

Ramiro Ribeiro

Signer Name: Ramiro Ribeiro
Signing Reason: I approve this document
Signing Time: 10-Aug-2021 | 13:28 EDT
1EFAA7344980487D8986A4A6D69E6A7B

Ramiro Ribeiro, MD, PhD<br>Sr. Medical Director, Ophthalmology, Clinical Development<br>Apellis Pharmaceuticals, Inc	Date (dd-Mmm-yyyy)

DocuSigned by:

Federico Grossi

Signer Name: Federico Grossi
Signing Reason: I approve this document
Signing Time: 10-Aug-2021 | 15:27 EDT
A870049663B24A6CB18A128EEA807A6F

Federico Grossi, MD, PhD<br>Chief Medical Officer<br>Apellis Pharmaceuticals, Inc	Date (dd-Mmm-yyyy)

TABLE OF CONTENTS

REVISION HISTORY 2
TABLE OF CONTENTS 5
LIST OF TABLES 10
LIST OF FIGURES 10
ABBREVIATIONS 11
1. INTRODUCTION 13
2. OBJECTIVES AND ENDPOINTS 14
2.1. Objectives 14
2.1.1. Primary Objective 14
2.1.2. Key Secondary Objectives 14
2.1.3. Secondary Objectives 14
2.1.4. Safety Objectives 14
2.1.5. Exploratory Objectives 15
2.2. Endpoints 15
2.2.1. Primary Endpoint 15
2.2.2. Key Secondary Endpoints 15
2.2.3. Secondary Endpoints 15
2.2.4. Exploratory Endpoints 16
2.2.5. Safety Endpoints 17
2.2.6. Exploratory Endpoints Closely Related to the Primary Efficacy Endpoint 17
2.2.7. Pharmacokinetic Endpoint 17
2.2.8. Pharmacodynamics Endpoints 17
3. STUDY DESIGN 18
3.1. General Description 18
3.2. Randomization 19
3.3. Masking 19
3.4. Sample Size and Power Considerations 19
3.5. Analysis Timing and Unmasking 20
3.6. Definition of 12-Month Data in the Month 12 Dataset 21
4. STATISTICAL ANALYSIS SETS 22
4.1. Screened Set 22

4.2. Intent-to-Treat Set 22
4.3. Modified Intent-to-Treat Set 22
4.4. Safety Set 22
4.5. Per-Protocol Sets 22
4.6. Pharmacokinetic Set 23
4.7. Pharmacodynamic Set....24
4.8. Genotyping Set 24
5. STUDY SUBJECTS....25
5.1. Disposition of Subjects 25
5.2. Demographic and Other Baseline Characteristics....26
5.3. Medical ocular and non-ocular History 28
5.4. Prior and Concomitant Medications....29
5.5. Concomitant Procedures 29
5.6. Exposure to Investigational Product....29
5.7. Protocol Deviations 31
6. EFFICACY ANALYSES 32
6.1. Estimands....32
6.2. Analyses of Primary Efficacy Endpoint 35
6.2.1. Type I Error Management....35
6.2.2. Main Analysis of Primary Efficacy Endpoint 35
6.2.3. Sensitivity Analyses of Primary Efficacy Endpoint 36
6.2.3.1. Missing Data Analyses Based on Multiple Imputation....36
6.2.3.2. Other Sensitivity Analyses 38
6.2.4. Supplemental Analyses of the Primary Efficacy Endpoint....38
6.2.4.1. COVID-19 Adjusted Estimand 38
6.2.4.2. Per Protocol Set....39
6.2.4.3. Rate of Change Analyses....39
6.2.5. Subgroup Analyses of Primary Efficacy Endpoint....40
6.2.6. Full 24 Month Analyses 41
6.2.7. Exploratory Endpoints Closely Related to the Primary Efficacy Endpoint....41
6.2.7.1. Change in Square Root of GA Area....42
6.2.7.2. Percent Change in GA Area and Square Root of GA Area....42
6.3. Analyses of Key Secondary Efficacy Endpoints....42

6.3.1. Type I Error Management 42
6.3.2. Main Analyses of Key Secondary Efficacy Endpoints 46
6.3.2.1. Monocular Maximum Reading Speed 46
6.3.2.2. Mean FRI Index Score 46
6.3.2.3. NL-BCVA Score 47
6.3.3. Sensitivity Analyses of Key Secondary Efficacy Endpoints 48
6.3.4. Supplementary Analyses of Key Secondary Efficacy Endpoints 48
6.3.5. Subgroup Analyses of Key Secondary Efficacy Endpoints 48
6.4. Analyses of Other Secondary Efficacy Endpoints 49
6.4.1. Low Luminance Best-Corrected Visual Acuity (LL-BCVA) 49
6.4.2. Total Area of GA lesion(s) at Each Planned Assessment 50
6.4.3. Monocular Critical Print Size 50
6.4.4. NEI VFQ-25 Distance Activity Subscale Score 50
6.5. Multiplicity Adjustment 51
6.6. Analyses of Exploratory Endpoints 51
6.6.1. NEI VFQ-25 and NEI VFQ-39 Additional Analyses 51
6.6.2. Binocular Maximum Reading Speed and Critical Print Size 52
6.6.3. GA Lesion (s) Study Eye to Fellow Eye Comparisons 52
6.6.4. Digital Reading Index and Visual Function Application 52
6.6.5. Genetic Polymorphisms 53
6.6.6. Dry AMD Analysis 53
6.6.7. Analyses of Other Ophthalmology Efficacy Imaging Data 53
7. SAFETY ANALYSES 54
7.1. Adverse Events 54
7.1.1. TEAEs of New Onset Exudative AMD 55
7.2. Clinical Laboratory Data 56
7.3. Vital Signs 58
7.4. Physical Examination Findings 58
7.5. Complete Ophthalmic Exam 58
7.6. NL-BCVA 59
7.7. Other Ophthalmology Imaging Assessments 59
7.8. Immunogenicity 61
7.9. Death 61

7.10. Other Safety Data 61
8. PHARMACOKINETICS ANALYSIS 62
9. PHARMACODYNAMIC ANALYSES 63
10. OTHER ANALYSES 64
11. INTERIM ANALYSIS 65
12. DATA MONITORING COMMITTEE 66
13. DATA HANDLING CONVENTIONS 67
13.1. General Data Reporting Conventions 67
13.2. Definition of Baseline 67
13.3. Definition of Relative Study Days 67
13.4. Definition of Visit Windows 67
13.5. Derived Efficacy Endpoints 69
13.5.1. GA Lesion Size 69
13.5.2. MNRead Reading Chart 69
13.5.2.1. Reading Speed 69
13.5.2.2. Maximum reading speed 70
13.5.2.3. Critical Print Size 70
13.5.2.4. Reading acuity 70
13.5.3. Radner Reading Chart 70
13.5.3.1. Reading Speed 70
13.5.3.2. Maximum Reading Speed 71
13.5.3.3. Critical Print Size 71
13.5.3.4. Reading Acuity 71
13.5.4. Mean FRI Index Score and FRI Level 71
13.5.5. NL-BCVA and LL-BCVA 71
13.5.5.1. BCVA Letter Score 71
13.5.5.2. Conversion of BCVA Letter Score to Snellen Equivalent 72
13.5.6. Low Luminance Deficit 72
13.5.7. NEI VFQ-25 scores 72
13.5.7.1. NEI VFQ-25 Distance Activity Subscale Score 72
13.5.7.2. NEI VFQ-25 Near Activity Subscale Score 72
13.5.7.3. NEI VFQ-25 Driving Subscale Score 72
13.5.7.4. NEI VFQ-25 Composite Scores 72

13.5.8. NEI VFQ-39 Scores ... 73
13.5.8.1. NEI VFQ-39 Distance Activity Subscale Score ... 73
13.5.8.2. NEI VFQ-39 Near Activity Subscale Score ... 73
13.5.8.3. NEI VFQ-39 Composite score ... 73
13.6. Repeated or Unscheduled Assessments of Safety Parameters ... 73
13.7. Handling of Missing, Unused, and Spurious Data ... 73
13.7.1. Missing Date of Investigational Product ... 73
13.7.2. Missing Date Information for Prior or Concomitant Medications (Therapies/Procedures) ... 73
13.7.2.1. Incomplete Start Date ... 73
13.7.2.2. Incomplete Stop Date ... 74
13.7.3. Missing Date Information for Adverse Events ... 75
13.7.3.1. Incomplete Start Date ... 75
13.7.3.2. Incomplete Stop Date ... 75
13.7.4. Missing Severity Assessment for Adverse Events ... 75
13.7.5. Missing Relationship to Investigational Product for Adverse Events ... 75
13.7.6. Character Values of Clinical Laboratory Variables ... 76
14. ANALYSIS SOFTWARE ... 77
15. CHANGES TO ANALYSIS SPECIFIED IN PROTOCOL ... 78
16. REFERENCES ... 79
17. APPENDIX ... 80
APPENDIX 1. SCHEDULE OF ACTIVITIES ... 81
1.1 Protocol Changes to be followed during COVID-19 Restrictions ... 95
1.2 Sample of SAS Code ... 110
1.2.1. Mixed Effect Model for Repeated Measure ... 110
1.2.2. Multiple Imputation ... 111
1.2.3. Rate of Change Models ... 114
1.2.4. Sample Code for Maximum Reading Speed and Critical Print Size ... 117

LIST OF TABLES

Table 1: Power to Detect a Difference among Three Groups with an Equal Size of 200 Subjects 20

Table 2: Estimands of Study 34

Table 3: Criteria for Potentially Clinically Significant Laboratory Tests 57

Table 4: Criteria for Potentially Clinically Significant Vital Signs 58

Table 5: Post-Baseline Analysis Visit Window for Unscheduled and Early Termination Visits: Monthly Regimen 68

Table 6: Post-Baseline Analysis Visit Window for Unscheduled and Early Termination Visits: Every Other Month Regimen 69

Table 7: Examples for Coding of Special Character Values for Clinical Laboratory Variables 76

Table 8: Visit Schedule - Monthly Group- Screening, Day 1 through Month 12 82

Table 9: Visit Schedule - Monthly Group- Month 13 to Month 24 86

Table 10: Visit Schedule - Every-Other-Month Group Screening, Day 1 Through Month 12 89

Table 11: Visit Schedule - Every-Other-Month Group Month 13 to Month 24 92

Table 12 (TRACKED): COVID-19 VISIT SCHEDULE—Monthly Group—Screening, Day 1 Through Month 12 98

Table 13 (TRACKED): COVID-19 VISIT SCHEDULE—Monthly Group—Month 13 to Month 24 101

Table 14 (TRACKED): COVID-19 VISIT SCHEDULE—Every-Other-Month Group—Screening, Day 1 Through Month 12 104

Table 15 (TRACKED): COVID-19 VISIT SCHEDULE—Every-Other-Month Group—Month 13 to Month 24 107

LIST OF FIGURES

Figure 1: Study Schema 18

Figure 2: APL2-303 and APL2-304 Hypothesis Testing and Type I Error Control 44

Figure 3: APL2-303 Hypothesis Testing and Type I Error Control If There is No α Remaining in APL2-304 to Allow Pooling Secondary Functional Endpoints 45

ABBREVIATIONS

ADA	Anti-drug Antibodies
ADY	Analysis Study Day
AE	Adverse Event
ALP	Alkaline Phosphatase
ALT	Alanine Aminotransferase
AMD	Age-related Macular Degeneration
AST	Aspartate Aminotransferase
ATC	Anatomical Therapeutic Class
BCVA/NL-BCVA	(Normal luminance) Best Corrected Visual Acuity
BLQ	Below the Limit of Quantification
BUN	Blood Urea Nitrogen
C3	Complement component 3
C_{max}	Maximum Observed Concentration
CMH	Cochran Mantel Haenszel
CNV	Choroidal Neovascularization
CRO	Contract Research Organization
cRORA	Complete retinal pigment epithelium and outer retinal atrophy
CSR	Clinical Study Report
DA	Disk Areas
DCFP	Digital Color Fundus Photography
DLS	Double-layer sign
DMC	Data Monitoring Committee
eCRF	Electronic Case Report Form
EOM	Every Other Month
ETDRS	Early Treatment Diabetic Retinopathy Study
FAF	Fundus Autofluorescence
FDA	Food and Drug Administration
FFA/FA	Fundus Fluorescein Angiography or Fluorescein Angiography
FRI	Functional Reading Independence
GA	Geographic Atrophy
IOP	Intra Ocular Pressure
iRORA	Incomplete retinal pigment epithelium and outer retinal atrophy
ITT	Intent-to-Treat
IVT	Intravitreal
kg	Kilogram
LOV	Last Observed Value
LL-BCVA	Low Luminance Best Corrected Visual Acuity
LLD	Low Luminance Deficit
LS	Least Square
MAR	Missing at Random
MCMC	Markov Chain Monte Carlo
MedDRA	Medical Dictionary for Regulatory Activities
mg	Milligram
mITT	Modified Intention to Treat
mL	Milliliter

mmHg	Millimeter of Mercury
MMRM	Mixed effect Model for Repeated Measure
MNAR	Missing Not At Random
MNRead	Minnesota Reading Chart
NEI VFQ-25	National Eye Institute Visual Functioning Questionnaire 25 Item Version
NEI VFQ-39	National Eye Institute Visual Functioning Questionnaire 39 Item Version
NIR	Near Infrared Reflectance
OCT	Optical Coherence Tomography
OCT-A	Optical Coherence Tomography Angiography
PCS	Potentially Clinically Significant
PD	Pharmacodynamic
PDV	Protocol Deviation
PEG	Polyethylene glycol
PEOM	Pegcetacoplan Every-Other-Month
PK	Pharmacokinetics
PM	Pegcetacoplan Monthly
PP	Per Protocol
PT	Preferred Term
RBC	Red Blood Cell
SAE	Serious Adverse Event
SAP	Statistical Analysis Plan
SAS	Statistical Analysis System
SD-OCT	Spectral Domain Optical Coherence Tomography
SEOM	Sham Every-Other-Month
SI	International System of Units
SM	Sham Monthly
SNP	Single Nucleotide Polymorphism
SOC	System Organ Class
TEAE	Treatment Emergent Adverse Event
t_{max}	Time to Maximum Measured Concentration
µL	Micro liter
WBC	White Blood Cell
WHO	World Health Organization
WPM	Words per Minute

1. INTRODUCTION

This study is being conducted as part of a series of studies for the clinical development of pegcetacoplan (also known as APL-2) for geographic atrophy [GA] secondary to age-related macular degeneration (AMD). This statistical analysis plan (SAP) provides a technical and detailed elaboration of the statistical analyses of efficacy, safety, and pharmacokinetic data, supplementing what is described in the final study protocol, version 1.0, amendment 5, dated 12 August 2020.

The primary study analysis will be based on complete data from the first 12 months of the study and performed after all subjects have completed the Month 12 visit in the study or discontinued early, and all corresponding data have been entered into the database, reviewed, cleaned, and finalized as the Month 12 dataset. Analysis will include testing of hypotheses of the primary efficacy endpoint at Month 12. Key secondary, secondary, and exploratory endpoints will be evaluated based on data in the Month 12 datasets in a descriptive manner. An analysis of the safety data in the Month 12 dataset will also be performed.

The final study analysis will be based on data after all subjects have either completed the Month 24 visit in the study or discontinued the study early, and all data from the study are in the database and the database is reviewed, cleaned, and locked as the final dataset. Analysis will include testing of hypotheses of the key secondary endpoints at Month 24 as described in Section 6.3.1. A cumulative analysis of all data through the end of the study will be produced based on the final dataset.

See Section 3.5 for further details on analysis timing.

The analyses specified in this document supersede the analysis plan described in the study protocol (protocol version 1.0, amendment 5 dated 12 August 2020).

2. OBJECTIVES AND ENDPOINTS

2.1. Objectives

2.1.1. Primary Objective

The primary objective of this study is to evaluate the efficacy of pegcetacoplan compared to sham in subjects with GA secondary to AMD assessed by change in the total area of GA lesions in the study eye from baseline as measured by Fundus Autofluorescence (FAF).

2.1.2. Key Secondary Objectives

The key secondary objectives are to evaluate the efficacy of pegcetacoplan compared to sham in subjects with GA secondary to AMD with respect to:

- Monocular maximum reading speed (study eye), as assessed by Minnesota Reading (MNRead) or Radner Reading Charts (in select countries)
- Functional Reading Independence (FRI) Index score (subject-level assessment)
- Normal luminance best-corrected visual acuity (NL-BCVA) score in the study eye

2.1.3. Secondary Objectives

To evaluate the efficacy of pegcetacoplan compared to sham in subjects with GA secondary to AMD with respect to:

- Low luminance best corrected visual acuity (LL-BCVA) score in the study eye
- Low luminance deficit (LLD) in the study eye
- Total area of GA lesion(s) in the study eye
- Monocular critical print size (study eye), as assessed by MNRead or Radner Reading Charts (in select countries)
- National Eye Institute Visual Functioning Questionnaire 25 Item Version (NEI VFQ-25) distance activity subscale score (in select countries)

To evaluate the pharmacokinetics (PK) of pegcetacoplan as assessed by systemic serum concentration of pegcetacoplan (in select sites).

2.1.4. Safety Objectives

To evaluate the safety and tolerability of pegcetacoplan compared to sham injection in subjects with GA secondary to AMD as indicated by:

- Incidence and severity of ocular and systemic treatment-emergent adverse events (TEAEs)
- Incidence of anti-drug antibodies (ADA) directed against pegcetacoplan peptide or polyethylene glycol (PEG)
- Incidence of new active choroidal neovascularization (CNV) in the study eye

2.1.5. Exploratory Objectives

- To evaluate the efficacy of pegcetacoplan compared to sham in subjects with GA secondary to AMD as indicated by:
  - NEI VFQ-25 composite score
  - NEI VFQ-25 near activity subscale score (in select countries)
  - Comparison between study eye and fellow eye of change in GA lesion size
  - To evaluate the binocular reading speed as assessed by MNRead or Radner Reading Charts (in select countries)
  - To evaluate the binocular critical print size as assessed by MNRead or Radner Reading Charts (in select countries)
  - To evaluate the relationship between genetic polymorphisms associated with AMD with GA progression and response to pegcetacoplan
- To evaluate the incidence of new onset of subclinical CNV in the study eye.
- To assess sensitivity and specificity of a digital reading speed application to detect disease progression / regression (optional, select sites)
- To assess sensitivity and specificity of a digital visual function application to detect disease progression / regression (optional, select sites)

2.2. Endpoints

2.2.1. Primary Endpoint

The primary endpoint is the change from baseline to Month 12 in total area of GA lesion(s) in the study eye (in mm^2) based on FAF.

2.2.2. Key Secondary Endpoints

The key secondary endpoints include:

- Change from baseline in uncorrected monocular maximum reading speed (study eye), at Month 24 as assessed by MNRead or Radner reading charts (in select countries).
- Change from baseline in mean FRI Index score (subject-level assessment) at Month 24
- Change from baseline in NL-BCVA score (study eye) at Month 24 as assessed by Early Treatment Diabetic Retinopathy Study (ETDRS) chart.

2.2.3. Secondary Endpoints

Other secondary endpoints include:

- Change from baseline in LL-BCVA score (study eye) over time as assessed by ETDRS chart.

- Change from baseline in the total area of GA lesion(s) in the study eye (in mm^2) as assessed by FAF over time other than Month 12.
- Change from baseline in monocular critical print size (study eye), as assessed by MNRead or Radner reading charts (in select countries) over time.
- Change from baseline in the NEI VFQ-25 distance activity subscale score (in select sites) (subject level assessment) over time.
- Systemic serum concentration of pegcetacoplan (selected sites) over time.

2.2.4. Exploratory Endpoints

- Additional NEI-VFQ endpoints including:
  - Change from baseline in the NEI VFQ-39 distance activity subscale score (in select sites) over time.
  - Change from baseline in NEI VFQ-25 composite score over time.
  - Change from baseline in NEI VFQ-39 composite score over time.
  - Change from baseline in NEI VFQ-25 near activity subscale score (in select countries) over time.
  - Change from baseline in NEI VFQ-39 near activity subscale score (in select countries) over time.
  - Change from baseline in NEI VFQ-25 driving subscale score (in select countries) over time as well as the number and percentage of subjects with worse driving outcomes for subjects who are currently driving at baseline.
- Difference between study eye and fellow eye in change in GA lesion size from baseline over time in subjects with bilateral GA.
- Change from baseline in binocular maximum reading speed as assessed by MNRead or Radner Reading Charts (in select countries) over time.
- Change from baseline in binocular critical print size as assessed by MNRead or Radner Reading Charts (in select countries) over time.
- Change from baseline in digital reading index (in select sites) over time as measured by the Spotlight instrument.
- Change from baseline in digital visual function (metamorphopsia) (in select sites) over time as measured by the Alleye instrument.
- Progression from incomplete retinal pigment epithelium and outer retinal atrophy (iRORA) to complete retinal pigment epithelium and outer retinal atrophy (cRORA).
- Progression from large drusen to iRORA or cRORA.
- Other efficacy imaging endpoints including change in the distance of the atrophy junction to the fovea, and non-subfoveal atrophy to subfoveal atrophy conversion.

2.2.5. Safety Endpoints

- Incidence and severity of ocular and systemic treatment-emergent adverse events.
- Incidence of ADA directed against pegcetacoplan peptide or PEG.
- Incidence of new active CNV in the study eye.
- Incidence of new onset of subclinical CNV in the study eye.
- Incidence of subjects who lost letters based on NL-BCVA categories (≥15, ≥15 - <30, ≥30 ETDRS letters).
- Change/shift from baseline in clinical labs and incidence of abnormal lab values.
- Change from baseline in vital signs and incidence of abnormal vital sign results.
- Shift from baseline in ocular examination assessments including slit-lamp examination and indirect ophthalmoscopy.
- Change from baseline in Intra Ocular Pressure (IOP) and incidence of IOP above specified thresholds.
- Change/shift from baseline in ocular imaging assessments (including specular microscopy; select sites).

2.2.6. Exploratory Endpoints Closely Related to the Primary Efficacy Endpoint

- Change from baseline in square root of the total area of GA lesion(s) in the study eye (in mm) based on FAF over time.
- Percent change in total area of GA lesion(s) in the study eye (in mm^2) based on FAF over time.
- Percent change in square root of the total area of GA lesion(s) in the study eye (in mm) based on FAF over time.

2.2.7. Pharmacokinetic Endpoint

Serum pegcetacoplan concentrations will be determined from samples taken during the course of the study. Maximum serum concentration (C_{max}) will be determined from serum concentration data at Day 7 following pegcetacoplan treatment ($C_{Day\ 7}$).

2.2.8. Pharmacodynamics Endpoints

Change from baseline in serum complement profile (C3, CH50, AH50).

3. STUDY DESIGN

3.1. General Description

This is a Phase 3, multicenter, randomized, double-masked, sham-injection controlled study to assess the efficacy and safety of multiple intravitreal (IVT) injections of pegcetacoplan (also known as APL-2) in subjects with GA secondary to AMD.

The study will randomize approximately 600 subjects across approximately 100 multinational sites. Subjects will be screened within 28 days before receiving pegcetacoplan or Sham injection. Upon providing written informed consent and entry into the study, subjects will be assigned a screening number. Subjects who meet all inclusion and none of the exclusion criteria will return to the clinic for randomization and treatment on Visit 2 (Day 1). At this visit, subjects will be randomized 2:2:1:1 to receive pegcetacoplan Monthly (PM), pegcetacoplan Every-Other-Month (PEOM), Sham injection Monthly (SM) or Sham-injection Every-Other-Month (SEOM), respectively. Randomization will be stratified according to GA lesion area at screening ($< 7.5\ mm^2$; $\geq 7.5\ mm^2$), and presence of CNV in the fellow eye.

The planned length of participation in the study for each subject is approximately 24 months (from the beginning of the screening period through Month 24 visit). After the COVID-19 pandemic started, Apellis added a rescreening procedure where the screening period was extended to up to 90 days (applicable only to subjects that fully qualified for the trial during a complete screening prior to March 30, 2020). The total length of participation in this study can be up to 27 months for subjects that were screened using this updated procedure. The schedules of assessments are presented in Appendices A, B, C, and D of Study Protocol Amendment 5, Version 1.0 dated 12 August 2020 and are also available in Appendix 1.

A study schematic diagram is shown in Figure 1.

Figure 1: Study Schema

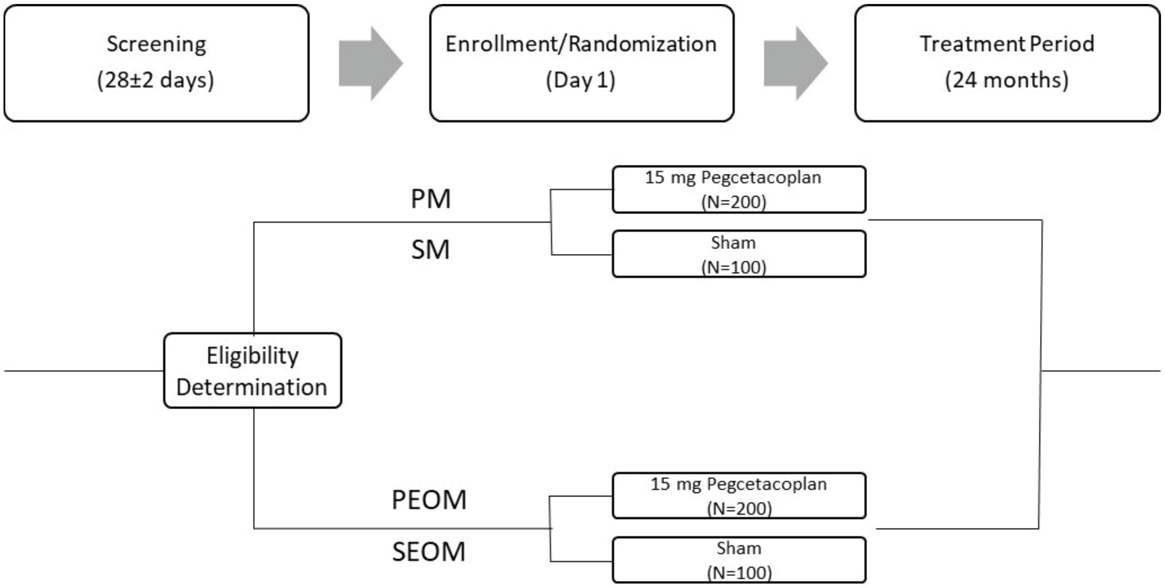

PM= Pegcetacoplan Monthly; SM= Sham Monthly; PEOM= Pegcetacoplan Every-Other-Month; SEOM= Sham Every-Other-Month

3.2. Randomization

Subjects will be randomized 2:2:1:1 using a web-based randomization system to receive treatment with PM, PEOM, SM or SEOM, respectively.

The randomization scheme will be maintained by the Sponsor, or designee. Subject randomization will be stratified by GA lesion area at screening based on assessment from the reading center (< 7.5 mm^2; ≥ 7.5 mm^2) and presence of CNV in the fellow eye (yes; no).

3.3. Masking

This is a double-masked study. Designated masked study site staff (e.g., assistant(s), VA technicians, optical coherence tomography (OCT) technicians, photographers, technicians administering questionnaires, subjects, Reading Center personnel, the assigned evaluating physician(s), and the Sponsor) will be masked to treatment assignment. However, the treating physician and any associated support staff involved in performing the intravitreal or sham injections will be unmasked to study treatment. These individuals are only responsible for administering the study drug and are not involved in assessing adverse events. In addition, the unmasked individuals are not allowed to discuss treatment and/or subject outcomes with masked study staff, including the evaluating physician. The Principal Investigator must be masked to subjects' treatment assignment. To prevent bias in treatment assignment, eligible subjects will be randomized using a web-based randomization system. Processes and plans will be put in place to avoid unintentional unmasking during the study. All study roles will be clearly documented on the site delegation of authority log and once the roles have been designated and executed, these roles should not be switched during the conduct of the study. In unforeseen circumstances, a site can contact the Sponsor to switch a study staff member from the masked role to the unmasked role but not vice versa. Details on unmasking at the Month 12 reporting are in Section 3.5.

While the actual study treatment (pegcetacoplan vs. sham) is masked, the treatment frequency (monthly vs. every other month) for each individual subject is known.

3.4. Sample Size and Power Considerations

A total of approximately 600 subjects will be randomized in a 2:2:1:1 ratio to receive treatment with PM, PEOM, SM, or SEOM. The annual growth rate in GA lesion area is expected to have a mean of 1.47, 1.70 and 2.13 mm^2/year for PM, PEOM and Sham-Pooled (Sham) groups, respectively, as estimated from the results of a Phase 2 trial of pegcetacoplan. The standard deviation of the lesion growth is estimated to be 1.50 mm^2 based on the same Phase 2 trial data or 1.25 mm^2 based on natural history data (Holekamp N, 2019).

The power of the study for the primary endpoint is presented in Table 1 for sample size of 200 in each treatment arm. The approximation is calculated using PROC POWER ONEWAYANOVA, Statistical Analysis System (SAS) 9.4. The study power for the primary endpoint is likely larger when utilizing the longitudinal data to model the primary endpoint. The actual study power for the primary endpoint may also vary based on the distribution of the stratification factors (i.e., lesion area at screening, presence of CNV in fellow eye). Study power was not calculated for key secondary functional endpoints.

Table 1: Power to Detect a Difference among Three Groups with an Equal Size of 200 Subjects

Common Standard Deviation (mm^2)	Alpha (two-sided)	Power for a true mean of 1.47, 1.70, and 2.13 mm^2/year for PM, PEOM and Sham, respectively		
		PM vs Sham	PEOM vs Sham	Overall (Among 3 groups)
1.25	0.0495	> 99.9%	92.9%	99.9%
1.25	0.0248	99.9%	88.2%	99.7%
1.40	0.0495	99.7%	86.5%	99.3%
1.40	0.0248	99.3%	79.4%	98.6%
1.50	0.0495	99.2%	81.5%	98.4%
1.50	0.0248	98.4%	73.1%	97.0%

PM = pegcetacoplan Monthly, PEOM = pegcetacoplan Every-Other-Month; Sham = Sham Monthly + Sham Every-Other-Month

3.5. Analysis Timing and Unmasking

The analysis of data from the first 12 months of the study will be performed when all subjects have completed the Month 12 visit in the study or discontinued early and all corresponding data have been entered into the database, reviewed, cleaned, and finalized as the Month 12 dataset per the 12-Month Primary Analysis Data Cut Plan and the Programming Plan. At the time of the analysis of the Month 12 dataset, subjects who have not completed or discontinued from the study will still be followed by the sites as part of the second year.

At the time of the Month 12 reporting, Sponsor personnel who are analyzing, interpreting, and reporting data from the Month 12 dataset will be unmasked to treatment assignment. To maintain data integrity for the remainder of the study, sponsor and contract research organization (CRO) personnel responsible for continuing study oversight will remain masked as documented in the Study Masking Plan and the GA Internal Unmasking Plan.

Analyses will include formal hypothesis testing of the primary efficacy endpoint at Month 12. Key secondary, secondary, and exploratory endpoints will be evaluated based on the Month 12 dataset in a descriptive manner. An analysis of the safety data in the Month 12 dataset will also be performed.

The final study reporting will be based on data after all subjects have either completed the Month 24 visit in the study or discontinued the study early, and all data from the study are entered in the database and the database is reviewed, cleaned, and locked as the final dataset. Minor changes are expected from the Month 12 dataset (e.g. adverse event end dates for events ongoing in the Month 12 dataset), however, all subsequent differences in data prior to the Month 12 visit will be listed. Analysis of the final dataset will include testing of hypotheses of the key secondary endpoints at Month 24 as described in Section 6.3.1. A cumulative analysis of all data through the end of the study will be produced based on the final dataset.

Aggregate results of the Month 12 reporting may be reported before completion of the study. However, subjects, masked study site personnel, masked CRO personnel at Covance, Sponsor team members with study oversight responsibility, and central reading center personnel will remain masked to individual treatment assignments until after the study is completed

(after all subjects have either completed Month 24 or discontinued early from the study), the database is locked and the unmasking has been approved.

3.6. Definition of 12-Month Data in the Month 12 Dataset

All screening and post-baseline data with a clinical date (i.e., administration/assessment/onset/start date) on or before the defined Month 12 data cutoff date will be included in the Month 12 Dataset. The 12-month data cutoff will include all data regardless of the type of study visit at which it was collected. This may include data collected at unscheduled visits or early termination visits if the visit date was on or before the 12-month data cutoff date. The Month 12 data cutoff is defined as:

- If the subject completed Month 12 (Visit 14) assessments, then the data cutoff will be the day that the Month 12 (Visit 14) assessments are completed, regardless of Study Day.
- If the subject did not complete the Month 12 (Visit 14) assessments (e.g. missed or early terminated) then Study Day 375 (Study Day 360 (target date) + 15) will be used as the Month 12 cutoff.

4. STATISTICAL ANALYSIS SETS

Two sham treatment arms (SM and SEOM) will be pooled into a single control (Sham) group for analyses.

4.1. Screened Set

The screened set consists of all subjects who provided written informed consent and are screened for participation in this study. This population will only be used for the purposes of describing the subject disposition and for listing the data.

4.2. Intent-to-Treat Set

The Intent-to-treat (ITT) set consists of all randomized subjects. Subjects will be analyzed in the treatment arm assigned at randomization.

4.3. Modified Intent-to-Treat Set

The modified ITT (mITT) set consists of all randomized subjects who receive at least one injection of pegcetacoplan or sham and have baseline and at least one post-baseline value of GA lesion area in the study eye as assessed by FAF. Subjects will be analyzed in the treatment arm assigned at randomization.

4.4. Safety Set

The Safety set consists of all subjects randomized who receive at least one injection of pegcetacoplan or sham. Subjects will be analyzed according to the actual treatment received. In the case a subject received an incorrect injection of study medication than what they were randomized to, subjects will be presented under the corresponding pegcetacoplan arm if they received at least one injection of pegcetacoplan during the study and will only be presented under the corresponding sham arm if they did not receive any injections of pegcetacoplan. This population will be used for all safety analyses.

4.5. Per-Protocol Sets

The Per-Protocol (PP) sets will be identified separately for Month 12 and Month 24 analysis, respectively (i.e., Month 12 PP set and Month 24 PP set). The PP sets consist of all mITT subjects who have a valid GA lesion area assessment for either Month 10 or 12 (Month 12 PP set) or a valid GA lesion area assessment for at least one of Month 18, 20, 22, 24 (Month 24 PP set) and who follow the protocol without any major deviation(s) that could affect the primary efficacy data.

A valid GA lesion area assessment is defined as a non-missing measured GA lesion area assessment at a given timepoint where at least 75% of the expected injections over the course of participation ahead of the given timepoint have been received by the subject. For example:

- A valid GA lesion measurement at Month 10 in the Monthly arm would be a result available at Month 10 with no more than 2 missed injections prior to the Month 10 assessment (10 scheduled before Month 10, 2 missed = 80% compliance).

- A valid GA lesion measurement at Month 20 in the every other month (EOM) arm would be a result available at Month 20 with no more than 2 missing injections prior to the Month 20 assessment (10 scheduled before Month 20, 2 missed = 80% compliance).

Major protocol deviations (PDVs) that lead to exclusion from the Month 12 PP set and the Month 24 PP set are as follows:

(i) Violations of inclusion and/or exclusion criteria

A PDV with any of the following inclusion and/or exclusion criteria violations will be classified as a major PDV:

Inclusion Criterion #3: Clinical diagnosis of GA of the macula secondary to AMD as determined by the Investigator and confirmed by the Reading Center

Inclusion Criterion #4: The GA lesion must meet the criteria listed in the protocol as determined by the central Reading Center's assessment of FAF

- Total GA area must be ≥ 2.5 and ≤ 17.5 mm^2 (1 and 7 disk areas [DA] respectively).
- If GA is multifocal, at least one focal lesion must be ≥ 1.25 mm^2 (0.5 DA), with the overall aggregate area of GA as specified above in 4a.
- The entire GA lesion must be completely visualized on the macula centered image and must be able to be imaged in its entirety and not contiguous with any areas of peripapillary atrophy.
- Presence of any pattern of hyperautofluorescence in the junctional zone of GA. Absence of hyperautofluorescence (i.e., pattern = none) is exclusionary.

Exclusion Criterion #1: GA secondary to a condition other than AMD such as Stargardt disease, cone rod dystrophy or toxic maculopathies like plaquenil maculopathy in either eye.

Exclusion Criterion #3: Any history or active choroidal neovascularization (CNV), associated with AMD or any other cause, including any evidence of retinal pigment epithelium rips or evidence of neovascularization anywhere based on spectral domain optical coherence tomography (SD-OCT) imaging and/or fluorescein angiography as assessed by the Reading Center.

Exclusion Criterion #13: Prior participation in another interventional clinical study for geographic atrophy in either eye including investigational oral medication and placebo.

(ii) Not receiving assigned treatment

Not receiving assigned treatment at more than 25% of the treatment visits over the analysis timeframe, either 12-month or 24-month, will be classified as a major PDV.

4.6. Pharmacokinetic Set

The PK set will include all subjects in the safety set who have at least one quantifiable post-dose concentration of pegcetacoplan (even with values below the limit of quantification (BLQ)).

4.7. Pharmacodynamic Set

The pharmacodynamic (PD) set will include all subjects in the safety set who have at least one quantifiable post-dose PD endpoint (C3, CH50, or AH50) evaluated.

4.8. Genotyping Set

The genotyping set consists of all ITT subjects who have at least one non-missing genotyping result for a single nucleotide polymorphism (SNP) associated with age-related macular degeneration from the genotype sequencing analysis.

5. STUDY SUBJECTS

5.1. Disposition of Subjects

The number of subjects screened, passed screening, screened failed, and the reasons for screen failure will be presented overall.

The number of subjects randomized and in each defined analysis set will be summarized by treatment group and overall for the ITT Set. In addition, the reasons for exclusion from the mITT and per-protocol populations will be summarized.

Subjects' disposition by region and country will also be provided for the ITT set. All summaries on subject disposition will be produced based on the Month 12 dataset for the Month 12 reporting and based on the final study dataset for the final reporting, as appropriate.

Overall summary of subjects' disposition includes:

- Number of subjects randomized
- Number of subjects completed treatment [through Month 12 or Month 24, as appropriate]
- Number of subjects discontinued from treatment and reason for discontinuation [prior to Month 12 or Month 24 as appropriate]
    - Subjects who discontinued from treatment without concurrent study discontinuation will be singled out [prior to Month 12 or Month 24 as appropriate]
- Number of subjects completed study [through Month 12 or Month 24, as appropriate]
- Number of subjects discontinued from study and reason for discontinuation [prior to Month 12 or Month 24 as appropriate]

A summary of the disposition through each study visit will also be presented by treatment group and overall for the ITT set.

In addition, to assess the impact of COVID-19 pandemic on disposition of subjects, the following summary will be provided for each study visit and by reason (COVID-19 vs. non-COVID-19) for the ITT set:

- Number of subjects discontinued from treatment
- Number of subjects discontinued from study

5.2. Demographic and Other Baseline Characteristics

Demographic and baseline characteristics will be tabulated using descriptive statistics by treatment group and overall. Tables will be produced for the following analysis sets: ITT, Safety, mITT, Month 12 PP and Month 24 PP (as appropriate), PK, PD, and Genotyping. The following variables will be included in the tables:

The demographic data are:

- Age at screening (years) and in categories
  - < 65
  - 65-<75
  - 75-<85
  - ≥ 85
- Sex
- Race
- Ethnicity
- Geographic Region (United States vs. Rest of World)
- Country
- Weight (kg) at baseline
- Height (cm) at baseline
- Body Mass Index (kg/m^2)
- Tobacco use status (ever, never)

Baseline characteristics will be summarized for both study eye and fellow eye whenever applicable. Baseline characteristics include:

- Study eye laterality (OD, OS)
- Study eye status (better-seeing eye vs. worse-seeing eye, based on NL-BCVA – in the case of ties, study eye status will be classified as the better-seeing eye)
- GA lesion size (mm^2) (assessed by FAF)
- GA lesion size in categories, determined by reading center (as randomized status and actual status):
  - < 7.5 mm^2
  - ≥ 7.5 mm^2
- GA lesion size categories (approximately tertiles based on APL2-303/304 combined data*) – study eye only
- Square root GA lesion size (mm)

- GA lesion location (subfoveal involvement vs. non-subfoveal involvement) (assessed by FAF)
- GA focality (unifocal vs. multifocal) (assessed by FAF)
- Number of areas of atrophy (none, 1, 2-5, 6-10, 11-20, >20, cannot be determined) (assessed from FAF)
- Presence of double-layer sign (DLS) (assessed by SD-OCT)
- Central subfield thickness (CST) (assessed by SD-OCT)
- Presence of macular neovascularization (MNV) (i.e., subclinical CNV) (assessed by Optical Coherence Tomography Angiography (OCT-A))
- Presence of pseudodrusen (assessed by near-infrared reflectance imaging)
- Number of intermediate/large drusen (0-5, 6-10, 11-20, and >20) (assessed by color fundus photography)
- NL-BCVA in ETDRS letters
- NL-BCVA categories (≥70, ≥60 - <70, ≥35 - <60, <35 ETDRS letters)
- NL-BCVA (<60 vs. ≥60 ETDRS letters)
- LL-BCVA in ETDRS letters
- LL-BCVA categories (approximately tertiles based on APL2-303/304 combined data*) – study eye only
- IOP
- LLD in ETDRS letters
- LLD categories (<20 vs. ≥ 20 ETDRS letters)
- LLD categories (approximately tertiles based on the APL2-303/304 combined data*) – study eye only
- NL-BCVA (<60 vs. ≥60 ETDRS letters) and LLD (<20 vs. ≥ 20 ETDRS letters) combinations
- Advanced AMD (fellow eye with: GA only, GA with CNV, CNV only, neither GA nor CNV)
- GA laterality (bilateral GA vs. study eye GA Only)
- Presence of CNV in fellow eye (as randomized and actual status (based on medical history))
- Lens status:
    - Phakic
    - Pseudophakic
- Reading speed assessment method

- Monocular and binocular maximum reading speed and critical print size (without adjustment for reading inaccuracy)
- Monocular maximum reading speed categories (<60, 60-<160, ≥160 words per minute (wpm))
- Monocular maximum reading speed categories (approximately tertiles based on APL2-303/304 combined data*) – study eye only
- Mean FRI Index score
- FRI Level (1,2,3,4)
- NEI VFQ-25 distance activity subscale score
- NEI VFQ-25 near activity subscale score
- NEI VFQ-25 driving subscale score
- Number of subjects currently driving at baseline
- NEI VFQ-25 composite score
- C3 Complement level
- C3 Complement level categories (tertiles of C3)
- Dry AMD status (iRORA, large drusen) (at Month 24 reporting only)

*Approximately tertiles will be specified consistently across the APL2-303/304 studies to the nearest clinically relevant level.

Baseline will be defined as the last available pre-treatment value taken on or before the first dose date of study drug. In the case a subject did not receive study drug in the ITT population, the last assessment prior to study exit will be used as baseline.

Conversions for height and BMI are as follows:

- Height (m) = Height (cm) x 0.01
- Body mass index (kg/m^2) = Weight (kg) / [Height (m)]^2

Demographics and baseline characteristics will also be listed for the ITT set.

5.3. Medical ocular and non-ocular History

Medical history will be coded using the Medical Dictionary for Regulatory Activities (MedDRA) dictionary version 23.1. Summaries will be presented by System Organ Class (SOC) and Preferred Term (PT) with counts and percentages by treatment group and overall. Each subject will be counted only once in each SOC or SOC/PT summary.

All ocular history will be summarized by SOC and PT with counts and percentages by treatment group and overall. The summaries will be produced separately for the study eye and the fellow eye. Any ocular condition associated with both eyes will be included in both summaries. Data listings of medical history and ocular history will be provided for the ITT set.

5.4. Prior and Concomitant Medications

Prior and concomitant medications will be coded using World Health Organization (WHO) Drug Dictionary version WHO Drug Global B3-format March 2021. Non-ocular medications will be presented for the ITT set by anatomical therapeutic class (ATC) level 2 (therapeutic main group) and preferred term with counts and percentages by treatment group and overall. A subject who took more than one medication will be counted only once if these medications belong to the same extended ATC classification. In addition, prior and concomitant ocular medications used in study eye will be summarized. Similar summaries will be provided for the ocular medications used in the fellow eye. Any ophthalmological medications (ATC2 = 'Ophthalmologicals') that are not assigned to an eye will be presented under both eyes in the ocular medications tables and will not be presented in the non-ocular medications tables.

Prior medications will be defined as those medications taken prior to the administration of study drug on Day 1. Concomitant medications will be defined as those medications taken following the first administration of study drug on Day 1. Hence medications started before study dosing and continuing into the treatment period are considered as both prior and concomitant medications.

Separate tables will be provided for prior and concomitant medications, respectively. A data listing of prior and concomitant medications, for both ocular and non-ocular medications will be provided for the ITT set.

5.5. Concomitant Procedures

Concomitant procedures will be coded using MedDRA version 23.1. Non-ocular procedures will be presented for the ITT set by preferred term with counts and percentages by treatment group and overall. A subject who had more than one procedure will be counted only once in the summary. In addition, concomitant ocular procedures in study eye will be summarized. Similar summaries will be provided for the ocular procedures used in the fellow eye.

Concomitant procedures will be defined as those procedures taken following the first administration of study drug on Day 1.

A data listing of all procedures, for both non-ocular and ocular procedures will be provided for the ITT set.

5.6. Exposure to Investigational Product

Exposure to the investigational product will be summarized for the ITT, mITT, and Safety sets. All analyses will be repeated for the first 12 months at both the Month 12 and final reporting as well as the full 24 months for the final reporting.

For the analysis of exposure data for the first 12 months, exposure data prior to the Month 12 visit will be summarized (i.e., the Month 12 injection will not be included in the summaries). For analyses of the full 24 months, all data will be summarized.

Injections in the fellow eye will be considered in the calculation of the duration of treatment but will otherwise be considered as a missed injection in the study eye for all other measures of exposure.

The total number of injections and total number of missed injections received in each treatment group will be summarized by treatment group to support per-injection based TEAE rates.

The following categories for reason for missed injections will be summarized by treatment group using frequency counts and percentages based on the total number of missed injections: COVID-19 (with a breakdown of COVID-19 reasons as collected in the electronic case report form (eCRF)) vs. non-COVID-19 reasons (any reasons other than eCRF documented COVID-19 reasons). The total number of injections per subject will be summarized by treatment group both as a continuous summary and as a frequency and percent summary. In addition, the following categories of the total number of injections per subject will be summarized by treatment group using frequency counts and percentages:

- First 12 months summaries
  - Monthly: 1-3, 4-6, 7-9, 10-12
  - EOM: 1-2, 3-5, 5-6
- Full 24 months summaries
  - Monthly: 1-3, 4-6, 7-9, 10-12, 13-15, 16-18, 19-21, 22-24
  - EOM: 1-2, 3-5, 5-6, 7-8, 9-10, 11-12

The number of subjects who received at least 75% of the planned injections for the study period (first 12 months or full 24 months) will be presented. For the first 12 months, subjects will be considered to have received 75% of the planned injections if they received at least 9 (out of 12) injections for the Monthly group or 5 at least (out of 6) injections for the EOM group. For the full 24 months, subjects will be considered to have received 75% of the planned injections if they received at least 18 (out of 24) injections for the Monthly group or at least 9 (out of 12) injections for the EOM group.

The number of subjects missing at least one injection will be summarized by treatment group. The number of missed injections per subject as well as well as a breakdown of the number of missed injections per subject due to COVID-19 reasons and due to non-COVID-19 reasons will be summarized by frequency and counts. Additionally, for the missed injections due to COVID-19, the subjects will be summarized by their maximum drug holiday in the following categories:

- No Consecutive Injections (Subjects who are missing an injection due to COVID-19 but not at 2 injection visits in a row)
- 2 Consecutive Injections
- 3 Consecutive Injections
- 4 or More Consecutive Injections

The number of missed injections is defined as the scheduled injections missed up to completion or discontinuation of study treatment.

Additionally, the duration of treatment and compliance will be summarized by treatment group.

Duration of treatment will be defined as

- Monthly group: (date of last injection +30) – date of first injection +1
- EOM group: (date of last injection +60) – date of first injection +1

The duration of treatment will be truncated to a subject's early termination date, month 12 cutoff date, or study completion date as appropriate.

Compliance (%) is defined as the number of injections administered divided by the number of scheduled injections up to completion or discontinuation of study treatment × 100. Compliance will be summarized in the following groups: <75%, ≥75% to <100%, 100%, and >100%.

The number of subjects with scheduled IVT injection visits as well as the number of subjects receiving injections at each of the Months (1-24) will also be summarized by treatment group using frequency counts and percentages. For the EOM group, any injections given on the odd-numbered months will be presented as an unscheduled injection. In addition, to assess the impact of the COVID-19 pandemic on study drug exposure, the following summary will be provided by study visit:

- Number of missed IVT injection visits for study drug
- Number of missed IVT injection visits for study drug due to COVID-19 pandemic reasons
- Number of missed IVT injection visits for study drug due to non-COVID-19 pandemic reasons

Study drug administration will be listed by treatment group. In addition, a listing of total number of injections, duration of treatment, date and day of first and last dose and number of missed injections will be provided for the ITT set.

5.7. Protocol Deviations

Major and minor PDVs will be assessed by sponsor personnel following Protocol Deviation Management Plan.

A PDV is classified as major if there is the potential to significantly impact the completeness, accuracy, and/or reliability of the study data, or affect a subject's rights, safety, or well-being. All PDVs will be identified and finalized prior to the analysis of the first 12 months as well as the analysis of the full 24 month and documented.

In addition, a major PDV will be derived for any subjects who did not have a valid GA lesion area assessment for either Month 10 or 12 and who had not discontinued treatment prior to Month 12. The category for this deviation will be "Study Conduct/Procedures", the subcategory will be "Study Assessment", and the study specific category will be "No valid GA lesion area assessment for either Month 10 or Month 12".

PDVs will be presented for the ITT set by deviation category, subcategory, and study specific category with counts and percentages by treatment group and overall. In addition, similar summaries will be provided for major PDVs as well as for PDVs due to the COVID-19 pandemic.

All PDVs will be listed for the ITT set.

6. EFFICACY ANALYSES

Efficacy analysis including primary, key secondary, secondary, and exploratory analysis will be performed primarily using the mITT set, with subjects grouped according to the treatment assigned at randomization. Available data from all randomized subjects regardless of adherence to the protocol will be included in the efficacy analyses; this includes data from subjects who discontinued study drug early but continued with study assessments. All efficacy data will be listed for the ITT set.

Unless otherwise noted, hypothesis testing and estimation of treatment effects will be performed with a mixed effect model for repeated measure (MMRM) that includes data from all three treatment arms (PM, PEOM, and Sham). The Sham arm will represent the pool of the two sham treatment groups: SM and SEOM (i.e., the two sham arms will be pooled into a single "control" group). All hypothesis tests for efficacy endpoints will be two-sided.

Unless otherwise noted, analysis of efficacy endpoints (primary, key secondary, secondary, and exploratory) in the overall population will be adjusted for the following randomization stratification factors (actual status) and baseline covariates:

For primary efficacy endpoint

- Presence of CNV in the fellow eye (yes; no)
- Baseline GA lesion area (< 7.5 mm^2 or ≥ 7.5 mm^2)

For key secondary, secondary, and exploratory endpoints

- Presence of CNV in the fellow eye (yes; no)
- Baseline GA lesion area (< 7.5 mm^2 or ≥ 7.5 mm^2)
- Baseline value of the endpoint

Of note, the randomization stratification factors were assessed at the screening visit. In the event of a change in status in the GA lesion area between the screening visit and the baseline visit (i.e., GA lesion area < 7.5 mm^2 at screening to ≥ 7.5 mm^2) then the baseline status will be used.

If a statistical model is not directly specified and if necessary, data will be analyzed with the following approaches:

- For continuous endpoints, the MMRM analysis described for the primary endpoint in Section 6.2.2.
- For categorical or ordinal variables, a Cochran Mantel Haenszel (CMH) test with the baseline category status, presence of CNV in the fellow eye (yes or no), and baseline GA lesion area (< 7.5 mm^2 or ≥ 7.5 mm^2) as stratification factors.

6.1. Estimands

The primary scientific research question of this study is to assess the effect of pegcetacoplan compared with that of sham at Month 12 regarding the impact on GA lesion progression under-real-life conditions following the ITT principal.

The primary estimand in the study is defined through the following 5 attributes:

- **Population:** GA subjects defined through inclusion and exclusion criteria in the mITT population.
- **Treatment conditions:** The treatment regimen of interest in this study is PM, PEOM, and Sham.
- **Variable (or endpoint):** Change in total area of GA lesions in the study eye from baseline measured by FAF to Month 12.
- **Strategy for addressing intercurrent events:** See description of intercurrent events below.
- **Population-level summary:** Difference in mean change of GA lesion from baseline to Month 12 between pegcetacoplan and sham groups according to MMRM analysis.

The intercurrent events that will be considered are:

- Treatment discontinuation
- Lost to follow up
- Withdrawal from the study

The intercurrent events will be handled with a treatment policy strategy whereby any measured value will be used as is. Missing data resulting from these intercurrent events will be handled implicitly within the MMRM analysis that assumes missing at random.

The key secondary estimands will be defined and analyzed in the similar fashion as the primary estimand. The primary and key secondary estimands are summarized in Table 2.

Table 2: Estimands of Study

Estimand	Definition	Attributes			
		**A: Population**	**B: Variable (or endpoint)**	**C: Strategy for addressing intercurrent event**	**D: Population-level summary**
		**Main Analyses**			
Primary	The effect of pegcetacoplan compared to sham at Month 12 in impact on GA progression	mITT	Change in the total area of GA lesions from baseline measured by FAF to Month 12	• Treatment policy strategy for subjects who discontinue treatment, are lost to follow-up, or withdraw from the study	Difference in mean change of GA lesion from baseline at Month 12 between pegcetacoplan and sham groups based on MMRM analysis
Key Secondary #1	The effects of pegcetacoplan compared to sham at Month 24 in impact on monocular maximum reading speed	mITT	Change in monocular maximum reading speed from baseline to Month 24	• Treatment policy strategy for subjects who discontinue treatment, are lost to follow-up, or withdraw from the study	Difference in mean change in monocular maximum reading speed from baseline at Month 24 between pegcetacoplan and Sham groups based on MMRM analysis
Key Secondary #2	The effects of pegcetacoplan compared to Sham at Month 24 in impact on mean FRI Index score	mITT	Change in mean FRI Index score from baseline to Month 24	• Treatment policy strategy for subjects who discontinue treatment, are lost to follow-up, or withdraw from the study	Difference in mean change in mean FRI Index score, from baseline at Month 24 between pegcetacoplan and sham groups based on MMRM analysis
Key Secondary #3	The effects of pegcetacoplan compared to Sham at Month 24 in impact on NL-BCVA score	mITT	Change in NL-BCVA score from baseline to Month 24	• Treatment policy strategy for subjects who discontinue treatment, are lost to follow-up, or withdraw from the study	Difference in mean change of NL-BCVA score from baseline at Month 24 between pegcetacoplan and sham groups based on MMRM analysis

6.2. Analyses of Primary Efficacy Endpoint

The primary efficacy endpoint is change from baseline to Month 12 in total area of GA lesion(s) in the study eye (in mm^2) based on FAF.

The hypotheses of interest and Type I error management for analyses of the primary endpoint are specified below and take precedence over that specified in the study protocol.

For the primary efficacy endpoint, sensitivity analyses (Section 6.2.3), supplementary analyses (Section 6.2.4), and subgroup analyses (Section 6.2.5) will be performed.

All analyses of the first 12 months will be performed at the Month 12 reporting as well as repeated at the final reporting. Unless otherwise specified, at the time of the final reporting, all analyses will be repeated using the data for the full 24 months.

6.2.1. Type I Error Management

The null hypotheses for the primary efficacy endpoint are the following:

- H_{1a}: There is no difference between PM and Sham in mean change from baseline to Month 12 in total area of GA lesion(s) in the study eye (in mm^2) based on FAF for the mITT set.
- H_{1b}: There is no difference between PEOM and Sham in mean change from baseline to Month 12 in total area of GA lesion(s) in the study eye (in mm^2) based on FAF for the mITT set.

For hypothesis testing of the primary efficacy endpoint, type I error will be controlled using a fixed sequencing approach by testing the two hypotheses (H_{1a} and H_{1b}) sequentially, beginning with H_{1b}. All hypothesis tests for the primary endpoint will be based on a two-sided alpha level of 0.05. The alpha will be adjusted in accordance with the protocol for the number of Data Monitoring Committee (DMC) unmasked reviews prior to the Month 12 analysis timepoint (i.e., 4 DMC unmasked reviews prior to Month 12: 2-sided alpha level = 0.0496). Testing for statistical significance proceeds to H_{1b} only if H_{1a} is statistically significant. The study will be considered positive if the first hypothesis (H_{1a}) is rejected.

6.2.2. Main Analysis of Primary Efficacy Endpoint

The primary endpoint will be analyzed in the mITT population with subjects grouped according to the treatment assigned at randomization.

A MMRM model will be used to analyze the primary endpoint. The analysis model will include treatment (PM, PEOM, sham), presence of choroidal neovascularization in the fellow eye at baseline (Yes, No) and baseline GA lesion area ($< 7.5\ mm^2$ or $\geq 7.5\ mm^2$) as fixed effects, time (study month, categorical) as a factor, the time × treatment interaction term, and the baseline GA lesion area ($< 7.5\ mm^2$ or $\geq 7.5\ mm^2$) × time interaction term. The least square [LS] mean change from baseline to Month 12 will be estimated from the model for each of three arms as well as the comparisons of each of the three arms to each other. For other time points of interest, LS mean change from baseline will be estimated and compared between treatments. For each estimated LS mean, the corresponding 95% CI will be presented based on the model. For the comparison of the LSmeans, the corresponding 95% CI and the 2-sided P-value along with the percentage difference (difference in LS means between the arms/the comparison group LS mean) will be

presented. A common unstructured covariance matrix will be used to model the within-subject errors, the sandwich estimator (Diggle, Liang, and Zeger 1994) will be used to estimate the standard errors of the fixed effects parameters, and the degrees of freedom will be partitioned into between-subject and within-subject portions. If there are convergence problems with the model, then a heterogeneous autoregressive (1) covariance matrix will be used. If convergence problems still exist, an autoregressive (1) covariance matrix will be used. The LS mean of the change from baseline in the total area of GA lesion(s) ± the standard error will be plotted over time by treatment group.

The observed values for the total area of GA lesion(s) will be summarized by treatment group and visit for both the study and the fellow eye. Summaries will present the descriptive statistics for baseline, absolute values and change from baseline data by visit.

The mean change from baseline in the total area of GA lesion (s) in the study eye ± the standard error will be plotted over time by treatment group.

All GA lesion (s) size data will be listed for the ITT set.

6.2.3. Sensitivity Analyses of Primary Efficacy Endpoint

Sensitivity analyses will be performed to evaluate the robustness of the primary analysis results. Analyses will be performed for the overall population based on the outcome of the primary analyses including all subjects in the mITT set.

6.2.3.1. Missing Data Analyses Based on Multiple Imputation

The following sensitivity analysis based on multiple imputation will be performed, as appropriate, using the same statistical approach as the one used in the primary analysis:

- Subjects with treatment discontinuation or study withdrawal assessed as potentially related to study drug or lack of efficacy will have missing data after the discontinuation or withdrawal imputed via a control-based imputation based on the sham control subjects. This will be based on the mITT set.
  - Subjects who discontinue the treatment or study due to reasons that are potentially related to study drug or lack of efficacy include
    - Subjects who discontinue due to ocular adverse event, adverse event related to treatment, or adverse event related to injection procedure
    - Subjects who withdraw consent due to lack of efficacy (specify field includes ‘Lack of efficacy’) or not otherwise specified as not related to efficacy (specify field includes “Not specified”)
- Subjects with a monotone missing data pattern due to treatment discontinuation or study withdrawal will have missing data after the last assessment explicitly imputed by multiple imputation using a tipping point analysis method assuming missing not at random (MNAR). This will be based on the mITT set.

Imputation for the non-monotone missing pattern (i.e., arbitrary missing pattern) will be performed prior to the multiple imputation for the monotone missing pattern (i.e., where a missing GA area measurement at a visit for a subject implies that GA area measurements at all subsequent visits for that subject are missing).

For the nonmonotone missing pattern, missing value(s) between two visits with measured GA area will be imputed using the monotone data MCMC method, which was first proposed by Li (1988) and Liu (1993) described the algorithm. Multiple imputation will then be carried out for monotone missing pattern.

The two imputation methods will be implemented in SAS using the three standard steps to generate inference from imputed data: imputation step, analysis step, and pooling step. Details are illustrated with SAS codes in Section 1.2.2.

- The missing data are filled in 1000 times to generate 1000 complete datasets.
- The 1000 complete datasets are analyzed by using the same approach as for the analysis for the primary objective.
- The results from the 1000 complete datasets are combined for the inference.

Methods used in the imputation step are described below.

- Pattern Mixture Model Method (a copy-reference approach): The pattern mixture model method will be implemented.
    - Subjects with monotone missing data in the pooled sham arm will have missing data imputed based on the observed values in the pooled sham arm.
    - For the active treatment arms, subjects with monotone missing data who discontinued treatment or withdrew from the study due to reasons that are potentially related to study drug or lack of efficacy will have missing data imputed based on the pooled sham arm.
    - For the active treatment arms, subjects with monotone missing data who did not discontinue treatment or withdraw from the study due to reasons that are potentially related to study drug or lack of efficacy will have missing data imputed based on the observed values in the corresponding treatment arm.
- Tipping Point Analysis Method (a delta-adjusted approach): The tipping point analysis method will be implemented for each active treatment arm under the MNAR assumption by searching for a tipping point that reverses the conclusion regarding positive treatment effect. For the pooled sham arm, subjects will have missing data imputed based on the observed values in the pooled sham arm. For the active treatments, subjects with monotone missing data without treatment discontinuation or early withdrawal will have missing data imputed based on the observed values in the corresponding treatment arm. For the active treatment arms, subjects with monotone missing data due to treatment discontinuation or early withdrawal will have the missing data imputed based on the available values (observed values plus values imputed for non-monotone missing data) in the same treatment arm with a shift parameter added to the imputed values. Multiple imputation will be implemented on the differences between two consecutive visits, with the shift parameter allocated to

the missing data point(s) proportionally across timepoints. (e.g., For a shift parameter of 0.48 mm^2 at Month 12, incremental shifts of 0.08 mm^2 would apply to Month 2, Month 4, … and Month 12, respectively. 0.08 mm^2 x 6 visits = 0.48 mm^2) The range of the shift parameters will be from 0.06 mm^2 to 0.72 mm^2 by increments of 0.06 mm^2 for Month 12 and 0.12 mm^2 to 1.44 mm^2 for Month 24 for the difference in the change of GA area from baseline between each pegcetacoplan arm (PM or PEOM) and Sham arm. The precision of the tipping point will be at two decimal points. A tipping point may not exist within reasonable clinical assumptions.

For each analysis, the number and percent of subjects without monotone missing data, the number of subjects with monotone missing data and a breakdown of reasons will be reported. The reasons will include monotone missing without treatment discontinuation or early withdrawal, monotone missing due to treatment discontinuation or early withdrawal due to reasons that are potentially related to study drug or lack of efficacy, and monotone missing due to treatment discontinuation or early withdrawal not due to reasons that are potentially related to study drug or lack of efficacy.

6.2.3.2. Other Sensitivity Analyses

The primary endpoint will also be analyzed without pooling the two sham arms (SM and SEOM). The comparison for pegcetacoplan and sham injection within each dose schedule (i.e., PM vs SM and PEOM vs SEOM, respectively) will be conducted using the MMRM analyses described in Section 6.2.2.

The primary endpoint will also be summarized excluding GA total area assessments with an indeterminate boundary. The summary and presentation of the observed values for the total area of GA lesion(s) by treatment group and visit described in Section 6.2.2 will be repeated. The summary and presentation of the analysis results for all timepoints described in Section 6.2.2 will be repeated.

6.2.4. Supplemental Analyses of the Primary Efficacy Endpoint

6.2.4.1. COVID-19 Adjusted Estimand

Despite the occurrence of the COVID-19 pandemic, the scientific question of interest in this study remains unchanged. The primary scientific research question of this study is to assess the effect of pegcetacoplan compared with that of sham at Month 12 on GA lesion progression under-real life conditions without COVID-19 pandemic impact.

To manage the increase in missed/not received injections due to COVID-19 pandemic (as collected on the eCRF), i.e., undertreatment with an expected relevant impact on efficacy, the hypothetical strategy will be used whereby assessments after the intercurrent event (relevant undertreatment) occurs will be set to missing/censored in the analysis. The threshold for missed/not received injections due to the COVID-19 pandemic is missing a pre-specified number of scheduled injections prior to the analysis timepoint of interest. For the Monthly schedule group, this is missing 2 or more injections and for the EOM schedule group, this is missing 1 or more injections prior to the Month 12 visit attributable due to the COVID-19 pandemic.

In terms of the underlying cause of the excessive missing/not received injections and subsequent missing data, it is assumed that the missingness resulting from censoring occurs at random because of the ongoing pandemic and not because of the subjects' unobserved outcome, so

missing data resulting from this intercurrent event in the primary analysis model will be handled implicitly in the MMRM analysis.

The number of subjects with any censoring included in the model, the number of subjects with any censoring not included in the model, and the number of assessments censored by visit will be presented for each treatment group.

All summaries and presentations of the observed values for the total area of GA lesion(s) in the study eye by treatment group and visit described in Section 6.2.2 will be repeated for the COVID-19 adjusted estimand. All summaries and presentations of the analysis results for all timepoints described in Section 6.2.2 will be repeated for the COVID-19 adjusted estimand. The same plots as for the primary endpoint analysis will be presented.

In addition, a supplemental analysis will also be performed where the threshold for missed/not received injections will be restricted to external events attributable to COVID-19. These events are defined based on the COVID-19 collection form and include COVID-19 diagnosis, COVID-19 Suspected, Site Closure, Travel Ban, Shelter in Place, City Lockdown, and Other. Except for the plots, the analyses described above will be repeated for the threshold based only on these external events attributable to COVID-19.

6.2.4.2. Per Protocol Set

The main analysis described in Section 6.2.2 will be repeated using the Month 12 PP set to investigate the impact of changing the population in the estimand. Any GA lesion area assessments that are deemed not valid according to what is described in Section 4.5 will be set to missing in this analysis. The summary and presentation of the observed values for the total area of GA lesion(s) by treatment group and visit described in Section 6.2.2 will be repeated. The summary and presentation of the analysis results for all timepoints described in Section 6.2.2 will be repeated.

6.2.4.3. Rate of Change Analyses

The mean rate of change in GA area (i.e., slope) will be compared between each pegcetacoplan arm and the pooled sham arm by use of linear mixed effects model assuming time as continuous and linear ("slope model"). The analysis model will include treatment (PM, PEOM, sham), presence of choroidal neovascularization in the fellow eye at baseline (Yes, No), baseline GA lesion area ($< 7.5\ mm^2$ or $\geq 7.5\ mm^2$) as fixed effects, time (study month, continuous assuming linearity), the time × treatment interaction term as well as the baseline GA lesion area × time interaction term. The response variable will be the GA lesion area. A common unstructured covariance matrix will be used to model the within-subject errors, the sandwich estimator will be used to estimate the standard errors of the fixed effects parameters, and the degrees of freedom will be partitioned into between-subject and within-subject portions. If there are convergence problems with the model, then a heterogeneous autoregressive (1) covariance matrix will be used. If convergence problems still exist, an autoregressive (1) covariance matrix will be used. The mean rate of change (slope), standard error, and confidence interval will be estimated for the baseline to Month 12 as well as the baseline to Month 6 and Month 6 to Month 12 periods for each treatment group. In addition, the estimated difference in slopes among the treatment groups along with the 95% CIs and p-value will be reported. The slopes and 95% CIs will be converted to an annualized rate of growth for each period and each treatment group.

The observed values for the total area of GA lesion(s) will be summarized by treatment group and visit for the baseline to Month 6 and Month 6 to Month 12 time periods. Summaries will present the descriptive statistics for baseline, absolute values and change from baseline to Month 6 and change from Month 6 to Month 12 data. In addition, the annualized rate of growth for each time period will also be presented.

The mean rate of change in GA area will be compared between each pegcetacoplan arm and the pooled sham arm by use of a piecewise linear mixed effect model assuming time as continuous and piecewise linear ("piecewise slope model"). The analysis to be performed will be similar to what is described above in the "slope model" except that a knot at the Month 6 visit will be added which allows for the slope of lesion growth to differ between the two periods for each of the treatment groups. The same presentation as for the "slope model" will be prepared except for the Baseline to Month 12 slope. Additionally, an investigation of the impact of changing the knot to Month 2, 4, 8, 10 will be explored if necessary.

6.2.5. Subgroup Analyses of Primary Efficacy Endpoint

Subgroup analyses will be performed to evaluate the consistency of the primary analysis results across subgroups defined by demographic and baseline characteristics. Analyses will be performed for the primary efficacy endpoint (change from baseline in total area of GA lesion(s) in the study eye at Month 12) for each of the following subgroups (as appropriate per actual subgroup sample size, levels with low sample size may be pooled to allow for an analysis to be conducted):

- Age Group (<75 years, 75 to <85 years, ≥85 years)
- Sex (male, female)
- Race (White, Black or African American, Asian, American Indian or Alaskan Native, Native Hawaiian or other Pacific Islander, multiple, unknown)
  - Note: If most subjects (e.g., >90%) are of a single race, this analysis will not be conducted.
- Geographic Region (United States vs Rest of World)
- Subgroups indicative of disease severity at baseline
  - Study eye baseline GA lesion size (<7.5 mm^2; ≥7.5 mm^2)
  - Study eye baseline GA lesion size categories (approximately tertiles based on APL2-303/304 combined data*)
  - Study eye baseline NL-BCVA categories (≥70, ≥60 - <70, ≥35 - <60, <35 ETDRS letters)
  - Study eye baseline NL-BCVA categories (<60 vs. ≥60 ETDRS letters)
  - Study eye baseline LL-BCVA categories (approximately tertiles based on APL2-303/304 combined data*)
  - Study eye baseline monocular maximum reading speed categories (approximately tertiles based on APL2-303/304 combined data*)
  - Baseline FRI Level (1,2,3,4)

- Subgroups associated with GA progression
  - Study eye baseline GA focality (multifocal, unifocal)
  - Study eye baseline GA lesion location (subfoveal involvement, without subfoveal involvement)
  - Baseline GA laterality (bilateral GA (with or without CNV in fellow eye) vs. Study eye GA Only)
  - Baseline CNV in fellow eye (Fellow eye CNV vs. No fellow eye CNV)
  - Study eye baseline LLD categories (<20 vs. ≥ 20 ETDRS letters)
  - Study eye baseline LLD categories (approximately tertiles based on the APL2-303/304 combined data*)
  - Study eye baseline NL-BCVA (<60 vs. ≥60 ETDRS letters) and LLD (<20 vs. ≥ 20 ETDRS letters) combinations

*Approximately tertiles will be specified consistently across the APL2-303/304 studies to the nearest clinically relevant level.

For the primary efficacy endpoint, the approach described in the Section 6.2.2 will be used for each subgroup analysis based on the data subset for the subject subgroup of interest. Baseline covariates included in the main analysis but no longer relevant given the subgroup of interest will be excluded from the model. The estimated treatment effects (PM vs. Sham; PEOM vs. Sham) and corresponding 95% CIs and p-values from the models will be displayed graphically for each pegcetacoplan treatment arm and each level of the subgroups specified (e.g., via forest plots).

6.2.6. Full 24 Month Analyses

In general, the same analysis approach for the changes from baseline GA lesion size for the final reporting of the full 24 months will be repeated as described in Sections 6.2.2, 6.2.3, 6.2.4, 6.2.5. Key differences include:

- The definition of excessive missed/not received injection in the supplementary estimand will be defined based on the Month 24 period. The threshold for missed/not received injections due to the COVID-19 pandemic is missing 4 or more injections for the Monthly schedule group and 2 or more injections for the EOM schedule group prior to the Month 24 visit attributable due to the COVID-19 pandemic.
- The Month 12 PP set will be replaced with the Month 24 PP set for the supplementary analysis.

6.2.7. Exploratory Endpoints Closely Related to the Primary Efficacy Endpoint

Analyses of the following endpoints closely related to the primary efficacy endpoint, will be performed for the mITT set to provide supplemental information.

6.2.7.1. Change in Square Root of GA Area

For each subject and timepoint, the square root of GA area will be calculated. The change from baseline in square root of GA area will be analyzed using the same methodology as described for the primary endpoint analysis in Section 6.2.2, except that change from baseline in the square root of GA area will be the response variable. This analysis will be conducted both for the first 12 months summaries as well as the full 24 months summaries.

6.2.7.2. Percent Change in GA Area and Square Root of GA Area

For each subject and timepoint, the percent change in GA area will be defined as the change from baseline in GA area (mm^2) divided by the GA area (mm^2) at baseline and the percent change in square root of GA area will be defined as the change from baseline in GA area (mm) divided by the GA area (mm) at baseline. The percent change in GA area from baseline and percent change in square root of GA area will be analyzed using the same methodology as described for the primary endpoint analysis in Section 6.2.2, except that percent change from baseline will be the response variable. This analysis will be conducted both for the first 12 months summaries as well as the full 24 months summaries.

6.3. Analyses of Key Secondary Efficacy Endpoints

The key secondary efficacy endpoints are listed in Section 2.2.2. At the Month 12 reporting, all key secondary efficacy endpoints will be evaluated based on data from baseline to Month 12 (see Section 3.5). For the Month 12 analyses, post-baseline visits, up to and including Month 12, will be included and nominal p-values will be presented. At the time of the final study analysis, all key secondary efficacy endpoints will be evaluated based on data from baseline to Month 24, and formal statistical testing for key secondary endpoints will be performed as described in Section 6.3.1. Unless otherwise specified, all secondary efficacy endpoints will be analyzed for the mITT set.

6.3.1. Type I Error Management

The planned submission of pegcetacoplan in GA consists of 2 studies of very similar design (APL2-303 and APL2-304), each with multiple endpoints. It is planned to control the type I error rate for the primary endpoint hypotheses testing at the level of the individual studies (as described in Section 6.2.1), and at a level of the submission as a whole for the key secondary endpoints hypotheses testing based on pooling APL2-303 and APL2-304. Figure 2 presents the overall hypotheses testing strategy for the APL2-303 and APL2-304 studies.

The primary endpoint hypotheses in the PM vs. Sham and PEOM vs. Sham will be tested in a hierarchical order within a study. If both hypothesis tests for the primary endpoint in both studies are statistically significant, then hypotheses testing will be performed for the key secondary endpoints within the pooled APL2-303/APL2-304 data for hypotheses $H_{2a} – H_{4b}$ and within the APL2-304 study for H_{5a} and H_{5b} at the presented α level. The key secondary efficacy endpoints hypotheses that will be tested in the pooled APL2-303/APL2-304 studies are below:

- H_{2a} There is no difference between PM and Sham regarding mean change from baseline in monocular maximum reading speed (study eye), as assessed by MNRead or Radner Reading Charts at Month 24.

- H_{2b}: There is no difference between PEOM and Sham regarding mean change from baseline in monocular maximum reading speed (study eye), as assessed by MNRead or Radner Reading Charts at Month 24.
- H_{3a}: There is no difference between PM and Sham regarding mean change from baseline in mean FRI Index score at Month 24.
- H_{3b}: There is no difference between PEOM and Sham regarding mean change from baseline in mean FRI Index score at Month 24.
- H_{4a}: There is no difference between PM and Sham regarding mean change from baseline in NL-BCVA score at Month 24 (study eye) as assessed by ETDRS chart.
- H_{4b}: There is no difference between PEOM and Sham regarding mean change from baseline in NL-BCVA score at Month 24 (study eye) as assessed by ETDRS chart.

Hypothesis tests will be conducted in the order indicated by the arrows in Figure 2 and will continue as long as all preceding hypotheses are rejected at a given α level. If both the PM vs. Sham and the PEOM vs. Sham comparisons are statistically significant for a given key secondary endpoint, then the α will be allocated equally to the other key secondary hypotheses. At the study-level, the type I error rate (one-sided) is controlled at 0.025 for the primary endpoint hypotheses. In the submission, the type I error rate (one-sided) is controlled at 0.000625 (0.025^2) for the primary endpoint hypotheses and at 0.025 for the key secondary endpoint hypotheses. All hypothesis tests will be adjusted for the number of DMC unmasked reviews prior to the Month 12 analysis.

Type I error will be controlled by the testing procedure via the graphical methods approach (Bretz 2009). All confidence intervals and p-values will be presented without adjustments.

If at least one of the hypothesis tests for the primary endpoint in the APL2-304 study is not statistically significant but both hypothesis tests for the primary endpoint in the APL2-303 study are statistically significant, then any hypothesis testing for the key secondary endpoints will not be rejected at the submission level. Within the APL2-303 study, hypotheses testing for the key secondary endpoints will be tested as shown in Figure 3 and type I error will be controlled using a fixed sequencing approach. All hypotheses tests will be adjusted for the number of DMC unmasked reviews prior to the Month 12 analysis. All confidence intervals and p-values will be presented without adjustments.

Figure 2: APL2-303 and APL2-304 Hypothesis Testing and Type I Error Control

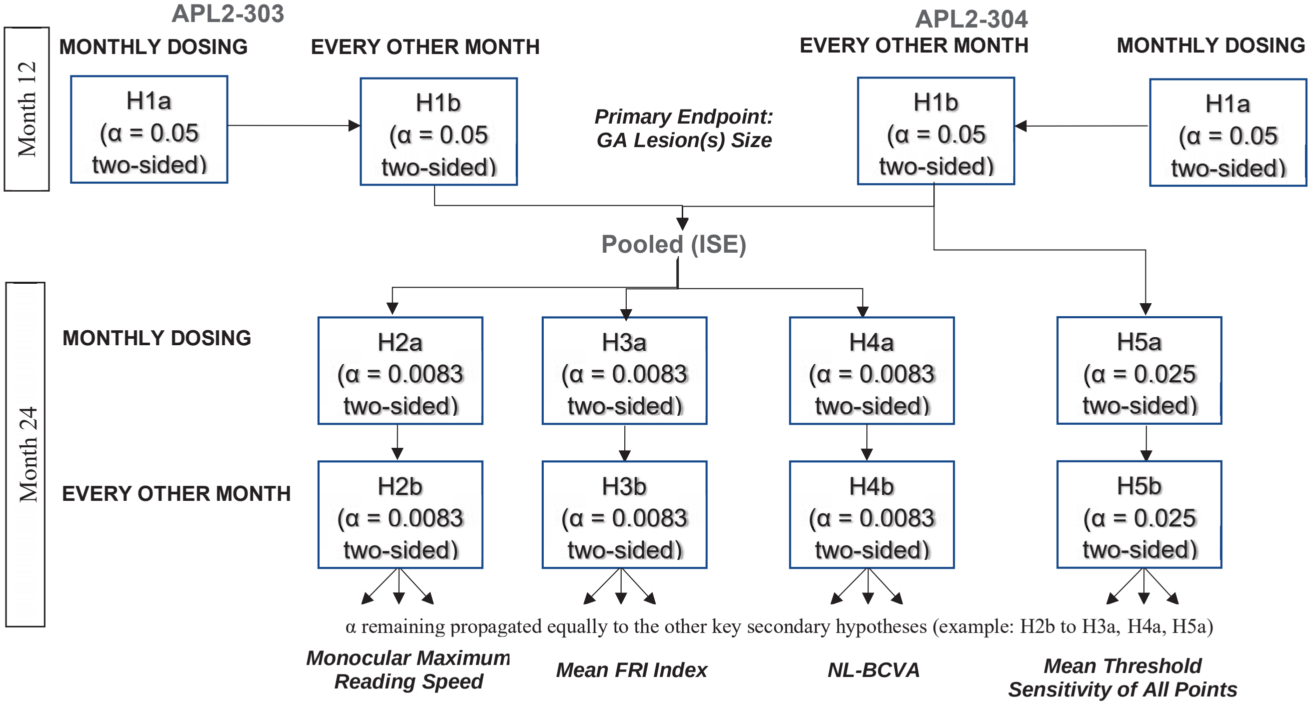

Endpoint data are compared to sham injections in hypotheses testing. Hypotheses can only be tested in sequential order as indicated by arrows. Sequential testing will continue as long as all preceding hypotheses can successfully be rejected at the given alpha level. At the study-level, the Type I error rate (one-sided) is controlled at 0.025 for the primary endpoint hypotheses. In the submission, the Type-I error rate (one-sided) is controlled at 0.000625 (0.025^2) for the primary endpoint hypotheses and at 0.025 for the key secondary endpoints.

Figure 3: APL2-303 Hypothesis Testing and Type I Error Control If There is No α Remaining in APL2-304 to Allow Pooling Secondary Functional Endpoints

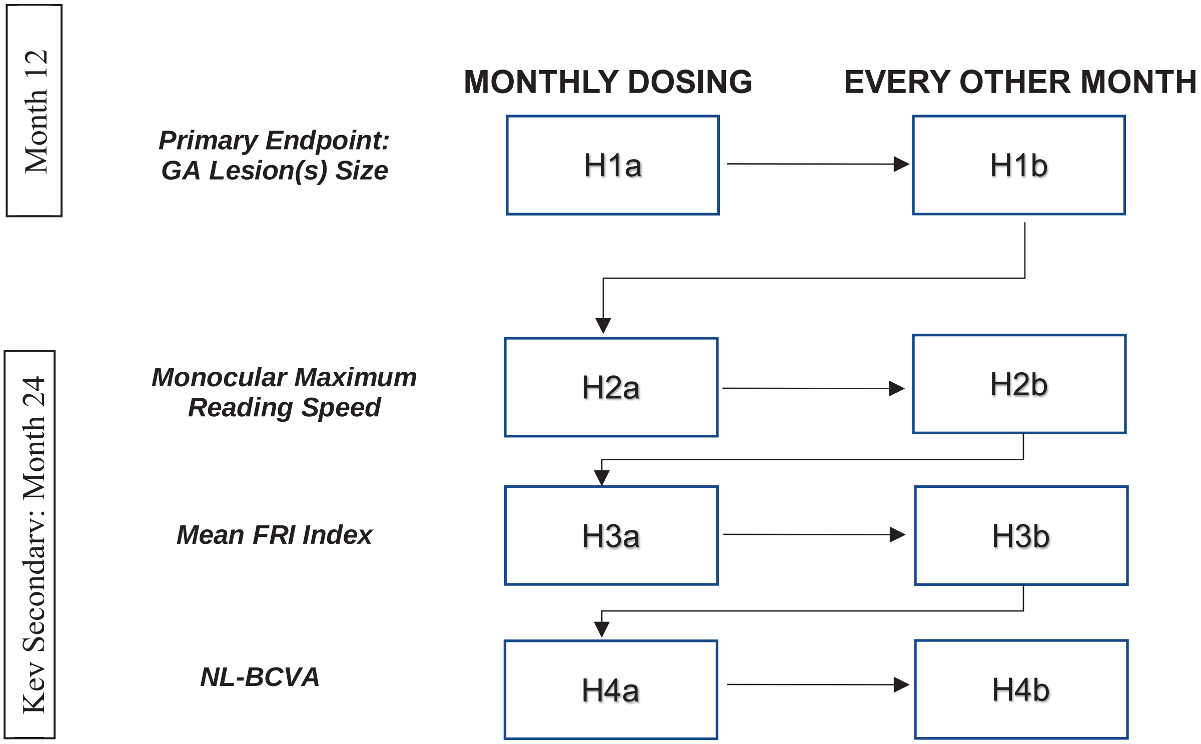

Endpoint data are compared to sham injections in hypotheses testing. Hypotheses can only be tested in sequential order as indicated by arrows. Sequential testing will continue as long as all preceding hypotheses can successfully be rejected at the given alpha level. This testing tree will only be used when APL2-304 cannot pass α from the primary endpoint testing to the pooled secondary functional endpoint testing.

6.3.2. Main Analyses of Key Secondary Efficacy Endpoints

6.3.2.1. Monocular Maximum Reading Speed

Maximum reading speed will be calculated per MNRead or Radner user manuals, with no adjustment for reading inaccuracy. An additional step to cap resulting reading speed values at a maximum of 300 words per minute will be implemented. Values above 300 words per minute exceed values reported in the literature for adults with no vision impairment for MNRead (Calabrese et al. 2016) or Radner (Radner et al. 2002; Radner and Diendorfer 2014) assessments. Maximum reading speed will be calculated as the mean of the three highest nonzero reading speeds (or two, or one value, as available) (Baskaran et al. 2019; Patel et al. 2011), except when all words per minute are calculated as 0: then the maximum reading speed will be calculated as 0.

Monocular maximum reading speed in the study eye will be summarized and analyzed in a similar fashion as the primary efficacy endpoint using MMRM described in Section 6.2.2. The model will include treatment (PM, PEOM, Sham), presence of CNV in the fellow eye (yes or no), and baseline GA lesion area (< 7.5 mm^2 or ≥ 7.5 mm^2) as fixed effects; baseline monocular maximum reading speed as well as the chart type (MNRead or Radner) as covariates, time (in months) as a factor; the time × treatment interaction term as well as the baseline monocular maximum reading speed × time interaction term.

The observed values for the monocular maximum reading speed will be summarized by treatment group and visit for both the study and the fellow eye. Summaries will present the descriptive statistics for baseline, absolute values and change from baseline data.

The mean change from baseline in the monocular maximum reading speed of the study eye ± the standard error as well as the LS mean of the change from baseline in the monocular maximum reading speed of the study eye ± the standard error will be plotted over time by treatment group.

In addition, the categorical changes (improvements of ≥ 20 to <40, ≥ 40 to <60, ≥ 60 and minimal/no change (-20 to 20), worsening of ≥ 20 to <40, ≥ 40 to <60, ≥ 60) for the study eye will be summarized by treatment group and visit.

The shift from baseline in the monocular maximum reading speed for the study eye categories over time will be presented by treatment group for the following categories:

- < 40 wpm
- 40 to < 80 wpm
- 80 to <160 wpm
- ≥ 160 wpm

All monocular maximum reading speed data will be listed for the ITT set.

6.3.2.2. Mean FRI Index Score

The mean FRI Index score will be calculated per the scoring algorithm in the FRI Index user's manual.

The mean FRI Index score will be summarized and analyzed in a similar fashion as the primary efficacy endpoint using MMRM described in Section 6.2.2. The model will include treatment

(PM, PEOM, Sham), presence of CNV in the fellow eye (yes or no), and baseline GA lesion area (< 7.5 mm^2 or ≥ 7.5 mm^2) as fixed effects; baseline mean FRI Index score and baseline study eye status (Better-seeing Eye vs. Worse-seeing Eye) as covariates, time (in months) as a factor; the time × treatment interaction term as well as the baseline mean FRI Index score × time interaction term.

The observed values for the mean FRI Index score will be summarized by treatment group and visit. Summaries will present the descriptive statistics for baseline, absolute values and change from baseline data.

The mean change from baseline in the mean FRI Index score ± the standard error as well as the LS mean of the change from baseline in the mean FRI Index score ± the standard error will be plotted over time by treatment group.

In addition, the mean FRI Index score will be converted to the FRI Level variable, as per the user manual, ranging from 1 (Unable to do) to 4 (Totally independent). The categorical changes from baseline (improvements (an increase in FRI level), minimal/no change (no change in FRI level), and worsening (decrease in FRI level)) will be summarized by treatment group and visit.
The shift from baseline in the FRI level over time will be presented by treatment group.

All FRI data will be listed for the ITT set.

6.3.2.3. NL-BCVA Score

The mean NL-BCVA score for the study eye will be summarized and analyzed in a similar fashion as the primary efficacy endpoint using MMRM described in Section 6.2.2. The model will include treatment (PM, PEOM, Sham), presence of CNV in the fellow eye (yes or no), and baseline GA lesion area (< 7.5 mm^2 or ≥ 7.5 mm^2) as fixed effects; baseline NL-BCVA score as a covariate, time (in months) as a factor; the time × treatment interaction term as well as the baseline NL-BCVA score × time interaction term. Note, for the second year, only timepoints that were measured for all three treatment groups will be included in the model.

The observed values for the NL-BCVA will be summarized by treatment group and visit for both the study and the fellow eye. Summaries will present the descriptive statistics for baseline, absolute values and change from baseline data.

The mean change from baseline in the NL-BCVA score for the study eye ± the standard error as well as the LS mean of the change from baseline in the NL-BCVA score for the study eye ± the standard error will be plotted over time by treatment group.

In addition, the categorical changes (improvements of ≥15, ≥10 to <15, ≥5 to <10, and minimal/no change (-4 to 4), worsening of ≥5 to <10, and ≥10 to <15 and ≥15) for the study eye will be summarized by treatment group and visit.

The shift from baseline in the NL-BCVA of the study eye categories (≥70, ≥60 - <70, ≥35 - <60, ≥20 - <35, <20 ETDRS letters over time will be presented by treatment group and visit. The above-mentioned ETDRS letter thresholds are based on WHO definitions for vision impairment and blindness. WHO criteria were implemented in an attempt to capture the progressive nature of

Geographic Atrophy and the ultimate treatment goal of avoiding successive vision impairment and blindness through application of active treatment (Pegcetacoplan vs. Sham).[1]

The average NL-BCVA score over the last 3 months for each reporting period (Month 10 - Month 12 for the Month 12 reporting; Month 22 - Month 24 for the Month 24 reporting) will be calculated for each subject who has at least one NL-BCVA assessment in these windows. The observed values for the NL-BCVA and changes from baseline will be summarized by treatment group for the study eye. Summaries will present the descriptive statistics for baseline, absolute values and change from baseline data.

All NL-BCVA data will be listed for the ITT set.

6.3.3. Sensitivity Analyses of Key Secondary Efficacy Endpoints

The missing data analyses based on imputation described in Section 6.2.3.1 will be repeated for the key secondary efficacy endpoints at the time of the final study analysis for the full 24 months only. The key secondary endpoints will also be analyzed without pooling the two sham arms (SM and SEOM) for the full 24 months only.

In addition, to assess the potential effect of different assessment tools, the main analysis of monocular maximum reading speed will be repeated by reading chart type. Maximum reading speed with adjustment for reading inaccuracy will also be analyzed, only in the study eye, for the first 12 months as well as the full 24 months.

6.3.4. Supplementary Analyses of Key Secondary Efficacy Endpoints

The supplementary analyses described for the primary endpoint in Section 6.2.4.1 and Section 6.2.4.2 will also be performed for the key secondary endpoints for the first 12 months as well as the full 24 months.

6.3.5. Subgroup Analyses of Key Secondary Efficacy Endpoints

The subgroup analyses of the key secondary endpoints will be performed for the full 24 months. Select subgroup analyses of the key secondary endpoints may be performed at Month 12, if necessary, to support regulatory filings. The subgroups evaluated for key secondary endpoints will be the same as evaluated for the primary efficacy endpoint (see Section 6.2.5).

In addition, the following subgroup analyses will be performed:

<u>Monocular Maximum Reading Speed</u>

- Study eye baseline monocular maximum reading speed (<60, 60-<160, ≥160 wpm)

<u>Mean FRI Index score</u>

- Baseline Study Eye Status (Better-seeing Eye vs. Worse-seeing Eye)

For each endpoint, a MMRM similar to that specified for the main analysis of the key secondary endpoints (Section 6.3.2) will be used for each subgroup analysis based on the data subset for the subject subgroup of interest. Baseline covariates included in the main analysis but no longer

[1] approximated from 6/18; see https://icd.who.int/browse11/l-m/en#/http://id.who.int/icd/entity/1103667651

relevant given the subgroup of interest will be excluded from the model. The estimated treatment effects (PM vs. Sham or PEOM vs. Sham) and corresponding 95% CIs and p-values from the models will be displayed graphically for each pegcetacoplan arm and each level of the subgroups specified (e.g., via forest plots).

6.4. Analyses of Other Secondary Efficacy Endpoints

The secondary efficacy endpoints are listed in Section 2.2.3. At the time of the Month 12 reporting, all secondary efficacy endpoints will be evaluated based on data from baseline to Month 12 (see Section 3.5). For the Month 12 analyses, post-baseline visits, up to and including Month 12, will be included. At the time of the final study analysis, all secondary efficacy endpoints will be evaluated based on data from baseline to Month 24. For the Month 24 analyses, post-baseline visits, up to and including Month 24, will be included.

Unless otherwise specified, all secondary efficacy endpoints will be analyzed for the mITT set.

6.4.1. Low Luminance Best-Corrected Visual Acuity (LL-BCVA)

LL-BCVA of the study eye will be summarized and analyzed in the similar fashion as the primary efficacy endpoint using MMRM described in Section 6.2.2. The model will include treatment (PM, PEOM, Sham), presence of CNV in the fellow eye (yes or no), and baseline GA lesion area (< 7.5 mm^2 or ≥ 7.5 mm^2) as fixed effects; baseline LL-BCVA score as a covariate, time (in months) as a factor; the time × treatment interaction term as well as the baseline LL-BCVA score × time interaction term. Note, for the second year, only timepoints that were measured for all three treatment groups will be included in the model.

The observed values for the LL-BCVA will be summarized by treatment group and visit for both the study and the fellow eye. Summaries will present the descriptive statistics for baseline, absolute values and change from baseline data.

The mean change from baseline in the LL-BCVA score in the study eye ± the standard error as well as the LS mean of the change from baseline in the LL-BCVA score in the study eye ± the standard error will be plotted over time by treatment group.

The categorical changes (improvements of ≥15, ≥10 to <15, ≥5 to <10, and minimal/no change

(-4 to 4), worsening of ≥5 to <10, and ≥10 to <15 and ≥15) for the study eye will be summarized by treatment group and visit.

Additionally, the average LL-BCVA score over the last 3 months for each reporting period (Month 10 - Month 12 for the Month 12 reporting; Month 22 - Month 24 for the Month 24 reporting) will be calculated for each subject who has at least one LL-BCVA assessment in these windows. The observed values for the LL-BCVA and changes from baseline will be summarized by treatment group for the study eye. Summaries will present the descriptive statistics for baseline, absolute values and change from baseline data.

The same subgroup analyses as described for NL-BCVA in Section 6.3.5 will also be repeated for LL-BCVA for the full 24 months.

All LL-BCVA data will be listed for the ITT set. In addition, the low luminance deficit will be calculated as the difference between NL-BCVA and LL-BCVA and listed for the ITT set.

6.4.2. Total Area of GA lesion(s) at Each Planned Assessment

The change from baseline at each planned assessment (other than Month 12 and Month 24) in the total area of GA lesion(s) in the study eye, as assessed by FAF, will be estimated from the MMRM analyses described in Section 6.2.2.

6.4.3. Monocular Critical Print Size

Critical print size will be calculated as the smallest print size which support reading speed at 80% of the maximum reading speed, without adjustment for reading inaccuracy (Baskaran et al. 2019).

Monocular critical print size of the study eye will be summarized and analyzed in a similar fashion as the primary efficacy endpoint using MMRM described in Section 6.2.2. The model will include treatment (PM, PEOM, Sham), presence of CNV in the fellow eye (yes or no), and baseline GA lesion area (< 7.5 mm^2 or ≥ 7.5 mm^2) as fixed effects; baseline monocular critical print size as well as the chart type (MNRead or Radner) as covariates, time (in months) as a factor; the time × treatment interaction term as well as the baseline monocular critical print size × time interaction term.

The observed values for the monocular critical print size will be summarized by treatment group and visit for both the study and the fellow eye. Summaries will present the descriptive statistics for baseline, absolute values and change from baseline data.

To assess the potential effect of different assessment tools, the analysis of monocular critical print size will be repeated by chart type.

All monocular critical print size data will be listed for the ITT set.

6.4.4. NEI VFQ-25 Distance Activity Subscale Score

NEI VFQ-25 distance activity subscale score will be calculated per the scoring algorithms in user's manual.

NEI VFQ-25 distance activity subscale score will be summarized and analyzed in a similar fashion as the primary efficacy endpoint using MMRM described in Section 6.2.2. The model will include treatment (PM, PEOM, Sham), presence of CNV in the fellow eye (yes or no), and baseline GA lesion area (< 7.5 mm^2 or ≥ 7.5 mm^2) as fixed effects; baseline NEI VFQ-25 distance activity subscale score and baseline study eye status (Better-seeing Eye vs. Worse-seeing Eye) as covariates, time (in months) as a factor; as well as the time × treatment interaction term as well as the baseline NEI VFQ-25 distance activity subscale score × time interaction term.

The observed values for the NEI VFQ-25 distance activity subscale score will be summarized by treatment group and visit. Summaries will present the descriptive statistics for baseline, absolute values and change from baseline data.

The categorical changes (improvements of >5 units and minimal/no change (-5 to 5), worsening of a decrease of >5 units) will be summarized by treatment group and visit.

In addition, the shift from baseline in NEI VFQ-25 distance activity subscale score categories (>80, >65 - ≤80, >50 - ≤65, ≥35 - ≤50, <35) will be summarized by treatment group and visit.

All NEI VFQ-25 data will be listed for the ITT set.

6.5. Multiplicity Adjustment

No hypothesis testing of the (non-key) secondary endpoints will be performed. Control of Type I error for (non-key) secondary endpoints is not applicable.

6.6. Analyses of Exploratory Endpoints

The exploratory efficacy endpoints are listed in Section 2.2.4. At the time of Month 12 reporting, all exploratory efficacy endpoints will be evaluated based on data from baseline to Month 12 (see Section 3.5). For the Month 12 analyses, post-baseline visits, up to and including Month 12, will be included. At the time of the final study analysis, all exploratory efficacy endpoints will be evaluated based on data from baseline to Month 24. For the Month 24 analyses, post-baseline visits, up to and including Month 24, will be included.

Unless otherwise specified, all exploratory efficacy endpoints will be analyzed for the mITT set.

6.6.1. NEI VFQ-25 and NEI VFQ-39 Additional Analyses

Descriptive summary statistics for baseline, absolute values and change from baseline data will be provided by treatment group and visit for the following NEI VFQ-25 and NEI VFQ-39 scores:

- NEI VFQ-39 distance activity subscale
- NEI VFQ-25 composite score
- NEI VFQ-39 composite score
- NEI VFQ-25 near activity subscale score
- NEI VFQ-39 near activity subscale score
- NEI VFQ-25 driving subscale score (for subjects who are currently driving at baseline)

The shift from baseline in NEI VFQ score categories (>80, >65 - ≤80, >50 - ≤65, ≥35 - ≤50, <35) will be summarized by treatment group and visit for the scores described above.

In addition, the categorical changes (improvements of >5 units and minimal/no change (-5 to 5), worsening of a decrease of >5 units) will be summarized by treatment group and visit for the following NEI VFQ-25 Scores: Composite and Near Activity.

For the NEI VFQ-25 Driving subscale score, the number and percentage of subjects with a worsening driving outcome compared to baseline will be summarized by treatment group and visit. Each visit will display the number of patients who were driving at baseline with at least one of the worsening driving outcomes. The categories to determine worsening driving outcomes are: conversion to no longer currently driving, increase in difficulty driving in familiar places in daytime, increase in difficult driving at night or stopped doing this due to vision, increase in difficulty driving in difficult conditions or stopped doing this due to vision.

All NEI VFQ-25 data will be listed for the ITT set.

6.6.2. Binocular Maximum Reading Speed and Critical Print Size

Descriptive summary statistics for baseline, absolute values and change from baseline data will be provided by treatment group and visit for the binocular maximum reading speed and critical print size with no adjustment for reading inaccuracy. All binocular maximum reading speed and critical print size data will be listed for the ITT set.

Categorical changes over time and categorical shifts from baseline will be presented, as will be done for the monocular reading speed.

6.6.3. GA Lesion (s) Study Eye to Fellow Eye Comparisons

Descriptive summary statistics will be provided by treatment and visit for the change from baseline in total area of GA Lesion(s) for the study eye as well as for the fellow eye in subjects with bilateral GA with fellow eyes that satisfy the following characteristics at baseline:

- Absence of CNV in the medical history
- Baseline GA lesion size between 2.5 and 17.5 mm^2
- Presence of any pattern of hyperautofluorescence in the junctional zone of GA
- GA not confluent with any peripapillary atrophy

The change from baseline in total area of GA Lesion(s) ± the standard error in the study eye as well as the fellow eye will be plotted over time by treatment group for the subjects with bilateral GA with fellow eyes that satisfy the above characteristics.

6.6.4. Digital Reading Index and Visual Function Application

The digital reading index will only be analyzed at the Month 24 reporting while the digital visual functional application will be analyzed at both the Month 12 and Month 24 reporting.

Descriptive summary statistics for baseline, absolute values, and change from baseline data to Month 12 and Month 24 will be provided by treatment group for digital reading index.

Additionally, the average digital reading index score over the last 3 months for each reporting period (Month 10 - Month 12 for the Month 12 reporting; Month 22 - Month 24 for the Month 24 reporting) will be calculated for each subject who has at least one digital reading index assessment in these windows. The observed values for the digital reading index and changes from baseline will be summarized by treatment group. Summaries will present the descriptive statistics for baseline, absolute values and change from baseline data.

Descriptive summary statistics for baseline, absolute values and change from baseline data to Month 12 and Month 24 will be provided by treatment group for digital visual function.

Additionally, the average digital visual function score over the last 3 months for each reporting period (Month 10 - Month 12 for the Month 12 reporting; Month 22 - Month 24 for the Month 24 reporting) will be calculated for each subject who has at least one digital visual function assessment in these windows. The observed values for the digital visual function and changes from baseline will be summarized by treatment group. Summaries will present the descriptive statistics for baseline, absolute values and change from baseline data.

6.6.5. Genetic Polymorphisms

The genetic polymorphisms data will be analyzed and reported in a separate report and briefly summarized in the clinical study report (CSR). The genetic polymorphisms data will be listed for the genotyping set.

6.6.6. Dry AMD Analysis

Biomarkers of early and intermediate stage of AMD outside the GA area will also be evaluated in this study. The number and percentage of subjects with at least one lesion (up to 5) area in the study eye classified as iRORA will be summarized at baseline by treatment group in the mITT set. The number and percentage of subjects with a progression from iRORA to cRORA based on SD-OCT in the study eye will be summarized over time by treatment group. A CMH test with the number of baseline iRORA lesions (0, 1, 2, 3, 4, ≥ 5), presence of CNV in the fellow eye (yes or no), and baseline GA lesion area (< 7.5 mm^2 or ≥ 7.5 mm^2) as the stratification factors will be presented for each post-baseline visit. If necessary due to sparse cells, an unadjusted analysis may be performed. In addition, the percentage of baseline lesions with a progression will also be presented over time by treatment group.

The above summary and analysis will also be repeated in those subjects with at least one lesion in the study eye classified as large drusen at baseline and whether these progressed to iRORA or cRORA in the study eye. Large drusen is defined as a drusen that is greater than or equal to 40 microns in height on SD-OCT.

This analysis will not be conducted at the Month 12 reporting and will only be conducted for the final study reporting.

All dry AMD data will be listed for the ITT set.

6.6.7. Analyses of Other Ophthalmology Efficacy Imaging Data

In addition to the GA lesion(s) size data from the FAF, analysis of additional efficacy parameters will be performed from this imaging instrument for the mITT set. The following summaries will be performed separately by study eye and by fellow eye and will be presented by treatment group.

- Descriptive summary statistics for baseline, absolute values and change from baseline data for the distance of GA lesion from atrophy junction to the fovea in study eyes with non-subfoveal involvement of the GA lesion at baseline (assessed from FAF). For the fellow eye, this will be presented for fellow-eyes with non-subfoveal involvement of the GA lesion at baseline. Non-subfoveal involvement is defined as distance of the atrophy junction to the fovea > 0.
- Conversion from non-subfoveal involvement to subfoveal involvement of the GA lesion at each visit (assessed from FAF). This will include only study eyes with non-subfoveal involvement for GA lesions at baseline. For the fellow eye, this should be presented for fellow eyes with non-subfoveal involvement at baseline. This will be presented as a cumulative incidence at each scheduled visit.

All FAF data will be listed for the ITT set.

7. SAFETY ANALYSES

Safety analyses will be performed using the Safety set. Safety variables include adverse events (AEs), deaths, clinical laboratory results, vital signs, incidence of ADA against pegcetacoplan, ocular assessments (e.g., NL-BCVA, IOP and ophthalmic examinations), and ocular imaging.

At the time of the Month 12 reporting, safety summaries will be produced based on the complete Month 12 data. At the time of the final study reporting, safety summaries will be produced based on cumulative data.

For each safety variable, the last value collected before the first dose of investigational product will be used as baseline for all analyses of that safety variable. Last Observed Value (LOV) will be defined as the last valid assessment obtained after baseline. The LOV will be presented for the Month 12 reporting as well as the final study reporting.

All safety analyses will be conducted according to the actual treatment the subject received. Unless otherwise specified, all safety summaries will be performed on the Safety set.

7.1. Adverse Events

AEs will be coded using the MedDRA version 23.1 and summarized separately for those occurred in the study eye, those in the fellow eye, and the non-ocular events.

An AE will be considered a TEAE if it has a start date on or after the first dose of investigational product or if it has a start date before the date of the first dose of investigational product but increases in severity on or after the date of the first dose of investigational product.

An overall summary of the number of subjects with TEAEs will be presented, including the number and percentage of subjects with:

- Any TEAEs
- TEAEs related to study drug (evaluated by the investigator as definitely related, possibly related)
- TEAEs related to study drug injection procedure
- All TEAEs by maximum severity
- Serious TEAEs
- Serious TEAEs related to study drug (evaluated by the investigator as definitely related, possibly related)
- Serious TEAEs related to study drug injection procedure
- Serious TEAEs by maximum severity
- TEAEs leading to interruption of study treatment
- TEAEs leading to discontinuation of study treatment
- TEAEs leading to study discontinuation
- TEAEs leading to death

This overall summary will also include the total number of TEAEs reported. This overall summary will also be summarized separately for those occurred in the study eye, those in the fellow eye, and the non-ocular events.

The number and percentage of subjects reporting TEAEs in each treatment group and overall will be tabulated by SOC and PT for all of the categories in the list above. If more than 1 AE occurs with the same preferred term for the same subject, then the subject will be counted only once for that preferred term using the most severe and most related occurrence for the summarization by severity and by relationship to investigational product.

Endophthalmitis and intraocular inflammation ocular TEAEs will be presented by PT for study eye and fellow eye.

Endophthalmitis and intraocular inflammation ocular TEAEs in the study eye will also be presented on a per-injection basis (i.e., number of events/total number of injections given).

All SOC and PT summaries will be ordered alphabetically by SOC and within SOC, by descending order of the total number of subjects in the PM, PEOM, and Sham groups combined.

Non-ocular AEs, ocular AEs in the study eye, and ocular AEs in the fellow eye will be listed separately. This presentation will be repeated for serious AEs. Additionally, separate listings for deaths, AEs leading to discontinuation of study, AEs leading to discontinuation of study drug, and AEs due to COVID-19 will also be generated.

7.1.1. TEAEs of New Onset Exudative AMD

For subjects who experience a TEAE of "Choroidal neovascularization" or "Neovascular AMD" ("Exudative AMD TEAE"), the following summaries will be performed:

- The overall summary table described in Section 7.1 will be repeated replacing "TEAEs" with "Exudative AMD TEAEs". This will be reported for the study eye.
- The incidence of Exudative AMD TEAEs in the study eye will be presented by treatment group and overall. In addition, the analysis will be repeated by fellow eye CNV status at baseline and study eye DLS on SD-OCT at baseline. A similar analysis will be repeated for the Exudative AMD TEAEs in the fellow eye with an additional analysis by fellow eye DLS on SD-OCT at baseline. Subjects with a medical history of CNV in the corresponding eye will be excluded from the analysis.
- The incidence and rate per 100 subject-years of Exudative AMD TEAEs in the study and fellow eye as well as a summary (for study and fellow eye) and Kaplan Meier plot for the time to development of Exudative AMD TEAEs in the study eye will be presented by treatment group and overall. Subjects with a medical history of CNV in the corresponding eye will be excluded from the analysis.
- In addition, the characteristics of the Exudative AMD TEAEs in the study eye will be presented at baseline, study visit preceding exudation, study visit at exudation and at Month 12 (or Month 24 if the final analysis) for characteristics based on SD-OCT and baseline, study visit at exudation and at Month 12 (or Month 24 if the final analysis) for characteristics based on Fluorescein Angiography (FA). The following characteristics will be presented by treatment group and overall:

- o Presence of cystoid spaces on SD-OCT
- o Central (center point) retinal thickness on SD-OCT
- o Central subfield thickness on SD-OCT
- o Presence of CNV on FA.
- o CNV type on FA
- o Presence of subretinal fluid on SD-OCT

- A summary of concomitant anti-VEGF therapy for new exudative AMD in the study eye will be provided by treatment group and overall. This summary will include the total number of subjects that received any anti-VEGF for new exudative AMD as well as the average number of injections per month post exudative AMD. There will also be a breakdown by preferred term of ranibizumab or aflibercept and the average number of injections per month for each term. Subjects will be presented by the number of injections received for that medication.
- BCVA scores at the baseline visit, study visit preceding exudation, the study visit at the exudation, and at Month 12 (or Month 24 if the final analysis) will be presented for all subjects with an Exudative AMD TEAE in the study eye by treatment group and overall. In addition, the change in BCVA scores from baseline, the visit prior to the exudation, and the exudation visit will be presented where appropriate.

A listing of Exudative AMD TEAEs and baseline characteristics including fellow eye CNV status, study eye double layer sign and study eye MNV presence, a listing of the characteristics of the new Exudative AMD TEAEs, anti-VEGF exposure, and the BCVA values for subjects with the new Exudative AMD TEAEs will be provided.

7.2. Clinical Laboratory Data

All laboratory parameters collected at each center's local laboratory will be normalized by converting values in original units to values in SI units and classified as normal, low, or high based on normal ranges supplied by the local laboratories and upon employing standardization.

Observed and change from baseline clinical laboratory data (hematology, chemistry and urinalysis) will be summarized by treatment group and protocol specified time points for the following clinical laboratory variables.

**Hematology**	Hemoglobin, hematocrit, red blood cells (RBC), platelet count, white blood cell count (WBC)– total and differential.
**Biochemistry**	Aspartate transaminase, alanine transaminase, alkaline phosphatase, sodium, potassium, chloride, bicarbonate, blood urea nitrogen (BUN), bilirubin (total, direct and indirect), creatinine, creatine kinase, glucose, and albumin.
**Urinalysis**	Glucose, specific gravity, blood, ketones, protein, bilirubin, pH, urobilinogen, leukocyte esterase, and nitrite.

Clinical laboratory test values are potentially clinically significant (PCS) if they meet either the low or high PCS criteria listed in Table 3. The number and percentage of subjects with baseline and/or post-baseline PCS values will be tabulated by treatment group and overall.
The percentages will be calculated relative to the number of subjects with available values for

each lab parameter in each treatment group at the analysis visit. The numerator is the total number of subjects with at least 1 PCS value at the analysis visit. A supportive listing of subjects with post-baseline PCS values will be provided including the subject number, site, baseline, and post-baseline values.

Table 3: Criteria for Potentially Clinically Significant Laboratory Tests

Parameter	SI Unit	Lower Limit	Higher Limit
**Biochemistry**			
Albumin	g/L	<25 g/L	
Alanine Aminotransferase (ALT)	U/L		≥3*ULN
Alkaline Phosphatase (ALP)	U/L		≥3*ULN
Aspartate Aminotransferase (AST)	U/L		≥3*ULN
Bilirubin, Direct	umol/L		>1.5*ULN
Bilirubin, Total	umol/L		>1.5*ULN
Bicarbonate	mmol/L	<LLN	
Blood Urea nitrogen	mmol/L		>2.5*ULN
Chloride	mmol/L	<90 mmol/L	>115mmol/L
Creatinine	umol/L		≥178.8 umol/L
Creatine kinases	U/L		≥3*ULN
Glucose	mmol/L	<3.05 mmol/L	≥8.88 mmol/L
Potassium	mmol/L	<3.5 mmol/L	>5.5 mmol/L
Sodium	mmol/L	<130 mmol/L	>150 mmol/L
**Hematology**			
Hemoglobin	g/L	<0.9*LLN	
Hematocrit	%	<0.9*LLN	
Platelet Count	10^9/L	<LLN	
**Urinalysis**			
Specific Gravity		<0.9*LLN	>1.1*ULN
pH		<LLN	
Protein			>2+
Glucose			>1+
Blood			>2+

LLN: Lower limit of normal value provided by the laboratory
ULN: Upper limit of normal value provided by the laboratory

Descriptive statistics will be used for continuous data and frequency counts and percentages for categorical data. A shift table from baseline, by treatment group and protocol specified time points, of normal, abnormal low, and abnormal high records will also be summarized for hematology and chemistry data with marginal totals using frequency counts and percentages.

All laboratory data will be listed for the safety set.

7.3. Vital Signs

Descriptive statistics for vital signs (e.g., systolic and diastolic blood pressure, respiratory rate, temperature and pulse rate) and their changes from baseline at each post-baseline visit will be presented by treatment group and overall.

Vital sign values will be considered PCS if they meet both the observed value criteria and the change from baseline criteria listed in Table 4. The number and percentage of subjects with PCS baseline and/or post-baseline values will be tabulated by treatment group and overall. The percentages will be calculated relative to the number of subjects with values for each vital sign parameter in each treatment group at the analysis visit. The numerator is the total number of subjects with at least 1 PCS vital sign value at the analysis visit. A supportive listing of subjects with post-baseline PCS values will be provided including the subject number, site, baseline, and post-baseline PCS values.

Table 4: Criteria for Potentially Clinically Significant Vital Signs

Vital Sign Parameter	Flag	Criteria[a]	
		Observed Value	Change from Baseline
Systolic blood pressure (mmHg)	High	≥180	Increase of ≥20
	Low	≤90	Decrease of ≥20
Diastolic blood pressure (mmHg)	High	≥105	Increase of ≥15
	Low	≤50	Decrease of ≥15
Pulse rate (beats per minute)	High	≥120	Increase of ≥15
	Low	≤50	Decrease of ≥15

[a] A post-baseline value is considered as a PCS value if its meets both criteria for observed value and change from baseline.

All vital signs data will be listed for the safety set.

7.4. Physical Examination Findings

Any relevant findings from the baseline physical examination are to be reported on the medical history. Any relevant findings from the post-baseline physical examination are to be reported as AE's. No separate physical examination findings were collected to summarize.

7.5. Complete Ophthalmic Exam

Results of the ocular assessments will be summarized by shift tables showing the shift from baseline in the individual slit-lamp examination and indirect ophthalmoscopy endpoint. These will be provided by treatment group, overall and visit for study eye and fellow eye. The visits to be presented are Month 12, Month 24, and LOV.

Any relevant findings from the post-baseline ophthalmic examination are to be reported as AEs.

The change from baseline in chronic (first measurement of the day) IOP will be summarized over time by treatment group. In addition, the incidence of IOP above specified thresholds (i.e., >21 mmHg at pre-injection/chronic (first measurement of the day); >30 mmHg post injection) will be summarized by treatment group and overall, protocol specified timepoint (at Month 12 and Month 24 readout, respectively) and over the whole study.

The mean change from baseline in the chronic IOP ± the standard error will be plotted over time by treatment group.

Details of the ocular assessments will be provided in listings for the safety set and for IOP, the ITT set.

7.6. NL-BCVA

The number and percentage of subjects who lost letters compared with baseline at each visit based on NL-BCVA in the study and fellow eye will be presented. The three categories are >=15, ≥15- < 30 and ≥30 ETDRS letters.

7.7. Other Ophthalmology Imaging Assessments

Unless otherwise stated, ocular imaging data collected will be listed by treatment group and overall for the Safety set. The summary will be performed separately by study eye and by fellow eye. The ophthalmology imaging assessments performed in this study include the following:

- Digital Color Fundus Photographs (DCFP)
    - Shift from baseline in the presence/absence of hemorrhage at each visit
- FA
    - The following analyses will be performed in all study eyes and in non-study eyes without a history of CNV at baseline (in medical history) in the corresponding eye.
        - Incidence of new CNV. This will be summarized as the cumulative number of CNV events by each visit and the cumulative incidence by that visit based on Kaplan Meier methods.
        - Incidence of new CNV by type (classic, occult, classic & occult, disciform scar) at first emergence will be summarized at each visit for the cumulative number of events.
        - Total CNV size (diameter) at first emergence will be summarized at each visit for the cumulative number of events.
- SD-OCT
    - Descriptive statistics on central subfield thickness at each visit
    - Plot of the mean change from baseline in central subfield thickness in the study eye ± the standard error over time by treatment group.
    - Descriptive statistics on central (center point) retinal thickness at each visit
    - Shift from baseline in the presence/absence of subretinal fluid at each visit
    - Shift from baseline in the presence/absence of cystoid spaces at each visit
- Specular Microscopy

  - Endothelial Cell Count is assessed using specular microscopy, and the change from baseline in cell density (cells/mm^2) as well as the change from baseline in average cell-size will be summarized with descriptive statistics.
- OCT-A
  - Incidence of subclinical CNV (referred to as MNV in the datasets) determined by OCT-A in eyes without previous CNV reported in the medical history or as an adverse event in the corresponding eye at each visit.

In addition, the following summary of suspected and reading-center confirmed CNV cases will be presented by treatment group. This will be presented separately for the study eye only.

- The number and percentage of subjects with an Exudative AMD TEAE on or before Month 12 (or Month 24 if the final analysis)
- The number and percentage of subjects with at least one suspected new onset CNV case submitted to the reading center by the Investigator post-baseline through Month 12 (or Month 24 if the final analysis)
- The number and percentage of subjects with a reading-center confirmed new onset CNV case (out of the suspected CNV cases submitted by the Investigator) post-baseline through Month 12 (or Month 24 if the final analysis). Reading-center confirmed CNV case is identified as either:
  - Presence of CNV on FA (Type = classic, occult, classic & occult, disciform scar) OR
  - Presence of CNV thickness on SD-OCT
- The number and percentage of subjects with a reading-center confirmed new onset CNV case (out of the suspected CNV cases submitted by the Investigator) post-baseline through Month 12 (or Month 24 if the final analysis) and also reported as an Exudative AMD TEAE.
- The number and percentage of subjects with a reading-center confirmed new onset CNV case (out of the suspected CNV cases submitted by the Investigator) post-baseline through Month 12 (or Month 24 if the final analysis) without an Exudative AMD TEAE ever reported.
- The number and percentage of subjects with at least one suspected CNV case, confirmed by the reading-center but previously reported as an Exudative AMD TEAE.
- The number and percentage of subjects with at least one suspected CNV case, not confirmed by the reading-center but still reported as an Exudative AMD TEAE.
- The number and percentage of subjects with a reading-center finding of presence of new-onset CNV (defined in the same way as a confirmed new onset CNV case) without any other submission to the reading center of a suspected case post-baseline through Month 12 (or Month 24 if the final analysis) and also reported as an Exudative AMD TEAE on or before Month 12 (or Month 24 if the final analysis)

- The number and percentage of subjects with a reading-center finding of presence of new-onset CNV (defined in the same way as a confirmed new onset CNV case) without any other submission to the reading center of a suspected case post-baseline through Month 12 (or Month 24 if the final analysis) and no report of an Exudative AMD TEAE.
- The number and percentage of subjects with Exudative AMD TEAE and without a suspected case submitted to the reading center as well as without a reading center finding of new-onset CNV (defined in the same way as a confirmed new onset CNV case).

7.8. Immunogenicity

Immunogenicity data will be listed separately for anti-pegcetacoplan peptide antibody and anti-PEG antibody results. The number and percentage of samples confirmed positive for ADA response will be summarized by treatment groups (i.e., PM, PEOM, and Sham). The number and percentage of subjects with treatment-emergent and treatment-boosted responses will be presented by treatment group. Treatment emergent response is defined as a confirmed positive antibody result postdose after a negative antibody result reported at baseline. Treatment-boosted response is defined as a ≥4-fold increase in titer from the baseline level.

7.9. Death

Subject deaths and primary cause of death will be summarized.

7.10. Other Safety Data

Urine pregnancy test will be provided in a listing.

8. PHARMACOKINETICS ANALYSIS

All summaries and analyses of the pharmacokinetic data will be based on the Pharmacokinetic set.

Pegcetacoplan concentrations reported as BLQ will be taken as zero for linear plots, and equal to the lower limit of quantification (LLOQ) for semi-logarithmic plots. For the computation of descriptive statistics, BLQ will be taken as zero, except for the calculation of the geometric mean where the LLOQ will be used.

Pegcetacoplan concentrations will be summarized by pegcetacoplan treatment group (i.e., PM and PEOM) at each scheduled time point using descriptive statistics ((including at least Mean, SD, CV, Median, Min, Max, Geometric Mean/%CV).

Linear and log-linear individual concentration profile plots against time will be produced for each pegcetacoplan treatment group. The actual sampling time will be used on the x-axis.

Linear and log-linear median (± standard error) concentration profile plots against time will be produced by treatment group. The nominal sampling time will be used on the x-axis.

Linear and log-linear mean (± standard error) concentration profile plots against time will be produced for each pegcetacoplan treatment group (i.e., PM and PEOM), with nominal sampling time on the x-axis.

The number of subjects with values BLQ will be tabulated.

A listing of serum concentration data will be presented by treatment arm. The actual time, deviation and percent deviation from nominal time will also be listed.

The C_{max} values are obtained from serum samples collected at Day 7 following pegcetacoplan treatment ($C_{Day\ 7}$) and will be listed and summarized as part of the concentration data. The results will be listed and summarized using descriptive statistics. No additional presentations are required for other pharmacokinetic parameters.

Population pharmacokinetic and exposure-response modelling of the safety and efficacy data may be performed using a separate Population Pharmacokinetic/Pharmacodynamic Analysis Plan.

9. PHARMACODYNAMIC ANALYSES

The PD parameters (CH50, AH50, and C3) will be evaluated based on the Pharmacodynamic set. Absolute values, changes from baseline and percentage changes from baseline for the PD parameters will be summarized by treatment group at each protocol specified time point using descriptive statistics.

For each PD parameter, the individual absolute values and individual changes from baseline will be presented graphically for each treatment group. Actual sampling times will be used for the graphical presentation of individual data. If a baseline PD value is zero, then the percentage change from baseline will not be calculated. For the PD plots, a BLQ value will be set equal to LLOQ.

The mean absolute values, mean changes from baseline and mean percentage changes from baseline will also be presented graphically by treatment group. Nominal sampling times will be used for the mean plots.

Individual PD parameters will be listed together with changes from baseline and percentage changes from baseline by treatment group.

10. OTHER ANALYSES

No other analyses are planned for this study.

11. INTERIM ANALYSIS

No formal interim analyses are planned for this study.

To support regulatory submissions, data cuts of the Month 12 – Month 24 data of the study may be taken prior to the final database lock and summarized prior to the final study reporting. The analysis of this data will not be used to modify the pre-planned analyses of this study.

12. DATA MONITORING COMMITTEE

An external, independent DMC reviews unmasked data across the conduct of the study approximately every 6 months. A charter for the DMC as well as a DMC statistical analysis plan was prepared separately.

13. DATA HANDLING CONVENTIONS

13.1. General Data Reporting Conventions

Continuous variables will be summarized using the number of non-missing observations (n), mean, SD, median, quartile 1, quartile 3, minimum, and maximum. Geometric mean and CV will be included for PK parameters, where appropriate. Categorical variables will be summarized using frequencies and percentages. Unless stated otherwise, for all percentages, the number of subjects in the analysis population for the treatment group will be the denominator.

Unless otherwise specified, the estimated mean and median for a set of values should be printed out to 1 more decimal place than the original values, and standard deviations should be printed out to 2 more decimal places than the original values. The minimum and maximum should report the same number of decimal places as the original values. Percentages will be displayed with 1 decimal place; except percentages will not be presented when the count is zero and 100% will be presented as an integer.

13.2. Definition of Baseline

Unless stated otherwise, baseline will be defined as the last available pre-treatment value taken on or before the first dose date of study drug, and will be used for summary of baseline characteristics, as well as for all change-from-baseline analyses of efficacy and safety endpoints.

13.3. Definition of Relative Study Days

Unless otherwise noted, relative study days (Rel Days) of an evaluation are defined as number of days relative to the first dose date of study drug which is designated as Day 1, and the preceding day is Day -1, the day before that is Day -2, etc.

If evaluation date is on or after first dose date, then relative study days are calculated as

Evaluation date - first dose date of study drug + 1.

If evaluation date is before first dose date, then relative study days are calculated as

Evaluation date - first dose date of study drug

Relative study days take negative values if evaluation date occurs prior to first dose date and take positive values if evaluation date occurs on or after first dose date of study drug.

13.4. Definition of Visit Windows

All assessments occurring on or before the first date of dosing (Analysis Study Day (ADY) ≤ 1) will be assigned to the Baseline analysis visit window.

Unless otherwise specified, the actual scheduled nominal post-baseline visit will be used for over time summaries. Post-baseline unscheduled visits and early termination visits will be mapped to a scheduled visit and will only be used in the analysis if the nominal scheduled visit result is missing, Table 5 and Table 6 presents the analysis visit window mapping for unscheduled and early term visits. In the case that multiple unscheduled or early termination visits are in the same analysis window, the one closest to the target date will be used. In the event that windowed visit is mapped to an illogical sequence of visits when considering nearby scheduled visits

(i.e., windowed visit is higher than the subsequent visit or lower than the preceding visit), the windowed visit will be set to the logical scheduled visit.

Table 5: Post-Baseline Analysis Visit Window for Unscheduled and Early Termination Visits: Monthly Regimen

Analysis Visit	Target Study Day	Analysis Window (days)
Day 7	7	2 to 15
Month 1	30	16 to 45
Month 2	60	46 to 75
Month 3	90	76 to 105
Month 4	120	106 to 135
Month 5	150	136 to 165
Month 6	180	166 to 195
Month 7	210	196 to 225
Month 8	240	226 to 255
Month 9	270	256 to 285
Month 10	300	286 to 315
Month 11	330	316 to 345
Month 12	360	346 to 375
Month 13	390	376 to 405
Month 14	420	406 to 435
Month 15	450	436 to 465
Month 16	480	466 to 495
Month 17	510	496 to 525
Month 18	540	526 to 555
Month 19	570	556 to 585
Month 20	600	586 to 615
Month 21	630	616 to 645
Month 22	660	646 to 675
Month 23	690	676 to 705
Month 24	720	706 to EOS

Table 6: Post-Baseline Analysis Visit Window for Unscheduled and Early Termination Visits: Every Other Month Regimen

Analysis Visit	Target Study Day	Analysis Window (days)
Day 7	7	2 to 15
Month 1	30	16 to 45
Month 2	60	46 to 75
Month 3	90	76 to 105
Month 4	120	106 to 135
Month 5	150	136 to 165
Month 6	180	166 to 195
Month 7	210	196 to 225
Month 8	240	226 to 255
Month 9	270	256 to 285
Month 10	300	286 to 315
Month 11	330	316 to 345
Month 12	360	346 to 375
Month 14	420	376 to 450
Month 16	480	451 to 510
Month 18	540	511 to 570
Month 20	600	571 to 630
Month 22	660	631 to 690
Month 24	720	690 to EOS

13.5. Derived Efficacy Endpoints

13.5.1. GA Lesion Size

For all independent ophthalmic assessments (performed by DARC laboratory), which includes GA lesion size, if assessments by only two independent readers are available, then the median of the 2 readings will be used in the calculation of summary statistics. If assessments by three independent readers are available, then the median of the three readings will be used for the summary calculations.

In the case that the GA lesion size has an indeterminate boundary noted, then the lesion size for this indeterminate boundary will be used in all analyses of GA lesion size unless otherwise noted in the case that primary lesions size assessment for an individual reader is missing.

13.5.2. MNRead Reading Chart

13.5.2.1. Reading Speed

13.5.2.1.1. Without adjustment for reading inaccuracy

Any print size with “Not Done” will have the associated wpm marked as 0.

Reading speed (wpm) = 600 / (reading time in seconds)

If reading speed >300 wpm, reading speed = 300.

13.5.2.1.2. With adjustment for reading inaccuracy

Any print size with “Not Done” will have the associated wpm marked as 0.

Reading speed (wpm) = 60 (10 – errors) / (reading time in seconds)

If reading speed >300 wpm, reading speed = 300.

If 10 or more errors were made in a sentence (implying wpm<0), then the reading speed for that sentence can be assumed to be zero (wpm).

13.5.2.2. Maximum reading speed

Maximum reading speed will be calculated as the mean of the three highest nonzero reading speeds (or 2 or 1 if that is all that exists). For subjects having <u>all</u> wpm values = 0, the maximum reading speed will be set to 0, and for those same visits the critical print size will be set to missing. Reading speed will equal 0 wpm at each print size for which (time=0) or Not Done (indicating no words in the sentence could not be read or sentence was not attempted due to vision) is checked for that print size.

13.5.2.3. Critical Print Size

Critical print size will be calculated as the smallest print size which support reading speed at 80% of the maximum reading speed. If all wpm at all print sizes = 0 at a visit then that visit’s critical print size will be missing. Print size will be adjusted for viewing distance as follows:

- MNRead Charts:
  - For all viewing distances:
    - Print size = Print size + correction, where correction=log10 [40 / (viewing distance in cm)], rounded to two decimal places

Note: the CRFs for the MNRead charts have print sizes pre-filled for a 40 cm viewing distance, while the Radner charts have print sizes pre-filled for a 32 cm viewing distance.

13.5.2.4. Reading acuity

Reading acuity will be calculated at each visit as: 1.4 – (# sentences read x 0.1) + (# of words read incorrectly x 0.01). This variable will not be included in the CSR.

13.5.3. Radner Reading Chart

13.5.3.1. Reading Speed

13.5.3.1.1. Without adjustment for reading inaccuracy

Reading Speed (wpm) = 14 / (time in seconds) x 60 = 840 / (time in seconds)

If reading speed >300 wpm, reading speed = 300.

13.5.3.1.2. With adjustment for reading inaccuracy

Reading Speed (wpm) = (14 – errors)/ (time in seconds) x 60

If 14 or more errors were made in a sentence (implying wpm<0), then the reading speed for that sentence can be assumed to be zero (wpm).

If reading speed >300 wpm, reading speed = 300.

13.5.3.2. Maximum Reading Speed

Maximum reading speed will be calculated as the mean of the three highest nonzero reading speeds (or 2 or 1 if that is all that exists). For subjects having all wpm values = 0, the maximum reading speed will be set to 0, and for those same visits the critical print size will be set to missing. Reading speed will equal 0 wpm at each print size for which (time=0) or Not Done (indicating no words in the sentence could not be read or sentence was not attempted due to vision) is checked for that print size.

13.5.3.3. Critical Print Size

Critical print size will be calculated as the smallest print size which support reading speed at 80% of the maximum reading speed. If all wmp at all print sizes = 0 at a visit then that visit's critical print size will be missing. Print size needs to be adjusted for viewing distance as follows:

- Radner Charts:
    - For viewing distance of 32 cm
        - No correction is needed.
    - For viewing distance of other than 32 cm:
        - Print size= Print size – 0.1 + correction, where correction=log10 [40 / (viewing distance in cm)], rounded to two decimal places

Note: the CRFs for the MNRead charts have print sizes pre-filled for a 40 cm viewing distance, while the Radner charts have print sizes pre-filled for a 32 cm viewing distance.

13.5.3.4. Reading Acuity

Reading acuity will be calculated at each visit as: 1.4 – (# sentences read x 0.1) + (# of words read incorrectly x 0.01). This variable will not be included in the CSR.

13.5.4. Mean FRI Index Score and FRI Level

Follow Section 4.3.2 of FRI Index User Manual (Version 1.4).

SAS code for scoring FRI Index and FRI Level is available in Appendix C of FRI Index Under Manual (Version 1.4).

13.5.5. NL-BCVA and LL-BCVA

13.5.5.1. BCVA Letter Score

- If the 4-meter score is >19 letters read correctly, the visual acuity score is the sum of total letters correctly read at 4 meters plus the addition of 30.
- If the 4-meter score is ≤19 letters read correctly, the visual acuity score is the sum of total letters read correctly at 4 meters and total letters read correctly at the 1-meter distance.

- If no letters are read correctly at either the 4-meter distance or the 1-meter distance, the visual acuity score is 0.

13.5.5.2. Conversion of BCVA Letter Score to Snellen Equivalent

Conversions between letter, logMAR, and Snellen visual acuity scores are available in Beck et al. (2003) and Holladay et al. (2004). Letter scores are converted to logMAR equivalents using the formula logMAR = 1.7 – (.02)(letter score). With this conversion, a 5-letter difference in visual acuity is equivalent to a difference of 0.1 logMAR and to one Snellen line. Conversion from logMAR to Snellen can be done after rounding logMAR values to one decimal place following Table 1 in Holladay et al. (2004).

13.5.6. Low Luminance Deficit

LLD = NL-BCVA - LL-BCVA

13.5.7. NEI VFQ-25 scores

Scoring VFQ-25 with or without optional item is a two-step process:

- First, original numeric values from the survey are re-coded following the scoring rules outlined in Table 2 of user's manual. All items are scored so that a high score represents better functioning. Each item is then converted to a 0 to 100 scale so that the lowest and highest possible scores are set at 0 and 100 points, respectively. In this format, scores represent the achieved percentage of the total possible score, e.g., a score of 50 represents 50% of the highest possible score.
- In step 2, items within each sub-scale are averaged together to create the 12 sub-scale scores, Table 3 of user's manual indicates which items contribute to each specific sub-scale. Items that are left blank (missing data) are not taken into account when calculating the scale scores. Sub-scales with at least one item answered can be used to generate a sub-scale score. Hence, scores represent the average for all items in the sub-scale that the respondent answered.

13.5.7.1. NEI VFQ-25 Distance Activity Subscale Score

Average of items 8, 9, and 14

13.5.7.2. NEI VFQ-25 Near Activity Subscale Score

Average of items 5, 6, and 7

13.5.7.3. NEI VFQ-25 Driving Subscale Score

For subjects who are currently driving at baseline only. Average of items 15c, 16, 16a

13.5.7.4. NEI VFQ-25 Composite Scores

To calculate an overall composite score for the VFQ-25, simply average the vision-targeted sub-scale scores, excluding the general health rating question.

13.5.8. NEI VFQ-39 Scores

For subjects responded to additional questions A1-A13, VFQ-39 subscale scores can be calculated using Table 4 of user's manual.

13.5.8.1. NEI VFQ-39 Distance Activity Subscale Score

Average of items 8, 9, 14, A6, A7, and A8

13.5.8.2. NEI VFQ-39 Near Activity Subscale Score

Average of items 5, 6, 7, A3, A4, and A5

13.5.8.3. NEI VFQ-39 Composite score

Calculated by simply averaging the vision related subscales, excluding the general health rating question.

13.6. Repeated or Unscheduled Assessments of Safety Parameters

If a subject has repeated assessments before the start of investigational product, then the results from the final assessment made prior to the start of investigational product will be used as baseline. If end-of-study assessments are repeated or unscheduled, the last post-baseline assessment will be used as the end of study assessment for generating descriptive statistics. However, all post-baseline assessments will be used for PCS value determination and all assessments will be presented in the data listings.

13.7. Handling of Missing, Unused, and Spurious Data

13.7.1. Missing Date of Investigational Product

When the date of the last dose of investigational product is missing for a subject in the Safety set, all efforts should be made to obtain the date from the investigator.

13.7.2. Missing Date Information for Prior or Concomitant Medications (Therapies/Procedures)

If either the start or stop date of medication is missing, the worst or most conservative case will be considered when assigning medications to categories. So, for a missing start date (where stop date is after date of first dose or missing) the date will be imputed as the date of first dose; for a missing stop date the date will be imputed as the last study date.

13.7.2.1. Incomplete Start Date

The following rules will be applied to impute the missing numerical fields. If the stop date is complete and the imputed start date is after the stop date, then the start date will be imputed using the stop date.

13.7.2.1.1. Missing Day and Month

If the year of the incomplete start date is the same as the year of the date of the first dose of investigational product, then the day and month of the date of the first dose of investigational product will be assigned to the missing fields.

If the year of the incomplete start date is before the year of the date of the first dose of investigational product, then December 31 will be assigned to the missing fields.

If the year of the incomplete start date is after the year of the date of the first dose of investigational product, then 01 January will be assigned to the missing fields.

13.7.2.1.2. Missing Month Only

The day will be treated as missing and both month and day will be replaced according to the above procedure.

13.7.2.1.3. Missing Day Only

If the month and year of the incomplete start date are the same as the month and year of the date of the first dose of investigational product, then the day of the date of the first dose of investigational product will be assigned to the missing day.

If either the year is before the year of the date of the first dose of investigational product or if both years are the same but the month is before the month of the date of the first dose of investigational product, then the last day of the month will be assigned to the missing day.

If either the year is after the year of the date of the first dose of investigational product or if both years are the same but the month is after the month of the date of the first dose of investigational product, then the first day of the month will be assigned to the missing day.

13.7.2.2. Incomplete Stop Date

The following rules will be applied to impute the missing numerical fields. If the imputed stop date is before the start date (imputed or non-imputed start date), then the imputed stop date will be equal to the start date.

13.7.2.2.1. Missing Day and Month

If the year of the incomplete stop date is the same as the year as of the last study date, then the day and month of the date of the last study date will be assigned to the missing fields.

If the year of the incomplete stop date is before the year of the last study date, then 31 December will be assigned to the missing fields.

If the year of the incomplete stop date is after the year of the last study date, then 01 January will be assigned to the missing fields.

13.7.2.2.2. Missing Month Only

The day will be treated as missing and both month and day will be replaced according to the above procedure.

13.7.2.2.3. Missing Day Only

If the month and year of the incomplete stop date are the same as the month and year of the last study date, then the day of the last study date will be assigned to the missing day

If either the year is before the year of the last study date or if both years are the same but the month is before the month of the last study date, then the last day of the month will be assigned to the missing day

If either the year is after the year of the last study date or if both years are the same but the month is after the month of the last study date, then the first day of the month will be assigned to the missing day.

13.7.3. Missing Date Information for Adverse Events

Events with missing or partial dates will be handled such that in the absence of contradictory information an AE is treatment emergent. So, for a missing start date (where stop date is after first dosing date or missing) the date will be imputed as the first dose date; for a missing stop date the date will be imputed as the last study date. If a partial date is recorded, the following convention will be used to assign the AE.

13.7.3.1. Incomplete Start Date

If a start date is missing the day information and month/year is the same as first dose date then use first dose date, else ‘01’ will be used for the day; if a start date is missing the month and the year is the same as first dose date then use first dose date, else January will be used for the start month.

If AE end date is earlier than treatment start date (i.e., could be inferred by any combination of year/month/day), then any missing part of AE start date will be imputed based on the AE end date.

13.7.3.2. Incomplete Stop Date

If a stop date is missing the day information and month/year is same as last study date then use last study date, else last day of the given month will be used for the stop day; if a stop date is missing the month and year is the same as last study date then use last study date, else December will be used for the stop month.

13.7.4. Missing Severity Assessment for Adverse Events

If severity is missing for an AE starting prior to the date of the first dose of investigational product, then a severity of “Mild” will be assigned. If the severity is missing for an AE starting on or after the date of the first dose of investigational product, then a severity of “Severe” will be assigned. The imputed values for severity assessment will be used for incidence summaries, while the actual values will be used in data listings.

13.7.5. Missing Relationship to Investigational Product for Adverse Events

If the relationship to investigational product is missing for an AE starting on or after the date of the first dose of investigational product, a causality of “Related” to the investigational product will be assigned. The imputed values for relationship to double-blind investigational product will be used for incidence summaries, while both the actual and the imputed values will be presented in data listings.

13.7.6. Character Values of Clinical Laboratory Variables

If the reported value of a clinical laboratory variable cannot be used in a statistical analysis (e.g., a character string is reported for a numerical variable), the appropriately determined coded value will be used in the statistical analysis, several examples are shown in Table 7 for an illustration purpose. The actual values as reported in the database will be presented in data listings.

Table 7: Examples for Coding of Special Character Values for Clinical Laboratory Variables

Clinical Laboratory Test	Possible Results (in SI units)	Coded Value for Analysis
Chemistry: Bilirubin, Total/Direct	<1.7	0.85
	<2	1
Urinalysis: Specific Gravity	≤1.005	0.5025
	>1.030	1.030
Urinalysis: Glucose	≥55	Positive
	≤0	Negative
Urinalysis: pH	≥8.0	8.0
Urinalysis: Protein	≥500	500
Other: FSH	<0.2	0.1

14. ANALYSIS SOFTWARE

Statistical analyses will be performed using Version 9.4 (or newer) of SAS® on a suitably qualified environment.

15. CHANGES TO ANALYSIS SPECIFIED IN PROTOCOL

The following changes were made to the analyses specified in the protocol:

- The mITT set, which includes subjects with a baseline and at least one post-baseline GA lesion assessment, replaced the ITT set as the main efficacy population to be aligned with the primary analysis methodology.
- The change in GA lesion(s) size at Month 24 between PM and Sham was to be the third hypothesis tested in the hypotheses testing strategy as specified in the protocol. This was removed from the hypotheses testing strategy in the SAP.
- The hypotheses testing strategy and α allocation for the key secondary endpoints was not precisely specified within the protocol and has been clarified to follow the hypotheses testing procedure and α allocation as described in Section 6.3.1.
- Several new exploratory endpoints not specified in the protocol were added.
    - NEI-VFQ-39 distance activity, composite, near activity subscale score
    - NEI-VFQ-25 driving subscale score
    - Progression from iRORA to cRORA
    - Progression from large drusen to iRORA or cRORA
    - Change in the distance of the atrophy junction to the fovea and without subfoveal atrophy to subfoveal atrophy conversion
- No pre-specified analysis will be performed on the secondary endpoint of change from baseline in LLD over time. This data will only be listed.
- No formal interim analyses are planned for this study: however, analyses of interim data from Months 12 to 24 that may support regulatory submissions may be conducted.
- The pharmacokinetic endpoints of AUC, C_{max}, and T_{max} were specified in the protocol; however, since only trough concentrations were collected throughout the study it is only possible to assess C_{max}. C_{max} should occur at Day 7 following pegcetacoplan treatment. Therefore, the concentration on Day 7 will be used ($C_{Day\ 7}$) as the observed C_{max} values.
- Other minor editorial changes were made to provide clarity.

16. REFERENCES

Baskaran K, Macedo AF, and He Y, et al. Scoring reading parameters: An inter-rater reliability study using the MNREAD, *PloSONE* (2019) 14(6): e0216775

Beck RW, Moke PS, and Turpin AH et al. A Computerized Method of Visual Acuity Testing: Adaptation of the Early Treatment of Diabetic Retinopathy Study Testing Protocol, *American Journal of Ophthalmology* (2003) 135 (2):194-205

Bretz, F, Maurer W, Brannath W, Posch M. A Graphical Approach to Sequentially Rejective Multiple Test Procedures, *Statistics in Medicine* (2009) 28.4:586-604.

Calabrese A, Cheong AMY, Cheung S-H, et al. Baseline MNREAD measures for normally sighted subjects from childhood to old age. *Invest Ophthalmol Vis Sci* (2016) 57(8):3836-3843.

Diggle, P.J., Liang, K.-Y., and Zeger, S. L. (1994). Analysis of Longitudinal Data. Oxford: Clarendon Press.

Holekamp N, Wykoff CC, and Schmitz-Valckenberg S et al., Natural History of Geographic Atrophy Secondary to Age-Related Macular Degeneration: Results from the Prospective Proxima A and B Clinical Trials, *Ophthalmology* (2020), doi: https://doi.org/10.1016/j.ophtha.2019.12.009.

Holladay J. Visual Acuity Measurements. *Journal of Cataract & Refractive Surgery* (2004) 30(2):287-290.

Li, K. H. Imputation Using Markov Chains. *Journal of Statistical Computation and Simulation* (1988) 30:57-79.

Liu, C. Bartlett's Decomposition of the Posterior Distribution of the Covariance for Normal Monotone Ignorable Missing Data. *Journal of Multivariate Analysis* (1993) 46:198-206

Patel P, Chen F, Da Cruz L, et al. Test-retest variability of reading performance metrics using MNREAD in patients with age-related macular degeneration. *Invest Ophthalmol Vis Sci* (2011) 52(6): 3854-9. doi: 10.1167/iovs.10-6601.

Radner W and Diendorfer G. English sentence optotypes for measuring reading acuity and speed - The English version of the Radner Reading Charts. *Graefes Arch Clin Exp Ophthalmol* (2014) 252:1297-1303.

Radner W, Obermayer W, Richter-Meuksch S, et al. The validity and reliability of short German sentences for measuring reading speed. *Graefes Arch Clin Exp Ophthalmol* (2002) 240:461-367.

17. APPENDIX

APPENDIX 1. SCHEDULE OF ACTIVITIES

Table 8: Visit Schedule - Monthly Group- Screening, Day 1 through Month 12

	**Screening**	**Treatment**														**Early Term[A]**
**Visit #**	**1**	**2**	**2A**	**3**	**4**	**5**	**6**	**7**	**8**	**9**	**10**	**11**	**12**	**13**	**14**	
**Day**	**–28 to –1**	**1**	**7**	**30**	**60**	**90**	**120**	**150**	**180**	**210**	**240**	**270**	**300**	**330**	**360**	
**Week**	**0**	**0**	**1**	**4**	**8**	**12**	**16**	**20**	**24**	**28**	**32**	**36**	**40**	**44**	**48**	
**Month**	**0**	**0**	**0**	**1**	**2**	**3**	**4**	**5**	**6**	**7**	**8**	**9**	**10**	**11**	**12**	
**Window (+ or – days)**	**2**	**0**	**1**	**8**	**8**	**8**	**8**	**8**	**8**	**8**	**8**	**8**	**8**	**8**	**8**	
Informed Consent/Assign Screening Number	x															
Demographic Data	x															
Inclusion/Exclusion Criteria [B]	x	x														
Medical/Surgical/Ocular History [C]	x															
Blood Draw—Safety Labs [D,E,F]	x	x			x				x						x	x
Urine Sample Collection [D, E,F]	x	x			x				x						x	x
Urine Pregnancy Test [D,E,F]		x		x	x	x	x	x	x	x	x	x	x	x	x	
Blood Draw—PK and Complement Profile (C3, CH50, AH50) [D, S]		x	x	x					x						x	x
Blood Draw—Genotyping (if applicable) [D]					x											
Blood Draw—Anti-Pegcetacoplan Ab [D]		x		x	x				x						x	x
Blood Draw for Clinical Repository (if applicable)[D,G]					x				x						x	x
Vital Signs [H]	x	x		x	x	x	x	x	x	x	x	x	x	x	x	x
Physical Examination [I]	x														x	x
BCVA [J]	x	x		x	x	x	x	x	x	x	x	x	x	x	x	x
LL-BCVA [J]		x		x	x	x	x	x	x	x	x	x	x	x	x	x
MNREAD or Radner Reading Charts (select countries) [J,K]		x							x						x	x
Slitlamp Examination	x	x		x	x	x	x	x	x	x	x	x	x	x	x	x

Table 8: Visit Schedule - Monthly Group- Screening, Day 1 through Month 12

	Screening	Treatment														Early Term[A]
**Visit #**	**1**	**2**	**2A**	**3**	**4**	**5**	**6**	**7**	**8**	**9**	**10**	**11**	**12**	**13**	**14**	
**Day**	**–28 to –1**	**1**	**7**	**30**	**60**	**90**	**120**	**150**	**180**	**210**	**240**	**270**	**300**	**330**	**360**	
**Week**	**0**	**0**	**1**	**4**	**8**	**12**	**16**	**20**	**24**	**28**	**32**	**36**	**40**	**44**	**48**	
**Month**	**0**	**0**	**0**	**1**	**2**	**3**	**4**	**5**	**6**	**7**	**8**	**9**	**10**	**11**	**12**	
**Window (+ or – days)**	**2**	**0**	**1**	**8**	**8**	**8**	**8**	**8**	**8**	**8**	**8**	**8**	**8**	**8**	**8**	
Endothelial Cell Count[S]		x							x						x	x
NEI VFQ-25 [L]		x							x						x	x
FRI [L]		x							x						x	x
Home-Based Digital Applications [L,M S]		x		x	x	x			x						x	
Dilated Indirect Ophthalmoscopy	x	x		x	x	x	x	x	x	x	x	x	x	x	x	x
IOP Measurement	x	x		x	x	x	x	x	x	x	x	x	x	x	x	x
SD-OCT [N]	x	x		x	x	x	x	x	x	x	x	x	x	x	x	x
FAF [N]	x	x			SE		SE		x		SE		SE		x	x
NIR [N]	x	x			SE		SE		x		SE		SE		x	x
DCFP [N]	x														x	x
FFA [N]	x														x	x
OCT-A[R,S]		x[R]							x[R]						x[R]	x
Study Eye Determination	x															
Randomization		x														
Pegcetacoplan administration or Sham Injection[T]		x		x	x	x	x	x	x	x	x	x	x	x	x	
Postinjection Assessment [O]		x		x	x	x	x	x	x	x	x	x	x	x	x	
Follow-Up Call [P]		x		x	x	x										
Concomitant Medication/Concomitant Ocular Procedures [Q]	x	x	x	x	x	x	x	x	x	x	x	x	x	x	x	x
Adverse Events	x	x	x	x	x	x	x	x	x	x	x	x	x	x	x	x

Abbreviations: AH50 = alternative pathway of complement functional test; BCVA = best corrected visual acuity; CH50 = classical pathway of complement functional test; CNV = choroidal neovascularization; DCFP = digital color fundus photography; eCRF = electronic case report form; FAF = fundus autofluorescence; FFA = fundus fluorescein angiography; FRI = Functional Reading Independence Index; IOP = intraocular pressure; LL-BCVA = low luminance best corrected visual acuity; MNREAD = Minnesota Low-Vision Reading Test; NEI VFG-25 = National Eye Institute Visual Functioning Questionnaire 25-Item Version; NIR = near infrared reflectance; OCT-A = optical coherence tomography angiography; PK = pharmacokinetics; SD-OCT = spectral domain optical coherence tomography; SE = study eye; Term = termination; VEGF = vascular endothelial growth factor.

Note: All ocular assessments are to be performed for both eyes unless annotated with 'SE' (study eye) in the above schedule. All assessments should be performed on the same day. All study visits should be scheduled and projected based on the Day 1 visit date with the exception of Visit 2a which should be based on the Day 1 dose date.

A. For subjects that discontinue the study early, the early termination assessments should be performed after a minimum of 30 days have passed from the last dosing visit. If a subject reports for a scheduled visit and decides to terminate early prior to dosing, the visit should be considered the early termination visit and all early termination procedures should be performed. At Month 24, all subjects should be offered entry into an open-label study.

B. At Day 1 (Visit 2), confirm subject eligibility through reviewing the inclusion/exclusion criteria and receive confirmation of eligibility from the reading center.

C. Significant medical/surgical history from the previous 5 years. Anti-VEGF treatments (fellow eye) and invasive ocular procedures performed within the past 5 years and while on study should also be recorded. Any history of tobacco use should be recorded.

D. Obtain prior to fluorescein angiography and before study drug administration.

E. At screening, serum pregnancy should be performed for women of childbearing potential. If positive, subject is not eligible to continue in the study.

F. Beginning at Day 1, perform the urine pregnancy test for women of childbearing potential at each treatment visit. If positive, perform a serum pregnancy test. If serum test is positive, study drug should not be administered and an early term visit should be completed.

G. Only subjects that sign the separate consent for the clinical repository and genotyping will have these samples collected. A 14-mL whole-blood sample will be collected at each of the specified visits.

H. Blood pressure, respiratory rate, heart rate, and temperature. On dosing days, vital signs should be taken predose.

I. Height and weight should be collected at screening.

J. Perform assessments prior to dilating the eyes.

K. In select countries, the MNREAD or Radner Reading Charts should be done during the study visit, prior to dilating the eyes. It should be performed monocularly first, then binocularly.

L. To be administered by the masked site staff prior to any other assessments performed on that day. In-clinic assessments on the digital application should be completed after completion of all functional tests and quality of life measures prior to dilating the eyes.

M. At select sites, and for those subjects who decide to participate, the digital applications will be completed on an electronic device and will consist of assessments for visual function and reading speed. Subjects will receive training at Day 1 on the use of the digital applications and the electronic device and will perform the tests using the application in the clinic at Month 1, Month 2, Month 3, Month 6, Month 12, Month 18, and Month 24. Beginning at Day 1, subjects will complete the functional assessments weekly at home. Subjects should be instructed to complete the assessments on the same day each week and at approximately the same time of day each week, if possible.

N. FAF and SD-OCT images, near infrared reflectance, fluorescein angiograms, and fundus photographs will be performed for the study eye only on days where 'SE' is specified and for both eyes at all other visits as specified in the above schedule and will be sent to the reading center for evaluation (consult the reading center manual for specifics on image capture, processing, and transmission). Images should be captured prior to dosing on dosing days. If a subject misses a study visit or images cannot be obtained at a specific visit, study staff should make every effort to obtain images at the next scheduled visit. If new active CNV is suspected, SD-OCT, FFA, and OCT-A (select sites) images must be collected and sent to the reading center for analysis.

O. Postinjection assessments should be performed within 5 minutes after dosing by the unmasked physician or study staff and should include a gross assessment of vision (finger-counting, hand motion, then light perception when applicable). If subject passes gross vision test, the subject may leave the site. If subject fails gross vision test, the tonometry should be performed. IOP should be ≤30 mm Hg in order for the subject to leave the site. If necessary, antiglaucomatous medication can be given to lower the IOP. If IOP is >30 mm Hg, assessments will continue every approximately 30 minutes from the previous measurement until the subject passes gross vision test and IOP is ≤30 mm Hg. Note: if the study eye is treated with a ranibizumab or aflibercept injection during the same visit as the study treatment (pegcetacoplan or sham), the treatment with ranibizumab or aflibercept must be performed first. The pre- and post- anti-VEGF IOP values must be measured and recorded on the eCRF.

P. Starting at Day 1, study subjects will be contacted by study site staff within 4 ± 2 days after each study treatment visit (through Month 3) to collect any information on any safety concerns, decrease in vision, eye pain, unusual ocular events, or any new ocular symptoms in the study eye. If the physician determines that there are any safety concerns, a follow-up visit should be scheduled as soon as possible.

Q. Record concomitant medications (ie, prescription and over-the-counter medications) used by the patient within 30 days of screening and throughout the subject's participation in the study.

R. In addition to the time points indicated on the study schedule, OCT-A should be performed at the time of any suspected new active CNV. If new active CNV is confirmed in the study eye, OCT-A should be repeated every 2 months for the study eye.

S. At select sites only.

T. Administration of study treatment (pegcetacoplan or sham) can be done on a separate day from the assessment visit if both days fall within the visit window. If this occurs on the randomization visit, then the administration of pegcetacoplan or sham should be done within 3 days of randomization and after approval from the medical monitor. When study treatment administration is on a day other than a study visit, then the only assessment that must be done on the day of study treatment administration is the preinjection IOP.

Table 9: Visit Schedule - Monthly Group- Month 13 to Month 24

	Treatment												Early Term [A]
**Visit #**	**15**	**16**	**17**	**18**	**19**	**20**	**21**	**22**	**23**	**24**	**25**	**26**	
**Day**	**390**	**420**	**450**	**480**	**510**	**540**	**570**	**600**	**630**	**660**	**690**	**720**	
**Week**	**52**	**56**	**60**	**64**	**68**	**72**	**76**	**80**	**84**	**88**	**92**	**96**	
**Month**	**13**	**14**	**15**	**16**	**17**	**18**	**19**	**20**	**21**	**22**	**23**	**24**	
**Window (+ or – days)**	**8**	**8**	**8**	**8**	**8**	**8**	**8**	**8**	**8**	**8**	**8**	**8**	
Informed Consent/Assign Screening Number													
Demographic Data													
Inclusion/Exclusion Criteria [B]													
Medical/Surgical/Ocular History [C]													
Blood Draw—Safety Labs [D,E,F]						x						x	x
Urine Sample Collection [D,E,F]						x						x	x
Urine Pregnancy Test [D,E,F]	x	x	x	x	x	x	x	x	x	x	x	x	
Blood Draw—PK and Complement Profile (C3, CH50, AH50)[D,S]												x	x
Blood Draw—Genotyping (if applicable) [D]													
Blood Draw—Anti-Pegcetacoplan Ab [D]		x				x						x	x
Blood Draw for Clinical Repository (if applicable) [D,G]												x	x
Vital Signs [H]	x	x	x	x	x	x	x	x	x	x	x	x	x
Physical Examination [I]												x	x
BCVA [J]	x	x	x	x	x	x	x	x	x	x	x	x	x
LL-BCVA [I]	x	x	x	x	x	x	x	x	x	x	x	x	x
MNREAD or Radner Reading Charts (select countries) [J,K]						x						x	x
Slitlamp Examination	x	x	x	x	x	x	x	x	x	x	x	x	x
Endothelial Cell Count[S]												X	X
NEI VFQ-25 [L]						x						x	x
FRI [L]						x						x	x
Dilated Indirect Ophthalmoscopy	x	x	x	x	x	x	x	x	x	x	x	x	x
Home-Based Digital Applications [L,M,S]						x						x	

Table 9: Visit Schedule - Monthly Group- Month 13 to Month 24

	Treatment												Early Term [A]
**Visit #**	**15**	**16**	**17**	**18**	**19**	**20**	**21**	**22**	**23**	**24**	**25**	**26**	
**Day**	**390**	**420**	**450**	**480**	**510**	**540**	**570**	**600**	**630**	**660**	**690**	**720**	
**Week**	**52**	**56**	**60**	**64**	**68**	**72**	**76**	**80**	**84**	**88**	**92**	**96**	
**Month**	**13**	**14**	**15**	**16**	**17**	**18**	**19**	**20**	**21**	**22**	**23**	**24**	
**Window (+ or – days)**	**8**	**8**	**8**	**8**	**8**	**8**	**8**	**8**	**8**	**8**	**8**	**8**	
IOP Measurement	x	x	x	x	x	x	x	x	x	x	x	x	x
SD-OCT [N]	x	x	x	x	x	x	x	x	x	x	x	x	x
FAF [N]		SE		SE		x		SE		SE		x	x
NIR [N]		SE		SE		x		SE		SE		x	x
DCFP [N]												x	x
FFA [N]												x	x
OCT-A [R,S]						x [R]						x [R]	x
Study Eye Determination													
Randomization													
Pegcetacoplan administration or Sham Injection [T]	x	x	x	x	x	x	x	x	x	x	x		
Postinjection Assessment [O]	x	x	x	x	x	x	x	x	x	x	x		
Follow-Up Call [P]													
Concomitant Medication/Concomitant Ocular Procedures [Q]	x	x	x	x	x	x	x	x	x	x	x	x	x
Adverse Events	x	x	x	x	x	x	x	x	x	x	x	x	x

Abbreviations: AH50 = alternative pathway of complement functional test; BCVA = best corrected visual acuity; CH50 = classical pathway of complement functional test; CNV = choroidal neovascularization; DCFP = digital color fundus photography; eCRF = electronic case report form; FAF = fundus autofluorescence; FFA = fundus fluorescein angiography; FRI = Functional Reading Independence Index; IOP = intraocular pressure; LL-BCVA = low luminance best corrected visual acuity; MNREAD = Minnesota Low-Vision Reading Test; NEI VFG-25 = National Eye Institute Visual Functioning Questionnaire 25-Item Version; NIR = near infrared reflectance; OCT-A = optical coherence tomography angiography; PK = pharmacokinetics; SD-OCT = spectral domain optical coherence tomography; SE = study eye; Term = termination; VEGF = vascular endothelial growth factor.

Note: All ocular assessments are to be performed for both eyes unless annotated with 'SE' (study eye) in the above schedule. All assessments should be performed on the same day. All study visits should be scheduled and projected based on the Day 1 visit date with the exception of Visit 2a which should be based on the Day 1 dose date.

A. For subjects that discontinue the study early, the early termination assessments should be performed after a minimum of 30 days have passed from the last dosing visit. If a subject reports for a scheduled visit and decides to terminate early prior to dosing, the visit should be considered the early termination visit and all early termination procedures should be performed. At Month 24, all subjects should be offered entry into an open-label study.

B. At Day 1 (Visit 2), confirm subject eligibility through reviewing the inclusion/exclusion criteria and receive confirmation of eligibility from the reading center.

C. Significant medical/surgical history from the previous 5 years. Anti-VEGF treatments (fellow eye) and invasive ocular procedures performed within the past 5 years and while on study should also be recorded. Any history of tobacco use should be recorded.
D**.** Obtain prior to fluorescein angiography and before study drug administration.
E. At screening, serum pregnancy should be performed for women of childbearing potential. If positive, subject is not eligible to continue in the study.
F. Beginning at Day 1, perform the urine pregnancy test for women of childbearing potential at each treatment visit. If positive, perform a serum pregnancy test. If serum test is positive, study drug should not be administered and an early term visit should be completed.
G. Only subjects that sign the separate consent for the clinical repository and genotyping will have these samples collected. A 14-mL whole-blood sample will be collected at each of the specified visits.
H. Blood pressure, respiratory rate, heart rate, and temperature. On dosing days, vital signs should be taken predose.
I. Height and weight should be measured at screening.
J. Perform assessments prior to dilating the eyes.
K. In select countries, the MNREAD or Radner Reading Charts should be done during the study visit, prior to dilating the eyes. It should be performed monocularly first, then binocularly.
L. To be administered by the masked site staff prior to any other assessments performed on that day. In-clinic assessments on the digital application should be completed after completion of all functional tests and quality of life measures prior to dilating the eyes.
M. At select sites, and for those subjects who decide to participate, the digital applications will be completed on an electronic device and will consist of assessments for visual function and reading speed. Subjects will receive training at Day 1 on the use of the digital applications and the electronic device and will perform the tests using the application in the clinic at Month 1, Month 2, Month 3, Month 6, Month 12, Month 18, and Month 24. Beginning at Day 1, subjects will complete the functional assessments weekly at home. Subjects should be instructed to complete the assessments on the same day each week and at approximately the same time of day each week, if possible.
N. FAF and SD-OCT images, near infrared reflectance, fluorescein angiograms, and fundus photographs will be performed for the study eye only on days where 'SE' is specified and for both eyes at all other visits as specified in the above schedule and will be sent to the reading center for evaluation (consult the reading center manual for specifics on image capture, processing, and transmission). Images should be captured prior to dosing on dosing days. If a subject misses a study visit or images cannot be obtained at a specific visit, study staff should make every effort to obtain images at the next scheduled visit. If new active CNV is suspected, SD-OCT, FFA, and OCT-A (selected sites) images should be collected and sent to the reading center for analysis.
O. Postinjection assessments should be performed within 5 minutes after dosing by the unmasked physician or study staff and should include a gross assessment of vision (finger-counting, hand motion, then light perception when applicable). If subject passes gross vision test, the subject may leave the site. If subject fails gross vision test, the tonometry should be performed. IOP should be $\leq$30 mm Hg in order for the subject to leave the site. If necessary, antiglaucomatous medication can be given to lower the IOP. If IOP is >30 mm Hg, assessments will continue every approximately 30 minutes from the previous measurement until the subject passes gross vision test and IOP is $\leq$30 mm Hg. Note: if the study eye is treated with a ranibizumab or aflibercept injection during the same visit as the study treatment (pegcetacoplan or sham), the treatment with ranibizumab or aflibercept must be performed first. The pre- and post- anti-VEGF IOP values must be measured and recorded on the eCRF.
P. Starting at Day 1, study subjects will be contacted by study site staff within 4 ± 2 days after each study treatment visit (through Month 3) to collect any information on any safety concerns, decrease in vision, eye pain, unusual ocular events, or any new ocular symptoms in the study eye. If the physician determines that there are any safety concerns, a follow-up visit should be scheduled as soon as possible.
Q. Record concomitant medications (ie, prescription and over-the-counter medications) used by the patient within 30 days of screening and throughout the subject's participation in the study.
R**.** In addition to the time points indicated on the study schedule, OCT-A should be performed at the time of any suspected new active CNV. If new active CNV is confirmed in the study eye, OCT-A should be repeated every 2 months for the study eye.
S. At select sites only.
T. Administration of study treatment (pegcetacoplan or sham) can be done on a separate day from the assessment visit if both days fall within the visit window. If this occurs on the randomization visit, then the administration of pegcetacoplan or sham should be done within 3 days of randomization and after approval from the medical monitor. When study treatment administration is on a day other than a study visit, then the only assessment that must be done on the day of study treatment administration is the preinjection IOP.

Table 10: Visit Schedule - Every-Other-Month Group Screening, Day 1 Through Month 12

	Screening	Treatment														Early Term[A]
**Visit #**	**1**	**2**	**2A**	**3**	**4**	**5**	**6**	**7**	**8**	**9**	**10**	**11**	**12**	**13**	**14**	
**Day**	**–28 to –1**	**1**	**7**	**30**	**60**	**90**	**120**	**150**	**180**	**210**	**240**	**270**	**300**	**330**	**360**	
**Week**	**0**	**0**	**1**	**4**	**8**	**12**	**16**	**20**	**24**	**28**	**32**	**36**	**40**	**44**	**48**	
**Month**	**0**	**0**	**0**	**1**	**2**	**3**	**4**	**5**	**6**	**7**	**8**	**9**	**10**	**11**	**12**	
**Window (+ or – days)**	**2**	**0**	**1**	**8**	**8**	**8**	**8**	**8**	**8**	**8**	**8**	**8**	**8**	**8**	**8**	
Informed Consent/Assign Screening Number	x															
Demographic Data	x															
Inclusion/Exclusion Criteria [B]	x	x														
Medical/Surgical/Ocular History [C]	x															
Blood Draw—Safety Labs [D,E,F]	x	x			x				x						x	x
Urine Sample Collection [D,E,F]	x	x			x				x						x	x
Urine Pregnancy Test [D,E,F]		x			x		x		x		x		x		x	
Blood Draw—PK and Complement Profile (C3, CH50, AH50) [D,S]		x	x	x					x						x	x
Blood Draw—Genotyping (if applicable) [D]					x											
Blood Draw- Anti-Pegcetacoplan Ab [D]		x		x	x				x						x	x
Blood Draw for Clinical Repository (if applicable) [D,G]					x				x						x	x
Vital Signs [H]	x	x		x	x	x	x	x	x	x	x	x	x	x	x	x
Physical Examination [I]	x														x	x
BCVA [J]	x	x		x	x	x	x	x	x	x	x	x	x	x	x	x
LL-BCVA [J]		x		x	x	x	x	x	x	x	x	x	x	x	x	x
MNREAD or Radner Reading Charts (select countries) [J,K]		x							x						x	x
Slitlamp Examination	x	x		x	x	x	x	x	x	x	x	x	x	x	x	x
Endothelial Cell Count[S]		x							x						x	x
NEI VFQ-25 [L]		x							x						x	x
FRI [L]		x							x						x	x
Home-Based Digital Applications [L,M,S]		x		x	x	x			x						x	
Dilated Indirect Ophthalmoscopy	x	x		x	x	x	x	x	x	x	x	x	x	x	x	x
IOP Measurement	x	x		x	x	x	x	x	x	x	x	x	x	x	x	x

Table 10: Visit Schedule - Every-Other-Month Group Screening, Day 1 Through Month 12

	Screening	Treatment														
**Visit #**	**1**	**2**	**2A**	**3**	**4**	**5**	**6**	**7**	**8**	**9**	**10**	**11**	**12**	**13**	**14**	**Early Term[A]**
**Day**	**–28 to –1**	**1**	**7**	**30**	**60**	**90**	**120**	**150**	**180**	**210**	**240**	**270**	**300**	**330**	**360**	
**Week**	**0**	**0**	**1**	**4**	**8**	**12**	**16**	**20**	**24**	**28**	**32**	**36**	**40**	**44**	**48**	
**Month**	**0**	**0**	**0**	**1**	**2**	**3**	**4**	**5**	**6**	**7**	**8**	**9**	**10**	**11**	**12**	
**Window (+ or – days)**	**2**	**0**	**1**	**8**	**8**	**8**	**8**	**8**	**8**	**8**	**8**	**8**	**8**	**8**	**8**	
SD-OCT [N]	x	x		x	x	x	x	x	x	x	x	x	x	x	x	x
FAF [N]	x	x			SE		SE		x		SE		SE		x	x
NIR [N]	x	x			SE		SE		x		SE		SE		x	x
DCFP [N]	x														x	x
FFA [N]	x														x	x
OCT-A[R, S]		x[R]							x[R]						x[R]	x
Study Eye Determination	x															
Randomization		x														
Pegcetacoplan administration or Sham Injection[T]		x			x		x		x		x		x		x	x
Postinjection Assessment [O]		x			x		x		x		x		x		x	x
Follow-Up Call [P]		x			x		x									
Concomitant Medication/Concomitant Ocular Procedures [Q]	x	x	x	x	x	x	x	x	x	x	x	x	x	x	x	x
Adverse Events	x	x	x	x	x	x	x	x	x	x	x	x	x	x	x	x

Abbreviations: AH50 = alternative pathway of complement functional test; BCVA = best corrected visual acuity; CH50 = classical pathway of complement functional test; CNV = choroidal neovascularization; DCFP = digital color fundus photography; eCRF = electronic case report form; FAF = fundus autofluorescence; FFA = fundus fluorescein angiography; FRI = Functional Reading Independence Index; IOP = intraocular pressure; LL-BCVA = low luminance best corrected visual acuity; MNREAD = Minnesota Low-Vision Reading Test; NEI VFG-25 = National Eye Institute Visual Functioning Questionnaire 25-Item Version; NIR = near infrared reflectance; OCT-A = optical coherence tomography angiography; PK = pharmacokinetics; SD-OCT = spectral domain optical coherence tomography; SE = study eye; Term = termination; VEGF = vascular endothelial growth factor.

Note: All ocular assessments are to be performed for both eyes unless annotated with 'SE' (study eye) in the above schedule. All assessments should be performed on the same day. All study visits should be scheduled and projected based on the Day 1 visit date with the exception of Visit 2a which should be based on the Day 1 dose date.

A. For subjects that discontinue the study early, the early termination assessments should be performed after a minimum of 30 days have passed from the last dosing visit. If a subject reports for a scheduled visit and decides to terminate early prior to dosing, the visit should be considered the early termination visit and all early termination procedures should be performed At Month 24, all subjects should be offered entry into an open-label study.

B. At Day 1 (Visit 2), confirm subject eligibility through reviewing the inclusion/exclusion criteria and receive confirmation of eligibility from the reading center.

C. Significant medical/surgical history from the previous 5 years. Anti-VEGF treatments (fellow eye) and invasive ocular procedures performed within the past 5 years and while on study should also be recorded. Any history of tobacco use should be recorded.

D. Obtain prior to fluorescein angiography and before study drug administration.

E. At screening, serum pregnancy should be performed for women of childbearing potential. If positive, subject is not eligible to continue in the study.

F. Beginning at Day 1, perform the urine pregnancy test for women of childbearing potential at each treatment visit. If positive, perform a serum pregnancy test. If serum test is positive, study drug should not be administered and an early term visit should be completed.
G. Only subjects that sign the separate consent for the clinical repository and genotyping will have these samples collected. A 14-mL whole-blood sample will be collected each of the specified visits.
H. Blood pressure, respiratory rate, heart rate, and temperature. On dosing days, vital signs should be taken predose.
I. Height and weight should be measured at screening.
J. Perform assessments prior to dilating the eyes.
K. In select countries, the MNREAD or Radner Reading Charts should be done during the study visit, prior to dilating the eyes. It should be performed monocularly first, then binocularly.
L. To be administered by the masked site staff prior to any other assessments performed on that day. In-clinic assessments on the digital application should be completed after completion of all functional tests and quality of life measures prior to dilating the eyes.
M. At select sites, and for those subjects who decide to participate, the digital application will be completed on an electronic device and will consist of assessments for visual function and reading speed. Subjects will receive training at Day 1 on the use of the digital applications and the electronic device and will perform the tests using the application in the clinic at Month 1, Month 2, Month 3, Month 6, Month 12, Month 18, and Month 24. Beginning at Day 1, subjects will complete the functional assessments weekly at home. Subjects should be instructed to complete the assessments on the same day each week and at approximately the same time of day each week, if possible.
N. FAF and SD-OCT images, near infrared reflectance, fluorescein angiograms, and fundus photographs will be performed for the study eye only on days where 'SE' is specified and for both eyes at all other visits as specified in the above schedule and will be sent to the reading center for evaluation (consult the reading center manual for specifics on image capture, processing, and transmission). Images should be captured prior to dosing on dosing days. If a subject misses a study visit or images cannot be obtained at a specific visit, study staff should make every effort to obtain images at the next scheduled visit. If new active CNV is suspected, SD-OCT, FFA, and OCT-A (select sites) images should be collected and sent to the reading center for analysis.
O. Postinjection assessments should be performed within 5 minutes after dosing by the unmasked physician or study staff and should include a gross assessment of vision (finger-counting, hand motion, then light perception when applicable). If subject passes gross vision test, the subject may leave the site. If subject fails gross vision test, the tonometry should be performed. IOP should be ≤30 mm Hg in order for the subject to leave the site. If necessary, antiglaucomatous medication can be given to lower the IOP. If IOP is >30 mm Hg, assessments will continue every approximately 30 minutes from the previous measurement until the subject passes gross vision test and IOP is ≤30 mm Hg. Note: if the study eye is treated with a ranibizumab or aflibercept injection during the same visit as the study treatment (pegcetacoplan or sham), the treatment with ranibizumab or aflibercept must be performed first. The pre- and post- anti-VEGF IOP values must be measured and recorded on the eCRF.
P. Starting at Day 1, study subjects will be contacted by study site staff within 4 ± 2 days after each study treatment visit (through Month 4) to collect any information on any safety concerns, decrease in vision, eye pain, unusual ocular events, or any new ocular symptoms in the study eye. If the physician determines that there are any safety concerns, a follow-up visit should be scheduled as soon as possible.
Q. Record concomitant medications (ie, prescription and over-the-counter medications) used by the patient within 30 days of screening and throughout the subject's participation in the study.
R. In addition to the time points indicated on the study schedule, OCT-A should be performed at the time of any suspected new active CNV. If new active CNV is confirmed in the study eye, OCT-A should be repeated every 2 months for the study eye.
S. At select sites only.
T. Administration of study treatment (pegcetacoplan or sham) can be done on a separate day from the assessment visit if both days fall within the visit window. If this occurs on the randomization visit, then the administration of pegcetacoplan or sham should be done within 3 days of randomization and after approval from the medical monitor. When study treatment administration is on a day other than a study visit, then the only assessment that must be done on the day of study treatment administration is the preinjection IOP.

Table 11: Visit Schedule - Every-Other-Month Group Month 13 to Month 24

	Treatment												Early Term [A]
**Visit #**		**15**		**16**		**17**		**18**		**19**		**20**	
**Day**	**390**	**420**	**450**	**480**	**510**	**540**	**570**	**600**	**630**	**660**	**690**	**720**	
**Week**	**52**	**56**	**60**	**64**	**68**	**72**	**76**	**80**	**84**	**88**	**92**	**96**	
**Month**	**13**	**14**	**15**	**16**	**17**	**18**	**19**	**20**	**21**	**22**	**23**	**24**	
**Window (+ or – days)**		**16**		**16**		**16**		**16**		**16**		**16**	
Informed Consent/Assign Screening Number													
Demographic Data													
Inclusion/Exclusion Criteria [B]													
Medical/Surgical/Ocular History [C]													
Blood Draw—Safety Labs [D,E,F]						x						x	x
Urine Sample Collection [D, E,F]						x						x	x
Urine Pregnancy Test [D,E,F]		x		x		x		x		x		x	
Blood Draw—PK and Complement Profile (C3, CH50, AH50) [D,S]												x	x
Blood Draw—Anti-Pegcetacoplan Ab [D]		x				x						x	x
Blood Draw for Clinical Repository (if applicable) [D,G]												x	x
Vital Signs [H]		x		x		x		x		x		x	x
Physical Examination [I]												x	x
BCVA [J]		x		x		x		x		x		x	x
LL-BCVA [J]		x		x		x		x		x		x	x
MNREAD or Radner Reading Charts (select countries) [J,K]						x						x	x
Slitlamp Examination		x		x		x		x		x		x	x
Endothelial Cell Count[S]												x	x
NEI VFQ-25 [L]						x						x	x
FRI [L]						x						x	x
Home-Based Digital Applications (optional, select sites) [L,M,S]						x						x	
Dilated Indirect Ophthalmoscopy		x		x		x		x		x		x	x

Table 11: Visit Schedule - Every-Other-Month Group Month 13 to Month 24

	Treatment												Early Term [A]
**Visit #**		**15**		**16**		**17**		**18**		**19**		**20**	
**Day**	**390**	**420**	**450**	**480**	**510**	**540**	**570**	**600**	**630**	**660**	**690**	**720**	
**Week**	**52**	**56**	**60**	**64**	**68**	**72**	**76**	**80**	**84**	**88**	**92**	**96**	
**Month**	**13**	**14**	**15**	**16**	**17**	**18**	**19**	**20**	**21**	**22**	**23**	**24**	
**Window (+ or – days)**		**16**		**16**		**16**		**16**		**16**		**16**	
IOP Measurement		x		x		x		x		x		x	x
SD-OCT [N]		x		x		x		x		x		x	x
FAF [N]		SE		SE		x		SE		SE		x	x
NIR[N]		SE		SE		x		SE		SE		x	x
DCFP [N]												x	x
FFA [N]												x	x
OCT-A[R,S]						x[R]						x[R]	x
Study Eye Determination													
Pegcetacoplan administration or Sham Injection[T]		x		x		x		x		x			
Postinjection Assessment [O]		x		x		x		x		x			
Follow-Up Call [P]													
Concomitant Medication/Concomitant Ocular Procedures [Q]		x		x		x		x		x		x	x
Adverse Events		x		x		x		x		x		x	x

Abbreviations: AH50 = alternative pathway of complement functional test; BCVA = best corrected visual acuity; CH50 = classical pathway of complement functional test; CNV = choroidal neovascularization; DCFP = digital color fundus photography; eCRF = electronic case report form; FAF = fundus autofluorescence; FFA = fundus fluorescein angiography; FRI = Functional Reading Independence Index; IOP = intraocular pressure; LL-BCVA = low luminance best corrected visual acuity; MNREAD = Minnesota Low-Vision Reading Test; NEI VFG-25 = National Eye Institute Visual Functioning Questionnaire 25-Item Version; NIR = near infrared reflectance; OCT-A = optical coherence tomography angiography; PK = pharmacokinetics; SD-OCT = spectral domain optical coherence tomography; SE = study eye; Term = termination; VEGF = vascular endothelial growth factor.

Note: All ocular assessments are to be performed for both eyes unless annotated with 'SE' (study eye) in the above schedule. . All assessments should be performed on the same day. All study visits should be scheduled and projected based on the Day 1 visit date with the exception of Visit 2a which should be based on the Day 1 dose date.

A. For subjects that discontinue the study early, the early termination assessments should be performed after a minimum of 30 days have passed from the last dosing visit. If a subject reports for a scheduled visit and decides to terminate early prior to dosing, the visit should be considered the early termination visit and all early termination procedures should be performed. At Month 24, all subjects should be offered entry into an open-label study.

B. At Day 1 (Visit 2), confirm subject eligibility through reviewing the inclusion/exclusion criteria and receive confirmation of eligibility from the reading center.

C. Significant medical/surgical history from the previous 5 years. Anti-VEGF treatments (fellow eye) and invasive ocular procedures performed within the past 5 years and while on study should also be recorded. Any history of tobacco use should be recorded.

D. Obtain prior to fluorescein angiography and before study drug administration.
E. At screening, serum pregnancy should be performed for women of childbearing potential. If positive, subject is not eligible to continue in the study.
F. Beginning at Day 1, perform the urine pregnancy test for women of childbearing potential at each treatment visit. If positive, perform a serum pregnancy test. If serum test is positive, study drug should not be administered and an early term visit should be completed.
G. Only subjects that sign the separate consent for the clinical repository and genotyping will have these samples collected. A 14-mL whole-blood sample will be collected at each of the specified visits.
H. Blood pressure, respiratory rate, heart rate, and temperature. On dosing days, vital signs should be taken predose.
I. Height and weight should be measured at screening.
J. Perform assessments prior to dilating the eyes.
K. In select countries, the MNREAD or Radner Reading Charts should be done during the study visit, prior to dilating the eyes. It should be performed monocularly first, then binocularly.
L. To be administered by the masked site staff prior to any other assessments performed on that day. In-clinic assessments on the digital application should be completed after completion of all functional tests and quality of life measures prior to dilating the eyes.
M. At select sites, and for those subjects who decide to participate, the digital applications will be completed on an electronic device and will consist of assessments for visual function and reading speed. Subjects will receive training at Day 1 on the use of the digital applications and the electronic device and will perform the tests using the application in the clinic at Month 1, Month 2, Month 3, Month 6, Month 12, Month 18, and Month 24. Beginning at Day 1, subjects will complete the functional assessments weekly at home. Subjects should be instructed to complete the assessments on the same day each week and at approximately the same time of day each week, if possible.
N. FAF and SD-OCT images, near infrared reflectance, fluorescein angiograms, and fundus photographs will be performed for the study eye only on days where 'SE' is specified and for both eyes at all other visits as specified in the above schedule and will be sent to the reading center for evaluation (consult the reading center manual for specifics on image capture, processing, and transmission). Images should be captured prior to dosing on dosing days. If a subject misses a study visit or images cannot be obtained at a specific visit, study staff should make every effort to obtain images at the next scheduled visit. If new active CNV is suspected, SD-OCT, FFA, and OCT-A (select sites) images should be collected and sent to the reading center for analysis.
O. Postinjection assessments should be performed within 5 minutes after dosing by the unmasked physician or study staff and should include a gross assessment of vision (finger-counting, hand motion, then light perception when applicable). If subject passes gross vision test, the subject may leave the site. If subject fails gross vision test, the tonometry should be performed. IOP should be ≤30 mm Hg in order for the subject to leave the site. If necessary, antiglaucomatous medication can be given in order to lower IOP. If IOP is >30 mm Hg, assessments will continue every approximately 30 minutes from the previous measurement until the subject passes the gross vision test and IOP is ≤30 mm Hg. Note: if the study eye is treated with a ranibizumab or aflibercept injection during the same visit as the study treatment (pegcetacoplan or sham), the treatment with ranibizumab or aflibercept must be performed first. The pre- and post- anti-VEGF IOP values must be measured and recorded on the eCRF.
P. Starting at Day 1, study subjects will be contacted by study site staff within 4 ± 2 days after each study treatment visit (through Month 4) to collect any information on any safety concerns, decrease in vision, eye pain, unusual ocular events, or any new ocular symptoms in the study eye. If the investigator determines that there are any safety concerns, a follow-up visit should be scheduled as soon as possible.
Q. Record concomitant medications (ie, prescription and over-the-counter medications) used by the patient within 30 days of screening and throughout the subject's participation in the study.
R. In addition to the time points indicated on the study schedule, OCT-A should be performed at the time of any suspected new active CNV. If new active CNV is confirmed in the study eye, OCT-A should be repeated every 2 months for the study eye.
S. At select sites only.
T. Administration of study treatment (pegcetacoplan or sham) can be done on a separate day from the assessment visit if both days fall within the visit window. If this occurs on the randomization visit, then the administration of pegcetacoplan or sham should be done within 3 days of randomization and after approval from the medical monitor. When study treatment administration is on a day other than a study visit, then the only assessment that must be done on the day of study treatment administration is the preinjection IOP.

1.1 Protocol Changes to be followed during COVID-19 Restrictions

OVERVIEW

In response to the COVID-19 crisis, to ensure the safety of study subjects and Investigative Sites as well as proper conduct of the study, TEMPORARY changes to the protocol have been implemented. These changes should be followed only during COVID-19 restrictions and include extended IP administration windows, changes to masking rules, rescreening instructions, and a revised schedule of assessments.

Where feasible, sites could continue to follow the full schedule of assessments (based on their treatment group assignment).

EXTENDED IP ADMINISTRATION WINDOWS

In order to allow more flexibility to sites and subjects, and to potentially mitigate missed IP administration, an extended IP administration window can be followed. The extended window can ONLY be used in situations related to COVID-19 restrictions and after medical monitor approval. Footnote "T" of each COVID-19 assessment table below reflects these extended IP options.

Per protocol, the IP administration window is as follows:

- Monthly treatment group: +/– 8 days for the entire study duration
- EOM treatment group: +/– 8 days for the first study year and +/- 16 days for the second study year

During COVID-19 restrictions, the IP administration window can be extended to the following:

- Monthly treatment group: –8 days to +15 days. Note, interval for consecutive injections must be at least 14 days.
- EOM treatment group: –8 days to +30 days for the first study year and –16 days to +30 days for the second study year.

MASKING RULES

Due to current COVID-19 restrictions, clinical sites might encounter difficulties maintaining appropriate clinic staffing to satisfy the approved masking rules for the APL2-303 (Derby) study. Based on this, and in an attempt to minimize the amount of missed data and IP administrations, Apellis is implementing a temporary adjustment to the study masking rules.

This temporary change must be approved by the Apellis Medical Director **prior to** implementation and must be documented via a temporary and modified delegation of authority Log. Each masked assessment performed by an unmasked staff and vice versa (even with Apellis approval and following the below guidelines) should be documented.

The principal investigator (PI) is responsible for the overall oversight of the study site data and s/he will not be allowed to switch into an unmasked role. Every masked individual that performs IP administration and/or postinjection assessment (all unmasked assessment) as a temporary measure, will **permanently** be considered an unmasked individual and will not be able to perform masked assessments once these exemptions are lifted.

RESCREENING PROCEDURE

Prior to the implementation of these temporary changes, sites continuing to screen patients have been encouraged to complete the screening and baseline assessments in their entirety. However, if a subject was deemed a screen failure for not being able to meet the original screening window (Day –28 to Day –1 [+/- 2 days]) due to COVID-19 related restrictions, a rescreening visit is allowed and should be followed according to the 2 scenarios below.

Subjects Who Completed Screening and Were Considered Eligible by Reading Center and Investigator

Subjects who were screened prior to 30 March 2020 and completed all screening assessments (as described in the Schedule of Assessments Table 8 [every month treatment group] and Table 10 [every other month [EOM] treatment group]) and considered eligible by the reading center and investigator and are able to return to the clinic within 90 days of initial screening, will receive a new subject ID number and undergo an *abbreviated screening,* prior to randomization, that includes the following assessments:

- Informed consent/assign new screening number
- Normal luminance best corrected visual acuity assessment
- Slitlamp examination
- Dilated indirect ophthalmoscopy
- IOP measurement
- SD-OCT*
- Concomitant medication/concomitant ocular procedures collection
- AE collection

*SD-OCT images collected at this visit will not be used by the reading center to determine eligibility but should be used by the investigator to detect any potential new exclusion criterion.

If the investigator deems it necessary, additional assessments can be performed if there is a concern that the subject might now meet an exclusion criterion that was not the case during the original screening (eg, FFA to exclude the presence of CNV).

Subjects With Incomplete Screening Assessment

Subjects that signed the informed consent but were not able to complete all screening assessments due to COVID-19-related restrictions are not eligible for the abbreviated screening. These subjects can be rescreened but must follow the standard screening schedule of assessment (as described in the Schedule of Assessments Table 8 [every month treatment group] and Table 10 [EOM treatment group]). These subjects will also receive a new screening ID number.

MINIMUM SCHEDULE OF ASSESSMENT

Schedule of Assessments

Where feasible, sites could continue to follow the full schedule of assessments (based on their treatment group assignment). The minimum assessment tables, only to be followed during this COVID-19 effort and if determined necessary to use based on the investigator's clinical judgment, are provided below to reduce the time required for each study visit. Subjects in the EOM treatment group do not need to be seen for the non-IP administration visits. Assessments not performed (even those that have been removed in the minimum assessment table) should be documented.

Subjects that are not able to come into the clinic for a study visit due to COVID-19-related restrictions, including visits for the EOM group that do not include IP administration, should be contacted via the phone for the collection of AEs (including SAEs) and concomitant medications. **All SAEs are still required to be reported to Apellis within 24 hours of site awareness, even if reported via phone call**. All communications via phone call should also be documented in the source documents and in the respective CRF page. In addition, these subjects should be instructed to self-monitor their vision at home and report any changes in vision or their overall health via phone call. The site must inform the Sponsor of any subjects lost to follow-up.

It is critical that local, country, and regional governance regarding COVID-19 is followed along with your best clinical judgment when managing this situation. All visits or assessments missed as a result of COVID-19 will be captured in the case report forms.

Table 12 (TRACKED): COVID-19 VISIT SCHEDULE—Monthly Group—Screening, Day 1 Through Month 12

	Screening	Treatment														Early Term[A]
**Visit #**	**1**	**2**	**2A**	**3**	**4**	**5**	**6**	**7**	**8**	**9**	**10**	**11**	**12**	**13**	**14**	
**Day**	**–28 to –1**	**1**	**7**	**30**	**60**	**90**	**120**	**150**	**180**	**210**	**240**	**270**	**300**	**330**	**360**	
**Week**	**0**	**0**	**1**	**4**	**8**	**12**	**16**	**20**	**24**	**28**	**32**	**36**	**40**	**44**	**48**	
**Month**	**0**	**0**	**0**	**1**	**2**	**3**	**4**	**5**	**6**	**7**	**8**	**9**	**10**	**11**	**12**	
**Window (+ or – days)**	**2**	**0**	**1**	**8**	**8**	**8**	**8**	**8**	**8**	**8**	**8**	**8**	**8**	**8**	**8**	
Informed Consent/Assign Screening Number	x															
Demographic Data	x															
Inclusion/Exclusion Criteria[B]	x	x														
Medical/Surgical/Ocular History[C]	x															
Blood Draw—Safety Labs[D,E,F]	x	x			~~x~~				~~x~~						x	x
Urine Sample Collection[D, E,F]	x	x			~~x~~				~~x~~						x	x
Urine Pregnancy Test[D,E,F]		x		x	x	x	x	x	x	x	x	x	x	x	x	
Blood Draw—PK and Complement Profile (C3, CH50, AH50)[D, S]		x	x	x					x						x	x
Blood Draw—Genotyping (if applicable)[D]					~~x~~											
Blood Draw- Anti-Pegcetacoplan Ab[D]		x		~~x~~	~~x~~				~~x~~						x	x
Blood Draw for Clinical Repository (if applicable)[D,G]					~~x~~				~~x~~						~~x~~	~~x~~
Vital Signs[H]	x	x		~~x~~	~~x~~	~~x~~	~~x~~	~~x~~	x	~~x~~	~~x~~	~~x~~	~~x~~	~~x~~	x	x
Physical Examination[I]	x														x	x
BCVA[J]	x	x		x	x	x	x	x	x	x	x	x	x	x	x	x
LL-BCVA[J]		x		~~x~~	~~x~~	~~x~~	~~x~~	~~x~~	x	~~x~~	~~x~~	~~x~~	~~x~~	~~x~~	x	x
MNREAD or Radner Reading Charts (select countries)[J,K]		x							~~x~~						x	x
Slitlamp Examination	x	x		x	x	x	x	x	x	x	x	x	x	x	x	x
Endothelial Cell Count[S]		x							x						x	x
NEI VFQ-25[L]		x							~~x~~						x	x
FRI[L]		x							~~x~~						x	x

Table 12 (TRACKED): COVID-19 VISIT SCHEDULE—Monthly Group—Screening, Day 1 Through Month 12

	Screening	Treatment														Early Term[A]
**Visit #**	**1**	**2**	**2A**	**3**	**4**	**5**	**6**	**7**	**8**	**9**	**10**	**11**	**12**	**13**	**14**	
**Day**	**–28 to –1**	**1**	**7**	**30**	**60**	**90**	**120**	**150**	**180**	**210**	**240**	**270**	**300**	**330**	**360**	
**Week**	**0**	**0**	**1**	**4**	**8**	**12**	**16**	**20**	**24**	**28**	**32**	**36**	**40**	**44**	**48**	
**Month**	**0**	**0**	**0**	**1**	**2**	**3**	**4**	**5**	**6**	**7**	**8**	**9**	**10**	**11**	**12**	
**Window (+ or – days)**	**2**	**0**	**1**	**8**	**8**	**8**	**8**	**8**	**8**	**8**	**8**	**8**	**8**	**8**	**8**	
Home-Based Digital Applications [L,M S]		x		~~x~~	~~x~~	~~x~~			~~x~~						x	
Dilated Indirect Ophthalmoscopy	x	x		x	x	x	x	x	x	x	x	x	x	x	x	x
IOP Measurement	x	x		x	x	x	x	x	x	x	x	x	x	x	x	x
SD-OCT [N]	x	x		~~x~~	~~x~~	~~x~~	~~x~~	~~x~~	x	~~x~~	~~x~~	~~x~~	~~x~~	~~x~~	x	x
FAF [N]	x	x			~~SE~~		~~SE~~		x		~~SE~~		~~SE~~		x	x
NIR [N]	x	x			~~SE~~		~~SE~~		x		~~SE~~		~~SE~~		x	x
DCFP [N]	x														x	x
FFA [N]	x														x	x
OCT-A[R.S]		x [R]							~~x [R]~~						x [R]	x
Study Eye Determination	x															
Randomization		x														
Pegcetacoplan administration or Sham Injection[T]		x		x	x	x	x	x	x	x	x	x	x	x	x	
Postinjection Assessment [O]		x		x	x	x	x	x	x	x	x	x	x	x	x	
Follow-Up Call [P]		x		x	x	x										
Concomitant Medication/Concomitant Ocular Procedures [Q]	x	x	~~x~~	x	x	x	x	x	x	x	x	x	x	x	x	x
Adverse Events	x	x	~~x~~	x	x	x	x	x	x	x	x	x	x	x	x	x

Abbreviations: AH50 = alternative pathway of complement functional test; BCVA = best corrected visual acuity; CH50 = classical pathway of complement functional test; CNV = choroidal neovascularization; DCFP = digital color fundus photography; eCRF = electronic case report form; FAF = fundus autofluorescence; FFA = fundus fluorescein angiography; FRI = Functional Reading Independence Index; IOP = intraocular pressure; LL-BCVA = low luminance best corrected visual acuity; MNREAD = Minnesota Low-Vision Reading Test; NEI VFG-25 = National Eye Institute Visual Functioning Questionnaire 25-Item Version; NIR = near infrared reflectance; OCT-A = optical coherence tomography angiography; PK = pharmacokinetics; SD-OCT = spectral domain optical coherence tomography; SE = study eye; Term = termination; VEGF = vascular endothelial growth factor.

Note: All ocular assessments are to be performed for both eyes unless annotated with 'SE' (study eye) in the above schedule. All assessments should be performed on the same day. All study visits should be scheduled and projected based on the Day 1 visit date with the exception of Visit 2a which should be based on the Day 1 dose date.

A. For subjects that discontinue the study early, the early termination assessments should be performed after a minimum of 30 days have passed from the last dosing visit. If a subject reports for a scheduled visit and decides to terminate early prior to dosing, the visit should be considered the early termination visit and all early termination procedures should be performed. At Month 24, all subjects should be offered entry into an open-label study.
B. At Day 1 (Visit 2), confirm subject eligibility through reviewing the inclusion/exclusion criteria and receive confirmation of eligibility from the reading center.
C. Significant medical/surgical history from the previous 5 years. Anti-VEGF treatments (fellow eye) and invasive ocular procedures performed within the past 5 years and while on study should also be recorded. Any history of tobacco use should be recorded.
D. Obtain prior to fluorescein angiography and before study drug administration.
E. At screening, serum pregnancy should be performed for women of childbearing potential. If positive, subject is not eligible to continue in the study.
F. Beginning at Day 1, perform the urine pregnancy test for women of childbearing potential at each treatment visit. If positive, perform a serum pregnancy test. If serum test is positive, study drug should not be administered and an early term visit should be completed.
G. Only subjects that sign the separate consent for the clinical repository and genotyping will have these samples collected. A 14-mL whole-blood sample will be collected at each of the specified visits.
H. Blood pressure, respiratory rate, heart rate, and temperature. On dosing days, vital signs should be taken predose.
I. Height and weight should be collected at screening.
J. Perform assessments prior to dilating the eyes.
K. In select countries, the MNREAD or Radner Reading Charts should be done during the study visit, prior to dilating the eyes. It should be performed monocularly first, then binocularly.
L. To be administered by the masked site staff prior to any other assessments performed on that day. In-clinic assessments on the digital application should be completed after completion of all functional tests and quality of life measures prior to dilating the eyes.
M. At select sites, and for those subjects who decide to participate, the digital applications will be completed on an electronic device and will consist of assessments for visual function and reading speed. Subjects will receive training at Day 1 on the use of the digital applications and the electronic device and will perform the tests using the application in the clinic at Month 1, Month 2, Month 3, Month 6, Month 12, Month 18, and Month 24. Beginning at Day 1, subjects will complete the functional assessments weekly at home. Subjects should be instructed to complete the assessments on the same day each week and at approximately the same time of day each week, if possible.
N. FAF and SD-OCT images, near infrared reflectance, fluorescein angiograms, and fundus photographs will be performed for the study eye only on days where 'SE' is specified and for both eyes at all other visits as specified in the above schedule and will be sent to the reading center for evaluation (consult the reading center manual for specifics on image capture, processing, and transmission). Images should be captured prior to dosing on dosing days. If a subject misses a study visit or images cannot be obtained at a specific visit, study staff should make every effort to obtain images at the next scheduled visit. If new active CNV is suspected, SD-OCT, FFA, and OCT-A (select sites) images must be collected and sent to the reading center for analysis.
O. Postinjection assessments should be performed within 5 minutes after dosing by the unmasked physician or study staff and should include a gross assessment of vision (finger-counting, hand motion, then light perception when applicable). If subject passes gross vision test, the subject may leave the site. If subject fails gross vision test, the tonometry should be performed. IOP should be ≤30 mm Hg in order for the subject to leave the site. If necessary, antiglaucomatous medication can be given to lower the IOP. If IOP is >30 mm Hg, assessments will continue every approximately 30 minutes from the previous measurement until the subject passes gross vision test and IOP is ≤30 mm Hg. Note: if the study eye is treated with a ranibizumab or aflibercept injection during the same visit as the study treatment (pegcetacoplan or sham), the treatment with ranibizumab or aflibercept must be performed first. The pre- and post- anti-VEGF IOP values must be measured and recorded on the eCRF.
P. Starting at Day 1, study subjects will be contacted by study site staff within 4 ± 2 days after each study treatment visit (through Month 3) to collect any information on any safety concerns, decrease in vision, eye pain, unusual ocular events, or any new ocular symptoms in the study eye. If the physician determines that there are any safety concerns, a follow-up visit should be scheduled as soon as possible.
Q. Record concomitant medications (ie, prescription and over-the-counter medications) used by the patient within 30 days of screening and throughout the subject's participation in the study.
R. In addition to the time points indicated on the study schedule, OCT-A should be performed at the time of any suspected new active CNV. If new active CNV is confirmed in the study eye, OCT-A should be repeated every 2 months for the study eye.
S. At select sites only.
T. Administration of study treatment (pegcetacoplan or sham) can be done on a separate day from the assessment visit if both days fall within the visit window. If this occurs on the randomization visit, then the administration of pegcetacoplan or sham should be done within 3 days of randomization and after approval from the medical monitor. When study treatment administration is on a day other than a study visit, then the only assessment that must be done on the day of study treatment administration is the preinjection IOP. **During the COVID-19 pandemic, the following flexibility is allowed for IP administration: –8 days to +15 days after medical monitor approval. Note, interval for consecutive injections must be at least 14 days.**

Table 13 (TRACKED): COVID-19 VISIT SCHEDULE—Monthly Group—Month 13 to Month 24

	Treatment												Early Term [A]
**Visit #**	**15**	**16**	**17**	**18**	**19**	**20**	**21**	**22**	**23**	**24**	**25**	**26**	
**Day**	**390**	**420**	**450**	**480**	**510**	**540**	**570**	**600**	**630**	**660**	**690**	**720**	
**Week**	**52**	**56**	**60**	**64**	**68**	**72**	**76**	**80**	**84**	**88**	**92**	**96**	
**Month**	**13**	**14**	**15**	**16**	**17**	**18**	**19**	**20**	**21**	**22**	**23**	**24**	
**Window (+ or – days)**	**8**	**8**	**8**	**8**	**8**	**8**	**8**	**8**	**8**	**8**	**8**	**8**	
Informed Consent/Assign Screening Number													
Demographic Data													
Inclusion/Exclusion Criteria [B]													
Medical/Surgical/Ocular History [C]													
Blood Draw—Safety Labs [D,E,F]						~~x~~						x	x
Urine Sample Collection [D,E,F]						~~x~~						x	x
Urine Pregnancy Test [D,E,F]	x	x	x	x	x	x	x	x	x	x	x	x	
Blood Draw—PK and Complement Profile (C3, CH50, AH50) [D,S]												x	x
Blood Draw—Genotyping (if applicable) [D]													
Blood Draw—Anti-Pegcetacoplan Ab [D]		~~x~~				~~x~~						x	x
Blood Draw for Clinical Repository (if applicable)[D,G]												~~x~~	~~x~~
Vital Signs [H]	~~x~~	~~x~~	~~x~~	~~x~~	~~x~~	x	~~x~~	~~x~~	~~x~~	~~x~~	~~x~~	x	x
Physical Examination [I]												x	x
BCVA [J]	x	x	x	x	x	x	x	x	x	x	x	x	x
LL-BCVA [I]	~~x~~	~~x~~	~~x~~	~~x~~	~~x~~	x	~~x~~	~~x~~	~~x~~	~~x~~	~~x~~	x	x
MNREAD or Radner Reading Charts (select countries) [J,K]						~~x~~						x	x
Slitlamp Examination	x	x	x	x	x	x	x	x	x	x	x	x	x
Endothelial Cell Count[S]												x	x
NEI VFQ-25 [L]						~~x~~						x	x
FRI [L]						~~x~~						x	x
Dilated Indirect Ophthalmoscopy	x	x	x	x	x	x	x	x	x	x	x	x	x
Home-Based Digital Applications [L,M,S]						~~x~~						~~x~~	

Table 13 (TRACKED): COVID-19 VISIT SCHEDULE—Monthly Group—Month 13 to Month 24

	**Treatment**												**Early Term [A]**
**Visit #**	**15**	**16**	**17**	**18**	**19**	**20**	**21**	**22**	**23**	**24**	**25**	**26**	
**Day**	**390**	**420**	**450**	**480**	**510**	**540**	**570**	**600**	**630**	**660**	**690**	**720**	
**Week**	**52**	**56**	**60**	**64**	**68**	**72**	**76**	**80**	**84**	**88**	**92**	**96**	
**Month**	**13**	**14**	**15**	**16**	**17**	**18**	**19**	**20**	**21**	**22**	**23**	**24**	
**Window (+ or – days)**	**8**	**8**	**8**	**8**	**8**	**8**	**8**	**8**	**8**	**8**	**8**	**8**	
IOP Measurement	x	x	x	x	x	x	x	x	x	x	x	x	x
SD-OCT [N]	~~x~~	~~x~~	~~x~~	~~x~~	~~x~~	x	~~x~~	~~x~~	~~x~~	~~x~~	~~x~~	x	x
FAF [N]		~~SE~~		~~SE~~		x		~~SE~~		~~SE~~		x	x
NIR [N]		~~SE~~		~~SE~~		x		~~SE~~		~~SE~~		x	x
DCFP [N]												x	x
FFA [N]												x	x
OCT-A [R,S]						~~x [R]~~						x [R]	x
Study Eye Determination													
Randomization													
Pegcetacoplan administration or Sham Injection[T]	x	x	x	x	x	x	x	x	x	x	x		
Postinjection Assessment [O]	x	x	x	x	x	x	x	x	x	x	x		
Follow-Up Call [P]													
Concomitant Medication/Concomitant Ocular Procedures [Q]	x	x	x	x	x	x	x	x	x	x	x	x	x
Adverse Events	x	x	x	x	x	x	x	x	x	x	x	x	x

Abbreviations: AH50 = alternative pathway of complement functional test; BCVA = best corrected visual acuity; CH50 = classical pathway of complement functional test; CNV = choroidal neovascularization; DCFP = digital color fundus photography; eCRF = electronic case report form; FAF = fundus autofluorescence; FFA = fundus fluorescein angiography; FRI = Functional Reading Independence Index; IOP = intraocular pressure; LL-BCVA = low luminance best corrected visual acuity; MNREAD = Minnesota Low-Vision Reading Test; NEI VFG-25 = National Eye Institute Visual Functioning Questionnaire 25-Item Version; NIR = near infrared reflectance; OCT-A = optical coherence tomography angiography; PK = pharmacokinetics; SD-OCT = spectral domain optical coherence tomography; SE = study eye; Term = termination; VEGF = vascular endothelial growth factor.

Note: All ocular assessments are to be performed for both eyes unless annotated with 'SE' (study eye) in the above schedule. All assessments should be performed on the same day. All study visits should be scheduled and projected based on the Day 1 visit date.

A. For subjects that discontinue the study early, the early termination assessments should be performed after a minimum of 30 days have passed from the last dosing visit. If a subject reports for a scheduled visit and decides to terminate early prior to dosing, then the visit should be considered the early termination visit and all early termination procedures should be performed. At Month 24, all subjects should be offered entry into an open-label study.
B. At Day 1 (Visit 2), confirm subject eligibility through reviewing the inclusion/exclusion criteria and receive confirmation of eligibility from the reading center.
C. Significant medical/surgical history from the previous 5 years. Anti-VEGF treatments (fellow eye) and invasive ocular procedures performed within the past 5 years and while on study should also be recorded. Any history of tobacco use should be recorded.
D**.** Obtain prior to fluorescein angiography and before study drug administration.
E. At screening, serum pregnancy should be performed for women of childbearing potential. If positive, subject is not eligible to continue in the study.
F. Beginning at Day 1, perform the urine pregnancy test for women of childbearing potential at each treatment visit. If positive, perform a serum pregnancy test. If serum test is positive, study drug should not be administered and an early term visit should be completed.
G. Only subjects that sign the separate consent for the clinical repository and genotyping will have these samples collected. A 14-mL whole-blood sample will be collected at each of the specified visits.
H. Blood pressure, respiratory rate, heart rate, and temperature. On dosing days, vital signs should be taken predose.
I. Height and weight should be measured at screening.
J**.** Perform assessments prior to dilating the eyes.
K. In select countries, the MNREAD or Radner Reading Charts should be done during the study visit, prior to dilating the eyes. It should be performed monocularly first, then binocularly.
L. To be administered by the masked site staff prior to any other assessments performed on that day. In-clinic assessments on the digital application should be completed after completion of all functional tests and quality of life measures prior to dilating the eyes.
M. At select sites, and for those subjects who decide to participate, the digital applications will be completed on an electronic device and will consist of assessments for visual function and reading speed. Subjects will receive training at Day1 on the use of the digital applications and the electronic device and will perform the tests using the application in the clinic at Month 1, Month 2, Month 3, Month 6, Month 12, Month 18, and Month 24. Beginning at Day 1, subjects will complete the functional assessments weekly at home. Subjects should be instructed to complete the assessments on the same day each week and at approximately the same time of day each week, if possible.
N. FAF and SD-OCT images, near infrared reflectance, fluorescein angiograms, and fundus photographs will be performed for the study eye only on days where 'SE' is specified and for both eyes at all other visits as specified in the above schedule and will be sent to the reading center for evaluation (consult the reading center manual for specifics on image capture, processing, and transmission). Images should be captured prior to dosing on dosing days. If a subject misses a study visit or images cannot be obtained at a specific visit, study staff should make every effort to obtain images at the next scheduled visit. If new active CNV is suspected, SD-OCT, FFA, and OCT-A (selected sites) images should be collected and sent to the reading center for analysis.
O. Postinjection assessments should be performed within 5 minutes after dosing by the unmasked physician or study staff and should include a gross assessment of vision (finger-counting, hand motion, then light perception when applicable). If subject passes gross vision test, the subject may leave the site. If subject fails gross vision test, the tonometry should be performed. IOP should be ≤30 mm Hg in order for the subject to leave the site. If necessary, antiglaucomatous medication can be given to lower the IOP. If IOP is >30 mm Hg, assessments will continue every approximately 30 minutes from the previous measurement until the subject passes gross vision test and IOP is ≤30 mm Hg. Note: if the study eye is treated with a ranibizumab or aflibercept injection during the same visit as the study treatment (pegcetacoplan or sham), the treatment with ranibizumab or aflibercept must be performed first. The pre- and post- anti-VEGF IOP values must be measured and recorded on the eCRF.
P. Starting at Day 1, study subjects will be contacted by study site staff within 4 ± 2 days after each study treatment visit (through Month 3) to collect any information on any safety concerns, decrease in vision, eye pain, unusual ocular events, or any new ocular symptoms in the study eye. If the physician determines that there are any safety concerns, a follow-up visit should be scheduled as soon as possible.
Q. Record concomitant medications (ie, prescription and over-the-counter medications) used by the patient within 30 days of screening and throughout the subject's participation in the study.
R. In addition to the time points indicated on the study schedule, OCT-A should be performed at the time of any suspected new active CNV. If new active CNV is confirmed in the study eye, OCT-A should be repeated every 2 months for the study eye.
S. At select sites only.
T. Administration of pegcetacoplan or sham can be done on separate days from the assessment visit if both days fall within the visit window. Administration of study treatment (pegcetacoplan or sham) can be done on a separate day from the assessment visit if both days fall within the visit window. If this occurs on the randomization visit, then the administration of pegcetacoplan or sham should be done within 3 days of randomization and after approval from the medical monitor. When study treatment administration is on a day other than a study visit, then the only assessment that must be done on the day of study treatment administration is preinjection IOP. During the COVID-19 pandemic, the following flexibility is allowed for IP administration: –8 days to +15 days after medical monitor approval. Note, interval for consecutive injections must be at least 14 days.

Table 14 (TRACKED): COVID-19 VISIT SCHEDULE—Every-Other-Month Group—Screening, Day 1 Through Month 12

	Screening	Treatment														Early Term[A]
**Visit #**	**1**	**2**	**2A**	**3**	**4**	**5**	**6**	**7**	**8**	**9**	**10**	**11**	**12**	**13**	**14**	
**Day**	**–28 to –1**	**1**	**7**	**30**	**60**	**90**	**120**	**150**	**180**	**210**	**240**	**270**	**300**	**330**	**360**	
**Week**	**0**	**0**	**1**	**4**	**8**	**12**	**16**	**20**	**24**	**28**	**32**	**36**	**40**	**44**	**48**	
**Month**	**0**	**0**	**0**	**1**	**2**	**3**	**4**	**5**	**6**	**7**	**8**	**9**	**10**	**11**	**12**	
**Window (+ or – days)**	**2**	**0**	**1**	**8**	**8**	**8**	**8**	**8**	**8**	**8**	**8**	**8**	**8**	**8**	**8**	
Informed Consent/Assign Screening Number	x															
Demographic Data	x															
Inclusion/Exclusion Criteria [B]	x	x														
Medical/Surgical/Ocular History [C]	x															
Blood Draw—Safety Labs [D,E,F]	x	x			~~x~~				~~x~~						x	x
Urine Sample Collection [D,E,F]	x	x			~~x~~				~~x~~						x	x
Urine Pregnancy Test [D,E,F]		x			x		x		x		x		x		x	
Blood Draw—PK and Complement Profile (C3, CH50, AH50) [D,S]		x	x	x					x						x	x
Blood Draw—Genotyping (if applicable) [D]					~~x~~											
Blood Draw- Anti-Pegcetacoplan Ab [D]		x		~~x~~	~~x~~				~~x~~						x	x
Blood Draw for Clinical Repository (if applicable) [D,G]					~~x~~				~~x~~						~~x~~	x
Vital Signs [H]	x	x		~~x~~	~~x~~	~~x~~	~~x~~	~~x~~	x	~~x~~	~~x~~	~~x~~	~~x~~	~~x~~	x	x
Physical Examination [I]	x														x	x
BCVA [J]	x	x		~~x~~	x	~~x~~	x	~~x~~	x	~~x~~	x	~~x~~	x	~~x~~	x	x
LL-BCVA [J]		x		~~x~~	~~x~~	~~x~~	~~x~~	~~x~~	x	~~x~~	~~x~~	~~x~~	~~x~~	~~x~~	x	x
MNREAD or Radner Reading Charts (select countries) [J,K]		x							~~x~~						x	x
Slitlamp Examination	x	x		~~x~~	x	~~x~~	x	~~x~~	x	~~x~~	x	~~x~~	x	~~x~~	x	x
Endothelial Cell Count[S]		x							x						x	x
NEI VFQ-25 [L]		x							~~x~~						x	x
FRI [L]		x							~~x~~						x	x
Home-Based Digital Applications [L,M,S]		x		~~x~~	~~x~~	~~x~~			~~x~~						x	

Table 14 (TRACKED): COVID-19 VISIT SCHEDULE—Every-Other-Month Group—Screening, Day 1 Through Month 12

	Screening	Treatment														Early Term[A]
**Visit #**	**1**	**2**	**2A**	**3**	**4**	**5**	**6**	**7**	**8**	**9**	**10**	**11**	**12**	**13**	**14**	
**Day**	**–28 to –1**	**1**	**7**	**30**	**60**	**90**	**120**	**150**	**180**	**210**	**240**	**270**	**300**	**330**	**360**	
**Week**	**0**	**0**	**1**	**4**	**8**	**12**	**16**	**20**	**24**	**28**	**32**	**36**	**40**	**44**	**48**	
**Month**	**0**	**0**	**0**	**1**	**2**	**3**	**4**	**5**	**6**	**7**	**8**	**9**	**10**	**11**	**12**	
**Window (+ or – days)**	**2**	**0**	**1**	**8**	**8**	**8**	**8**	**8**	**8**	**8**	**8**	**8**	**8**	**8**	**8**	
Dilated Indirect Ophthalmoscopy	x	x		~~x~~	x	~~x~~	x	~~x~~	x	~~x~~	x	~~x~~	x	~~x~~	x	x
IOP Measurement	x	x		~~x~~	x	~~x~~	x	~~x~~	x	~~x~~	x	~~x~~	x	~~x~~	x	x
SD-OCT [N]	x	x		~~x~~	~~x~~	~~x~~	~~x~~	~~x~~	x	~~x~~	~~x~~	~~x~~	~~x~~	~~x~~	x	x
FAF [N]	x	x			~~SE~~		~~SE~~		x		~~SE~~		~~SE~~		x	x
NIR [N]	x	x			~~SE~~		~~SE~~		x		~~SE~~		~~SE~~		x	x
DCFP [N]	x														x	x
FFA [N]	x														x	x
OCT-A[R, S]		x[R]							~~x~~[R]						x[R]	x
Study Eye Determination	x															
Randomization		x														
Pegcetacoplan administration or Sham Injection[T]		x			x		x		x		x		x		x	
Postinjection Assessment [O]		x			x		x		x		x		x		x	
Follow-Up Call [P]		x			x		x									
Concomitant Medication/Concomitant Ocular Procedures [Q`]	x	x	x	~~x~~	x	~~x~~	x	~~x~~	x	~~x~~	x	~~x~~	x	~~x~~	x	x
Adverse Events	x	x	x	~~x~~	x	~~x~~	x	~~x~~	x	~~x~~	x	~~x~~	x	~~x~~	x	x

Abbreviations: AH50 = alternative pathway of complement functional test; BCVA = best corrected visual acuity; CH50 = classical pathway of complement functional test; CNV = choroidal neovascularization; DCFP = digital color fundus photography; eCRF = electronic case report form; FAF = fundus autofluorescence; FFA = fundus fluorescein angiography; FRI = Functional Reading Independence Index; IOP = intraocular pressure; LL-BCVA = low luminance best corrected visual acuity; MNREAD = Minnesota Low-Vision Reading Test; NEI VFG-25 = National Eye Institute Visual Functioning Questionnaire 25-Item Version; NIR = near infrared reflectance; OCT-A = optical coherence tomography angiography; PK = pharmacokinetics; SD-OCT = spectral domain optical coherence tomography; SE = study eye; Term = termination; VEGF = vascular endothelial growth factor.

Note: All ocular assessments are to be performed for both eyes unless annotated with 'SE' (study eye) in the above schedule. All assessments should be performed on the same day. All study visits should be scheduled and projected based on the Day 1 visit date with the exception of Visit 2a which should be based on the Day 1 dose date.

A. For subjects that discontinue the study early, the early termination assessments should be performed after a minimum of 30 days have passed from the last dosing visit. If a subject reports for a scheduled visit and decides to terminate early prior to dosing, the visit should be considered the early termination visit and all early termination procedures should be performed At Month 24, all subjects should be offered entry into an open-label study.
B. At Day 1 (Visit 2), confirm subject eligibility through reviewing the inclusion/exclusion criteria and receive confirmation of eligibility from the reading center.
C. Significant medical/surgical history from the previous 5 years. Anti-VEGF treatments (fellow eye) and invasive ocular procedures performed within the past 5 years and while on study should also be recorded. Any history of tobacco use should be recorded.
D. Obtain prior to fluorescein angiography and before study drug administration.
E. At screening, serum pregnancy should be performed for women of childbearing potential. If positive, subject is not eligible to continue in the study.
F. Beginning at Day 1, perform the urine pregnancy test for women of childbearing potential at each treatment visit. If positive, perform a serum pregnancy test. If serum test is positive, study drug should not be administered and an early term visit should be completed.
G. Only subjects that sign the separate consent for the clinical repository and genotyping will have these samples collected. A 14-mL whole-blood sample will be collected each of the specified visits.
H. Blood pressure, respiratory rate, heart rate, and temperature. On dosing days, vital signs should be taken predose.
I. Height and weight should be measured at screening.
J. Perform assessments prior to dilating the eyes.
K. In select countries, the MNREAD or Radner Reading Charts should be done during the study visit, prior to dilating the eyes. It should be performed monocularly first, then binocularly.
L. To be administered by the masked site staff prior to any other assessments performed on that day. In-clinic assessments on the digital application should be completed after completion of all functional tests and quality of life measures prior to dilating the eyes.
M. At select sites, and for those subjects who decide to participate, the digital application will be completed on an electronic device and will consist of assessments for visual function and reading speed. Subjects will receive training at Day 1 on the use of the digital applications and the electronic device and will perform the tests using the application in the clinic at Month 1, Month 2, Month 3, Month 6, Month 12, Month 18, and Month 24. Beginning at Day 1, subjects will complete the functional assessments weekly at home. Subjects should be instructed to complete the assessments on the same day each week and at approximately the same time of day each week, if possible.
N. FAF and SD-OCT images, near infrared reflectance, fluorescein angiograms, and fundus photographs will be performed for the study eye only on days where 'SE' is specified and for both eyes at all other visits as specified in the above schedule and will be sent to the reading center for evaluation (consult the reading center manual for specifics on image capture, processing, and transmission). Images should be captured prior to dosing on dosing days. If a subject misses a study visit or images cannot be obtained at a specific visit, study staff should make every effort to obtain images at the next scheduled visit. If new active CNV is suspected, SD-OCT, FFA, and OCT-A (select sites) images should be collected and sent to the reading center for analysis.
O. Post- injection assessments should be performed within 5 minutes after dosing by the unmasked physician or study staff and should include a gross assessment of vision (finger-counting, hand motion, then light perception when applicable). If subject passes gross vision test, the subject may leave the site. If subject fails gross vision test, the tonometry should be performed. IOP should be ≤30 mm Hg in order for the subject to leave the site. If necessary, antiglaucomatous medication can be given to lower the IOP. If IOP is >30 mm Hg, assessments will continue every approximately 30 minutes from the previous measurement until the subject passes gross vision test and IOP is ≤30 mm Hg. Note: if the study eye is treated with a ranibizumab or aflibercept injection during the same visit as the study treatment (pegcetacoplan or sham), the treatment with ranibizumab or aflibercept must be performed first. The pre- and post- anti-VEGF IOP values must be measured and recorded on the eCRF.
P. Starting at Day 1, study subjects will be contacted by study site staff within 4 ± 2 days after each study treatment visit (through Month 4) to collect any information on any safety concerns, decrease in vision, eye pain, unusual ocular events, or any new ocular symptoms in the study eye. If the physician determines that there are any safety concerns, a follow-up visit should be scheduled as soon as possible.
Q. Record concomitant medications (ie, prescription and over-the-counter medications) used by the patient within 30 days of screening and throughout the subject's participation in the study.
R. In addition to the time points indicated on the study schedule, OCT-A should be performed at the time of any suspected new active CNV. If new active CNV is confirmed in the study eye, OCT-A should be repeated every 2 months for the study eye.
S. At select sites only.
T. Administration of study treatment (pegcetacoplan or sham) can be done on a separate day from the assessment visit if both days fall within the visit window. If this occurs on the randomization visit, then the administration of pegcetacoplan or sham should be done within 3 days of randomization and after approval from the medical monitor. When study treatment administration is on a day other than a study visit, then the only assessment that must be done on the day of study treatment administration is the preinjection IOP. During the COVID-19 pandemic, the following flexibility is allowed for IP administration: –8 days to +30 days after medical monitor approval.

Table 15 (TRACKED): COVID-19 VISIT SCHEDULE—Every-Other-Month Group—Month 13 to Month 24

	Treatment												Early Term [A]
**Visit #**		**15**		**16**		**17**		**18**		**19**		**20**	
**Day**	**390**	**420**	**450**	**480**	**510**	**540**	**570**	**600**	**630**	**660**	**690**	**720**	
**Week**	**52**	**56**	**60**	**64**	**68**	**72**	**76**	**80**	**84**	**88**	**92**	**96**	
**Month**	**13**	**14**	**15**	**16**	**17**	**18**	**19**	**20**	**21**	**22**	**23**	**24**	
**Window (+ or – days)**		**16**		**16**		**16**		**16**		**16**		**16**	
Informed Consent/Assign Screening Number													
Demographic Data													
Inclusion/Exclusion Criteria [B]													
Medical/Surgical/Ocular History [C]													
Blood Draw—Safety Labs [D,E,F]						~~x~~						x	x
Urine Sample Collection [D, E,F]						~~x~~						x	x
Urine Pregnancy Test [D,E,F]		x		x		x		x		x		x	
Blood Draw—PK and Complement Profile (C3, CH50, AH50) [D,S]												x	x
Blood Draw—Anti-Pegcetacoplan Ab [D]		~~x~~				~~x~~						x	x
Blood Draw—Genotyping (if applicable) [D]													
Blood Draw for Clinical Repository (if applicable) [D,G]												~~x~~	~~x~~
Vital Signs [H]		~~x~~		~~x~~		x		~~x~~		~~x~~		x	x
Physical Examination [I]												x	x
BCVA [J]		x		x		x		x		x		x	x
LL-BCVA [J]		~~x~~		~~x~~		x		~~x~~		~~x~~		x	x
MNREAD or Radner Reading Charts (select countries) [J,K]						~~x~~						x	x
Slitlamp Examination		x		x		x		x		x		x	x
Endothelial Cell Count [S]												x	x
NEI VFQ-25 [L]						~~x~~						x	x
FRI [L]						~~x~~						x	x
Home-Based Digital Applications [L,M,S]						~~x~~						~~x~~	

Table 15 (TRACKED): COVID-19 VISIT SCHEDULE—Every-Other-Month Group—Month 13 to Month 24

	Treatment												Early Term [A]
**Visit #**		**15**		**16**		**17**		**18**		**19**		**20**	
**Day**	**390**	**420**	**450**	**480**	**510**	**540**	**570**	**600**	**630**	**660**	**690**	**720**	
**Week**	**52**	**56**	**60**	**64**	**68**	**72**	**76**	**80**	**84**	**88**	**92**	**96**	
**Month**	**13**	**14**	**15**	**16**	**17**	**18**	**19**	**20**	**21**	**22**	**23**	**24**	
**Window (+ or – days)**		**16**		**16**		**16**		**16**		**16**		**16**	
Dilated Indirect Ophthalmoscopy		x		x		x		x		x		x	x
IOP Measurement		x		x		x		x		x		x	x
SD-OCT [N]		~~x~~		~~x~~		x		~~x~~		~~x~~		x	x
FAF [N]		~~SE~~		~~SE~~		x		~~SE~~		~~SE~~		x	x
NIR [N]		~~SE~~		~~SE~~		x		~~SE~~		~~SE~~		x	x
DCFP [N]												x	x
FFA [N]												x	x
OCT-A [R,S]						~~x~~[R]						x[R]	x
Study Eye Determination													
Randomization													
Pegcetacoplan administration or Sham Injection[T]		x		x		x		x		x			
Postinjection Assessment [O]		x		x		x		x		x			
Follow-Up Call [P]													
Concomitant Medication/Concomitant Ocular Procedures [Q]		x		x		x		x		x		x	x
Adverse Events		x		x		x		x		x		x	x

Abbreviations: AH50 = alternative pathway of complement functional test; BCVA = best corrected visual acuity; CH50 = classical pathway of complement functional test; CNV = choroidal neovascularization; DCFP = digital color fundus photography; eCRF = electronic case report form; FAF = fundus autofluorescence; FFA = fundus fluorescein angiography; FRI = Functional Reading Independence Index; IOP = intraocular pressure; LL-BCVA = low luminance best corrected visual acuity; MNREAD = Minnesota Low-Vision Reading Test; NEI VFG-25 = National Eye Institute Visual Functioning Questionnaire 25-Item Version; NIR = near infrared reflectance; OCT-A = optical coherence tomography angiography; PK = pharmacokinetics; SD-OCT = spectral domain optical coherence tomography; SE = study eye; Term = termination; VEGF = vascular endothelial growth factor.

Note: All ocular assessments are to be performed for both eyes unless annotated with 'SE' (study eye) in the above schedule. All assessments should be performed on the same day. All study visits should be scheduled and projected based on the Day 1 visit date.

A. For subjects that discontinue the study early, the early termination assessments should be performed after a minimum of 30 days have passed from the last dosing visit. If a subject reports for a scheduled visit and decides to terminate early prior to dosing, then the visit should be considered the early termination visit and all early termination procedures should be performed. At Month 24, all subjects should be offered entry into an open label study.
B. At Day 1 (Visit 2), confirm subject eligibility through reviewing the inclusion/exclusion criteria and receive confirmation of eligibility from the reading center.
C. Significant medical/surgical history from the previous 5 years. Anti-VEGF treatments (fellow eye) and invasive ocular procedures performed within the past 5 years and while on study should be recorded. Any history of tobacco use should be recorded.
D. Obtain prior to fluorescein angiography and before study drug administration.
E. At screening, serum pregnancy should be performed for women of childbearing potential. If positive, subject is not eligible to continue in the study.
F. Beginning at Day 1, perform the urine pregnancy test for women of childbearing potential at each treatment visit. If positive, perform a serum pregnancy test. If serum test is positive, study drug should not be administered and an early term visit should be completed.
G. Only subjects that sign the separate consent for the clinical repository and genotyping will have these samples collected. A 14-mL whole-blood sample will be collected at the specified time points.
H. Blood pressure, respiratory rate, heart rate, and temperature. On dosing days, vital signs should be taken pre- dose.
I. Height and weight should be measured at screening.
J. Perform assessments prior to dilating the eyes.
K. In select countries, the MNREAD or Radner Reading Charts should be done during the study visit, prior to dilating the eyes. It should be performed monocularly first, then binocularly.
L. To be administered by the masked site staff prior to any other assessments performed on that day. In-clinic assessments on the digital application should be completed after completion of all functional tests and quality of life measures prior to dilating the eyes.
M. At select sites, and for those subjects who decide to participate, the digital applications will be completed on an electronic device and will consist of assessments for visual function and reading speed. Subjects will receive training at Day 1 on the use of the digital applications and the electronic device and will perform the tests using the application in the clinic at Month 1, Month 2, Month 3, Month 6, Month 12, Month 18, and Month 24. Beginning at Day 1, subjects will complete the functional assessments weekly at home. Subjects should be instructed to complete the assessments on the same day each week and at approximately the same time of day each week, if possible.
N. FAF and SD-OCT images, near infrared reflectance, fluorescein angiograms, and fundus photographs will be performed for the study eye only on days where 'SE' is specified and for both eyes at all other visits as specified in the above schedule and will be sent to the reading center for evaluation (consult the reading center manual for specifics on image capture, processing, and transmission). Images should be captured prior to dosing on dosing days. If a subject misses a study visit or images cannot be obtained at a specific visit, study staff should make every effort to obtain images at the next scheduled visit. If new active CNV is suspected, SD-OCT, FFA, and OCT-A (selected sites) images should be collected and sent to the reading center for analysis.
O. Postinjection assessments should be performed within 5 minutes after dosing by the unmasked physician or study staff and should include a gross assessment of vision (finger-counting, hand motion, then light perception when applicable). If subject passes gross vision test, the subject may leave the site. If subject fails gross vision test, the tonometry should be performed. IOP should be ≤30 mm Hg in order for the subject to leave the site. If necessary, antiglaucomatous medication can be given in order to lower IOP. If IOP is >30 mm Hg, assessments will continue every approximately 30 minutes from the previous measurement until the subject passes the gross vision test and IOP is ≤30 mm Hg. Note: if the study eye is treated with a ranibizumab or aflibercept injection during the same visit as the study treatment (pegcetacoplan or sham), the treatment with ranibizumab or aflibercept must be performed first. The pre- and post- anti-VEGF IOP values must be measured and recorded on the eCRF.
P. Starting at Day 1, study subjects will be contacted by study site staff within 4 ± 2 days after each study treatment visit (through Month 4) to collect any information on any safety concerns, decrease in vision, eye pain, unusual ocular events, or any new ocular symptoms in the study eye. If the investigator determines that there are any safety concerns, a follow-up visit should be scheduled as soon as possible.
Q. Record concomitant medications (ie, prescription and over-the-counter medications) used by the patient within 30 days of screening and throughout the subject's participation in the study.
R. In addition to the time points indicated on the study schedule, OCT-A should be performed at the time of any suspected new active CNV. If new active CNV is confirmed in the study eye, OCT-A should be repeated every 2 months for the study eye.
S. At select sites only.
T. Administration of pegcetacoplan or sham can be done on separate days from the assessment visit if both days fall within the visit window. Administration of study treatment (pegcetacoplan or sham) can be done on a separate day from the assessment visit if both days fall within the visit window. If this occurs on the randomization visit, then the administration of pegcetacoplan or sham should be done within 3 days of randomization and after approval from the medical monitor. When study treatment administration is on a day other than a study visit, then the only assessment that must be done on the day of study treatment administration is preinjection IOP. During the COVID-19 pandemic, the following flexibility is allowed for IP administration: –16 days to +30 days after medical monitor approval.

1.2 Sample of SAS Code

```
* The SAS codes in this section are shown as examples
* Some modifications (such as variables used in the model statement)
* may require further changes to reflect the methods specified in the
* SAP and analysis data structure
* e.g., change trtan to trtpn to reflect analysis w/ the ITT set;
```

1.2.1. Mixed Effect Model for Repeated Measure

```
*-----------------------------------------------------
* Read Analysis data
*-----------------------------------------------------
* In this example, repeated measures are available at
* avisitn = 0.5 (baseline), 2 (Month 2),
*   6 (Month 6), and 12 (Month 12)
*-----------------------------------------------------;

data ga;
 set adam.adga;
 if paramcd = 'SEGAORG' and
 avisitn in (0.5, 2, 6, 12) and
 ANL01FL = 'Y';

  * trtan (actual treatment[number])
  * 1 = APL-2 Monthly
  * 2 = APL-2 Every Other Month
  * 3 = pooled Sham group;
 if trtan in (4,5) then trtan = 3;

 * trt01 are trt02 are used in multiple imputation
 * trt01 = 1 if Sham group
 * trt02 = 1 if APL2 Monthly group
 * trt01 = trt02 = 0 for APL2 EOM group;
 if trtan = 3 then trt01= 1; else trt01 = 0;
  if trtan = 1 then trt02= 1; else trt02 = 0;

 * avisitc will be used in PROC TRANSPOSE;
 if avisitn = 0.5 then avisitc = '00'; * Baseline;
 if avisitn = 2 then avisitc = '02'; * Month 2;
 if avisitn = 6 then avisitc = '06'; * Month 6;
 if avisitn = 12 then avisitc = '12'; * Month 12;
run;

********************************;
*** MAIN ANALYSIS using MMRM ***;
********************************;

proc mixed data=ga method=reml covtest empirical;
  where avisitn NE 0.5; * exclude rows for baseline;
  * cnv (CNV in the fellow eye)
  * 0 = No
  * 1 = Yes;
  *base_cat (baseline lesion size (<7.5 vs. ≥ 7.5);
  class trtan cnv avisitn usubjid base_cat;
  model chg = trtan cnv base_cat avisitn trtan*avisitn base_cat*avisitn
  /cl;
```

```
 repeated avisitn / subject=usubjid type=un r;
 lsmeans trtan*avisitn/pdiff cl e alpha=.05;
 ods output diffs= diff1 LSMeans= LSMean1;
run;
```

1.2.2. Multiple Imputation

```
********************************;
*** RESHAPE DATA FOR PROC MI ***;
********************************;

proc sort data= ga;
 by usubjid cnv trta trtan trt01 trt02 param paramcd base base_cat avisitc;
run;

proc transpose data= ga out= ga_t(drop= _NAME_ _LABEL_) prefix=V;
 by usubjid cnv trta trtan trt01 trt02 param paramcd base base_cat;
 id avisitc;
 var aval;
run;

* Examine the missing patterns of the data;
proc mi data=ga_t nimpute=0;
 var cnv trtan v00 -- v12;
 ods output missPattern=pattern;
run;

****************************************;
*** MCMC (impute nonmonotone missing) ***;
****************************************;

* Below statements invoke MCMC procedure and specify IMPUTE=MONOTONE
 to turn the arbitrary missing patterns to monotone missing patterns
 under missing at random (MAR) assumption;

proc mi data=ga_t out=ga_t_mono seed= 12135541 nimpute=1000;
 mcmc chain=multiple impute= monotone displayinit
     initial=em(itprint);
 var cnv trt01 trt02 v00 -- v12;
run;

* Examine the missing patterns of the data;
proc mi data=ga_t_mono nimpute=0;
 var cnv trt01 trt02 v00 -- v12;
 ods output missPattern=pattern;
run;

*******************************************************************;
*** Control-based pattern imputation(impute monotone missing) ***;
*******************************************************************;
data ga_t_mono2;
      set ga_t_mono;
      **Identify the subjects in the active groups we want to keep
  imputed as MAR;
      **DSCREASN is just an example;
      if trtan in (1,2) and DCSREASN = 1 then mar = 1;
      else mar = 0;
```

```
      run;

**First impute general MAR based on regression method for all subjects to get
the true MAR;
proc mi data=ga_t_mono2 out=ga_step1 nimpute=1 seed=1284054;
 by _Imputation_;
 class trtan;
 monotone reg(v00 -- v12/details);
 var cnv trtan v00 -- v12;
run;

**Identify and keep the data from the subjects in the active group that
should remain as a MAR;
data ga_step2;
      set ga_step1;
      if Mar = 1;
      run;

**Update the dataset and keep the MAR imputed data for the subjects in the
active group that should remain as a MAR, all others remain to be imputed
with control based imputation;
data ga_t_mono3;
      update ga_t_mono2 ga_step2;
      by _Imputation_ subjid;
      run;

**Fill out the remaining missing where missing data should be imputed based
on the controls;
proc mi data=ga_t_mono3 out=ga_t_cbp nimpute=1 seed=1284054;
 by _imputation_;
 class trtan;
 var cnv v00 -- v12;
 monotone reg(/details);
 mnar model(v02 -- v12 / modelobs= (trtan='3'));
run;

**General control based (all monotone missing);
proc mi data=ga_t_mono out=ga_t_cbp2 nimpute=1 seed=1284054;
 by _imputation_;
 class trtan;
 var cnv v00 -- v12;
 monotone reg(/details);
 mnar model(v02 -- v12 / modelobs= (trtan='3'));
run;

*******************************************************************;
*** Delta-Adjusted Pattern Imputation (impute monotone missing) ***;
*******************************************************************;
**First identify the subjects in the active treatment groups that we want to
apply the shift parameter if they have monotone missing data;
data ga_t_mono2;
      set ga_t_mono;

      **This is just an example of a reason that we do not want to
  apply the shift parameter. The actual reasons are in the text;
      if DCSREAS1 = "Death" then flag = 1;
      else flag = 0;
```

```
      if trtan in (1,2) and flag = 0 then adjustthis = 1;
      else adjustthis = 0;
      run;

proc mi data=ga_t_mono2 out=ga_t_tip6 nimpute=1 seed=9484353;
 by _imputation_;
 class trtan adjustthis;
 var cnv trtan v00 -- v12;
 monotone reg(/details);
 mnar adjust(v02 / shift=0.12 adjustobs=(adjustthis = '1' ))
  adjust(v06 / shift=0.12 adjustobs=(adjustthis = '1' ))
  adjust(v12 / shift=0.12 adjustobs=(adjustthis = '1' ));
run;

*****************************************************;
*** SAS macro to run MIANALYZE on outputs from MMRM *;
*****************************************************;

%macro mi_results(in, lsm, dif);

* After imputation, the data sets are in the wide format in which
 different variables (V00--V12) represent the outcome measured at
 different occasions. Before analyzing using PROC MIXED,
 the data sets need to be converted into long format in which one
 variable represents all outcome with different values of AVISITN
 differentiating different occasions;

proc transpose data= &in
    out= ga_mi (rename= (_NAME_ = AVISITN COL1 = AVAL));
 by _imputation_ usubjid trta trtan trt01 trt02 param paramcd base base_cat;
 var V00 -- V12;
run;

data ga_mi;
 set ga_mi;
 label AVISITN = 'AVISITN';
  chg = aval - base;
  * baseline record is not used in MMRM;
 if AVISITN = 'V00' then delete;
run;

proc sort; by _imputation_; run;

proc mixed data=ga_mi method=reml empirical;
 by _imputation_;
 class trtan cnv avisitn usubjid base_cat;
 model chg=trtan cnv base_cat avisitn trtan*avisitn base_cat*avisitn / cl;
 repeated avisitn / subject=usubjid type=UN;
 lsmeans trtan*avisitn / pdiff cl alpha=.05;
 ods output diffs= diffs lsmeans= lsmeans;
run;

* In the final step, the analysis results obtained from PROC MIXED
  procedure are combined into a single estimation with standard error
  using PROC MIANALYZE;
```

```
proc sort data=lsmeans;
 by avisitn trtan _imputation_;
run;
proc mianalyze parms=lsmeans;
 by avisitn trtan;
 modeleffects trtan*avisitn;
 ods output ParameterEstimates=&lsm;
run;

proc sort data=diffs (where= (_trtan= 3 and (avisitn = _avisitn)));
 by avisitn trtan _trtan _imputation_;
run;

proc mianalyze parms=diffs;
 by avisitn trtan _trtan;
 modeleffects trtan*avisitn;
 ods output ParameterEstimates=&dif;
run;

proc sql; *** LSMEANS using Multiple Imputation;
  select Parm as Effect, AVISITN, TRTAN, Estimate, StdErr, DF,
    tValue, Probt, LCLMean as L95, UCLMean as U95
 from &lsm;

  select Parm as Effect, AVISITN, TRTAN, _TRTAN, Estimate, StdErr, DF,
    tValue, Probt, LCLMean as L95, UCLMean as U95
 from &dif;
quit;
%mend;

%mi_results(in= ga_t_mcmc, lsm= lsm_mcmc, dif=dif_mcmc);
%mi_results(in= ga_t_reg, lsm= lsm_reg, dif=dif_reg );
%mi_results(in= ga_t_cbp, lsm= lsm_cbp, dif=dif_cbp );
%mi_results(in= ga_t_tip6, lsm= lsm_tip6, dif=dif_tip6);
```

1.2.3. Rate of Change Models

```
********************************;
*** Rate of Change analyses ***;

* In this example, repeated measures are available at
* avisitn = 0 (baseline), 2 (Month 2), 4 (Month 4)
*   6 (Month 6), 8 (Month 8), 10 (Month 10) and 12 (Month 12)
*
* AVAL is the actual GA lesion measurement at the corresponding visit * for
the study eye from ADGA.
* SGALESAT is the GA lesion size (categorical) at baseline
* FCNVAT is the fellow eye CNV status at baseline
* TR01PG2N is the pooled treatment group (1 = PM, 2 = PEOM, 3 = Sham)
* T is equivalent to AVISITN
*
* The data is read in from ADGA
********************************;


proc mixed data=ga method=reml covtest empirical;
```

```
        class subjid TR01PG2N FCNVAT SGALESAT t;
        model aval = SGALESAT TR01PG2N avisitn TR01PG2N * avisitn
              SGALESAT*avisitn FCNVAT/cl solution;
        repeated t /type=un sub=subjid r ;

        **Month 6;
        estimate 'PM Month 0 to 6 slope' TR01PG2N*avisitn 6 0 0
              avisitn 6 SGALESAT*avisitn 3 3 /cl;
        estimate 'PEOM Month 0 to 6 slope' TR01PG2N*avisitn 0 6 0
              avisitn 6 SGALESAT*avisitn 3 3 /cl;
        estimate 'Sham Month 0 to 6 slope' TR01PG2N*avisitn 0 0 6
              avisitn 6 SGALESAT*avisitn 3 3 /cl;

        estimate 'Month 0 to Month 6 Difference in slope PM - Sham'
              TR01PG2N*avisitn 6 0 -6 /cl;
        estimate 'Month 0 to Month 6 Difference in slope PEOM - Sham'
              TR01PG2N*avisitn 0 6 -6 /cl;
        estimate 'Month 0 to Month 6 Difference in slope PM - PEOM'
              TR01PG2N*avisitn 6 -6 0 /cl;

        **Month 12;
        estimate 'PM Month 6 to 12 slope' TR01PG2N*avisitn 6 0 0
              avisitn 6 SGALESAT*avisitn 3 3 /cl;
        estimate 'PEOM Month 6 to 12 slope' TR01PG2N*avisitn 0 6 0
              avisitn 6 SGALESAT*avisitn 3 3 /cl;
        estimate 'Sham Month 6 to 12 slope' TR01PG2N*avisitn 0 0 6
              avisitn 6 SGALESAT*avisitn 3 3 /cl;

        estimate 'Month 6 to 12 Difference in slope PM - Sham'
              TR01PG2N*avisitn 6 0 -6 /cl;
        estimate 'Month 6 to 12 Difference in slope PEOM - Sham'
              TR01PG2N*avisitn 0 6 -6 /cl;
        estimate 'Month 6 to 12 Difference in slope PM - PEOM'
              TR01PG2N*avisitn 6 -6 0 /cl;

        ods output diffs= diff2 LSMeans= LSMean2 CovParms = COV2
              estimates = estimates2;

        **Month 0 to 12;
        estimate 'PM Month 0 to 12 slope' TR01PG2N*avisitn 12 0 0
              avisitn 12 SGALESAT*avisitn 6 6 /cl;
        estimate 'PEOM Month 0 to 12 slope' TR01PG2N*avisitn 0 12 0
              avisitn 12 SGALESAT*avisitn 6 6 /cl;
        estimate 'Sham Month 0 to 12 slope' TR01PG2N*avisitn 0 0 12
              avisitn 12 SGALESAT*avisitn 6 6 /cl;

        estimate 'Month 0 to Month 12 Difference in slope PM - Sham'
              TR01PG2N*avisitn 12 0 -12 /cl;
        estimate 'Month 0 to Month 12 Difference in slope PEOM - Sham'
              TR01PG2N*avisitn 0 12 -12 /cl;
        estimate 'Month 0 to Month 12 Difference in slope PM - PEOM'
              TR01PG2N*avisitn 12 -12 0 /cl;

run;
```

```
* For the piecewise model, a knot at Month 6 is added to the dataset and
included in the model to allow for different slopes for the two 6 month
periods;

data ga2;
      set ga;

      k = 6;
      if avisitn <= k then avisitnspl1 = 0;
      if avisitn > k then avisitnspl1 = avisitn - k;
run;

proc mixed data=ga2 method=reml covtest empirical;
      class subjid TR01PG2N FCNVAT SGALESAT t;
      model aval = SGALESAT TR01PG2N avisitn avisitnspl1 TR01PG2N *
            avisitn TR01PG2N*avisitnspl1 SGALESAT*avisitn
            SGALESAT*avisitnspl1 FCNVAT/cl ;
      repeated t /type=un sub=subjid r ;

      **Month 6;
      estimate 'PM Month 0 to 6 slope' TR01PG2N*avisitn 6 0 0
            avisitn 6 SGALESAT*avisitn 3 3 /cl;
      estimate 'PEOM Month 0 to 6 slope' TR01PG2N*avisitn 0 6 0
            avisitn 6 SGALESAT*avisitn 3 3 /cl;
      estimate 'Sham Month 0 to 6 slope' TR01PG2N*avisitn 0 0 6
            avisitn 6 SGALESAT*avisitn 3 3 /cl;

      estimate 'Month 0 to Month 6 Difference in slope PM - Sham'
            TR01PG2N*avisitn 6 0 -6 /cl;
      estimate 'Month 0 to Month 6 Difference in slope PEOM - Sham'
            TR01PG2N*avisitn 0 6 -6 /cl;
      estimate 'Month 0 to Month 6 Difference in slope PM - PEOM'
            TR01PG2N*avisitn 6 -6 0 /cl;

      **Month 12;
      estimate 'PM Month 6 to 12 slope' TR01PG2N*avisitn 6 0 0
            avisitn 6 SGALESAT*avisitn 3 3 TR01PG2N*avisitnspl1 6 0 0
            avisitnspl1 6 SGALESAT*avisitnspl1 3 3 /cl;
      estimate 'PEOM Month 6 to 12 slope' TR01PG2N*avisitn 0 6 0
            avisitn 6 SGALESAT*avisitn 3 3 TR01PG2N*avisitnspl1 0 6 0
            avisitnspl1 6 SGALESAT*avisitnspl1 3 3 /cl;
      estimate 'Sham Month 6 to 12 slope' TR01PG2N*avisitn 0 0 6
            avisitn 6 SGALESAT*avisitn 3 3 TR01PG2N*avisitnspl1 0 0 6
            avisitnspl1 6 SGALESAT*avisitnspl1 3 3 /cl;

      estimate 'Month 6 to 12 Difference in slope PM - Sham'
            TR01PG2N*avisitn 6 0 -6 TR01PG2N*avisitnspl1 6 0 -6/cl;
      estimate 'Month 6 to 12 Difference in slope PEOM - Sham'
            TR01PG2N*avisitn 0 6 -6 TR01PG2N*avisitnspl1 0 6 -6/cl;
      estimate 'Month 6 to 12 Difference in slope PM - PEOM'
            TR01PG2N*avisitn 6 -6 0 TR01PG2N*avisitnspl1 6 -6 0/cl;

      ods output diffs= diff2 LSMeans= LSMean2 CovParms = COV2
            estimates = estimates2;
run;
```

1.2.4. Sample Code for Maximum Reading Speed and Critical Print Size

Please see below regarding the uncorrected and corrected reading speed calculation, and Critical Print Size: Flag = 1 MNREAD , 2 = Radner .

```
if flag = 1 then do;
   if (time  = 0 or notdone = 1) then urs = 0;
   /** "Not Done" indicates the print size was not attempted due to vision or
that the sentence was attempted but could not be read **/
   else if time > 0 then urs = 600/time;
   if urs > 300 then urs = 300;
    /** Calculate Corrected Reading Speed **/
   if (time = 0 or numerr >= 10) or (notdone = 1) then crs = 0;
   else if numerr >=0 and time > 0 then crs = 60*(10-numerr)/time;
   else if (numerr = 0 or time = 0) then crs = urs;
   if crs > 300 then crs = 300;
  end;
  if flag = 2 then do;
   if (time  = 0 or notdone = 1) then urs = 0;
   else if time > 0 then urs =  840/time;
   if urs > 300 then urs = 300;
   if (time = 0 or numerr >= 14) or (notdone = 1) then crs = 0;
   else if numerr >=0 and time  > 0 then crs = 60*(14-numerr)/time;
   else if (numerr = 0 or time = 0) then crs = urs;
   if crs > 300 then crs = 300;
end;

Critical Print Size:
if flag = 1 and n(aval,dist) =2 and dist ne 40 then aval = aval +
round(log10(40/dist),.01); /** Apply correction for viewing distance ne 40
**/
if flag = 2 and n(aval,dist) =2 and dist not in (32 40) then aval = aval -
0.1 + round(log10(40/dist),.01);
```

PEGCETACOPLAN (APL-2)

PROTOCOL APL2-304

A PHASE 3, MULTICENTER, RANDOMIZED, DOUBLE-MASKED, SHAM-CONTROLLED STUDY TO COMPARE THE EFFICACY AND SAFETY OF INTRAVITREAL PEGCETACOPLAN THERAPY WITH SHAM INJECTIONS IN PATIENTS WITH GEOGRAPHIC ATROPHY (GA) SECONDARY TO AGE-RELATED MACULAR DEGENERATION (AMD)

US IND No.: 124784

EudraCT No.: 2018-001435-52

Phase: 3

Version: Amendment 5

Date: 12 August 2020

Confidentiality Statement

This document is confidential. It contains proprietary information of Apellis Pharmaceuticals, Inc. Any viewing or disclosure of such information that is not authorized in writing by Apellis Pharmaceuticals, Inc, is strictly prohibited. Such information may be used solely for the purpose of reviewing or performing this study.

INVESTIGATOR AGREEMENT

**Long Title:**	A Phase 3, Multi-Center, Randomized, Double-Masked, Sham-Controlled Study to Compare the Efficacy and Safety of Intravitreal Pegcetacoplan Therapy with Sham Injections in Patients with Geographic Atrophy (GA) Secondary to Age-Related Macular Degeneration (AMD)
**Short Title:**	Oaks
**Protocol Number, Version, and Date:**	APL2-304 / Amendment 5 / 12 August 2020
**Study Phase:**	Phase 3
**Sponsor Name and Address:**	Apellis Pharmaceuticals, Inc<br>100 5th Ave<br>Waltham, MA 02451<br>USA
**Investigational Test Article:**	Pegcetacoplan intravitreal injection (also known as APL-2)
**US IND Number:**	124784
**EudraCT Number:**	2018-001435-52
**Indication Studied:**	Geographic atrophy secondary to age-related macular degeneration
**Investigator Agreement:**	I have read the clinical study described herein, recognize its confidentiality, and agree to conduct the described trial in compliance with Good Clinical Practice (GCP), the ethical principles contained within the Declaration of Helsinki, this protocol, and all applicable regulatory requirements.

Principal Investigator:

Name: ____________________________________

Signature: ________________________________

Date: ______/______/______ (DD/MMM/YYYY)

SPONSOR INFORMATION

Sponsor

Apellis Pharmaceuticals, Inc.
100 5th Ave
Waltham, MA 02451
USA

Sponsor Representative

PPD
PPD
Tel: PPD
Email: PPD

Signature: PPD ____________________

Date PPD (DD/MMM/YYYY)

PROTOCOL AMENDMENT SUMMARY OF CHANGES

Amendment 5: Summary of Changes From the Previous Version

Amendment Date: 12 August 2020

Updates to the protocol implemented in this amendment are provided in the table below.

Description of change	Section(s) affected by change
Nonsubstantial changes that did not impact content of the document have been made for clarity.	Entire document
Updated company address	Investigator Agreement and Sponsor Information
Changed APL-2 to International Nonproprietary Name: pegcetacoplan, updated related abbreviations and protocol title	Entire document
Added the descriptor "maximum" when referencing reading speed	Synopsis, Section 5.1.2, Section 5.1.5, Section 12.7.2, Section 12.7.3
Removed the 6-month follow-up period and updated the study length from 30 months to 24 months and made it a 30- or 60-day follow-up period based on treatment group because that length of time is deemed sufficient to evaluate the safety of pegcetacoplan based on its half-life in the vitreous. Additionally, there is an option for subjects to enroll in a separate extension study (Study APL2-GA-305) during which longer-term safety and efficacy will be collected.	Synopsis, Section 8, Section 8.1.3.2.1, Section 11.8
Inclusion criterion 6c was updated from "Reliability test ratio must be ≤20%" to "Fixation losses must be ≤20%" on the basis of regulatory feedback provided to the microperimetry device manufacturer	Synopsis, Section 6.1.1
Updated the text regarding the sample size to both simplify and reduce redundancies	Synopsis, Section 12.5
Previous Section 4.1.3.1, Pharmacokinetics, moved to Section 4.1.3.2 to follow Section 4.1.3.1, Toxicology	Section 4.1.3.1, Section 4.1.3.2
Updated the formulation information	Section 4.1.2, Section 7.3.3.1
Revised number of injections for each treatment arm because the injections at Month 24 will no longer be administered	Section 7.3.1 (Table 1), Section 8
Updated the text regarding endophthalmitis for clarity	Section 7.5.3
Updated Study Schema	Section 8
Revised Study Procedures based on the removal of the Month 24 injection and the 6-month follow-up period	Section 8
Revised the language regarding genotyping samples to indicate that these samples will be collected only for subjects who consent to the analysis	Section 9.7
Updated the language within the Adverse Events definition section to be consistent with the new Apellis standard language	Section 11.1
Updated the language within the Recording Adverse Events section to be consistent with the new Apellis standard language	Section 11.2

Removed the section on Treatment and Follow-up of Adverse Events because the information contained within it is now included in other revised sections to align with new Apellis standard language	Section 11.3
Updated the language within the Reporting Adverse Events section to be consistent with the new Apellis standard language	Section 11.3
Updated the language within the Serious Adverse Events section to be consistent with the new Apellis standard language	Section 11.4
Removed the section on Unexpected Adverse Events or Unexpected Suspected Adverse Reactions because the information contained within it is now included in other revised sections to align with new Apellis standard language	previously Section 11.6
Updated the language within the Pregnancy section to be consistent with the new Apellis standard language	Section 11.6
Removed APL-2 investigator's brochure from references	Section 15
Updated Footnote G and M	Appendix A, Appendix B, Appendix C, Appendix D, and Appendix K
Deleted follow-up visits from Schedules of Activities	Appendix B and Appendix D, and Table B and Table D of Appendix K
Deleted pegcetacoplan and sham administration and postinjection assessment from Visit 20 of the Schedule of Activities for monthly and every-other-month treatment groups Month 13 to Month 24	Appendix B and Appendix D and Tables B and D of Appendix K

1. TABLE OF CONTENTS

INVESTIGATOR AGREEMENT....2
SPONSOR INFORMATION....3
PROTOCOL AMENDMENT SUMMARY OF CHANGES....4
1. TABLE OF CONTENTS....6
LIST OF TABLES....11
LIST OF FIGURES....11
2. SYNOPSIS....12
3. ABBREVIATIONS....20
4. INTRODUCTION....23
4.1. Background....23
4.1.1. Age-Related Macular Degeneration....23
4.1.2. Pegcetacoplan....23
4.1.3. Nonclinical Data....24
CCI [redacted]
4.1.4. Clinical Data....27
4.1.5. Rationale....28
4.1.5.1. Rationale for Pegcetacoplan for Treatment of Geographic Atrophy....28
4.1.6. Dose Selection....28
4.2. Risk/Benefit....28
5. STUDY OBJECTIVES....30
5.1.1. Primary Objective....30
5.1.2. Key Secondary Objectives....30
5.1.3. Secondary Objectives....30
5.1.4. Safety Objectives....30
5.1.5. Exploratory Objectives....31
6. PATIENT POPULATION....32
6.1.1. Inclusion Criteria....32

6.1.2. Exclusion Criteria 33
6.2. Women of Childbearing Potential 34
6.3. Women of Nonchildbearing Potential 34
6.4. Approved Methods of Contraception 34
6.5. Discontinuation of Subjects 35
7. TREATMENT OF SUBJECTS 36
7.1. Allocation to Treatment 36
7.2. Masking and Minimization of Bias 36
7.2.1. Unmasking 37
7.3. Dosage and Administration 37
7.3.1. Dose Levels and Treatment Arms 37
7.3.2. Treatment Administration 37
7.3.3. Drug Supplies 38
7.3.3.1. Identity of Investigational Product 38
7.3.3.2. Storage 38
7.3.3.3. Accountability 38
7.3.4. Intravitreal Pegcetacoplan Administration 39
7.4. Sham Injection Administration 39
7.5. Concomitant Therapies 39
7.5.1. Treatment of New Exudation Related to Active Choroidal Neovascularization in the Study Eye and/or Fellow Eye 39
7.5.2. Prohibited Therapies 40
7.5.3. Endophthalmitis Treatment 40
8. STUDY PROCEDURES 42
8.1. Study Visit Schedule 43
8.1.1. Screening Period—Within 28 Days Prior to Randomization/ Treatment (Day –28 to –1) 43
8.1.1.1. Visit 1—All Subjects 43
8.1.2. Randomization/ Initial Treatment—Day 1—Within 28 Days of Screening 44
8.1.2.1. Visit 2—All Groups 44
8.1.3. Treatment Phase—24 Months 44
8.1.3.1. Months 1-12 44

8.1.3.2. Months 13-24 45
8.1.4. Early Termination Visit 46
8.1.5. Unscheduled Visits 46
9. ASSESSMENTS 47
9.1. Informed Consent 47
9.2. Demographic Information/ Medical/ Surgical History 47
9.3. Ocular History/ Ocular Procedures 47
9.4. Vital Signs 47
9.5. Physical Examination 47
9.6. Laboratory Analysis of Blood and Urine 47
9.7. Genotyping Samples 48
9.8. Urine Pregnancy Test 48
9.9. Patient-Reported Outcomes 49
9.9.1. The National Eye Institute Visual Functioning Questionnaire 25-Item Version (NEI-VFQ) 49
9.9.2. The Functional Reading Independence (FRI) Index 49
9.10. Best Corrected Visual Acuity and Low Luminance Best Corrected Visual Acuity 49
9.11. Minnesota Low-Vision Reading (MNREAD) Test or Radner Reading Charts (in select countries) 50
9.12. Home-Based Functional Digital Applications (optional, select sites) 50
9.13. Complete Ophthalmic Exam 50
9.14. Ocular Imaging and Microperimetry 50
9.15. Postinjection Assessment 51
9.16. Blood Volume for Study Assessments 52
9.17. Samples for Clinical Repository (Optional, Select Sites) 52
9.17.1. Approval by the Institutional Review Board or Ethics Committee 53
9.17.2. Sample Collection 53
9.17.3. Confidentiality and Data Ownership 53
9.17.4. Consent to Participate in the Clinical Repository 54
9.17.5. Withdrawal From the Clinical Repository 54
9.17.6. Monitoring and Oversight 54
10. SAFETY EVALUATIONS 55

10.1. Data Monitoring Committee 55
11. ADVERSE EVENTS 56
11.1. Definition 56
11.2. Recording Adverse Events 56
11.3. Reporting Adverse Events 57
11.3.1. Relationship of Events to Study Treatment 57
11.3.2. Severity of Events 57
11.4. Serious Adverse Events 58
11.5. Treatment and Follow-up of Adverse Events 58
11.6. Pregnancy 59
11.7. Disease Progression 59
11.8. Withdrawal 59
12. DATA MANAGEMENT AND STATISTICAL CONSIDERATIONS 60
12.1. Data Collection 60
12.2. Clinical Data Management 60
12.3. Statistical Analysis Process 60
12.4. Planned Interim Analysis and Data Safety Monitoring Committee 60
12.5. Sample Size Calculation and Power Considerations 61
12.6. Statistical Analysis Sets 61
12.7. Efficacy Analyses 62
12.7.1. Primary Efficacy Analysis 62
12.7.2. Secondary Efficacy Analysis 63
12.7.3. Exploratory Efficacy Analysis 64
12.8. Safety Analyses 64
12.8.1. Anti-Therapeutic Antibodies 65
12.8.2. Death 65
12.8.3. Ocular Assessments 65
12.9. Handling of Missing Data 65
12.10. Visit Windows 65
13. ETHICS 66
13.1. Ethical Conduct of the Study 66
13.2. Institutional Review Board/Ethic Committee 66
13.3. Subject Information and Consent 66

14. ADMINISTRATIVE CONSIDERATIONS 67
14.1. Direct Access to Source Data/Documents 67
14.2. Quality Control and Quality Assurance 67
14.3. Monitoring 67
14.4. Data Handling and Record Keeping 67
14.5. Protocol Amendments 68
14.6. Report Format 68
14.7. Finance and Insurance 68
14.8. Publication Policy 68
14.9. ClinicalTrials.gov 69
14.10. Termination of Study 69
15. REFERENCES 70
16. APPENDICES 72
APPENDIX A: VISIT SCHEDULE—Monthly Group—Screening, Day 1 through Month 12 72
APPENDIX B: VISIT SCHEDULE—Monthly Group—Month 13 to Month 24 75
APPENDIX C: VISIT SCHEDULE—Every-Other-Month Group—Screening, Day 1 Through Month 12 78
APPENDIX D: VISIT SCHEDULE—Every-Other-Month Group—Month 13 to Month 24 81
APPENDIX E: NATIONAL EYE INSTITUTE VISUAL FUNCTIONING QUESTIONNAIRE 25-ITEM VERSION 84
APPENDIX F: FUNCTIONAL READING INDEPENDENCE INDEX (FRI) 101
APPENDIX G: MINNESOTA LOW-VISION READING TEST (MNREAD) AND RADNER READING CARDS 102
APPENDIX H: GRADING SCALE FOR ASSESSMENT OF ANTERIOR CHAMBER FLARE OR CELLS 103
APPENDIX I: GRADING SCALE FOR ASSESSMENT OF VITREOUS CELLS 105
APPENDIX J: AMENDMENT HISTORY 106
APPENDIX K: PROTOCOL CHANGES TO BE FOLLOWED DURING COVID-19 RESTRICTIONS 118

LIST OF TABLES

Table 1: Treatment Arms with Approximate Number of Subjects 37
Table 2: Laboratory Sampling and Analysis 48
Table 3: Total Study Blood Volume 52
Table 4: Power to Detect a Difference Among 3 Groups With an Equal Size of 200 Subjects 61

LIST OF FIGURES

Figure 1: Study Schema 42

2. SYNOPSIS

Study Title

A Phase 3, Multicenter, Randomized, Double-Masked, Sham-Controlled Study to Compare the Efficacy and Safety of Intravitreal Pegcetacoplan Therapy With Sham Injections in Patients With Geographic Atrophy (GA) Secondary to Age-Related Macular Degeneration (AMD)

Protocol Number, Version, and Date

APL2-304/ Amendment 5/ 12 August 2020

Investigational Product, Dose, and Route of Administration

- Pegcetacoplan (also known as APL-2)
- 15 mg/0.1 mL
- Intravitreal (IVT) Injection

Study Arms

Arm	Abbreviation	Randomization
Pegcetacoplan 15 mg/0.1 mL monthly for 24 months	PM	2
Pegcetacoplan 15 mg/0.1 mL every other month for 24 months	PEOM	2
Sham monthly for 24 months	SM	1
Sham every other month for 24 months	SEOM	1

Study Phase and Type

Phase 3, multicenter, randomized, double-masked, sham-injection controlled

Number of Subjects and Sites

- Approximately 600 subjects
- Approximately 100 sites

Objectives

Primary

To evaluate the efficacy of pegcetacoplan compared to sham injection in patients with GA secondary to AMD assessed by change in the total area of GA lesions from baseline as measured by FAF.

Key Secondary

To evaluate the efficacy of pegcetacoplan compared to sham injection in patients with GA secondary to AMD with respect to:

- Monocular maximum reading speed (study eye), as assessed by Minnesota Low-Vision Reading Test (MNREAD) or Radner Reading Charts (in select countries)
- Functional Reading Independence (FRI) index score
- Normal luminance best corrected visual acuity score (NL-BCVA) in the study eye

Secondary:

- To evaluate the efficacy of pegcetacoplan compared to sham injection in patients with GA secondary to AMD with respect to:
  - Low luminance best corrected visual acuity score (LL-BCVA) in the study eye
  - Low luminance deficit (LLD) in the study eye
  - Total area of GA lesion(s) in the study eye
  - Monocular critical print size (study eye), as assessed by MNREAD or Radner Reading Charts (in select countries)
  - National Eye Institute Visual Functioning Questionnaire 25 Item Version (NEI VFQ-25) distance activity subscale score (in select countries)
  - Macular functional response as assessed by mesopic microperimetry

Safety:

- To evaluate the safety and tolerability of pegcetacoplan compared to sham injection in patients with GA secondary to AMD as indicated by:
  - Incidence and severity of ocular and systemic treatment-emergent adverse events
  - Incidence of anti-therapeutic antibodies directed against pegcetacoplan
  - Incidence of new active CNV in the study eye

Exploratory

- To evaluate the efficacy of pegcetacoplan compared to sham injection in patients with GA secondary to AMD as indicated by:
  - NEI VFQ-25 composite score
  - NEI VFQ-25 near activity subscale score (in select countries)
  - Comparison between study eye and fellow eye in change in GA lesion size
  - To evaluate the binocular maximum reading speed as assessed by MNREAD or Radner Reading Charts (in select countries)
  - To evaluate the binocular critical print size as assessed by MNREAD or Radner Reading Charts (in select countries)
- To evaluate the relationship between genetic polymorphisms associated with AMD with GA progression and response to pegcetacoplan
- To evaluate the incidence of new onset of subclinical CNV in the study eye
- To assess sensitivity and specificity of a digital reading speed application to detect disease progression / regression (optional, select sites)
- To assess sensitivity and specificity of a digital visual function application to detect disease progression / regression (optional, select sites)

Inclusion Criteria

The study eye must meet all inclusion criteria. If both eyes meet the inclusion criteria, the eye with the worst visual acuity at the screening visit will be designated as the study eye. If both eyes have the same visual acuity, the right eye will be selected as the study eye.

Ocular-specific inclusion criteria apply to the **study eye** only, unless otherwise specified.

1. Age ≥60 years.
2. Normal Luminance best corrected visual acuity of 24 letters or better using Early Treatment Diabetic Retinopathy Study (ETDRS) charts (approximately 20/320 Snellen equivalent).
3. Clinical diagnosis of GA of the macula secondary to AMD as determined by the investigator and confirmed by the reading center.
4. The GA lesion must meet the following criteria as determined by the central reading center's assessment of fundus autofluorescence (FAF) imaging at screening:
    a. Total GA area must be ≥2.5 and ≤17.5 mm^2 (1 and 7 disk areas [DA] respectively)
    b. If GA is multifocal, at least 1 focal lesion must be ≥1.25 mm^2 (0.5 DA), with the overall aggregate area of GA as specified above in 4a.
    c. The entire GA lesion must be completely visualized on the macula centered image and must be able to be imaged in its entirety and not contiguous with any areas of peripapillary atrophy.
    d. Presence of any pattern of hyperautofluorescence in the junctional zone of GA. Absence of hyperautofluorescence (ie, pattern = none) is exclusionary.[1]
5. Adequate clarity of ocular media, adequate pupillary dilation, and fixation to permit the collection of good quality images as determined by the investigator.
6. Meets the following criteria related to microperimetry:
    a. Able to detect fixation target.
    b. Total elapsed time to complete the 10-2 68-point exam is ≤30 minutes in duration.
    c. Fixation losses must be ≤20%.
    d. Subject is willing and able to undertake microperimetry assessment in the opinion of the investigator.
7. Female subjects must be:
    a. Women of non–childbearing potential (WONCBP), or
    b. Women of childbearing potential (WOCBP) with a negative serum pregnancy test at screening and must agree to use protocol defined methods of contraception for the duration of the study and refrain from breastfeeding for the duration of the study.
8. Males with female partners of childbearing potential must agree to use protocol defined methods of contraception and agree to refrain from donating sperm for the duration of the study.
9. Willing and able to give informed consent and to comply with the study procedures and assessments.

Exclusion Criteria

Ocular specific exclusion criteria apply to the **study eye** only, unless otherwise specified.

1. GA secondary to a condition other than AMD such as Stargardt disease, cone rod dystrophy, or toxic maculopathies like plaquenil maculopathy in either eye.

2. Spherical equivalent of the refractive error demonstrating > 6 diopters of myopia or an axial length >26 mm.

3. Any history or active choroidal neovascularization (CNV), associated with AMD or any other cause, including any evidence of retinal pigment epithelium rips or evidence of neovascularization anywhere based on SD-OCT imaging and/or fluorescein angiography as assessed by the reading center.

4. Presence of an active ocular disease that in the opinion of the investigator compromises or confounds visual function, including but not limited to, uveitis, other macular diseases (eg, clinically significant epiretinal membrane [ERM], full thickness macular hole or uncontrolled glaucoma/ocular hypertension). Benign conditions in the opinion of the investigator such as peripheral retina dystrophy are not exclusionary.

5. Intraocular surgery (including lens replacement surgery) within 3 months prior to randomization.

6. History of laser therapy in the macular region.

7. Aphakia or absence of the posterior capsule. Note: YAG laser posterior capsulotomy for posterior capsule opacification done at least 60 days prior to screening is not exclusionary.

8. Any ocular condition other than GA secondary to AMD that may require surgery or medical intervention during the study period or, in the opinion of the investigator, could compromise visual function during the study period.

9. Any contraindication to IVT injection including current ocular or periocular infection.

10. History of prior intravitreal injection.

11. Unable to perform microperimetry reliably in the opinion of the investigator.

12. Prior participation in another interventional clinical study for intravitreal therapies in either eye (including subjects receiving sham).

13. Prior participation in another interventional clinical study for geographic atrophy in either eye including investigational oral medication and placebo.

14. Participation in any systemic experimental treatment or any other systemic investigational new drug within 6 weeks or 5 half-lives of the active ingredient (whichever is longer) prior to the start of study treatment. Note: clinical trials solely involving observation, over-the-counter vitamins, supplements, or diets are not exclusionary.

15. Medical or psychiatric conditions that, in the opinion of the investigator, make consistent follow-up over the 24-month treatment period unlikely, or would make the subject an unsafe study candidate.

16. Any screening laboratory value (hematology, serum chemistry or urinalysis) that in the opinion of the investigator is clinically significant and not suitable for study participation.

17. Known hypersensitivity to fluorescein sodium for injection or hypersensitivity to pegcetacoplan or any of the excipients in pegcetacoplan solution.

Study Design

This is a 24-month, Phase 3, multicenter, randomized, double-masked, sham-injection controlled study to assess the efficacy and safety of multiple IVT injections of pegcetacoplan in subjects with GA secondary to AMD.

The study will randomize approximately 600 subjects across approximately 100 multinational sites. Subjects will be screened within 28 days before receiving pegcetacoplan or sham injection. Upon entry into the study, subjects will be assigned a screening number. Subjects who meet all inclusion and none of the exclusion criteria will return to the clinic for randomization and treatment on Visit 2 (Day 1). At this visit, subjects will be randomized 2:2:1:1 to

receive pegcetacoplan monthly, pegcetacoplan every other month, sham injection monthly, or sham injection every other month, respectively. Randomization will be stratified according to GA lesion area at screening (< 7.5 mm^2; ≥ 7.5 mm^2), and presence of CNV in the fellow eye.

All subjects will be assessed monthly during the first 12 months regardless of treatment regimen. From Month 12 to Month 24, subjects will follow the outlined visit schedule (Appendix A to Appendix D) based on treatment assignment (ie, subjects in the monthly groups will be assessed monthly while subjects in the every-other-month [EOM] groups will be assessed every other month). The last visit in the study will be at Month 24, approximately 30 days (monthly treatment group) or 60 days (EOM treatment group) after the last visit at which investigational product is administered. At the end of the 24-month study period, subjects will have the option to enroll into a separate open-label study.

Subjects who discontinue study treatment can continue participation in the study and should be encouraged to return to the clinical site for as many follow-up visits as they can (with the exception of pegcetacoplan/sham administration). Subjects who wish to fully withdraw from the study before Month 24, should be encouraged to complete the early termination visit.

Endpoints and statistical analysis:

Endpoints

Primary Efficacy Endpoint

- Change from baseline to Month 12 in total area of GA lesion(s) in the study eye (in mm^2) based on fundus autofluorescence (FAF).

Key Secondary Efficacy Endpoints

- Change from baseline in monocular maximum reading speed (study eye), as assessed by MNREAD or Radner Reading Charts at Month 24 (in select countries)
- Change from baseline in Functional Reading Independence (FRI) index score, at Month 24.
- Change from baseline in normal luminance best corrected visual acuity score (NL-BCVA) at Month 24 as assessed by ETDRS chart.

Secondary Efficacy Endpoints

- Change from baseline in low luminance best corrected visual acuity score (LL-BCVA) at Month 12 and Month 24 as assessed by ETDRS chart.
- Change from baseline in low luminance deficit (LLD) at Month 12 and Month 24.
- Change from baseline at each planned assessment in the total area of GA lesion(s) in the study eye (in mm^2) as assessed by FAF.
- Change from baseline in monocular critical print size (study eye), as assessed by MNREAD or Radner Reading Charts, at Month 12 and Month 24 (in select countries).
- Change from baseline in the National Eye Institute Visual Functioning Questionnaire 25-Item Version (NEI VFQ-25) distance activity subscale score at Month 12 and Month 24 (in select sites).
- Number of scotomatous points assessed by mesopic microperimetry for the evaluation of the macular functional response.

- Change in macular sensitivity as assessed by mesopic microperimetry for the evaluation of the macular functional response.

Exploratory Endpoints

- Change from baseline in NEI VFQ-25 composite score at Month 12 and Month 24.
- Change from baseline in NEI VFQ-25 near activity subscale score at Month 12 and Month 24 (in select countries).
- Comparison between study eye and fellow eye in change in GA lesion size from baseline to Month 12 and Month 24.
- Binocular maximum reading speed as assessed by MNREAD or Radner Reading Charts over time (in select countries).
- Binocular critical print size as assessed by MNREAD or Radner Reading Charts over time (in select countries).
- Relationship between genetic polymorphisms associated with AMD with GA progression and response to pegcetacoplan.
- Incidence of new onset of subclinical CNV in the study eye.
- Assess sensitivity and specificity of a digital reading speed application to detect disease progression / regression (optional, select sites).
- Assess sensitivity and specificity of a digital visual function application to detect disease progression / regression (optional, select sites).

Safety Endpoints

- Incidence and severity of ocular and systemic treatment-emergent adverse events.
- Incidence of anti-therapeutic antibodies directed against pegcetacoplan.
- Incidence of new active CNV in the study eye.

Analysis of Primary Efficacy Endpoint

The primary efficacy endpoint is the change from baseline to Month 12 in the total area of GA lesion(s) in eyes injected with pegcetacoplan, either monthly (PM) or every-other month (PEOM), or sham injections. GA lesion area (mm^2) as measured by a quantified central reading center based on FAF images. The primary analysis will be the comparison of pegcetacoplan, either monthly (PM) or every-other month (PEOM) versus the combined 2 sham arms (the 2 sham arms will be combined into a single 'control' group).

The null and alternative hypotheses for the primary efficacy analysis are:

H_0: $\mu_S = \mu_{PM}$ vs H_A: $\mu_S \neq \mu_{PM}$, and

H_0: $\mu_S = \mu_{PEOM}$ vs H_A: $\mu_S \neq \mu_{PEOM}$

Note: here μ indicates each group's respective mean change from baseline to Month 12 in GA lesion area for the comparison of the primary endpoint.

A mixed effect model for longitudinal data will be used to analyze the change from baseline in GA lesion area. The model will include treatment, and presence of CNV in the fellow eye as fixed effects; baseline GA lesion area (at screening), time (in months) as a factor; as well as the time × treatment interaction term. All available data up to 12

months will be included in the model for the primary analysis. The mean change from baseline to 12 months will be estimated from the model (ie, least square [LS] mean) and compared between each of the pegcetacoplan arms to the sham control. For other time point of interest, LS mean change from baseline will be estimated and compared between treatments. Unstructured variance covariance will be used,

For the analysis of final study data, a similar model including data up to 24 months will be used and LS means at time points of interest will be estimated and compared between treatment.

This study is expected to have an approximately 5 DMC data reviews. Allocating an alpha level of 0.0001 for each DMC data review, the alpha level remains for the efficacy analysis at 0.0495 to maintain an overall study alpha of 0.05.

The hypothesis testing strategy for the primary and secondary efficacy endpoints will be based on the Gate-keeping multiple testing procedures controlling for the study wide type I error strongly at 2-sided 0.0495 as follows:

Step 1. The mean GA lesion growth at 12 months will be compared between the PM group and the Control at the α level of 0.0495. If the null hypotheses of no difference between groups in this step is rejected, the testing proceeds to **Step 2** and **Step 3**. If it's not rejected, the testing procedure stops at this step.

Step 2. The mean GA lesion growth at 12 months will be compared between the PEOM group and the Control at the **α1** level. If the null hypotheses of no difference between groups in this step is rejected, the **α1** level will be passed down to **Step 3**. The actual value of **α1** will be specified in the SAP and it will be defined to ensure an adequate power of at least 80% for the comparison in this step.

Step 3. The mean GA lesion growth at 24 months will be compared between the PM group and the Control at the α level of **0.0495** if the null hypotheses are rejected at both **Step 1** and **Step 2**; or at the α level of **(0.0495 – α1)** if **Step 2** testing does not reject the null hypothesis. If the null hypothesis at this step is rejected, the α level used at this step will be passed down to the next step of testing. If it's not rejected, the testing procedure stops at this step.

Step 4. The prioritization and alpha allocation for the remaining secondary endpoints will be specified in the SAP.

The following sensitivity and supportive analyses will be performed to evaluate the robustness of the results from the primary analysis method:

- Analyses will be repeated using the mITT and per-protocol sets
- Primary and secondary endpoints will also be summarized with no pooling of the 2 sham arms. The comparison for pegcetacoplan and sham injection within each dose regimen (ie, PM vs SM and PEOM vs SEOM) will be conducted
- Multiple imputation (MI) methods and other sensitivity analyses will be explored, and details will be provided in the SAP

Analysis for Key Secondary and Secondary Endpoint(s)

The key secondary and secondary endpoints will be analyzed in the same fashion as the primary endpoint using mixed effect model. The binary secondary endpoints will be analyzed using Cochran-Mantel-Haenszel test stratified by the randomization stratification factors. Hypothesis testing for 3 key secondary endpoints will be performed in the order specified. The SAP will provide more details on multiplicity adjustment and the approach for alpha spending among the endpoints.

Sample Size Justification

Subjects will be randomized in a 2:2:1:1 ratio to receive treatment with pegcetacoplan monthly, pegcetacoplan every other month, sham monthly, or sham every other month. The annual growth rate in GA lesion area is expected to have a mean of 1.47, 1.70 and 2.13 mm^2/year for pegcetacoplan monthly, pegcetacoplan every-other-month, and sham-pooled groups, respectively, as estimated from the results of a Phase 2 trial for pegcetacoplan. The standard deviation of the lesion growth is estimated to be 1.50 mm^2 based on the same Phase 2 trial data or 1.25 mm^2 based on natural history data.[27] ie, With a sample size of 200 subjects in each group (ie, a total enrollment of 600 subjects) and the observed annual growth rate in GA lesion area from Phase 2 trial for pegcetacoplan, the study will have 99.2% power to show the difference between PM and sham group and 81.5% power to show the difference between PEOM and sham group using the common standard deviation of 1.5 mm^2 and 2-sided alpha of 0.05. The approximation is calculated using PROC POWER one-way analysis of variance, SAS 9.4. The study power is likely larger when utilizing the longitudinal data to model the primary endpoint. The actual study power may also vary based on the distribution of the stratification factors (ie, lesion area at screening, presence of CNV in fellow eye), and site enrollment.

3. ABBREVIATIONS

Abbreviation	Term
AE	adverse event
AMD	age-related macular degeneration
ATA	anti-therapeutic antibodies
BCVA	best corrected visual acuity
CH50	classical pathway of complement functional test
CNV	choroidal neovascularization
CRF (eCRF)	case report form (electronic CRF) (used interchangeably)
DA	disk area
DCFP	digital color fundus photography
DMC	data monitoring committee
EDC	electronic data capture
EOM	every other month
ERG	electroretinography
ERM	epiretinal membrane
ETDRS	Early Treatment Diabetic Retinopathy Study
FAF	fundus autofluorescence
FFA/FA	fundus fluorescein angiography or fluorescein angiography
FRI	functional reading independence index
FSH	follicle-stimulating hormone
GA	geographic atrophy
GCP	Good Clinical Practice
HCG	human chorionic gonadotropin
IB	investigator's brochure
ICF	informed consent form<br>form
ICH	International Council for Harmonisation
IEC	independent ethics committee

Abbreviation	Term
IOP	intraocular pressure
IRB	institutional review board
ITT	intent-to-treat
IV	intravenous
IVT	intravitreal
IWR	interactive web response
LH	luteinizing hormone
LL-BCVA	low luminance best corrected visual acuity
LLD	low luminance deficit
MedDRA	Medical Dictionary for Regulatory Activities
mITT	modified intention to treat
MNREAD	Minnesota Low-Vision Reading Test
MOP	manual of procedures
NEI VFQ-25	National Eye Institute Visual Functioning Questionnaire 25-Item Version
NIR	near infrared reflectance
NOEL	no observable effect level
NL-BCVA	normal luminance best corrected visual acuity
OCT	optical coherence tomography
OCT-A	optical coherence tomography angiography
PEG	polyethylene glycol
PEG40	polyethylene glycol (40-kDa nominal molecular weight)
PEOM	pegcetacoplan every-other-month
PI	principal investigator
PK	pharmacokinetics
PM	pegcetacoplan monthly
PP	per protocol
RPE	retinal pigment epithelium
SAE	serious adverse event
SC	subcutaneous

Abbreviation	Term
SC5b-9	soluble terminal complement complex
SD-OCT	spectral domain optical coherence tomography
SEOM	sham every-other-month
SM	sham monthly
SOP	standard operating procedures
TEAE	treatment-emergent adverse event
$t_{1/2}$	terminal elimination half-life
VA	visual acuity
VEGF	vascular endothelial growth factor
WOCBP	women of childbearing potential
WONCBP	women of nonchildbearing potential

4. INTRODUCTION

4.1. Background

This study is being conducted as part of a series of studies for the clinical development of pegcetacoplan for advanced Age-related Macular Degeneration (AMD) (neovascular AMD and geographic atrophy [GA]). The trial will be conducted in compliance with this protocol, Good Clinical Practice (GCP), and applicable regulatory requirements. The subject population will comprise adult male and female subjects with GA secondary to AMD.

4.1.1. Age-Related Macular Degeneration

Age-related macular degeneration is the leading cause of severe vision loss in people over the age of 65 in the United States and other Western countries.[2] In the United States, about 1.75 million people have the advanced forms of AMD.[3] The early signs of AMD (drusen and pigmentary changes) are common in individuals over age 65 and precede the late stage forms, which are visually devastating. The late stage forms of AMD are classified into either macular neovascularization (neovascular, wet, or exudative AMD) or GA.

Geographic Atrophy is a disease characterized by thinning and loss of the retinal pigment epithelium (RPE) and concurrent atrophy of photoreceptors and choriocapillaris.[4,5,6] Clinically, GA is characterized by gradually expanding atrophy leaving islands of dead retinal cells in the back of the eye. Although GA can result in significant visual function deficits in reading, night vision, and dark adaptation, and produce dense, irreversible scotomas in the visual field, the initial decline in visual acuity may be relatively limited if the fovea is spared. When the fovea is involved, GA quickly causes blindness.

Genetic susceptibility has become increasingly recognized as a risk factor and important contributor to AMD. More than 19 genetic polymorphisms have been demonstrated to influence AMD risk, with as many as 5 of these encoded by genes that modulate the complement system. Inflammatory processes, especially those mediated by complement are thought to play a key role in AMD.[5] It is thought that these may contribute to loss of choriocapillaris, photoreceptors, and RPE cells.

GA is responsible for approximately 20% of all cases of legal blindness in North America (ie, BCVA 20/200 or worse) with increasing incidence and prevalence owing to a higher life expectancy.[4] While there is treatment for exudative AMD with anti-VEGF therapies, no approved therapy exists for GA which is usually bilateral and relentlessly progressive. It represents a significant unmet need as it leads to significant visual impairment and affects more than 5 million people worldwide.[13]

An overview of available information regarding pegcetacoplan follows below. Further details can be found in the pegcetacoplan investigator's brochure (IB).[26]

4.1.2. Pegcetacoplan

Pegcetacoplan is a PEGylated cyclic peptide inhibitor of complement C3. Pegcetacoplan is formed by 2 identical pentadecapeptides (combining a bioactive cyclic tridecapeptide C3-

inhibiting moiety and a 2-amino acid linker) covalently coupled to each end of a linear 40 kDa polyethylene glycol (PEG) chain. There are 2 peptide moieties per molecule of pegcetacoplan.

The peptide portion of the drug binds to complement C3 and is a broad inhibitor of the complement cascade, a biological process that is part of innate immunity and is involved in multiple inflammatory processes. The PEGylation of the molecule imparts slower clearance from the vitreous humor following administration.

Pegcetacoplan Intravitreal Injection 15 mg/0.1 mL will be provided as a 150 mg/ mL sterile solution of pegcetacoplan in stoppered glass vials. Pegcetacoplan is a sterile, isotonic solution in acetate-buffer, pH 5.0, containing trehalose. The drug product is packaged in 2R clear Type I glass vials with 13 mm FluroTec-coated chlorobutyl grey stoppers and sealed with 13 mm aluminum/polypropylene flip-off type seals.

4.1.3. Nonclinical Data

This section is intended to briefly summarize information on the safety, tolerability, and PK of intravitreal (IVT), intravenous (IV) and subcutaneous (SC) injections of pegcetacoplan. For complete and detailed information, refer to the IB.

As pegcetacoplan is only pharmacologically active in primates, the pivotal IVT toxicological studies have been conducted in cynomolgus monkeys. The safety and tolerability of IVT-administered pegcetacoplan has been assessed in a Good Laboratory Practice (GLP)-compliant pivotal chronic (9-month) repeat-dose study in cynomolgus monkeys. In addition, 2 GLP-compliant 2-month ocular bridging studies (the first comparing drug substance from 2 different CMOs, the second assessing the safety and tolerability profiles of 3 different formulations to support the Phase 3 clinical study) have been conducted in cynomolgus monkeys as well. . Pegcetacoplan was observed to be minimally immunogenic in the 9-month study as evidenced by a lack of circulating antibodies in the majority of monkeys studied. The NOEL for chronic (9-month) IVT dosing was concluded to be >24.8 mg/eye. The results of this 9-month chronic study in cynomolgus monkeys support the safety of chronic IVT injections of pegcetacoplan at monthly or bimonthly intervals in humans.

PK assessments included in the chronic (9-month) study revealed serum concentrations of pegcetacoplan that were approximately dose-proportional 24 hours after the first IVT dose. The serum $t_{1/2}$ of IVT-administered pegcetacoplan, determined in a separate PK assessing a 10 mg/eye dose (in 50 µL), was 10.4 days although the pegcetacoplan levels measured in the serum following infrequent IVT administration are orders of magnitude lower than pharmacological serum levels achieved with SC or IV administration.

Additional nonclinical studies in monkeys and rabbits, including assessments of other routes of administration (SC and IV), have further defined pegcetacoplan's safety profile. . Pegcetacoplan has been shown to be generally tolerated through all 3 routes of administration assessed (IVT, SC, and IV), with no adverse effects observed in safety pharmacology studies (*in vivo* cardiopulmonary telemetry and *in vitro* hERG inhibition assay) and genotoxicity studies (Ames, *in vitro* aneugenicity and clastogenicity in TK6 cells, and *in vivo* clastogenicity in mouse micronuclei).

CCI

[redacted]

CCI

[redacted]

4.1.4. Clinical Data

The initial safety and tolerability of pegcetacoplan following IVT administration in humans was tested in an open-label, single dose escalation, Phase I clinical study in patients with wet age-related macular degeneration under protocol POT-CP043014 (NCT02461771). The study was conducted in multiple ophthalmology clinical sites in the US and Australia. A single dose of pegcetacoplan was administered on Day 1 to patients suffering from wet AMD currently receiving anti-VEGF standard of care. Three escalating doses of pegcetacoplan administered IVT were studied. Three subjects received 4 mg; 3 subjects received 10 mg, and 7 subjects received 20 mg. No serious adverse events (SAEs) or drug-related adverse events (AEs) of concern were observed. It was concluded that administration of a single dose of pegcetacoplan IVT up to 20 mg is safe and well tolerated.

A Phase 2 study (Protocol POT-CP121614; NCT02503332) to assess the safety, tolerability, and evidence of activity of multiple intravitreal injections of pegcetacoplan in subjects with geographic atrophy secondary to AMD has been completed. The study was conducted at multiple ophthalmology clinical sites in the US, Australia, and New Zealand. Subjects were randomized in a 2:1:2:1 manner to either receive pegcetacoplan IVT 15 mg monthly for 12 months; sham IVT monthly for 12 months; pegcetacoplan IVT 15 mg every other month for 12 months; or sham IVT every other month for 12 months.

This study demonstrated a statistically significant slowing of disease progression at Month 12 at the prespecified alpha of 0.1. Pegcetacoplan administered monthly showed a 29% (P=.008) reduction in the rate of GA lesion growth compared to sham and pegcetacoplan administered every other month showed a 20% (P=.067) reduction. Pegcetacoplan has been generally well tolerated. The most frequently reported AEs have been related to the injection procedure (intravitreal injection), which are commonly found in this type of study. An imbalance in new active choroidal neovascularization (CNV) in subjects treated with pegcetacoplan was observed and the risk of developing new exudation may be increased in subjects with a prior history of neovascular AMD in the fellow eye. One subject in the sham arm, 1/81 (1%) was observed to have developed new active study eye exudation compared with 25/165 (15%) subjects that received pegcetacoplan IVT injections, 26 subjects in total. Of the 25 pegcetacoplan treated subjects with new active CNV, 18 subjects received PM treatment and 7 subjects received PEOM treatment.

Seventeen of the 25 (68%) subjects that developed new active study eye CNV had a prior history of neovascular AMD in the nonstudy fellow eye. No significant imbalance in history of neovascular AMD in the fellow eye was observed among the 3 arms to explain the imbalance in new exudation observed in study eyes. Visual acuity data did not demonstrate clear differences between subjects developing new study eye exudation compared with those that did not.

A Phase 1b study (Protocol APL2-103; NCT 03777332) to assess the safety of pegcetacoplan in subjects with GA secondary to AMD and low vision is currently ongoing. This study is being conducted at multiple ophthalmology clinical sites across the US. Subjects receive monthly treatment with intravitreal pegcetacoplan for 24 months.

4.1.5. Rationale

4.1.5.1. Rationale for Pegcetacoplan for Treatment of Geographic Atrophy

The rationale for the use of a complement inhibitor in patients with AMD is based on evidence from both human and animal studies. Human biochemical, genetic, and clinical lines of evidence indicate that the complement system plays a role in the etiology of AMD. Complement components including C3, the membrane attack complex, and complement factor H, are present in drusen and basal laminar deposits in eyes from patients with AMD.[7,8,9,10] Genetic variants of complement factor H,[8,11,12,13,14] C3,[15,16] complement factor I[17] and other complement components[18,19,20] have been associated with altered risks for the development of both the neovascular and atrophic forms of AMD. Patients with AMD also have signs of systemic complement activation, exhibiting higher serum levels of Complement Factor B, C3a, C5a, SC5b-9 (soluble terminal complement complex), C3d, and Ba compared to age-matched controls.[21,22,23,24]

4.1.6. Dose Selection

A single dose of 15 mg/0.1 mL injection administered monthly or EOM for 24 months will be tested in this study (see Section 7.3.1). Pegcetacoplan was well tolerated in a panel of animal toxicology studies. A 9-month, repeat-dose GLP study in cynomolgus monkeys was conducted to evaluate the safety of IVT injections of pegcetacoplan at doses up to 24.8 mg/eye at a frequency of 1 injection every 4 weeks.

In this pivotal chronic toxicological study conducted in monkeys, the NOEL was established as >24.8 mg of pegcetacoplan delivered intravitreally every 4 weeks for 9 months using a 100 µL injection. The volume of the human vitreous is approximately 4 mL, which is approximately 2.7-fold larger than the mean vitreous volume of cynomolgus monkeys, 1.5 mL.[25] Based on the difference in vitreous volume between man and cynomolgus and the NOEL defined in nonhuman primates, the human equivalent dose was determined to be 67 mg/eye every 4 weeks. The dose (15 mg/injection) of pegcetacoplan that will be evaluated in this clinical study is expected to result in drug concentrations approximately 4.5-fold lower that the NOEL observed in cynomolgus monkeys.

To support appropriate assessments of dose response to pegcetacoplan, pegcetacoplan is administered in 2 different frequencies in the study: monthly and every other month.

Physician feedback injecting a 200 mg/mL solution in the Phase 1 (Study POT-CP043014; NCT02461771) trial confirmed that 150 mg/mL is the highest practical concentration that can be routinely administered, which set the dose of the Phase 2 trial to 15 mg (ie, 0.1 mL of a 150 mg/mL solution). The 15-mg dose given monthly was found to be the most efficacious in the Phase 2 trial. The 15 mg dose given every other month demonstrated slightly less efficacy but offers a reduced treatment burden for patients and physicians.

4.2. Risk/Benefit

The Phase 1 (Study POT-CP043014; NCT02461771) and Phase 2 (Study POT-CP121614; NCT02503332) studies provide supporting evidence of a positive benefit-risk profile for the use of pegcetacoplan in treating patients with AMD, specifically patients with GA. These

preliminary results of an up to 28% reduction of GA progression rate support the evaluation of pegcetacoplan in a Phase 3 study.

The reported safety data from these studies demonstrated an acceptable safety and tolerability profile with no clinically significant safety concerns observed. A total of 178 patients have received at least 1 dose (15 mg/injection) of pegcetacoplan as part of these studies. In the Phase 2 study in patients with GA an imbalance in new exudation in subjects treated with pegcetacoplan was observed. Section 4.1.4 provides a summary of the study design and key results from both studies.

The safety monitoring practices employed by this protocol (complete ophthalmologic exam, IOP monitoring, OCT, fluorescein angiography, vital signs, hematology, serum chemistry, urinalysis, physical exam, vital signs, and AE questioning) are adequate to protect the subjects' safety. There are also risks associated with the ophthalmic procedures required for participants in this study. However, these are all standard procedures that are widely performed in ophthalmology.

In the days following any IVT injection, patients are at risk of developing sterile or infectious intraocular inflammation (eg, endophthalmitis). In recent studies conducted with pegcetacoplan IVT from a single manufacturer, events of transient moderate and severe intraocular inflammation have been observed. Other risks of IVT injection include traumatic cataract, retinal detachment, increased IOP, and hemorrhage.

The amount of blood (See Section 9.16) planned for collection from each subject over the 24 months of the study does not pose an undue risk in this patient population.

There is a potential health benefit for trial participants from receipt of study drug. If efficacious, pegcetacoplan is expected to alter the course of GA and slow its rate of progression.

5. STUDY OBJECTIVES

5.1.1. Primary Objective

To evaluate the efficacy of pegcetacoplan compared to sham injection in patients with GA secondary to AMD assessed by change in the total area of GA lesions from baseline as measured by fundus autofluorescence (FAF).

5.1.2. Key Secondary Objectives

To evaluate the efficacy of pegcetacoplan compared to sham injection in patients with GA secondary to AMD with respect to:

- Monocular maximum reading speed (study eye), as assessed by Minnesota Low-Vision Reading Test (MNREAD) or Radner Reading Charts (in select countries)
- Functional Reading Independence (FRI) index score
- Normal luminance best corrected visual acuity score (NL-BCVA) in the study eye

5.1.3. Secondary Objectives

- To evaluate the efficacy of pegcetacoplan compared to sham injection in patients with GA secondary to AMD with respect to:
  - Low luminance best corrected visual acuity score (LL-BCVA) in the study eye
  - Low luminance deficit (LLD) in the study eye
  - Total area of GA lesion(s) in the study eye
  - Monocular critical print size (study eye), as assessed by MNREAD or Radner Reading Charts (in select countries)
  - National Eye Institute Visual Functioning Questionnaire 25-Item Version (NEI VFQ-25) distance activity subscale score (in select countries)
  - Macular functional response as assessed by mesopic microperimetry

5.1.4. Safety Objectives

- To evaluate the safety and tolerability of pegcetacoplan compared to sham injection in patients with GA secondary to AMD as indicated by:
  - Incidence and severity of ocular and systemic treatment-emergent adverse events
  - Incidence of anti-therapeutic antibodies directed against pegcetacoplan
  - Incidence of new active CNV in the study eye

5.1.5. Exploratory Objectives

- To evaluate the efficacy of pegcetacoplan compared to sham injection in patients with GA secondary to AMD as indicated by:
    - NEI VFQ-25 composite score
    - NEI VFQ-25 near activity subscale score (in select countries)
    - Comparison between study eye and fellow eye in change in GA lesion size
    - To evaluate the binocular maximum reading speed as assessed by MNREAD or Radner Reading Charts (in select countries)
    - To evaluate the binocular critical print size as assessed by MNREAD or Radner Reading Charts (in select countries)
- To evaluate the relationship between genetic polymorphisms associated with AMD with GA progression and response to pegcetacoplan
- To evaluate the incidence of new onset of subclinical CNV in the study eye
- To assess sensitivity and specificity of a digital reading speed application to detect disease progression / regression (optional, select sites)
- To assess sensitivity and specificity of a digital visual function application to detect disease progression / regression (optional, select sites)

6. PATIENT POPULATION

The study population includes approximately 600 subjects to be randomized at approximately 100 multinational sites. To participate in the study, subjects must be diagnosed with GA of the macula secondary to AMD in the study eye.

6.1.1. Inclusion Criteria

The study eye must meet all inclusion criteria. If both eyes meet the inclusion criteria, the eye with the worst normal luminance visual acuity at the screening visit will be designated as the study eye. If both eyes have the same visual acuity, the right eye will be selected as the study eye.

Ocular-specific inclusion criteria apply to the **study eye** only, unless otherwise specified.

1. Age ≥60 years.
2. Normal Luminance best corrected visual acuity of 24 letters or better using Early Treatment Diabetic Retinopathy Study (ETDRS) charts (approximately 20/320 Snellen equivalent).
3. Clinical diagnosis of GA of the macula secondary to AMD as determined by the investigator and confirmed by the reading center.
4. The GA lesion must meet the following criteria as determined by the central reading center's assessment of FAF imaging at screening:
    a. Total GA area must be ≥2.5 and ≤17.5 mm2 (1 and 7 disk areas [DA] respectively).
    b. If GA is multifocal, at least 1 focal lesion must be ≥1.25 mm2 (0.5 DA), with the overall aggregate area of GA as specified above in 4a.
    c. The entire GA lesion must be completely visualized on the macula centered image and must be able to be imaged in its entirety and not contiguous with any areas of peripapillary atrophy.
    d. Presence of any pattern of hyperautofluorescence in the junctional zone of GA. Absence of hyperautofluorescence (ie, pattern = none) is exclusionary.[1]
5. Adequate clarity of ocular media, adequate pupillary dilation, and fixation to permit the collection of good quality images as determined by the investigator.
6. Meets the following criteria related to microperimetry:
    a. Able to detect fixation target.
    b. Total elapsed time to complete the 10-2 68-point exam is ≤30 minutes in duration.
    c. Fixation losses must be ≤20%.
    d. Subject is willing and able to undertake microperimetry assessment in the opinion of the investigator.
7. Female subjects must be:
    a. Women of non–childbearing potential (WONCBP), or
    b. Women of childbearing potential (WOCBP) with a negative serum pregnancy test at screening and must agree to use protocol defined methods of contraception for the duration of the study and refrain from breastfeeding for the duration of the study.

8. Males with female partners of childbearing potential must agree to use protocol defined methods of contraception and agree to refrain from donating sperm for the duration of the study.
9. Willing and able to give informed consent and to comply with the study procedures and assessments.

6.1.2. Exclusion Criteria

Ocular specific exclusion criteria apply to the **study eye** only, unless otherwise specified.

1. GA secondary to a condition other than AMD such as Stargardt disease, cone rod dystrophy, or toxic maculopathies like plaquenil maculopathy in either eye.
2. Spherical equivalent of the refractive error demonstrating > 6 diopters of myopia or an axial length >26 mm.
3. Any history or active CNV, associated with AMD or any other cause, including any evidence of retinal pigment epithelium rips or evidence of neovascularization anywhere based on SD-OCT imaging and/or fluorescein angiography as assessed by the reading center.
4. Presence of an active ocular disease that in the opinion of the investigator compromises or confounds visual function, including but not limited to, uveitis, other macular diseases (eg, clinically significant epiretinal membrane [ERM], full thickness macular hole) or uncontrolled glaucoma/ocular hypertension). Benign conditions in the opinion of the investigator such as peripheral retina dystrophy are not exclusionary.
5. Intraocular surgery (including lens replacement surgery) within 3 months prior to randomization.
6. History of laser therapy in the macular region.
7. Aphakia or absence of the posterior capsule. Note: YAG laser posterior capsulotomy for posterior capsule opacification done at least 60 days prior to screening is not exclusionary.
8. Any ocular condition other than GA secondary to AMD that may require surgery or medical intervention during the study period or, in the opinion of the investigator, could compromise visual function during the study period.
9. Any contraindication to IVT injection including current ocular or periocular infection.
10. History of prior intravitreal injection.
11. Unable to perform microperimetry reliably in the opinion of the investigator.
12. Prior participation in another interventional clinical study for intravitreal therapies in either eye (including subjects receiving sham).
13. Prior participation in another interventional clinical study for geographic atrophy in either eye including investigational oral medication and placebo.
14. Participation in any systemic experimental treatment or any other systemic investigational new drug including within 6 weeks or 5 half-lives of the active ingredient

(whichever is longer) prior to the start of study treatment. Note: clinical trials solely involving observation, over-the-counter vitamins, supplements, or diets are not exclusionary.

15. Medical or psychiatric conditions that, in the opinion of the investigator, make consistent follow-up over the 24-month treatment period unlikely, or would make the subject an unsafe study candidate.
16. Any screening laboratory value (hematology, serum chemistry or urinalysis) that in the opinion of the investigator is clinically significant and not suitable for study participation.
17. Known hypersensitivity to fluorescein sodium for injection or hypersensitivity to pegcetacoplan or any of the excipients in pegcetacoplan solution.

6.2. Women of Childbearing Potential

WOCBP are defined as premenopausal women physiologically capable of becoming pregnant.

6.3. Women of Nonchildbearing Potential

WONCBP are defined as women meeting any of the following criteria:

- Older than 45 years with amenorrhea for > 2 years or older than 60 years with amenorrhea for > 1 year. Both confirmed by follicle-stimulating hormone (FSH) and LH levels.
- Has undergone hysterectomy,
- Has undergone bilateral oophorectomy,
- Has undergone bilateral salpingectomy.

6.4. Approved Methods of Contraception

Approved methods of contraception include:

- Combined (estrogen-and progestogen-containing) hormonal contraception associated with inhibition of ovulation:
    - Oral
    - Intravaginal
    - Transdermal
- Progestogen-only hormonal contraception associated with inhibition of ovulation:
    - Oral
    - Injectable
    - Implantable
- Intrauterine device
- Intrauterine hormone-releasing system

- Bilateral tubal occlusion
- Vasectomized partner *(provided that partner is the sole sexual partner of the WOCBP trial participant and that the vasectomized partner has received medical assessment of the surgical success)*
- Sexual abstinence *(defined as refraining from heterosexual intercourse during the entire period of risk associated with the study treatments). Sexual abstinence is only accepted when it is the preferred and usual lifestyle of the subject.*

Subjects must agree to use an approved method of contraception during the study and 90 days after their last dose of study drug.

6.5. Discontinuation of Subjects

A subject may withdraw from the study at any time for any reason without prejudice to his/her future medical care by the physician or at the institution. The investigator or sponsor may withdraw the subject at any time (eg, in the interest of subject safety). The investigator is encouraged to discuss withdrawal of a subject from treatment with the investigational product (IP) with the medical monitor, when possible.

Subjects who discontinue treatment with the IP can continue participation in the study and should be encouraged to return to the clinical site for as many follow-up visits as they can. In the event that a subject terminates early from the study, all early termination procedures should be performed even if they are outside the allowed study window.

The reason for termination, date of stopping treatment with IP, all follow-up information and the total amount of IP administered must be recorded in the case report form (CRF) and source documents.

7. TREATMENT OF SUBJECTS

7.1. Allocation to Treatment

Each subject will be assigned a unique screening number after signing the informed consent. Subjects who complete the study screening assessments and meet all the eligibility criteria will be scheduled to enter the study and randomized on Day 1. As part of the screening process, the reading center will evaluate FAF, OCT, Digital Color Fundus Photograph (DCFP), Near Infrared Reflectance (NIR), and fluorescein angiography (FA) to provide an objective assessment of subject eligibility. Subjects will be randomized 2:2:1:1 to receive treatment with PM, PEOM, sham injection monthly (SM) or sham injection every other month (SEOM), respectively. Table 1 presents the treatment arms along with the approximate number of subjects and injections per arm.

The randomization scheme will be generated and maintained by the sponsor, or designee. Subject randomization will be stratified by GA lesion area at screening (< 7.5 mm^2; ≥ 7.5 mm^2) and presence of CNV in the fellow eye (yes; no). Further details on the randomization procedures will be described in the statistical analysis plan.

7.2. Masking and Minimization of Bias

The intent of masking is to limit the occurrence of conscious and unconscious bias in the conduct and interpretation of the clinical study. Bias could arise from the influence that the knowledge of a specific treatment assignment may have on the recruitment and allocation of subjects, their subsequent care, the assessment of endpoints, the handling of withdrawals, and so on. The essential aim of masking, therefore, is to prevent identification of the treatments by the subject and the masked assessors associated with the conduct of the study until all such opportunities for bias have passed.

This is a double-masked study. Designated masked study site staff (eg, assistant(s), visual acuity technicians, OCT technicians, photographers, technicians administering questionnaires, subjects, reading center personnel, the assigned evaluating physician(s), and the sponsor) will be masked to treatment assignment. However, the treating physician and any associated support staff involved in performing the intravitreal or sham injections will be unmasked to study treatment. These individuals are not allowed to discuss treatment and/or patient outcome with masked study staff, including the evaluating physician. The principal investigator must be masked to subjects' treatment assignment. To prevent bias in treatment assignment, eligible subjects will be randomized using a web-based randomization system. Documentation will be put in place to avoid unintentional unmasking during the study. All study roles will be clearly documented on the site delegation of authority log and once the roles have been designated and executed, these roles should not be switched during the conduct of the study. In unforeseen circumstances, a site can contact the sponsor to switch a study staff member from the masked role to the unmasked role but not vice versa.

7.2.1. Unmasking

In the event of a medical emergency where the knowledge of subject treatment by masked individuals (eg, the subject or his/her physician) is required, an individual investigator (or designee) will have the ability to unmask the treatment assignment for a specific subject and share that information with the appropriate parties. All documentation indicating unmasking must be retained with the subject's source documentation in a secure manner. A DMC will be set-up to monitor patient safety and review data. The DMC will be provided unmasked safety data but will be masked to efficacy data unless this data is deemed medically necessary. Procedures for DMC unmasking will be documented in a DMC Charter.

For regulatory reporting and if required by local regulations, the sponsor will unmask study treatment for all serious, unexpected adverse reactions that are considered to be related to study drug. Subjects who have had their treatment assignment unmasked secondary to a serious or unexpected AE or medical emergency will no longer receive study treatment. However, they should continue to complete as many of the follow-up visits as possible.

The study unmasking for the primary analysis at 12 months will be limited to the analysis team and personnel only on an as-needed basis. All other personnel in the "masked" role will remain masked until the end of study. A document listing out the roles and responsibilities of the individuals participating in the unmasking analysis will be provided prior to the unmasking.

7.3. Dosage and Administration

7.3.1. Dose Levels and Treatment Arms

After randomization and during the treatment phase beginning at Day 1, all subjects will receive a single dose of 15 mg pegcetacoplan/0.1 mL or sham injection intravitreally either monthly or every other month depending on treatment designation as presented in Table 1 below.

Table 1: Treatment Arms with Approximate Number of Subjects

Treatment Arms
Pegcetacoplan 15 mg/0.1 mL monthly for 24 months (n= approximately 200 subjects; 24 pegcetacoplan injections)
Pegcetacoplan 15 mg/0.1 mL EOM for 24 months (n= approximately 200 subjects; 12 pegcetacoplan injections)
Sham monthly for 24 months (n= approximately 100 subjects; 24 sham injections)
Sham EOM for 24 months (n= approximately 100 subjects; 12 sham injections)

7.3.2. Treatment Administration

Only qualified study staff and those delegated the responsibility of study drug administration on the delegation of authority log should perform this procedure. All staff should be appropriately trained on all procedures prior to performing the procedures. Sites should follow the Visit Schedule for order of procedures and assessments.

Administration of study treatment (pegcetacoplan or sham) can be done on a separate day from the assessment visit if both days fall within the visit window. If this occurs on the randomization

visit, then the administration of pegcetacoplan or sham should be done within 3 days of randomization and after approval from the medical monitor. When study treatment administration is on a day other than a study visit, then the only assessment that must be done on the day of study treatment administration is the IOP preinjection.

If a subject falls outside the visit window for a dosing visit, the dose should be skipped and the subject should be scheduled on time for the next dosing visit.

7.3.3. Drug Supplies

7.3.3.1. Identity of Investigational Product

Pegcetacoplan will be supplied as a liquid solution in stoppered glass vials and should be stored according to the label. Specific instructions for preparing pegcetacoplan for the IVT injection procedure will be provided in the manual of procedures. Pegcetacoplan Intravitreal Injection 15 mg/0.1 mL (150 mg/mL) is a sterile, isotonic solution of pegcetacoplan in acetate-buffer, pH 5.0, containing trehalose. The drug product is packaged in 2R clear Type I glass vials with 13 mm FluroTec-coated chlorobutyl grey stoppers and sealed with 13-mm aluminum/polypropylene flip-off type seals.

Sham will be provided as empty stoppered glass vials and should be stored according to the label.

7.3.3.2. Storage

Vials should be automatically stored as per the instructions until ready for use. Each vial should only be used once. Vials should not be shaken and should be protected from sunlight.

7.3.3.3. Accountability

Pegcetacoplan drug product and sham vials will be provided to a designee at the study site and must be stored in a pharmacy or otherwise locked and secured, at the temperature specified on the label. The drug product supply is accessible only to those individuals authorized by the PI. The sponsor will supply sufficient quantities of pegcetacoplan drug product and sham to allow completion of this study. The site should only use the investigational medicinal product provided by the sponsor for use in the study.

Designated unmasked study staff will provide the study treatments to the subjects in accordance with their assigned subject numbers and the randomization schedule. During the study, the receipt of the drugs supplied at the clinical site and of study treatment dispensation for each subject will be documented in drug accountability records. These drug accountability records are to be kept separate from the patient medical records and other source documents.

All used vials should be retained by the clinical site until drug accountability monitoring is performed and then returned to the sponsor or designee or destroyed per sponsor instructions. At the conclusion of the study, any unused IP returned to the sponsor or designee, or destroyed per sponsor instructions, and this will be documented in the drug accountability records.

7.3.4. Intravitreal Pegcetacoplan Administration

Subjects receiving active treatment will be administered 0.1 mL IVT injection of pegcetacoplan according to their treatment designation using a thin wall needle. Detailed instructions on drug preparation, preinjection procedures, administration of pegcetacoplan, and postinjection procedures will be provided in the manual of procedures.

Clinic staff involved in the injection tray assembly, anesthetic preparation, and study drug preparation and administration will follow appropriate aseptic techniques to minimize the risk of potential AEs associated with IVT injections.

Administration of pegcetacoplan is only allowed if preinjection IOP ≤21 mm Hg. If necessary, antiglaucomatous medication can be given to lower the IOP. To minimize transient IOP elevation after IVT injection of pegcetacoplan, decompression of the eye **must** be performed before all pegcetacoplan injections. This is done by applying moderate pressure to the globe with cotton swabs for 30-60 seconds during anesthetic preparation.

In addition to the procedures outlined in the protocol and manual of procedures (MOP), adherence to specific institutional policies associated with IVT injections will be observed.

7.4. Sham Injection Administration

The procedure for sham injection will be the same as that used for IVT injection until the actual injection but no actual injection will occur. The injecting physician will only touch the study eye with the blunt end of the syringe. No needle or medication will be injected inside the eye. Detailed instructions on sham injection procedures and postinjection procedures will be provided in the manual of procedures.

Subjects randomized to the monthly or every-other-month sham-injection groups will receive sham injection monthly or every other month, respectively. The same assessments will be performed as for the subjects in the pegcetacoplan groups.

7.5. Concomitant Therapies

Any concomitant medications a participant is receiving at the start of the study, within 30 days prior to screening, or that are given for any reason during the study (except for routine medications given for ocular procedures required by the protocol, such as topical anesthetic) must be recorded in the source documents and CRF including start and stop date and time, dose, route, and indication. In addition, all invasive intraocular procedures from the previous 5 years must also be recorded in the source documents and CRF including start and stop dates. Surgical anesthetics, paramedical or alternative therapies (eg, acupuncture, herbal supplements) should also be recorded in the source documents and CRF within 30 days prior to screening.

Metoclopramide or other agents to prevent nausea induced by fluorescein injection may be administered at the discretion of the PI.

7.5.1. Treatment of New Exudation Related to Active Choroidal Neovascularization in the Study Eye and/or Fellow Eye

The suspected onset or presence of new exudation related to active CNV secondary to AMD in the study eye and/or fellow eye must be documented in the source documents and CRF. If the

investigator suspects new exudation related to active CNV in the study eye based on fundus examination and/or OCT findings (eg, subretinal fluid, intraretinal fluid, cystoid macular edema, serous pigment epithelial detachment), a fluorescein angiography and Optical Coherence Tomography Angiography (OCT-A; select sites only) must also be captured following the imaging protocol procedure. All images, as outlined above, must be sent to the reading center. The reading center will provide a report indicating whether or not evidence of active, exudative AMD is present or absent, based on the images sent for assessment.

The determination about initiation of anti-VEGF treatment for the exudation related to active CNV is the sole responsibility of the investigator. Treatment with anti-VEGF should start after the report is received by the site from the reading center, with the exception of cases with clear evidence of disease activity (eg,; subretinal hemorrhage, extensive subretinal fluid and/or edema, and/or presence of subretinal hyperreflective material) that, in the opinion of the investigator, may have a detrimental visual impact if not treated immediately.

If it is determined that the subject requires anti-VEGF therapy, ranibizumab or aflibercept should be selected and administered by the injecting (unmasked) physician. Ranibizumab should be given monthly and aflibercept every other month after 3 monthly loading doses. The frequency of aflibercept can be changed to monthly if deemed necessary by the investigator, however the physician should refrain from using as-needed treatment (PRN) or treat and extend protocols. Every effort should be made to use the same anti-VEGF therapy for a subject during the course of the study and all treatments should be documented in the CRF.

If anti-VEGF therapy is administered in the study eye on the same day as an pegcetacoplan (or sham) injection, the anti-VEGF therapy shall be administered first and the pegcetacoplan or sham injection shall occur at least 30 minutes after the anti-VEGF injection and only if the IOP is ≤21 mm Hg. Anti-glaucomatous medication can be given to lower the IOP to the appropriate range to allow for the pegcetacoplan injection.

In order to avoid potential unmasking, if anti-VEGF and pegcetacoplan are given at the same visit, the unmasked physician must perform both procedures. If the anti-VEGF therapy is given on a separate day from the pegcetacoplan administration, either the masked or the unmasked physician may perform this injection.

Treatment with anti-VEGF is allowed in the fellow eye. The treatments for the fellow eye can be administered according to the site’s standard protocol for CNV treatment. Any treatments or therapies administered to the fellow eye within 5 years of screening and while on study should be recorded as a concomitant medication.

7.5.2. Prohibited Therapies

The PI should make a determination regarding patient continuation of therapies used to treat concomitant medical conditions. Therapies as noted in the inclusion/exclusion criteria are prohibited as specified.

7.5.3. Endophthalmitis Treatment

Endophthalmitis cases must be reported as SAEs. The decision to treat a participant for endophthalmitis or suspected endophthalmitis will be guided by the clinical judgment of the investigator and in accordance with local guidelines (as applicable). A culture sample should be

performed prior to making a decision on treatment. The treatment method (pars plana vitrectomy vs intravitreal injection of antibiotics) and choice of antimicrobial agents are also at the discretion of the physician and should follow current standard practice patterns. The decision to use IVT steroids (eg, dexamethasone) for the treatment of endophthalmitis is also at the discretion of the physician.

8. STUDY PROCEDURES

A study schema is presented below in Figure 1. All randomized subjects will return every month to the clinical site for assessments and additional pegcetacoplan or sham injections according to their randomization scheme until Month 12. From Month 12 onwards, subjects will return to the clinical site based on their randomized treatment schedule (monthly or EOM) and will follow the treatment regimen and assessments outlined in the Visit Schedule until Month 24. In addition, subjects will be contacted via phone by masked study staff 4 (±2) days after the first 3 study treatments to collect safety information.

The end of this trial for each subject is defined as when the subject completes their Month 24 study visit, approximately 30 days (monthly treatment group) and approximately 60 days (EOM treatment group) after the last visit at which IP is administered. The period following the last dose of IP is sufficient to evaluate the safety of pegcetacoplan based on its half-life in the vitreous; further details can be found in the pegcetacoplan IB. At the end of the 24-month study period, subjects will be offered entry to enroll into a separate open-label study.

Figure 1: Study Schema

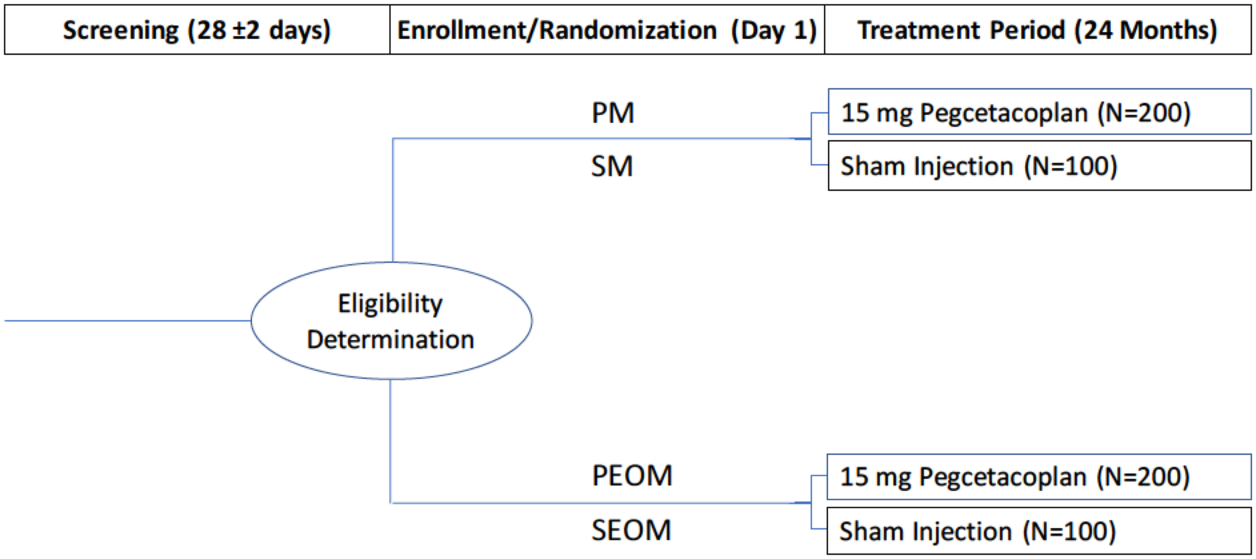

Abbreviations: PM= pegcetacoplan monthly; SM= sham monthly; PEOM= pegcetacoplan every-other-month; SEOM= sham every-other-month.

Subjects who discontinue study treatment should return to the clinic for study assessments as per the outlined visit schedule for their assigned treatment arm.

Safety will be assessed throughout the study by a number of evaluations including: monitoring of AEs, preinjection and postinjection monitoring, blood and urine samples will be collected, physical examination, vital signs, and follow-up phone calls will be performed. Blood samples will also be collected for anti-therapeutic antibodies, genotyping, and clinical repository (if the subject consents to this portion).

The planned length of participation in the study for each subject is approximately 25 months (from the beginning of the screening period through completion of the Month 24 visit). After completion of the 24-month treatment period, subjects will be offered entry into a separate open-label study.

8.1. Study Visit Schedule

Below is a condensed description of the study visits and the procedures and examinations that will be performed. Please refer to the Visit Schedule in Appendix A to Appendix D for a detailed schedule of procedures/assessments for the monthly and EOM visit schedules. Additional safety assessments not listed in this section or the flow chart may be performed if considered necessary at the discretion of the investigator.

8.1.1. Screening Period—Within 28 Days Prior to Randomization/ Treatment (Day –28 to –1)

8.1.1.1. Visit 1—All Subjects

Note: All ophthalmic procedures (including imaging) are to be performed on both eyes, except where specified.

Before any study-specific procedures are performed, the purpose and nature of the study should be explained and the patient should read, sign, and date the Institutional Review Board/Independent Ethics Committee (IRB/IEC)-approved Informed Consent Form (ICF). The individual obtaining consent from the patient and a witness, if applicable, should also sign and date the ICF. Once the patient has signed the ICF, a screening number should be assigned to the patient. Demographic information, significant medical/surgical history within the previous 5 years, invasive ocular procedures within the previous 5 years, and concomitant medications used within 30 days prior to screening should be collected (including vitamins and all over-the-counter as well as prescription medications). Complete smoking/ tobacco history should also be collected.

Subject eligibility should then be determined by reviewing the inclusion/exclusion criteria and the study eye should be selected. Prior to the administration of fluorescein, blood and urine should be collected for safety labs (including blood for human chorionic gonadotropin/follicle-stimulating hormone/luteinizing hormone [HCG/FSH/LH], if applicable) and vital signs along with a physical examination including weight and height should be performed.

A complete ophthalmic exam including slitlamp exam of the cornea, iris, anterior chamber, aqueous reaction (cells and flare), dilated fundus exam of the vitreous and retina, and intraocular pressure (IOP) measurement. Normal luminance BCVA should be performed prior to dilating the eyes. Images should be captured as outlined in the Visit Schedule (FAF, NIR, DCFP, FFA) and forwarded to the reading center for determination of eligibility if applicable.

Mesopic microperimetry should be performed on both eyes post dilation of the eyes and forwarded to the reading center.

8.1.2. Randomization/ Initial Treatment—Day 1—Within 28 Days of Screening

8.1.2.1. Visit 2—All Groups

At this visit, all inclusion/exclusion criteria should be reviewed prior to randomization and dosing, including the determination of eligibility by the reading center. Subjects will be randomized using the Interactive Web Response (IWR) System. A complete ophthalmic exam including slitlamp exam of the cornea, iris, anterior chamber, lens, and aqueous reaction (cells and flare), dilated fundus exam of the vitreous and retina, and IOP measurement will be performed and imaging collected as per the Visit Schedule.

All assessments should be performed on the same day. All study visits should be scheduled and projected based on the Day 1 visit date.

Blood should be drawn for anti-pegcetacoplan antibodies.

Prior to dilating the eyes, all functional tests should be performed (NL-BCVA, LL-BCVA, and MNREAD or Radner Reading Charts [in select countries]). Subjects at select sites will be trained on how to use the home-based digital applications for visual function and reading speed if the subject decides to participate in this portion. This training must occur after completion of all functional tests (NL-BCVA, LL-BCVA). Tests completed using the digital application at select sites (optional) should be completed prior to dilating the eyes but after completion of all functional tests and quality of life measures (NL-BCVA, LL-BCVA, MNREAD or Radner Reading Charts [in select countries], NEI VFQ-25, and FRI). The subject should be instructed to take the electronic device home and to complete the digital application weekly on the same day each week, if possible. The quality of life measures (NEI VFQ-25 and FRI) should be administered by the masked site staff.

Imaging should be performed including FAF, SD-OCT, OCT-A (select sites), endothelial cell count (select sites) and NIR and sent to the reading center for evaluation.

Study drug or sham injection should be performed by the unmasked physician as described in the manual of procedures and the study eye should be monitored post injection as outlined in Section 9.16. A follow-up phone call should be scheduled with the subject 4 ± 2 days after randomization Day 1 to assess for any AEs.

8.1.3. Treatment Phase—24 Months

8.1.3.1. Months 1-12

8.1.3.1.1. Visits 3-14 (Monthly and Every-Other Month Group)

During this phase, there will be clinic visits every month. Dosing and assessments will occur monthly in the monthly pegcetacoplan and sham injection treatment arms. Dosing will occur every other month in the EOM pegcetacoplan and sham injection treatment arms, however, the subjects will return monthly for assessments (with no dose given).

A complete ophthalmic exam including slitlamp exam of the cornea, iris, anterior chamber, lens, and aqueous reaction (cells and flare), dilated fundus exam of the vitreous and retina, and IOP measurement will be performed and imaging collected as per the Visit Schedule.

Blood will be drawn for safety labs, anti-pegcetacoplan antibodies, and genotyping (Month 2 only) prior to the administration of fluorescein as per the Visit Schedule. Samples will be collected for the clinical repository for those subjects that consent to this portion.

Prior to dilating the eyes, all functional tests will be performed (NL-BCVA, LL-BCVA, and MNREAD or Radner Reading Charts [in select countries]). Tests completed using the digital application at select sites (optional) will be completed prior to dilating the eyes but after completion of all functional tests and quality of life measures (NL-BCVA, LL-BCVA, MNREAD or Radner Reading Charts [in select countries], NEI VFQ-25, and FRI). At select sites the subject should be instructed to take the electronic device home and to complete the digital application weekly on the same day each week, if possible. The subject will be instructed to bring back the electronic device for the visits specified in the schedule of events. The quality of life measures (NEI VFQ-25 and FRI) will be administered by the masked site staff.

Mesopic microperimetry should be performed on both eyes (where specified) post dilation of the eyes and forwarded to the reading center.

Imaging will be performed including FAF, FFA, SD-OCT, OCT-A (select sites), endothelial cell count (select sites) and NIR and sent to the reading center for evaluation per the visit schedule.

Study drug or sham injection should be performed by the unmasked physician as described in the manual of procedures and the study eye should be monitored post injection as outlined in Section 9.16. A follow-up phone call should be scheduled with the subject as outlined in the schedule of events.

In the event that a subject is early terminated from the study, all early termination procedures should be performed even if they are outside the allowed study window.

If the subject would like to discontinue dosing but is amenable to continuing in the study, the site should make every effort to have the subject complete as many follow-up visits as possible.

8.1.3.2. Months 13-24

8.1.3.2.1. Visits 15-26 (Monthly Group) and 15-20 (Every-Other-Month Group)

During this phase, clinic visits will follow treatment designation (ie, the monthly subjects will return monthly for dosing and assessments and the EOM group will return EOM for dosing and assessments). At select sites, subjects will complete the home-based digital assessments weekly during this period.

A complete ophthalmic exam including slitlamp exam of the cornea, iris, anterior chamber, lens, and aqueous reaction (cells and flare), dilated fundus exam of the vitreous and retina, and IOP measurement will be performed and imaging collected as per the Visit Schedule.

Blood should be drawn for safety labs, genotyping, and anti-pegcetacoplan antibodies prior to the administration of fluorescein as per the Visit Schedule. Samples will be collected for the clinical repository for those subjects that consented to this portion.

Prior to dilating the eyes, all functional tests should be performed (NL-BCVA, LL-BCVA, and MNREAD or Radner Reading Charts [in select countries]). The quality of life measures (NEI VFQ-25 and FRI) should be administered by the masked site staff.

Mesopic microperimetry should be performed on both eyes (where specified) post dilation of the eyes and forwarded to the reading center.

Imaging should be performed including FAF, FFA, SD-OCT, OCT-A (select sites), endothelial cell count (select sites) and NIR and sent to the reading center for evaluation per the visit schedule.

IVT (or sham) injection should be performed by the unmasked physician as described in the manual of procedures and the study eye should be monitored post injection as outlined in Section 9.16.

In the event that a subject is early terminated from the study, all early termination procedures should be performed even if they are outside the allowed study window.

If the subject would like to discontinue dosing but is amenable to continuing in the study, the site should make every effort to have the subject complete as many follow-up visits as possible.

The completion of the 24-month study period occurs approximately 30 days (monthly treatment group) and approximately 60 days (EOM treatment group) after the last visit at which investigational product (IP) is administered. The period following last dose of IP is sufficient to evaluate the safety of pegcetacoplan based on its half-life in the vitreous; further details can be found in the pegcetacoplan IB. At the end of the 24-month study period, subjects will have the option to enroll in a separate open-label study.

8.1.4. Early Termination Visit

A list of all assessments to be performed at the early termination visit can be found on the Visit Schedule. All subjects who end the study early for any reason must complete the early termination visit, however all efforts should be made to have the subject return for as many follow-up visits as possible even if dosing does not occur. All ophthalmic procedures are to be performed on **BOTH EYES**.

8.1.5. Unscheduled Visits

If a subject returns to the clinical site before their next scheduled visit for an assessment of an AE or at the request of the physician, all safety assessments should be performed and any additional assessments as deemed medically necessary by the physician.

9. ASSESSMENTS

The following evaluations will be performed during the study as outlined in the Visit Schedule in Appendix A to Appendix D. Refer to the MOP for detailed descriptions of study-related procedures.

9.1. Informed Consent

Written informed consent for participation must be obtained before performing any study-specific assessments. Informed consent for all subjects should be maintained within the subject source documentation.

9.2. Demographic Information/ Medical/ Surgical History

Demographic information will be collected from all subjects including but not limited to date of birth, race/ ethnicity (where locally permitted). All significant medical conditions and surgeries within the past 5 years should be captured for the subject including chronic and ongoing conditions. Any history or current use of tobacco is to be collected.

9.3. Ocular History/ Ocular Procedures

Ocular history within the previous 5 years should be collected and recorded for all subjects. The history should include any significant previous ocular surgeries, procedures and/ or medications or treatments used for these conditions.

9.4. Vital Signs

Vital signs consist of body temperature, respiratory rate, blood pressure (systolic and diastolic), and heart rate measurements.

On injection visits, vital signs will be measured prior to dosing. Vital signs should be taken with the patient in a seated position after resting for 5 minutes. Vital signs will be measured before venipuncture.

9.5. Physical Examination

A physical exam will be performed and should include but should not be limited to an evaluation of the eyes, ears, nose, throat, lymph nodes, head, and neurological function. A patient's height and weight should also be measured at screening. If any abnormalities are noted at screening, the PI, or designee, should determine the clinical significance of the finding and whether this will pose any safety risk to the subject. Any changes from baseline should be noted and the clinical significance assessed. Any new, clinically significant, findings should be documented as AEs.

9.6. Laboratory Analysis of Blood and Urine

Collection of blood and urine will occur at the study site and the samples will be shipped to a central laboratory for analysis. All samples (including urine) should be collected prior to treatment and FFA/OCT-A assessments (if applicable). Procedures for the collection and processing of blood and urine are provided in the laboratory manual.

Table 2 presents the laboratory and urine analysis that will be performed.

Table 2: Laboratory Sampling and Analysis

Hematology	Chemistry	Urinalysis	Other
Hemoglobin	Blood urea nitrogen	pH	Genotyping
Hematocrit	Creatinine	Specific gravity	Anti-pegcetacoplan Ab
Red blood cell count	Bilirubin (total, direct and indirect)	Protein	Human chorionic gonadotropin[a]
White blood cell count with differential	Albumin	Glucose	Follicle-stimulating hormone[b]
Platelet count	Alkaline phosphatase	Ketones	Luteinizing hormone[b]
	Aspartate aminotransferase	Bilirubin	
	Alanine aminotransferase	Blood	
	Creatine kinase	Nitrite	
	Glucose	Urobilinogen	
	Electrolytes (sodium, potassium, chloride, bicarbonate)	Leukocyte esterase	

[a] Serum Pregnancy Test (ie, HCG) will be performed for females of childbearing potential at screening only.
[b] FSH and LH will be performed for postmenopausal females at screening only.

The principal investigator, or designee, must review the results of the screening visit clinical laboratory tests (including any retest results) and confirm that these results do not show evidence of any medical condition that would make study participation inappropriate. The principal investigator, or designee, should also assess any changes from baseline at the follow-up visits and the final visit. Clinically significant laboratory values are to be recorded as AEs.

9.7. Genotyping Samples

The genetic marker sample will be used to evaluate the relationship between genetic polymorphisms associated with AMD with disease progression and response to pegcetacoplan.

A whole-blood sample will be collected for genetic marker analysis. These samples will be collected only for those subjects who consent to this analysis.

These samples will be stored as per the clinical repository guidelines outlined in Section 9.17 if the subject consents to this portion of the study. These samples will be stored up to 15 years after the date of the final closure of the associated clinical database if the patient consented to the clinical repository.

9.8. Urine Pregnancy Test

Urine pregnancy test will be performed in WOCBP only as outlined in the Visit Schedule in Appendix A to Appendix D.

9.9. Patient-Reported Outcomes

Data will be collected via interview-administered questionnaires to assess patient-reported outcomes during scheduled visits as outlined in the Visit Schedule. The questionnaire should be administered by the masked site staff and should be performed prior to any other assessments being performed that day.

Questionnaire data will be used to assess subject-reported efficacy of pegcetacoplan and changes in quality of life over time. Questionnaires will be translated into the appropriate language for each country or region.

9.9.1. The National Eye Institute Visual Functioning Questionnaire 25-Item Version (NEI-VFQ)

The NEI-VFQ is an interviewer-administered questionnaire designed to assess patient-reported visual function (Appendix E). The NEI-VFQ is to be administered by the masked staff. It is a 25-item questionnaire with a composite score and covers 12 domains of functional health status and well-being (general health, general vision, ocular pain, near activities (select countries), distance activities (select countries), social functioning, mental health, role difficulties, dependency, driving, color vision, and peripheral vision). Scoring yields 12 subscales based on the 12 domains covered in the questionnaire. These scales are scored from 0 to 100 with higher scores indicating better visual function. The recall period is not specified; however, it is important that the patient uses the same recall period each time the questionnaire is administered throughout their participation in the study.

9.9.2. The Functional Reading Independence (FRI) Index

The FRI will be interviewer-administered and is an individualized assessment of functional reading independence (Appendix F). The questionnaire has 7 items with 1 total index score. Higher levels on the scale represent higher functional reading independence. The recall period is 7 days.

9.10. Best Corrected Visual Acuity and Low Luminance Best Corrected Visual Acuity

Best corrected visual acuity (including best corrected visual acuity under low luminance [LL-BCVA]) will be measured at each visit as per the visit schedule by certified study staff. The study staff performing visual acuity should be masked to the treatment assignment. Best corrected visual acuity testing will be assessed on ETDRS chart starting at a distance of 4 m, performed by a certified VA examiner, and should precede any examination requiring administration of eye drops to dilate the eye or any examination requiring contact with the eye.

The LL-BCVA acuity will be measured by placing a neutral density trial lens causing a reduction of 2.0 log units in luminance. The same requirements apply to measurement of low luminance visual acuity as described above for best corrected visual acuity. Low luminance deficit will be auto-calculated.

A Visual Acuity Specifications procedure manual and training materials will be provided to all sites. All examiners will require certification prior to performing this assessment as part of the study.

9.11. Minnesota Low-Vision Reading (MNREAD) Test or Radner Reading Charts (in select countries)

MNREAD Test or Radner Reading Charts (Appendix G) should be administered first monocularly for both eyes and then binocularly. The Manual of Operations should be referenced for a list of versions that should be administered based on country. These tests should be administered prior to dilating the eyes.

9.12. Home-Based Functional Digital Applications (optional, select sites)

In addition to in-clinic assessments, visual function and reading speed will also be evaluated using applications on an electronic device. Subjects who decide to participate in this portion will be trained on how to use the digital applications during the Day 1 visit and will complete the assessments using the digital application in the clinic at Day 1, Months 1-3, Month 6, 12, 18, and 24. In-clinic assessments on the digital application should be completed after completion of all functional tests and quality of life measures prior to dilating the eyes (NL-BCVA, LL-BCVA, MNREAD or Radner Reading Charts [in select countries], NEI VFQ 25, and FRI).

Subjects will also take home the electronic device to complete visual function and reading speed assessments weekly beginning at Day 1. Subjects should be instructed to complete the assessments on the same day each week and at approximately the same time of day each week, if possible.

9.13. Complete Ophthalmic Exam

The complete ophthalmic exam will consist of the following:

- External examination of the eye and adnexa.
- Routine screening for eyelids/pupil responsiveness (including ptosis, abnormal pupil shape, unequal pupils, abnormal reaction to light, and afferent pupillary defect).
- Slitlamp examination (cornea, anterior chamber, iris, lens, aqueous reaction [cells and flare]). Please see Appendix H for grading scales.
- Dilated fundus exam including evaluation of retina and vitreous (ie, posterior segment abnormalities, retinal hemorrhage/detachment, and vitreal hemorrhage density and vitreous cells). Vitreal hemorrhage density and vitreous cells grading scales are outlined in Appendix I.
- IOP measurement—A measurement of intraocular pressure will be conducted using either Tono-Pen or Goldmann applanation tonometer as outlined in the MOP. This should be performed prior to dilating the eyes and the same method should be used for all measurements in the same subject throughout the study.

9.14. Ocular Imaging and Microperimetry

The following ocular images will be obtained and sent to the reading center as outlined in the visit schedule. A reading center manual along with training materials will be provided to all sites which will provide information on standardized procedures for the collection, storage, and transmission of all images. Prior to any images being taken at the site, site personnel must be

properly trained and certified and test images and systems and software must be certified and validated by the reading center. Only trained and certified site staff delegated the responsibility of image collection should perform this task. Ocular images obtained as part of this study are:

- Digital Color Fundus Photographs
- Fluorescein Angiography
- Spectral Domain Optical Coherence Tomography imaging
- Fundus Autofluorescence (Heidelberg Spectralis Instrument)
- Near Infrared Reflectance
- Endothelial Cell Count (Specular Microscopy): Select sites only
- Mesopic Microperimetry of the study eye only (at screening, performed on both eyes)
    - To account for the learning curve of this test, the patient is allowed up to 3 attempts to meet the criteria for this portion
- Optical Coherence Tomography Angiography (OCT-A): Select sites only

If a patient misses a visit during which ocular images should have been taken, the images should be collected at the next scheduled study visit.

In the event that a subject is suspected to have new active CNV in the study eye and/or the fellow eye, an SD-OCT and FFA using the protocol specified procedures should be performed and sent to the reading center to confirm the diagnosis (Section 7.5.1). In addition, in select sites, OCT-A should also be captured according to the study imaging protocol and sent to the reading center.

9.15. Postinjection Assessment

The study eye will be assessed after the intravitreal injection of pegcetacoplan or sham to ensure that the injection procedure and/or the study medication have not endangered the health of the eye. The initial postinjection assessments should be done within 5 minutes post injection and include a gross assessment of vision (light perception, hand motion). If the subject passes the gross vision test, he/she can be released from the clinic. If the subject does not pass the gross vision test, IOP must be measured at that time. Additional IOP measurement must be taken approximately every 30 minutes thereafter until IOP ≤30 mm Hg and the subject is able to be released from the clinic.

All subjects receiving an anti-VEGF (ranibizumab or aflibercept) and pegcetacoplan/sham injection on the same day, should have the IOP measured prior to and after the anti-VEGF (prepegcetacoplan). The second injection (pegcetacoplan/sham) can only be given if the IOP ≤21 mm Hg. Antiglaucomatous medication can be given to lower the IOP. The subject can only be released from the clinic if the IOP is ≤30 mm Hg.

Any subject who develops a significant and sustained raise in IOP (>30 mm Hg) after any injection, should be monitored according to the investigator's clinical judgment and may undergo additional procedures and measurements of IOP beyond those specified in the protocol

as well as IOP lowering procedures. If any concern or immediate toxicity is noted, the subject will remain at the site and will be treated according to the physician's clinical judgment.

9.16. Blood Volume for Study Assessments

Table 3: Total Study Blood Volume

Assay	Number of time points	Approximate volume per time point * (mL)	Approximate sample volume over course of study (mL)—all groups
Anti-pegcetacoplan antibodies	9	2	18
*Hematology	8	4	32
**Chemistry (incl. HCG/LH)	8	5	40
FSH/LH (postmenopausal women only)	-	5	-
Genotyping sample[a]	1	8	8
Clinical repository (select sites and only subjects that consent to clinical repository)	5	14	70
**Total blood volume**			168 [a]

Abbreviations: HCG=human chorionic gonadotropin; FSH=follicle-stimulating hormone ; LH=luteinizing hormone
[a] Represents the standard collection volume planned over the duration of the study, actual volume may vary by group and across sites.
* Volume will vary slightly between regions and analyzing labs based on the standard methodology.
** In some regions, may be included in the chemistry sample based on local methodology.

9.17. Samples for Clinical Repository (Optional, Select Sites)

Apellis intends to apply genomic research across the pegcetacoplan development program to explore how genomic variations may affect the clinical parameters associated with and response to pegcetacoplan. Select sites will be asked to collect additional whole-blood samples and derivatives thereof in a centrally administered facility for the long-term storage of human biologic specimens. The collection and analysis of these specimens will facilitate the rational design of new pharmaceutical agents and the development of diagnostic tests, which may allow for individualized drug therapy for subjects in the future. Specimens for the Genetic Biorepository will be collected from subjects who give specific consent to participate in this optional research only and this will only be done at a select group of sites.

Specimens will be used to achieve the following objectives:

- To study the association of biomarkers with efficacy, AEs, or disease progression
- To increase knowledge and understanding of disease biology

- To study drug response, including drug effects and the processes of drug absorption and disposition
- To develop biomarker or diagnostic assays and establish the performance characteristics of these assays

Future research may suggest other genes, gene categories, proteins, etc. as candidates for influencing not only response to pegcetacoplan but also susceptibility to AMD for which pegcetacoplan may be evaluated. Thus, this additional genomic research may involve the future study of additional unnamed genes or gene categories, but only as they relate to AMD disease susceptibility and drug action.

9.17.1. Approval by the Institutional Review Board or Ethics Committee

Collection and submission of biological samples to the clinical repository is contingent upon the review and approval of the exploratory research and the clinical repository portion of the N by each site's IRB or IEC and, if applicable, an appropriate regulatory body. If a site has not been granted approval for clinical repository sampling, this section of the protocol (Section 9.17) will not be applicable at that site.

9.17.2. Sample Collection

The following samples will be collected for research purposes, including but not limited to research on dynamic (noninherited) biomarkers related to AMD and related diseases, pegcetacoplan, and signaling pathways related to AMD and the complement pathway:

- Residual whole-blood clinical genotyping sample
- 14-mL whole-blood sample collected at the specified time points

For all samples, dates of consent and specimen collection should be recorded on the associated clinical repository page of the eCRF. For sampling procedures, storage conditions, and shipment instructions, see the laboratory manual.

All collected specimens will be destroyed no later than 15 years after the date of final closure of the clinical database. The clinical repository storage period will be in accordance with the IRB/EC-approved ICF and applicable laws (eg, health authority requirements).

9.17.3. Confidentiality and Data Ownership

Patient medical information associated with clinical repository specimens is confidential and may only be disclosed to third parties as permitted by the ICF (or separate authorization for use and disclosure of personal health information) signed by the patient, unless required by law.

Data derived from clinical repository specimen analysis on individual subjects will generally not be provided to the subjects or to study investigators unless required by law. The aggregate results of any research conducted using clinical repository specimens will be available in accordance with the effective Apellis policy on study data publication.

Any inventions and resulting patents, improvements, and/or know-how originating from the use of the clinical repository data will become and remain the exclusive and unburdened property of Apellis, including the right to sell, license, or assign the invention to another entity.

9.17.4. Consent to Participate in the Clinical Repository

The ICF will contain a separate section or separate ICF that will address participation in the clinical repository. The investigator or authorized designee will explain to each patient the objectives, methods, and potential hazards of participation in the clinical repository. Subjects will be told that they are free to refuse to participate and may withdraw their specimens at any time and for any reason during the storage period. Subjects who decline to participate in the clinical repository can still participate in this clinical study. The investigator should document whether the patient has given consent to participate by completing the clinical repository Research Sample Informed Consent eCRF. In the event of a clinical repository participant's death or loss of competence, the participant's specimens and data will continue to be used as part of the clinical repository research.

9.17.5. Withdrawal From the Clinical Repository

Subjects who give consent to provide clinical repository specimens have the right to withdraw their specimens from the clinical repository at any time for any reason. If a patient wishes to withdraw consent to the testing of his or her specimens, the investigator must inform the sponsor in writing of the patient's wishes using the clinical repository Patient Withdrawal Form and, if the study is ongoing, must enter the date of withdrawal on the Clinical Repository Research Sample Withdrawal of Informed Consent eCRF. The patient will be provided with instructions on how to withdraw consent after the study is closed. A patient's withdrawal from this study does not, by itself, constitute withdrawal of specimens from the clinical repository. Likewise, a patient's withdrawal from the clinical repository does not constitute withdrawal from this portion of the study.

9.17.6. Monitoring and Oversight

Clinical Repository specimens will be tracked in a manner consistent with GCP by a quality-controlled, auditable, and appropriately validated laboratory information management system to ensure compliance with data confidentiality as well as adherence to authorized use of specimens as specified in this protocol and in the ICF. Apellis monitors and auditors will have direct access to appropriate parts of records relating to patient participation in the clinical repository for the purposes of verifying the data provided to Apellis. The site will permit monitoring, audits, IRB/EC review, and health authority inspections by providing direct access to source data and documents related to the clinical repository samples.

10. SAFETY EVALUATIONS

Any clinically significant abnormalities persisting at the end of the study/ early withdrawal or end of the posttreatment phase will be followed by the investigator until resolution or until a clinically stable endpoint is reached. The study includes a number of evaluations to monitor safety including monitoring of AEs, postinjection monitoring, laboratory and urine sampling, physical examination, and vital signs.

10.1. Data Monitoring Committee

An external, independent data monitoring committee (DMC) will be formed with the purpose of reviewing all data across the conduct of the study on an ongoing basis. The DMC will follow a charter that will outline the frequency of meetings and the roles and responsibilities of all members. The DMC will meet at the beginning of the study and approximately every 6 months thereafter and will perform a masked review of all relevant events on an ongoing basis. An ad hoc meeting of the DMC may be convened by the sponsor or the DMC chairperson at any time between the regularly scheduled DMC meetings and data reviews, if warranted by new safety information or for any other reason. The DMC will communicate their recommendations to the sponsor who will notify the appropriate health authorities according to local regulatory requirements.

11. ADVERSE EVENTS

11.1. Definition

An AE is any untoward medical occurrence associated with the use of a drug in humans, whether or not it is considered drug related. An AE can, therefore be any unfavorable and unintended sign, including a clinically significant abnormal laboratory finding, symptom, or disease temporally associated with the use of an IP, whether or not considered related to the IP.

Adverse events can be spontaneously reported by the subject and/or in response to an open question from the study personnel or revealed by observation and will be recorded during the study at the investigational site. All identified AEs must be recorded and described on the appropriate AE or SAE page of the eCRF.

Fluctuating or nonsignificant changes in laboratory values do not necessarily qualify for AE recording but are still collected and recorded via the appropriate eCRF form, if applicable. If these changes in laboratory values are linked to a diagnosis, this diagnosis should be reported as an AE, especially if the diagnosis constitutes an SAE or leads to discontinuation of administration of IP.

11.2. Recording Adverse Events

Adverse events and SAEs will be collected from the signing of the consent form until the last visit or early termination visit, 30 days after the last IP administration for the monthly treatment groups and 60 days after the last IP administration for the EOM treatment groups.

Any events that occur prior to dosing will be categorized as pretreatment events; events occurring after dosing will be recorded as treatment-emergent adverse events (TEAEs) (start date of dosing and, therefore, categorization of the event will be dependent on randomization assignment).

For each AE, the investigator will evaluate and report the onset date (and time if applicable), resolution date (and time if applicable), intensity, causality, action taken, serious outcome, and whether or not it caused the subject to discontinue the study.

If possible, the outcome of any AE that caused permanent discontinuation or was present at the end of the study should be reported, particularly if the AE was considered by the investigator to be related to the IP. Subjects experiencing AEs that cause interruption or discontinuation of IP, or those experiencing AEs that are present at the last visit or early termination visit should receive follow-up as appropriate.

All SAEs must be reported to the sponsor/Apellis Safety via eCRF within 24 hours of becoming aware of the event, whether or not the event is deemed treatment-related. If the electronic data capture (EDC) system is not operational (or for paper-based study[ies]), the site must complete the paper SAE form and email to CCI [redacted] immediately and also within 24 hours of becoming aware of the event. The reported information submitted as a paper SAE must be entered into the EDC system once it becomes operational.

Adverse events will be coded in accordance with the Medical Dictionary for Regulatory Activities (MedDRA). If known, the diagnosis of the underlying illness or disorder should be recorded, rather than its individual symptoms.

11.3. Reporting Adverse Events

The sponsor has the responsibility to inform concerned health authorities, ethic committees, and investigators about suspected unexpected serious adverse reactions in line with GCP guidance and applicable regulatory requirements.

If required, specific SAEs should be reported to the concerned ethic committees in compliance with local requirements

11.3.1. Relationship of Events to Study Treatment

All AEs that occur during this study will be recorded. The investigator will review each event and assess its relationship to study treatment (definitely related, possibly related, unlikely related, not related, unknown). The date and time of onset, time relationship to drug dosing, duration, and outcome (recovered/resolved, recovered/resolved with sequelae, recovering/resolving, not recovered/not resolved, fatal, or unknown) of each event will be noted.

The following definitions should be considered when evaluating the relationship of AEs and SAEs to the study treatment:

Definitely Related	Event or laboratory test abnormality, with plausible time relationship to drug intake<br>Cannot be explained by disease or other drugs<br>Response to withdrawal plausible (pharmacologically, pathologically)<br>Event definitive pharmacologically or phenomenologically (ie, an objective and specific medical disorder or a recognized pharmacological phenomenon)<br>Re-challenge satisfactory, if necessary
Possibly Related	Event or laboratory test abnormality, with reasonable time relationship to drug intake<br>Could also be explained by disease or other drugs<br>Information on drug withdrawal may be lacking or unclear
Unlikely Related	Event or laboratory test abnormality, with a time to drug intake that makes a relationship improbable (but not impossible)<br>Disease or other drugs provide plausible explanations
Not Related	Event or laboratory test abnormality, is plausibly related to the participant's clinical state, underlying disease, or the study procedure/conditions<br>Time relationship to drug intake makes a relationship unreasonable<br>Other obvious causes for event or laboratory test abnormality exist
Unknown	Report suggests an adverse event, however, cannot be judged at this time because information is insufficient or contradictory<br>More data for proper assessment is needed, or additional data is under examination

11.3.2. Severity of Events

The following definitions should be considered when evaluating the severity of AEs and SAEs:

Mild	Asymptomatic or mild symptoms; clinical or diagnostic observations only; intervention not indicated.
Moderate	Minimal, local or noninvasive intervention indicated; limiting age-appropriate instrumental ADL [a].
Severe	Medically significant but not immediately life-threatening; hospitalization or prolongation of hospitalization indicated; disabling; limiting self-care ADL[b].<br>Note**:** An experience may be severe but may not be serious, eg, severe headache).

Abbreviation: ADL=activities of daily living
A semicolon indicates 'or' within the description of the grade.
[a] Instrumental ADL refer to preparing meals, shopping for groceries or clothes, using the telephone, managing money, etc.
[b] Self-care ADL refer to bathing, dressing and undressing, feeding self, using the toilet, taking medications, and not bedridden.

11.4. Serious Adverse Events

An SAE is any AE or suspected adverse reaction that, in the view of either the investigator or sponsor, results in any of the following outcomes: death; life-threatening; inpatient hospitalization or prolongation of existing hospitalization; a persistent or significant incapacity or substantial disruption of the ability to conduct normal life functions; or a congenital anomaly/birth defect.

Important medical events that may not result in death, be life-threatening*, or require hospitalization may be considered serious when, based upon appropriate medical judgment, they may jeopardize the subject and may require medical or surgical intervention to prevent one of the outcomes listed in the above definition.

Examples of such medical events include allergic bronchospasm requiring intensive treatment in an emergency room or at home; blood dyscrasias or convulsions that do not result in inpatient hospitalization; or the development of drug dependency or drug abuse.

**Life-threatening* is defined as an AE or suspected adverse reaction, which in the view of either the investigator or sponsor places the subject at immediate risk of death as it occurred. It does not include an AE or suspected adverse reaction that, had it occurred in a more severe form, might have caused death.

Unexpected Adverse Event

An AE is considered "unexpected" if it is not listed in the Reference Safety Information section of the IB.

11.5. Treatment and Follow-up of Adverse Events

AEs (whether serious or nonserious), including clinically significant abnormal laboratory test values, will be evaluated by the investigator and treated and/or followed up until the symptoms or value(s) return to baseline or are clinically stable. Treatment of AEs will be performed by appropriately trained medical personnel, either at the clinical site or at a nearby hospital emergency room. When appropriate, medical tests and/or examinations will be performed to document resolution of the event(s).

AEs continuing after completion of the study will be followed up by telephone or with visits per the discretion of the investigator. If possible, the outcome of any AE that caused discontinuation from the study or was present at the end of the study should be reported, particularly if the AE was considered by the investigator to be related to the study drug.

11.6. Pregnancy

Although pregnancy is not an AE, all pregnancies and suspected pregnancies (including a positive pregnancy test regardless of age or disease state) occurring with a female subject or the female partner of a male subject, must be followed to conclusion to determine their outcome and are considered immediately reportable events.

The pregnancy, suspected pregnancy, or positive pregnancy test must be reported to Apellis Safety within 24 hours of the investigator's awareness using the paper Pregnancy Report Form. The Pregnancy Report Form shall be signed and dated by the investigator and submitted via email to [redacted].

The investigator must follow the subject until completion of the pregnancy and must report the outcome of the pregnancy (eg, delivery, termination, etc.) and neonatal status up to 12 months postdelivery. An abnormal outcome is defined as any pregnancy that results in the birth of a child with persistent or significant incapacity or substantial disruption of the ability to conduct normal life functions (formerly referred to as disabilities), congenital anomalies, or birth defects. In the event of an abnormal outcome, an SAE Report Form will be required.

11.7. Disease Progression

Normal progression or worsening of the medical condition under study (eg, vision loss due to the progression of GA in either eye), by itself, does not necessarily constitute an AE unless the change can be reasonably attributed to an action of the test article and not only to its lack of efficacy. Disease progression that requires an intervention (eg, administration of IVT anti-VEGF agents for neovascular AMD) should be recorded as an AE in the eCRF.

11.8. Withdrawal

Participants may choose to discontinue from treatment or to completely withdraw from this study for any reason at any time without penalty or prohibition from enrolling in other clinical protocols.

Participants wishing to withdraw from the study completely will be offered an early termination visit. This early termination visit will include the examinations outlined in Section 8.1.5.

Participants wishing to discontinue treatment but willing to continue with other study procedures, will return to the clinical site for follow-up visits, as per protocol, until Month 24.

Additional information on subject discontinuation is provided in Section 6.5.

12. DATA MANAGEMENT AND STATISTICAL CONSIDERATIONS

12.1. Data Collection

The investigators' authorized site personnel must enter the information required by the protocol on the CRF. A study monitor will visit each site in accordance with the monitoring plan and review the CRF data against the source data for completeness and accuracy. Discrepancies between source data and data entered on the CRF will be addressed by qualified site personnel. When a data discrepancy warrants correction, the correction will be made by authorized site personnel. Data collection procedures will be discussed with the site at the site initiation visit and/or at the investigator's meeting.

12.2. Clinical Data Management

Data are to be entered into a clinical database as specified in the contract research organization's data management plan. Quality control and data validation procedures are applied to ensure the validity and accuracy of the clinical database.

Data are to be reviewed and checked for omissions, errors, and values requiring further clarification using computerized and manual procedures. Data queries requiring clarification are to be communicated to the site for resolution. Only authorized personnel will make corrections to the clinical database, and all corrections are documented in an auditable manner.

12.3. Statistical Analysis Process

The study will be analyzed by the sponsor or its agent.

The statistical analysis plan (SAP) will provide the statistical methods and definitions for the analysis of the efficacy and safety data, as well as describe the approaches to be taken for summarizing other study information such as subject disposition, demographics and baseline characteristics, IP exposure, and prior and concomitant medications. The SAP will also include a description of how missing, unused, and spurious data will be addressed.

To preserve the integrity of the statistical analysis and study conclusions, the SAP will be finalized prior to database lock. All statistical analyses will be performed using SAS (SAS Institute, Cary, NC 27513).

12.4. Planned Interim Analysis and Data Safety Monitoring Committee

No interim analysis is planned for this study. During the study, patient safety will be monitored on a continuous basis by the medical monitor until the last patient completes his or her last scheduled study assessment.

An independent DMC will also be established to provide an ongoing, independent review and assessment of the safety data, and to safeguard the interests and

safety of the participating patients in the study. The ongoing review of SAEs and other responsibilities of the DMC will be described in the DMC Charter.

The overall Type I (alpha) error rate for the study will be 0.05. To accommodate DMC data reviews, the alpha available for efficacy hypothesis testing will be reduced by 0.0001 for each DMC review. The study is expected to have 3-4 DMC data reviews prior to the primary analysis of 12-month data and 1 review afterward.

12.5. Sample Size Calculation and Power Considerations

Subjects will be randomized in a 2:2:1:1 ratio to receive treatment with pegcetacoplan monthly, pegcetacoplan every other month, sham monthly, or sham every other month. The annual growth rate in GA lesion area is expected to have a mean of 1.47, 1.70 and 2.13 mm^2/year for pegcetacoplan monthly, pegcetacoplan every-other-month, and sham-pooled groups, respectively, as estimated from the results of a Phase 2 trial for pegcetacoplan. The standard deviation of the lesion growth is estimated to be 1.50 mm^2 based on the same Phase 2 trial data or 1.25 mm^2 based on natural history data.[27] The following table provides an approximation of the study power for a sample size of 200 subjects in each group (ie, a total enrollment of 600 subjects) under different alpha values and standard deviations. The approximation is calculated using PROC POWER one-way analysis of variance, SAS 9.4. The study power is likely larger when utilizing the longitudinal data to model the primary endpoint. The actual study power may also vary based on the distribution of the stratification factors (ie, lesion area at screening, presence of CNV in fellow eye), and site enrollment.

Table 4: Power to Detect a Difference Among 3 Groups With an Equal Size of 200 Subjects

Common standard deviation (mm^2)	Alpha (2-sided)	Power for a true mean of 1.47, 1.70, and 2.13 mm^2/year for PM, PEOM, and sham, respectively		
		PM vs sham	PEOM vs sham	Overall (among 3 groups)
1.25	0.0495	> 99.9%	92.9%	99.9%
1.25	0.0248	99.9%	88.2%	99.7%
1.40	0.0495	99.7%	86.5%	99.3%
1.40	0.0248	99.3%	79.4%	98.6%
1.50	0.0495	99.2%	81.5%	98.4%
1.50	0.0248	98.4%	73.1%	97.0%

Abbreviations: PM = pegcetacoplan monthly; PEOM = pegcetacoplan every other month.

12.6. Statistical Analysis Sets

The **safety set** will consist of all subjects who receive any amount of IP.

The **intent-to-treat (ITT) set** will include all randomized subjects. Subjects will be analyzed in the treatment arm assigned at randomization with the 2 sham treatment arms being combined into a single ‘control’ group.

The **modified ITT (mITT) set** will include all randomized subjects who receive at least 1 injection of pegcetacoplan or sham and have baseline and at least 1 postbaseline value of GA lesion in the study eye as assessed by FAF.

The **per-protocol (PP) set** will include all ITT subjects who follow the protocol without any major deviation(s) that could impact the integrity of the data. A detailed description of the reasons for exclusion from the PP population will be included in the statistical analysis plan (SAP).

12.7. Efficacy Analyses

The primary, secondary, and exploratory efficacy analyses will be performed using the ITT set and presented by treatment group.

12.7.1. Primary Efficacy Analysis

The primary efficacy endpoint is the change from baseline to Month 12 in the total area of GA lesion(s) in eyes injected with pegcetacoplan, either monthly (PM) or every-other month (PEOM), or sham injections. GA lesion area (mm^2) as measured by a quantified central reading center based on FAF images. The primary analysis will be the comparison of pegcetacoplan, either monthly (PM) or every-other month (PEOM) versus the combined 2 sham arms (the 2 sham arms will be combined into a single 'control' group).

The null and alternative hypotheses for the primary efficacy analysis are:

H_0: $\mu_S = \mu_{PM}$ vs H_A: $\mu_S \neq \mu_{PM}$, and

H_0: $\mu_S = \mu_{PEOM}$ vs H_A: $\mu_S \neq \mu_{PEOM}$

Note: here μ indicates each group's respective mean change from baseline to Month 12 in GA lesion area for the comparison of the primary endpoint.

A mixed effect model for longitudinal data will be used to analyze the change from baseline in GA lesion area. The model will include treatment and presence of CNV in the fellow eye as fixed effects; baseline GA lesion area (at screening), time (in months) as a factor; as well as the time × treatment interaction term. All available data up to 12 months will be included in the model for the primary analysis. The mean change from baseline to 12 months will be estimated from the model (ie, least square [LS] mean) and compared between each of the pegcetacoplan arms to the sham control. For other time point of interest, LS mean change from baseline will be estimated and compared between treatments. Unstructured variance covariance will be used.

For the analysis of final study data, a similar model including data up to 24 months will be used and LS means at time points of interest will be estimated and compared between treatment.

As indicated in an earlier section, the study is expected to have an approximately 5 DMC data reviews. Allocating an alpha level of 0.0001 for each DMC data review, the alpha level remains for the efficacy analysis at 0.0495 to maintain an overall study alpha of 0.05.

The hypothesis testing strategy for the primary and secondary efficacy endpoints will be based on the Gate-keeping multiple testing procedures controlling for the study wide type I error strongly at 2-sided 0.0495 as follows:

Step 1. The mean GA lesion growth at 12 months will be compared between the PM group and the Control at the α level of 0.0495. If the null hypotheses of no difference between groups in this step is rejected, the testing proceeds to **Step 2** and **Step 3**. If it's not rejected, the testing procedure stops at this step.

Step 2. The mean GA lesion growth at 12 months will be compared between the PEOM group and the Control at the **α1** level. If the null hypotheses of no difference between groups in this step is rejected, the **α1** level will be passed down to **Step 3**. The actual value of **α1** will be specified in the SAP and it will be defined to ensure an adequate power of at least 80% for the comparison in this step.

Step 3. The mean GA lesion growth at 24 months will be compared between the PM group and the Control at the α level of **0.0495** if the null hypotheses are rejected at both **Step 1** and **Step 2**; or at the α level of **(0.0495 – α1)** if **Step 2** testing does not reject the null hypothesis. If the null hypothesis at this step is rejected, the α level used at this step will be passed down to the next step of testing. If it's not rejected, the testing procedure stops at this step.

Step 4. The prioritization and alpha allocation for the remaining secondary endpoints will be specified in the SAP.

The following sensitivity and supportive analyses will be performed to evaluate the robustness of the results from the primary analysis method:

- Analyses will be repeated using the mITT and PP sets
- Primary and secondary endpoints will also be summarized with no pooling of the 2 sham arms. The comparison for pegcetacoplan and sham injection within each dose regimen (ie, PM vs SM and PEOM vs SEOM) will be conducted
- Multiple imputation methods and other sensitivity analyses will be explored, and details will be provided in the SAP

12.7.2. Secondary Efficacy Analysis

The key secondary and secondary endpoints will be analyzed in the same fashion as the primary endpoint using mixed effect model. The binary secondary endpoints will be analyzed using Cochran-Mantel-Haenszel test stratified by the randomization stratification factors. Hypothesis testing for 3 key secondary endpoints will be performed in the order specified. The SAP will provide more details on multiplicity adjustment and the approach for alpha spending among the endpoints.

Key Secondary Efficacy Endpoints

- Change from baseline in maximum monocular reading speed (study eye), as assessed by MNREAD or Radner Reading Charts at Month 24 (in select countries)
- Change from baseline in FRI index score, at Month 24.
- Change from baseline in NL-BCVA at Month 24 as assessed by ETDRS chart.

Secondary Efficacy Endpoints

- Change from baseline in LL-BCVA at Month 12 and Month 24 as assessed by ETDRS chart.
- Change from baseline in LLD at Month 12 and Month 24.
- Change from baseline at each planned assessment in the total area of GA lesion(s) in the study eye (in mm^2) as assessed by FAF.

- Change from baseline in monocular critical print size (study eye), as assessed by MNREAD or Radner Reading Charts, at Month 12 and Month 24 (in select countries).
- Change from baseline in the NEI VFQ-25 distance activity subscale score at Month 12 and Month 24 (in select countries).
- Number of scotomatous points assessed by mesopic microperimetry for the evaluation of the macular functional response.
- Change in macular sensitivity as assessed by mesopic microperimetry for the evaluation of the macular functional response.

12.7.3. Exploratory Efficacy Analysis

Summary statistics will be provided for the following exploratory endpoints:

- Change from baseline in NEI VFQ-25 composite score at Month 12 and Month 24.
- Change from baseline in NEI VFQ-25 near activity subscale score at Month 12 and Month 24 (in select countries).
- Comparison between study eye and fellow eye in change in GA lesion size from baseline to Month 12 and Month 24.
- Binocular maximum reading speed as assessed by MNREAD or Radner Reading Charts over time (in select countries).
- Binocular critical print size as assessed by MNREAD or Radner Reading Charts over time (in select countries).
- Relationship between genetic polymorphisms associated with AMD with GA progression and response to pegcetacoplan.
- Incidence of new onset of subclinical CNV in the study eye.
- Assess sensitivity and specificity of a digital reading speed application to detect disease progression / regression (optional, select sites).
- Assess sensitivity and specificity of a digital visual function application to detect disease progression / regression (optional, select sites).

12.8. Safety Analyses

Adverse events will be collected from the time of the first study drug administration until a subject completes the study or discontinues prematurely. Treatment-emergent adverse events are defined as those AEs that develop or worsen after the first dose of study medication and up to 30 days beyond the last dose of study medication. The current version of MedDRA will be used to classify all AEs. Treatment-emergent adverse events will be summarized by System Organ Class and Preferred Term, in accordance with the MedDRA coding dictionary. The number of subjects reporting each AE Preferred Term will be tabulated for all TEAEs and separately for those considered as related to study treatment by the investigator or designee. Number of

subjects reporting SAEs will also be tabulated. Adverse event summaries will be presented for each treatment group separately.

Adverse events will be summarized by MedDRA coding terms, and separate tabulations also will be produced for related AEs (those considered by the investigator as definitively drug related), SAEs, and discontinuations due to AEs. Vital signs data and findings from physical and ophthalmologic examinations will be tabulated for changes over time on study. Laboratory parameters will be summarized for changes across study by using descriptive statistics. Separate summaries will be prepared for systemic (nonocular) and ocular AEs, with events in the study eye and nonstudy eye summarized separately

12.8.1. Anti-Therapeutic Antibodies

Data on anti-therapeutic antibodies (ATAs) directed against pegcetacoplan will be summarized by the number and percentage of subjects with confirmed positive ATAs.

12.8.2. Death

Patient deaths and primary cause of death will be summarized.

12.8.3. Ocular Assessments

Descriptive summaries will be generated for ocular assessments, such as VA and IOP.

12.9. Handling of Missing Data

All efforts will be made to minimize missing data. A full description of the imputation methods will be provided in the SAP.

12.10. Visit Windows

Analysis visits will be derived with windows for the monthly visits to assess the primary endpoint. Baseline is defined as the date of randomization. If 2 or more treatment visits occur within a window, the closest visit to the target day will be used as that analysis visit; if 2 visits are equidistant from the scheduled analysis visit day, the later analysis visit will be used.

13. ETHICS

13.1. Ethical Conduct of the Study

This research will be carried out in accordance with the protocol, applicable regulations, the ethical principles set forth in the Declaration of Helsinki, and the ICH Harmonized Tripartite Guidance for Good Clinical Practice, E6, R1 (ICH GCP).

13.2. Institutional Review Board/Ethic Committee

The study protocol, any amendments to the protocol, ICF, the IB, and other study-specific information will be reviewed and approved by the IRB/IEC. The study will not be initiated until the IRB/IEC has approved the protocol or a modification thereof. All records pertaining to IRB/IEC submission and approval should be kept in the site's regulatory files and sponsor's Trial Master File.

The IRB/IEC must be constituted and operate in accordance with the principles and requirements described in ICH Guidance E6 and national and local regulations as deemed appropriate.

13.3. Subject Information and Consent

The principal investigator, or designee, is responsible for obtaining an informed consent. A written informed consent, in compliance with ICH Guidance E6, must be obtained from each subject at the screening visit, prior to performing any study-related procedures.

The purpose of the study, the procedures to be carried out, and the potential hazards will be described to the subjects in nontechnical terms. The subject will be given sufficient time to consider the study's implications before deciding to participate in the study. The subject and/or legal guardian will be required to sign and date an ICF and will be assured that they may withdraw from the study at any time without jeopardizing their medical care. The PI shall retain the original, signed informed consent for study participation in the subject's medical record and shall provide the subject and/or legal guardian with a copy of the signed consent.

If there are any changes/amendments to the approved protocol, which may directly affect the subject's decision to continue participation in the study, the ICF shall be amended to incorporate the changes to the protocol and the subject must re-sign the IRB/IEC approved amended ICF.

14. ADMINISTRATIVE CONSIDERATIONS

14.1. Direct Access to Source Data/Documents

The principal investigator, or designee, must maintain, at all times, the primary records (ie, source documents) of each subject's data for data verification. Examples of source documents are medical records, laboratory reports, study drug records, and printed CRF pages that are used as the source.

The investigator will permit trial-related monitoring, audits, and inspections by the sponsor and/or its designee, IRB/IEC, and the regulatory agencies at any time during the study. The investigator will ensure that the auditor is allowed direct access to the source data, medical records, eCRFs, and the site's regulatory file for the study and any other pertinent information.

14.2. Quality Control and Quality Assurance

This study is to be performed in full compliance with the protocol, GCP, and applicable regulatory requirements. The principal investigator, sponsor, and/or its designee are responsible for ensuring that the study staff receive appropriate training on the protocol, study procedures, and any other relevant information.

Quality assurance and quality control systems are implemented and maintained using written Investigative site, sponsor and/or designee Standard Operating Procedures (SOPs) to ensure that the study is conducted and data are generated, documented (recorded), and reported in compliance with the protocol, GCP, and the applicable regulatory requirement(s) and local laws, rules, regulations.

Quality control checks will be applied at each stage of data handling (eg, edit checks) to ensure that all data are reliable and have been processed correctly.

14.3. Monitoring

On-site monitoring will be performed by the sponsor's designee for the duration of the study. The monitor will ensure that the study is conducted, recorded, and reported in accordance with the protocol, SOPs, GCP, and the applicable regulatory requirements. The monitor will verify the accuracy and completeness of the eCRF entries, source documents, and other study-related records against each other. The investigator, or designee, will provide direct access to source data/documents for study-related monitoring. It is important that the investigator and the investigator site staff are available at these visits. The monitor will record the date of each visit together with a summary of the status and progress of the study. Proposed actions will be documented in writing to the investigator.

14.4. Data Handling and Record Keeping

The investigator must maintain all documentation related to this study. All essential documents (as defined in the ICH Guideline E6 and applicable local regulations) and the data generated in connection with this study, together with the original copy of the final report, will be retained for at least 2 years after the last approval of a marketing application in an ICH region and until there

are no pending or contemplated marketing applications in an ICH region or at least 2 years have elapsed since the formal discontinuation of clinical development of the IP. These documents should be retained for a longer period if required by the applicable regulatory requirements or by an agreement with the sponsor.

It is the responsibility of the sponsor to inform the investigator/institution as to when these documents no longer need to be retained.

14.5. Protocol Amendments

Any amendments to the study protocol deemed necessary as the study progresses will be discussed between sponsor and the investigator. The investigator will not implement any changes to the protocol without an agreement by the sponsor and prior review and documented approval/favorable opinion from the IRB/IEC of an amendment, except where necessary to eliminate immediate hazards to study subject or when the changes involve only logistical or administrative aspects of the study (eg, change in staff, telephone numbers).

Changes resulting in amendments will be made jointly between the sponsor and the investigator and must be confirmed in writing. Amendment(s) will be approved and signed off in the same way as the protocol.

14.6. Report Format

According to the ICH Harmonized Tripartite Guideline (Organization of the Common Technical Document for the Registration of Pharmaceuticals for Human Use M4 and the ICH M2 Expert Working Group), the final report will be written according to the ICH E3 Guideline (Structure and Content of Clinical Study Reports).

14.7. Finance and Insurance

Finance and insurance will be addressed in a Clinical Trial Agreement between the sponsor and the investigator/institution.

14.8. Publication Policy

The data generated for this study are considered confidential information and are the property of the sponsor. All study information provided to the PI and site personnel by the sponsor shall not be published or disclosed to a third party without the prior written consent of the sponsor.

Apellis will endeavor to publish the results of all qualifying, applicable, and covered studies according to external guidelines in a timely manner regardless of whether the outcomes are perceived as positive, neutral, or negative. Additionally, Apellis adheres to external guidelines (eg, Good Publication Practices 2) when forming a publication steering committee, which is done for large, multicenter Phase 2-4 and certain other studies as determined by Apellis. The purpose of the publication steering committee is to act as a noncommercial body that advises or decides on dissemination of scientific study data in accordance with the scope of this policy.

All publications relating to Apellis products or projects must undergo appropriate technical and intellectual property review, with Apellis agreement to publish prior to release of information. The review is aimed at protecting the sponsor's proprietary information existing either at the

commencement of the study or generated during the study. To the extent permitted by the publisher and copyright law, the investigator will own (or share with other authors) the copyright on his/her publications. To the extent that the investigator has such sole, joint, or shared rights, the investigator grants the sponsor a perpetual, irrevocable, royalty-free license to make and distribute copies of such publications.

The term “publication” refers to any public disclosure including original research articles, review articles, oral presentations, abstracts and posters at medical congresses, journal supplements, letters to the editor, invited lectures, opinion pieces, book chapters, electronic postings on medical/scientific websites, or other disclosure of the study results, in printed, electronic, oral, or other form.

Subject to the terms of the paragraph below, the investigator shall have the right to publish the study results, and any background information provided by the sponsor that is necessary to include in any publication of study results, or necessary for other scholars to verify such study results. Notwithstanding the foregoing, no publication that incorporates the sponsor’s confidential information shall be submitted for publication without the sponsor’s prior written agreement to publish and shall be given to the sponsor for review at least 60 days prior to submission for publication. If requested in writing by Apellis, the institution and investigator shall withhold submission of such publication for up to an additional 60 days to allow for filing of a patent application.

If the study is part of a multicenter study, the first publication of the study results shall be made by the sponsor in conjunction with the sponsor’s presentation of a joint, multicenter publication of the compiled and analyzed study results. If such a multicenter publication is not submitted to a journal for publication by the sponsor within an 18-month period after conclusion, abandonment, or termination of the study at all sites, or after the sponsor confirms there shall be no multicenter study publication of the study results, an investigator may individually publish the study results from the specific site in accordance with this section. The investigator must, however, acknowledge in the publication the limitations of the single-site data being presented.

Unless otherwise required by the journal in which the publication appears, or the forum in which it is made, authorship will comply with the International Committee of Medical Journal Editors current standards. Participation as an investigator does not confer any rights to authorship of publications.

14.9. ClinicalTrials.gov

This study will be listed with ClinicalTrials.gov.

14.10. Termination of Study

The sponsor reserves the right to suspend or discontinue this study for administrative and/or safety reasons at any time. The investigator reserves the right to discontinue dosing subjects at any time for safety reasons.

15. REFERENCES

1. Holz FG, Bindewald-Wittich A, Fleckenstein M, et al. Progression of geographic atrophy and impact of fundus autofluorescence patterns in age-related macular degeneration. *American journal of ophthalmology.* 2007;143(3):463-472.
2. Rein DB, Wittenborn JS, Zhang X, et al. Forecasting age-related macular degeneration through the year 2050: the potential impact of new treatments. *Archives of ophthalmology.* 2009;127(4):533-540.
3. Friedman DS, O'Colmain BJ, Munoz B, et al. Prevalence of age-related macular degeneration in the United States. *Archives of ophthalmology.* 2004;122(4):564-572.
4. Holz FG, Strauss EC, Schmitz-Valckenberg S, van Lookeren Campagne M. Geographic atrophy: clinical features and potential therapeutic approaches. *Ophthalmology.* 2014;121(5):1079-1091.
5. Sunness JS, Bressler NM, Tian Y, Alexander J, Applegate CA. Measuring geographic atrophy in advanced age-related macular degeneration. *Investigative ophthalmology & visual science.* 1999;40(8):1761-1769.
6. Sunness JS, Gonzalez-Baron J, Applegate CA, et al. Enlargement of atrophy and visual acuity loss in the geographic atrophy form of age-related macular degeneration. *Ophthalmology.* 1999;106(9):1768-1779.
7. Crabb JW, Miyagi M, Gu X, et al. Drusen proteome analysis: an approach to the etiology of age-related macular degeneration. *Proceedings of the National Academy of Sciences of the United States of America.* 2002;99(23):14682-14687.
8. Hageman GS, Anderson DH, Johnson LV, et al. A common haplotype in the complement regulatory gene factor H (HF1/CFH) predisposes individuals to age-related macular degeneration. *Proceedings of the National Academy of Sciences of the United States of America.* 2005;102(20):7227-7232.
9. Lommatzsch A, Hermans P, Muller KD, Bornfeld N, Bird AC, Pauleikhoff D. Are low inflammatory reactions involved in exudative age-related macular degeneration? Morphological and immunhistochemical analysis of AMD associated with basal deposits. *Graefe's archive for clinical and experimental ophthalmology = Albrecht von Graefes Archiv fur klinische und experimentelle Ophthalmologie.* 2008;246(6):803-810.
10. Mullins RF, Russell SR, Anderson DH, Hageman GS. Drusen associated with aging and age-related macular degeneration contain proteins common to extracellular deposits associated with atherosclerosis, elastosis, amyloidosis, and dense deposit disease. *FASEB journal : official publication of the Federation of American Societies for Experimental Biology.* 2000;14(7):835-846.
11. Edwards AO, Ritter R, 3rd, Abel KJ, Manning A, Panhuysen C, Farrer LA. Complement factor H polymorphism and age-related macular degeneration. *Science.* 2005;308(5720):421-424.
12. Haines JL, Hauser MA, Schmidt S, et al. Complement factor H variant increases the risk of age-related macular degeneration. *Science.* 2005;308(5720):419-421.

13. Klein RJ, Zeiss C, Chew EY, et al. Complement factor H polymorphism in age-related macular degeneration. *Science.* 2005;308(5720):385-389.

14. Maller J, George S, Purcell S, et al. Common variation in three genes, including a noncoding variant in CFH, strongly influences risk of age-related macular degeneration. *Nature genetics.* 2006;38(9):1055-1059.

15. Seddon JM, Yu Y, Miller EC, et al. Rare variants in CFI, C3 and C9 are associated with high risk of advanced age-related macular degeneration. *Nature genetics.* 2013;45(11):1366-1370.

16. Yates JR, Sepp T, Matharu BK, et al. Complement C3 variant and the risk of age-related macular degeneration. *The New England journal of medicine.* 2007;357(6):553-561.

17. van de Ven JP, Nilsson SC, Tan PL, et al. A functional variant in the CFI gene confers a high risk of age-related macular degeneration. *Nature genetics.* 2013;45(7):813-817.

18. Fritsche LG, Chen W, Schu M, et al. Seven new loci associated with age-related macular degeneration. *Nature genetics.* 2013;45(4):433-439, 439e431-432.

19. Gold B, Merriam JE, Zernant J, et al. Variation in factor B (BF) and complement component 2 (C2) genes is associated with age-related macular degeneration. *Nature genetics.* 2006;38(4):458-462.

20. Horie-Inoue K, Inoue S. Genomic aspects of age-related macular degeneration. *Biochemical and biophysical research communications.* 2014;452(2):263-275.

21. Hecker LA, Edwards AO. Genetic control of complement activation in humans and age related macular degeneration. *Advances in experimental medicine and biology.* 2010;703:49-62.

22. Hecker LA, Edwards AO, Ryu E, et al. Genetic control of the alternative pathway of complement in humans and age-related macular degeneration. *Human molecular genetics.* 2010;19(1):209-215.

23. Machalinska A, Dziedziejko V, Mozolewska-Piotrowska K, Karczewicz D, Wiszniewska B, Machalinski B. Elevated plasma levels of C3a complement compound in the exudative form of age-related macular degeneration. *Ophthalmic research.* 2009;42(1):54-59.

24. Scholl HP, Charbel Issa P, Walier M, et al. Systemic complement activation in age-related macular degeneration. *PloS one.* 2008;3(7):e2593.

25. Leeds JM, Henry SP, Bistner S, Scherrill S, Williams K, Levin AA. Pharmacokinetics of an antisense oligonucleotide injected intravitreally in monkeys. *Drug metabolism and disposition: the biological fate of chemicals.* 1998;26(7):670-675.

26. Fleckenstein M, Mitchell P, Freund KB, et al. The Progression of Geographic Atrophy Secondary to Age-Related Macular Degeneration. *Ophthalmology*. 2018; 125(3):369-390.

16. APPENDICES

APPENDIX A: VISIT SCHEDULE—Monthly Group—Screening, Day 1 through Month 12

	Screening	Treatment													Early Term[A]
**Visit #**	1	2	3	4	5	6	7	8	9	10	11	12	13	14	
**Day**	–28 to –1	1	30	60	90	120	150	180	210	240	270	300	330	360	
**Week**	0	0	4	8	12	16	20	24	28	32	36	40	44	48	
**Month**	0	0	1	2	3	4	5	6	7	8	9	10	11	12	
**Window (+ or – days)**	2	0	8	8	8	8	8	8	8	8	8	8	8	8	
**Informed consent / assign screening number**	x														
**Demographic data**	x														
**Inclusion/exclusion criteria** [B]	x	x													
**Medical/surgical/ocular history** [C]	x														
**Blood draw—safety labs** [D,E,F]	x	x		x				x						x	x
**Urine sample collection** [D, E,F]	x	x		x				x						x	x
**Urine pregnancy test** [D,E,F]		x	x	x	x	x	x	x	x	x	x	x	x	x	
**Blood draw—anti-pegcetacoplan Ab** [D]		x	x	x				x						x	x
**Blood draw—genotyping (if applicable)** [D]				x											
**Blood draw for clinical repository (if applicable)**[D,G]				x				x						x	x
**Vital signs** [H]	x	x	x	x	x	x	x	x	x	x	x	x	x	x	x
**Physical examination** [I]	x													x	x
**BCVA** [J]	x	x	x	x	x	x	x	x	x	x	x	x	x	x	x
**LL-BCVA** [J]		x	x	x	x	x	x	x	x	x	x	x	x	x	x
**MNREAD or Radner Reading Charts (select countries)** [J,K]		x						x						x	x
**Mesopic microperimetry**[L]	x							SE						x	x
**Slitlamp examination**	x	x	x	x	x	x	x	x	x	x	x	x	x	x	x
**Endothelial cell count**[S]		x						x						x	x
**NEI VFQ-25** [M]		x						x						x	x
**FRI** [M]		x						x						x	x
**Home-based digital applications** [M,N,S]		x	x	x	x			x						x	
**Dilated indirect ophthalmoscopy**	x	x	x	x	x	x	x	x	x	x	x	x	x	x	x
**IOP measurement**	x	x	x	x	x	x	x	x	x	x	x	x	x	x	x

	Screening	Treatment													Early Term[A]
Visit #	1	2	3	4	5	6	7	8	9	10	11	12	13	14	
Day	–28 to –1	1	30	60	90	120	150	180	210	240	270	300	330	360	
Week	0	0	4	8	12	16	20	24	28	32	36	40	44	48	
Month	0	0	1	2	3	4	5	6	7	8	9	10	11	12	
Window (+ or – days)	2	0	8	8	8	8	8	8	8	8	8	8	8	8	
SD-OCT [O]	x	x	x	x	x	x	x	x	x	x	x	x	x	x	x
FAF [O]	x	x		SE		SE		x		SE		SE		x	x
NIR [O]	x	x		SE		SE		x		SE		SE		x	x
DCFP [O]	x													x	x
FFA [O]	x													x	x
OCT-A[S]		x[S]						x[S]						x[S]	x
Study eye determination	x														
Randomization		x													
Pegcetacoplan administration or Sham Injection[T]		x	x	x	x	x	x	x	x	x	x	x	x	x	
Postinjection assessment [P]		x	x	x	x	x	x	x	x	x	x	x	x	x	
Follow-up call [Q]		x	x	x	x										
Concomitant medication/ concomitant ocular procedures [R]	x	x	x	x	x	x	x	x	x	x	x	x	x	x	x
Adverse events	x	x	x	x	x	x	x	x	x	x	x	x	x	x	x

Abbreviations: BCVA=best corrected visual acuity; CNV=choroidal neovascularization; DCFP=digital color fundus photography; eCRF=electronic case report form; FAF=fundus autofluorescence; FFA=fundus fluorescein angiography; FRI=Functional Reading Independence index; IOP=intraocular pressure; LL BCVA= low luminance best corrected visual acuity; MNREAD=Minnesota Low-Vision Reading Test; NEI VFG-25=National Eye Institute Visual Functioning Questionnaire 25-item Version; NIR=near infrared reflectance; OCT-A=optical coherence tomography angiography; SD-OCT=spectral domain optical coherence tomography; SE=study eye; Term=termination; VEGF=vascular endothelial growth factor.

Note: All ocular assessments are to be performed for both eyes unless annotated with 'SE' (study eye) in the above schedule. All assessments should be performed on the same day. All study visits should be scheduled and projected based on the Day 1 visit date.

A. For subjects that discontinue the study early, the early termination assessments should be performed after a minimum of 30 days have passed from the last dosing visit. If a subject reports for a scheduled visit and decides to terminate early prior to dosing, then the visit should be considered the early termination visit and all early termination procedures should be performed. At Month 24, all subjects should be offered entry into an open-label study.

B. At Day 1 (Visit 2), confirm subject eligibility through reviewing the inclusion/ exculsion criteria and receive confirmation of eligibility from the reading center.

C. Significant medical/ surgical history from the previous 5 years. Anti-VEGF treatments (fellow eye) and invasive ocular procedures performed within the past 5 years and while on study should also be recorded. Any history of tobacco use should be recorded.

D. Obtain prior to fluorescein angiography and before study drug administration.

E. At screening, serum pregnancy should be performed for women of childbearing potential. If positive, subject is not eligible to continue in the study.

F. Beginning at Day 1, perform the urine pregnancy test for women of childbearing potential at each treatment visit. If positive, perform a serum pregnancy test. If serum test is positive, study drug should not be administered and an early term visit should be completed.

G. Only subjects that sign the separate consent for the clinical repository and genotyping will have these samples collected. A 14-mL whole-blood sample will be collected at each of the specified visits.
H. Blood pressure, respiratory rate, heart rate, and temperature. On dosing days, vital signs should be taken predose.
I. Height and weight should be collected at screening.
J. Perform assessments prior to dilating the eyes.
K. In select countries, the MNREAD or Radner Reading Charts should be done during the study visit, prior to dilating the eyes. It should be performed monocularly first, then binocularly.
L. Microperimetry assessments will be performed post dilation. Data will be forwarded to the reading center.
M. To be administered by the masked site staff prior to any other assessments performed on that day. In-clinic assessments on the digital application should be completed after completion of all functional tests and quality of life measures prior to dilating the eyes.
N. At select sites, and for those subjects who decide to participate, the digital applications will be completed on an electronic device and will consist of assessments for visual function and reading speed. Subjects will receive training at Day 1 on the use of the digital applications and the electronic device and will perform the tests using the application in the clinic at Month 1, Month 2, Month 3, Month 6, Month 12, Month 18, and Month 24. Beginning at Day 1, subjects will complete the functional assessments weekly at home. Subjects should be instructed to complete the assessments on the same day each week and at approximately the same time of day each week, if possible.
O. FAF and SD-OCT images, near infrared reflectance, fluorescein angiograms, and fundus photographs will be performed for the study eye only on days where 'SE' is specified and for both eyes at all other visits as specified in the above schedule and will be sent to the reading center for evaluation (consult the reading center manual for specifics on image capture, processing, and transmission). Images should be captured prior to dosing on dosing days. If a subject misses a study visit or images cannot be obtained at a specific visit, study staff should make every effort to obtain images at the next scheduled visit. If new active CNV is suspected, SD-OCT, FFA and OCT-A (select sites) images must be collected and sent to the reading center for analysis.
P. Postinjection assessments should be performed within 5 minutes after dosing by the unmasked physician or study staff and should include a gross assessment of vision (finger-counting, hand-motion, then light perception when applicable). If subject passes gross vision test, the subject may leave the site. If subject fails gross vision test, the tonometry should be performed. IOP should be ≤30 mm Hg in order for the subject to leave the site. If necessary, antiglaucomatous medication can be given to lower the IOP. If IOP is >30 mm Hg, assessments will continue every approximately 30 minutes from the previous measurement until the subject passes gross vision test and IOP is ≤30 mm Hg. Note: if the study eye is treated with a ranibizumab or aflibercept injection during the same visit as the study treatment (pegcetacoplan or sham), the treatment with ranibizumab or aflibercept must be performed first. The pre- and post- anti-VEGF IOP values must be measured and recorded on the eCRF.
Q. Starting at Day 1, study subjects will be contacted by study site staff within 4 ± 2 days after each study treatment visit (through Month 3) to collect any information on any safety concerns, decrease in vision, eye pain, unusual ocular events, or any new ocular symptoms in the study eye. If the physician determines that there are any safety concerns, a follow-up visit should be scheduled as soon as possible.
R. Record concomitant medications (ie, prescription and over-the-counter medications) used by the patient within 30 days of screening and throughout the subject's participation in the study.
S. In addition to the time points indicated on the study schedule, OCT-A should be performed at the time of any suspected new active CNV. If new active CNV is confirmed in the study eye, OCT-A should be repeated every 2 months for the study eye.
T. Administration of pegcetacoplan or sham can be done on separate days from the assessment visit if both days fall within the visit window. Administration of study treatment (pegcetacoplan or sham) can be done on a separate day from the assessment visit if both days fall within the visit window. If this occurs on the randomization visit, then the administration of pegcetacoplan or sham should be done within 3 days of randomization and after approval from the medical monitor. When study treatment administration is on a day other than a study visit, then the only assessment that must be done on the day of study treatment administration is preinjection IOP.

APPENDIX B: VISIT SCHEDULE—Monthly Group—Month 13 to Month 24

	Treatment												Early Term[A]
**Visit #**	15	16	17	18	19	20	21	22	23	24	25	26	
**Day**	390	420	450	480	510	540	570	600	630	660	690	720	
**Week**	52	56	60	64	68	72	76	80	84	88	92	96	
**Month**	13	14	15	16	17	18	19	20	21	22	23	24	
**Window (+ or – days)**	8	8	8	8	8	8	8	8	8	8	8	8	
**Informed consent / assign screening number**													
**Demographic data**													
**Inclusion/exclusion criteria [B]**													
**Medical/surgical/ocular history [C]**													
**Blood draw—safety labs [D,E,F]**						x						x	x
**Urine sample collection [D,E,F]**						x						x	x
**Urine pregnancy test [D,E,F]**	x	x	x	x	x	x	x	x	x	x	x	x	
**Blood draw—anti-pegcetacoplan Ab [D]**		x				x						x	x
**Blood draw—genotyping (if applicable) [D]**													
**Blood draw for clinical repository (if applicable)[D,G]**												x	x
**Vital signs [H]**	x	x	x	x	x	x	x	x	x	x	x	x	x
**Physical examination [I]**												x	x
**BCVA [J]**	x	x	x	x	x	x	x	x	x	x	x	x	x
**LL-BCVA [I]**	x	x	x	x	x	x	x	x	x	x	x	x	x
**MNREAD or Radner Reading Charts (select countries) [J,K]**						x						x	x
**Mesopic microperimetry[L]**						SE						x	x
**Slitlamp examination**	x	x	x	x	x	x	x	x	x	x	x	x	x
**Endothelial cell count[S]**												x	x
**NEI VFQ-25 [M]**						x						x	x
**FRI [M]**						x						x	x
**Dilated Indirect Ophthalmoscopy**	x	x	x	x	x	x	x	x	x	x	x	x	x
**Home-based digital applications [M,N,S]**						x						x	
**IOP measurement**	x	x	x	x	x	x	x	x	x	x	x	x	x
**SD-OCT [O]**	x	x	x	x	x	x	x	x	x	x	x	x	x

	Treatment												Early Term[A]
**Visit #**	**15**	**16**	**17**	**18**	**19**	**20**	**21**	**22**	**23**	**24**	**25**	**26**	
**Day**	**390**	**420**	**450**	**480**	**510**	**540**	**570**	**600**	**630**	**660**	**690**	**720**	
**Week**	**52**	**56**	**60**	**64**	**68**	**72**	**76**	**80**	**84**	**88**	**92**	**96**	
**Month**	**13**	**14**	**15**	**16**	**17**	**18**	**19**	**20**	**21**	**22**	**23**	**24**	
**Window (+ or – days)**	**8**	**8**	**8**	**8**	**8**	**8**	**8**	**8**	**8**	**8**	**8**	**8**	
**FAF [O]**		SE		SE		x		SE		SE		x	x
**NIR [O]**		SE		SE		x		SE		SE		x	x
**DCFP [O]**												x	x
**FFA [O]**												x	x
**OCT-A[S]**						x[S]						x[S]	x
**Study eye determination**													
**Randomization**													
**Pegcetacoplan administration or Sham Injection[T]**	x	x	x	x	x	x	x	x	x	x	x		
**Postinjection assessment [P]**	x	x	x	x	x	x	x	x	x	x	x		
**Follow-up call [Q]**													
**Concomitant medication/ concomitant ocular procedures [R]**	x	x	x	x	x	x	x	x	x	x	x	x	x
**Adverse events**	x	x	x	x	x	x	x	x	x	x	x	x	x

Abbreviations: BCVA=best corrected visual acuity; CNV=choroidal neovascularization; DCFP=digital color fundus photography; eCRF=electronic case report form; FAF=fundus autofluorescence; FFA=fundus fluorescein angiography; FRI=Functional Reading Independence index; IOP=intraocular pressure; LL BCVA= low luminance best corrected visual acuity; MNREAD=Minnesota Low-Vision Reading Test; NEI VFG-25=National Eye Institute Visual Functioning Questionnaire 25-item Version; NIR=near infrared reflectance; OCT-A=optical coherence tomography angiography; SD-OCT=spectral domain optical coherence tomography; SE=study eye; Term=termination; VEGF=vascular endothelial growth factor.

Note: All ocular assessments are to be performed for both eyes unless annotated with 'SE' (study eye) in the above schedule. All assessments should be performed on the same day. All study visits should be scheduled and projected based on the Day 1 visit date.

A. For subjects that discontinue the study early, the early termination assessments should be performed after a minimum of 30 days have passed from the last dosing visit. If a subject reports for a scheduled visit and decides to terminate early prior to dosing, then the visit should be considered the early termination visit and all early termination procedures should be performed. At Month 24, all subjects should be offered entry into an open-label study.

B. At Day 1 (Visit 2), confirm subject eligibility through reviewing the inclusion/ exculsion criteria and receive confirmation of eligibility from the reading center.

C. Significant medical/ surgical history from the previous 5 years. Anti-VEGF treatments (fellow eye) and invasive ocular procedures performed within the past 5 years and while on study should also be recorded. Any history of tobacco use should be recorded.

D. Obtain prior to fluorescein angiography and before study drug administration.

E. At screening, serum pregnancy should be performed for women of childbearing potential. If positive, subject is not eligible to continue in the study.

F. Beginning at Day 1, perform the urine pregnancy test for women of childbearing potential at each treatment visit. If positive, perform a serum pregnancy test. If serum test is positive, study drug should not be administered and an early term visit should be completed.

G. Only subjects that sign the separate consent for the clinical repository and genotyping will have these samples collected. A 14-mL whole-blood sample will be collected at each of the specified visits.

H. Blood pressure, respiratory rate, heart rate, and temperature. On dosing days, vital signs should be taken predose.

I. Height and weight should be measured at screening.
J. Perform assessments prior to dilating the eyes.
K. In select countries, the MNREAD or Radner Reading Charts should be done during the study visit, prior to dilating the eyes. It should be performed monocularly first, then binocularly.
L. Microperimetry assessments will be performed post dilation. Data will be forwarded to the reading center.
M. To be administered by the masked site staff prior to any other assessments performed on that day. In-clinic assessments on the digital application should be completed after completion of all functional tests and quality of life measures prior to dilating the eyes.
N. At select sites, and for those subjects who decide to participate, the digital applications will be completed on an electronic device and will consist of assessments for visual function and reading speed. Subjects will receive training at Day1 on the use of the digital applications and the electronic device and will perform the tests using the application in the clinic at Month 1, Month 2, Month 3, Month 6, Month 12, Month 18, and Month 24. Beginning at Day 1, subjects will complete the functional assessments weekly at home. Subjects should be instructed to complete the assessments on the same day each week and at approximately the same time of day each week, if possible.
O. FAF and SD-OCT images, near infrared reflectance, fluorescein angiograms, and fundus photographs will be performed for the study eye only on days where 'SE' is specified and for both eyes at all other visits as specified in the above schedule and will be sent to the reading center for evaluation (consult the reading center manual for specifics on image capture, processing, and transmission). Images should be captured prior to dosing on dosing days. If a subject misses a study visit or images cannot be obtained at a specific visit, study staff should make every effort to obtain images at the next scheduled visit. If new active CNV is suspected, SD-OCT, FFA, and OCT-A (selected sites) images should be collected and sent to the reading center for analysis.
P. Postinjection assessments should be performed within 5 minutes after dosing by the unmasked physician or study staff and should include a gross assessment of vision (finger-counting, hand motion, then light perception when applicable). If subject passes gross vision test, the subject may leave the site. If subject fails gross vision test, the tonometry should be performed. IOP should be ≤30 mm Hg in order for the subject to leave the site. If necessary, antiglaucomatous medication can be given to lower the IOP. If IOP is >30 mm Hg, assessments will continue every approximately 30 minutes from the previous measurement until the subject passes gross vision test and IOP is ≤30 mm Hg. Note: if the study eye is treated with a ranibizumab or aflibercept injection during the same visit as the study treatment (pegcetacoplan or sham), the treatment with ranibizumab or aflibercept must be performed first. The pre- and post- anti-VEGF IOP values must be measured and recorded on the eCRF.
Q. Starting at Day 1, study subjects will be contacted by study site staff within 4 ± 2 days after each study treatment visit (through Month 3) to collect any information on any safety concerns, decrease in vision, eye pain, unusual ocular events, or any new ocular symptoms in the study eye. If the physician determines that there are any safety concerns, a follow-up visit should be scheduled as soon as possible.
R. Record concomitant medications (ie, prescription and over-the-counter medications) used by the patient within 30 days of screening and throughout the subject's participation in the study.
S. In addition to the time points indicated on the study schedule, OCT-A should be performed at the time of any suspected new active CNV. If new active CNV is confirmed in the study eye, OCT-A should be repeated every 2 months for the study eye.
T. Administration of pegcetacoplan or sham can be done on separate days from the assessment visit if both days fall within the visit window. Administration of study treatment (pegcetacoplan or sham) can be done on a separate day from the assessment visit if both days fall within the visit window. If this occurs on the randomization visit, then the administration of pegcetacoplan or sham should be done within 3 days of randomization and after approval from the medical monitor. When study treatment administration is on a day other than a study visit, then the only assessment that must be done on the day of study treatment administration is preinjection IOP.

APPENDIX C: VISIT SCHEDULE—Every-Other-Month Group—Screening, Day 1 Through Month 12

	Screening	Treatment													Early Term[A]
Visit #	1	2	3	4	5	6	7	8	9	10	11	12	13	14	
Day	–28 to –1	1	30	60	90	120	150	180	210	240	270	300	330	360	
Week	0	0	4	8	12	16	20	24	28	32	36	40	44	48	
Month	0	0	1	2	3	4	5	6	7	8	9	10	11	12	
Window (+ or – days)	2	0	8	8	8	8	8	8	8	8	8	8	8	8	
Informed consent / assign screening number	x														
Demographic data	x														
Inclusion/exclusion criteria [B]	x	x													
Medical/surgical/ocular history [C]	x														
Blood draw—safety labs [D,E,F]	x	x		x				x						x	x
Urine sample collection [D,E,F]	x	x		x				x						x	x
Urine pregnancy test [D,E,F]		x		x		x		x		x		x		x	
Blood draw—anti–pegcetacoplan Ab [D]		x	x	x				x						x	x
Blood draw—genotyping (if applicable) [D]				x											
Blood draw for clinical repository (if applicable) [D,G]				x				x						x	x
Vital signs [H]	x	x	x	x	x	x	x	x	x	x	x	x	x	x	x
Physical examination [I]	x													x	x
BCVA [J]	x	x	x	x	x	x	x	x	x	x	x	x	x	x	x
LL-BCVA [J]		x	x	x	x	x	x	x	x	x	x	x	x	x	x
MNREAD or Radner Reading Charts (select countries) [J,K]		x						x						x	x
Mesopic microperimetry[L]	x							SE						x	x
Slitlamp examination	x	x	x	x	x	x	x	x	x	x	x	x	x	x	x
Endothelial cell count[S]		x						x						x	x
NEI VFQ-25 [M]		x						x						x	x
FRI [M]		x						x						x	x
Home-based digital applications [M,N,S]		x	x	x	x			x						x	
Dilated indirect ophthalmoscopy	x	x	x	x	x	x	x	x	x	x	x	x	x	x	x
IOP measurement	x	x	x	x	x	x	x	x	x	x	x	x	x	x	x
SD-OCT [O]	x	x	x	x	x	x	x	x	x	x	x	x	x	x	x
FAF [O]	x	x		SE		SE		x		SE		SE		x	x

	Screening	Treatment													Early Term[A]
**Visit #**	1	2	3	4	5	6	7	8	9	10	11	12	13	14	
**Day**	–28 to –1	1	30	60	90	120	150	180	210	240	270	300	330	360	
**Week**	0	0	4	8	12	16	20	24	28	32	36	40	44	48	
**Month**	0	0	1	2	3	4	5	6	7	8	9	10	11	12	
**Window (+ or – days)**	2	0	8	8	8	8	8	8	8	8	8	8	8	8	
**NIR [O]**	x	x		SE		SE		x		SE		SE		x	x
**DCFP [O]**	x													x	x
**FFA [O]**	x													x	x
**OCT-A[S]**		x[S]						x[S]						x[S]	x
**Study eye determination**	x														
**Randomization**		x													
**Pegcetacoplan administration or Sham Injection[T]**		x		x		x		x		x		x		x	
**Postinjection assessment [P]**		x		x		x		x		x		x		x	
**Follow-up call [Q]**		x		x		x									
**Concomitant medication/ concomitant ocular procedures [R]**	x	x	x	x	x	x	x	x	x	x	x	x	x	x	x
**Adverse events**	x	x	x	x	x	x	x	x	x	x	x	x	x	x	x

Abbreviations: BCVA=best corrected visual acuity; CNV=choroidal neovascularization; DCFP=digital color fundus photography; eCRF=electronic case report form; FAF=fundus autofluorescence; FFA=fundus fluorescein angiography; FRI=Functional Reading Independence index; IOP=intraocular pressure; LL BCVA= low luminance best corrected visual acuity; MNREAD=Minnesota Low-Vision Reading Test; NEI VFG-25=National Eye Institute Visual Functioning Questionnaire 25-item Version; NIR=near infrared reflectance; OCT-A=optical coherence tomography angiography; SD-OCT=spectral domain optical coherence tomography; SE=study eye; Term=termination; VEGF=vascular endothelial growth factor.

Note: All ocular assessments are to be performed for both eyes unless annotated with 'SE' (study eye) in the above schedule. All assessments should be performed on the same day. All study visits should be scheduled and projected based on the Day 1 visit date.

A. For subjects that discontinue the study early, the early termination assessments should be performed after a minimum of 30 days have passed from the last dosing visit. If a subject reports for a scheduled visit and decides to terminate early prior to dosing, then the visit should be considered the early termination visit and all early termination procedures should be performed. At Month 24, all subjects should be offered entry into an open-label study.

B. At Day 1 (Visit 2), confirm subject eligibility through reviewing the inclusion/exclusion criteria and receive confirmation of eligibility from the reading center.

C. Significant medical/ surgical history from the previous 5 years. Anti-VEGF treatments (fellow eye) and invasive ocular procedures performed within the past 5 years and while on study should also be recorded. Any history of tobacco use should be recorded.

D. Obtain prior to fluorescein angiography and before study drug administration.

E. At screening, serum pregnancy should be performed for women of childbearing potential. If positive, subject is not eligible to continue in the study.

F. Beginning at Day 1, perform the urine pregnancy test for women of childbearing potential at each treatment visit. If positive, perform a serum pregnancy test. If serum test is positive, study drug should not be administered and an early term visit should be completed.

G. Only subjects that sign the separate consent for the clinical repository and genotyping will have these samples collected. A 14-mL whole-blood sample will be collected at each of the specified visits.

H. Blood pressure, respiratory rate, heart rate, and temperature. On dosing days, vital signs should be taken pre- dose.

I. Height and Weight should be measured at screening.

J. Perform assessments prior to dilating the eyes.

K. In select countries, the MNREAD or Radner Reading Charts should be done during the study visit, prior to dilating the eyes. It should be performed monocularly first, then binocularly.

L. Microperimetry assessments will be performed post dilation. Data will be forwarded to the reading center.

M. To be administered by the masked site staff prior to any other assessments performed on that day. In-clinic assessments on the digital application should be completed after completion of all functional tests and quality of life measures prior to dilating the eyes.

N. At select sites, and for those subjects who decide to participate, the digital application will be completed on an electronic device and will consist of assessments for visual function and reading speed. Subjects will receive training at Day 1 on the use of the digital applications and the electronic device and will perform the tests using the application in the clinic at Month 1, Month 2, Month 3, Month 6, Month 12, Month 18, and Month 24. Beginning at Day 1, subjects will complete the functional assessments weekly at home. Subjects should be instructed to complete the assessments on the same day each week and at approximately the same time of day each week, if possible.

O. FAF and SD-OCT images, near infrared reflectance, fluorescein angiograms, and fundus photographs will be performed for the study eye only on days where 'SE' is specified and for both eyes at all other visits as specified in the above schedule and will be sent to the reading center for evaluation (consult the reading center manual for specifics on image capture, processing, and transmission). Images should be captured prior to dosing on dosing days. If a subject misses a study visit or images cannot be obtained at a specific visit, study staff should make every effort to obtain images at the next scheduled visit. If new active CNV is suspected, SD-OCT, FFA, and OCT-A (selected sites) images should be collected and sent to the reading center for analysis.

P. Postinjection assessments should be performed within 5 minutes after dosing by the unmasked physician or study staff and should include a gross assessment of vision (finger-counting, hand motion, then light perception when applicable). If subject passes gross vision test, the subject may leave the site. If subject fails gross vision test, the tonometry should be performed. IOP should be ≤30 mm Hg in order for the subject to leave the site. If necessary, antiglaucomatous medication can be given to lower the IOP. If IOP is >30 mm Hg, assessments will continue every approximately 30 minutes from the previous measurement until the subject passes gross vision test and IOP is ≤30 mm Hg. Note: if the study eye is treated with a ranibizumab or aflibercept injection during the same visit as the study treatment (pegcetacoplan or sham), the treatment with ranibizumab or aflibercept must be performed first. The pre- and post- anti-VEGF IOP values must be measured and recorded on the eCRF.

Q. Starting at Day 1, study subjects will be contacted by study site staff within 4 ± 2 days after each study treatment visit (through Month 4) to collect any information on any safety concerns, decrease in vision, eye pain, unusual ocular events, or any new ocular symptoms in the study eye. If the physician determines that there are any safety concerns, a follow-up visit should be scheduled as soon as possible.

R. Record concomitant medications (ie, prescription and over-the-counter medications) used by the patient within 30 days of screening and throughout the subject's participation in the study.

S. In addition to the time points indicated on the study schedule, OCT-A should be performed at the time of any suspected new active CNV. If new active CNV is confirmed in the study eye, OCT-A should be repeated every 2 months for the study eye.

T. Administration of pegcetacoplan or sham can be done on separate days from the assessment visit if both days fall within the visit window. Administration of study treatment (pegcetacoplan or sham) can be done on a separate day from the assessment visit if both days fall within the visit window. If this occurs on the randomization visit, then the administration of pegcetacoplan or sham should be done within 3 days of randomization and after approval from the medical monitor. When study treatment administration is on a day other than a study visit, then the only assessment that must be done on the day of study treatment administration is preinjection IOP.

APPENDIX D: VISIT SCHEDULE—Every-Other-Month Group—Month 13 to Month 24

	Treatment												Early Term [A]
Visit #		15		16		17		18		19		20	
Day	390	420	450	480	510	540	570	600	630	660	690	720	
Week	52	56	60	64	68	72	76	80	84	88	92	96	
Month	13	14	15	16	17	18	19	20	21	22	23	24	
Window (+ or – days)		16		16		16		16		16		16	
**Informed consent / assign screening number**													
**Demographic data**													
**Inclusion/exclusion criteria** [B]													
**Medical/surgical/ocular history** [C]													
**Blood draw—safety labs** [D,E,F]						x						x	x
**Urine sample collection** [D, E,F]						x						x	x
**Urine pregnancy test** [D,E,F]		x		x		x		x		x		x	
**Blood draw—anti-pegcetacoplan Ab** [D]		x				x						x	x
**Blood draw—genotyping (if applicable)** [D]													
**Blood draw for clinical repository (if applicable)** [D,G]												x	x
**Vital signs** [H]		x		x		x		x		x		x	x
**Physical examination** [I]												x	x
**BCVA** [J]		x		x		x		x		x		x	x
**LL-BCVA** [J]		x		x		x		x		x		x	x
**MNREAD or Radner Reading Charts (select countries)** [J,K]						x						x	x
**Mesopic microperimetry**[L]						SE						x	x
**Slitlamp examination**		x		x		x		x		x		x	x
**Endothelial cell count**[S]												x	x
**NEI VFQ-25** [M]						x						x	x
**FRI** [M]						x						x	x
**Home-based digital applications** [M,N,S]						x						x	
**Dilated indirect ophthalmoscopy**		x		x		x		x		x		x	x
**IOP measurement**		x		x		x		x		x		x	x
**SD-OCT** [O]		x		x		x		x		x		x	x
**FAF** [O]		SE		SE		x		SE		SE		x	x

	Treatment												Early Term[A]
Visit #		15		16		17		18		19		20	
Day	390	420	450	480	510	540	570	600	630	660	690	720	
Week	52	56	60	64	68	72	76	80	84	88	92	96	
Month	13	14	15	16	17	18	19	20	21	22	23	24	
Window (+ or – days)		16		16		16		16		16		16	
**NIR[O]**		SE		SE		x		SE		SE		x	x
**DCFP[O]**												x	x
**FFA[O]**												x	x
**OCT-A[S]**						x[S]						x[S]	x
**Study eye determination**													
**Randomization**													
**Pegcetacoplan administration or Sham Injection[T]**		x		x		x		x		x			
**Postinjection assessment[P]**		x		x		x		x		x			
**Follow-up call[Q]**													
**Concomitant medication/ concomitant ocular procedures[R]**		x		x		x		x		x		x	x
**Adverse events**		x		x		x		x		x		x	x

Abbreviations: BCVA=best corrected visual acuity; CNV=choroidal neovascularization; DCFP=digital color fundus photography; eCRF=electronic case report form; FAF=fundus autofluorescence; FFA=fundus fluorescein angiography; FRI=Functional Reading Independence index; IOP=intraocular pressure; LL BCVA= low luminance best corrected visual acuity; MNREAD=Minnesota Low-Vision Reading Test; NEI VFG-25=National Eye Institute Visual Functioning Questionnaire 25-item Version; NIR=near infrared reflectance; OCT-A=optical coherence tomography angiography; SD-OCT=spectral domain optical coherence tomography; SE=study eye; Term=termination; VEGF=vascular endothelial growth factor.

Note: All ocular assessments are to be performed for both eyes unless annotated with 'SE' (study eye) in the above schedule. All assessments should be performed on the same day. All study visits should be scheduled and projected based on the Day 1 visit date.

A. For subjects that discontinue the study early, the early termination assessments should be performed after a minimum of 30 days have passed from the last dosing visit. If a subject reports for a scheduled visit and decides to terminate early prior to dosing, then the visit should be considered the early termination visit and all early termination procedures should be performed. At Month 24, all subjects should be offered entry into an open-label study.

B. At Day 1 (Visit 2), confirm subject eligibility through reviewing the inclusion/ exclusion criteria and receive confirmation of eligibility from the reading center.

C. Significant medical/ surgical history from the previous 5 years. Anti-VEGF treatments (fellow eye) and invasive ocular procedures performed within the past 5 years and while on study should be recorded. Any history of tobacco use should be recorded.

D. Obtain prior to fluorescein angiography and before study drug administration.

E. At screening, serum pregnancy should be performed for women of childbearing potential. If positive, subject is not eligible to continue in the study.

F. Beginning at Day 1, perform the urine pregnancy test for women of childbearing potential at each treatment visit. If positive, perform a serum pregnancy test. If serum test is positive, study drug should not be administered and an early term visit should be completed.

G. Only subjects that sign the separate consent for the clinical repository and genotyping will have these samples collected. A 14-mL whole-blood sample will be collected at the specified time points.

H. Blood pressure, respiratory rate, heart rate, and temperature. On dosing days, vital signs should be taken predose.

I. Height and weight should be measured at screening.
J. Perform assessments prior to dilating the eyes.
K. In select countries, the MNREAD or Radner Reading Charts should be done during the study visit, prior to dilating the eyes. It should be performed monocularly first, then binocularly.
L. Microperimetry assessments will be performed post dilation. Data will be forwarded to the reading center.
M. To be administered by the masked site staff prior to any other assessments performed on that day. In-clinic assessments on the digital application should be completed after completion of all functional tests and quality of life measures prior to dilating the eyes.
N. At select sites, and for those subjects who decide to participate, the digital applications will be completed on an electronic device and will consist of assessments for visual function and reading speed. Subjects will receive training at Day 1 on the use of the digital applications and the electronic device and will perform the tests using the application in the clinic at Month 1, Month 2, Month 3, Month 6, Month 12, Month 18, and Month 24. Beginning at Day 1, subjects will complete the functional assessments weekly at home. Subjects should be instructed to complete the assessments on the same day each week and at approximately the same time of day each week, if possible.
O. FAF and SD-OCT images, near infrared reflectance, fluorescein angiograms, and fundus photographs will be performed for the study eye only on days where 'SE' is specified and for both eyes at all other visits as specified in the above schedule and will be sent to the reading center for evaluation (consult the reading center manual for specifics on image capture, processing, and transmission). Images should be captured prior to dosing on dosing days. If a subject misses a study visit or images cannot be obtained at a specific visit, study staff should make every effort to obtain images at the next scheduled visit. If new active CNV is suspected, SD-OCT, FFA, and OCT-A (selected sites) images should be collected and sent to the reading center for analysis.
P. Postinjection assessments should be performed within 5 minutes after dosing by the unmasked physician or study staff and should include a gross assessment of vision (finger-counting, hand motion, then light perception when applicable). If subject passes gross vision test, the subject may leave the site. If subject fails gross vision test, the tonometry should be performed. IOP should be ≤30 mm Hg in order for the subject to leave the site. If necessary, antiglaucomatous medication can be given to lower the IOP. If IOP is >30 mm Hg, assessments will continue every approximately 30 minutes from the previous measurement until the subject passes the gross vision test and IOP is ≤30 mm Hg. Note: if the study eye is treated with a ranibizumab or aflibercept injection during the same visit as the study treatment (pegcetacoplan or sham), the treatment with ranibizumab or aflibercept must be performed first. The pre- and post- anti-VEGF IOP values must be measured and recorded on the eCRF.
Q. Starting at Day 1, study subjects will be contacted by study site staff within 4 ± 2 days after each study treatment visit (through Month 4) to collect any information on any safety concerns, decrease in vision, eye pain, unusual ocular events, or any new ocular symptoms in the study eye. If the investigator determines that there are any safety concerns, a follow-up visit should be scheduled as soon as possible.
R. Record concomitant medications (ie, prescription and over-the-counter medications) used by the patient within 30 days of screening and throughout the subject's participation in the study.
S. In addition to the time points indicated on the study schedule, OCT-A should be performed at the time of any suspected new active CNV. If new active CNV is confirmed in the study eye, OCT-A should be repeated every 2 months for the study eye.
T. Administration of pegcetacoplan or sham can be done on separate days from the assessment visit if both days fall within the visit window. Administration of study treatment (pegcetacoplan or sham) can be done on a separate day from the assessment visit if both days fall within the visit window. If this occurs on the randomization visit, then the administration of pegcetacoplan or sham should be done within 3 days of randomization and after approval from the medical monitor. When study treatment administration is on a day other than a study visit, then the only assessment that must be done on the day of study treatment administration is preinjection IOP.

APPENDIX E: NATIONAL EYE INSTITUTE VISUAL FUNCTIONING QUESTIONNAIRE 25-ITEM VERSION

PB/IA

National Eye Institute Visual Functioning Questionnaire - 25 (VFQ-25)

version 2000

(INTERVIEWER ADMINISTERED FORMAT)

January 2000

RAND hereby grants permission to use the "National Eye Institute Visual Functioning Questionnaire 25 (VFQ-25) July 1996, in accordance with the following conditions which shall be assumed by all to have been agreed to as a consequence of accepting and using this document:

1. Changes to the NEI VFQ-25 - July 1996 may be made without the written permission of RAND. However, all such changes shall be clearly identified as having been made by the recipient.

2. The user of this NEI VFQ-25 - July 1996 accepts full responsibility, and agrees to hold RAND harmless, for the accuracy of any translations of the NEI VFQ-25 Test Version - July 1996 into another language and for any errors, omissions, misinterpretations, or consequences thereof.

3. The user of this NEI VFQ-25 - July 1996 accepts full responsibility, and agrees to hold RAND harmless, for any consequences resulting from the use of the NEI VFQ-25.

4. The user of the NEI VFQ-25 - July 1996 will provide a credit line when printing and distributing this document or in publications of results or analyses based on this instrument acknowledging that it was developed at RAND under the sponsorship of the National Eye Institute.

5. No further written permission is needed for use of this NEI VFQ-25 - July 1996.

7/29/96

© R 1996

Instructions:

I'm going to read you some statements about problems which involve your vision or feelings that you have about your vision condition. After each question I will read you a list of possible answers. Please choose the response that best describes your situation.

Please answer all the questions as if you were wearing your glasses or contact lenses (if any).

Please take as much time as you need to answer each question. All your answers are confidential. In order for this survey to improve our knowledge about vision problems and how they affect your quality of life, your answers must be as accurate as possible. Remember, if you wear glasses or contact lenses for a particular activity, please answer all of the following questions as though you were wearing them.

© R 1996

Visual Functioning Questionnaire - 25

PART 1 - GENERAL HEALTH AND VISION

1. In general, would you say your overall health is*:

(Circle One)

READ CATEGORIES:

Excellent	1
Very Good	2
Good	3
Fair	4
Poor	5

2. At the present time, would you say your eyesight using both eyes (with glasses or contact lenses, if you wear them) is excellent, good, fair, poor, or very poor or are you completely blind?

(Circle One)

READ CATEGORIES:

Excellent	1
Good	2
Fair	3
Poor	4
Very Poor	5
Completely Blind	6

*** Skip Question 1 when the VFQ-25 is administered at the same time as the SF-36 or RAND 36-Item Health Survey 1.0**

© R 1996

3. How much of the time do you worry about your eyesight?

(Circle One)

READ CATEGORIES:

None of the time	1
A little of the time	2
Some of the time	3
Most of the time	4
All of the time?	5

4. How much pain or discomfort have you had in and around your eyes (for example, burning, itching, or aching)? Would you say it is:

(Circle One)

READ CATEGORIES:

None	1
Mild	2
Moderate	3
Severe, or	4
Very severe?	5

PART 2 - DIFFICULTY WITH ACTIVITIES

The next questions are about how much difficulty, if any, you have doing certain activities wearing your glasses or contact lenses if you use them for that activity.

5. How much difficulty do you have reading ordinary print in newspapers? Would you say you have:
(READ CATEGORIES AS NEEDED)

(Circle One)

No difficulty at all	1
A little difficulty	2
Moderate difficulty	3
Extreme difficulty	4
Stopped doing this because of your eyesight	5
Stopped doing this for other reasons or not interested in doing this	6

© R 1996

6. How much difficulty do you have doing work or hobbies that require you to <u>see well up close</u>, such as cooking, sewing, fixing things around the house, or using hand tools? Would you say:
(READ CATEGORIES AS NEEDED)

(Circle One)

No difficulty at all.. 1
A little difficulty.. 2
Moderate difficulty... 3
Extreme difficulty.. 4
Stopped doing this because of your eyesight.... 5
Stopped doing this for other reasons or not interested in doing this 6

7. Because of your eyesight, how much difficulty do you have <u>finding something on a crowded shelf</u>?
(READ CATEGORIES AS NEEDED)

(Circle One)

No difficulty at all.. 1
A little difficulty.. 2
Moderate difficulty... 3
Extreme difficulty.. 4
Stopped doing this because of your eyesight.... 5
Stopped doing this for other reasons or not interested in doing this 6

8. How much difficulty do you have <u>reading street signs or the names of stores</u>?
(READ CATEGORIES AS NEEDED)

(Circle One)

No difficulty at all.. 1
A little difficulty.. 2
Moderate difficulty... 3
Extreme difficulty.. 4
Stopped doing this because of your eyesight.... 5
Stopped doing this for other reasons or not interested in doing this 6

© R 1996

9. **Because of your eyesight, how much difficulty do you have <u>going down steps, stairs, or curbs in dim light or at night</u>?**
(READ CATEGORIES AS NEEDED)

(Circle One)

No difficulty at all.. 1
A little difficulty... 2
Moderate difficulty.. 3
Extreme difficulty... 4
Stopped doing this because of your eyesight.... 5
Stopped doing this for other reasons or not interested in doing this 6

10. **Because of your eyesight, how much difficulty do you have <u>noticing objects off to the side while you are walking along</u>?**
(READ CATEGORIES AS NEEDED)

(Circle One)

No difficulty at all.. 1
A little difficulty... 2
Moderate difficulty.. 3
Extreme difficulty... 4
Stopped doing this because of your eyesight.... 5
Stopped doing this for other reasons or not interested in doing this 6

11. **Because of your eyesight, how much difficulty do you have <u>seeing how people react to things</u> you say?**
(READ CATEGORIES AS NEEDED)

(Circle One)

No difficulty at all.. 1
A little difficulty... 2
Moderate difficulty.. 3
Extreme difficulty... 4
Stopped doing this because of your eyesight.... 5
Stopped doing this for other reasons or not interested in doing this 6

© R 1996

12. **Because of your eyesight, how much difficulty do you have <u>picking out and matching your own clothes</u>?**
(READ CATEGORIES AS NEEDED)

(Circle One)

**No difficulty at all**	**1**
**A little difficulty**	**2**
**Moderate difficulty**	**3**
**Extreme difficulty**	**4**
**Stopped doing this because of your eyesight**	**5**
**Stopped doing this for other reasons or not interested in doing this**	**6**

13. **Because of your eyesight, how much difficulty do you have <u>visiting with people in their homes, at parties, or in restaurants</u> ?**
(READ CATEGORIES AS NEEDED)

(Circle One)

**No difficulty at all**	**1**
**A little difficulty**	**2**
**Moderate difficulty**	**3**
**Extreme difficulty**	**4**
**Stopped doing this because of your eyesight**	**5**
**Stopped doing this for other reasons or not interested in doing this**	**6**

14. **Because of your eyesight, how much difficulty do you have <u>going out to see movies, plays, or sports events</u>?**
(READ CATEGORIES AS NEEDED)

(Circle One)

**No difficulty at all**	**1**
**A little difficulty**	**2**
**Moderate difficulty**	**3**
**Extreme difficulty**	**4**
**Stopped doing this because of your eyesight**	**5**
**Stopped doing this for other reasons or not interested in doing this**	**6**

© R 1996

15. Now, I'd like to ask about driving a car. Are you currently driving, at least once in a while?

(Circle One)

Yes .... 1 *Skip To Q 15c*

No 2

15a. IF NO, ASK: Have you never driven a car or have you given up driving?

(Circle One)

Never drove ...... 1 *Skip To Part 3, Q 17*

Gave up............ 2

15b. IF GAVE UP DRIVING: Was that mainly because of your eyesight, mainly for some other reason, or because of both your eyesight and other reasons?

(Circle One)

Mainly eyesight .... 1 *Skip To Part 3, Q 17*

Mainly other reasons .... 2 *Skip To Part 3, Q 17*

Both eyesight and other reasons ... 3 *Skip To Part 3, Q 17*

15c. IF CURRENTLY DRIVING: How much difficulty do you have driving during the daytime in familiar places? Would you say you have:

(Circle One)

No difficulty at all 1
A little difficulty 2
Moderate difficulty 3
Extreme difficulty 4

© R 1996

16. How much difficulty do you have driving at night? Would you say you have: (READ CATEGORIES AS NEEDED)

(Circle One)

**No difficulty at all**	**1**
**A little difficulty**	**2**
**Moderate difficulty**	**3**
**Extreme difficulty**	**4**
**Have you stopped doing this because of your eyesight**	**5**
**Have you stopped doing this for other reasons or are you not interested in doing this**	**6**

16a. How much difficulty do you have driving in difficult conditions, such as in bad weather, during rush hour, on the freeway, or in city traffic? Would you say you have:
(READ CATEGORIES AS NEEDED)

(Circle One)

**No difficulty at all**	**1**
**A little difficulty**	**2**
**Moderate difficulty**	**3**
**Extreme difficulty**	**4**
**Have you stopped doing this because of your eyesight**	**5**
**Have you stopped doing this for other reasons or are you not interested in doing this**	**6**

© R 1996

PART 3: RESPONSES TO VISION PROBLEMS

The next questions are about how things you do may be affected by your vision. For each one, I'd like you to tell me if this is true for you <u>all</u>, <u>most</u>, <u>some</u>, <u>a little</u>, or <u>none</u> of the time.

(Circle One On Each Line)

READ CATEGORIES:	All of the time	Most of the time	Some of the time	A little of the time	None of the time
17. Do you accomplish less than you would like because of your vision?	1	2	3	4	5
18. Are you limited in how long you can work or do other activities because of your vision?	1	2	3	4	5
19. How much does pain or discomfort in or around your eyes, for example, burning, itching, or aching, keep you from doing what you'd like to be doing? Would you say:	1	2	3	4	5

© R 1996

For each of the following statements, please tell me if it is definitely true, mostly true, mostly false, or definitely false for you or you are not sure.

(Circle One On Each Line)

	Definitely True	Mostly True	Not Sure	Mostly False	Definitely False
20. I stay home most of the time because of my eyesight.....	1	2	3	4	5
21. I feel frustrated a lot of the time because of my eyesight..............................	1	2	3	4	5
22. I have much less control over what I do, because of my eyesight.	1	2	3	4	5
23. Because of my eyesight, I have to rely too much on what other people tell me..	1	2	3	4	5
24. I need a lot of help from others because of my eyesight..............................	1	2	3	4	5
25. I worry about doing things that will embarrass myself or others, because of my eyesight..............................	1	2	3	4	5

That's the end of the interview. Thank you very much for your time and your help.

© R 1996

Appendix of Optional Additional Questions

SUBSCALE: GENERAL HEALTH

A1. How would you rate your overall health, on a scale where zero is as bad as death and 10 is best possible health?

(Circle One)

0	1	2	3	4	5	6	7	8	9	10
Worst										Best

SUBSCALE: GENERAL VISION

A2. How would you rate your eyesight now (with glasses or contact lens on, if you wear them), on a scale of from 0 to 10, where zero means the worst possible eyesight, as bad or worse than being blind, and 10 means the best possible eyesight?

(Circle One)

0	1	2	3	4	5	6	7	8	9	10
Worst										Best

SUBSCALE: NEAR VISION

A3. Wearing glasses, how much difficulty do you have reading the small print in a telephone book, on a medicine bottle, or on legal forms? Would you say:
(READ CATEGORIES AS NEEDED)

(Circle One)

**No difficulty at all**	**1**
**A little difficulty**	**2**
**Moderate difficulty**	**3**
**Extreme difficulty**	**4**
**Stopped doing this because of your eyesight**	**5**
**Stopped doing this for other reasons or not interested in doing this**	**6**

© R 1996

A4. Because of your eyesight, how much difficulty do you have figuring out whether bills you receive are accurate?
(READ CATEGORIES AS NEEDED)

(Circle One)

**No difficulty at all**	**1**
**A little difficulty**	**2**
**Moderate difficulty**	**3**
**Extreme difficulty**	**4**
**Stopped doing this because of your eyesight**	**5**
**Stopped doing this for other reasons or not interested in doing this**	**6**

A5. Because of your eyesight, how much difficulty do you have doing things like shaving, styling your hair, or putting on makeup?
(READ CATEGORIES AS NEEDED)

(Circle One)

**No difficulty at all**	**1**
**A little difficulty**	**2**
**Moderate difficulty**	**3**
**Extreme difficulty**	**4**
**Stopped doing this because of your eyesight**	**5**
**Stopped doing this for other reasons or not interested in doing this**	**6**

SUBSCALE: DISTANCE VISION

A6. Because of your eyesight, how much difficulty do you have recognizing people you know from across a room?
(READ CATEGORIES AS NEEDED)

(Circle One)

**No difficulty at all**	**1**
**A little difficulty**	**2**
**Moderate difficulty**	**3**
**Extreme difficulty**	**4**
**Stopped doing this because of your eyesight**	**5**
**Stopped doing this for other reasons or not interested in doing this**	**6**

© R 1996

A7. Because of your eyesight, how much difficulty do you have taking part in active sports or other outdoor activities that you enjoy (like golf, bowling, jogging, or walking)?
(READ CATEGORIES AS NEEDED)

	(Circle One)
**No difficulty at all**	**1**
**A little difficulty**	**2**
**Moderate difficulty**	**3**
**Extreme difficulty**	**4**
**Stopped doing this because of your eyesight**	**5**
**Stopped doing this for other reasons or not interested in doing this**	**6**

A8. Because of your eyesight, how much difficulty do you have seeing and enjoying programs on TV?
(READ CATEGORIES AS NEEDED)

	(Circle One)
**No difficulty at all**	**1**
**A little difficulty**	**2**
**Moderate difficulty**	**3**
**Extreme difficulty**	**4**
**Stopped doing this because of your eyesight**	**5**
**Stopped doing this for other reasons or not interested in doing this**	**6**

SUBSCALE: SOCIAL FUNCTION

A9. Because of your eyesight, how much difficulty do you have entertaining friends and family in your home?
(READ CATEGORIES AS NEEDED)

	(Circle One)
**No difficulty at all**	**1**
**A little difficulty**	**2**
**Moderate difficulty**	**3**
**Extreme difficulty**	**4**
**Stopped doing this because of your eyesight**	**5**
**Stopped doing this for other reasons or not interested in doing this**	**6**

© R 1996

SUBSCALE: DRIVING

A10. [This items, "driving in difficult conditions", has been included as item 16a as part of the base set of 25 vision-targeted items.]

SUBSCALE: ROLE LIMITATIONS

A11. The next questions are about things you may do because of your vision. For each item, I'd like you to tell me if this is true for you all, most, some, a little, or none of the time.
(READ CATEGORIES AS NEEDED)

(Circle One On Each Line)

	All of the time	Most of the time	Some of the time	A little of the time	None of the time
a. Do you have more help from others because of your vision?	1	2	3	4	5
b. Are you limited in the kinds of things you can do because of your vision?	1	2	3	4	5

© R 1996

SUBSCALES: WELL-BEING/DISTRESS (#A12) and DEPENDENCY (#A13)

The next questions are about how you deal with your vision. For each statement, please tell me if it is definitely true, mostly true, mostly false, or definitely false for you or you don't know.

(Circle One On Each Line)

	Definitely True	Mostly True	Not Sure	Mostly False	Definitely False
A12. I am often irritable because of my eyesight..................	1	2	3	4	5
A13. I don't go out of my home alone, because of my eyesight..............................	1	2	3	4	5

© R 1996

APPENDIX F: FUNCTIONAL READING INDEPENDENCE INDEX (FRI)

Kimel M, Yu R, Leidy N. The Functional Reading Independence Index (FRI Index) - User Manual (Version 1.2). Evidera. 2015

CCI

[redacted]

CCI

[redacted]

CCI

[redacted]

CCI

[redacted]

CCI

[redacted]

CCI

[redacted]

CCI

[redacted]

CCI

[redacted]

CCI

[redacted]

CCI

[redacted]

CCI

[redacted]

CCI

[redacted]

CCI

[redacted]

CCI

[redacted]

CCI

[redacted]

CCI

[redacted]

CCI

[redacted]

CCI

[redacted]

CCI

[redacted]

CCI

[redacted]

CCI

[redacted]

CCI

[redacted]

CCI

[redacted]

CCI

[redacted]

CCI

[redacted]

CCI

[redacted]

CCI

[redacted]

CCI

[redacted]

CCI

[redacted]

CCI

[redacted]

CCI

[redacted]

CCI

[redacted]

APPENDIX G: MINNESOTA LOW-VISION READING TEST (MNREAD) AND RADNER READING CARDS

The manual of procedures should be referenced for a detailed table of countries and languages to determine which test will be administered. Equipment and testing procedures will also be described in detail in the manual of procedures for both tests.

Minnesota Low-Vision Reading Test:

The Minnesota Low-Vision Reading Test acuity cards are continuous-text reading-acuity cards used for measuring the reading acuity and speed of normal and low-vision patients. These charts were developed at the Minnesota Laboratory for Low-Vision Research, University of Minnesota, Minneapolis, Minnesota, USA.

The MNREAD Acuity Charts will be used to measure:

- Critical Print Size: the smallest print that the patient can read with maximum speed
- Maximum Reading Speed: the patient’s reading speed when reading is not limited by print size

Radner Reading Cards:

The Radner Reading Cards consist of sentence optotypes, which are optimized reading test items, standardized by construction and statistical selection. These cards allow for accurate and comparable measurements of reading acuity and reading speed and measurement of critical print size.

APPENDIX H: GRADING SCALE FOR ASSESSMENT OF ANTERIOR CHAMBER FLARE OR CELLS

AQUEOUS REACTION—FLARE

Grade	Description
0	No protein is visible in the anterior chamber when viewed by an experienced observer using slitlamp biomicroscopy; a small, bright, focal slit-beam of white light; and high magnification.
Trace	Trace amount of protein detectable in the anterior chamber. This protein is visible only with careful scrutiny by an experienced observer using slitlamp biomicroscopy; a small, bright, focal slit-beam of white light; and high magnification.
1+	Mild amount of protein detectable in the anterior chamber. This protein is immediately apparent to an experienced observer using slitlamp biomicroscopy and high magnification, but such protein is detected only with careful observation with the naked eye and a small, bright, focal slit-beam of white light. Note presence of any fibrin.
2-3+	Moderate amount of protein detectable in the anterior chamber. These grades are similar to 1+ but the opacity would be readily visible to the naked eye of an observer using any source of a focused beam of white light. This is a continuum of moderate opacification, with 2+ being less apparent than 3+. Note presence of any fibrin.
4+	A large (severe) amount of protein is detectable in the anterior chamber. Similar to 3+, but the density of the protein approaches that of the lens. Additionally, frank fibrin deposition is frequently seen in acute circumstances. It needs to be noted that because fibrin may persist for a period of time after partial or complete restoration of the blood-aqueous barrier, it is possible to have resorbing fibrin present with lower numeric assignations for flare (eg, 1+ flare with fibrin).

AQUEOUS REACTION—CELLS

Grade	Description
0	No cells are seen in any optical section when a large slitlamp beam is swept across the anterior chamber.
Trace	Rare (1-3) cells are observed when the slitlamp beam is swept across the anterior chamber. When the instrument is held stationary, not every optical section contains circulating cells.
1+	3-10 cells/optical section are seen when the slitlamp beam is swept across the anterior chamber. When the instrument is held stationary, every optical section contains circulating cells. Note stage of hypopyon, if applicable.
2+	10-25 cells are seen when the slitlamp beam is swept across the anterior chamber. When the instrument is held stationary, every optical section contains circulating cells. Note stage of hypopyon, if applicable.
3+	25-50 cells are seen when the slitlamp beam is swept across the anterior chamber. When the instrument is held stationary, every optical section contains circulating cells. Keratic precipitates or cellular deposits on the anterior lens capsule may be present. Note stage of hypopyon, if applicable.
4+	More than 50 cells are seen when the slitlamp beam is swept across the anterior chamber. When the instrument is held stationary, every optical section contains circulating cells or

	hypopyon is noted. As for the fibrin deposition, hypopyon may persist for a period of time after exudation of cells into the anterior chamber has diminished or ceased entirely, making it possible for have 1+ circulating cells in the anterior chamber with a resolving hypopyon.

APPENDIX I: GRADING SCALE FOR ASSESSMENT OF VITREOUS CELLS

Grade	Description	Cells in Retro-Illuminated Field
0	Clear	0
Trace	Few opacities	1-20
1	Scattered opacities	21-50
2	Moderate opacities	51-100
3	Many opacities	101-250
4	Dense opacities	≥ 251

APPENDIX J: AMENDMENT HISTORY

A summary of changes from previous amendments is provided below.

Protocol Versions		
**Summary of Change(s) Since Last Version of Approved Protocol**		
**Amendment 4**	**Amendment Date<br>27 April 2020**	**Global**
**Description of Change**		**Section(s) Affected by Change**
Non-substantial changes that did not impact content of the document have been made for clarity.		Entire document
Information related to COVID-19-related changes added		Appendix K

Protocol Versions		
**Summary of Change(s) Since Last Version of Approved Protocol**		
**Amendment 3<br>Version 1.0**	**Amendment Date<br>14 February 2019**	**Global**
**Description of Change**		**Section(s) Affected by Change**
Non-substantial changes that did not impact content of the document have been made for clarity.		Entire document
PK and complement profile assessments have been removed from this study.		Entire document
It was noted that:<br><u>In addition, two GLP-compliant 2-month ocular bridging studies (the first comparing drug substance from two different CMOs, the second assessing the safety and tolerability profiles of three different formulations to support the Phase 3 clinical study) have been conducted, in cynomolgus monkeys as well.</u><br>The previous version of the protocol noted that 1 2-month ocular bridging study was completed and that data from a second study would be performed to assess the safety and tolerability profiles of three different formulations in order to support the Phase 3 formulation.		Section 4.1.3 Nonclinical Data
Updated to include Clinical Study APL2-103.		Section 4.1.4 Clinical Data
Updated to include the following statement:<br><u>In recent studies conducted with APL 2 IVT from a single manufacturer, events of transient moderate and severe intraocular inflammation have been observed.</u>		Section 4.2 Risk/Benefit

Section revised to further define approved methods of contraception include:<br>• Combined (estrogen-and progestogen-containing) hormonal contraception associated with inhibition of ovulation:<br>o Oral<br>o Intravaginal<br>o Transdermal<br>• Progestogen-only hormonal contraception associated with inhibition of ovulation:<br>o Oral<br>o Injectable<br>o Implantable<br>• Intrauterine device (IUD)<br>• Intrauterine hormone-releasing system (IUS)<br>• Bilateral tubal occlusion<br>• Vasectomized partner (provided that partner is the sole sexual partner of the WOCBP trial participant and that the vasectomized partner has received medical assessment of the surgical success)<br>• Sexual abstinence (defined as refraining from heterosexual intercourse during the entire period of risk associated with the study treatments). Sexual abstinence is only accepted when it is the preferred and usual lifestyle of the subject.<br><br>~~Approved methods of contraception include: oral contraceptives, intrauterine device, medically acceptable barrier methods (diaphragm or condom), implantable or injectable contraceptives (like DepoProvera) or removable birth control device (like NuvaRing or Ortho Evra patches); and/or surgical sterilization (at least 6 months before dosing). Subjects practicing abstinence and coitus interruptus (pull out method) must agree to use an approved method of contraception during the study and 90 days after their last dose of study drug.~~	Section 6.4 Approved Methods of Contraception
The following section was added:<br><br>A subject may withdraw from the study at any time for any reason without prejudice to his/her future medical care by the physician or at the institution. The investigator or sponsor may withdraw the subject at any time (e.g., in the interest of subject safety). The investigator is encouraged to discuss withdrawal of a subject from treatment with the investigational product with the medical monitor when possible. Subjects who discontinue treatment with the investigational product can continue participation in the study and should be encouraged to return to the clinical site for as many follow-up visits as they can. In the event that a subject terminates early from the study, all early termination procedures should be performed even if they are outside the allowed study window.<br>The reason for termination, date of stopping treatment with investigational product, all follow-up information and the total amount of investigational product administered must be recorded in the case report form (CRF) and source documents.	Section 6.5 Discontinuation of Subjects

The requirement to notify the sponsor prior to unmasking was removed:<br><br>In the event of a medical emergency where the knowledge of subject treatment by masked individuals (e.g., the subject or his/her physician) is required, an individual Investigator (or designee) will have the ability to unmask the treatment assignment for a specific subject and share that information with the appropriate parties. ~~The Investigator (or designee) must endeavor to notify the Sponsor prior to unmasking a subject.~~	Section 7.2.1 Unmasking
Section was revised to allow study treatment administration to occur on a day separate from the assessment visit:<br><br>Section was revised to allow study treatment administration to occur on a day separate from the assessment visit:<br><br>Only qualified study staff and those delegated the responsibility of study drug administration on the Delegation of Authority log should perform this procedure. All staff should be appropriately trained on all procedures prior to performing the procedures. Sites should follow the Visit Schedule for order of procedures and assessments.<br><br>Administration of study treatment (APL-2 or Sham) can be done on a separate day from the assessment visit if both days fall within the visit window. If this occurs on the randomization visit, then the administration of APL-2 or Sham should be done within 3 days of randomization and after approval from the medical monitor. When study treatment administration is on a day other than a study visit, then the only assessment that must be done on the day of study treatment administration is the pre-injection IOP.<br><br>If a subject falls outside the visit window for a dosing visit, the dose should be skipped and the subject should be scheduled on time for the next dosing visit.	Section 7.3.2 Treatment Administration
The description of sham injection was modified as there is no requirement for sham to be sterile:<br><br>Sham will be provided as ~~sterile~~ empty stoppered glass vials and should be stored according to the label.	Section 7.3.3.1 Identity of the Investigational Product
The procedure for sham injection was further described:<br><br>The procedure for sham injection will be the same as that used for IVT injection until the actual injection but no actual injection will occur. The injecting physician will only touch the study eye with the blunt end of the syringe. No needle or medication will be injected inside the eye.	Section 7.4 Sham Injection Administration

Detailed instructions on sham injection procedures and post-injection procedures will be provided in the Manual of Procedures.	
The end of the trial for each subject was defined:<br><br>The end of the trial for each subject is defined as when the subject either completes their Month 24 Visit and enrolls in the open-label extension study or, should a subject elect not to enter the open label extension study, when the subject completes their exit visit at Month 30.<br><br>Through the section it has also been noted that endothelial cell count assessment is for "select sites only". This was previously noted in the schedule of events but not consistently in the body of the protocol. In addition FAF and NAR are no longer "study eye only".	Section 8 Study Procedures
The following assessments were removed as they will no longer be conducted at the screening Visit 1:<br>• ~~Low luminance BCVA~~<br>• ~~Endothelial cell count~~<br>• ~~OCT-A~~<br>The following text was moved to Section 8.1.2 as training for home-based digital applications will now be conducted at Visit 2:<br><br>~~Prior to dilating the eyes, subjects at select sites will be trained on how to use the home-based digital applications for visual function and reading speed if the subject decides to participate in this portion. This training must occur after completion of all functional tests (NL-BCVA, LL-BCVA).~~	Section 8.1.1 Visit 1 All Subjects
It was clarified that:<br>All assessments should be performed on the same day. All study visits should be scheduled and projected based on the Day 1 visit.<br><br>The following text was added as home-based digital application training will now occur at Visit 2:<br>Subjects at select sites will be trained on how to use the home-based digital applications for visual function and reading speed if the subject decides to participate in this portion. This training must occur after completion of all functional tests (NL-BCVA, LL-BCVA).<br><br>The following assessments were added:<br>• Endothelial cell count<br>• OCT-A (select sites)<br><br>The following text was removed as subject discontinuation is now covered in Section 6.5.<br>~~In the event that a subject is early terminated from the study, all early termination procedures should be performed even if they are outside the allowed study window.~~<br>~~If the subject would like to discontinue dosing but is amenable to~~	Section 8.1.2 Randomization/Initial Treatment—Day—Within 28 Days of Screening

~~continuing in the study, the site should make every effort to have the subject complete as many follow-up visits as possible.~~	
It has been noted in the text which assessments will be conducted at Month 27 and Month 30.	Section 8.1.4 Follow-up Phase
Instructions were revised to include the following:<br><br>All AEs encountered during the study will be monitored and reported in detail in the source documents and documented on the eCRF, from signing of the ICF until the Exit Visit. AEs should be recorded by maximum severity. AEs, especially those for which the relationship to ~~test drug~~ study treatment are considered by the Investigator to be possibly or definitely related, should be followed up until they have returned to the baseline status or stabilized. If a clear explanation is established, it should be recorded on the eCRF.	Section 11.2 Recording Adverse Events
Instructions modified as follows:<br>~~If any AEs are serious, special procedures will be followed. All SAEs will be reported to the Safety Monitor by the Investigator via fax or email within one calendar day of becoming aware of the event, whether or not the serious events are deemed drug-related. SAE reporting contact information will be provided separately and as included in the Safety Monitoring Plan. All SAEs must be reported to the applicable ethics committee by the Investigator in accordance with their regulations.~~<br><br>**Special procedures will be followed for reporting SAEs.** All SAEs will be reported to the Safety Monitor by the Investigator via eCRF or fax/email (if eCRF is not available) within **24 hours** of becoming aware of the event, whether or not the event is deemed treatment-related. If the EDC system is not operational, the site must complete the appropriate paper SAE form and fax/email to the number listed on the SAE form, also within 24 hours of becoming aware of the event. The reported information submitted as a paper SAE form must be entered into the EDC system once it becomes operational.<br><br>SAE reporting contact information will be provided separately and included in the Safety Monitoring Plan. All SAEs must be reported to the applicable ethics committee by the Investigator in accordance with their regulations.	Section 11.5 Serious Adverse Events
The following text was added:<br>A suspected adverse reaction means any AE for which there is a reasonable possibility that the drug caused the AE. Reasonable possibility means there is evidence (such as a temporal relationship) to suggest a causal relationship between the drug and the AE.	Section 11.6 Unexpected Adverse Events or Unexpected Suspected Adverse Reactions
The analysis description was modified as follows as this will be conducted at all sites. This is an error correction.<br>• Change from baseline at each planned assessment in the total area of GA lesion(s) in the study eye (in mm2) as assessed by FAF ~~(in select sites)~~	Section 12.7.2 Secondary Efficacy Analysis

Section 12.10 was added to describe how visit windows will be used in the assessment of the primary endpoint:<br><br>Analysis visits will be derived with windows for the monthly visits to assess the primary endpoint. Baseline is defined as the date of randomization. If 2 or more treatment visits occur within a window, the closest visit to the target day will be used as that analysis visit; if 2 visits are equidistant from the scheduled analysis visit day, the later analysis visit will be used.	Section 12.10 Visit Windows
All study schedules were modified to align with the changes noted above, including removal of PK and complement profile study assessments.	Appendix A, B, C, and D: Visit Schedules

<table>
<tr><th colspan="3">Protocol Versions</th></tr>
<tr><th colspan="3">Summary of Change(s) Since Last Version of Approved Protocol</th></tr>
<tr><th>Amendment 2
Version 1.0</th><th>Version Date
2018</th><th>Global</th></tr>
<tr><th colspan="2">Description of Change</th><th>Section(s) Affected by Change</th></tr>
<tr><td colspan="2">Non-substantial changes that did not impact content of the document have been made for clarity and to align with language used in the protocol for Study APL2-303.</td><td>Entire document</td></tr>
<tr><td colspan="2">Exclusion criteria #4 and #10 were modified as follows for clarity and to exclude subjects with history of IVT injection in either eye:
4. Presence ~~in either eye~~ of an active ocular disease that in the opinion of the Investigator compromises or confounds visual function, including but not limited to, uveitis, other macular diseases (e.g., clinically significant epiretinal membrane [ERM], full thickness macular hole or uncontrolled glaucoma/ocular hypertension). Benign conditions in the opinion of the investigator such as peripheral retina dystrophy are not exclusionary.

10. History of prior intravitreal injection ~~in the study eye~~.
Exclusion criteria #14 was modified as follows for clarity:

14. Participation in any systemic experimental treatment or any other systemic investigational new drug including within 6 weeks or 5 half-lives of the active ingredient (whichever is longer) prior to the start of study treatment. Note: clinical trials solely involving observation, over-the-counter vitamins, supplements, or diets are not exclusionary.</td><td>Synopsis
Section 6.1.2 Exclusion Criteria</td></tr>
<tr><td colspan="2">The following exclusion criteria was implemented as Exclusion Criteria #13 (EC criteria following this were renumbered):

13. Prior participation in another interventional clinical study for geographic atrophy in either eye including investigational oral medication and placebo.</td><td>Synopsis
Section 6.1.2 Exclusion Criteria</td></tr>
<tr><td colspan="2">Noted that the NEI VFQ-25 distance activity and near activity subscale score endpoints will be conducted in select countries as these subscales are not available in all languages.</td><td>Synopsis
Section 5.1.3 Secondary Objectives
Section 5.1.5 Exploratory Objectives
Section 12.7.2 Secondary Efficacy Analysis
Section 12.7.3 Exploratory Efficacy Analysis
Section 9.9.1 The National Eye Institute Visual Functioning Questionnaire 25-Item Version (NEI VFQ)</td></tr>
<tr><td colspan="2">Contraception text was updated to align with the Investigator's Brochure and in studies with APL-2 in other indications:
Approved methods of contraception include: ~~hormonal contraceptives~~</td><td>Section 6.4 Approved Methods of Contraception</td></tr>
</table>

~~associated with inhibition of ovulation,~~ oral contraceptives, intrauterine device, ~~intrauterine hormone-releasing system; and/or bilateral tubal occlusion (at least 6 months before dosing)~~ medically acceptable barrier methods (diaphragm or condom), implantable or injectable contraceptives (like DepoProvera) or removable birth control device (like NuvaRing or Ortho Evra patches); and/or surgical sterilization (at least 6 months before dosing). Subjects practicing abstinence and coitus interruptus (pull out method) must agree to use an approved method of contraception during the study and ~~60~~ 90 days after their last dose of study drug.	
Masking language was modified to indicate that the PI is required to remain masked to subjects' treatment assignments, while the treating physician and any associated support staff involved in IP administration will be unmasked.	Section 7.2 Masking and Minimization of Bias
Guidance on the treatment of new exudation related to active choroidal neovascularization in the study eye and/or fellow eye was modified to clarify that the reading center will provide a report to indicate whether or not there is evidence of active, exudative AMD, and the Investigator will then determine if anti-VEGF treatment should be initiated. The Investigator should wait for the reading center report before making a decision regarding treatment, except in cases where there is clear evidence of disease activity that may have a detrimental visual impact if not treated immediately.<br><br>It was also clarified that either ranibizumab or aflibercept should be selected as the anti-VEGF therapy, and that the unmasked physician should administer anti-VEGF treatment if the treatment is administered on the same day as APL-2.<br><br>It was also noted that any treatments of therapies administered to the fellow eye within 5 years of screening should be recorded as a concomitant medication.	Section 7.5.1 Treatment of New Exudation Related to Active Choroidal Neovascularization in the Study Eye and/or Fellow Eye<br><br>Visit Schedules A-D
The following instruction regarding electronic devices was added:<br><br>The subject will be instructed to bring back the electronic device for the visits specified in the schedule of events.	Section 8.1.3.1 Months 1-12
Month 6 and Month 12 were added as times of in-clinic assessment.<br><br>It was noted that subjects will take electronic devices home at Day 1 instead of at Screening.	Section 9.12 Home-Based Functional Digital Applications (in select sites/countries)<br><br>Visit Schedules A-D.
Clarified that:<br>In the event that a subject is suspected to have new active CNV in the study eye and/or the fellow eye, an SD-OCT and FA using the protocol specified procedures should be performed and sent to the Reading Center to confirm the diagnosis. In addition, in selected sites, OCT-A should also be captured according to the study imaging protocol and sent to the Reading Center.	Section 9.14 Ocular Imaging
It was clarified that if the subject does not pass the gross vision test, IOP must be measured at that time. Additional IOP measurement must be taken approximately every 30 minutes thereafter until IOP ≤ 30 mm Hg	Section 9.15 Post-Injection Assessment; Visit Schedule Footnote "O" (all Visit Schedules)

and the subject is able to be released from the clinic.<br><br>It was clarified that anti-VEGF treatment will be either ranibizumab or aflibercept, and that IOP should be measured before and after the anti-VEGF injection, but prior to administration of APL-2.	
Blood volume for study assessments was updated based on the lab manual.	Section 9.16 Blood Volume for Study Assessments
It was clarified that ~~15~~14 mL whole blood sample will be collected at the specified time points for research samples.	Section 9.17.2 Sample Collection<br><br>Visit Schedules A-D
The screening urine pregnancy test was removed as a serum pregnancy test will be performed for screening.<br><br>Footnotes "M" (All Visit Schedules) was modified to indicate that "Beginning at Day 1 Screening, subjects will complete the functional assessments weekly at home."	Visit Schedules A and C
Week 24 blood draw for clinical repository was added (error correction).	Visit Schedule C
The full version of the NEI VFQ-25 has been included in Appendix E.	Appendix E.
An amendment history appendix was added to show changes enacted by previous amendments.	Appendix J

Amendment 1 Version 1.0 / Version Date 31 May 2018	Sections Affected by Change
Non-substantial changes that did not impact content of the document have been made for clarity and to align with language used in the protocol for Study APL2-303. In addition, section numbering was modified to unify document structure between this protocol and the protocol for Study APL2-303.	Entire document
The changes noted below were also incorporated into the synopsis where applicable.	Synopsis
Study objectives were expanded to describe the endpoints that will be utilized to meet them. Endpoint descriptions were moved to Section 12: DATA MANAGEMENT AND STATISTICAL CONSIDERATIONS.	Section 5. STUDY OBJECTIVES ~~AND ENDPOINTS~~<br>Section 12. DATA MANAGEMENT AND STATISTICAL CONSIDERATIONS
It was noted that exploratory objectives utilizing digital applications will be conducted in select sites/countries only as these applications will be utilized according to local regulation.	Section 5.1.3 Exploratory Objectives<br>Section 8 STUDY PROCEDURES<br>Section 9.13 Home-Based Digital Applications (in select sites/countries)
It was clarified that to participate in the study, subjects must be diagnosed with GA of the macula secondary to AMD in ~~one or both eyes~~ the study eye.	Section 6. Patient Population
It was noted that if both eyes have the same visual acuity score, the right eye will be selected as the study eye.<br><br>The inclusion criteria for microperimetry was incorporated to the main list of inclusion criteria (as #6) as all patients in this study will now be participating in this portion.	Section 6.1.1 Inclusion Criteria
The exclusion criteria for microperimetry was moved to the main list of exclusion criteria (as #11) as all patients in this study will now be participating in this portion.<br><br>Exclusion criteria 11 was updated as follows:<br>~~11~~12. Prior participation in another interventional clinical study for intravitreal therapies in either eye (including subjects receiving sham).	Section 6.1.2 Exclusion Criteria
Contraception requirements were updated as follows based on requirements from ethics committees:<br>Approved methods of contraception include: ~~oral~~ hormonal contraceptives associated with inhibition of ovulation, intrauterine device, ~~medically acceptable barrier methods (diaphragm or condom), implantable or injectable contraceptives or removable birth control device~~ intrauterine hormone-releasing system; and/or ~~sterilization~~ bilateral tubal occlusion (at least 6 months before dosing). Subjects practicing abstinence and coitus interruptus (pull out method) must agree to use an approved method of contraception during the study and 60 days after their last dose of study drug.	Section 6.4 Approved Methods of Contraception

"Endothelial cell count" was removed as an assessment that will be provided to the reading center for objective assessment of subject eligibility.<br><br>"Optical Coherence Tomography Angiography (OCT-A, selected sites only)" was added as an assessment that will be provided to the reading center for objective assessment of subject eligibility.	Section 7.1 Allocation to Treatment<br>Section 8.1. Study Visit Schedule<br>Section 8.1.2 Randomization/Initial Treatment
"Country" and "microperimetry eligibility (yes; no)" were removed as a stratification factor.	Section 7.1 Allocation to Treatment
References to "DSMB" updated to "DMC".	Section 7.2.1 Unmasking<br>Section 10.1 Data Monitoring Committee
Reference to preparing APL-2 for injection was removed from the paragraph describing sham.	Section 7.3.3.1 Identity of the Investigational Product
Text was updated to indicate that:<br><br>The suspected onset or presence of new active choroidal neovascularization (CNV) secondary to AMD in the study eye and/or fellow eye must be documented in the source document and CRF.	Section 7.5.1 Treatment of New Active Choroidal Neovascularization in the Study Eye and/or Fellow Eye<br>Section 9.15 Ocular Imaging
Optical Coherence Tomography Angiography (OCT-A) was added as a monitoring assessment for select sites to confirm the diagnosis of new active CNV.	Section 7.5.1 Treatment of New Active Choroidal Neovascularization in the Study Eye and/or Fellow Eye<br>Section 9.15 Ocular Imaging
Text corrected as follows:<br><br>The treatment method (pars plana vitrectomy vs intravitreal injection of antibiotics ~~vitreous tap~~) and choice of antimicrobial agents are also at the discretion of the physician and should follow current standard practice patterns.	Section 7.5.3 Endophthalmitis Treatment
Content in these sections was combined.	Section 7.5.1 Treatment of New Active Choroidal Neovascularization in the Study Eye and/or Fellow Eye<br><br>~~Section 7.5.2 Treatment of Neovascular AMD in the Fellow Eye~~
References to "biobanking" have been replaced by references to the "genetic biorepository" and the "clinical repository".	Section 8. STUDY DESIGN<br>Section 9.7 Genotyping Samples<br>Section 9.17 Blood Volume for Study Assessments
Protocol updated to specify that microperimetry should be conducted on both eyes.	Section 8 STUDY DESIGN
Noted that blood draws will be conducted for genotyping.	Section 8.1.3.2 Months 13-24
Noted that OCT-A may be conducted, if applicable as per instructions of the protocol.	Section 9.6 Laboratory Analysis of Blood and Urine
Text was modified to clarify that the study staff performing visual acuity	Section 9.11 Best-corrected Visual

should be masked to the treatment assignment only; the protocol previously stated that staff should also be blinded to the study eye.	Acuity and Low Luminance Best-corrected Visual Acuity
Blood volume for genetic biorepository was updated.	Section 9.17 Blood Volume for Study Assessments
The following samples were removed:<br>• ~~All residual serum and whole blood samples collected during the course of the study for PK analysis, genotyping and anti-APL-2 antibody formation~~<br>• ~~Residual serum PK sample~~<br>• ~~Residual serum Anti-Therapeutic Antibodies (ATA) sample~~<br><br>It was noted that the 15 mL whole blood sample will be collected at Month 2, ~~baseline~~, Month 12 and Month 24 of the study	Section 9.18.2 Sample Collection
It was clarified that the DMC will meet at the beginning of the study and every 6 months thereafter.	Section 10.1 Data Monitoring Committee
Reference to "possibly related" AEs was removed as there is no "possibly related" AE categorization for this study.<br><br>Resolution outcome possibilities were updated to accurately align with the SAE report forms that will be used for this study.	Section 11.2 Recording Adverse Events<br><br>Section 11.4.1 Relationship of Events to Study Treatment
Clarified that:<br>All SAEs will be reported to the Safety Monitor by the Investigator via fax or email within one ~~working~~ calendar day of becoming aware of the event, whether or not the serious events are deemed drug-related.	Section 11.5 Serious Adverse Events
Section reorganized, modified, and updated to depict protocol specifications for data management and statistical considerations more clearly, accurately, and thoroughly.	Section 12. DATA MANAGEMENT AND STATISTICAL CONSIDERATIONS
Footnote was updated to indicate that: **All assessments should be performed on the same day~~, with the exception of screening~~.**<br><br>The blood draw for the clinical repository was removed at baseline, Month 1, Month 8, Month 18, and Month 27.<br><br>OCT-A was added at screening, Month 6, Month 12, Month 18, and Month 24 for select sites. Footnote "R" was added to note that in addition to the time points indicated on the study schedule, OCT-A should be performed at the time of any suspected new active CNV. If new active CNV is confirmed in the study eye, OCT-A should be repeated every 2 months for the study eye.	All Study Schedules (Appendices: A, B, C, and D)

APPENDIX K: PROTOCOL CHANGES TO BE FOLLOWED DURING COVID-19 RESTRICTIONS

OVERVIEW

In response to the COVID-19 crisis, to ensure the safety of study subjects and Investigative Sites as well as proper conduct of the study, TEMPORARY changes to the protocol have been implemented. These changes should be followed only during COVID-19 restrictions and include extended IP administration windows, changes to masking rules, rescreening instructions, and a revised schedule of assessments.

Where feasible, sites could continue to follow the full schedule of assessments (based on their treatment group assignment).

EXTENDED IP ADMINISTRATION WINDOWS

In order to allow more flexibility to sites and subjects, and to potentially mitigate missed IP administration, an extended IP administration window can be followed. The extended window can ONLY be used in situations related to COVID-19 restrictions and after medical monitor approval. Footnote "T" of each COVID-19 assessment table below reflects these extended IP options.

Per protocol, the study window is as followed:

- Monthly treatment group: +/–8 days for the entire study duration
- EOM treatment group: +/–8 days for the first study year and +/–16 days for the second study year

During COVID-19 restrictions, the IP administration window can be extended to the following:

- Monthly treatment group: –8 days to +15 days. Note, interval for consecutives injection must be at least 14 days.
- EOM treatment group: –8 days to +30 days for the first study year and –16 days to +30 days for the second study year

MASKING RULES

Due to current COVID-19 restrictions, clinical sites might encounter difficulties maintaining appropriate clinic staffing to satisfy the approved masking rules for the APL2-304 (Oaks) study. Based on this, and in an attempt to minimize the amount of missed data and IP administrations, Apellis is implementing a temporary adjustment to the study masking rules.

This temporary change must be approved by the Apellis Medical Director **prior to** implementation and must be documented via a temporary and modified Delegation of Authority Log. Each masked assessment performed by an unmasked staff and vice versa (even with Apellis approval and following the below guidelines) should be documented.

The principal investigator (PI) is responsible for the overall safety oversight of the study site data and s/he will not be allowed to switch into an unmasked role. Every masked individual that performs IP administration and/or postinjection assessment (all unmasked assessment) as a temporary measure, will **permanently** be considered an unmasked individual and will not be able to perform masked assessments once these exemptions are lifted.

RESCREENING PROCEDURE

Prior to the implementation of these temporary changes, sites continuing to screen patients have been encouraged to complete the screening and baseline assessments in their entirety. However, if a subject was deemed a screen failure for not being able to meet the original screening window (Day –28 to Day –1 [+/–2 days]) due to COVID-19 related restrictions, a rescreening visit is allowed and should be followed according to the 2 scenarios below.

Subjects Who Completed Screening and Were Considered Eligible by Reading Center and Investigator

Subjects who were screened prior to 30 March 2020 and completed all screening assessments (as described in the Schedule of Assessments Appendix A [every month treatment group] and Appendix C [every-other-month [EOM] treatment group]) and considered eligible by the reading center and investigator and are able to return to the clinic within 90 days of initial screening, will receive a new subject ID number and undergo an *abbreviated screening*, prior to randomization, that includes the following assessments:

- Informed consent/Assign new screening number
- NL-BCVA assessment
- Slitlamp examination
- Dilated indirect ophthalmoscopy
- IOP measurement
- SD-OCT*
- Concomitant medication/concomitant ocular procedures collection
- AE collection

*SD-OCT images collected at this visit will not be used by the reading center to determine eligibility but should be used by the investigator to detect any potential new exclusion criterion.

If the investigator deems it necessary, additional assessments can be performed if there is a concern that the subject might now meet an exclusion criterion that was not the case during the original screening (eg, fundus fluorescein angiography to exclude the presence of CNV).

Subjects With Incomplete Screening Assessment

Subjects that signed the informed consent but were not able to complete all screening assessments due to COVID-19-related restrictions are not eligible for the abbreviated screening. These subjects can be rescreened but must follow the standard screening schedule of assessment (as described in the Schedule of Assessments Appendix A [every month treatment group] and Appendix C [EOM treatment group]). These subjects will also receive a new screening ID number.

MINIMUM SCHEDULE OF ASSESSMENT

Schedule of Assessments

Where feasible, sites could continue to follow the full schedule of assessments (based on their treatment group assignment). The minimum assessment tables, only to be followed during this

COVID-19 effort and if determined necessary to use based on the investigator's clinical judgment, are provided below to reduce the time required for each study visit. Subjects in the EOM treatment group do not need to be seen for the non-IP administration visits. Assessments not performed (even those that have been removed in the minimum assessment table) should be documented.

Subjects that are not able to come into the clinic for a study visit due to COVID-19-related restrictions, including visits for the EOM group that do not include IP administration, should be contacted via the phone for the collection of adverse events (including SAEs) and concomitant medications. **All SAEs are still required to be reported to Apellis within 24 hours of site awareness, even if reported via phone call**. All communications via phone call should also be documented in the source documentation and in the respective CRF page. In addition, these subjects should be instructed to self-monitor their vision at home and report any changes in vision or their overall health via phone call. The site must inform the Sponsor of any subjects lost to follow-up.

It is critical that local, country, and regional governance regarding COVID-19 is followed along with your best clinical judgment when managing this situation. All visits or assessments missed as a result of COVID-19 will be captured in the Case Report Forms.

The Schedule of Assessments tables shown below supersede those sent on 30 March 2020. These changes were previously communicated to the sites via a memorandum.

APPENDIX A (TRACKED): COVID-19 VISIT SCHEDULE—Monthly Group—Screening, Day 1 through Month 12

	Screening	Treatment													Early Term[A]
Visit #	1	2	3	4	5	6	7	8	9	10	11	12	13	14	
Day	–28 to –1	1	30	60	90	120	150	180	210	240	270	300	330	360	
Week	0	0	4	8	12	16	20	24	28	32	36	40	44	48	
Month	0	0	1	2	3	4	5	6	7	8	9	10	11	12	
Window (+ or – days)	2	0	8	8	8	8	8	8	8	8	8	8	8	8	
Informed Consent / Assign Screening Number	x														
Demographic Data	x														
Inclusion/Exclusion Criteria [B]	x	x													
Medical/Surgical/Ocular History [C]	x														
Blood Draw—Safety Labs [D,E,F]	x	x		~~x~~				~~x~~						x	x
Urine Sample Collection [D, E,F]	x	x		~~x~~				~~x~~						x	x
Urine Pregnancy Test [D,E,F]		x	x	x	x	x	x	x	x	x	x	x	x	x	
Blood Draw—Anti-Pegcetacoplan Ab [D]		x	~~x~~	~~x~~				~~x~~						x	x
Blood Draw—Genotyping (if applicable) [D]				~~x~~											
Blood Draw for Clinical Repository (if applicable)[D,G]				~~x~~				~~x~~						~~x~~	~~x~~
Vital Signs [H]	x	x	~~x~~	~~x~~	~~x~~	~~x~~	~~x~~	x	~~x~~	~~x~~	~~x~~	~~x~~	~~x~~	x	x
Physical Examination [I]	x													x	x
BCVA [J]	x	x	x	x	x	x	x	x	x	x	x	x	x	x	x
LL-BCVA [J]		x	~~x~~	~~x~~	~~x~~	~~x~~	~~x~~	x	~~x~~	~~x~~	~~x~~	~~x~~	~~x~~	x	x
MNREAD or Radner Reading Charts (select countries) [J,K]		x						~~x~~						x	x
Mesopic Microperimetry[L]	x							~~SE~~						x	x
Slitlamp Examination	x	x	x	x	x	x	x	x	x	x	x	x	x	x	x
Endothelial Cell Count[S]		x						x						x	x
NEI VFQ-25 [M]		x						~~x~~						x	x
FRI [M]		x						~~x~~						x	x
Home-Based Digital Applications [M,N,S]		x	~~x~~	~~x~~	~~x~~			~~x~~						x	
Dilated Indirect Ophthalmoscopy	x	x	x	x	x	x	x	x	x	x	x	x	x	x	x
IOP Measurement	x	x	x	x	x	x	x	x	x	x	x	x	x	x	x
SD-OCT [O]	x	x	~~x~~	~~x~~	~~x~~	~~x~~	~~x~~	x	~~x~~	~~x~~	~~x~~	~~x~~	~~x~~	x	x
FAF [O]	x	x		~~SE~~		~~SE~~		x		~~SE~~		~~SE~~		x	x
NIR [O]	x	x		~~SE~~		~~SE~~		x		~~SE~~		~~SE~~		x	x

APPENDIX A (TRACKED): <u>COVID-19</u> VISIT SCHEDULE—Monthly Group—Screening, Day 1 through Month 12

	Screening	Treatment													Early Term[A]
**Visit #**	1	2	3	4	5	6	7	8	9	10	11	12	13	14	
**Day**	–28 to –1	1	30	60	90	120	150	180	210	240	270	300	330	360	
**Week**	0	0	4	8	12	16	20	24	28	32	36	40	44	48	
**Month**	0	0	1	2	3	4	5	6	7	8	9	10	11	12	
**Window (+ or – days)**	2	0	8	8	8	8	8	8	8	8	8	8	8	8	
**DCFP [O]**	x													x	x
**FFA[O]**	x													x	x
**OCT-A[S]**		X[S]						~~x[S]~~						x[S]	x
**Study Eye Determination**	x														
**Randomization**		x													
**Pegcetacoplan administration or Sham Injection[T]**		x	x	x	x	x	x	x	x	x	x	x	x	x	
**Postinjection Assessment [P]**		x	x	x	x	x	x	x	x	x	x	x	x	x	
**Follow-Up Call [Q]**		x	x	x	x										
**Concomitant Medication/ Concomitant Ocular Procedures [R]**	x	x	x	x	x	x	x	x	x	x	x	x	x	x	x
**Adverse Events**	x	x	x	x	x	x	x	x	x	x	x	x	x	x	x

Abbreviations: BCVA=best corrected visual acuity; CNV=choroidal neovascularization; DCFP=digital color fundus photography; eCRF=electronic case report form; FAF=fundus autofluorescence; FFA=fundus fluorescein angiography; FRI=Functional Reading Independence index; IOP=intraocular pressure; LL BCVA= low luminance best corrected visual acuity; MNREAD=Minnesota Low-Vision Reading Test; NEI VFG-25=National Eye Institute Visual Functioning Questionnaire 25-item Version; NIR=near infrared reflectance; OCT-A=optical coherence tomography angiography; SD-OCT=spectral domain optical coherence tomography; SE=study eye; Term=termination; VEGF=vascular endothelial growth factor.

Note: All ocular assessments are to be performed for both eyes unless annotated with 'SE' (study eye) in the above schedule. All assessments should be performed on the same day. All study visits should be scheduled and projected based on the Day 1 visit date.

A. For subjects that discontinue the study early, the early termination assessments should be performed after a minimum of 30 days have passed from the last dosing visit. If a subject reports for a scheduled visit and decides to terminate early prior to dosing, then the visit should be considered the early termination visit and all early termination procedures should be performed. At Month 24, all subjects should be offered entry into an open-label study.

B. At Day 1 (Visit 2), confirm subject eligibility through reviewing the inclusion/ exclusion criteria and receive confirmation of eligibility from the reading center.

C. Significant medical/ surgical history from the previous 5 years. Anti-VEGF treatments (fellow eye) and invasive ocular procedures performed within the past 5 years and while on study should also be recorded. Any history of tobacco use should be recorded.

D. Obtain prior to fluorescein angiography and before study drug administration.

E. At screening, serum pregnancy should be performed for women of childbearing potential. If positive, subject is not eligible to continue in the study.

F. Beginning at Day 1, perform the urine pregnancy test for women of childbearing potential at each treatment visit. If positive, perform a serum pregnancy test. If serum test is positive, study drug should not be administered and an early term visit should be completed.

G. Only subjects that sign the separate consent for the clinical repository will have these samples collected. A 14-mL whole-blood sample will be collected at each of the specified visits.

H. Blood pressure, respiratory rate, heart rate, and temperature. On dosing days, vital signs should be taken predose.

I. Height and weight should be collected at screening.

J. Perform assessments prior to dilating the eyes.
K. In select countries, the MNREAD or Radner Reading Charts should be done during the study visit, prior to dilating the eyes. It should be performed monocularly first, then binocularly.
L. Microperimetry assessments will be performed post dilation. Data will be forwarded to the reading center.
M. To be administered by the masked site staff prior to any other assessments performed on that day. In-clinic assessments on the digital application should be completed after completion of all functional tests and quality of life measures prior to dilating the eyes.
N. At select sites, and for those subjects who decide to participate, the digital applications will be completed on an electronic device and will consist of assessments for visual function and reading speed. Subjects will receive training at Day 1 on the use of the digital applications and the electronic device and will perform the tests using the application in the clinic at Month 1, Month 2, Month 3, Month 6, Month 12, Month 18, and Month 24. Beginning at Day 1, subjects will complete the functional assessments weekly at home. Subjects should be instructed to complete the assessments on the same day each week and at approximately the same time of day each week, if possible.
O. FAF and SD-OCT images, near infrared reflectance, fluorescein angiograms, and fundus photographs will be performed for the study eye only on days where 'SE' is specified and for both eyes at all other visits as specified in the above schedule and will be sent to the reading center for evaluation (consult the reading center manual for specifics on image capture, processing, and transmission). Images should be captured prior to dosing on dosing days. If a subject misses a study visit or images cannot be obtained at a specific visit, study staff should make every effort to obtain images at the next scheduled visit. If new active CNV is suspected, SD-OCT, FFA and OCT-A (select sites) images must be collected and sent to the reading center for analysis.
P. Postinjection assessments should be performed within 5 minutes after dosing by the unmasked physician or study staff and should include a gross assessment of vision (finger-counting, hand motion, then light perception when applicable). If subject passes gross vision test, the subject may leave the site. If subject fails gross vision test, the tonometry should be performed. IOP should be ≤30 mm Hg in order for the subject to leave the site. If necessary, antiglaucomatous medication can be given to lower the IOP. If IOP is >30 mm Hg, assessments will continue every approximately 30 minutes from the previous measurement until the subject passes gross vision test and IOP is ≤30 mm Hg. Note: if the study eye is treated with a ranibizumab or aflibercept injection during the same visit as the study treatment (pegcetacoplan or sham), the treatment with ranibizumab or aflibercept must be performed first. The pre- and post- anti-VEGF IOP values must be measured and recorded on the eCRF.
Q. Starting at Day 1, study subjects will be contacted by study site staff within 4 ± 2 days after each study treatment visit (through Month 3) to collect any information on any safety concerns, decrease in vision, eye pain, unusual ocular events, or any new ocular symptoms in the study eye. If the physician determines that there are any safety concerns, a follow-up visit should be scheduled as soon as possible.
R. Record concomitant medications (ie, prescription and over-the-counter medications) used by the patient within 30 days of screening and throughout the subject's participation in the study.
S. In addition to the time points indicated on the study schedule, OCT-A should be performed at the time of any suspected new active CNV. If new active CNV is confirmed in the study eye, OCT-A should be repeated every 2 months for the study eye.
T. Administration of pegcetacoplan or sham can be done on separate days from the assessment visit if both days fall within the visit window. Administration of study treatment (pegcetacoplan or sham) can be done on a separate day from the assessment visit if both days fall within the visit window. If this occurs on the randomization visit, then the administration of pegcetacoplan or sham should be done within 3 days of randomization and after approval from the medical monitor. When study treatment administration is on a day other than a study visit, then the only assessment that must be done on the day of study treatment administration is preinjection IOP. During the COVID-19 pandemic, the following flexibility is allowed for IP administration: –8 days to + 15 days after medical monitor approval. Note, interval for consecutive injections must be at least 14 days.

APPENDIX B (TRACKED): COVID-19 VISIT SCHEDULE—Monthly Group—Month 13 to Month 24

	Treatment												Early Term[A]
Visit #	15	16	17	18	19	20	21	22	23	24	25	26	
Day	390	420	450	480	510	540	570	600	630	660	690	720	
Week	52	56	60	64	68	72	76	80	84	88	92	96	
Month	13	14	15	16	17	18	19	20	21	22	23	24	
Window (+ or – days)	8	8	8	8	8	8	8	8	8	8	8	8	
Informed Consent / Assign Screening Number													
Demographic Data													
Inclusion/Exclusion Criteria [B]													
Medical/Surgical/Ocular History [C]													
Blood Draw—Safety Labs [D,E,F]						~~x~~						x	x
Urine Sample Collection [D,E,F]						~~x~~						x	x
Urine Pregnancy Test [D,E,F]	x	x	x	x	x	x	x	x	x	x	x	x	
Blood Draw—Anti-Pegcetacoplan Ab [D]		~~x~~				~~x~~						x	x
Blood Draw—Genotyping (if applicable) [D]													
Blood Draw for Clinical Repository (if applicable)[D,G]												~~x~~	~~x~~
Vital Signs [H]	~~x~~	~~x~~	~~x~~	~~x~~	~~x~~	x	~~x~~	~~x~~	~~x~~	~~x~~	~~x~~	x	x
Physical Examination [I]												x	x
BCVA [J]	x	x	x	x	x	x	x	x	x	x	x	x	x
LL-BCVA [I]	~~x~~	~~x~~	~~x~~	~~x~~	~~x~~	x	~~x~~	~~x~~	~~x~~	~~x~~	~~x~~	x	x
MNREAD or Radner Reading Charts (select countries) [J,K]						~~x~~						x	x
Mesopic Microperimetry[L]						~~SE~~						x	x
Slitlamp Examination	x	x	x	x	x	x	x	x	x	x	x	x	x
Endothelial Cell Count[S]												x	x
NEI VFQ-25 [M]						~~x~~						x	x
FRI [M]						~~x~~						x	x
Dilated Indirect Ophthalmoscopy	x	x	x	x	x	x	x	x	x	x	x	x	x
Home-Based Digital Applications [M,N,S]						~~x~~						~~x~~	
IOP Measurement	x	x	x	x	x	x	x	x	x	x	x	x	x
SD-OCT [O]	~~x~~	~~x~~	~~x~~	~~x~~	~~x~~	x	~~x~~	~~x~~	~~x~~	~~x~~	~~x~~	x	x
FAF [O]		~~SE~~		~~SE~~		x		~~SE~~		~~SE~~		x	x
NIR [O]		~~SE~~		~~SE~~		x		~~SE~~		~~SE~~		x	x

APPENDIX B (TRACKED): COVID-19 VISIT SCHEDULE—Monthly Group—Month 13 to Month 24

	Treatment												Early Term[A]
Visit #	15	16	17	18	19	20	21	22	23	24	25	26	
Day	390	420	450	480	510	540	570	600	630	660	690	720	
Week	52	56	60	64	68	72	76	80	84	88	92	96	
Month	13	14	15	16	17	18	19	20	21	22	23	24	
Window (+ or – days)	8	8	8	8	8	8	8	8	8	8	8	8	
DCFP [O]												x	x
FFA [O]												x	x
OCT-A[S]						~~x~~[S]						x [S]	X
Study Eye Determination													
Randomization													
Pegcetacoplan administration or Sham Injection[T]	x	x	x	x	x	x	x	x	x	x	x		
Postinjection Assessment [P]	x	x	x	x	x	x	x	x	x	x	x		
Follow-Up Call [Q]													
Concomitant Medication/ Concomitant Ocular Procedures [R]	x	x	x	x	x	x	x	x	x	x	x	x	x
Adverse Events	x	x	x	x	x	x	x	x	x	x	x	x	x

Abbreviations: BCVA=best corrected visual acuity; CNV=choroidal neovascularization; DCFP=digital color fundus photography; eCRF=electronic case report form; FAF=fundus autofluorescence; FFA=fundus fluorescein angiography; FRI=Functional Reading Independence index; IOP=intraocular pressure; LL BCVA= low luminance best corrected visual acuity; MNREAD=Minnesota Low-Vision Reading Test; NEI VFG-25=National Eye Institute Visual Functioning Questionnaire 25-item Version; NIR=near infrared reflectance; OCT-A=optical coherence tomography angiography; SD-OCT=spectral domain optical coherence tomography; SE=study eye; Term=termination; VEGF=vascular endothelial growth factor.

Note: All ocular assessments are to be performed for both eyes unless annotated with 'SE' (study eye) in the above schedule. All assessments should be performed on the same day. All study visits should be scheduled and projected based on the Day 1 visit date.

A. For subjects that discontinue the study early, the early termination assessments should be performed after a minimum of 30 days have passed from the last dosing visit. If a subject reports for a scheduled visit and decides to terminate early prior to dosing, then the visit should be considered the early termination visit and all early termination procedures should be performed. At Month 24, all subjects should be offered entry into an open-label study.

B. At Day 1 (Visit 2), confirm subject eligibility through reviewing the inclusion/ exclusion criteria and receive confirmation of eligibility from the reading center.

C. Significant medical/ surgical history from the previous 5 years. Anti-VEGF treatments (fellow eye) and invasive ocular procedures performed within the past 5 years and while on study should also be recorded. Any history of tobacco use should be recorded.

D. Obtain prior to fluorescein angiography and before study drug administration.

E. At screening, serum pregnancy should be performed for women of childbearing potential. If positive, subject is not eligible to continue in the study.

F. Beginning at Day 1, perform the urine pregnancy test for women of childbearing potential at each treatment visit. If positive, perform a serum pregnancy test. If serum test is positive, study drug should not be administered and an early term visit should be completed.

G. Only subjects that sign the separate consent for the clinical repository will have these samples collected. A 14-mL whole-blood sample will be collected at each of the specified visits.

H. Blood pressure, respiratory rate, heart rate, and temperature. On dosing days, vital signs should be taken predose.

I. Height and weight should be measured at screening.

J. Perform assessments prior to dilating the eyes.

K. In select countries, the MNREAD or Radner Reading Charts should be done during the study visit, prior to dilating the eyes. It should be performed monocularly first, then binocularly.

L. Microperimetry assessments will be performed post dilation. Data will be forwarded to the reading center.

M. To be administered by the masked site staff prior to any other assessments performed on that day. In-clinic assessments on the digital application should be completed after completion of all functional tests and quality of life measures prior to dilating the eyes.

N. At select sites, and for those subjects who decide to participate, the digital applications will be completed on an electronic device and will consist of assessments for visual function and reading speed. Subjects will receive training at Day1 on the use of the digital applications and the electronic device and will perform the tests using the application in the clinic at Month 1, Month 2, Month 3, Month 6, Month 12, Month 18, and Month 24. Beginning at Day 1, subjects will complete the functional assessments weekly at home. Subjects should be instructed to complete the assessments on the same day each week and at approximately the same time of day each week, if possible.

O. FAF and SD-OCT images, near infrared reflectance, fluorescein angiograms, and fundus photographs will be performed for the study eye only on days where 'SE' is specified and for both eyes at all other visits as specified in the above schedule and will be sent to the reading center for evaluation (consult the reading center manual for specifics on image capture, processing, and transmission). Images should be captured prior to dosing on dosing days. If a subject misses a study visit or images cannot be obtained at a specific visit, study staff should make every effort to obtain images at the next scheduled visit. If new active CNV is suspected, SD-OCT, FFA, and OCT-A (selected sites) images should be collected and sent to the reading center for analysis.

P. Postinjection assessments should be performed within 5 minutes after dosing by the unmasked physician or study staff and should include a gross assessment of vision (finger-counting, hand motion, then light perception when applicable). If subject passes gross vision test, the subject may leave the site. If subject fails gross vision test, the tonometry should be performed. IOP should be ≤30 mm Hg in order for the subject to leave the site. If necessary, antiglaucomatous medication can be given to lower the IOP. If IOP is >30 mm Hg, assessments will continue every approximately 30 minutes from the previous measurement until the subject passes gross vision test and IOP is ≤30 mm Hg. Note: if the study eye is treated with a ranibizumab or aflibercept injection during the same visit as the study treatment (pegcetacoplan or sham), the treatment with ranibizumab or aflibercept must be performed first. The pre- and post- anti-VEGF IOP values must be measured and recorded on the eCRF.

Q. Starting at Day 1, study subjects will be contacted by study site staff within 4 ± 2 days after each study treatment visit (through Month 3) to collect any information on any safety concerns, decrease in vision, eye pain, unusual ocular events, or any new ocular symptoms in the study eye. If the physician determines that there are any safety concerns, a follow-up visit should be scheduled as soon as possible.

R. Record concomitant medications (ie, prescription and over-the-counter medications) used by the patient within 30 days of screening and throughout the subject's participation in the study.

S. In addition to the time points indicated on the study schedule, OCT-A should be performed at the time of any suspected new active CNV. If new active CNV is confirmed in the study eye, OCT-A should be repeated every 2 months for the study eye.

T. Administration of pegcetacoplan or sham can be done on separate days from the assessment visit if both days fall within the visit window. Administration of study treatment (pegcetacoplan or sham) can be done on a separate day from the assessment visit if both days fall within the visit window. If this occurs on the randomization visit, then the administration of pegcetacoplan or sham should be done within 3 days of randomization and after approval from the medical monitor. When study treatment administration is on a day other than a study visit, then the only assessment that must be done on the day of study treatment administration is preinjection IOP. During the COVID-19 pandemic, the following flexibility is allowed for IP administration: –8 days to + 15 days after medical monitor approval. Note, interval for consecutive injections must be at least 14 days.

APPENDIX C (TRACKED): COVID-19 VISIT SCHEDULE—Every-Other-Month Group—Screening, Day 1 Through Month 12

	Screening	Treatment													Early Term[A]
Visit #	1	2	3	4	5	6	7	8	9	10	11	12	13	14	
Day	–28 to –1	1	30	60	90	120	150	180	210	240	270	300	330	360	
Week	0	0	4	8	12	16	20	24	28	32	36	40	44	48	
Month	0	0	1	2	3	4	5	6	7	8	9	10	11	12	
Window (+ or – days)	2	0	8	8	8	8	8	8	8	8	8	8	8	8	
**Informed Consent / Assign Screening Number**	x														
**Demographic Data**	x														
**Inclusion/Exclusion Criteria** [B]	x	x													
**Medical/Surgical/Ocular History** [C]	x														
**Blood Draw—Safety Labs** [D,E,F]	x	x		~~x~~				~~x~~						x	x
**Urine Sample Collection** [D,E,F]	x	x		~~x~~				~~x~~						x	x
**Urine Pregnancy Test** [D,E,F]		x		x		x		x		x		x		x	
**Blood Draw—Anti-Pegcetacoplan Ab** [D]		x	~~x~~	~~x~~				~~x~~						x	x
**Blood Draw—Genotyping (if applicable)** [D]				~~x~~											
**Blood Draw for Clinical Repository (if applicable)** [D,G]				~~x~~				~~x~~						~~x~~	~~x~~
**Vital Signs** [H]	x	x	~~x~~	~~x~~	~~x~~	~~x~~	~~x~~	x	~~x~~	~~x~~	~~x~~	~~x~~	~~x~~	x	x
**Physical Examination** [I]	x													x	x
**BCVA** [J]	x	x	~~x~~	x	~~x~~	x	~~x~~	x	~~x~~	x	~~x~~	x	~~x~~	x	x
**LL-BCVA** [J]		x	~~x~~	~~x~~	~~x~~	~~x~~	~~x~~	x	~~x~~	~~x~~	~~x~~	~~x~~	~~x~~	x	x
**MNREAD or Radner Reading Charts (select countries)** [J,K]		x						~~x~~						x	x
**Mesopic Microperimetry**[L]	x							~~SE~~						x	x
**Slitlamp Examination**	x	x	~~x~~	x	~~x~~	x	~~x~~	x	~~x~~	x	~~x~~	x	~~x~~	x	x
**Endothelial Cell Count**[S]		x						x						x	x
**NEI VFQ-25** [M]		x						~~x~~						x	x
**FRI** [M]		x						~~x~~						x	x
**Home-Based Digital Applications** [M,N,S]		x	~~x~~	~~x~~	~~x~~			~~x~~						x	
**Dilated Indirect Ophthalmoscopy**	x	x	~~x~~	x	~~x~~	x	~~x~~	x	~~x~~	x	~~x~~	x	~~x~~	x	x
**IOP Measurement**	x	x	~~x~~	x	~~x~~	x	~~x~~	x	~~x~~	x	~~x~~	x	~~x~~	x	x
**SD-OCT** [O]	x	x	~~x~~	~~x~~	~~x~~	~~x~~	~~x~~	x	~~x~~	~~x~~	~~x~~	~~x~~	~~x~~	x	x
**FAF** [O]	x	x		~~SE~~		~~SE~~		x		~~SE~~		~~SE~~		x	x
**NIR** [O]	x	x		~~SE~~		~~SE~~		x		~~SE~~		~~SE~~		x	x

APPENDIX C (TRACKED): COVID-19 VISIT SCHEDULE—Every-Other-Month Group—Screening, Day 1 Through Month 12

	Screening	Treatment													Early Term[A]
Visit #	1	2	3	4	5	6	7	8	9	10	11	12	13	14	
Day	–28 to –1	1	30	60	90	120	150	180	210	240	270	300	330	360	
Week	0	0	4	8	12	16	20	24	28	32	36	40	44	48	
Month	0	0	1	2	3	4	5	6	7	8	9	10	11	12	
Window (+ or – days)	2	0	8	8	8	8	8	8	8	8	8	8	8	8	
**DCFP** [O]	x													x	x
**FFA** [O]	x													x	x
**OCT-A**[S]		x[S]						~~x[S]~~						x[S]	x
**Study Eye Determination**	x														
**Randomization**		x													
**Pegcetacoplan administration or Sham Injection**[T]		x		x		x		x		x		x		x	
**Postinjection Assessment** [P]		x		x		x		x		x		x		x	
**Follow-Up Call** [Q]		x		x		x									
**Concomitant Medication/ Concomitant Ocular Procedures** [R]	x	x	~~x~~	x	~~x~~	x	~~x~~	x	~~x~~	x	~~x~~	x	~~x~~	x	x
**Adverse Events**	x	x	~~x~~	x	~~x~~	x	~~x~~	x	~~x~~	x	~~x~~	x	~~x~~	x	x

Abbreviations: BCVA=best corrected visual acuity; CNV=choroidal neovascularization; DCFP=digital color fundus photography; eCRF=electronic case report form; FAF=fundus autofluorescence; FFA=fundus fluorescein angiography; FRI=Functional Reading Independence index; IOP=intraocular pressure; LL BCVA= low luminance best corrected visual acuity; MNREAD=Minnesota Low-Vision Reading Test; NEI VFG-25=National Eye Institute Visual Functioning Questionnaire 25-item Version; NIR=near infrared reflectance; OCT-A=optical coherence tomography angiography; SD-OCT=spectral domain optical coherence tomography; SE=study eye; Term=termination; VEGF=vascular endothelial growth factor.

Note: All ocular assessments are to be performed for both eyes unless annotated with 'SE' (study eye) in the above schedule. All assessments should be performed on the same day. All study visits should be scheduled and projected based on the Day 1 visit date.

A. For subjects that discontinue the study early, the early termination assessments should be performed after a minimum of 30 days have passed from the last dosing visit. If a subject reports for a scheduled visit and decides to terminate early prior to dosing, then the visit should be considered the early termination visit and all early termination procedures should be performed. At Month 24, all subjects should be offered entry into an open-label study.

B. At Day 1 (Visit 2), confirm subject eligibility through reviewing the inclusion/ exclusion criteria and receive confirmation of eligibility from the reading center.

C. Significant medical/ surgical history from the previous 5 years. Anti-VEGF treatments (fellow eye) and invasive ocular procedures performed within the past 5 years and while on study should also be recorded. Any history of tobacco use should be recorded.

D. Obtain prior to fluorescein angiography and before study drug administration.

E. At screening, serum pregnancy should be performed for women of childbearing potential. If positive, subject is not eligible to continue in the study.

F. Beginning at Day 1, perform the urine pregnancy test for women of childbearing potential at each treatment visit. If positive, perform a serum pregnancy test. If serum test is positive, study drug should not be administered and an early term visit should be completed.

G. Only subjects that sign the separate consent for genetic biorepository will have these samples collected. A 14-mL whole-blood sample will be collected at each of the specified visits.

H. Blood pressure, respiratory rate, heart rate, and temperature. On dosing days, vital signs should be taken predose.

I. Height and weight should be measured at screening.

J. Perform assessments prior to dilating the eyes.

K. In select countries, the MNREAD or Radner Reading Charts should be done during the study visit, prior to dilating the eyes. It should be performed monocularly first, then binocularly.
L. Microperimetry assessments will be performed post dilation. Data will be forwarded to the reading center.
M. To be administered by the masked site staff prior to any other assessments performed on that day. In-clinic assessments on the digital application should be completed after completion of all functional tests and quality of life measures prior to dilating the eyes.
N. At select sites, and for those subjects who decide to participate, the digital application will be completed on an electronic device and will consist of assessments for visual function and reading speed. Subjects will receive training at Day 1 on the use of the digital applications and the electronic device and will perform the tests using the in the clinic at Month 1, Month 2, Month 3, Month 6, Month 12, Month 18, and Month 24. Beginning at Day 1, subjects will complete the functional assessments weekly at home. Subjects should be instructed to complete the assessments on the same day each week and at approximately the same time of day each week, if possible.
O. FAF and SD-OCT images, near infrared reflectance, fluorescein angiograms, and fundus photographs will be performed for the study eye only on days where 'SE' is specified and for both eyes at all other visits as specified in the above schedule and will be sent to the reading center for evaluation (consult the reading center manual for specifics on image capture, processing, and transmission). Images should be captured prior to dosing on dosing days. If a subject misses a study visit or images cannot be obtained at a specific visit, study staff should make every effort to obtain images at the next scheduled visit. If new active CNV is suspected, SD-OCT, FFA, and OCT-A (selected sites) images should be collected and sent to the reading center for analysis.
P. Postinjection assessments should be performed within 5 minutes after dosing by the unmasked physician or study staff and should include a gross assessment of vision (finger-counting, hand motion, then light perception when applicable). If subject passes gross vision test, the subject may leave the site. If subject fails gross vision test, the tonometry should be performed. IOP should be ≤30 mm Hg in order for the subject to leave the site. If necessary, antiglaucomatous medication can be given to lower the IOP. If IOP is >30 mm Hg, assessments will continue every approximately 30 minutes from the previous measurement until the subject passes gross vision test and IOP is ≤30 mm Hg. Note: if the study eye is treated with a ranibizumab or aflibercept injection during the same visit as the study treatment (pegcetacoplan or sham), the treatment with ranibizumab or aflibercept must be performed first. The pre- and post- anti-VEGF IOP values must be measured and recorded on the eCRF.
Q. Starting at Day 1, study subjects will be contacted by study site staff within 4 ± 2 days after each study treatment visit (through Month 4) to collect any information on any safety concerns, decrease in vision, eye pain, unusual ocular events, or any new ocular symptoms in the study eye. If the physician determines that there are any safety concerns, a follow-up visit should be scheduled as soon as possible.
R. Record concomitant medications (ie, prescription and over-the-counter medications) used by the patient within 30 days of screening and throughout the subject's participation in the study.
S. In addition to the time points indicated on the study schedule, OCT-A should be performed at the time of any suspected new active CNV. If new active CNV is confirmed in the study eye, OCT-A should be repeated every 2 months for the study eye.
T. Administration of pegcetacoplan or sham can be done on separate days from the assessment visit if both days fall within the visit window. Administration of study treatment (pegcetacoplan or sham) can be done on a separate day from the assessment visit if both days fall within the visit window. If this occurs on the randomization visit, then the administration of pegcetacoplan or sham should be done within 3 days of randomization and after approval from the medical monitor. When study treatment administration is on a day other than a study visit, then the only assessment that must be done on the day of study treatment administration is preinjection IOP. During the COVID-19 pandemic, the following flexibility is allowed for IP administration: –8 days to + 30 days after medical monitor approval.

APPENDIX D (TRACKED): COVID-19 VISIT SCHEDULE—Every-Other-Month Group Month 13 to Month 24

	Treatment												Early Term [A]
Visit #		15		16		17		18		19		20	
Day	390	420	450	480	510	540	570	600	630	660	690	720	
Week	52	56	60	64	68	72	76	80	84	88	92	96	
Month	13	14	15	16	17	18	19	20	21	22	23	24	
Window (+ or – days)		16		16		16		16		16		16	
Informed Consent / Assign Screening Number													
Demographic Data													
Inclusion/Exclusion Criteria [B]													
Medical/Surgical/Ocular History [C]													
Blood Draw—Safety Labs [D,E,F]						~~x~~						x	x
Urine Sample Collection [D, E,F]						~~x~~						x	x
Urine Pregnancy Test [D,E,F]		x		x		x		x		x		x	
Blood Draw—Anti-Pegcetacoplan Ab [D]		~~x~~				~~x~~						x	x
Blood Draw—Genotyping (if applicable) [D]													
Blood Draw for Clinical Repository (if applicable) [D,G]												~~x~~	~~x~~
Vital Signs [H]		~~x~~		~~x~~		x		~~x~~		~~x~~		x	x
Physical Examination [I]												x	x
BCVA [J]		x		x		x		x		x		x	x
LL-BCVA [J]		~~x~~		~~x~~		x		~~x~~		~~x~~		x	x
MNREAD or Radner Reading Charts (select countries) [J,K]						~~x~~						x	x
Mesopic Microperimetry [L]						~~SE~~						x	x
Slitlamp Examination		x		x		x		x		x		x	x
Endothelial Cell Count [S]												x	x
NEI VFQ-25 [M]						~~x~~						x	x
FRI [M]						~~x~~						x	x
Home-Based Digital Applications [M,N,S]						~~x~~						~~x~~	
Dilated Indirect Ophthalmoscopy		x		x		x		x		x		x	x
IOP Measurement		x		x		x		x		x		x	x
SD-OCT [O]		~~x~~		~~x~~		x		~~x~~		~~x~~		x	x
FAF [O]		~~SE~~		~~SE~~		x		~~SE~~		~~SE~~		x	x
NIR[O]		~~SE~~		~~SE~~		x		~~SE~~		~~SE~~		x	x
DCFP [O]												x	x

APPENDIX D (TRACKED): COVID-19 VISIT SCHEDULE—Every-Other-Month Group Month 13 to Month 24

	Treatment												Early Term [A]
**Visit #**		15		16		17		18		19		20	
**Day**	390	420	450	480	510	540	570	600	630	660	690	720	
**Week**	52	56	60	64	68	72	76	80	84	88	92	96	
**Month**	13	14	15	16	17	18	19	20	21	22	23	24	
**Window (+ or – days)**		16		16		16		16		16		16	
**FFA [O]**												x	x
**OCT-A[S]**						~~x~~[S]						x[S]	x
**Study Eye Determination**													
**Randomization**													
**Pegcetacoplan administration or Sham Injection[T]**		x		x		x		x		x			
**Postinjection Assessment [P]**		x		x		x		x		x			
**Follow-Up Call [Q]**													
**Concomitant Medication/ Concomitant Ocular Procedures [R]**		x		x		x		x		x		x	x
**Adverse Events**		x		x		x		x		x		x	x

Abbreviations: BCVA=best corrected visual acuity; CNV=choroidal neovascularization; DCFP=digital color fundus photography; eCRF=electronic case report form; FAF=fundus autofluorescence; FFA=fundus fluorescein angiography; FRI=Functional Reading Independence index; IOP=intraocular pressure; LL BCVA= low luminance best corrected visual acuity; MNREAD=Minnesota Low-Vision Reading Test; NEI VFG-25=National Eye Institute Visual Functioning Questionnaire 25-item Version; NIR=near infrared reflectance; OCT-A=optical coherence tomography angiography; SD-OCT=spectral domain optical coherence tomography; SE=study eye; Term=termination; VEGF=vascular endothelial growth factor.

Note: All ocular assessments are to be performed for both eyes unless annotated with 'SE' (study eye) in the above schedule. All assessments should be performed on the same day. All study visits should be scheduled and projected based on the Day 1 visit date.

A. For subjects that discontinue the study early, the early termination assessments should be performed after a minimum of 30 days have passed from the last dosing visit. If a subject reports for a scheduled visit and decides to terminate early prior to dosing, then the visit should be considered the early termination visit and all early termination procedures should be performed. At Month 24, all subjects should be offered entry into an open-label study.

B. At Day 1 (Visit 2), confirm subject eligibility through reviewing the inclusion/ exclusion criteria and receive confirmation of eligibility from the reading center.

C. Significant medical/ surgical history from the previous 5 years. Anti-VEGF treatments (fellow eye) and invasive ocular procedures performed within the past 5 years and while on study should be recorded. Any history of tobacco use should be recorded.

D. Obtain prior to fluorescein angiography and before study drug administration.

E. At screening, serum pregnancy should be performed for women of childbearing potential. If positive, subject is not eligible to continue in the study.

F. Beginning at Day 1, perform the urine pregnancy test for women of childbearing potential at each treatment visit. If positive, perform a serum pregnancy test. If serum test is positive, study drug should not be administered and an early term visit should be completed.

G. Only subjects that sign the separate consent for the clinical repository will have these samples collected. A 14-mL whole-blood sample will be collected at the specified time points.

H. Blood pressure, respiratory rate, heart rate, and temperature. On dosing days, vital signs should be taken pre- dose.

I. Height and weight should be measured at screening.

J. Perform assessments prior to dilating the eyes.

K. In select countries, the MNREAD or Radner Reading Charts should be done during the study visit, prior to dilating the eyes. It should be performed monocularly first, then binocularly.

L. Microperimetry assessments will be performed post dilation. Data will be forwarded to the reading center.

M. To be administered by the masked site staff prior to any other assessments performed on that day. In-clinic assessments on the digital application should be completed after completion of all functional tests and quality of life measures prior to dilating the eyes.

N. At select sites, and for those subjects who decide to participate, the digital applications will be completed on an electronic device and will consist of assessments for visual function and reading speed. Subjects will receive training at Day 1 on the use of the digital applications and the electronic device and will perform the tests using the application in the clinic at Month 1, Month 2, Month 3, Month 6, Month 12, Month 18, and Month 24. Beginning at Day 1, subjects will complete the functional assessments weekly at home. Subjects should be instructed to complete the assessments on the same day each week and at approximately the same time of day each week, if possible.

O. FAF and SD-OCT images, near infrared reflectance, fluorescein angiograms, and fundus photographs will be performed for the study eye only on days where 'SE' is specified and for both eyes at all other visits as specified in the above schedule and will be sent to the reading center for evaluation (consult the reading center manual for specifics on image capture, processing, and transmission). Images should be captured prior to dosing on dosing days. If a subject misses a study visit or images cannot be obtained at a specific visit, study staff should make every effort to obtain images at the next scheduled visit. If new active CNV is suspected, SD-OCT, FFA, and OCT-A (selected sites) images should be collected and sent to the reading center for analysis.

P. Postinjection assessments should be performed within 5 minutes after dosing by the unmasked physician or study staff and should include a gross assessment of vision (finger-counting, hand motion, then light perception when applicable). If subject passes gross vision test, the subject may leave the site. If subject fails gross vision test, the tonometry should be performed. IOP should be ≤30 mm Hg in order for the subject to leave the site. If necessary, antiglaucomatous medication can be given to lower the IOP. If IOP is >30 mm Hg, assessments will continue every approximately 30 minutes from the previous measurement until the subject passes the gross vision test and IOP is ≤30 mm Hg. Note: if the study eye is treated with a ranibizumab or aflibercept injection during the same visit as the study treatment (pegcetacoplan or sham), the treatment with ranibizumab or aflibercept must be performed first. The pre- and post- anti-VEGF IOP values must be measured and recorded on the eCRF.

Q. Starting at Day 1, study subjects will be contacted by study site staff within 4 ± 2 days after each study treatment visit (through Month 4) to collect any information on any safety concerns, decrease in vision, eye pain, unusual ocular events, or any new ocular symptoms in the study eye. If the investigator determines that there are any safety concerns, a follow-up visit should be scheduled as soon as possible.

R. Record concomitant medications (ie, prescription and over-the-counter medications) used by the patient within 30 days of screening and throughout the subject's participation in the study.

S. In addition to the time points indicated on the study schedule, OCT-A should be performed at the time of any suspected new active CNV. If new active CNV is confirmed in the study eye, OCT-A should be repeated every 2 months for the study eye.

T. Administration of pegcetacoplan or sham can be done on separate days from the assessment visit if both days fall within the visit window. Administration of study treatment (pegcetacoplan or sham) can be done on a separate day from the assessment visit if both days fall within the visit window. If this occurs on the randomization visit, then the administration of pegcetacoplan or sham should be done within 3 days of randomization and after approval from the medical monitor. When study treatment administration is on a day other than a study visit, then the only assessment that must be done on the day of study treatment administration is preinjection IOP. During the COVID-19 pandemic, the following flexibility is allowed for IP administration: –16 days to + 30 days after medical monitor approval.

STATISTICAL ANALYSIS PLAN

APL2-304

A Phase III, Multi-Center, Randomized, Double-Masked, Sham-Controlled Study to Compare the Efficacy and Safety of Intravitreal APL-2 Therapy with Sham Injections in Patients with Geographic Atrophy (GA) Secondary to Age-Related Macular Degeneration (AMD)

PROTOCOL IDENTIFIER: OAKS

Study Sponsor:	Apellis Pharmaceuticals, Inc.<br>100 5th Ave<br>Waltham, MA 02451
Author:	Caleb Bliss
Protocol:	Amendment 5, Version 1.0, 12 August 2020
SAP Version #:	2.0
SAP Date:	10 August 2021
Status:	Final

REVISION HISTORY

Version	Issue Date	Summary of Changes
1.0	07/23/2021	New Document
2.0	08/10/2021	• Changed the thresholds for censoring COVID-19 impacted data in the supplementary analyses for the primary and key secondary endpoints.<br>• Added an additional covariance matrix to the MMRM models if the statistical modeling fails to converge with the first two covariance matrices<br>• Fixed an error in the sample code and text for maximum reading speed calculation.<br>• Fixed an error in Figure 2 with extraneous text boxes included.<br>• Provided further details on the analysis of suspected, confirmed, and exudative AMD's.

DocuSign Envelope ID: 4DCAEAF1-DCE2-4D46-BD9C-599424AFAE81

APPROVAL SIGNATURES

DocuSigned by:
Caleb Bliss
Signer Name: Caleb Bliss
Signing Reason: I approve this document
Signing Time: 10-Aug-2021 | 12:52 EDT
F3BA2A1310AF4B61B42694F4D40BCC5B

Caleb Bliss, PhD<br>Director, Biostatistics<br>Apellis Pharmaceuticals, Inc.	Date (dd-Mmm-yyyy)

DocuSigned by:
Jason Raines
Signer Name: Jason Raines
Signing Reason: I approve this document
Signing Time: 10-Aug-2021 | 12:58 EDT
DDF9F44C0B7943249C1956ECB8C87617

Jason Raines, MS, MPH<br>Vice President, Biometrics & Data Management<br>Apellis Pharmaceuticals, Inc.	Date (dd-Mmm-yyyy)

DocuSigned by:
Patrick Murphy
Signer Name: Patrick Murphy
Signing Reason: I approve this document
Signing Time: 10-Aug-2021 | 13:18 EDT
FFAFEE1C127040998DAC2D44E0D1736B

Patrick Murphy, MSc<br>Senior Director, Regulatory Affairs<br>Apellis Ireland Limited	Date (dd-Mmm-yyyy)

DocuSigned by:
Ramiro Ribeiro
Signer Name: Ramiro Ribeiro
Signing Reason: I approve this document
Signing Time: 10-Aug-2021 | 13:30 EDT
1FFAA7344980487D8986A4A6D69E6A7B

Ramiro Ribeiro, MD, PhD<br>Sr. Medical Director, Ophthalmology, Clinical Development<br>Apellis Pharmaceuticals, Inc	Date (dd-Mmm-yyyy)

DocuSign Envelope ID: 4DCAEAF1-DCE2-4D46-BD9C-599424AFAE81

DocuSigned by:

Federico Grossi

Signer Name: Federico Grossi
Signing Reason: I approve this document
Signing Time: 10-Aug-2021 | 15:29 EDT
A870049663B24A6CB18A128EEA807A6F

Federico Grossi, MD, PhD	Date (dd-Mmm-yyyy)
Chief Medical Officer	
Apellis Pharmaceuticals, Inc	

TABLE OF CONTENTS

REVISION HISTORY 2
TABLE OF CONTENTS 5
LIST OF TABLES 10
LIST OF FIGURES 10
ABBREVIATIONS 11
1. INTRODUCTION 13
2. OBJECTIVES AND ENDPOINTS 14
2.1. Objectives 14
2.1.1. Primary Objective 14
2.1.2. Key Secondary Objectives 14
2.1.3. Secondary Objectives 14
2.1.4. Safety Objectives 14
2.1.5. Exploratory Objectives 15
2.2. Endpoints 15
2.2.1. Primary Endpoint 15
2.2.2. Key Secondary Endpoints 15
2.2.3. Secondary Endpoints 15
2.2.4. Exploratory Endpoints 16
2.2.5. Safety Endpoints 17
2.2.6. Exploratory Endpoints Closely Related to the Primary Efficacy Endpoint 17
3. STUDY DESIGN 18
3.1. General Description 18
3.2. Randomization 19
3.3. Masking 19
3.4. Sample Size and Power Considerations 19
3.5. Analysis Timing and Unmasking 20
3.6. Definition of 12-Month Data in the Month 12 Dataset 21
4. STATISTICAL ANALYSIS SETS 22
4.1. Screened Set 22
4.2. Intent-to-Treat Set 22
4.3. Modified Intent-to-Treat Set 22

4.4. Safety Set 22
4.5. Per-Protocol Sets 22
4.6. Genotyping Set 23
5. STUDY SUBJECTS 24
5.1. Disposition of Subjects 24
5.2. Demographic and Other Baseline Characteristics 25
5.3. Medical ocular and non-ocular History 28
5.4. Prior and Concomitant Medications 28
5.5. Concomitant Procedures 28
5.6. Exposure to Investigational Product 29
5.7. Protocol Deviations 30
6. EFFICACY ANALYSES 32
6.1. Estimands 32
6.2. Analyses of Primary Efficacy Endpoint 36
6.2.1. Type I Error Management 36
6.2.2. Main Analysis of Primary Efficacy Endpoint 36
6.2.3. Sensitivity Analyses of Primary Efficacy Endpoint 37
6.2.3.1. Missing Data Analyses Based on Multiple Imputation 37
6.2.3.2. Other Sensitivity Analyses 39
6.2.4. Supplemental Analyses of the Primary Efficacy Endpoint 39
6.2.4.1. COVID-19 Adjusted Estimand 39
6.2.4.2. Per Protocol Set 40
6.2.4.3. Rate of Change Analyses 40
6.2.5. Subgroup Analyses of Primary Efficacy Endpoint 41
6.2.6. Full 24 Month Analyses 42
6.2.7. Exploratory Endpoints Closely Related to the Primary Efficacy Endpoint 42
6.2.7.1. Change in Square Root of GA Area 42
6.2.7.2. Percent Change in GA Area and Square Root of GA Area 43
6.3. Analyses of Key Secondary Efficacy Endpoints 43
6.3.1. Type I Error Management 43
6.3.2. Main Analyses of Key Secondary Efficacy Endpoints 47
6.3.2.1. Mean Threshold Sensitivity of All Points 47
6.3.2.2. Monocular Maximum Reading Speed 47

6.3.2.3. Mean FRI Index Score....48
6.3.2.4. NL-BCVA Score....49
6.3.3. Sensitivity Analyses of Key Secondary Efficacy Endpoints....49
6.3.4. Supplementary Analyses of Key Secondary Efficacy Endpoints....50
6.3.5. Subgroup Analyses of Key Secondary Efficacy Endpoints....50
6.4. Analyses of Other Secondary Efficacy Endpoints....50
6.4.1. Low Luminance Best-Corrected Visual Acuity (LL-BCVA)....51
6.4.2. Total Area of GA lesion(s) at each planned assessment....51
6.4.3. Monocular critical print size....51
6.4.4. NEI VFQ-25 Distance Activity Subscale Score....52
6.4.5. Macular Functional Response based on Microperimetry....52
6.5. Multiplicity Adjustment....53
6.6. Analyses of Exploratory Endpoints....53
6.6.1. Other Microperimetry Endpoints....53
6.6.2. NEI VFQ-25 and NEI VFQ-39 Additional Analyses....54
6.6.3. Binocular Maximum Reading Speed and Critical Print Size....54
6.6.4. GA Lesion (s) Study Eye to Fellow Eye Comparisons....55
6.6.5. Digital Reading Index and Visual Function Application....55
6.6.6. Genetic Polymorphisms....55
6.6.7. Dry AMD Analysis....56
6.6.8. Analyses of Other Ophthalmology Efficacy Imaging Data....56
7. SAFETY ANALYSES....57
7.1. Adverse Events....57
7.1.1. TEAEs of New Onset Exudative AMD....58
7.2. Clinical Laboratory Data....59
7.3. Vital Signs....61
7.4. Physical Examination Findings....61
7.5. Complete Ophthalmic Exam....61
7.6. NL-BCVA....62
7.7. Other Ophthalmology Imaging Assessments....62
7.8. Immunogenicity....64
7.9. Death....64
7.10. Other Safety Data....64

8. PHARMACOKINETICS ANALYSIS 65
9. PHARMACODYNAMIC ANALYSES 66
10. OTHER ANALYSES 67
11. INTERIM ANALYSIS 68
12. DATA MONITORING COMMITTEE 69
13. DATA HANDLING CONVENTIONS 70
13.1. General Data Reporting Conventions 70
13.2. Definition of Baseline 70
13.3. Definition of Relative Study Days 70
13.4. Definition of Visit Windows 70
13.4.1. All Assessments Except Microperimetry 70
13.4.2. Microperimetry Assessments 72
13.5. Derived Efficacy Endpoints 73
13.5.1. GA Lesion Size 73
13.5.2. MNRead Reading Chart 73
13.5.2.1. Reading Speed 73
13.5.2.2. Maximum reading speed 73
13.5.2.3. Critical Print Size 73
13.5.2.4. Reading acuity 74
13.5.3. Radner Reading Chart 74
13.5.3.1. Reading Speed 74
13.5.3.2. Maximum Reading Speed 74
13.5.3.3. Critical Print Size 74
13.5.3.4. Reading Acuity 75
13.5.4. Mean FRI Index Score and FRI Level 75
13.5.5. NL-BCVA and LL-BCVA 75
13.5.5.1. BCVA Letter Score 75
13.5.5.2. Conversion of BCVA Letter Score to Snellen Equivalent 75
13.5.6. Low Luminance Deficit 75
13.5.7. NEI VFQ-25 scores 76
13.5.7.1. NEI VFQ-25 Distance Activity Subscale Score 76
13.5.7.2. NEI VFQ-25 Near Activity Subscale Score 76
13.5.7.3. NEI VFQ-25 Driving Subscale Score 76

13.5.7.4. NEI VFQ-25 Composite Scores 76
13.5.8. NEI VFQ-39 Scores 76
13.5.8.1. NEI VFQ-39 Distance Activity Subscale Score 76
13.5.8.2. NEI VFQ-39 Near Activity Subscale Score 76
13.5.8.3. NEI VFQ-39 Composite score 76
13.5.9. Microperimetry 77
13.6. Repeated or Unscheduled Assessments of Safety Parameters 77
13.7. Handling of Missing, Unused, and Spurious Data 77
13.7.1. Missing Date of Investigational Product 77
13.7.2. Missing Date Information for Prior or Concomitant Medications (Therapies/Procedures) 77
13.7.2.1. Incomplete Start Date 77
13.7.2.2. Incomplete Stop Date 78
13.7.2.3. Missing Day and Month 78
13.7.3. Missing Date Information for Adverse Events 79
13.7.3.1. Incomplete Start Date 79
13.7.3.2. Incomplete Stop Date 79
13.7.4. Missing Severity Assessment for Adverse Events 79
13.7.5. Missing Relationship to Investigational Product for Adverse Events 79
13.7.6. Character Values of Clinical Laboratory Variables 79
14. ANALYSIS SOFTWARE 81
15. CHANGES TO ANALYSIS SPECIFIED IN PROTOCOL 82
16. REFERENCES 83
17. APPENDIX 84
APPENDIX 1. SCHEDULE OF ACTIVITIES 85
1.1. Protocol Changes to be followed during COVID-19 Restrictions 98
1.2. Sample of SAS Code 113
1.2.1. Mixed Effect Model for Repeated Measure 113
1.2.2. Multiple Imputation 114
1.2.3. Rate of Change Models 117
1.2.4. Sample Code for Maximum Reading Speed and Critical Print Size 120

LIST OF TABLES

Table 1: Power to Detect a Difference among Three Groups with an Equal Size of 200 Subjects 20
Table 2: Estimands of Study 34
Table 3: Criteria for Potentially Clinically Significant Laboratory Tests 60
Table 4: Criteria for Potentially Clinically Significant Vital Signs 61
Table 5: Post-Baseline Analysis Visit Window for Unscheduled and Early Termination Visits: All Assessments Except Microperimetry: Monthly Regimen 71
Table 6: Post-Baseline Analysis Visit Window for Unscheduled and Early Termination Visits: All Assessments Except Microperimetry: Every Other Month Regimen 72
Table 7: Post-Baseline Analysis Visit Window for Unscheduled and Early Termination Visits: Microperimetry Assessments 72
Table 8: Examples for Coding of Special Character Values for Clinical Laboratory Variables 80
Table 9: Visit Schedule - Monthly Group- Screening, Day 1 through Month 12 86
Table 10: Visit Schedule - Monthly Group- Month 13 to Month 24 89
Table 11: Visit Schedule - Every-Other-Month Group Screening, Day 1 Through Month 12 92
Table 12: Visit Schedule - Every-Other-Month Group Month 13 to Month 24 95
Table 13 (TRACKED): COVID-19 VISIT SCHEDULE—Monthly Group—Screening, Day 1 Through Month 12 101
Table 14 (TRACKED): COVID-19 VISIT SCHEDULE—Monthly Group—Month 13 to Month 24 104
Table 15 (TRACKED): COVID-19 VISIT SCHEDULE—Every-Other-Month Group—Screening, Day 1 Through Month 12 107
Table 16 (TRACKED): COVID-19 VISIT SCHEDULE—Every-Other-Month Group—Month 13 to Month 24 110

LIST OF FIGURES

Figure 1: Study Schema 18
Figure 2: APL2-303 and APL2-304 Hypothesis Testing and Type I Error Control 45
Figure 3: APL2-304 Hypothesis Testing and Type I Error Control If There is No α Remaining in APL2-303 to Allow Pooling Secondary Functional Endpoints 46

ABBREVIATIONS

ADA	Anti-drug Antibodies
ADY	Analysis Study Day
AE	Adverse Event
ALP	Alkaline Phosphatase
ALT	Alanine Aminotransferase
AMD	Age-related Macular Degeneration
AST	Aspartate Aminotransferase
ATC	Anatomical Therapeutic Class
BCVA/NL-BCVA	(Normal luminance) Best Corrected Visual Acuity
BCEA	Bivariate contour ellipse area
BUN	Blood Urea Nitrogen
CMH	Cochran Mantel Haenszel
CNV	Choroidal Neovascularization
CRO	Contract Research Organization
cRORA	Complete retinal pigment epithelium and outer retinal atrophy
CSR	Clinical Study Report
DA	Disk Areas
DCFP	Digital Color Fundus Photography
DLS	Double-layer sign
DMC	Data Monitoring Committee
eCRF	Electronic Case Report Form
EOM	Every Other Month
ETDRS	Early Treatment Diabetic Retinopathy Study
FAF	Fundus Autofluorescence
FDA	Food and Drug Administration
FFA/FA	Fundus Fluorescein Angiography or Fluorescein Angiography
FRI	Functional Reading Independence
GA	Geographic Atrophy
IOP	Intra Ocular Pressure
iRORA	Incomplete retinal pigment epithelium and outer retinal atrophy
ITT	Intent-to-Treat
IVT	Intravitreal
kg	Kilogram
LOV	Last Observed Value
LL-BCVA	Low Luminance Best Corrected Visual Acuity
LLD	Low Luminance Deficit
LS	Least Square
MAR	Missing at Random
MCMC	Markov Chain Monte Carlo
MedDRA	Medical Dictionary for Regulatory Activities
mg	Milligram
mITT	Modified Intention to Treat
mL	Milliliter
mmHg	Millimeter of Mercury
MMRM	Mixed effect Model for Repeated Measure

MNAR	Missing Not At Random
MNRead	Minnesota Reading Chart
NEI VFQ-25	National Eye Institute Visual Functioning Questionnaire 25 Item Version
NEI VFQ-39	National Eye Institute Visual Functioning Questionnaire 39 Item Version
NIR	Near Infrared Reflectance
OCT	Optical Coherence Tomography
OCT-A	Optical Coherence Tomography Angiography
PCS	Potentially Clinically Significant
PDV	Protocol Deviation
PEG	Polyethylene glycol
PEOM	Pegcetacoplan Every-Other-Month
PM	Pegcetacoplan Monthly
PP	Per Protocol
PT	Preferred Term
RBC	Red Blood Cell
SAE	Serious Adverse Event
SAP	Statistical Analysis Plan
SAS	Statistical Analysis System
SD-OCT	Spectral Domain Optical Coherence Tomography
SEOM	Sham Every-Other-Month
SI	International System of Units
SM	Sham Monthly
SNP	Single Nucleotide Polymorphism
SOC	System Organ Class
TEAE	Treatment Emergent Adverse Event
µL	Micro liter
WBC	White Blood Cell
WHO	World Health Organization
WPM	Words per Minute

1. INTRODUCTION

This study is being conducted as part of a series of studies for the clinical development of pegcetacoplan (also known as APL-2) for geographic atrophy [GA] secondary to age-related macular degeneration (AMD). This statistical analysis plan (SAP) provides a technical and detailed elaboration of the statistical analyses of efficacy and safety data supplementing what is described in the final study protocol, version 1.0, amendment 5, dated 12 August 2020.

The primary study analysis will be based on complete data from the first 12 months of the study and performed after all subjects have completed the Month 12 visit in the study or discontinued early, and all corresponding data have been entered into the database, reviewed, cleaned, and finalized as the Month 12 dataset. Analysis will include testing of hypotheses of the primary efficacy endpoint at Month 12. Key secondary, secondary, and exploratory endpoints will be evaluated based on data in the Month 12 datasets in a descriptive manner. An analysis of the safety data in the Month 12 dataset will also be performed.

The final study analysis will be based on data after all subjects have either completed the Month 24 visit in the study or discontinued the study early, and all data from the study are in the database and the database is reviewed, cleaned, and locked as the final dataset. Analysis will include testing of hypotheses of the key secondary endpoints at Month 24 as described in Section 6.3.1. A cumulative analysis of all data through the end of the study will be produced based on the final dataset.

See Section 3.5 for further details on analysis timing.

The analyses specified in this document supersede the analysis plan described in the study protocol (protocol version 1.0, amendment 5 dated 12 August 2020).

2. OBJECTIVES AND ENDPOINTS

2.1. Objectives

2.1.1. Primary Objective

The primary objective of this study is to evaluate the efficacy of pegcetacoplan compared to sham in subjects with GA secondary to AMD assessed by change in the total area of GA lesions in the study eye from baseline as measured by Fundus Autofluorescence (FAF).

2.1.2. Key Secondary Objectives

The key secondary objectives are to evaluate the efficacy of pegcetacoplan compared to sham in subjects with GA secondary to AMD with respect to:

- Monocular maximum reading speed (study eye), as assessed by Minnesota Reading (MNRead) or Radner Reading Charts (in select countries)
- Functional Reading Independence (FRI) Index score (subject-level assessment)
- Normal luminance best-corrected visual acuity (NL-BCVA) score in the study eye

2.1.3. Secondary Objectives

To evaluate the efficacy of pegcetacoplan compared to sham in subjects with GA secondary to AMD with respect to:

- Low luminance best corrected visual acuity (LL-BCVA) score in the study eye
- Low luminance deficit (LLD) in the study eye
- Total area of GA lesion(s) in the study eye
- Monocular critical print size (study eye), as assessed by MNRead or Radner Reading Charts (in select countries)
- National Eye Institute Visual Functioning Questionnaire 25 Item Version (NEI VFQ-25) distance activity subscale score (in select countries)
- Macular functional response (study eye) as assessed by mesopic microperimetry

2.1.4. Safety Objectives

To evaluate the safety and tolerability of pegcetacoplan compared to sham injection in subjects with GA secondary to AMD as indicated by:

- Incidence and severity of ocular and systemic treatment-emergent adverse events (TEAEs)
- Incidence of anti-drug antibodies (ADA) directed against pegcetacoplan peptide or polyethylene glycol (PEG)
- Incidence of new active choroidal neovascularization (CNV) in the study eye

2.1.5. Exploratory Objectives

- To evaluate the efficacy of pegcetacoplan compared to sham in subjects with GA secondary to AMD as indicated by:
    - NEI VFQ-25 composite score
    - NEI VFQ-25 near activity subscale score (in select countries)
    - Comparison between study eye and fellow eye of change in GA lesion size
    - To evaluate the binocular reading speed as assessed by MNRead or Radner Reading Charts (in select countries)
    - To evaluate the binocular critical print size as assessed by MNRead or Radner Reading Charts (in select countries)
    - To evaluate the relationship between genetic polymorphisms associated with AMD with GA progression and response to pegcetacoplan
- To evaluate the incidence of new onset of subclinical CNV in the study eye.
- To assess sensitivity and specificity of a digital reading speed application to detect disease progression / regression (optional, select sites)
- To assess sensitivity and specificity of a digital visual function application to detect disease progression / regression (optional, select sites)

2.2. Endpoints

2.2.1. Primary Endpoint

The primary endpoint is the change from baseline to Month 12 in total area of GA lesion(s) in the study eye (in mm^2) based on FAF.

2.2.2. Key Secondary Endpoints

The key secondary endpoints include:

- Change from baseline in the mean threshold sensitivity of all points (study eye) assessed by mesopic microperimetry at Month 24.
- Change from baseline in monocular maximum reading speed (study eye), at Month 24 as assessed by MNRead or Radner reading charts (in select countries).
- Change from baseline in mean FRI Index score (subject-level assessment) at Month 24
- Change from baseline in NL-BCVA score (study eye) at Month 24 as assessed by Early Treatment Diabetic Retinopathy Study (ETDRS) chart.

2.2.3. Secondary Endpoints

Other secondary endpoints include:

- Change from baseline in LL-BCVA score (study eye) over time as assessed by ETDRS chart.
- Change from baseline in the total area of GA lesion(s) in the study eye (in mm^2) as assessed by FAF over time other than Month 12.
- Change from baseline in monocular critical print size (study eye), as assessed by MNRead or Radner reading charts (in select countries) over time.
- Change from baseline in the NEI VFQ-25 distance activity subscale score (in select sites) (subject level assessment) over time.
- Change from baseline in the number of scotomatous points assessed by mesopic microperimetry over time
- Change from baseline in the mean threshold sensitivity within 500 microns outside the GA lesion, (perilesional points) assessed by mesopic microperimetry over time.

2.2.4. Exploratory Endpoints

- Additional microperimetry endpoints including:
  - Change from baseline in fixation stability (95% bivariate contour ellipse area (BCEA)) over time.
  - Change from baseline in the mean threshold sensitivity of points beyond 500 microns outside the atrophy border (paralesional points) over time.
  - Change from baseline in the mean threshold sensitivity of points beyond the GA lesion (extralesional points) over time.
  - Number of points with clinically significant progression over time, defined as a decrease of more than 4 dB.
- Additional NEI-VFQ endpoints including:
  - Change from baseline in the NEI VFQ-39 distance activity subscale score (in select sites) over time.
  - Change from baseline in NEI VFQ-25 composite score over time.
  - Change from baseline in NEI VFQ-39 composite score over time.
  - Change from baseline in NEI VFQ-25 near activity subscale score (in select countries) over time.
  - Change from baseline in NEI VFQ-39 near activity subscale score (in select countries) over time.
  - Change from baseline in NEI VFQ-25 driving subscale score (in select countries) over time as well as the number and percentage of subjects with worse driving outcomes for subjects who are currently driving at baseline.
- Difference between study eye and fellow eye in change in GA lesion size from baseline over time in subjects with bilateral GA.

- Change from baseline in binocular maximum reading speed as assessed by MNRead or Radner Reading Charts (in select countries) over time.
- Change from baseline in binocular critical print size as assessed by MNRead or Radner Reading Charts (in select countries) over time.
- Change from baseline in digital reading index (in select sites) over time as measured by the Spotlight instrument.
- Change from baseline in digital visual function (metamorphopsia) (in select sites) over time as measured by the Alleye instrument.
- Progression from incomplete retinal pigment epithelium and outer retinal atrophy (iRORA) to complete retinal pigment epithelium and outer retinal atrophy (cRORA).
- Progression from large drusen to iRORA or cRORA.
- Other efficacy imaging endpoints including change in the distance of the atrophy junction to the fovea, and non-subfoveal atrophy to subfoveal atrophy conversion.

2.2.5. Safety Endpoints

- Incidence and severity of ocular and systemic treatment-emergent adverse events.
- Incidence of ADA directed against pegcetacoplan peptide or PEG.
- Incidence of new active CNV in the study eye.
- Incidence of new onset of subclinical CNV in the study eye.
- Incidence of subjects who lost letters based on NL-BCVA categories (≥15, ≥15 -< 30, ≥30 ETDRS letters).
- Change/shift from baseline in clinical labs and incidence of abnormal lab values.
- Change from baseline in vital signs and incidence of abnormal vital sign results.
- Shift from baseline in ocular examination assessments including slit-lamp examination and indirect ophthalmoscopy.
- Change from baseline in Intra Ocular Pressure (IOP) and incidence of IOP above specified thresholds.
- Change/shift from baseline in ocular imaging assessments (including specular microscopy; select sites).

2.2.6. Exploratory Endpoints Closely Related to the Primary Efficacy Endpoint

- Change from baseline in square root of the total area of GA lesion(s) in the study eye (in mm) based on FAF over time.
- Percent change in total area of GA lesion(s) in the study eye (in mm^2) based on FAF over time.
- Percent change in square root of the total area of GA lesion(s) in the study eye (in mm) based on FAF over time.

3. STUDY DESIGN

3.1. General Description

This is a Phase 3, multicenter, randomized, double-masked, sham-injection controlled study to assess the efficacy and safety of multiple intravitreal (IVT) injections of pegcetacoplan (also known as APL-2) in subjects with GA secondary to AMD.

The study will randomize approximately 600 subjects across approximately 100 multinational sites. Subjects will be screened within 28 days before receiving pegcetacoplan or Sham injection. Upon providing written informed consent and entry into the study, subjects will be assigned a screening number. Subjects who meet all inclusion and none of the exclusion criteria will return to the clinic for randomization and treatment on Visit 2 (Day 1). At this visit, subjects will be randomized 2:2:1:1 to receive pegcetacoplan Monthly (PM), pegcetacoplan Every-Other-Month (PEOM), Sham injection Monthly (SM) or Sham-injection Every-Other-Month (SEOM), respectively. Randomization will be stratified according to GA lesion area at screening (< 7.5 mm^2; ≥ 7.5 mm^2), and presence of CNV in the fellow eye.

The planned length of participation in the study for each subject is approximately 24 months (from the beginning of the screening period through Month 24 visit). After the COVID-19 pandemic started, Apellis added a rescreening procedure where the screening period was extended to up to 90 days (applicable only to subjects that fully qualified for the trial during a complete screening prior to March 30, 2020). The total length of participation in this study can be up to 27 months for subjects that were screened using this updated procedure. The schedules of assessments are presented in Appendices A, B, C, and D of Study Protocol Amendment 5, Version 1.0 dated 12 August 2020 and are also available in Appendix 1.

A study schematic diagram is shown in Figure 1.

Figure 1: Study Schema

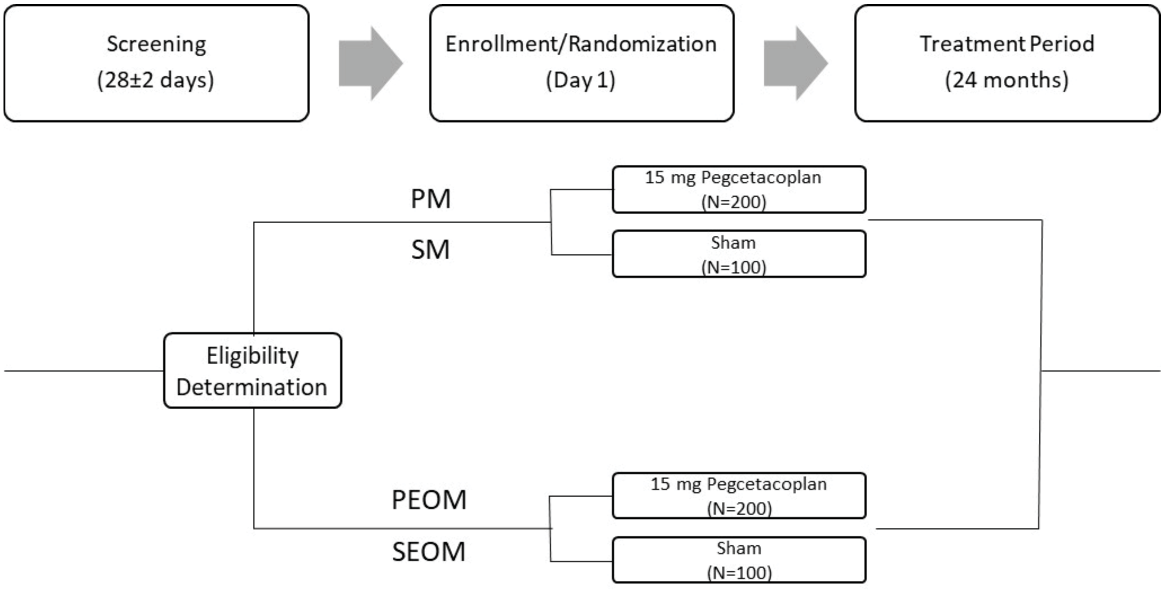

PM= Pegcetacoplan Monthly; SM= Sham Monthly; PEOM= Pegcetacoplan Every-Other-Month; SEOM= Sham Every-Other-Month

3.2. Randomization

Subjects will be randomized 2:2:1:1 using a web-based randomization system to receive treatment with PM, PEOM, SM or SEOM, respectively.

The randomization scheme will be maintained by the Sponsor, or designee. Subject randomization will be stratified by GA lesion area at screening based on assessment from the reading center (< 7.5 mm^2; ≥ 7.5 mm^2) and presence of CNV in the fellow eye (yes; no).

3.3. Masking

This is a double-masked study. Designated masked study site staff (e.g., assistant(s), VA technicians, optical coherence tomography (OCT) technicians, photographers, technicians administering questionnaires, subjects, Reading Center personnel, the assigned evaluating physician(s), and the Sponsor) will be masked to treatment assignment. However, the treating physician and any associated support staff involved in performing the intravitreal or sham injections will be unmasked to study treatment. These individuals are only responsible for administering the study drug and are not involved in assessing adverse events. In addition, the unmasked individuals are not allowed to discuss treatment and/or subject outcomes with masked study staff, including the evaluating physician. The Principal Investigator must be masked to subjects' treatment assignment. To prevent bias in treatment assignment, eligible subjects will be randomized using a web-based randomization system. Processes and plans will be put in place to avoid unintentional unmasking during the study. All study roles will be clearly documented on the site delegation of authority log and once the roles have been designated and executed, these roles should not be switched during the conduct of the study. In unforeseen circumstances, a site can contact the Sponsor to switch a study staff member from the masked role to the unmasked role but not vice versa. Details on unmasking at the Month 12 reporting are in Section 3.5.

While the actual study treatment (pegcetacoplan vs. sham) is masked, the treatment frequency (monthly vs. every other month) for each individual subject is known.

3.4. Sample Size and Power Considerations

A total of approximately 600 subjects will be randomized in a 2:2:1:1 ratio to receive treatment with PM, PEOM, SM, or SEOM. The annual growth rate in GA lesion area is expected to have a mean of 1.47, 1.70 and 2.13 mm^2/year for PM, PEOM and Sham-Pooled (Sham) groups, respectively, as estimated from the results of a Phase 2 trial of pegcetacoplan. The standard deviation of the lesion growth is estimated to be 1.50 mm^2 based on the same Phase 2 trial data or 1.25 mm^2 based on natural history data (Holekamp N, 2019).

The power of the study for the primary endpoint is presented in Table 1 for sample size of 200 in each treatment arm. The approximation is calculated using PROC POWER ONEWAYANOVA, Statistical Analysis System (SAS) 9.4. The study power for the primary endpoint is likely larger when utilizing the longitudinal data to model the primary endpoint. The actual study power for the primary endpoint may also vary based on the distribution of the stratification factors (i.e., lesion area at screening, presence of CNV in fellow eye). Study power was not calculated for key secondary functional endpoints.

Table 1: Power to Detect a Difference among Three Groups with an Equal Size of 200 Subjects

Common Standard Deviation (mm^2)	Alpha (two-sided)	Power for a true mean of 1.47, 1.70, and 2.13 mm^2/year for PM, PEOM and Sham, respectively		
		PM vs Sham	PEOM vs Sham	Overall (Among 3 groups)
1.25	0.0495	> 99.9%	92.9%	99.9%
1.25	0.0248	99.9%	88.2%	99.7%
1.40	0.0495	99.7%	86.5%	99.3%
1.40	0.0248	99.3%	79.4%	98.6%
1.50	0.0495	99.2%	81.5%	98.4%
1.50	0.0248	98.4%	73.1%	97.0%

PM = pegcetacoplan Monthly, PEOM = pegcetacoplan Every-Other-Month; Sham = Sham Monthly + Sham Every-Other-Month

3.5. Analysis Timing and Unmasking

The analysis of data from the first 12 months of the study will be performed when all subjects have completed the Month 12 visit in the study or discontinued early and all corresponding data have been entered into the database, reviewed, cleaned, and finalized as the Month 12 dataset per the 12-Month Primary Analysis Data Cut Plan and the Programming Plan. At the time of the analysis of the Month 12 dataset, subjects who have not completed or discontinued from the study will still be followed by the sites as part of the second year.

At the time of the Month 12 reporting, Sponsor personnel who are analyzing, interpreting, and reporting data from the Month 12 dataset will be unmasked to treatment assignment. To maintain data integrity for the remainder of the study, sponsor and contract research organization (CRO) personnel responsible for continuing study oversight will remain masked as documented in the Study Masking Plan and the GA Internal Unmasking Plan.

Analyses will include formal hypothesis testing of the primary efficacy endpoint at Month 12. Key secondary, secondary, and exploratory endpoints will be evaluated based on the Month 12 dataset in a descriptive manner. An analysis of the safety data in the Month 12 dataset will also be performed.

The final study reporting will be based on data after all subjects have either completed the Month 24 visit in the study or discontinued the study early, and all data from the study are entered in the database and the database is reviewed, cleaned, and locked as the final dataset. Minor changes are expected from the Month 12 dataset (e.g. adverse event end dates for events ongoing in the Month 12 dataset), however, all subsequent differences in data prior to the Month 12 visit will be listed. Analysis of the final dataset will include testing of hypotheses of the key secondary endpoints at Month 24 as described in Section 6.3.1. A cumulative analysis of all data through the end of the study will be produced based on the final dataset.

Aggregate results of the Month 12 reporting may be reported before completion of the study. However, subjects, masked study site personnel, masked CRO personnel at Covance, Sponsor team members with study oversight responsibility, and central reading center personnel will remain masked to individual treatment assignments until after the study is completed

(after all subjects have either completed Month 24 or discontinued early from the study), the database is locked and the unmasking has been approved.

3.6. Definition of 12-Month Data in the Month 12 Dataset

All screening and post-baseline data with a clinical date (i.e., administration/assessment/onset/start date) on or before the defined Month 12 data cutoff date will be included in the Month 12 Dataset. The 12-month data cutoff will include all data regardless of the type of study visit at which it was collected. This may include data collected at unscheduled visits or early termination visits if the visit date was on or before the 12-month data cutoff date. The Month 12 data cutoff is defined as:

<u>All data except microperimetry</u>

- If the subject completed Month 12 (Visit 14) assessments, then the data cutoff will be the day that the Month 12 (Visit 14) assessments are completed, regardless of Study Day.
- If the subject did not complete the Month 12 (Visit 14) assessments (e.g. missed or early terminated) then Study Day 375 (Study Day 360 (target date) + 15) will be used as the Month 12 cutoff.

<u>Microperimetry</u>

- Study Day 450 (Study Day 360 (target date) + 90) will be used as the Month 12 cutoff. This is to allow any unscheduled assessments including repeated microperimetry assessments collected as of the Month 12 datacut date to be considered for the Month 12 analysis visit according to the analysis visit windowing algorithm as specified in Section 13.4.2

4. STATISTICAL ANALYSIS SETS

Two sham treatment arms (SM and SEOM) will be pooled into a single control (Sham) group for analyses.

4.1. Screened Set

The screened set consists of all subjects who provided written informed consent and are screened for participation in this study. This population will only be used for the purposes of describing the subject disposition and for listing the data.

4.2. Intent-to-Treat Set

The Intent-to-treat (ITT) set consists of all randomized subjects. Subjects will be analyzed in the treatment arm assigned at randomization.

4.3. Modified Intent-to-Treat Set

The modified ITT (mITT) set consists of all randomized subjects who receive at least one injection of pegcetacoplan or sham and have baseline and at least one post-baseline value of GA lesion area in the study eye as assessed by FAF. Subjects will be analyzed in the treatment arm assigned at randomization.

4.4. Safety Set

The Safety set consists of all subjects randomized who receive at least one injection of pegcetacoplan or sham. Subjects will be analyzed according to the actual treatment received. In the case a subject received an incorrect injection of study medication than what they were randomized to, subjects will be presented under the corresponding pegcetacoplan arm if they received at least one injection of pegcetacoplan during the study and will only be presented under the corresponding sham arm if they did not receive any injections of pegcetacoplan. This population will be used for all safety analyses.

4.5. Per-Protocol Sets

The Per-Protocol (PP) sets will be identified separately for Month 12 and Month 24 analysis, respectively (i.e., Month 12 PP set and Month 24 PP set). The PP sets consist of all mITT subjects who have a valid GA lesion area assessment for either Month 10 or 12 (Month 12 PP set) or a valid GA lesion area assessment for at least one of Month 18, 20, 22, 24 (Month 24 PP set) and who follow the protocol without any major deviation(s) that could affect the primary efficacy data.

A valid GA lesion area assessment is defined as a non-missing measured GA lesion area assessment at a given timepoint where at least 75% of the expected injections over the course of participation ahead of the given timepoint have been received by the subject. For example:

- A valid GA lesion measurement at Month 10 in the Monthly arm would be a result available at Month 10 with no more than 2 missed injections prior to the Month 10 assessment (10 scheduled before Month 10, 2 missed = 80% compliance).

- A valid GA lesion measurement at Month 20 in the every other month (EOM) arm would be a result available at Month 20 with no more than 2 missing injections prior to the Month 20 assessment (10 scheduled before Month 20, 2 missed = 80% compliance).

Major protocol deviations (PDVs) that lead to exclusion from the Month 12 PP set and the Month 24 PP set are as follows:

(i) Violations of inclusion and/or exclusion criteria

A PDV with any of the following inclusion and/or exclusion criteria violations will be classified as a major PDV:

Inclusion Criterion #3: Clinical diagnosis of GA of the macula secondary to AMD as determined by the Investigator and confirmed by the Reading Center

Inclusion Criterion #4: The GA lesion must meet the criteria listed in the protocol as determined by the central Reading Center's assessment of FAF

- Total GA area must be ≥ 2.5 and ≤ 17.5 mm^2 (1 and 7 disk areas [DA] respectively).
- If GA is multifocal, at least one focal lesion must be ≥ 1.25 mm^2 (0.5 DA), with the overall aggregate area of GA as specified above in 4a.
- The entire GA lesion must be completely visualized on the macula centered image and must be able to be imaged in its entirety and not contiguous with any areas of peripapillary atrophy.
- Presence of any pattern of hyperautofluorescence in the junctional zone of GA. Absence of hyperautofluorescence (i.e., pattern = none) is exclusionary.

Exclusion Criterion #1: GA secondary to a condition other than AMD such as Stargardt disease, cone rod dystrophy or toxic maculopathies like plaquenil maculopathy in either eye.

Exclusion Criterion #3: Any history or active choroidal neovascularization (CNV), associated with AMD or any other cause, including any evidence of retinal pigment epithelium rips or evidence of neovascularization anywhere based on spectral domain optical coherence tomography (SD-OCT) imaging and/or fluorescein angiography as assessed by the Reading Center.

Exclusion Criterion #13: Prior participation in another interventional clinical study for geographic atrophy in either eye including investigational oral medication and placebo.

(ii) Not receiving assigned treatment

Not receiving assigned treatment at more than 25% of the treatment visits over the analysis timeframe, either 12-month or 24-month, will be classified as a major PDV.

4.6. Genotyping Set

The genotyping set consists of all ITT subjects who have at least one non-missing genotyping result for a single nucleotide polymorphism (SNP) associated with age-related macular degeneration from the genotype sequencing analysis.

5. STUDY SUBJECTS

5.1. Disposition of Subjects

The number of subjects screened, passed screening, screened failed, and the reasons for screen failure will be presented overall.

The number of subjects randomized and in each defined analysis set will be summarized by treatment group and overall for the ITT Set. In addition, the reasons for exclusion from the mITT and per-protocol populations will be summarized.

Subjects' disposition by region and country will also be provided for the ITT set. All summaries on subject disposition will be produced based on the Month 12 dataset for the Month 12 reporting and based on the final study dataset for the final reporting, as appropriate.

Overall summary of subjects' disposition includes:

- Number of subjects randomized
- Number of subjects completed treatment [through Month 12 or Month 24, as appropriate]
- Number of subjects discontinued from treatment and reason for discontinuation [prior to Month 12 or Month 24 as appropriate]
  - Subjects who discontinued from treatment without concurrent study discontinuation will be singled out [prior to Month 12 or Month 24 as appropriate]
- Number of subjects completed study [through Month 12 or Month 24, as appropriate]
- Number of subjects discontinued from study and reason for discontinuation [prior to Month 12 or Month 24 as appropriate]

A summary of the disposition through each study visit will also be presented by treatment group and overall for the ITT set.

In addition, to assess the impact of COVID-19 pandemic on disposition of subjects, the following summary will be provided for each study visit and by reason (COVID-19 vs. non-COVID-19) for the ITT set:

- Number of subjects discontinued from treatment
- Number of subjects discontinued from study

5.2. Demographic and Other Baseline Characteristics

Demographic and baseline characteristics will be tabulated using descriptive statistics by treatment group and overall. Tables will be produced for the following analysis sets: ITT, Safety, mITT, Month 12 PP and Month 24 PP (as appropriate), and Genotyping. The following variables will be included in the tables:

The demographic data are:

- Age at screening (years) and in categories
  - o < 65
  - o 65-<75
  - o 75-<85
  - o ≥ 85
- Sex
- Race
- Ethnicity
- Geographic Region (United States vs. Rest of World)
- Country
- Weight (kg) at baseline
- Height (cm) at baseline
- Body Mass Index (kg/m^2)
- Tobacco use status (ever, never)

Baseline characteristics will be summarized for both study eye and fellow eye whenever applicable. Baseline characteristics include:

- Study eye laterality (OD, OS)
- Study eye status (better-seeing eye vs. worse-seeing eye, based on NL-BCVA – in the case of ties, study eye status will be classified as the better-seeing eye)
- GA lesion size (mm^2) (assessed by FAF)
- GA lesion size in categories, determined by reading center (as randomized status and actual status):
  - o < 7.5 mm^2
  - o ≥ 7.5 mm^2
- GA lesion size categories (approximately tertiles based on APL2-303/304 combined data*) – study eye only
- Square root GA lesion size (mm)

- GA lesion location (subfoveal involvement vs. non-subfoveal involvement) (assessed by FAF)
- GA focality (unifocal vs. multifocal) (assessed by FAF)
- Number of areas of atrophy (none, 1, 2-5, 6-10, 11-20, >20, cannot be determined) (assessed from FAF)
- Presence of double-layer sign (DLS) (assessed by SD-OCT)
- Central subfield thickness (CST) (assessed by SD-OCT)
- Presence of macular neovascularization (MNV) (i.e., subclinical CNV) (assessed by Optical Coherence Tomography Angiography (OCT-A))
- Presence of pseudodrusen (assessed by near-infrared reflectance imaging)
- Number of intermediate/large drusen (0-5, 6-10, 11-20, and >20) (assessed by color fundus photography)
- NL-BCVA in ETDRS letters
- NL-BCVA categories (≥70, ≥60 - <70, ≥35 - <60, <35 ETDRS letters)
- NL-BCVA (<60 vs. ≥60 ETDRS letters)
- LL-BCVA in ETDRS letters
- LL-BCVA categories (approximately tertiles based on APL2-303/304 combined data*) – study eye only
- IOP
- LLD in ETDRS letters
- LLD categories (<20 vs. ≥ 20 ETDRS letters)
- LLD categories (approximately tertiles based on the APL2-303/304 combined data*) – study eye only
- NL-BCVA (<60 vs. ≥60 ETDRS letters) and LLD (<20 vs. ≥ 20 ETDRS letters) combinations
- Advanced AMD (fellow eye with: GA only, GA with CNV, CNV only, neither GA nor CNV)
- GA laterality (bilateral GA vs. study eye GA Only)
- Presence of CNV in fellow eye (as randomized and actual status (based on medical history))
- Lens status:
    - Phakic
    - Pseudophakic
- Reading speed assessment method

- Monocular and binocular maximum reading speed and critical print size (without adjustment for reading inaccuracy)
- Monocular maximum reading speed categories (<60, $60-<160$, ≥ 160 words per minute (wpm))
- Monocular maximum reading speed categories (approximately tertiles based on APL2-303/304 combined data*) – study eye only
- Mean FRI Index score
- FRI Level (1,2,3,4)
- Mean threshold sensitivity of (assessed by microperimetry)
  - All points
  - Perilesional points
  - Paralesional points
  - Extralesional points
- Mean threshold sensitivity of all points categories (approximately tertiles)
- Number of scotomatous points (assessed by microperimetry)
- Fixation stability (95% BCEA) (assessed by microperimetry)
- Fixation stability (95% BCEA) categories (approximately tertiles)
- NEI VFQ-25 distance activity subscale score
- NEI VFQ-25 near activity subscale score
- NEI VFQ-25 driving subscale score
- Number of subjects currently driving at baseline
- NEI VFQ-25 composite score
- Dry AMD status (iRORA, large drusen) (at Month 24 reporting only)

*Approximately tertiles will be specified consistently across the APL2-303/304 studies to the nearest clinically relevant level.

Baseline will be defined as the last available pre-treatment value taken on or before the first dose date of study drug. In the case a subject did not receive study drug in the ITT population, the last assessment prior to study exit will be used as baseline.

Conversions for height and BMI are as follows:

- Height (m) = Height (cm) x 0.01
- Body mass index (kg/m^2) = Weight (kg) / $[\text{Height (m)}]^2$

Demographics and baseline characteristics will also be listed for the ITT set.

5.3. Medical ocular and non-ocular History

Medical history will be coded using the Medical Dictionary for Regulatory Activities (MedDRA) dictionary version 23.1. Summaries will be presented by System Organ Class (SOC) and Preferred Term (PT) with counts and percentages by treatment group and overall. Each subject will be counted only once in each SOC or SOC/PT summary.

All ocular history will be summarized by SOC and PT with counts and percentages by treatment group and overall. The summaries will be produced separately for the study eye and the fellow eye. Any ocular condition associated with both eyes will be included in both summaries.

Data listings of medical history and ocular history will be provided for the ITT set.

5.4. Prior and Concomitant Medications

Prior and concomitant medications will be coded using World Health Organization (WHO) Drug Dictionary version WHO Drug Global B3-format March 2021. Non-ocular medications will be presented for the ITT set by anatomical therapeutic class (ATC) level 2 (therapeutic main group) and preferred term with counts and percentages by treatment group and overall. A subject who took more than one medication will be counted only once if these medications belong to the same extended ATC classification. In addition, prior and concomitant ocular medications used in study eye will be summarized. Similar summaries will be provided for the ocular medications used in the fellow eye. Any ophthalmological medications (ATC2 = 'Ophthalmologicals') that are not assigned to an eye will be presented under both eyes in the ocular medications tables and will not be presented in the non-ocular medications tables.

Prior medications will be defined as those medications taken prior to the administration of study drug on Day 1. Concomitant medications will be defined as those medications taken following the first administration of study drug on Day 1. Hence medications started before study dosing and continuing into the treatment period are considered as both prior and concomitant medications.

Separate tables will be provided for prior and concomitant medications, respectively. A data listing of prior and concomitant medications, for both ocular and non-ocular medications will be provided for the ITT set.

5.5. Concomitant Procedures

Concomitant procedures will be coded using MedDRA version 23.1. Non-ocular procedures will be presented for the ITT set by preferred term with counts and percentages by treatment group and overall. A subject who had more than one procedure will be counted only once in the summary. In addition, concomitant ocular procedures in study eye will be summarized. Similar summaries will be provided for the ocular procedures used in the fellow eye.

Concomitant procedures will be defined as those procedures taken following the first administration of study drug on Day 1.

A data listing of all procedures, for both non-ocular and ocular procedures will be provided for the ITT set.

5.6. Exposure to Investigational Product

Exposure to the investigational product will be summarized for the ITT, mITT, and Safety sets. All analyses will be repeated for the first 12 months at both the Month 12 and final reporting as well as the full 24 months for the final reporting.

For the analysis of exposure data for the first 12 months, exposure data prior to the Month 12 visit will be summarized (i.e., the Month 12 injection will not be included in the summaries). For analyses of the full 24 months, all data will be summarized.

Injections in the fellow eye will be considered in the calculation of the duration of treatment but will otherwise be considered as a missed injection in the study eye for all other measures of exposure.

The total number of injections and total number of missed injections received in each treatment group will be summarized by treatment group to support per-injection based TEAE rates. The following categories for reason for missed injections will be summarized by treatment group using frequency counts and percentages based on the total number of missed injections: COVID-19 (with a breakdown of COVID-19 reasons as collected in the electronic case report form (eCRF)) vs. non-COVID-19 reasons (any reasons other than eCRF documented COVID-19 reasons). The total number of injections per subject will be summarized by treatment group both as a continuous summary and as a frequency and percent summary. In addition, the following categories of the total number of injections per subject will be summarized by treatment group using frequency counts and percentages:

- First 12 months summaries
  - Monthly: 1-3, 4-6, 7-9, 10-12
  - EOM: 1-2, 3-5, 5-6
- Full 24 months summaries
  - Monthly: 1-3, 4-6, 7-9, 10-12, 13-15, 16-18, 19-21, 22-24
  - EOM: 1-2, 3-5, 5-6, 7-8, 9-10, 11-12

The number of subjects who received at least 75% of the planned injections for the study period (first 12 months or full 24 months) will be presented. For the first 12 months, subjects will be considered to have received 75% of the planned injections if they received at least 9 (out of 12) injections for the Monthly group or 5 at least (out of 6) injections for the EOM group. For the full 24 months, subjects will be considered to have received 75% of the planned injections if they received at least 18 (out of 24) injections for the Monthly group or at least 9 (out of 12) injections for the EOM group.

The number of subjects missing at least one injection will be summarized by treatment group. The number of missed injections per subject as well as well as a breakdown of the number of missed injections per subject due to COVID-19 reasons and due to non-COVID-19 reasons will be summarized by frequency and counts. Additionally, for the missed injections due to COVID-19, the subjects will be summarized by their maximum drug holiday in the following categories:

- No Consecutive Injections (Subjects who are missing an injection due to COVID-19 but not at 2 injection visits in a row)
- 2 Consecutive Injections
- 3 Consecutive Injections
- 4 or More Consecutive Injections

The number of missed injections is defined as the scheduled injections missed up to completion or discontinuation of study treatment.

Additionally, the duration of treatment and compliance will be summarized by treatment group.

Duration of treatment will be defined as

- Monthly group: (date of last injection +30) – date of first injection +1
- EOM group: (date of last injection +60) – date of first injection +1

The duration of treatment will be truncated to a subject's early termination date, month 12 cutoff date, or study completion date as appropriate.

Compliance (%) is defined as the number of injections administered divided by the number of scheduled injections up to completion or discontinuation of study treatment × 100. Compliance will be summarized in the following groups: <75%, ≥75% to <100%, 100%, and >100%.

The number of subjects with scheduled IVT injection visits as well as the number of subjects receiving injections at each of the Months (1-24) will also be summarized by treatment group using frequency counts and percentages. For the EOM group, any injections given on the odd-numbered months will be presented as an unscheduled injection. In addition, to assess the impact of the COVID-19 pandemic on study drug exposure, the following summary will be provided by study visit:

- Number of missed IVT injection visits for study drug
- Number of missed IVT injection visits for study drug due to COVID-19 pandemic reasons
- Number of missed IVT injection visits for study drug due to non-COVID-19 pandemic reasons

Study drug administration will be listed by treatment group. In addition, a listing of total number of injections, duration of treatment, date and day of first and last dose and number of missed injections will be provided for the ITT set.

5.7. Protocol Deviations

Major and minor PDVs will be assessed by sponsor personnel following Protocol Deviation Management Plan.

A PDV is classified as major if there is the potential to significantly impact the completeness, accuracy, and/or reliability of the study data, or affect a subject's rights, safety, or well-being. All PDVs will be identified and finalized prior to the analysis of the first 12 months as well as the analysis of the full 24 month and documented.

In addition, a major PDV will be derived for any subjects who did not have a valid GA lesion area assessment for either Month 10 or 12 and who had not discontinued treatment prior to Month 12. The category for this deviation will be “Study Conduct/Procedures”, the subcategory will be “Study Assessment”, and the study specific category will be “No valid GA lesion area assessment for either Month 10 or Month 12”.

PDVs will be presented for the ITT set by deviation category, subcategory, and study specific category with counts and percentages by treatment group and overall. In addition, similar summaries will be provided for major PDVs as well as for PDVs due to the COVID-19 pandemic.

All PDVs will be listed for the ITT set.

6. EFFICACY ANALYSES

Efficacy analysis including primary, key secondary, secondary, and exploratory analysis will be performed primarily using the mITT set, with subjects grouped according to the treatment assigned at randomization. Available data from all randomized subjects regardless of adherence to the protocol will be included in the efficacy analyses; this includes data from subjects who discontinued study drug early but continued with study assessments. All efficacy data will be listed for the ITT set.

Unless otherwise noted, hypothesis testing and estimation of treatment effects will be performed with a mixed effect model for repeated measure (MMRM) that includes data from all three treatment arms (PM, PEOM, and Sham). The Sham arm will represent the pool of the two sham treatment groups: SM and SEOM (i.e., the two sham arms will be pooled into a single "control" group). All hypothesis tests for efficacy endpoints will be two-sided.

Unless otherwise noted, analysis of efficacy endpoints (primary, key secondary, secondary, and exploratory) in the overall population will be adjusted for the following randomization stratification factors (actual status) and baseline covariates:

For primary efficacy endpoint

- Presence of CNV in the fellow eye (yes; no)
- Baseline GA lesion area ($< 7.5\ mm^2$ or $\geq 7.5\ mm^2$)

For key secondary, secondary, and exploratory endpoints

- Presence of CNV in the fellow eye (yes; no)
- Baseline GA lesion area ($< 7.5\ mm^2$ or $\geq 7.5\ mm^2$)
- Baseline value of the endpoint

Of note, the randomization stratification factors were assessed at the screening visit. In the event of a change in status in the GA lesion area between the screening visit and the baseline visit (i.e., GA lesion area $< 7.5\ mm^2$ at screening to $\geq 7.5\ mm^2$) then the baseline status will be used.

If a statistical model is not directly specified and if necessary, data will be analyzed with the following approaches:

- For continuous endpoints, the MMRM analysis described for the primary endpoint in Section 6.2.2.
- For categorical or ordinal variables, a Cochran Mantel Haenszel (CMH) test with the baseline category status, presence of CNV in the fellow eye (yes or no), and baseline GA lesion area ($< 7.5\ mm^2$ or $\geq 7.5\ mm^2$) as stratification factors.

6.1. Estimands

The primary scientific research question of this study is to assess the effect of pegcetacoplan compared with that of sham at Month 12 regarding the impact on GA lesion progression under-real-life conditions following the ITT principal.

The primary estimand in the study is defined through the following 5 attributes:

- **Population:** GA subjects defined through inclusion and exclusion criteria in the mITT population.
- **Treatment conditions:** The treatment regimen of interest in this study is PM, PEOM, and Sham.
- **Variable (or endpoint):** Change in total area of GA lesions in the study eye from baseline measured by FAF to Month 12.
- **Strategy for addressing intercurrent events:** See description of intercurrent events below.
- **Population-level summary:** Difference in mean change of GA lesion from baseline to Month 12 between pegcetacoplan and sham groups according to MMRM analysis.

The intercurrent events that will be considered are:

- Treatment discontinuation
- Lost to follow up
- Withdrawal from the study

The intercurrent events will be handled with a treatment policy strategy whereby any measured value will be used as is. Missing data resulting from these intercurrent events will be handled implicitly within the MMRM analysis that assumes missing at random.

The key secondary estimands will be defined and analyzed in the similar fashion as the primary estimand. The primary and key secondary estimands are summarized in Table 2.

Table 2: Estimands of Study

Estimand	Definition	Attributes			
		A: Population	B: Variable (or endpoint)	C: Strategy for addressing intercurrent event	D: Population-level summary
		**Main Analyses**			
Primary	The effect of pegcetacoplan compared to sham at Month 12 in impact on GA progression	mITT	Change in the total area of GA lesions from baseline measured by FAF to Month 12	• Treatment policy strategy for subjects who discontinue treatment, are lost to follow-up, or withdraw from the study	Difference in mean change of GA lesion from baseline at Month 12 between pegcetacoplan and sham groups based on MMRM analysis
Key Secondary #1	The effects of pegcetacoplan compared to sham at Month 24 in impact on macular functional response	mITT	Change in mean threshold sensitivity of all points from baseline to Month 24	• Treatment policy strategy for subjects who discontinue treatment, are lost to follow-up, or withdraw from the study	Difference in mean change in mean threshold sensitivity of all points from baseline at Month 24 between pegcetacoplan and Sham groups based on MMRM analysis
Key Secondary #2	The effects of pegcetacoplan compared to sham at Month 24 in impact on monocular maximum reading speed	mITT	Change in monocular maximum reading speed from baseline to Month 24	• Treatment policy strategy for subjects who discontinue treatment, are lost to follow-up, or withdraw from the study	Difference in mean change in monocular maximum reading speed from baseline at Month 24 between pegcetacoplan and Sham groups based on MMRM analysis
Key Secondary #3	The effects of pegcetacoplan compared to Sham at Month 24 in impact on mean FRI Index score	mITT	Change in mean FRI Index score from baseline to Month 24	• Treatment policy strategy for subjects who discontinue treatment, are lost to follow-up, or withdraw from the study	Difference in mean change in mean FRI Index score, from baseline at Month 24 between pegcetacoplan and sham groups based on MMRM analysis

Table 2: Estimands of Study

Estimand	Definition	Attributes			
		A: Population	B: Variable (or endpoint)	C: Strategy for addressing intercurrent event	D: Population-level summary
Key Secondary #4	The effects of pegcetacoplan compared to Sham at Month 24 in impact on NL-BCVA score	mITT	Change in NL-BCVA score from baseline to Month 24	• Treatment policy strategy for subjects who discontinue treatment, are lost to follow-up, or withdraw from the study	Difference in mean change of NL-BCVA score from baseline at Month 24 between pegcetacoplan and sham groups based on MMRM analysis

6.2. Analyses of Primary Efficacy Endpoint

The primary efficacy endpoint is change from baseline to Month 12 in total area of GA lesion(s) in the study eye (in mm^2) based on FAF.

The hypotheses of interest and Type I error management for analyses of the primary endpoint are specified below and take precedence over that specified in the study protocol.

For the primary efficacy endpoint, sensitivity analyses (Section 6.2.3), supplementary analyses (Section 6.2.4), and subgroup analyses (Section 6.2.5) will be performed.

All analyses of the first 12 months will be performed at the Month 12 reporting as well as repeated at the final reporting. Unless otherwise specified, at the time of the final reporting, all analyses will be repeated using the data for the full 24 months.

6.2.1. Type I Error Management

The null hypotheses for the primary efficacy endpoint are the following:

- H_{1a}: There is no difference between PM and Sham in mean change from baseline to Month 12 in total area of GA lesion(s) in the study eye (in mm^2) based on FAF for the mITT set.
- H_{1b}: There is no difference between PEOM and Sham in mean change from baseline to Month 12 in total area of GA lesion(s) in the study eye (in mm^2) based on FAF for the mITT set.

For hypothesis testing of the primary efficacy endpoint, type I error will be controlled using a fixed sequencing approach by testing the two hypotheses (H_{1a} and H_{1b}) sequentially, beginning with H_{1b}. All hypothesis tests for the primary endpoint will be based on a two-sided alpha level of 0.05. The alpha will be adjusted in accordance with the protocol for the number of Data Monitoring Committee (DMC) unmasked reviews prior to the Month 12 analysis timepoint (i.e., 4 DMC unmasked reviews prior to Month 12: 2-sided alpha level = 0.0496). Testing for statistical significance proceeds to H_{1b} only if H_{1a} is statistically significant. The study will be considered positive if the first hypothesis (H_{1a}) is rejected.

6.2.2. Main Analysis of Primary Efficacy Endpoint

The primary endpoint will be analyzed in the mITT population with subjects grouped according to the treatment assigned at randomization.

A MMRM model will be used to analyze the primary endpoint. The analysis model will include treatment (PM, PEOM, sham), presence of choroidal neovascularization in the fellow eye at baseline (Yes, No) and baseline GA lesion area (< 7.5 mm^2 or ≥ 7.5 mm^2) as fixed effects, time (study month, categorical) as a factor, the time × treatment interaction term, and the baseline GA lesion area (< 7.5 mm^2 or ≥ 7.5 mm^2) × time interaction term. The least square [LS] mean change from baseline to Month 12 will be estimated from the model for each of three arms as well as the comparisons of each of the three arms to each other. For other time points of interest, LS mean change from baseline will be estimated and compared between treatments. For each estimated LS mean, the corresponding 95% CI will be presented based on the model. For the comparison of the LSmeans, the corresponding 95% CI and the 2-sided P-value along with the percentage difference (difference in LS means between the arms/the comparison group LS mean) will be

presented. A common unstructured covariance matrix will be used to model the within-subject errors, the sandwich estimator (Diggle, Liang, and Zeger 1994) will be used to estimate the standard errors of the fixed effects parameters, and the degrees of freedom will be partitioned into between-subject and within-subject portions. If there are convergence problems with the model, then a heterogeneous autoregressive (1) covariance matrix will be used. If convergence problems still exist, an autoregressive (1) covariance matrix will be used. The LS mean of the change from baseline in the total area of GA lesion(s) ± the standard error will be plotted over time by treatment group.

The observed values for the total area of GA lesion(s) will be summarized by treatment group and visit for both the study and the fellow eye. Summaries will present the descriptive statistics for baseline, absolute values and change from baseline data by visit.

The mean change from baseline in the total area of GA lesion (s) in the study eye ± the standard error will be plotted over time by treatment group.

All GA lesion (s) size data will be listed for the ITT set.

6.2.3. Sensitivity Analyses of Primary Efficacy Endpoint

Sensitivity analyses will be performed to evaluate the robustness of the primary analysis results. Analyses will be performed for the overall population based on the outcome of the primary analyses including all subjects in the mITT set.

6.2.3.1. Missing Data Analyses Based on Multiple Imputation

The following sensitivity analysis based on multiple imputation will be performed, as appropriate, using the same statistical approach as the one used in the primary analysis:

- Subjects with treatment discontinuation or study withdrawal assessed as potentially related to study drug or lack of efficacy will have missing data after the discontinuation or withdrawal imputed via a control-based imputation based on the sham control subjects. This will be based on the mITT set.
    - Subjects who discontinue the treatment or study due to reasons that are potentially related to study drug or lack of efficacy include
        - Subjects who discontinue due to ocular adverse event, adverse event related to treatment, or adverse event related to injection procedure
        - Subjects who withdraw consent due to lack of efficacy (specify field includes ‘Lack of efficacy’) or not otherwise specified as not related to efficacy (specify field includes “Not specified”)
- Subjects with a monotone missing data pattern due to treatment discontinuation or study withdrawal will have missing data after the last assessment explicitly imputed by multiple imputation using a tipping point analysis method assuming missing not at random (MNAR). This will be based on the mITT set.

Imputation for the non-monotone missing pattern (i.e., arbitrary missing pattern) will be performed prior to the multiple imputation for the monotone missing pattern (i.e., where a missing GA area measurement at a visit for a subject implies that GA area measurements at all subsequent visits for that subject are missing).

For the nonmonotone missing pattern, missing value(s) between two visits with measured GA area will be imputed using the monotone data MCMC method, which was first proposed by Li (1988) and Liu (1993) described the algorithm. Multiple imputation will then be carried out for monotone missing pattern.

The two imputation methods will be implemented in SAS using the three standard steps to generate inference from imputed data: imputation step, analysis step, and pooling step. Details are illustrated with SAS codes in Section 1.2.2.

- The missing data are filled in 1000 times to generate 1000 complete datasets.
- The 1000 complete datasets are analyzed by using the same approach as for the analysis for the primary objective.
- The results from the 1000 complete datasets are combined for the inference.

Methods used in the imputation step are described below.

- Pattern Mixture Model Method (a copy-reference approach): The pattern mixture model method will be implemented.
    - Subjects with monotone missing data in the pooled sham arm will have missing data imputed based on the observed values in the pooled sham arm.
    - For the active treatment arms, subjects with monotone missing data who discontinued treatment or withdrew from the study due to reasons that are potentially related to study drug or lack of efficacy will have missing data imputed based on the pooled sham arm.
    - For the active treatment arms, subjects with monotone missing data who did not discontinue treatment or withdraw from the study due to reasons that are potentially related to study drug or lack of efficacy will have missing data imputed based on the observed values in the corresponding treatment arm.
- Tipping Point Analysis Method (a delta-adjusted approach): The tipping point analysis method will be implemented for each active treatment arm under the MNAR assumption by searching for a tipping point that reverses the conclusion regarding positive treatment effect. For the pooled sham arm, subjects will have missing data imputed based on the observed values in the pooled sham arm. For the active treatments, subjects with monotone missing data without treatment discontinuation or early withdrawal will have missing data imputed based on the observed values in the corresponding treatment arm. For the active treatment arms, subjects with monotone missing data due to treatment discontinuation or early withdrawal will have the missing data imputed based on the available values (observed values plus values imputed for non-monotone missing data) in the same treatment arm with a shift parameter added to the imputed values. Multiple imputation will be implemented on the differences between two consecutive visits, with the shift parameter allocated to the missing data point(s) proportionally across timepoints. (e.g. For a shift parameter of 0.48 mm^2 at Month 12, incremental shifts of 0.08 mm^2, would apply to Month 2, Month 4, … and Month 12, respectively. 0.08 mm^2 x 6 visits = 0.48 mm^2) The range of the shift parameters will be from 0.06 mm^2 to 0.72 mm^2 by increments of 0.06

mm^2 for Month 12 and 0.12 mm^2 to 1.44 mm^2 for Month 24 for the difference in the change of GA area from baseline between each pegcetacoplan arm (PM or PEOM) and Sham arm. The precision of the tipping point will be at two decimal points. A tipping point may not exist within reasonable clinical assumptions.

For each analysis, the number and percent of subjects without monotone missing data, the number of subjects with monotone missing data and a breakdown of reasons will be reported. The reasons will include monotone missing without treatment discontinuation or early withdrawal, monotone missing due to treatment discontinuation or early withdrawal due to reasons that are potentially related to study drug or lack of efficacy, and monotone missing due to treatment discontinuation or early withdrawal not due to reasons that are potentially related to study drug or lack of efficacy.

6.2.3.2. Other Sensitivity Analyses

The primary endpoint will also be analyzed without pooling the two sham arms (SM and SEOM). The comparison for pegcetacoplan and sham injection within each dose schedule (i.e., PM vs SM and PEOM vs SEOM, respectively) will be conducted using the MMRM analyses described in Section 6.2.2.

The primary endpoint will also be summarized excluding GA total area assessments with an indeterminate boundary. The summary and presentation of the observed values for the total area of GA lesion(s) by treatment group and visit described in Section 6.2.2 will be repeated. The summary and presentation of the analysis results for all timepoints described in Section 6.2.2 will be repeated.

6.2.4. Supplemental Analyses of the Primary Efficacy Endpoint

6.2.4.1. COVID-19 Adjusted Estimand

Despite the occurrence of the COVID-19 pandemic, the scientific question of interest in this study remains unchanged. The primary scientific research question of this study is to assess the effect of pegcetacoplan compared with that of sham at Month 12 on GA lesion progression under-real life conditions without COVID-19 pandemic impact.

To manage the increase in missed/not received injections due to COVID-19 pandemic (as collected on the eCRF), i.e., undertreatment with an expected relevant impact on efficacy, the hypothetical strategy will be used whereby assessments after the intercurrent event (relevant undertreatment) occurs will be set to missing/censored in the analysis. The threshold for missed/not received injections due to the COVID-19 pandemic is missing a pre-specified number of scheduled injections prior to the analysis timepoint of interest. For the Monthly schedule group, this is missing 2 or more injections and for the EOM schedule group, this is missing 1 or more injections prior to the Month 12 visit attributable due to the COVID-19 pandemic.

In terms of the underlying cause of the excessive missing/not received injections and subsequent missing data, it is assumed that the missingness resulting from censoring occurs at random because of the ongoing pandemic and not because of the subjects' unobserved outcome, so missing data resulting from this intercurrent event in the primary analysis model will be handled implicitly in the MMRM analysis.

The number of subjects with any censoring included in the model, the number of subjects with any censoring not included in the model, and the number of assessments censored by visit will be presented for each treatment group.

All summaries and presentations of the observed values for the total area of GA lesion(s) in the study eye by treatment group and visit described in Section 6.2.2 will be repeated for the COVID-19 adjusted estimand. All summaries and presentations of the analysis results for all timepoints described in Section 6.2.2 will be repeated for the COVID-19 adjusted estimand. The same plots as for the primary endpoint analysis will be presented.

In addition, a supplemental analysis will also be performed where the threshold for missed/not received injections will be restricted to external events attributable to COVID-19. These events are defined based on the COVID-19 collection form and include COVID-19 diagnosis, COVID-19 Suspected, Site Closure, Travel Ban, Shelter in Place, City Lockdown, and Other. Except for the plots, the analyses described above will be repeated for the threshold based only on these external events attributable to COVID-19.

6.2.4.2. Per Protocol Set

The main analysis described in Section 6.2.2 will be repeated using the Month 12 PP set to investigate the impact of changing the population in the estimand. Any GA lesion area assessments that are deemed not valid according to what is described in Section 4.5 will be set to missing in this analysis. The summary and presentation of the observed values for the total area of GA lesion(s) by treatment group and visit described in Section 6.2.2 will be repeated. The summary and presentation of the analysis results for all timepoints described in Section 6.2.2 will be repeated.

6.2.4.3. Rate of Change Analyses

The mean rate of change in GA area (i.e., slope) will be compared between each pegcetacoplan arm and the pooled sham arm by use of linear mixed effects model assuming time as continuous and linear (“slope model”). The analysis model will include treatment (PM, PEOM, sham), presence of choroidal neovascularization in the fellow eye at baseline (Yes, No), baseline GA lesion area (< 7.5 mm^2 or ≥ 7.5 mm^2) as fixed effects, time (study month, continuous assuming linearity), the time × treatment interaction term as well as the baseline GA lesion area × time interaction term. The response variable will be the GA lesion area. A common unstructured covariance matrix will be used to model the within-subject errors, the sandwich estimator will be used to estimate the standard errors of the fixed effects parameters, and the degrees of freedom will be partitioned into between-subject and within-subject portions. If there are convergence problems with the model, then a heterogeneous autoregressive (1) covariance matrix will be used. If convergence problems still exist, an autoregressive (1) covariance matrix will be used. The mean rate of change (slope), standard error, and confidence interval will be estimated for the baseline to Month 12 as well as the baseline to Month 6 and Month 6 to Month 12 periods for each treatment group. In addition, the estimated difference in slopes among the treatment groups along with the 95% CIs and p-value will be reported. The slopes and 95% CIs will be converted to an annualized rate of growth for each period and each treatment group.

The observed values for the total area of GA lesion(s) will be summarized by treatment group and visit for the baseline to Month 6 and Month 6 to Month 12 time periods. Summaries will present the descriptive statistics for baseline, absolute values and change from baseline to

Month 6 and change from Month 6 to Month 12 data. In addition, the annualized rate of growth for each time period will also be presented.

The mean rate of change in GA area will be compared between each pegcetacoplan arm and the pooled sham arm by use of a piecewise linear mixed effect model assuming time as continuous and piecewise linear ("piecewise slope model"). The analysis to be performed will be similar to what is described above in the "slope model" except that a knot at the Month 6 visit will be added which allows for the slope of lesion growth to differ between the two periods for each of the treatment groups. The same presentation as for the "slope model" will be prepared except for the Baseline to Month 12 slope. Additionally, an investigation of the impact of changing the knot to Month 2, 4, 8, 10 will be explored if necessary.

6.2.5. Subgroup Analyses of Primary Efficacy Endpoint

Subgroup analyses will be performed to evaluate the consistency of the primary analysis results across subgroups defined by demographic and baseline characteristics. Analyses will be performed for the primary efficacy endpoint (change from baseline in total area of GA lesion(s) in the study eye at Month 12) for each of the following subgroups (as appropriate per actual subgroup sample size, levels with low sample size may be pooled to allow for an analysis to be conducted):

- Age Group (<75 years, 75 to <85 years, ≥85 years)
- Sex (male, female)
- Race (White, Black or African American, Asian, American Indian or Alaskan Native, Native Hawaiian or other Pacific Islander, multiple, unknown)
  - Note: If most subjects (e.g., >90%) are of a single race, this analysis will not be conducted.
- Geographic Region (United States vs Rest of World)
- Subgroups indicative of disease severity at baseline
  - Study eye baseline GA lesion size (<7.5 mm^2; ≥7.5 mm^2)
  - Study eye baseline GA lesion size categories (approximately tertiles based on APL2-303/304 combined data*)
  - Study eye baseline NL-BCVA categories (≥70, ≥60 - <70, ≥35 - <60, <35 ETDRS letters)
  - Study eye baseline NL-BCVA categories (<60 vs. ≥60 ETDRS letters)
  - Study eye baseline LL-BCVA categories (approximately tertiles based on APL2-303/304 combined data*)
  - Study eye baseline monocular maximum reading speed categories (approximately tertiles based on APL2-303/304 combined data*)
  - Baseline FRI Level (1,2,3,4)
  - Study eye mean threshold sensitivity of all points categories (approximately tertiles)
- Subgroups associated with GA progression
  - Study eye baseline GA focality (multifocal, unifocal)

- Study eye baseline GA lesion location (subfoveal involvement, without subfoveal involvement)
- Baseline GA laterality (bilateral GA (with or without CNV in fellow eye) vs. Study eye GA Only)
- Baseline CNV in fellow eye (Fellow eye CNV vs. No fellow eye CNV)
- Study eye baseline LLD categories (<20 vs. ≥ 20 ETDRS letters)
- Study eye baseline LLD categories (approximately tertiles based on the APL2-303/304 combined data*)
- Study eye baseline NL-BCVA (<60 vs. ≥60 ETDRS letters) and LLD (<20 vs. ≥ 20 ETDRS letters) combinations

*Approximately tertiles will be specified consistently across the APL2-303/304 studies to the nearest clinically relevant level.

For the primary efficacy endpoint, the approach described in the Section 6.2.2 will be used for each subgroup analysis based on the data subset for the subject subgroup of interest. Baseline covariates included in the main analysis but no longer relevant given the subgroup of interest will be excluded from the model. The estimated treatment effects (PM vs. Sham; PEOM vs. Sham) and corresponding 95% CIs and p-values from the models will be displayed graphically for each pegcetacoplan treatment arm and each level of the subgroups specified (e.g., via forest plots).

6.2.6. Full 24 Month Analyses

In general, the same analysis approach for the changes from baseline GA lesion size for the final reporting of the full 24 months will be repeated as described in Sections 6.2.2, 6.2.3, 6.2.4, 6.2.5. Key differences include:

- The definition of excessive missed/not received injection in the supplementary estimand will be defined based on the Month 24 period. The threshold for missed/not received injections due to the COVID-19 pandemic is missing 4 or more injections for the Monthly schedule group and 2 or more injections for the EOM schedule group prior to the Month 24 visit attributable due to the COVID-19 pandemic.
- The Month 12 PP set will be replaced with the Month 24 PP set for the supplementary analysis.

6.2.7. Exploratory Endpoints Closely Related to the Primary Efficacy Endpoint

Analyses of the following endpoints closely related to the primary efficacy endpoint, will be performed for the mITT set to provide supplemental information.

6.2.7.1. Change in Square Root of GA Area

For each subject and timepoint, the square root of GA area will be calculated. The change from baseline in square root of GA area will be analyzed using the same methodology as described for the primary endpoint analysis in Section 6.2.2, except that change from baseline in the square

root of GA area will be the response variable. This analysis will be conducted both for the first 12 months summaries as well as the full 24 months summaries.

6.2.7.2. Percent Change in GA Area and Square Root of GA Area

For each subject and timepoint, the percent change in GA area will be defined as the change from baseline in GA area (mm^2) divided by the GA area (mm^2) at baseline and the percent change in square root of GA area will be defined as the change from baseline in GA area (mm) divided by the GA area (mm) at baseline. The percent change in GA area from baseline and percent change in square root of GA area will be analyzed using the same methodology as described for the primary endpoint analysis in Section 6.2.2, except that percent change from baseline will be the response variable. This analysis will be conducted both for the first 12 months summaries as well as the full 24 months summaries.

6.3. Analyses of Key Secondary Efficacy Endpoints

The key secondary efficacy endpoints are listed in Section 2.2.2. At the Month 12 reporting, all key secondary efficacy endpoints will be evaluated based on data from baseline to Month 12 (see Section 3.5). For the Month 12 analyses, post-baseline visits, up to and including Month 12, will be included and nominal p-values will be presented. At the time of the final study analysis, all key secondary efficacy endpoints will be evaluated based on data from baseline to Month 24, and formal statistical testing for key secondary endpoints will be performed as described in Section 6.3.1. Unless otherwise specified, all secondary efficacy endpoints will be analyzed for the mITT set.

6.3.1. Type I Error Management

The planned submission of pegcetacoplan in GA consists of 2 studies of very similar design (APL2-303 and APL2-304), each with multiple endpoints. It is planned to control the type I error rate for the primary endpoint hypotheses testing at the level of the individual studies (as described in Section 6.2.1), and at a level of the submission as a whole for the key secondary endpoints hypotheses testing based on pooling APL2-303 and APL2-304. Figure 2 presents the overall hypotheses testing strategy for the APL2-303 and APL2-304 studies.

The primary endpoint hypotheses in the PM vs. Sham and PEOM vs. Sham will be tested in a hierarchical order within a study. If both hypothesis tests for the primary endpoint in both studies are statistically significant, then hypotheses testing will be performed for the key secondary endpoints within the pooled APL2-303/APL2-304 data for hypotheses $H_{2a} – H_{4b}$ and within the APL2-304 study for H_{5a} and H_{5b} at the presented α level. The key secondary efficacy endpoints hypotheses that will be tested in the pooled APL2-303/APL2-304 studies are below:

- H_{2a} There is no difference between PM and Sham regarding mean change from baseline in monocular maximum reading speed (study eye), as assessed by MNRead or Radner Reading Charts at Month 24.
- H_{2b}: There is no difference between PEOM and Sham regarding mean change from baseline in monocular maximum reading speed (study eye), as assessed by MNRead or Radner Reading Charts at Month 24.

- H_{3a}: There is no difference between PM and Sham regarding mean change from baseline in mean FRI Index score at Month 24.
- H_{3b}: There is no difference between PEOM and Sham regarding mean change from baseline in mean FRI Index score at Month 24.
- H_{4a}: There is no difference between PM and Sham regarding mean change from baseline in NL-BCVA score at Month 24 (study eye) as assessed by ETDRS chart.
- H_{4b}: There is no difference between PEOM and Sham regarding mean change from baseline in NL-BCVA score at Month 24 (study eye) as assessed by ETDRS chart.

The key secondary efficacy endpoints hypotheses that will be tested within the APL2-304 study are below:

- H_{5a}: There is no difference between PM and Sham regarding mean change from baseline in mean threshold sensitivity of all points (study eye) for the evaluation of the macular functional response, as assessed by mesopic microperimetry at Month 24.
- H_{5b}: There is no difference between PEOM and Sham regarding mean change from baseline in mean threshold sensitivity of all points (study eye) for the evaluation of the macular functional response, as assessed by mesopic microperimetry at Month 24.

Hypothesis tests will be conducted in the order indicated by the arrows in Figure 2 and will continue as long as all preceding hypotheses are rejected at a given α level. If both the PM vs. Sham and the PEOM vs. Sham comparisons are statistically significant for a given key secondary endpoint, then the α will be allocated equally to the other key secondary hypotheses. At the study-level, the type I error rate (one-sided) is controlled at 0.025 for the primary endpoint hypotheses. In the submission, the type I error rate (one-sided) is controlled at 0.000625 (0.025^2) for the primary endpoint hypotheses and at 0.025 for the key secondary endpoint hypotheses. All hypothesis tests will be adjusted for the number of DMC unmasked reviews prior to the Month 12 analysis.

Type I error will be controlled by the testing procedure via the graphical methods approach (Bretz 2009). All confidence intervals and p-values will be presented without adjustments.

If at least one of the hypothesis tests for the primary endpoint in the APL2-303 study is not statistically significant but both hypothesis tests for the primary endpoint in the APL2-304 study are statistically significant, then any hypothesis testing for the key secondary endpoints will not be rejected at the submission level. Within the APL2-304 study hypotheses testing for the key secondary endpoints will be tested as shown in Figure 3 and type I error will be controlled using a fixed sequencing approach. All hypotheses tests will be adjusted for the number of DMC unmasked reviews prior to the Month 12 analysis. All confidence intervals and p-values will be presented without adjustments.

Figure 2: APL2-303 and APL2-304 Hypothesis Testing and Type I Error Control

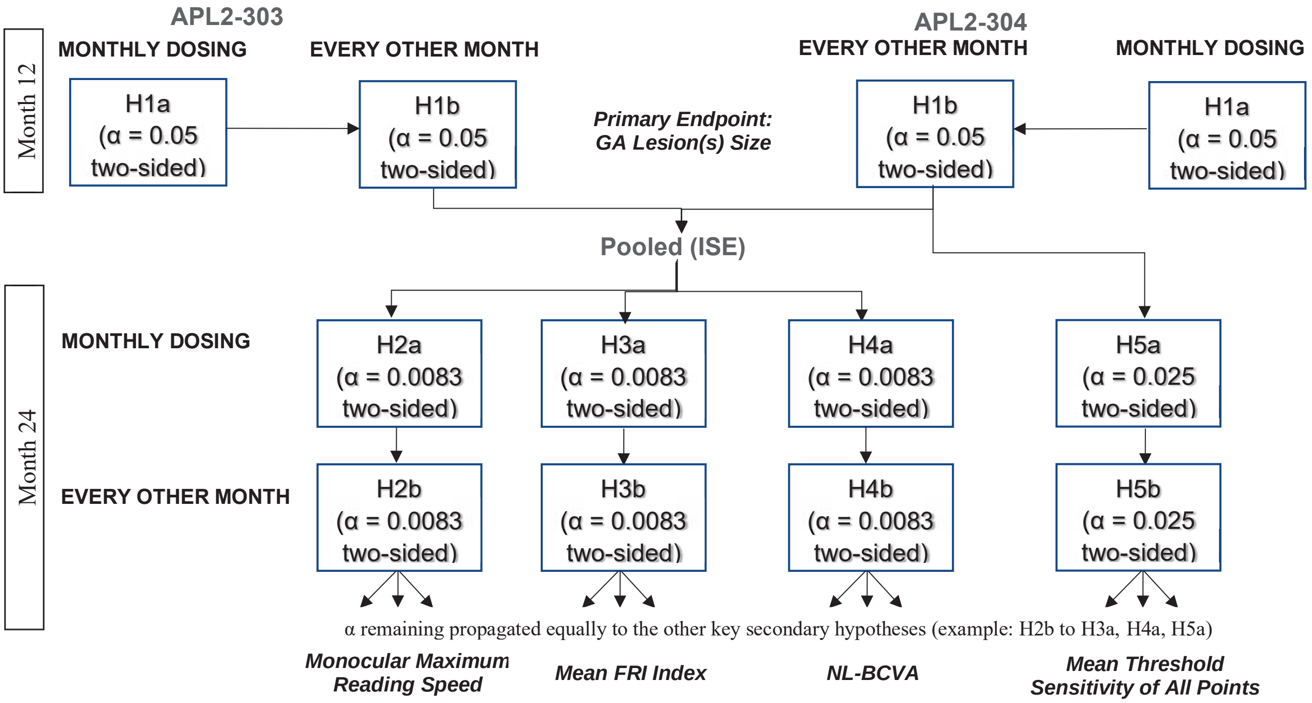

Endpoint data are compared to sham injections in hypotheses testing. Hypotheses can only be tested in sequential order as indicated by arrows. Sequential testing will continue as long as all preceding hypotheses can successfully be rejected at the given alpha level. At the study-level, the Type I error rate (one-sided) is controlled at 0.025 for the primary endpoint hypotheses. In the submission, the Type-I error rate (one-sided) is controlled at 0.000625 (0.025^2) for the primary endpoint hypotheses and at 0.025 for the key secondary endpoints.

Figure 3: APL2-304 Hypothesis Testing and Type I Error Control If There is No α Remaining in APL2-303 to Allow Pooling Secondary Functional Endpoints

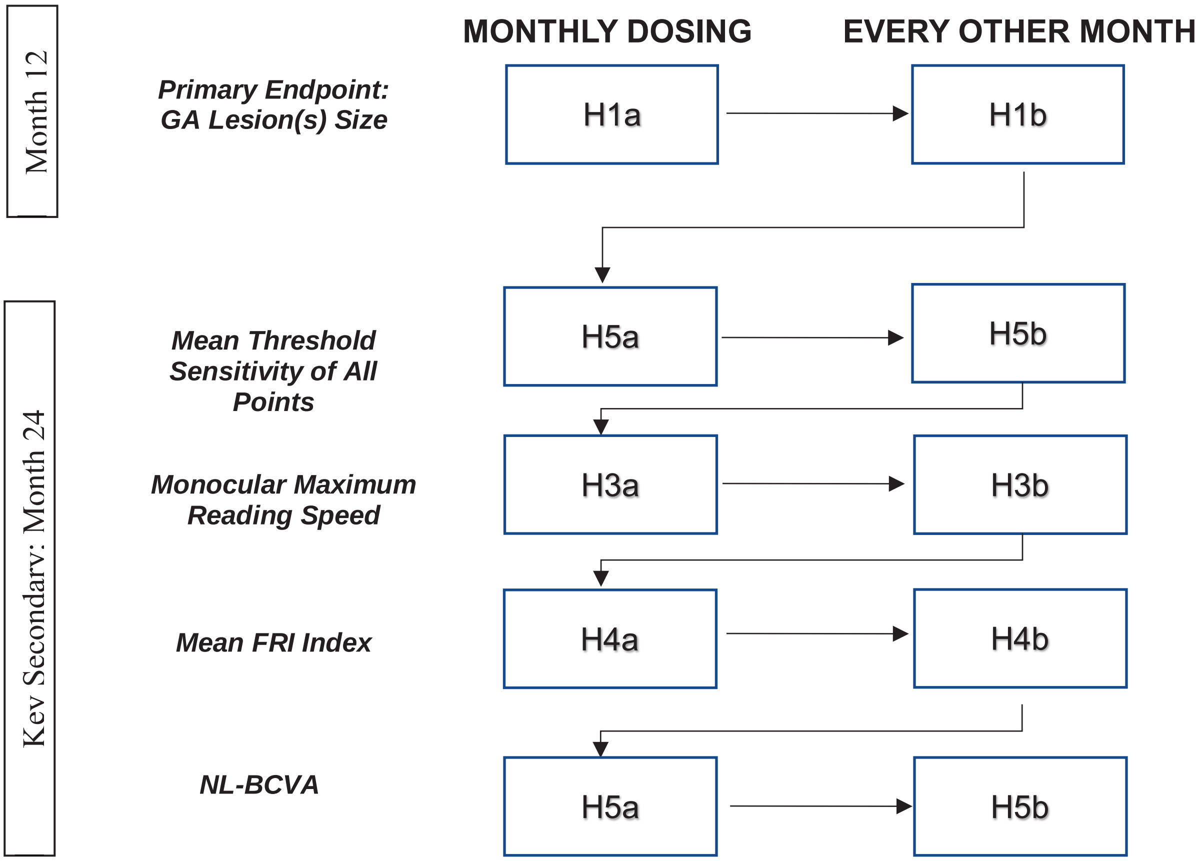

Endpoint data are compared to sham injections in hypotheses testing. Hypotheses can only be tested in sequential order as indicated by arrows. Sequential testing will continue as long as all preceding hypotheses can successfully be rejected at the given alpha level. This testing tree will only be used when APL2-303 cannot pass α from the primary endpoint testing to the pooled secondary functional endpoint testing.

6.3.2. Main Analyses of Key Secondary Efficacy Endpoints

6.3.2.1. Mean Threshold Sensitivity of All Points

Mean threshold sensitivity of all points will be determined from the mesopic microperimetry as an assessment of the macular functional response.

Mean threshold sensitivity of all points in the study eye will be summarized and analyzed in a similar fashion as the primary efficacy endpoint using MMRM described in Section 6.2.2. The model will include treatment (PM, PEOM, Sham), presence of CNV in the fellow eye (yes or no), and baseline GA lesion area (< 7.5 mm^2 or ≥ 7.5 mm^2) as fixed effects; baseline mean threshold sensitivity of all points as a covariate, time (in months) as a factor; the time × treatment interaction term as well as the mean threshold sensitivity of all points × time interaction term.

The observed values for mean threshold sensitivity of all points will be summarized by treatment group and visit for both the study and the fellow eye. Summaries will present the descriptive statistics for baseline, absolute values and change from baseline data.

The mean change from baseline in the mean threshold sensitivity of all points of the study eye ± the standard error as well as the LS mean of the change from baseline in the mean threshold sensitivity of all points of the study eye ± the standard error will be plotted over time by treatment group.

Data that is not acceptable quality for the main analysis, as identified by the reading center per the Imaging Charter, will not be included in this analysis.

All microperimetry data will be listed for the ITT Set.

6.3.2.2. Monocular Maximum Reading Speed

Maximum reading speed will be calculated per MNRead or Radner user manuals, with no adjustment for reading inaccuracy. An additional step to cap resulting reading speed values at a maximum of 300 words per minute will be implemented. Values above 300 words per minute exceed values reported in the literature for adults with no vision impairment for MNRead (Calabrese et al. 2016) or Radner (Radner et al. 2002; Radner and Diendorfer 2014) assessments. Maximum reading speed will be calculated as the mean of the three highest nonzero reading speeds (or two, or one value, as available) (Baskaran et al. 2019; Patel et al. 2011), except when all words per minute are calculated as 0: then the maximum reading speed will be calculated as 0.

Monocular maximum reading speed in the study eye will be summarized and analyzed in a similar fashion as the primary efficacy endpoint using MMRM described in Section 6.2.2. The model will include treatment (PM, PEOM, Sham), presence of CNV in the fellow eye (yes or no), and baseline GA lesion area (< 7.5 mm^2 or ≥ 7.5 mm^2) as fixed effects; baseline monocular maximum reading speed as well as the chart type (MNRead or Radner) as covariates, time (in months) as a factor; the time × treatment interaction term as well as the baseline monocular maximum reading speed × time interaction term.

The observed values for the monocular maximum reading speed will be summarized by treatment group and visit for both the study and the fellow eye. Summaries will present the descriptive statistics for baseline, absolute values and change from baseline data.

The mean change from baseline in the monocular maximum reading speed of the study eye ± the standard error as well as the LS mean of the change from baseline in the monocular maximum reading speed of the study eye ± the standard error will be plotted over time by treatment group.

In addition, the categorical changes (improvements of ≥ 20 to <40, ≥ 40 to <60, ≥ 60 and minimal/no change (-20 to 20), worsening of ≥ 20 to <40, ≥ 40 to <60, ≥ 60) for the study eye will be summarized by treatment group and visit.

The shift from baseline in the monocular maximum reading speed for the study eye categories over time will be presented by treatment group for the following categories:

- < 40 wpm
- 40 to < 80 wpm
- 80 to <160 wpm
- ≥ 160 wpm

All monocular maximum reading speed data will be listed for the ITT set.

6.3.2.3. Mean FRI Index Score

The mean FRI Index score will be calculated per the scoring algorithm in the FRI Index user's manual.

The mean FRI Index score will be summarized and analyzed in a similar fashion as the primary efficacy endpoint using MMRM described in Section 6.2.2. The model will include treatment (PM, PEOM, Sham), presence of CNV in the fellow eye (yes or no), and baseline GA lesion area (< 7.5 mm^2 or ≥ 7.5 mm^2) as fixed effects; baseline mean FRI Index score and baseline study eye status (Better-seeing Eye vs. Worse-seeing Eye) as covariates, time (in months) as a factor; the time × treatment interaction term as well as the baseline mean FRI Index score × time interaction term.

The observed values for the mean FRI Index score will be summarized by treatment group and visit. Summaries will present the descriptive statistics for baseline, absolute values and change from baseline data.

The mean change from baseline in the mean FRI Index score ± the standard error as well as the LS mean of the change from baseline in the mean FRI Index score ± the standard error will be plotted over time by treatment group.

In addition, the mean FRI Index score will be converted to the FRI Level variable, as per the user manual, ranging from 1 (Unable to do) to 4 (Totally independent). The categorical changes from baseline (improvements (an increase in FRI level), minimal/no change (no change in FRI level), and worsening (decrease in FRI level)) will be summarized by treatment group and visit.
The shift from baseline in the FRI level over time will be presented by treatment group.

All FRI data will be listed for the ITT set.

6.3.2.4. NL-BCVA Score

The mean NL-BCVA score for the study eye will be summarized and analyzed in a similar fashion as the primary efficacy endpoint using MMRM described in Section 6.2.2. The model will include treatment (PM, PEOM, Sham), presence of CNV in the fellow eye (yes or no), and baseline GA lesion area (< 7.5 mm^2 or ≥ 7.5 mm^2) as fixed effects; baseline NL-BCVA score as a covariate, time (in months) as a factor; the time × treatment interaction term as well as the baseline NL-BCVA score × time interaction term. Note, for the second year, only timepoints that were measured for all three treatment groups will be included in the model.

The observed values for the NL-BCVA will be summarized by treatment group and visit for both the study and the fellow eye. Summaries will present the descriptive statistics for baseline, absolute values and change from baseline data.

The mean change from baseline in the NL-BCVA score for the study eye +/- the standard error as well as the LS mean of the change from baseline in the NL-BCVA score for the study eye ± the standard error will be plotted over time by treatment group.

In addition, the categorical changes (improvements of ≥15, ≥10 to <15, ≥5 to <10, and minimal/no change (-4 to 4), worsening of ≥5 to <10, and ≥10 to <15 and ≥15) for the study eye will be summarized by treatment group and visit.

The shift from baseline in the NL-BCVA of the study eye categories (≥70, ≥60 - <70, ≥35 - <60, ≥20 - <35, <20 ETDRS letters over time will be presented by treatment group and visit.
The above-mentioned ETDRS letter thresholds are based on WHO definitions for vision impairment and blindness. WHO criteria were implemented in an attempt to capture the progressive nature of Geographic Atrophy and the ultimate treatment goal of avoiding successive vision impairment and blindness through application of active treatment (Pegcetacoplan vs. Sham).[1]

The average NL-BCVA score over the last 3 months for each reporting period (Month 10 - Month 12 for the Month 12 reporting; Month 22 - Month 24 for the Month 24 reporting) will be calculated for each subject who has at least one NL-BCVA assessment in these windows. The observed values for the NL-BCVA and changes from baseline will be summarized by treatment group for the study eye. Summaries will present the descriptive statistics for baseline, absolute values and change from baseline data.

All NL-BCVA data will be listed for the ITT set.

6.3.3. Sensitivity Analyses of Key Secondary Efficacy Endpoints

The missing data analyses based on imputation described in Section 6.2.3.1 will be repeated for the key secondary efficacy endpoints at the time of the final study analysis for the full 24 months only. The key secondary endpoints will also be analyzed without pooling the two sham arms (SM and SEOM) for the full 24 months only.

In addition, to assess the potential effect of different assessment tools, the main analysis of monocular maximum reading speed will be repeated by reading chart type.

---

[1] approximated from 6/18; see https://icd.who.int/browse11/l-m/en#/http://id.who.int/icd/entity/1103667651

Maximum reading speed with adjustment for reading inaccuracy will also be analyzed, only in the study eye, for the first 12 months as well as the full 24 months.

6.3.4. Supplementary Analyses of Key Secondary Efficacy Endpoints

The supplementary analyses described for the primary endpoint in Section 6.2.4.1 and Section 6.2.4.2 will also be performed for the key secondary endpoints for the first 12 months as well as the full 24 months.

In addition, the analysis of mean threshold sensitivity of all points will be repeated for the study eye excluding data not of acceptable quality for either the main or supplementary analysis, as identified by the reading center, per the Imaging Charter or where the fixation loss was > 20%. This analysis will be performed for the first 12 months as well as the full 24 months.

6.3.5. Subgroup Analyses of Key Secondary Efficacy Endpoints

The subgroup analyses of the key secondary endpoints will be performed for the full 24 months. Select subgroup analyses of the key secondary endpoints may be performed at Month 12, if necessary, to support regulatory filings. The subgroups evaluated for key secondary endpoints will be the same as evaluated for the primary efficacy endpoint (see Section 6.2.5).

In addition, the following subgroup analyses will be performed:

<u>Monocular Maximum Reading Speed</u>

- Study eye baseline monocular maximum reading speed (<60, 60-<160, ≥160)
- Study eye fixation stability (95% BCEA) categories (approximately tertiles)

<u>Mean FRI Index score</u>

- Baseline Study Eye Status (Better-seeing Eye vs. Worse-seeing Eye)
- Study eye fixation stability (95% BCEA) categories (approximately tertiles)

<u>NL-BCVA</u>

- Study eye fixation stability (95% BCEA) categories (approximately tertiles)

For each endpoint, a MMRM similar to that specified for the main analysis of the key secondary endpoints (Section 6.3.2) will be used for each subgroup analysis based on the data subset for the subject subgroup of interest. Baseline covariates included in the main analysis but no longer relevant given the subgroup of interest will be excluded from the model. The estimated treatment effects (PM vs. Sham or PEOM vs. Sham) and corresponding 95% CIs and p-values from the models will be displayed graphically for each pegcetacoplan arm and each level of the subgroups specified (e.g. via forest plots).

6.4. Analyses of Other Secondary Efficacy Endpoints

The secondary efficacy endpoints are listed in Section 2.2.3. At the time of the Month 12 reporting, all secondary efficacy endpoints will be evaluated based on data from baseline to Month 12 (see Section 3.5). For the Month 12 analyses, post-baseline visits, up to and including Month 12, will be included. At the time of the final study analysis, all secondary efficacy

endpoints will be evaluated based on data from baseline to Month 24. For the Month 24 analyses, post-baseline visits, up to and including Month 24, will be included.

Unless otherwise specified, all secondary efficacy endpoints will be analyzed for the mITT set.

6.4.1. Low Luminance Best-Corrected Visual Acuity (LL-BCVA)

LL-BCVA of the study eye will be summarized and analyzed in the similar fashion as the primary efficacy endpoint using MMRM described in Section 6.2.2. The model will include treatment (PM, PEOM, Sham), presence of CNV in the fellow eye (yes or no), and baseline GA lesion area (< 7.5 mm^2 or ≥ 7.5 mm^2) as fixed effects; baseline LL-BCVA score as a covariate, time (in months) as a factor; the time × treatment interaction term as well as the baseline LL-BCVA score × time interaction term. Note, for the second year, only timepoints that were measured for all three treatment groups will be included in the model.

The observed values for the LL-BCVA will be summarized by treatment group and visit for both the study and the fellow eye. Summaries will present the descriptive statistics for baseline, absolute values and change from baseline data.

The mean change from baseline in the LL-BCVA score in the study eye ± the standard error as well as the LS mean of the change from baseline in the LL-BCVA score in the study eye ± the standard error will be plotted over time by treatment group.

The categorical changes (improvements of ≥15, ≥10 to <15, ≥5 to <10, and minimal/no change (-4 to 4), worsening of ≥5 to <10, and ≥10 to <15 and ≥15) for the study eye will be summarized by treatment group and visit.

Additionally, the average LL-BCVA score over the last 3 months for each reporting period (Month 10 - Month 12 for the Month 12 reporting; Month 22 - Month 24 for the Month 24 reporting) will be calculated for each subject who has at least one LL-BCVA assessment in these windows. The observed values for the LL-BCVA and changes from baseline will be summarized by treatment group for the study eye. Summaries will present the descriptive statistics for baseline, absolute values and change from baseline data.

The same subgroup analyses as described for NL-BCVA in Section 6.3.5 will also be repeated for LL-BCVA for the full 24 months.

All LL-BCVA data will be listed for the ITT set. In addition, the low luminance deficit will be calculated as the difference between NL-BCVA and LL-BCVA and listed for the ITT set.

6.4.2. Total Area of GA lesion(s) at each planned assessment

The change from baseline at each planned assessment (other than Month 12 and Month 24) in the total area of GA lesion(s) in the study eye, as assessed by FAF, will be estimated from the MMRM analyses described in Section 6.2.2.

6.4.3. Monocular critical print size

Critical print size will be calculated as the smallest print size which support reading speed at 80% of the maximum reading speed, without adjustment for reading inaccuracy (Baskaran et al. 2019).

Monocular critical print size of the study eye will be summarized and analyzed in a similar fashion as the primary efficacy endpoint using MMRM described in Section 6.2.2. The model will include treatment (PM, PEOM, Sham), presence of CNV in the fellow eye (yes or no), and baseline GA lesion area (< 7.5 mm^2 or ≥ 7.5 mm^2) as fixed effects; baseline monocular critical print size as well as the chart type (MNRead or Radner) as covariates, time (in months) as a factor; the time × treatment interaction term as well as the baseline monocular critical print size × time interaction term.

The observed values for the monocular critical print size will be summarized by treatment group and visit for both the study and the fellow eye. Summaries will present the descriptive statistics for baseline, absolute values and change from baseline data.

To assess the potential effect of different assessment tools, the analysis of monocular critical print size will be repeated by chart type.

All monocular critical print size data will be listed for the ITT set.

6.4.4. NEI VFQ-25 Distance Activity Subscale Score

NEI VFQ-25 distance activity subscale score will be calculated per the scoring algorithms in user's manual.

NEI VFQ-25 distance activity subscale score will be summarized and analyzed in a similar fashion as the primary efficacy endpoint using MMRM described in Section 6.2.2. The model will include treatment (PM, PEOM, Sham), presence of CNV in the fellow eye (yes or no), and baseline GA lesion area (< 7.5 mm^2 or ≥ 7.5 mm^2) as fixed effects; baseline NEI VFQ-25 distance activity subscale score and baseline study eye status (Better-seeing Eye vs. Worse-seeing Eye) as covariates, time (in months) as a factor; as well as the time × treatment interaction term as well as the baseline NEI VFQ-25 distance activity subscale score × time interaction term.

The observed values for the NEI VFQ-25 distance activity subscale score will be summarized by treatment group and visit. Summaries will present the descriptive statistics for baseline, absolute values and change from baseline data.

The categorical changes (improvements of >5 units and minimal/no change (-5 to 5), worsening of a decrease of >5 units) will be summarized by treatment group and visit.

In addition, the shift from baseline in NEI VFQ-25 distance activity subscale score categories (>80, >65 - ≤80, >50 - ≤65, ≥35 - ≤50, <35) will be summarized by treatment group and visit.

All NEI VFQ-25 data will be listed for the ITT set.

6.4.5. Macular Functional Response based on Microperimetry

The number of scotomatous points will be determined from the mesopic microperimetry as an assessment of the macular functional response.

Number of scotomatous points in the study eye will be summarized and analyzed in a similar fashion as the primary efficacy endpoint using MMRM described in Section 6.2.2. The model will include treatment (PM, PEOM, Sham), presence of CNV in the fellow eye (yes or no), and baseline GA lesion area (< 7.5 mm^2 or ≥ 7.5 mm^2) as fixed effects; baseline number of

scotomatous points as a covariate, time (in months) as a factor; the time × treatment interaction term as well as the number of scotomatous points × time interaction term.

The observed values for number of scotomatous points will be summarized by treatment group and visit for both the study and the fellow eye. Summaries will present the descriptive statistics for baseline, absolute values and change from baseline data.

The mean change from baseline for number of scotomatous points in the study eye ± the standard error as well as the LS mean of the change from baseline for number of scotomatous points in the study eye ± the standard error will be plotted over time by treatment group.

The analysis described above will also be repeated for the change from baseline in the mean threshold sensitivity of perilesional points over time.

Data not of acceptable quality for the main analysis, as identified by the reading center as per the Imaging Charter, will not be included in these analyses.

6.5. Multiplicity Adjustment

No hypothesis testing of the (non-key) secondary endpoints will be performed. Control of Type I error for (non-key) secondary endpoints is not applicable.

6.6. Analyses of Exploratory Endpoints

The exploratory efficacy endpoints are listed in Section 2.2.4. At the time of Month 12 reporting, all exploratory efficacy endpoints will be evaluated based on data from baseline to Month 12 (see Section 3.5). For the Month 12 analyses, post-baseline visits, up to and including Month 12, will be included. At the time of the final study analysis, all exploratory efficacy endpoints will be evaluated based on data from baseline to Month 24. For the Month 24 analyses, post-baseline visits, up to and including Month 24, will be included.

Unless otherwise specified, all exploratory efficacy endpoints will be analyzed for the mITT set.

6.6.1. Other Microperimetry Endpoints

In addition to the analyses performed in Sections 6.3.2.1, 6.3.2.4, and 6.4.5, the following analyses will be performed for microperimetry.

- Change from baseline in the fixation stability (95% BCEA) over time.
- Change from baseline in the mean threshold sensitivity of paralesional points over time.
- Change from baseline in the mean threshold sensitivity of extralesional points over time.
- Number of points with a clinically significant progression (number of points with a ≥ 4 dB decrease from baseline for points ≥3 dB at baseline) over time.

These endpoints except for the last endpoint in the study eye will be summarized and analyzed in a similar fashion as the primary efficacy endpoint using MMRM described in Section 6.2.2. The model will include treatment (PM, PEOM, Sham), presence of CNV in the fellow eye (yes or no), and baseline GA lesion area (< 7.5 mm^2 or ≥ 7.5 mm^2) as fixed effects; the baseline

value of the endpoint as a covariate, time (in months) as a factor; as well as the time × treatment interaction term as well as the baseline value of the endpoint × time interaction term.

The observed values for the endpoints will be summarized by treatment group and visit for both the study and the fellow eye. Summaries will present the descriptive statistics for baseline, absolute values and change from baseline data.

The mean change from baseline for each endpoint in the study eye ± the standard error as well as the LS mean of the change from baseline for each endpoint in the study eye ± the standard error will be plotted over time by treatment group.

Data not acceptable quality for the main analysis as identified by the reading center per the Imaging Charter, will not be included in these analyses.

6.6.2. NEI VFQ-25 and NEI VFQ-39 Additional Analyses

Descriptive summary statistics for baseline, absolute values and change from baseline data will be provided by treatment group and visit for the following NEI VFQ-25 and NEI VFQ-39 scores:

- NEI VFQ-39 distance activity subscale
- NEI VFQ-25 composite score
- NEI VFQ-39 composite score
- NEI VFQ-25 near activity subscale score
- NEI VFQ-39 near activity subscale score
- NEI VFQ-25 driving subscale score (for subjects who are currently driving at baseline)

The shift from baseline in NEI VFQ score categories (>80, >65 - ≤80, >50 - ≤65, ≥35 - ≤50, <35) will be summarized by treatment group and visit for the scores described above.

In addition, the categorical changes (improvements of >5 units and minimal/no change (-5 to 5), worsening of a decrease of >5 units) will be summarized by treatment group and visit for the following NEI VFQ-25 Scores: Composite and Near Activity.

For the NEI VFQ-25 Driving subscale score, the number and percentage of subjects with a worsening driving outcome compared to baseline will be summarized by treatment group and visit. Each visit will display the number of patients who were driving at baseline with at least one of the worsening driving outcomes. The categories to determine worsening driving outcomes are: conversion to no longer currently driving, increase in difficulty driving in familiar places in daytime, increase in difficult driving at night or stopped doing this due to vision, increase in difficulty driving in difficult conditions or stopped doing this due to vision.

All NEI VFQ-25 data will be listed for the ITT set.

6.6.3. Binocular Maximum Reading Speed and Critical Print Size

Descriptive summary statistics for baseline, absolute values and change from baseline data will be provided by treatment group and visit for the binocular maximum reading speed and critical

print size with no adjustment for reading inaccuracy. All binocular maximum reading speed and critical print size data will be listed for the ITT set.

Categorical changes over time and categorical shifts from baseline will be presented, as will be done for the monocular reading speed.

6.6.4. GA Lesion (s) Study Eye to Fellow Eye Comparisons

Descriptive summary statistics will be provided by treatment and visit for the change from baseline in total area of GA Lesion(s) for the study eye as well as for the fellow eye in subjects with bilateral GA with fellow eyes that satisfy the following characteristics at baseline:

- Absence of CNV in the medical history
- Baseline GA lesion size between 2.5 and 17.5 mm^2
- Presence of any pattern of hyperautofluorescence in the junctional zone of GA
- GA not confluent with any peripapillary atrophy

The change from baseline in total area of GA Lesion(s) ± the standard error in the study eye as well as the fellow eye will be plotted over time by treatment group for the subjects with bilateral GA with fellow eyes that satisfy the above characteristics.

6.6.5. Digital Reading Index and Visual Function Application

The digital reading index will only be analyzed at the Month 24 reporting while the digital visual functional application will be analyzed at both the Month 12 and Month 24 reporting.

Descriptive summary statistics for baseline, absolute values and change from baseline data to Month 12 and Month 24 will be provided by treatment group for digital reading index.

Additionally, the average digital reading index score over the last 3 months for each reporting period (Month 10 - Month 12 for the Month 12 reporting; Month 22 - Month 24 for the Month 24 reporting) will be calculated for each subject who has at least one digital reading index assessment in these windows. The observed values for the digital reading index and changes from baseline will be summarized by treatment group. Summaries will present the descriptive statistics for baseline, absolute values and change from baseline data.

Descriptive summary statistics for baseline, absolute values and change from baseline data to Month 12 and Month 24 will be provided by treatment group for digital visual function.

Additionally, the average digital visual function score over the last 3 months for each reporting period (Month 10 - Month 12 for the Month 12 reporting; Month 22 - Month 24 for the Month 24 reporting) will be calculated for each subject who has at least one digital visual function assessment in these windows. The observed values for the digital visual function and changes from baseline will be summarized by treatment group. Summaries will present the descriptive statistics for baseline, absolute values and change from baseline data.

6.6.6. Genetic Polymorphisms

The genetic polymorphisms data will be analyzed and reported in a separate report and briefly summarized in the clinical study report (CSR). The genetic polymorphisms data will be listed for the genotyping set.

6.6.7. Dry AMD Analysis

Biomarkers of early and intermediate stage of AMD outside the GA area will also be evaluated in this study. The number and percentage of subjects with at least one lesion (up to 5) area in the study eye classified as iRORA will be summarized at baseline by treatment group in the mITT set. The number and percentage of subjects with a progression from iRORA to cRORA based on SD-OCT in the study eye will be summarized over time by treatment group. A CMH test with the number of baseline iRORA lesions (0, 1, 2, 3, 4, ≥ 5), presence of CNV in the fellow eye (yes or no), and baseline GA lesion area (< 7.5 mm^2 or ≥ 7.5 mm^2) as the stratification factors will be presented for each post-baseline visit. If necessary due to sparse cells, an unadjusted analysis may be performed. In addition, the percentage of baseline lesions with a progression will also be presented over time by treatment group.

The above summary and analysis will also be repeated in those subjects with at least one lesion in the study eye classified as large drusen at baseline and whether these progressed to iRORA or cRORA in the study eye. Large drusen is defined as a drusen that is greater than or equal to 40 microns in height on SD-OCT.

This analysis will not be conducted at the Month 12 reporting and will only be conducted for the final study reporting.

All dry AMD data will be listed for the ITT set.

6.6.8. Analyses of Other Ophthalmology Efficacy Imaging Data

In addition to the GA lesion(s) size data from the FAF, analysis of additional efficacy parameters will be performed from this imaging instrument for the mITT set. The following summaries will be performed separately by study eye and by fellow eye and will be presented by treatment group.

- Descriptive summary statistics for baseline, absolute values and change from baseline data for the distance of GA lesion from atrophy junction to the fovea in study eyes with non-subfoveal involvement of the GA lesion at baseline (assessed from FAF). For the fellow eye, this will be presented for fellow-eyes with non-subfoveal involvement of the GA lesion at baseline. Non-subfoveal involvement is defined as distance of the atrophy junction to the fovea > 0.
- Conversion from non-subfoveal involvement to subfoveal involvement of the GA lesion at each visit (assessed from FAF). This will include only study eyes with non-subfoveal involvement for GA lesions at baseline. For the fellow eye, this should be presented for fellow eyes with non-subfoveal involvement at baseline. This will be presented as a cumulative incidence at each scheduled visit.

All FAF data will be listed for the ITT set.

7. SAFETY ANALYSES

Safety analyses will be performed using the Safety set. Safety variables include adverse events (AEs), deaths, clinical laboratory results, vital signs, incidence of ADA against pegcetacoplan, ocular assessments (e.g., NL-BCVA, IOP and ophthalmic examinations), and ocular imaging.

At the time of the Month 12 reporting, safety summaries will be produced based on the complete Month 12 data. At the time of the final study reporting, safety summaries will be produced based on cumulative data.

For each safety variable, the last value collected before the first dose of investigational product will be used as baseline for all analyses of that safety variable. Last Observed Value (LOV) will be defined as the last valid assessment obtained after baseline. The LOV will be presented for the Month 12 reporting as well as the final study reporting.

All safety analyses will be conducted according to the actual treatment the subject received. Unless otherwise specified, all safety summaries will be performed on the Safety set.

7.1. Adverse Events

AEs will be coded using the MedDRA version 23.1 and summarized separately for those occurred in the study eye, those in the fellow eye, and the non-ocular events.

An AE will be considered a TEAE if it has a start date on or after the first dose of investigational product or if it has a start date before the date of the first dose of investigational product but increases in severity on or after the date of the first dose of investigational product.

An overall summary of the number of subjects with TEAEs will be presented, including the number and percentage of subjects with:

- Any TEAEs
- TEAEs related to study drug (evaluated by the investigator as definitely related, possibly related)
- TEAEs related to study drug injection procedure
- All TEAEs by maximum severity
- Serious TEAEs
- Serious TEAEs related to study drug (evaluated by the investigator as definitely related, possibly related)
- Serious TEAEs related to study drug injection procedure
- Serious TEAEs by maximum severity
- TEAEs leading to interruption of study treatment
- TEAEs leading to discontinuation of study treatment
- TEAEs leading to study discontinuation
- TEAEs leading to death

This overall summary will also include the total number of TEAEs reported. This overall summary will also be summarized separately for those occurred in the study eye, those in the fellow eye, and the non-ocular events.

The number and percentage of subjects reporting TEAEs in each treatment group and overall will be tabulated by SOC and PT for all of the categories in the list above. If more than 1 AE occurs with the same preferred term for the same subject, then the subject will be counted only once for that preferred term using the most severe and most related occurrence for the summarization by severity and by relationship to investigational product.

Endophthalmitis and intraocular inflammation ocular TEAEs will be presented by PT for study eye and fellow eye.

Endophthalmitis and intraocular inflammation ocular TEAEs in the study eye will also be presented on a per-injection basis (i.e., number of events/total number of injections given).

All SOC and PT summaries will be ordered alphabetically by SOC and within SOC, by descending order of the total number of subjects in the PM, PEOM, and Sham groups combined.

Non-ocular AEs, ocular AEs in the study eye and ocular AEs in the fellow eye will be listed separately. This presentation will be repeated for serious AEs. Additionally, separate listings for deaths, AEs leading to discontinuation of study, AEs leading to discontinuation of study drug, and AEs due to COVID-19 will also be generated.

7.1.1. TEAEs of New Onset Exudative AMD

For subjects who experience a TEAE of “Choroidal neovascularization” or “Neovascular AMD” (“Exudative AMD TEAE”), the following summaries will be performed:

- The overall summary table described in Section 7.1 will be repeated replacing “TEAEs” with “Exudative AMD TEAEs”. This will be reported for the study eye.
- The incidence of Exudative AMD TEAEs in the study eye will be presented by treatment group and overall. In addition, the analysis will be repeated by fellow eye CNV status at baseline and study eye DLS on SD-OCT at baseline. A similar analysis will be repeated for the Exudative AMD TEAEs in the fellow eye with an additional analysis by fellow eye DLS on SD-OCT at baseline. Subjects with a medical history of CNV in the corresponding eye will be excluded from the analysis.
- The incidence and rate per 100 subject-years of Exudative AMD TEAEs in the study and fellow eye as well as a summary (for study and fellow eye) and Kaplan Meier plot for the time to development of Exudative AMD TEAEs in the study eye will be presented by treatment group and overall. Subjects with a medical history of CNV in the corresponding eye will be excluded from the analysis.
- In addition, the characteristics of the Exudative AMD TEAEs in the study eye will be presented at baseline, study visit preceding exudation, study visit at exudation and at Month 12 (or Month 24 if the final analysis) for characteristics based on SD-OCT and baseline, study visit at exudation and at Month 12 (or Month 24 if the final analysis) for characteristics based on Fluorescein Angiography (FA). The following characteristics will be presented by treatment group and overall:

- Presence of cystoid spaces on SD-OCT
- Central (center point) retinal thickness on SD-OCT
- Central subfield thickness on SD-OCT
- Presence of CNV on FA.
- CNV type on FA
- Presence of subretinal fluid on SD-OCT

- A summary of concomitant anti-VEGF therapy for new exudative AMD in the study eye will be provided by treatment group and overall. This summary will include the total number of subjects that received any anti-VEGF for new exudative AMD as well as the average number of injections per month post exudative AMD. There will also be a breakdown by preferred term of ranibizumab or aflibercept and the average number of injections per month for each term. Subjects will be presented by the number of injections received for that medication.
- BCVA scores at the baseline visit, study visit preceding exudation, the study visit at the exudation, and at Month 12 (or Month 24 if the final analysis) will be presented for all subjects with an Exudative AMD TEAE in the study eye by treatment group and overall. In addition, the change in BCVA scores from baseline, the visit prior to the exudation, and the exudation visit will be presented where appropriate.

A listing of Exudative AMD TEAEs and baseline characteristics including fellow eye CNV status, study eye double layer sign and study eye MNV presence, a listing of the characteristics of the new Exudative AMD TEAEs, anti-VEGF exposure, and the BCVA values for subjects with the new Exudative AMD TEAEs will be provided.

7.2. Clinical Laboratory Data

All laboratory parameters collected at each center’s local laboratory will be normalized by converting values in original units to values in SI units and classified as normal, low, or high based on normal ranges supplied by the local laboratories and upon employing standardization.

Observed and change from baseline clinical laboratory data (hematology, chemistry and urinalysis) will be summarized by treatment group and protocol specified time points for the following clinical laboratory variables.

**Hematology**	Hemoglobin, hematocrit, red blood cells (RBC), platelet count, white blood cell count (WBC) – total and differential.
**Biochemistry**	Aspartate transaminase, alanine transaminase, alkaline phosphatase, sodium, potassium, chloride, bicarbonate, blood urea nitrogen (BUN), bilirubin (total, direct and indirect), creatinine, creatine kinase, glucose, and albumin.
**Urinalysis**	Glucose, specific gravity, blood, ketones, protein, bilirubin, pH, urobilinogen, leukocyte esterase, and nitrite.

Clinical laboratory test values are potentially clinically significant (PCS) if they meet either the low or high PCS criteria listed in Table 3. The number and percentage of subjects with baseline and/or post-baseline PCS values will be tabulated by treatment group and overall.
The percentages will be calculated relative to the number of subjects with available values for

each lab parameter in each treatment group at the analysis visit. The numerator is the total number of subjects with at least 1 PCS value at the analysis visit. A supportive listing of subjects with post-baseline PCS values will be provided including the subject number, site, baseline, and post-baseline values.

Table 3: Criteria for Potentially Clinically Significant Laboratory Tests

Parameter	SI Unit	Lower Limit	Higher Limit
**Biochemistry**			
Albumin	g/L	<25 g/L	
Alanine Aminotransferase (ALT)	U/L		≥3*ULN
Alkaline Phosphatase (ALP)	U/L		≥3*ULN
Aspartate Aminotransferase (AST)	U/L		≥3*ULN
Bilirubin, Direct	umol/L		>1.5*ULN
Bilirubin, Total	umol/L		>1.5*ULN
Bicarbonate	mmol/L	<LLN	
Blood Urea nitrogen	mmol/L		>2.5*ULN
Chloride	mmol/L	<90 mmol/L	>115mmol/L
Creatinine	umol/L		≥178.8 umol/L
Creatine kinases	U/L		≥3*ULN
Glucose	mmol/L	<3.05 mmol/L	≥8.88 mmol/L
Potassium	mmol/L	<3.5 mmol/L	>5.5 mmol/L
Sodium	mmol/L	<130 mmol/L	>150 mmol/L
**Hematology**			
Hemoglobin	g/L	<0.9*LLN	
Hematocrit	%	<0.9*LLN	
Platelet Count	10^9/L	<LLN	
**Urinalysis**			
Specific Gravity		<0.9*LLN	>1.1*ULN
pH		<LLN	
Protein			>2+
Glucose			>1+
Blood			>2+

LLN: Lower limit of normal value provided by the laboratory
ULN: Upper limit of normal value provided by the laboratory

Descriptive statistics will be used for continuous data and frequency counts and percentages for categorical data. A shift table from baseline, by treatment group and protocol specified time points, of normal, abnormal low, and abnormal high records will also be summarized for hematology and chemistry data with marginal totals using frequency counts and percentages.

All laboratory data will be listed for the safety set.

7.3. Vital Signs

Descriptive statistics for vital signs (e.g., systolic and diastolic blood pressure, respiratory rate, temperature, and pulse rate) and their changes from baseline at each post-baseline visit will be presented by treatment group and overall.

Vital sign values will be considered PCS if they meet both the observed value criteria and the change from baseline criteria listed in Table 4. The number and percentage of subjects with PCS baseline and/or post-baseline values will be tabulated by treatment group and overall. The percentages will be calculated relative to the number of subjects with values for each vital sign parameter in each treatment group at the analysis visit. The numerator is the total number of subjects with at least 1 PCS vital sign value at the analysis visit. A supportive listing of subjects with post-baseline PCS values will be provided including the subject number, site, baseline, and post-baseline PCS values.

Table 4: Criteria for Potentially Clinically Significant Vital Signs

Vital Sign Parameter	Flag	Criteria[a]	
		Observed Value	Change from Baseline
Systolic blood pressure (mmHg)	High	≥180	Increase of ≥20
	Low	≤90	Decrease of ≥20
Diastolic blood pressure (mmHg)	High	≥105	Increase of ≥15
	Low	≤50	Decrease of ≥15
Pulse rate (beats per minute)	High	≥120	Increase of ≥15
	Low	≤50	Decrease of ≥15

[a] A post-baseline value is considered as a PCS value if its meets both criteria for observed value and change from baseline.

All vital signs data will be listed for the safety set.

7.4. Physical Examination Findings

Any relevant findings from the baseline physical examination are to be reported on the medical history. Any relevant findings from the post-baseline physical examination are to be reported as AE's. No separate physical examination findings were collected to summarize.

7.5. Complete Ophthalmic Exam

Results of the ocular assessments will be summarized by shift tables showing the shift from baseline in the individual slit-lamp examination and indirect ophthalmoscopy endpoint. These will be provided by treatment group, overall and visit for study eye and fellow eye. The visits to be presented are Month 12, Month 24, and LOV.

Any relevant findings from the post-baseline ophthalmic examination are to be reported as AEs.

The change from baseline in chronic (first measurement of the day) IOP will be summarized over time by treatment group. In addition, the incidence of IOP above specified thresholds (i.e., >21 mmHg at pre-injection/chronic (first measurement of the day); >30 mmHg post injection) will be summarized by treatment group and overall, protocol specified timepoint (at Month 12 and Month 24 readout, respectively) and over the whole study.

The mean change from baseline in the chronic IOP ± the standard error will be plotted over time by treatment group.

Details of the ocular assessments will be provided in listings for the safety set and for IOP, the ITT set.

7.6. NL-BCVA

The number and percentage of subjects who lost letters compared with baseline at each visit based on NL-BCVA in the study and fellow eye will be presented. The three categories are >=15, ≥15- < 30 and ≥30 ETDRS letters.

7.7. Other Ophthalmology Imaging Assessments

Unless otherwise stated, ocular imaging data collected will be listed by treatment group and overall for the Safety set. The summary will be performed separately by study eye and by fellow eye. The ophthalmology imaging assessments performed in this study include the following:

- Digital Color Fundus Photographs (DCFP)
  - Shift from baseline in the presence/absence of hemorrhage at each visit
- FA
  - The following analyses will be performed in all study eyes and in non-study eyes without a history of CNV at baseline (in medical history) in the corresponding eye.
    - Incidence of new CNV. This will be summarized as the cumulative number of CNV events by each visit and the cumulative incidence by that visit based on Kaplan Meier methods.
    - Incidence of new CNV by type (classic, occult, classic & occult, disciform scar) at first emergence will be summarized at each visit for the cumulative number of events.
    - Total CNV size (diameter) at first emergence will be summarized at each visit for the cumulative number of events.
- SD-OCT
  - Descriptive statistics on central subfield thickness at each visit
  - Plot of the mean change from baseline in central subfield thickness in the study eye ± the standard error over time by treatment group.
  - Descriptive statistics on central (center point) retinal thickness at each visit
  - Shift from baseline in the presence/absence of subretinal fluid at each visit
  - Shift from baseline in the presence/absence of cystoid spaces at each visit
- Specular Microscopy

  - Endothelial Cell Count is assessed using specular microscopy, and the change from baseline in cell density (cells/mm^2) as well as the change from baseline in average cell-size will be summarized with descriptive statistics.
- OCT-A
  - Incidence of subclinical CNV (referred to as MNV in the datasets) determined by OCT-A in eyes without previous CNV reported in the medical history or as an adverse event in the corresponding eye at each visit.

In addition, the following summary of suspected and reading-center confirmed CNV cases will be presented by treatment group. This will be presented separately for the study eye only.

- The number and percentage of subjects with an Exudative AMD TEAE on or before Month 12 (or Month 24 if the final analysis)
- The number and percentage of subjects with at least one suspected new onset CNV case submitted to the reading center by the Investigator post-baseline through Month 12 (or Month 24 if the final analysis)
- The number and percentage of subjects with a reading-center confirmed new onset CNV case (out of the suspected CNV cases submitted by the Investigator) post-baseline through Month 12 (or Month 24 if the final analysis). Reading-center confirmed CNV case is identified as either:
  - Presence of CNV on FA (Type = classic, occult, classic & occult, disciform scar) OR
  - Presence of CNV thickness on SD-OCT
- The number and percentage of subjects with a reading-center confirmed new onset CNV case (out of the suspected CNV cases submitted by the Investigator) post-baseline through Month 12 (or Month 24 if the final analysis) and also reported as an Exudative AMD TEAE.
- The number and percentage of subjects with a reading-center confirmed new onset CNV case (out of the suspected CNV cases submitted by the Investigator) post-baseline through Month 12 (or Month 24 if the final analysis) without an Exudative AMD TEAE ever reported.
- The number and percentage of subjects with at least one suspected CNV case, confirmed by the reading-center but previously reported as an Exudative AMD TEAE.
- The number and percentage of subjects with at least one suspected CNV case, not confirmed by the reading-center but still reported as an Exudative AMD TEAE.
- The number and percentage of subjects with a reading-center finding of presence of new-onset CNV (defined in the same way as a confirmed new onset CNV case) without any other submission to the reading center of a suspected case post-baseline through Month 12 (or Month 24 if the final analysis) and also reported as an Exudative AMD TEAE on or before Month 12 (or Month 24 if the final analysis)

- The number and percentage of subjects with a reading-center finding of presence of new-onset CNV (defined in the same way as a confirmed new onset CNV case) without any other submission to the reading center of a suspected case post-baseline through Month 12 (or Month 24 if the final analysis) and no report of an Exudative AMD TEAE.
- The number and percentage of subjects with Exudative AMD TEAE and without a suspected case submitted to the reading center as well as without a reading center finding of new-onset CNV (defined in the same way as a confirmed new onset CNV case).

7.8. Immunogenicity

Immunogenicity data will be listed separately for anti-pegcetacoplan peptide antibody and anti-PEG antibody results. The number and percentage of samples confirmed positive for ADA response will be summarized by treatment groups (i.e., PM, PEOM, and Sham). The number and percentage of subjects with treatment-emergent and treatment-boosted responses will be presented by treatment group. Treatment emergent response is defined as a confirmed positive antibody result postdose after a negative antibody result reported at baseline. Treatment-boosted response is defined as a ≥4-fold increase in titer from the baseline level.

7.9. Death

Subject deaths and primary cause of death will be summarized.

7.10. Other Safety Data

Urine pregnancy test will be provided in a listing.

8. PHARMACOKINETICS ANALYSIS

No pharmacokinetic analyses are planned for this study.

9. PHARMACODYNAMIC ANALYSES

No pharmacodynamic analyses are planned for this study.

10. OTHER ANALYSES

No other analyses are planned for this study.

11. INTERIM ANALYSIS

No formal interim analyses are planned for this study.

To support regulatory submissions, data cuts of the Month 12 – Month 24 data of the study may be taken prior to the final database lock and summarized prior to the final study reporting. The analysis of this data will not be used to modify the pre-planned analyses of this study.

12. DATA MONITORING COMMITTEE

An external, independent DMC reviews unmasked data across the conduct of the study approximately every 6 months. A charter for the DMC as well as a DMC statistical analysis plan was prepared separately.

13. DATA HANDLING CONVENTIONS

13.1. General Data Reporting Conventions

Continuous variables will be summarized using the number of non-missing observations (n), mean, SD, median, quartile 1, quartile 3, minimum, and maximum. Categorical variables will be summarized using frequencies and percentages. Unless stated otherwise, for all percentages, the number of subjects in the analysis population for the treatment group will be the denominator.

Unless otherwise specified, the estimated mean and median for a set of values should be printed out to 1 more decimal place than the original values, and standard deviations should be printed out to 2 more decimal places than the original values. The minimum and maximum should report the same number of decimal places as the original values. Percentages will be displayed with 1 decimal place; except percentages will not be presented when the count is zero and 100% will be presented as an integer.

13.2. Definition of Baseline

Unless stated otherwise, baseline will be defined as the last available pre-treatment value taken on or before the first dose date of study drug, and will be used for summary of baseline characteristics, as well as for all change-from-baseline analyses of efficacy and safety endpoints.

13.3. Definition of Relative Study Days

Unless otherwise noted, relative study days (Rel Days) of an evaluation are defined as number of days relative to the first dose date of study drug which is designated as Day 1, and the preceding day is Day -1, the day before that is Day -2, etc.

If evaluation date is on or after first dose date, then relative study days are calculated as

Evaluation date - first dose date of study drug + 1.

If evaluation date is before first dose date, then relative study days are calculated as

Evaluation date - first dose date of study drug

Relative study days take negative values if evaluation date occurs prior to first dose date and take positive values if evaluation date occurs on or after first dose date of study drug.

13.4. Definition of Visit Windows

13.4.1. All Assessments Except Microperimetry

All assessments occurring on or before the first date of dosing (Analysis Study Day (ADY) ≤ 1) will be assigned to the Baseline analysis visit window.

Unless otherwise specified, the actual scheduled nominal post-baseline visit will be used for over time summaries. Post-baseline unscheduled visits and early termination visits will be mapped to a scheduled visit and will only be used in the analysis if the nominal scheduled visit result is missing, Table 5 and Table 6 presents the analysis visit window mapping for unscheduled and early term visits. In the case that multiple unscheduled or early termination visits are in the same analysis window, the one closest to the target date will be used. In the event that windowed visit

is mapped to an illogical sequence of visits when considering nearby scheduled visits (i.e., windowed visit is higher than the subsequent visit or lower than the preceding visit), the windowed visit will be set to the logical scheduled visit.

Table 5: Post-Baseline Analysis Visit Window for Unscheduled and Early Termination Visits: All Assessments Except Microperimetry: Monthly Regimen

Analysis Visit	Target Study Day	Analysis Window (days)
Month 1	30	2 to 45
Month 2	60	46 to 75
Month 3	90	76 to 105
Month 4	120	106 to 135
Month 5	150	136 to 165
Month 6	180	166 to 195
Month 7	210	196 to 225
Month 8	240	226 to 255
Month 9	270	256 to 285
Month 10	300	286 to 315
Month 11	330	316 to 345
Month 12	360	346 to 375
Month 13	390	376 to 405
Month 14	420	406 to 435
Month 15	450	436 to 465
Month 16	480	466 to 495
Month 17	510	496 to 525
Month 18	540	526 to 555
Month 19	570	556 to 585
Month 20	600	586 to 615
Month 21	630	616 to 645
Month 22	660	646 to 675
Month 23	690	676 to 705
Month 24	720	706 to EOS

Table 6: Post-Baseline Analysis Visit Window for Unscheduled and Early Termination Visits: All Assessments Except Microperimetry: Every Other Month Regimen

Analysis Visit	Target Study Day	Analysis Window (days)
Month 1	30	2 to 45
Month 2	60	46 to 75
Month 3	90	76 to 105
Month 4	120	106 to 135
Month 5	150	136 to 165
Month 6	180	166 to 195
Month 7	210	196 to 225
Month 8	240	226 to 255
Month 9	270	256 to 285
Month 10	300	286 to 315
Month 11	330	316 to 345
Month 12	360	346 to 375
Month 14	420	376 to 450
Month 16	480	451 to 510
Month 18	540	511 to 570
Month 20	600	571 to 630
Month 22	660	631 to 690
Month 24	720	690 to EOS

13.4.2. Microperimetry Assessments

All assessments occurring on or before the first date of dosing (Analysis Study Day (ADY) ≤ 1) will be assigned to the Baseline analysis visit window.

Unless otherwise specified, the actual scheduled nominal post-baseline visit will be used for over time summaries unless a better assessment, as determined by the reading center, is within the analysis window. Post-baseline unscheduled visits and early termination visits will be mapped to a scheduled visit and will only be used in the analysis if the nominal scheduled visit result is missing, not suitable for analysis by the reading center, or the assessment was assessed as better than the nominal scheduled visit by the reading center, Table 7 presents the analysis visit window mapping for unscheduled and early term visits. In the case that multiple unscheduled or early termination visits are in the same analysis window, the one closest to the target date will be used. In the event that windowed visit is mapped to an illogical sequence of visits when considering nearby scheduled visits (i.e., windowed visit is higher than the subsequent visit or lower than the preceding visit), the windowed visit will be set to the logical scheduled visit.

Table 7: Post-Baseline Analysis Visit Window for Unscheduled and Early Termination Visits: Microperimetry Assessments

Analysis Visit	Target Study Day	Analysis Window (days)
Month 6	180	2 to 270
Month 12	360	271 to 450
Month 18	540	451 to 630
Month 24	720	631 to EOS

13.5. Derived Efficacy Endpoints

13.5.1. GA Lesion Size

For all independent ophthalmic assessments (performed by DARC laboratory), which includes GA lesion size, if assessments by only two independent readers are available, then the median of the 2 readings will be used in the calculation of summary statistics. If assessments by three independent readers are available, then the median of the three readings will be used for the summary calculations.

In the case that the GA lesion size has an indeterminate boundary noted, then the lesion size for this indeterminate boundary will be used in all analyses of GA lesion size unless otherwise noted in the case that primary lesions size assessment for an individual reader is missing.

13.5.2. MNRead Reading Chart

13.5.2.1. Reading Speed

13.5.2.1.1. Without adjustment for reading inaccuracy

Any print size with "Not Done" will have the associated wpm marked as 0.

Reading speed (wpm) = 600 / (reading time in seconds)

If reading speed >300 wpm, reading speed = 300.

13.5.2.1.2. With adjustment for reading inaccuracy

Any print size with "Not Done" will have the associated wpm marked as 0.

Reading speed (wpm) = 60 (10 – errors) / (reading time in seconds)

If reading speed >300 wpm, reading speed = 300.

If 10 or more errors were made in a sentence (implying wpm<0), then the reading speed for that sentence can be assumed to be zero (wpm).

13.5.2.2. Maximum reading speed

Maximum reading speed will be calculated as the mean of the three highest nonzero reading speeds (or 2 or 1 if that is all that exists). For subjects having all wpm values = 0, the maximum reading speed will be set to 0, and for those same visits the critical print size will be set to missing. Reading speed will equal 0 wpm at each print size for which (time=0) or Not Done (indicating no words in the sentence could not be read or sentence was not attempted due to vision) is checked for that print size.

13.5.2.3. Critical Print Size

Critical print size will be calculated as the smallest print size which support reading speed at 80% of the maximum reading speed. If all wpm at all print sizes = 0 at a visit then that visit's critical print size will be missing. Print size will be adjusted for viewing distance as follows:

- MNRead Charts:
  - For all viewing distances:
    - Print size = Print size + correction, where correction=log10 [40 / (viewing distance in cm)], rounded to two decimal places

Note: the CRFs for the MNRead charts have print sizes pre-filled for a 40 cm viewing distance, while the Radner charts have print sizes pre-filled for a 32 cm viewing distance.

13.5.2.4. Reading acuity

Reading acuity will be calculated at each visit as: 1.4 – (# sentences read x 0.1) + (# of words read incorrectly x 0.01). This variable will not be included in the CSR.

13.5.3. Radner Reading Chart

13.5.3.1. Reading Speed

13.5.3.1.1. Without adjustment for reading inaccuracy

Reading Speed (wpm) = 14 / (time in seconds) x 60 = 840 / (time in seconds)

If reading speed >300 wpm, reading speed = 300.

13.5.3.1.2. With adjustment for reading inaccuracy

Reading Speed (wpm) = (14 – errors)/ (time in seconds) x 60

If 14 or more errors were made in a sentence (implying wpm<0), then the reading speed for that sentence can be assumed to be zero (wpm).

If reading speed >300 wpm, reading speed = 300.

13.5.3.2. Maximum Reading Speed

Maximum reading speed will be calculated as the mean of the three highest nonzero reading speeds (or 2 or 1 if that is all that exists). For subjects having all wpm values = 0, the maximum reading speed will be set to 0, and for those same visits the critical print size will be set to missing. Reading speed will equal 0 wpm at each print size for which (time=0) or Not Done (indicating no words in the sentence could not be read or sentence was not attempted due to vision) is checked for that print size.

13.5.3.3. Critical Print Size

Critical print size will be calculated as the smallest print size which support reading speed at 80% of the maximum reading speed. If all wmp at all print sizes = 0 at a visit then that visit's critical print size will be missing. Print size needs to be adjusted for viewing distance as follows:

- Radner Charts:
  - For viewing distance of 32 cm
    - No correction is needed.

- For viewing distance of other than 32 cm:
  - Print size= Print size – 0.1 + correction, where correction=log10 [40 / (viewing distance in cm)], rounded to two decimal places

Note: the CRFs for the MNRead charts have print sizes pre-filled for a 40 cm viewing distance, while the Radner charts have print sizes pre-filled for a 32 cm viewing distance.

13.5.3.4. Reading Acuity

Reading acuity will be calculated at each visit as: 1.4 – (# sentences read x 0.1) + (# of words read incorrectly x 0.01). This variable will not be included in the CSR.

13.5.4. Mean FRI Index Score and FRI Level

Follow Section 4.3.2 of FRI Index User Manual (Version 1.4).

SAS code for scoring FRI Index and FRI Level is available in Appendix C of FRI Index Under Manual (Version 1.4).

13.5.5. NL-BCVA and LL-BCVA

13.5.5.1. BCVA Letter Score

- If the 4-meter score is >19 letters read correctly, the visual acuity score is the sum of total letters correctly read at 4 meters plus the addition of 30.
- If the 4-meter score is ≤19 letters read correctly, the visual acuity score is the sum of total letters read correctly at 4 meters and total letters read correctly at the 1-meter distance.
- If no letters are read correctly at either the 4-meter distance or the 1-meter distance, the visual acuity score is 0.

13.5.5.2. Conversion of BCVA Letter Score to Snellen Equivalent

Conversions between letter, logMAR, and Snellen visual acuity scores are available in Beck et al. (2003) and Holladay et al. (2004). Letter scores are converted to logMAR equivalents using the formula logMAR = 1.7 – (.02)(letter score). With this conversion, a 5-letter difference in visual acuity is equivalent to a difference of 0.1 logMAR and to one Snellen line. Conversion from logMAR to Snellen can be done after rounding logMAR values to one decimal place following Table 1 in Holladay et al. (2004).

13.5.6. Low Luminance Deficit

LLD = NL-BCVA - LL-BCVA

13.5.7. NEI VFQ-25 scores

Scoring VFQ-25 with or without optional item is a two-step process:

- First, original numeric values from the survey are re-coded following the scoring rules outlined in Table 2 of user's manual. All items are scored so that a high score represents better functioning. Each item is then converted to a 0 to 100 scale so that the lowest and highest possible scores are set at 0 and 100 points, respectively. In this format, scores represent the achieved percentage of the total possible score, e.g., a score of 50 represents 50% of the highest possible score.
- In step 2, items within each sub-scale are averaged together to create the 12 sub-scale scores, Table 3 of user's manual indicates which items contribute to each specific sub-scale. Items that are left blank (missing data) are not taken into account when calculating the scale scores. Sub-scales with at least one item answered can be used to generate a sub-scale score. Hence, scores represent the average for all items in the sub-scale that the respondent answered.

13.5.7.1. NEI VFQ-25 Distance Activity Subscale Score

Average of items 8, 9, and 14

13.5.7.2. NEI VFQ-25 Near Activity Subscale Score

Average of items 5, 6, and 7

13.5.7.3. NEI VFQ-25 Driving Subscale Score

For subjects who are currently driving at baseline only. Average of items 15c, 16, 16a

13.5.7.4. NEI VFQ-25 Composite Scores

To calculate an overall composite score for the VFQ-25, simply average the vision-targeted sub-scale scores, excluding the general health rating question.

13.5.8. NEI VFQ-39 Scores

For subjects responded to additional questions A1-A13, VFQ-39 subscale scores can be calculated using Table 4 of user's manual.

13.5.8.1. NEI VFQ-39 Distance Activity Subscale Score

Average of items 8, 9, 14, A6, A7, and A8

13.5.8.2. NEI VFQ-39 Near Activity Subscale Score

Average of items 5, 6, 7, A3, A4, and A5

13.5.8.3. NEI VFQ-39 Composite score

Calculated by simply averaging the vision related subscales, excluding the general health rating question.

13.5.9. Microperimetry

The number of points with a clinically significant progression by mesopic microperimetry will be calculated as the number of points with a ≥ 4 dB decrease from baseline for points >= 3 dB at baseline. Only data from images of acceptable quality for the main analysis, as determined by the reading center per the Imaging Charter, will be included in the analysis of microperimetry variables.

13.6. Repeated or Unscheduled Assessments of Safety Parameters

If a subject has repeated assessments before the start of investigational product, then the results from the final assessment made prior to the start of investigational product will be used as baseline. If end-of-study assessments are repeated or unscheduled, the last post-baseline assessment will be used as the end of study assessment for generating descriptive statistics. However, all post-baseline assessments will be used for PCS value determination and all assessments will be presented in the data listings.

13.7. Handling of Missing, Unused, and Spurious Data

13.7.1. Missing Date of Investigational Product

When the date of the last dose of investigational product is missing for a subject in the Safety set, all efforts should be made to obtain the date from the investigator.

13.7.2. Missing Date Information for Prior or Concomitant Medications (Therapies/Procedures)

If either the start or stop date of medication is missing, the worst or most conservative case will be considered when assigning medications to categories. So for a missing start date (where stop date is after date of first dose or missing) the date will be imputed as the date of first dose; for a missing stop date the date will be imputed as the last study date.

13.7.2.1. Incomplete Start Date

The following rules will be applied to impute the missing numerical fields. If the stop date is complete and the imputed start date is after the stop date, then the start date will be imputed using the stop date.

13.7.2.1.1. Missing Day and Month

If the year of the incomplete start date is the same as the year of the date of the first dose of investigational product, then the day and month of the date of the first dose of investigational product will be assigned to the missing fields.

If the year of the incomplete start date is before the year of the date of the first dose of investigational product, then December 31 will be assigned to the missing fields.

If the year of the incomplete start date is after the year of the date of the first dose of investigational product, then 01 January will be assigned to the missing fields.

13.7.2.1.2. Missing Month Only

The day will be treated as missing and both month and day will be replaced according to the above procedure.

13.7.2.1.3. Missing Day Only

If the month and year of the incomplete start date are the same as the month and year of the date of the first dose of investigational product, then the day of the date of the first dose of investigational product will be assigned to the missing day.

If either the year is before the year of the date of the first dose of investigational product or if both years are the same but the month is before the month of the date of the first dose of investigational product, then the last day of the month will be assigned to the missing day.

If either the year is after the year of the date of the first dose of investigational product or if both years are the same but the month is after the month of the date of the first dose of investigational product, then the first day of the month will be assigned to the missing day.

13.7.2.2. Incomplete Stop Date

The following rules will be applied to impute the missing numerical fields. If the imputed stop date is before the start date (imputed or non-imputed start date), then the imputed stop date will be equal to the start date.

13.7.2.3. Missing Day and Month

If the year of the incomplete stop date is the same as the year as of the last study date, then the day and month of the date of the last study date will be assigned to the missing fields.

If the year of the incomplete stop date is before the year of the last study date, then 31 December will be assigned to the missing fields.

If the year of the incomplete stop date is after the year of the last study date, then 01 January will be assigned to the missing fields.

13.7.2.3.1. Missing Month Only

The day will be treated as missing and both month and day will be replaced according to the above procedure.

13.7.2.3.2. Missing Day Only

If the month and year of the incomplete stop date are the same as the month and year of the last study date, then the day of the last study date will be assigned to the missing day

If either the year is before the year of the last study date or if both years are the same but the month is before the month of the last study date, then the last day of the month will be assigned to the missing day

If either the year is after the year of the last study date or if both years are the same but the month is after the month of the last study date, then the first day of the month will be assigned to the missing day.

13.7.3. Missing Date Information for Adverse Events

Events with missing or partial dates will be handled such that in the absence of contradictory information an AE is treatment emergent. So, for a missing start date (where stop date is after first dosing date or missing) the date will be imputed as the first dose date; for a missing stop date the date will be imputed as the last study date. If a partial date is recorded, the following convention will be used to assign the AE:

13.7.3.1. Incomplete Start Date

If a start date is missing the day information and month/year is the same as first dose date then use first dose date, else '01' will be used for the day; if a start date is missing the month and the year is the same as first dose date then use first dose date, else January will be used for the start month.

If AE end date is earlier than treatment start date (i.e., could be inferred by any combination of year/month/day), then any missing part of AE start date will be imputed based on the AE end date.

13.7.3.2. Incomplete Stop Date

If a stop date is missing the day information and month/year is same as last study date then use last study date, else last day of the given month will be used for the stop day; if a stop date is missing the month and year is the same as last study date then use last study date, else December will be used for the stop month.

13.7.4. Missing Severity Assessment for Adverse Events

If severity is missing for an AE starting prior to the date of the first dose of investigational product, then a severity of "Mild" will be assigned. If the severity is missing for an AE starting on or after the date of the first dose of investigational product, then a severity of "Severe" will be assigned. The imputed values for severity assessment will be used for incidence summaries, while the actual values will be used in data listings.

13.7.5. Missing Relationship to Investigational Product for Adverse Events

If the relationship to investigational product is missing for an AE starting on or after the date of the first dose of investigational product, a causality of "Related" to the investigational product will be assigned. The imputed values for relationship to double-blind investigational product will be used for incidence summaries, while both the actual and the imputed values will be presented in data listings.

13.7.6. Character Values of Clinical Laboratory Variables

If the reported value of a clinical laboratory variable cannot be used in a statistical analysis (e.g., a character string is reported for a numerical variable), the appropriately determined coded value will be used in the statistical analysis, several examples are shown in Table 8 for an illustration purpose. The actual values as reported in the database will be presented in data listings.

Table 8: Examples for Coding of Special Character Values for Clinical Laboratory Variables

Clinical Laboratory Test	Possible Results (in SI units)	Coded Value for Analysis
Chemistry: Bilirubin, Total/Direct	<1.7	0.85
	<2	1
Urinalysis: Specific Gravity	≤1.005	0.5025
	>1.030	1.030
Urinalysis: Glucose	≥55	Positive
	≤0	Negative
Urinalysis: pH	≥8.0	8.0
Urinalysis: Protein	≥500	500
Other: FSH	<0.2	0.1

14. ANALYSIS SOFTWARE

Statistical analyses will be performed using Version 9.4 (or newer) of SAS® on a suitably qualified environment.

15. CHANGES TO ANALYSIS SPECIFIED IN PROTOCOL

The following changes were made to the analyses specified in the protocol

- The mITT set, which includes subjects with a baseline and at least one post-baseline GA lesion assessment, replaced the ITT set as the main efficacy population to be aligned with the primary analysis methodology.
- The change in GA lesion(s) size at Month 24 between PM and Sham was to be the third hypothesis tested in the hypotheses testing strategy as specified in the protocol. This was removed from the hypotheses testing strategy in the SAP.
- The change from baseline in mean threshold sensitivity in all points evaluating the macular functional response at Month 24 was promoted from secondary in the protocol to a key secondary efficacy endpoint here and included in the hypotheses testing algorithm.
- The hypotheses testing strategy and α allocation for the key secondary endpoints was not precisely specified within the protocol and has been clarified to follow the hypotheses testing procedure and α allocation as described in Section 6.3.1.
- Several new microperimetry endpoints were specified in the SAP that were not noted in the protocol.
    - Change from baseline in the mean threshold sensitivity within 500 microns outside the GA lesion, (perilesional points) assessed by mesopic microperimetry over time.
    - Change from baseline in 95% bivariate contour ellipse area (BCEA) over time.
    - Change from baseline in the mean threshold sensitivity of points beyond 500 microns outside the atrophy border (paralesional points) over time.
    - Change from baseline in the mean threshold sensitivity of points beyond the GA lesion (extralesional points) over time.
    - Number of points with clinically significant progression over time.
- Several new exploratory endpoints not specified in the protocol were added.
    - NEI-VFQ-39 distance activity, composite, near activity subscale score
    - NEI-VFQ-25 driving subscale score
    - Progression from iRORA to cRORA
    - Progression from large drusen to iRORA or cRORA
    - Change in the distance of the atrophy junction to the fovea and without subfoveal atrophy to subfoveal atrophy conversion
- No pre-specified analysis will be performed on the secondary endpoint of change from baseline in LLD over time. This data will only be listed.
- No formal interim analyses are planned for this study however, analyses of interim data from Months 12 to 24 that may support regulatory submissions may be conducted.
- Other minor editorial changes were made to provide clarity.

16. REFERENCES

Baskaran K, Macedo AF, and He Y, et al. Scoring reading parameters: An inter-rater reliability study using the MNREAD, *PloSONE* (2019) 14(6): e0216775

Beck RW, Moke PS, and Turpin AH et al. A Computerized Method of Visual Acuity Testing: Adaptation of the Early Treatment of Diabetic Retinopathy Study Testing Protocol, *American Journal of Ophthalmology* (2003) 135 (2):194-205

Bretz, F, Maurer W, Brannath W, Posch M. A Graphical Approach to Sequentially Rejective Multiple Test Procedures, *Statistics in Medicine* (2009) 28.4:586-604.

Calabrese A, Cheong AMY, Cheung S-H, et al. Baseline MNREAD measures for normally sighted subjects from childhood to old age. *Invest Ophthalmol Vis Sci* (2016) 57(8):3836-3843.

Diggle, P.J., Liang, K.-Y., and Zeger, S. L. (1994). Analysis of Longitudinal Data. Oxford: Clarendon Press.

Holekamp N, Wykoff CC, and Schmitz-Valckenberg S et al., Natural History of Geographic Atrophy Secondary to Age-Related Macular Degeneration: Results from the Prospective Proxima A and B Clinical Trials, *Ophthalmology* (2020), doi: https://doi.org/10.1016/j.ophtha.2019.12.009.

Holladay J. Visual Acuity Measurements. *Journal of Cataract & Refractive Surgery* (2004) 30(2):287-290.

Li, K. H. Imputation Using Markov Chains. *Journal of Statistical Computation and Simulation* (1988) 30:57-79.

Liu, C. Bartlett's Decomposition of the Posterior Distribution of the Covariance for Normal Monotone Ignorable Missing Data. *Journal of Multivariate Analysis* (1993) 46:198-206

Patel P, Chen F, Da Cruz L, et al. Test-retest variability of reading performance metrics using MNREAD in patients with age-related macular degeneration. *Invest Opthalmol Vis Sci* (2011). 52(6): 3854-9. doi: 10.1167/iovs. 10-6601.

Radner W and Diendorfer G. English sentence optotypes for measuring reading acuity and speed - the English version of the Radner Reading Charts. *Graefes Arch Clin Exp Ophthalmol* (2014) 252:1297-1303.

Radner W, Obermayer W, Richter-Meuksch S, et al. The validity and reliability of short German sentences for measuring reading speed. *Graefes Arch Clin Exp Ophthalmol* (2002) 240:461-367.

17. APPENDIX

APPENDIX 1. SCHEDULE OF ACTIVITIES

Table 9: Visit Schedule - Monthly Group- Screening, Day 1 through Month 12

	Screening	Treatment													Early Term[A]
Visit #	1	2	3	4	5	6	7	8	9	10	11	12	13	14	
Day	–28 to –1	1	30	60	90	120	150	180	210	240	270	300	330	360	
Week	0	0	4	8	12	16	20	24	28	32	36	40	44	48	
Month	0	0	1	2	3	4	5	6	7	8	9	10	11	12	
Window (+ or – days)	2	0	8	8	8	8	8	8	8	8	8	8	8	8	
**Informed consent / assign screening number**	x														
**Demographic data**	x														
**Inclusion/exclusion criteria** [B]	x	x													
**Medical/surgical/ocular history** [C]	x														
**Blood draw—safety labs** [D,E,F]	x	x		x				x						x	x
**Urine sample collection** [D, E,F]	x	x		x				x						x	x
**Urine pregnancy test** [D,E,F]		x	x	x	x	x	x	x	x	x	x	x	x	x	
**Blood draw—anti-pegcetacoplan Ab** [D]		x	x	x				x						x	x
**Blood draw—genotyping (if applicable)** [D]				x											
**Blood draw for clinical repository (if applicable)**[D,G]				x				x						x	x
**Vital signs** [H]	x	x	x	x	x	x	x	x	x	x	x	x	x	x	x
**Physical examination** [I]	x													x	x
**BCVA** [J]	x	x	x	x	x	x	x	x	x	x	x	x	x	x	x
**LL-BCVA** [J]		x	x	x	x	x	x	x	x	x	x	x	x	x	x
**MNREAD or Radner Reading Charts (select countries)** [J,K]		x						x						x	x
**Mesopic microperimetry**[L]	x							SE						x	x
**Slitlamp examination**	x	x	x	x	x	x	x	x	x	x	x	x	x	x	x
**Endothelial cell count**[S]		x						x						x	x
**NEI VFQ-25** [M]		x						x						x	x
**FRI** [M]		x						x						x	x
**Home-based digital applications** [M,N,S]		x	x	x	x			x						x	
**Dilated indirect ophthalmoscopy**	x	x	x	x	x	x	x	x	x	x	x	x	x	x	x
**IOP measurement**	x	x	x	x	x	x	x	x	x	x	x	x	x	x	x
**SD-OCT** [O]	x	x	x	x	x	x	x	x	x	x	x	x	x	x	x
**FAF** [O]	x	x		SE		SE		x		SE		SE		x	x
**NIR** [O]	x	x		SE		SE		x		SE		SE		x	x
**DCFP** [O]	x													x	x
**FFA** [O]	x													x	x

Table 9: Visit Schedule - Monthly Group- Screening, Day 1 through Month 12

	Screening	Treatment													Early Term[A]
**Visit #**	1	2	3	4	5	6	7	8	9	10	11	12	13	14	
**Day**	–28 to –1	1	30	60	90	120	150	180	210	240	270	300	330	360	
**Week**	0	0	4	8	12	16	20	24	28	32	36	40	44	48	
**Month**	0	0	1	2	3	4	5	6	7	8	9	10	11	12	
**Window (+ or – days)**	2	0	8	8	8	8	8	8	8	8	8	8	8	8	
**OCT-A[S]**		x[S]						x[S]						x[S]	x
**Study eye determination**	x														
**Randomization**		x													
**Pegcetacoplan administration or Sham Injection[T]**		x	x	x	x	x	x	x	x	x	x	x	x	x	
**Postinjection assessment [P]**		x	x	x	x	x	x	x	x	x	x	x	x	x	
**Follow-up call [Q]**		x	x	x	x										
**Concomitant medication/ concomitant ocular procedures [R]**	x	x	x	x	x	x	x	x	x	x	x	x	x	x	x
**Adverse events**	x	x	x	x	x	x	x	x	x	x	x	x	x	x	x

Abbreviations: BCVA=best corrected visual acuity; CNV=choroidal neovascularization; DCFP=digital color fundus photography; eCRF=electronic case report form; FAF=fundus autofluorescence; FFA=fundus fluorescein angiography; FRI=Functional Reading Independence index; IOP=intraocular pressure; LL BCVA= low luminance best corrected visual acuity; MNREAD=Minnesota Low-Vision Reading Test; NEI VFG-25=National Eye Institute Visual Functioning Questionnaire 25-item Version; NIR=near infrared reflectance; OCT-A=optical coherence tomography angiography; SD-OCT=spectral domain optical coherence tomography; SE=study eye; Term=termination; VEGF=vascular endothelial growth factor.

Note: All ocular assessments are to be performed for both eyes unless annotated with 'SE' (study eye) in the above schedule. All assessments should be performed on the same day. All study visits should be scheduled and projected based on the Day 1 visit date.

A. For subjects that discontinue the study early, the early termination assessments should be performed after a minimum of 30 days have passed from the last dosing visit. If a subject reports for a scheduled visit and decides to terminate early prior to dosing, then the visit should be considered the early termination visit and all early termination procedures should be performed. At Month 24, all subjects should be offered entry into an open-label study.

B. At Day 1 (Visit 2), confirm subject eligibility through reviewing the inclusion/ exculsion criteria and receive confirmation of eligibility from the reading center.

C. Significant medical/ surgical history from the previous 5 years. Anti-VEGF treatments (fellow eye) and invasive ocular procedures performed within the past 5 years and while on study should also be recorded. Any history of tobacco use should be recorded.

D. Obtain prior to fluorescein angiography and before study drug administration.

E. At screening, serum pregnancy should be performed for women of childbearing potential. If positive, subject is not eligible to continue in the study.

F. Beginning at Day 1, perform the urine pregnancy test for women of childbearing potential at each treatment visit. If positive, perform a serum pregnancy test. If serum test is positive, study drug should not be administered and an early term visit should be completed.

G. Only subjects that sign the separate consent for the clinical repository and genotyping will have these samples collected. A 14-mL whole-blood sample will be collected at each of the specified visits.

H. Blood pressure, respiratory rate, heart rate, and temperature. On dosing days, vital signs should be taken predose.

I. Height and weight should be collected at screening.

J. Perform assessments prior to dilating the eyes.

K. In select countries, the MNREAD or Radner Reading Charts should be done during the study visit, prior to dilating the eyes. It should be performed monocularly first, then binocularly.

L. Microperimetry assessments will be performed post dilation. Data will be forwarded to the reading center.
M. To be administered by the masked site staff prior to any other assessments performed on that day. In-clinic assessments on the digital application should be completed after completion of all functional tests and quality of life measures prior to dilating the eyes.
N. At select sites, and for those subjects who decide to participate, the digital applications will be completed on an electronic device and will consist of assessments for visual function and reading speed. Subjects will receive training at Day 1 on the use of the digital applications and the electronic device and will perform the tests using the application in the clinic at Month 1, Month 2, Month 3, Month 6, Month 12, Month 18, and Month 24. Beginning at Day 1, subjects will complete the functional assessments weekly at home. Subjects should be instructed to complete the assessments on the same day each week and at approximately the same time of day each week, if possible.
O. FAF and SD-OCT images, near infrared reflectance, fluorescein angiograms, and fundus photographs will be performed for the study eye only on days where 'SE' is specified and for both eyes at all other visits as specified in the above schedule and will be sent to the reading center for evaluation (consult the reading center manual for specifics on image capture, processing, and transmission). Images should be captured prior to dosing on dosing days. If a subject misses a study visit or images cannot be obtained at a specific visit, study staff should make every effort to obtain images at the next scheduled visit. If new active CNV is suspected, SD-OCT, FFA and OCT-A (select sites) images must be collected and sent to the reading center for analysis.
P. Postinjection assessments should be performed within 5 minutes after dosing by the unmasked physician or study staff and should include a gross assessment of vision (finger-counting, hand-motion, then light perception when applicable). If subject passes gross vision test, the subject may leave the site. If subject fails gross vision test, the tonometry should be performed. IOP should be ≤30 mm Hg in order for the subject to leave the site. If necessary, antiglaucomatous medication can be given to lower the IOP. If IOP is >30 mm Hg, assessments will continue every approximately 30 minutes from the previous measurement until the subject passes gross vision test and IOP is ≤30 mm Hg. Note: if the study eye is treated with a ranibizumab or aflibercept injection during the same visit as the study treatment (pegcetacoplan or sham), the treatment with ranibizumab or aflibercept must be performed first. The pre- and post- anti-VEGF IOP values must be measured and recorded on the eCRF.
Q. Starting at Day 1, study subjects will be contacted by study site staff within 4 ± 2 days after each study treatment visit (through Month 3) to collect any information on any safety concerns, decrease in vision, eye pain, unusual ocular events, or any new ocular symptoms in the study eye. If the physician determines that there are any safety concerns, a follow-up visit should be scheduled as soon as possible.
R. Record concomitant medications (ie, prescription and over-the-counter medications) used by the patient within 30 days of screening and throughout the subject's participation in the study.
S. In addition to the time points indicated on the study schedule, OCT-A should be performed at the time of any suspected new active CNV. If new active CNV is confirmed in the study eye, OCT-A should be repeated every 2 months for the study eye.
T. Administration of pegcetacoplan or sham can be done on separate days from the assessment visit if both days fall within the visit window. Administration of study treatment (pegcetacoplan or sham) can be done on a separate day from the assessment visit if both days fall within the visit window. If this occurs on the randomization visit, then the administration of pegcetacoplan or sham should be done within 3 days of randomization and after approval from the medical monitor. When study treatment administration is on a day other than a study visit, then the only assessment that must be done on the day of study treatment administration is preinjection IOP.

Table 10: Visit Schedule - Monthly Group- Month 13 to Month 24

	Treatment												Early Term[A]
**Visit #**	**15**	**16**	**17**	**18**	**19**	**20**	**21**	**22**	**23**	**24**	**25**	**26**	
**Day**	**390**	**420**	**450**	**480**	**510**	**540**	**570**	**600**	**630**	**660**	**690**	**720**	
**Week**	**52**	**56**	**60**	**64**	**68**	**72**	**76**	**80**	**84**	**88**	**92**	**96**	
**Month**	**13**	**14**	**15**	**16**	**17**	**18**	**19**	**20**	**21**	**22**	**23**	**24**	
**Window (+ or – days)**	**8**	**8**	**8**	**8**	**8**	**8**	**8**	**8**	**8**	**8**	**8**	**8**	
**Informed consent / assign screening number**													
**Demographic data**													
**Inclusion/exclusion criteria [B]**													
**Medical/surgical/ocular history [C]**													
**Blood draw—safety labs [D,E,F]**						x						x	x
**Urine sample collection [D,E,F]**						x						x	x
**Urine pregnancy test [D,E,F]**	x	x	x	x	x	x	x	x	x	x	x	x	
**Blood draw—anti-pegcetacoplan Ab [D]**		x				x						x	x
**Blood draw—genotyping (if applicable) [D]**													
**Blood draw for clinical repository (if applicable)[D,G]**												x	x
**Vital signs [H]**	x	x	x	x	x	x	x	x	x	x	x	x	x
**Physical examination [I]**												x	x
**BCVA [J]**	x	x	x	x	x	x	x	x	x	x	x	x	x
**LL-BCVA [I]**	x	x	x	x	x	x	x	x	x	x	x	x	x
**MNREAD or Radner Reading Charts (select countries) [J,K]**						x						x	x
**Mesopic microperimetry[L]**						SE						x	x
**Slitlamp examination**	x	x	x	x	x	x	x	x	x	x	x	x	x
**Endothelial cell count[S]**												x	x
**NEI VFQ-25 [M]**						x						x	x
**FRI [M]**						x						x	x
**Dilated Indirect Ophthalmoscopy**	x	x	x	x	x	x	x	x	x	x	x	x	x
**Home-based digital applications [M,N,S]**						x						x	
**IOP measurement**	x	x	x	x	x	x	x	x	x	x	x	x	x
**SD-OCT [O]**	x	x	x	x	x	x	x	x	x	x	x	x	x
**FAF [O]**		SE		SE		x		SE		SE		x	x
**NIR [O]**		SE		SE		x		SE		SE		x	x
**DCFP [O]**												x	x
**FFA [O]**												x	x

Table 10: Visit Schedule - Monthly Group- Month 13 to Month 24

	Treatment												Early Term[A]
**Visit #**	**15**	**16**	**17**	**18**	**19**	**20**	**21**	**22**	**23**	**24**	**25**	**26**	
**Day**	**390**	**420**	**450**	**480**	**510**	**540**	**570**	**600**	**630**	**660**	**690**	**720**	
**Week**	**52**	**56**	**60**	**64**	**68**	**72**	**76**	**80**	**84**	**88**	**92**	**96**	
**Month**	**13**	**14**	**15**	**16**	**17**	**18**	**19**	**20**	**21**	**22**	**23**	**24**	
**Window (+ or – days)**	**8**	**8**	**8**	**8**	**8**	**8**	**8**	**8**	**8**	**8**	**8**	**8**	
**OCT-A[S]**						x[S]						x[S]	x
**Study eye determination**													
**Randomization**													
**Pegcetacoplan administration or Sham Injection[T]**	x	x	x	x	x	x	x	x	x	x	x		
**Postinjection assessment [P]**	x	x	x	x	x	x	x	x	x	x	x		
**Follow-up call [Q]**													
**Concomitant medication/ concomitant ocular procedures [R]**	x	x	x	x	x	x	x	x	x	x	x	x	x
**Adverse events**	x	x	x	x	x	x	x	x	x	x	x	x	x

Abbreviations: BCVA=best corrected visual acuity; CNV=choroidal neovascularization; DCFP=digital color fundus photography; eCRF=electronic case report form; FAF=fundus autofluorescence; FFA=fundus fluorescein angiography; FRI=Functional Reading Independence index; IOP=intraocular pressure; LL BCVA= low luminance best corrected visual acuity; MNREAD=Minnesota Low-Vision Reading Test; NEI VFG-25=National Eye Institute Visual Functioning Questionnaire 25-item Version; NIR=near infrared reflectance; OCT-A=optical coherence tomography angiography; SD-OCT=spectral domain optical coherence tomography; SE=study eye; Term=termination; VEGF=vascular endothelial growth factor.

Note: All ocular assessments are to be performed for both eyes unless annotated with 'SE' (study eye) in the above schedule. All assessments should be performed on the same day. All study visits should be scheduled and projected based on the Day 1 visit date.

A. For subjects that discontinue the study early, the early termination assessments should be performed after a minimum of 30 days have passed from the last dosing visit. If a subject reports for a scheduled visit and decides to terminate early prior to dosing, then the visit should be considered the early termination visit and all early termination procedures should be performed. At Month 24, all subjects should be offered entry into an open-label study.

B. At Day 1 (Visit 2), confirm subject eligibility through reviewing the inclusion/ exculsion criteria and receive confirmation of eligibility from the reading center.

C. Significant medical/ surgical history from the previous 5 years. Anti-VEGF treatments (fellow eye) and invasive ocular procedures performed within the past 5 years and while on study should also be recorded. Any history of tobacco use should be recorded.

D. Obtain prior to fluorescein angiography and before study drug administration.

E. At screening, serum pregnancy should be performed for women of childbearing potential. If positive, subject is not eligible to continue in the study.

F. Beginning at Day 1, perform the urine pregnancy test for women of childbearing potential at each treatment visit. If positive, perform a serum pregnancy test. If serum test is positive, study drug should not be administered and an early term visit should be completed.

G. Only subjects that sign the separate consent for the clinical repository and genotyping will have these samples collected. A 14-mL whole-blood sample will be collected at each of the specified visits.

H. Blood pressure, respiratory rate, heart rate, and temperature. On dosing days, vital signs should be taken predose.

I. Height and weight should be measured at screening.

J. Perform assessments prior to dilating the eyes.

K. In select countries, the MNREAD or Radner Reading Charts should be done during the study visit, prior to dilating the eyes. It should be performed monocularly first, then binocularly.

L. Microperimetry assessments will be performed post dilation. Data will be forwarded to the reading center.
M. To be administered by the masked site staff prior to any other assessments performed on that day. In-clinic assessments on the digital application should be completed after completion of all functional tests and quality of life measures prior to dilating the eyes.
N. At select sites, and for those subjects who decide to participate, the digital applications will be completed on an electronic device and will consist of assessments for visual function and reading speed. Subjects will receive training at Day1 on the use of the digital applications and the electronic device and will perform the tests using the application in the clinic at Month 1, Month 2, Month 3, Month 6, Month 12, Month 18, and Month 24. Beginning at Day 1, subjects will complete the functional assessments weekly at home. Subjects should be instructed to complete the assessments on the same day each week and at approximately the same time of day each week, if possible.
O. FAF and SD-OCT images, near infrared reflectance, fluorescein angiograms, and fundus photographs will be performed for the study eye only on days where 'SE' is specified and for both eyes at all other visits as specified in the above schedule and will be sent to the reading center for evaluation (consult the reading center manual for specifics on image capture, processing, and transmission). Images should be captured prior to dosing on dosing days. If a subject misses a study visit or images cannot be obtained at a specific visit, study staff should make every effort to obtain images at the next scheduled visit. If new active CNV is suspected, SD-OCT, FFA, and OCT-A (selected sites) images should be collected and sent to the reading center for analysis.
P. Postinjection assessments should be performed within 5 minutes after dosing by the unmasked physician or study staff and should include a gross assessment of vision (finger-counting, hand motion, then light perception when applicable). If subject passes gross vision test, the subject may leave the site. If subject fails gross vision test, the tonometry should be performed. IOP should be ≤30 mm Hg in order for the subject to leave the site. If necessary, antiglaucomatous medication can be given to lower the IOP. If IOP is >30 mm Hg, assessments will continue every approximately 30 minutes from the previous measurement until the subject passes gross vision test and IOP is ≤30 mm Hg. Note: if the study eye is treated with a ranibizumab or aflibercept injection during the same visit as the study treatment (pegcetacoplan or sham), the treatment with ranibizumab or aflibercept must be performed first. The pre- and post- anti-VEGF IOP values must be measured and recorded on the eCRF.
Q. Starting at Day 1, study subjects will be contacted by study site staff within 4 ± 2 days after each study treatment visit (through Month 3) to collect any information on any safety concerns, decrease in vision, eye pain, unusual ocular events, or any new ocular symptoms in the study eye. If the physician determines that there are any safety concerns, a follow-up visit should be scheduled as soon as possible.
R. Record concomitant medications (ie, prescription and over-the-counter medications) used by the patient within 30 days of screening and throughout the subject's participation in the study.
S. In addition to the time points indicated on the study schedule, OCT-A should be performed at the time of any suspected new active CNV. If new active CNV is confirmed in the study eye, OCT-A should be repeated every 2 months for the study eye.
T. Administration of pegcetacoplan or sham can be done on separate days from the assessment visit if both days fall within the visit window. Administration of study treatment (pegcetacoplan or sham) can be done on a separate day from the assessment visit if both days fall within the visit window. If this occurs on the randomization visit, then the administration of pegcetacoplan or sham should be done within 3 days of randomization and after approval from the medical monitor. When study treatment administration is on a day other than a study visit, then the only assessment that must be done on the day of study treatment administration is preinjection IOP.

Table 11: Visit Schedule - Every-Other-Month Group Screening, Day 1 Through Month 12

	Screening	Treatment													Early Term[A]
**Visit #**	1	2	3	4	5	6	7	8	9	10	11	12	13	14	
**Day**	–28 to –1	1	30	60	90	120	150	180	210	240	270	300	330	360	
**Week**	0	0	4	8	12	16	20	24	28	32	36	40	44	48	
**Month**	0	0	1	2	3	4	5	6	7	8	9	10	11	12	
**Window (+ or – days)**	2	0	8	8	8	8	8	8	8	8	8	8	8	8	
**Informed consent / assign screening number**	x														
**Demographic data**	x														
**Inclusion/exclusion criteria** [B]	x	x													
**Medical/surgical/ocular history** [C]	x														
**Blood draw—safety labs** [D,E,F]	x	x		x				x						x	x
**Urine sample collection** [D,E,F]	x	x		x				x						x	x
**Urine pregnancy test** [D,E,F]		x		x		x		x		x		x		x	
**Blood draw—anti–pegcetacoplan Ab** [D]		x	x	x				x						x	x
**Blood draw—genotyping (if applicable)** [D]				x											
**Blood draw for clinical repository (if applicable)** [D,G]				x				x						x	x
**Vital signs** [H]	x	x	x	x	x	x	x	x	x	x	x	x	x	x	x
**Physical examination** [I]	x													x	x
**BCVA** [J]	x	x	x	x	x	x	x	x	x	x	x	x	x	x	x
**LL-BCVA** [J]		x	x	x	x	x	x	x	x	x	x	x	x	x	x
**MNREAD or Radner Reading Charts (select countries)** [J,K]		x						x						x	x
**Mesopic microperimetry**[L]	x							SE						x	x
**Slitlamp examination**	x	x	x	x	x	x	x	x	x	x	x	x	x	x	x
**Endothelial cell count**[S]		x						x						x	x
**NEI VFQ-25** [M]		x						x						x	x
**FRI** [M]		x						x						x	x
**Home-based digital applications** [M,N,S]		x	x	x	x			x						x	
**Dilated indirect ophthalmoscopy**	x	x	x	x	x	x	x	x	x	x	x	x	x	x	x
**IOP measurement**	x	x	x	x	x	x	x	x	x	x	x	x	x	x	x
**SD-OCT** [O]	x	x	x	x	x	x	x	x	x	x	x	x	x	x	x
**FAF** [O]	x	x		SE		SE		x		SE		SE		x	x
**NIR** [O]	x	x		SE		SE		x		SE		SE		x	x
**DCFP** [O]	x													x	x
**FFA** [O]	x													x	x

Table 11: Visit Schedule - Every-Other-Month Group Screening, Day 1 Through Month 12

	Screening	Treatment													Early Term[A]
**Visit #**	**1**	**2**	**3**	**4**	**5**	**6**	**7**	**8**	**9**	**10**	**11**	**12**	**13**	**14**	
**Day**	**–28 to –1**	**1**	**30**	**60**	**90**	**120**	**150**	**180**	**210**	**240**	**270**	**300**	**330**	**360**	
**Week**	**0**	**0**	**4**	**8**	**12**	**16**	**20**	**24**	**28**	**32**	**36**	**40**	**44**	**48**	
**Month**	**0**	**0**	**1**	**2**	**3**	**4**	**5**	**6**	**7**	**8**	**9**	**10**	**11**	**12**	
**Window (+ or – days)**	**2**	**0**	**8**	**8**	**8**	**8**	**8**	**8**	**8**	**8**	**8**	**8**	**8**	**8**	
**OCT-A[S]**		x[S]						x[S]						x[S]	x
**Study eye determination**	x														
**Randomization**		x													
**Pegcetacoplan administration or Sham Injection[T]**		x		x		x		x		x		x		x	
**Postinjection assessment [P]**		x		x		x		x		x		x		x	
**Follow-up call [Q]**		x		x		x									
**Concomitant medication/ concomitant ocular procedures [R]**	x	x	x	x	x	x	x	x	x	x	x	x	x	x	x
**Adverse events**	x	x	x	x	x	x	x	x	x	x	x	x	x	x	x

Abbreviations: BCVA=best corrected visual acuity; CNV=choroidal neovascularization; DCFP=digital color fundus photography; eCRF=electronic case report form; FAF=fundus autofluorescence; FFA=fundus fluorescein angiography; FRI=Functional Reading Independence index; IOP=intraocular pressure; LL BCVA= low luminance best corrected visual acuity; MNREAD=Minnesota Low-Vision Reading Test; NEI VFG-25=National Eye Institute Visual Functioning Questionnaire 25-item Version; NIR=near infrared reflectance; OCT-A=optical coherence tomography angiography; SD-OCT=spectral domain optical coherence tomography; SE=study eye; Term=termination; VEGF=vascular endothelial growth factor.

Note: All ocular assessments are to be performed for both eyes unless annotated with 'SE' (study eye) in the above schedule. All assessments should be performed on the same day. All study visits should be scheduled and projected based on the Day 1 visit date.

A. For subjects that discontinue the study early, the early termination assessments should be performed after a minimum of 30 days have passed from the last dosing visit. If a subject reports for a scheduled visit and decides to terminate early prior to dosing, then the visit should be considered the early termination visit and all early termination procedures should be performed. At Month 24, all subjects should be offered entry into an open-label study.

B. At Day 1 (Visit 2), confirm subject eligibility through reviewing the inclusion/exclusion criteria and receive confirmation of eligibility from the reading center.

C. Significant medical/ surgical history from the previous 5 years. Anti-VEGF treatments (fellow eye) and invasive ocular procedures performed within the past 5 years and while on study should also be recorded. Any history of tobacco use should be recorded.

D. Obtain prior to fluorescein angiography and before study drug administration.

E. At screening, serum pregnancy should be performed for women of childbearing potential. If positive, subject is not eligible to continue in the study.

F. Beginning at Day 1, perform the urine pregnancy test for women of childbearing potential at each treatment visit. If positive, perform a serum pregnancy test. If serum test is positive, study drug should not be administered and an early term visit should be completed.

G. Only subjects that sign the separate consent for the clinical repository and genotyping will have these samples collected. A 14-mL whole-blood sample will be collected at each of the specified visits.

H. Blood pressure, respiratory rate, heart rate, and temperature. On dosing days, vital signs should be taken pre- dose.

I. Height and Weight should be measured at screening.

J. Perform assessments prior to dilating the eyes.

K. In select countries, the MNREAD or Radner Reading Charts should be done during the study visit, prior to dilating the eyes. It should be performed monocularly first, then binocularly.

L. Microperimetry assessments will be performed post dilation. Data will be forwarded to the reading center.
M. To be administered by the masked site staff prior to any other assessments performed on that day. In-clinic assessments on the digital application should be completed after completion of all functional tests and quality of life measures prior to dilating the eyes.
N. At select sites, and for those subjects who decide to participate, the digital application will be completed on an electronic device and will consist of assessments for visual function and reading speed. Subjects will receive training at Day 1 on the use of the digital applications and the electronic device and will perform the tests using the application in the clinic at Month 1, Month 2, Month 3, Month 6, Month 12, Month 18, and Month 24. Beginning at Day 1, subjects will complete the functional assessments weekly at home. Subjects should be instructed to complete the assessments on the same day each week and at approximately the same time of day each week, if possible.
O. FAF and SD-OCT images, near infrared reflectance, fluorescein angiograms, and fundus photographs will be performed for the study eye only on days where 'SE' is specified and for both eyes at all other visits as specified in the above schedule and will be sent to the reading center for evaluation (consult the reading center manual for specifics on image capture, processing, and transmission). Images should be captured prior to dosing on dosing days. If a subject misses a study visit or images cannot be obtained at a specific visit, study staff should make every effort to obtain images at the next scheduled visit. If new active CNV is suspected, SD-OCT, FFA, and OCT-A (selected sites) images should be collected and sent to the reading center for analysis.
P. Postinjection assessments should be performed within 5 minutes after dosing by the unmasked physician or study staff and should include a gross assessment of vision (finger-counting, hand motion, then light perception when applicable). If subject passes gross vision test, the subject may leave the site. If subject fails gross vision test, the tonometry should be performed. IOP should be ≤30 mm Hg in order for the subject to leave the site. If necessary, antiglaucomatous medication can be given to lower the IOP. If IOP is >30 mm Hg, assessments will continue every approximately 30 minutes from the previous measurement until the subject passes gross vision test and IOP is ≤30 mm Hg. Note: if the study eye is treated with a ranibizumab or aflibercept injection during the same visit as the study treatment (pegcetacoplan or sham), the treatment with ranibizumab or aflibercept must be performed first. The pre- and post- anti-VEGF IOP values must be measured and recorded on the eCRF.
Q. Starting at Day 1, study subjects will be contacted by study site staff within 4 ± 2 days after each study treatment visit (through Month 4) to collect any information on any safety concerns, decrease in vision, eye pain, unusual ocular events, or any new ocular symptoms in the study eye. If the physician determines that there are any safety concerns, a follow-up visit should be scheduled as soon as possible.
R. Record concomitant medications (ie, prescription and over-the-counter medications) used by the patient within 30 days of screening and throughout the subject's participation in the study.
S. In addition to the time points indicated on the study schedule, OCT-A should be performed at the time of any suspected new active CNV. If new active CNV is confirmed in the study eye, OCT-A should be repeated every 2 months for the study eye.
T. Administration of pegcetacoplan or sham can be done on separate days from the assessment visit if both days fall within the visit window. Administration of study treatment (pegcetacoplan or sham) can be done on a separate day from the assessment visit if both days fall within the visit window. If this occurs on the randomization visit, then the administration of pegcetacoplan or sham should be done within 3 days of randomization and after approval from the medical monitor. When study treatment administration is on a day other than a study visit, then the only assessment that must be done on the day of study treatment administration is preinjection IOP

Table 12: Visit Schedule - Every-Other-Month Group Month 13 to Month 24

	Treatment												Early Term [A]
**Visit #**		15		16		17		18		19		20	
**Day**	390	420	450	480	510	540	570	600	630	660	690	720	
**Week**	52	56	60	64	68	72	76	80	84	88	92	96	
**Month**	13	14	15	16	17	18	19	20	21	22	23	24	
**Window (+ or – days)**		16		16		16		16		16		16	
**Informed consent / assign screening number**													
**Demographic data**													
**Inclusion/exclusion criteria [B]**													
**Medical/surgical/ocular history [C]**													
**Blood draw—safety labs [D,E,F]**						x						x	x
**Urine sample collection [D, E,F]**						x						x	x
**Urine pregnancy test [D,E,F]**		x		x		x		x		x		x	
**Blood draw—anti-pegcetacoplan Ab [D]**		x				x						x	x
**Blood draw—genotyping (if applicable) [D]**													
**Blood draw for clinical repository (if applicable) [D,G]**												x	x
**Vital signs [H]**		x		x		x		x		x		x	x
**Physical examination [I]**												x	x
**BCVA [J]**		x		x		x		x		x		x	x
**LL-BCVA [J]**		x		x		x		x		x		x	x
**MNREAD or Radner Reading Charts (select countries) [J,K]**						x						x	x
**Mesopic microperimetry[L]**						SE						x	x
**Slitlamp examination**		x		x		x		x		x		x	x
**Endothelial cell count[S]**												x	x
**NEI VFQ-25 [M]**						x						x	x
**FRI [M]**						x						x	x
**Home-based digital applications [M,N,S]**						x						x	
**Dilated indirect ophthalmoscopy**		x		x		x		x		x		x	x
**IOP measurement**		x		x		x		x		x		x	x
**SD-OCT [O]**		x		x		x		x		x		x	x
**FAF [O]**		SE		SE		x		SE		SE		x	x
**NIR[O]**		SE		SE		x		SE		SE		x	x
**DCFP [O]**												x	x
**FFA [O]**												x	x

Table 12: Visit Schedule - Every-Other-Month Group Month 13 to Month 24

	Treatment												Early Term [A]
**Visit #**		**15**		**16**		**17**		**18**		**19**		**20**	
**Day**	**390**	**420**	**450**	**480**	**510**	**540**	**570**	**600**	**630**	**660**	**690**	**720**	
**Week**	**52**	**56**	**60**	**64**	**68**	**72**	**76**	**80**	**84**	**88**	**92**	**96**	
**Month**	**13**	**14**	**15**	**16**	**17**	**18**	**19**	**20**	**21**	**22**	**23**	**24**	
**Window (+ or – days)**		**16**		**16**		**16**		**16**		**16**		**16**	
**OCT-A[S]**						x[S]						x[S]	x
**Study eye determination**													
**Randomization**													
**Pegcetacoplan administration or Sham Injection[T]**		x		x		x		x		x			
**Postinjection assessment [P]**		x		x		x		x		x			
**Follow-up call [Q]**													
**Concomitant medication/ concomitant ocular procedures [R]**		x		x		x		x		x		x	x
**Adverse events**		x		x		x		x		x		x	x

Abbreviations: BCVA=best corrected visual acuity; CNV=choroidal neovascularization; DCFP=digital color fundus photography; eCRF=electronic case report form; FAF=fundus autofluorescence; FFA=fundus fluorescein angiography; FRI=Functional Reading Independence index; IOP=intraocular pressure; LL BCVA= low luminance best corrected visual acuity; MNREAD=Minnesota Low-Vision Reading Test; NEI VFG-25=National Eye Institute Visual Functioning Questionnaire 25-item Version; NIR=near infrared reflectance; OCT-A=optical coherence tomography angiography; SD-OCT=spectral domain optical coherence tomography; SE=study eye; Term=termination; VEGF=vascular endothelial growth factor.

Note: All ocular assessments are to be performed for both eyes unless annotated with 'SE' (study eye) in the above schedule. All assessments should be performed on the same day. All study visits should be scheduled and projected based on the Day 1 visit date.

A. For subjects that discontinue the study early, the early termination assessments should be performed after a minimum of 30 days have passed from the last dosing visit. If a subject reports for a scheduled visit and decides to terminate early prior to dosing, then the visit should be considered the early termination visit and all early termination procedures should be performed. At Month 24, all subjects should be offered entry into an open-label study.

B. At Day 1 (Visit 2), confirm subject eligibility through reviewing the inclusion/ exclusion criteria and receive confirmation of eligibility from the reading center.

C. Significant medical/ surgical history from the previous 5 years. Anti-VEGF treatments (fellow eye) and invasive ocular procedures performed within the past 5 years and while on study should be recorded. Any history of tobacco use should be recorded.

D. Obtain prior to fluorescein angiography and before study drug administration.

E. At screening, serum pregnancy should be performed for women of childbearing potential. If positive, subject is not eligible to continue in the study.

F. Beginning at Day 1, perform the urine pregnancy test for women of childbearing potential at each treatment visit. If positive, perform a serum pregnancy test. If serum test is positive, study drug should not be administered and an early term visit should be completed.

G. Only subjects that sign the separate consent for the clinical repository and genotyping will have these samples collected. A 14-mL whole-blood sample will be collected at the specified time points.

H. Blood pressure, respiratory rate, heart rate, and temperature. On dosing days, vital signs should be taken predose.

I. Height and weight should be measured at screening.

J. Perform assessments prior to dilating the eyes.

K. In select countries, the MNREAD or Radner Reading Charts should be done during the study visit, prior to dilating the eyes. It should be performed monocularly first, then binocularly.

L. Microperimetry assessments will be performed post dilation. Data will be forwarded to the reading center.

M. To be administered by the masked site staff prior to any other assessments performed on that day. In-clinic assessments on the digital application should be completed after completion of all functional tests and quality of life measures prior to dilating the eyes.
N. At select sites, and for those subjects who decide to participate, the digital applications will be completed on an electronic device and will consist of assessments for visual function and reading speed. Subjects will receive training at Day 1 on the use of the digital applications and the electronic device and will perform the tests using the application in the clinic at Month 1, Month 2, Month 3, Month 6, Month 12, Month 18, and Month 24. Beginning at Day 1, subjects will complete the functional assessments weekly at home. Subjects should be instructed to complete the assessments on the same day each week and at approximately the same time of day each week, if possible.
O. FAF and SD-OCT images, near infrared reflectance, fluorescein angiograms, and fundus photographs will be performed for the study eye only on days where 'SE' is specified and for both eyes at all other visits as specified in the above schedule and will be sent to the reading center for evaluation (consult the reading center manual for specifics on image capture, processing, and transmission). Images should be captured prior to dosing on dosing days. If a subject misses a study visit or images cannot be obtained at a specific visit, study staff should make every effort to obtain images at the next scheduled visit. If new active CNV is suspected, SD-OCT, FFA, and OCT-A (selected sites) images should be collected and sent to the reading center for analysis.
P. Postinjection assessments should be performed within 5 minutes after dosing by the unmasked physician or study staff and should include a gross assessment of vision (finger-counting, hand motion, then light perception when applicable). If subject passes gross vision test, the subject may leave the site. If subject fails gross vision test, the tonometry should be performed. IOP should be ≤30 mm Hg in order for the subject to leave the site. If necessary, antiglaucomatous medication can be given to lower the IOP. If IOP is >30 mm Hg, assessments will continue every approximately 30 minutes from the previous measurement until the subject passes the gross vision test and IOP is ≤30 mm Hg. Note: if the study eye is treated with a ranibizumab or aflibercept injection during the same visit as the study treatment (pegcetacoplan or sham), the treatment with ranibizumab or aflibercept must be performed first. The pre- and post- anti-VEGF IOP values must be measured and recorded on the eCRF.
Q. Starting at Day 1, study subjects will be contacted by study site staff within 4 ± 2 days after each study treatment visit (through Month 4) to collect any information on any safety concerns, decrease in vision, eye pain, unusual ocular events, or any new ocular symptoms in the study eye. If the investigator determines that there are any safety concerns, a follow-up visit should be scheduled as soon as possible.
R. Record concomitant medications (ie, prescription and over-the-counter medications) used by the patient within 30 days of screening and throughout the subject's participation in the study.
S. In addition to the time points indicated on the study schedule, OCT-A should be performed at the time of any suspected new active CNV. If new active CNV is confirmed in the study eye, OCT-A should be repeated every 2 months for the study eye.
T. Administration of pegcetacoplan or sham can be done on separate days from the assessment visit if both days fall within the visit window. Administration of study treatment (pegcetacoplan or sham) can be done on a separate day from the assessment visit if both days fall within the visit window. If this occurs on the randomization visit, then the administration of pegcetacoplan or sham should be done within 3 days of randomization and after approval from the medical monitor. When study treatment administration is on a day other than a study visit, then the only assessment that must be done on the day of study treatment administration is preinjection IOP.

1.1. Protocol Changes to be followed during COVID-19 Restrictions

OVERVIEW

In response to the COVID-19 crisis, to ensure the safety of study subjects and Investigative Sites as well as proper conduct of the study, TEMPORARY changes to the protocol have been implemented. These changes should be followed only during COVID-19 restrictions and include extended IP administration windows, changes to masking rules, rescreening instructions, and a revised schedule of assessments.

Where feasible, sites could continue to follow the full schedule of assessments (based on their treatment group assignment).

EXTENDED IP ADMINISTRATION WINDOWS

In order to allow more flexibility to sites and subjects, and to potentially mitigate missed IP administration, an extended IP administration window can be followed. The extended window can ONLY be used in situations related to COVID-19 restrictions and after medical monitor approval. Footnote "T" of each COVID-19 assessment table below reflects these extended IP options.

Per protocol, the IP administration window is as follows:

- Monthly treatment group: +/– 8 days for the entire study duration
- EOM treatment group: +/– 8 days for the first study year and +/- 16 days for the second study year

During COVID-19 restrictions, the IP administration window can be extended to the following:

- Monthly treatment group: –8 days to +15 days. Note, interval for consecutive injections must be at least 14 days.
- EOM treatment group: –8 days to +30 days for the first study year and –16 days to +30 days for the second study year.

MASKING RULES

Due to current COVID-19 restrictions, clinical sites might encounter difficulties maintaining appropriate clinic staffing to satisfy the approved masking rules for the APL2-304 (Oaks) study. Based on this, and in an attempt to minimize the amount of missed data and IP administrations, Apellis is implementing a temporary adjustment to the study masking rules.

This temporary change must be approved by the Apellis Medical Director **prior to** implementation and must be documented via a temporary and modified delegation of authority Log. Each masked assessment performed by an unmasked staff and vice versa (even with Apellis approval and following the below guidelines) should be documented.

The principal investigator (PI) is responsible for the overall oversight of the study site data and s/he will not be allowed to switch into an unmasked role. Every masked individual that performs IP administration and/or postinjection assessment (all unmasked assessment) as a temporary measure, will **permanently** be considered an unmasked individual and will not be able to perform masked assessments once these exemptions are lifted.

RESCREENING PROCEDURE

Prior to the implementation of these temporary changes, sites continuing to screen patients have been encouraged to complete the screening and baseline assessments in their entirety. However, if a subject was deemed a screen failure for not being able to meet the original screening window (Day –28 to Day –1 [+/- 2 days]) due to COVID-19 related restrictions, a rescreening visit is allowed and should be followed according to the 2 scenarios below.

Subjects Who Completed Screening and Were Considered Eligible by Reading Center and Investigator

Subjects who were screened prior to 30 March 2020 and completed all screening assessments (as described in the Schedule of Assessments Table 9 [every month treatment group] and Table 11 [every other month [EOM] treatment group]) and considered eligible by the reading center and investigator and are able to return to the clinic within 90 days of initial screening, will receive a new subject ID number and undergo an ***abbreviated screening,*** prior to randomization, that includes the following assessments:

- Informed consent/assign new screening number
- Normal luminance best corrected visual acuity assessment
- Slitlamp examination
- Dilated indirect ophthalmoscopy
- IOP measurement
- SD-OCT*
- Concomitant medication/concomitant ocular procedures collection
- AE collection

*SD-OCT images collected at this visit will not be used by the reading center to determine eligibility but should be used by the investigator to detect any potential new exclusion criterion.

If the investigator deems it necessary, additional assessments can be performed if there is a concern that the subject might now meet an exclusion criterion that was not the case during the original screening (eg, FFA to exclude the presence of CNV).

Subjects With Incomplete Screening Assessment

Subjects that signed the informed consent but were not able to complete all screening assessments due to COVID-19-related restrictions are not eligible for the abbreviated screening. These subjects can be rescreened but must follow the standard screening schedule of assessment (as described in the Schedule of Assessments Table 9 [every month treatment group] and Table 11 [EOM treatment group]). These subjects will also receive a new screening ID number.

MINIMUM SCHEDULE OF ASSESSMENT

Schedule of Assessments

Where feasible, sites could continue to follow the full schedule of assessments (based on their treatment group assignment). The minimum assessment tables, only to be followed during this COVID-19 effort and if determined necessary to use based on the investigator's clinical judgment, are provided below to reduce the time required for each study visit. Subjects in the EOM treatment group do not need to be seen for the non-IP administration visits. Assessments not performed (even those that have been removed in the minimum assessment table) should be documented.

Subjects that are not able to come into the clinic for a study visit due to COVID-19-related restrictions, including visits for the EOM group that do not include IP administration, should be contacted via the phone for the collection of AEs (including SAEs) and concomitant medications. **All SAEs are still required to be reported to Apellis within 24 hours of site awareness, even if reported via phone call**. All communications via phone call should also be documented in the source documents and in the respective CRF page. In addition, these subjects should be instructed to self-monitor their vision at home and report any changes in vision or their overall health via phone call. The site must inform the Sponsor of any subjects lost to follow-up.

It is critical that local, country, and regional governance regarding COVID-19 is followed along with your best clinical judgment when managing this situation. All visits or assessments missed as a result of COVID-19 will be captured in the case report forms.

Table 13 (TRACKED): COVID-19 VISIT SCHEDULE—Monthly Group—Screening, Day 1 Through Month 12

	Screening	Treatment													Early Term[A]
Visit #	1	2	3	4	5	6	7	8	9	10	11	12	13	14	
Day	–28 to –1	1	30	60	90	120	150	180	210	240	270	300	330	360	
Week	0	0	4	8	12	16	20	24	28	32	36	40	44	48	
Month	0	0	1	2	3	4	5	6	7	8	9	10	11	12	
Window (+ or – days)	2	0	8	8	8	8	8	8	8	8	8	8	8	8	
Informed Consent / Assign Screening Number	x														
Demographic Data	x														
Inclusion/Exclusion Criteria [B]	x	x													
Medical/Surgical/Ocular History [C]	x														
Blood Draw—Safety Labs [D,E,F]	x	x		~~x~~				~~x~~						x	x
Urine Sample Collection [D, E,F]	x	x		~~x~~				~~x~~						x	x
Urine Pregnancy Test [D,E,F]		x	x	x	x	x	x	x	x	x	x	x	x	x	
Blood Draw—Anti-Pegcetacoplan Ab [D]		x	~~x~~	~~x~~				~~x~~						x	x
Blood Draw—Genotyping (if applicable) [D]				~~x~~											
Blood Draw for Clinical Repository (if applicable)[D,G]				~~x~~				~~x~~						~~x~~	~~x~~
Vital Signs [H]	x	x	~~x~~	~~x~~	~~x~~	~~x~~	~~x~~	x	~~x~~	~~x~~	~~x~~	~~x~~	~~x~~	x	x
Physical Examination [I]	x													x	x
BCVA [J]	x	x	x	x	x	x	x	x	x	x	x	x	x	x	x
LL-BCVA [J]		x	~~x~~	~~x~~	~~x~~	~~x~~	~~x~~	x	~~x~~	~~x~~	~~x~~	~~x~~	~~x~~	x	x
MNREAD or Radner Reading Charts (select countries) [J,K]		x						~~x~~						x	x
Mesopic Microperimetry[L]	x							~~SE~~						x	x
Slitlamp Examination	x	x	x	x	x	x	x	x	x	x	x	x	x	x	x
Endothelial Cell Count[S]		x						x						x	x
NEI VFQ-25 [M]		x						~~x~~						x	x
FRI [M]		x						~~x~~						x	x
Home-Based Digital Applications [M,N,S]		x	~~x~~	~~x~~	~~x~~			~~x~~						x	
Dilated Indirect Ophthalmoscopy	x	x	x	x	x	x	x	x	x	x	x	x	x	x	x
IOP Measurement	x	x	x	x	x	x	x	x	x	x	x	x	x	x	x
SD-OCT [O]	x	x	~~x~~	~~x~~	~~x~~	~~x~~	~~x~~	x	~~x~~	~~x~~	~~x~~	~~x~~	~~x~~	x	x
FAF [O]	x	x		~~SE~~		~~SE~~		x		~~SE~~		~~SE~~		x	x
NIR [O]	x	x		~~SE~~		~~SE~~		x		~~SE~~		~~SE~~		x	x
DCFP [O]	x													x	x
FFA [O]	x													x	x
OCT-A[S]		x[S]						~~x~~[S]						x[S]	x

Table 13 (TRACKED): COVID-19 VISIT SCHEDULE—Monthly Group—Screening, Day 1 Through Month 12

	Screening	Treatment													Early Term[A]
**Visit #**	**1**	**2**	**3**	**4**	**5**	**6**	**7**	**8**	**9**	**10**	**11**	**12**	**13**	**14**	
**Day**	**–28 to –1**	**1**	**30**	**60**	**90**	**120**	**150**	**180**	**210**	**240**	**270**	**300**	**330**	**360**	
**Week**	**0**	**0**	**4**	**8**	**12**	**16**	**20**	**24**	**28**	**32**	**36**	**40**	**44**	**48**	
**Month**	**0**	**0**	**1**	**2**	**3**	**4**	**5**	**6**	**7**	**8**	**9**	**10**	**11**	**12**	
**Window (+ or – days)**	**2**	**0**	**8**	**8**	**8**	**8**	**8**	**8**	**8**	**8**	**8**	**8**	**8**	**8**	
**Study Eye Determination**	x														
**Randomization**		x													
**Pegcetacoplan administration or Sham Injection[T]**		x	x	x	x	x	x	x	x	x	x	x	x	x	
**Postinjection Assessment [P]**		x	x	x	x	x	x	x	x	x	x	x	x	x	
**Follow-Up Call [Q]**		x	x	x	x										
**Concomitant Medication/ Concomitant Ocular Procedures [R]**	x	x	x	x	x	x	x	x	x	x	x	x	x	x	x
**Adverse Events**	x	x	x	x	x	x	x	x	x	x	x	x	x	x	x

Abbreviations: BCVA=best corrected visual acuity; CNV=choroidal neovascularization; DCFP=digital color fundus photography; eCRF=electronic case report form; FAF=fundus autofluorescence; FFA=fundus fluorescein angiography; FRI=Functional Reading Independence index; IOP=intraocular pressure; LL BCVA= low luminance best corrected visual acuity; MNREAD=Minnesota Low-Vision Reading Test; NEI VFG-25=National Eye Institute Visual Functioning Questionnaire 25-item Version; NIR=near infrared reflectance; OCT-A=optical coherence tomography angiography; SD-OCT=spectral domain optical coherence tomography; SE=study eye; Term=termination; VEGF=vascular endothelial growth factor.

Note: All ocular assessments are to be performed for both eyes unless annotated with 'SE' (study eye) in the above schedule. All assessments should be performed on the same day. All study visits should be scheduled and projected based on the Day 1 visit date.

A. For subjects that discontinue the study early, the early termination assessments should be performed after a minimum of 30 days have passed from the last dosing visit. If a subject reports for a scheduled visit and decides to terminate early prior to dosing, then the visit should be considered the early termination visit and all early termination procedures should be performed. At Month 24, all subjects should be offered entry into an open-label study.

B. At Day 1 (Visit 2), confirm subject eligibility through reviewing the inclusion/ exclusion criteria and receive confirmation of eligibility from the reading center.

C. Significant medical/ surgical history from the previous 5 years. Anti-VEGF treatments (fellow eye) and invasive ocular procedures performed within the past 5 years and while on study should also be recorded. Any history of tobacco use should be recorded.

D. Obtain prior to fluorescein angiography and before study drug administration.

E. At screening, serum pregnancy should be performed for women of childbearing potential. If positive, subject is not eligible to continue in the study.

F. Beginning at Day 1, perform the urine pregnancy test for women of childbearing potential at each treatment visit. If positive, perform a serum pregnancy test. If serum test is positive, study drug should not be administered and an early term visit should be completed.

G. Only subjects that sign the separate consent for the clinical repository will have these samples collected. A 14-mL whole-blood sample will be collected at each of the specified visits.

H. Blood pressure, respiratory rate, heart rate, and temperature. On dosing days, vital signs should be taken predose.

I. Height and weight should be collected at screening.

J. Perform assessments prior to dilating the eyes.

K. In select countries, the MNREAD or Radner Reading Charts should be done during the study visit, prior to dilating the eyes. It should be performed monocularly first, then binocularly.

L. Microperimetry assessments will be performed post dilation. Data will be forwarded to the reading center.

M. To be administered by the masked site staff prior to any other assessments performed on that day. In-clinic assessments on the digital application should be completed after completion of all functional tests and quality of life measures prior to dilating the eyes.
N. At select sites, and for those subjects who decide to participate, the digital applications will be completed on an electronic device and will consist of assessments for visual function and reading speed. Subjects will receive training at Day 1 on the use of the digital applications and the electronic device and will perform the tests using the application in the clinic at Month 1, Month 2, Month 3, Month 6, Month 12, Month 18, and Month 24. Beginning at Day 1, subjects will complete the functional assessments weekly at home. Subjects should be instructed to complete the assessments on the same day each week and at approximately the same time of day each week, if possible.
O. FAF and SD-OCT images, near infrared reflectance, fluorescein angiograms, and fundus photographs will be performed for the study eye only on days where 'SE' is specified and for both eyes at all other visits as specified in the above schedule and will be sent to the reading center for evaluation (consult the reading center manual for specifics on image capture, processing, and transmission). Images should be captured prior to dosing on dosing days. If a subject misses a study visit or images cannot be obtained at a specific visit, study staff should make every effort to obtain images at the next scheduled visit. If new active CNV is suspected, SD-OCT, FFA and OCT-A (select sites) images must be collected and sent to the reading center for analysis.
P. Postinjection assessments should be performed within 5 minutes after dosing by the unmasked physician or study staff and should include a gross assessment of vision (finger-counting, hand motion, then light perception when applicable). If subject passes gross vision test, the subject may leave the site. If subject fails gross vision test, the tonometry should be performed. IOP should be ≤30 mm Hg in order for the subject to leave the site. If necessary, antiglaucomatous medication can be given to lower the IOP. If IOP is >30 mm Hg, assessments will continue every approximately 30 minutes from the previous measurement until the subject passes gross vision test and IOP is ≤30 mm Hg. Note: if the study eye is treated with a ranibizumab or aflibercept injection during the same visit as the study treatment (pegcetacoplan or sham), the treatment with ranibizumab or aflibercept must be performed first. The pre- and post- anti-VEGF IOP values must be measured and recorded on the eCRF.
Q. Starting at Day 1, study subjects will be contacted by study site staff within 4 ± 2 days after each study treatment visit (through Month 3) to collect any information on any safety concerns, decrease in vision, eye pain, unusual ocular events, or any new ocular symptoms in the study eye. If the physician determines that there are any safety concerns, a follow-up visit should be scheduled as soon as possible.
R. Record concomitant medications (ie, prescription and over-the-counter medications) used by the patient within 30 days of screening and throughout the subject's participation in the study.
S. In addition to the time points indicated on the study schedule, OCT-A should be performed at the time of any suspected new active CNV. If new active CNV is confirmed in the study eye, OCT-A should be repeated every 2 months for the study eye.
T. Administration of pegcetacoplan or sham can be done on separate days from the assessment visit if both days fall within the visit window. Administration of study treatment (pegcetacoplan or sham) can be done on a separate day from the assessment visit if both days fall within the visit window. If this occurs on the randomization visit, then the administration of pegcetacoplan or sham should be done within 3 days of randomization and after approval from the medical monitor. When study treatment administration is on a day other than a study visit, then the only assessment that must be done on the day of study treatment administration is preinjection IOP. During the COVID-19 pandemic, the following flexibility is allowed for IP administration: –8 days to + 15 days after medical monitor approval. Note, interval for consecutive injections must be at least 14 days.

Table 14 (TRACKED): COVID-19 VISIT SCHEDULE—Monthly Group—Month 13 to Month 24

	Treatment												Early Term[A]
**Visit #**	**15**	**16**	**17**	**18**	**19**	**20**	**21**	**22**	**23**	**24**	**25**	**26**	
**Day**	**390**	**420**	**450**	**480**	**510**	**540**	**570**	**600**	**630**	**660**	**690**	**720**	
**Week**	**52**	**56**	**60**	**64**	**68**	**72**	**76**	**80**	**84**	**88**	**92**	**96**	
**Month**	**13**	**14**	**15**	**16**	**17**	**18**	**19**	**20**	**21**	**22**	**23**	**24**	
**Window (+ or – days)**	**8**	**8**	**8**	**8**	**8**	**8**	**8**	**8**	**8**	**8**	**8**	**8**	
**Informed Consent / Assign Screening Number**													
**Demographic Data**													
**Inclusion/Exclusion Criteria [B]**													
**Medical/Surgical/Ocular History [C]**													
**Blood Draw—Safety Labs [D,E,F]**						~~x~~						x	x
**Urine Sample Collection [D,E,F]**						~~x~~						x	x
**Urine Pregnancy Test [D,E,F]**	x	x	x	x	x	x	x	x	x	x	x	x	
**Blood Draw—Anti-Pegcetacoplan Ab [D]**		~~x~~				~~x~~						x	x
**Blood Draw—Genotyping (if applicable) [D]**													
**Blood Draw for Clinical Repository (if applicable)[D,G]**												~~x~~	~~x~~
**Vital Signs [H]**	~~x~~	~~x~~	~~x~~	~~x~~	~~x~~	x	~~x~~	~~x~~	~~x~~	~~x~~	~~x~~	x	x
**Physical Examination [I]**												x	x
**BCVA [J]**	x	x	x	x	x	x	x	x	x	x	x	x	x
**LL-BCVA [I]**	~~x~~	~~x~~	~~x~~	~~x~~	~~x~~	x	~~x~~	~~x~~	~~x~~	~~x~~	~~x~~	x	x
**MNREAD or Radner Reading Charts (select countries) [J,K]**						~~x~~						x	x
**Mesopic Microperimetry[L]**						~~SE~~						x	x
**Slitlamp Examination**	x	x	x	x	x	x	x	x	x	x	x	x	x
**Endothelial Cell Count[S]**												x	x
**NEI VFQ-25 [M]**						~~x~~						x	x
**FRI [M]**						~~x~~						x	x
**Dilated Indirect Ophthalmoscopy**	x	x	x	x	x	x	x	x	x	x	x	x	x
**Home-Based Digital Applications [M,N,S]**						~~x~~						~~x~~	
**IOP Measurement**	x	x	x	x	x	x	x	x	x	x	x	x	x
**SD-OCT [O]**	~~x~~	~~x~~	~~x~~	~~x~~	~~x~~	x	~~x~~	~~x~~	~~x~~	~~x~~	~~x~~	x	x
**FAF [O]**		~~SE~~		~~SE~~		x		~~SE~~		~~SE~~		x	x
**NIR [O]**		~~SE~~		~~SE~~		x		~~SE~~		~~SE~~		x	x
**DCFP [O]**												x	x
**FFA [O]**												x	x

Table 14 (TRACKED): COVID-19 VISIT SCHEDULE—Monthly Group—Month 13 to Month 24

	Treatment												Early Term[A]
**Visit #**	**15**	**16**	**17**	**18**	**19**	**20**	**21**	**22**	**23**	**24**	**25**	**26**	
**Day**	**390**	**420**	**450**	**480**	**510**	**540**	**570**	**600**	**630**	**660**	**690**	**720**	
**Week**	**52**	**56**	**60**	**64**	**68**	**72**	**76**	**80**	**84**	**88**	**92**	**96**	
**Month**	**13**	**14**	**15**	**16**	**17**	**18**	**19**	**20**	**21**	**22**	**23**	**24**	
**Window (+ or – days)**	**8**	**8**	**8**	**8**	**8**	**8**	**8**	**8**	**8**	**8**	**8**	**8**	
**OCT-A[S]**						~~x~~ [S]						x [S]	X
**Study Eye Determination**													
**Randomization**													
**Pegcetacoplan administration or Sham Injection[T]**	x	x	x	x	x	x	x	x	x	x	x		
**Postinjection Assessment [P]**	x	x	x	x	x	x	x	x	x	x	x		
**Follow-Up Call [Q]**													
**Concomitant Medication/ Concomitant Ocular Procedures [R]**	x	x	x	x	x	x	x	x	x	x	x	x	x
**Adverse Events**	x	x	x	x	x	x	x	x	x	x	x	x	x

Abbreviations: BCVA=best corrected visual acuity; CNV=choroidal neovascularization; DCFP=digital color fundus photography; eCRF=electronic case report form; FAF=fundus autofluorescence; FFA=fundus fluorescein angiography; FRI=Functional Reading Independence index; IOP=intraocular pressure; LL BCVA= low luminance best corrected visual acuity; MNREAD=Minnesota Low-Vision Reading Test; NEI VFG-25=National Eye Institute Visual Functioning Questionnaire 25-item Version; NIR=near infrared reflectance; OCT-A=optical coherence tomography angiography; SD-OCT=spectral domain optical coherence tomography; SE=study eye; Term=termination; VEGF=vascular endothelial growth factor.

Note: All ocular assessments are to be performed for both eyes unless annotated with 'SE' (study eye) in the above schedule. All assessments should be performed on the same day. All study visits should be scheduled and projected based on the Day 1 visit date.

A. For subjects that discontinue the study early, the early termination assessments should be performed after a minimum of 30 days have passed from the last dosing visit. If a subject reports for a scheduled visit and decides to terminate early prior to dosing, then the visit should be considered the early termination visit and all early termination procedures should be performed. At Month 24, all subjects should be offered entry into an open-label study.

B. At Day 1 (Visit 2), confirm subject eligibility through reviewing the inclusion/ exclusion criteria and receive confirmation of eligibility from the reading center.

C. Significant medical/ surgical history from the previous 5 years. Anti-VEGF treatments (fellow eye) and invasive ocular procedures performed within the past 5 years and while on study should also be recorded. Any history of tobacco use should be recorded.

D. Obtain prior to fluorescein angiography and before study drug administration.

E. At screening, serum pregnancy should be performed for women of childbearing potential. If positive, subject is not eligible to continue in the study.

F. Beginning at Day 1, perform the urine pregnancy test for women of childbearing potential at each treatment visit. If positive, perform a serum pregnancy test. If serum test is positive, study drug should not be administered and an early term visit should be completed.

G. Only subjects that sign the separate consent for the clinical repository will have these samples collected. A 14-mL whole-blood sample will be collected at each of the specified visits.

H. Blood pressure, respiratory rate, heart rate, and temperature. On dosing days, vital signs should be taken predose.

I. Height and weight should be measured at screening.

J. Perform assessments prior to dilating the eyes.

K. In select countries, the MNREAD or Radner Reading Charts should be done during the study visit, prior to dilating the eyes. It should be performed monocularly first, then binocularly.

L. Microperimetry assessments will be performed post dilation. Data will be forwarded to the reading center.
M. To be administered by the masked site staff prior to any other assessments performed on that day. In-clinic assessments on the digital application should be completed after completion of all functional tests and quality of life measures prior to dilating the eyes.
N. At select sites, and for those subjects who decide to participate, the digital applications will be completed on an electronic device and will consist of assessments for visual function and reading speed. Subjects will receive training at Day1 on the use of the digital applications and the electronic device and will perform the tests using the application in the clinic at Month 1, Month 2, Month 3, Month 6, Month 12, Month 18, and Month 24. Beginning at Day 1, subjects will complete the functional assessments weekly at home. Subjects should be instructed to complete the assessments on the same day each week and at approximately the same time of day each week, if possible.
O. FAF and SD-OCT images, near infrared reflectance, fluorescein angiograms, and fundus photographs will be performed for the study eye only on days where 'SE' is specified and for both eyes at all other visits as specified in the above schedule and will be sent to the reading center for evaluation (consult the reading center manual for specifics on image capture, processing, and transmission). Images should be captured prior to dosing on dosing days. If a subject misses a study visit or images cannot be obtained at a specific visit, study staff should make every effort to obtain images at the next scheduled visit. If new active CNV is suspected, SD-OCT, FFA, and OCT-A (selected sites) images should be collected and sent to the reading center for analysis.
P. Postinjection assessments should be performed within 5 minutes after dosing by the unmasked physician or study staff and should include a gross assessment of vision (finger-counting, hand motion, then light perception when applicable). If subject passes gross vision test, the subject may leave the site. If subject fails gross vision test, the tonometry should be performed. IOP should be ≤30 mm Hg in order for the subject to leave the site. If necessary, antiglaucomatous medication can be given to lower the IOP. If IOP is >30 mm Hg, assessments will continue every approximately 30 minutes from the previous measurement until the subject passes gross vision test and IOP is ≤30 mm Hg. Note: if the study eye is treated with a ranibizumab or aflibercept injection during the same visit as the study treatment (pegcetacoplan or sham), the treatment with ranibizumab or aflibercept must be performed first. The pre- and post- anti-VEGF IOP values must be measured and recorded on the eCRF.
Q. Starting at Day 1, study subjects will be contacted by study site staff within 4 ± 2 days after each study treatment visit (through Month 3) to collect any information on any safety concerns, decrease in vision, eye pain, unusual ocular events, or any new ocular symptoms in the study eye. If the physician determines that there are any safety concerns, a follow-up visit should be scheduled as soon as possible.
R. Record concomitant medications (ie, prescription and over-the-counter medications) used by the patient within 30 days of screening and throughout the subject's participation in the study.
S. In addition to the time points indicated on the study schedule, OCT-A should be performed at the time of any suspected new active CNV. If new active CNV is confirmed in the study eye, OCT-A should be repeated every 2 months for the study eye.
T. Administration of pegcetacoplan or sham can be done on separate days from the assessment visit if both days fall within the visit window. Administration of study treatment (pegcetacoplan or sham) can be done on a separate day from the assessment visit if both days fall within the visit window. If this occurs on the randomization visit, then the administration of pegcetacoplan or sham should be done within 3 days of randomization and after approval from the medical monitor. When study treatment administration is on a day other than a study visit, then the only assessment that must be done on the day of study treatment administration is preinjection IOP. During the COVID-19 pandemic, the following flexibility is allowed for IP administration: –8 days to + 15 days after medical monitor approval. Note, interval for consecutive injections must be at least 14 days.

Table 15 (TRACKED): COVID-19 VISIT SCHEDULE—Every-Other-Month Group—Screening, Day 1 Through Month 12

	Screening	Treatment													Early Term[A]
Visit #	1	2	3	4	5	6	7	8	9	10	11	12	13	14	
Day	–28 to –1	1	30	60	90	120	150	180	210	240	270	300	330	360	
Week	0	0	4	8	12	16	20	24	28	32	36	40	44	48	
Month	0	0	1	2	3	4	5	6	7	8	9	10	11	12	
Window (+ or – days)	2	0	8	8	8	8	8	8	8	8	8	8	8	8	
**Informed Consent / Assign Screening Number**	x														
**Demographic Data**	x														
**Inclusion/Exclusion Criteria** [B]	x	x													
**Medical/Surgical/Ocular History** [C]	x														
**Blood Draw—Safety Labs** [D,E,F]	x	x		~~x~~				~~x~~						x	x
**Urine Sample Collection** [D,E,F]	x	x		~~x~~				~~x~~						x	x
**Urine Pregnancy Test** [D,E,F]		x		x		x		x		x		x		x	
**Blood Draw—Anti-Pegcetacoplan Ab** [D]		x	~~x~~	~~x~~				~~x~~						x	x
**Blood Draw—Genotyping (if applicable)** [D]				~~x~~											
**Blood Draw for Clinical Repository (if applicable)** [D,G]				~~x~~				~~x~~						~~x~~	~~x~~
**Vital Signs** [H]	x	x	~~x~~	~~x~~	~~x~~	~~x~~	~~x~~	x	~~x~~	~~x~~	~~x~~	~~x~~	~~x~~	x	x
**Physical Examination** [I]	x													x	x
**BCVA** [J]	x	x	~~x~~	x	~~x~~	x	~~x~~	x	~~x~~	x	~~x~~	x	~~x~~	x	x
**LL-BCVA** [J]		x	~~x~~	~~x~~	~~x~~	~~x~~	~~x~~	x	~~x~~	~~x~~	~~x~~	~~x~~	~~x~~	x	x
**MNREAD or Radner Reading Charts (select countries)** [J,K]		x						~~x~~						x	x
**Mesopic Microperimetry**[L]	x							~~SE~~						x	x
**Slitlamp Examination**	x	x	~~x~~	x	~~x~~	x	~~x~~	x	~~x~~	x	~~x~~	x	~~x~~	x	x
**Endothelial Cell Count**[S]		x						x						x	x
**NEI VFQ-25** [M]		x						~~x~~						x	x
**FRI** [M]		x						~~x~~						x	x
**Home-Based Digital Applications** [M,N,S]		x	~~x~~	~~x~~	~~x~~			~~x~~						x	
**Dilated Indirect Ophthalmoscopy**	x	x	~~x~~	x	~~x~~	x	~~x~~	x	~~x~~	x	~~x~~	x	~~x~~	x	x
**IOP Measurement**	x	x	~~x~~	x	~~x~~	x	~~x~~	x	~~x~~	x	~~x~~	x	~~x~~	x	x
**SD-OCT** [O]	x	x	~~x~~	~~x~~	~~x~~	~~x~~	~~x~~	x	~~x~~	~~x~~	~~x~~	~~x~~	~~x~~	x	x
**FAF** [O]	x	x		~~SE~~		~~SE~~		x		~~SE~~		~~SE~~		x	x
**NIR** [O]	x	x		~~SE~~		~~SE~~		x		~~SE~~		~~SE~~		x	x
**DCFP** [O]	x													x	x

Table 15 (TRACKED): COVID-19 VISIT SCHEDULE—Every-Other-Month Group—Screening, Day 1 Through Month 12

	Screening	Treatment													Early Term[A]
**Visit #**	**1**	**2**	**3**	**4**	**5**	**6**	**7**	**8**	**9**	**10**	**11**	**12**	**13**	**14**	
**Day**	**–28 to –1**	**1**	**30**	**60**	**90**	**120**	**150**	**180**	**210**	**240**	**270**	**300**	**330**	**360**	
**Week**	**0**	**0**	**4**	**8**	**12**	**16**	**20**	**24**	**28**	**32**	**36**	**40**	**44**	**48**	
**Month**	**0**	**0**	**1**	**2**	**3**	**4**	**5**	**6**	**7**	**8**	**9**	**10**	**11**	**12**	
**Window (+ or – days)**	**2**	**0**	**8**	**8**	**8**	**8**	**8**	**8**	**8**	**8**	**8**	**8**	**8**	**8**	
**FFA [O]**	x													x	x
**OCT-A[S]**		x[S]						~~x~~[S]						x[S]	x
**Study Eye Determination**	x														
**Randomization**		x													
**Pegcetacoplan administration or Sham Injection[T]**		x		x		x		x		x		x		x	
**Postinjection Assessment [P]**		x		x		x		x		x		x		x	
**Follow-Up Call [Q]**		x		x		x									
**Concomitant Medication/ Concomitant Ocular Procedures [R`]**	x	x	~~x~~	x	~~x~~	x	~~x~~	x	~~x~~	x	~~x~~	x	~~x~~	x	x
**Adverse Events**	x	x	~~x~~	x	~~x~~	x	~~x~~	x	~~x~~	x	~~x~~	x	~~x~~	x	x

Abbreviations: BCVA=best corrected visual acuity; CNV=choroidal neovascularization; DCFP=digital color fundus photography; eCRF=electronic case report form; FAF=fundus autofluorescence; FFA=fundus fluorescein angiography; FRI=Functional Reading Independence index; IOP=intraocular pressure; LL BCVA= low luminance best corrected visual acuity; MNREAD=Minnesota Low-Vision Reading Test; NEI VFG-25=National Eye Institute Visual Functioning Questionnaire 25-item Version; NIR=near infrared reflectance; OCT-A=optical coherence tomography angiography; SD-OCT=spectral domain optical coherence tomography; SE=study eye; Term=termination; VEGF=vascular endothelial growth factor.

Note: All ocular assessments are to be performed for both eyes unless annotated with 'SE' (study eye) in the above schedule. All assessments should be performed on the same day. All study visits should be scheduled and projected based on the Day 1 visit date.

A. For subjects that discontinue the study early, the early termination assessments should be performed after a minimum of 30 days have passed from the last dosing visit. If a subject reports for a scheduled visit and decides to terminate early prior to dosing, then the visit should be considered the early termination visit and all early termination procedures should be performed. At Month 24, all subjects should be offered entry into an open-label study.

B. At Day 1 (Visit 2), confirm subject eligibility through reviewing the inclusion/ exclusion criteria and receive confirmation of eligibility from the reading center.

C. Significant medical/ surgical history from the previous 5 years. Anti-VEGF treatments (fellow eye) and invasive ocular procedures performed within the past 5 years and while on study should also be recorded. Any history of tobacco use should be recorded.

D. Obtain prior to fluorescein angiography and before study drug administration.

E. At screening, serum pregnancy should be performed for women of childbearing potential. If positive, subject is not eligible to continue in the study.

F. Beginning at Day 1, perform the urine pregnancy test for women of childbearing potential at each treatment visit. If positive, perform a serum pregnancy test. If serum test is positive, study drug should not be administered and an early term visit should be completed.

G. Only subjects that sign the separate consent for genetic biorepository will have these samples collected. A 14-mL whole-blood sample will be collected at each of the specified visits.

H. Blood pressure, respiratory rate, heart rate, and temperature. On dosing days, vital signs should be taken predose.

I. Height and weight should be measured at screening.

J. Perform assessments prior to dilating the eyes.

K. In select countries, the MNREAD or Radner Reading Charts should be done during the study visit, prior to dilating the eyes. It should be performed monocularly first, then binocularly.
L. Microperimetry assessments will be performed post dilation. Data will be forwarded to the reading center.
M. To be administered by the masked site staff prior to any other assessments performed on that day. In-clinic assessments on the digital application should be completed after completion of all functional tests and quality of life measures prior to dilating the eyes.
N. At select sites, and for those subjects who decide to participate, the digital application will be completed on an electronic device and will consist of assessments for visual function and reading speed. Subjects will receive training at Day 1 on the use of the digital applications and the electronic device and will perform the tests using the in the clinic at Month 1, Month 2, Month 3, Month 6, Month 12, Month 18, and Month 24. Beginning at Day 1, subjects will complete the functional assessments weekly at home. Subjects should be instructed to complete the assessments on the same day each week and at approximately the same time of day each week, if possible.
O. FAF and SD-OCT images, near infrared reflectance, fluorescein angiograms, and fundus photographs will be performed for the study eye only on days where 'SE' is specified and for both eyes at all other visits as specified in the above schedule and will be sent to the reading center for evaluation (consult the reading center manual for specifics on image capture, processing, and transmission). Images should be captured prior to dosing on dosing days. If a subject misses a study visit or images cannot be obtained at a specific visit, study staff should make every effort to obtain images at the next scheduled visit. If new active CNV is suspected, SD-OCT, FFA, and OCT-A (selected sites) images should be collected and sent to the reading center for analysis.
P. Postinjection assessments should be performed within 5 minutes after dosing by the unmasked physician or study staff and should include a gross assessment of vision (finger-counting, hand motion, then light perception when applicable). If subject passes gross vision test, the subject may leave the site. If subject fails gross vision test, the tonometry should be performed. IOP should be ≤30 mm Hg in order for the subject to leave the site. If necessary, antiglaucomatous medication can be given to lower the IOP. If IOP is >30 mm Hg, assessments will continue every approximately 30 minutes from the previous measurement until the subject passes gross vision test and IOP is ≤30 mm Hg. Note: if the study eye is treated with a ranibizumab or aflibercept injection during the same visit as the study treatment (pegcetacoplan or sham), the treatment with ranibizumab or aflibercept must be performed first. The pre- and post- anti-VEGF IOP values must be measured and recorded on the eCRF.
Q. Starting at Day 1, study subjects will be contacted by study site staff within 4 ± 2 days after each study treatment visit (through Month 4) to collect any information on any safety concerns, decrease in vision, eye pain, unusual ocular events, or any new ocular symptoms in the study eye. If the physician determines that there are any safety concerns, a follow-up visit should be scheduled as soon as possible.
R. Record concomitant medications (ie, prescription and over-the-counter medications) used by the patient within 30 days of screening and throughout the subject's participation in the study.
S. In addition to the time points indicated on the study schedule, OCT-A should be performed at the time of any suspected new active CNV. If new active CNV is confirmed in the study eye, OCT-A should be repeated every 2 months for the study eye.
T. Administration of pegcetacoplan or sham can be done on separate days from the assessment visit if both days fall within the visit window. Administration of study treatment (pegcetacoplan or sham) can be done on a separate day from the assessment visit if both days fall within the visit window. If this occurs on the randomization visit, then the administration of pegcetacoplan or sham should be done within 3 days of randomization and after approval from the medical monitor. When study treatment administration is on a day other than a study visit, then the only assessment that must be done on the day of study treatment administration is preinjection IOP. During the COVID-19 pandemic, the following flexibility is allowed for IP administration: –8 days to + 30 days after medical monitor approval.

Table 16 (TRACKED): COVID-19 VISIT SCHEDULE—Every-Other-Month Group—Month 13 to Month 24

	Treatment												Early Term [A]
Visit #		15		16		17		18		19		20	
Day	390	420	450	480	510	540	570	600	630	660	690	720	
Week	52	56	60	64	68	72	76	80	84	88	92	96	
Month	13	14	15	16	17	18	19	20	21	22	23	24	
Window (+ or – days)		16		16		16		16		16		16	
Informed Consent / Assign Screening Number													
Demographic Data													
Inclusion/Exclusion Criteria [B]													
Medical/Surgical/Ocular History [C]													
Blood Draw—Safety Labs [D,E,F]						~~x~~						x	x
Urine Sample Collection [D, E,F]						~~x~~						x	x
Urine Pregnancy Test [D,E,F]		x		x		x		x		x		x	
Blood Draw—Anti-Pegcetacoplan Ab [D]		~~x~~				~~x~~						x	x
Blood Draw—Genotyping (if applicable) [D]													
Blood Draw for Clinical Repository (if applicable) [D,G]												~~x~~	~~x~~
Vital Signs [H]		~~x~~		~~x~~		x		~~x~~		~~x~~		x	x
Physical Examination [I]												x	x
BCVA [J]		x		x		x		x		x		x	x
LL-BCVA [J]		~~x~~		~~x~~		x		~~x~~		~~x~~		x	x
MNREAD or Radner Reading Charts (select countries) [J,K]						~~x~~						x	x
Mesopic Microperimetry [L]						~~SE~~						x	x
Slitlamp Examination		x		x		x		x		x		x	x
Endothelial Cell Count [S]												x	x
NEI VFQ-25 [M]						~~x~~						x	x
FRI [M]						~~x~~						x	x
Home-Based Digital Applications [M,N,S]						~~x~~						~~x~~	
Dilated Indirect Ophthalmoscopy		x		x		x		x		x		x	x
IOP Measurement		x		x		x		x		x		x	x
SD-OCT [O]		~~x~~		~~x~~		x		~~x~~		~~x~~		x	x
FAF [O]		~~SE~~		~~SE~~		x		~~SE~~		~~SE~~		x	x
NIR[O]		~~SE~~		~~SE~~		x		~~SE~~		~~SE~~		x	x
DCFP [O]												x	x
FFA [O]												x	x
OCT-A[S]						~~x~~[S]						x[S]	x

Table 16 (TRACKED): COVID-19 VISIT SCHEDULE—Every-Other-Month Group—Month 13 to Month 24

	Treatment												Early Term [A]
Visit #		15		16		17		18		19		20	
Day	390	420	450	480	510	540	570	600	630	660	690	720	
Week	52	56	60	64	68	72	76	80	84	88	92	96	
Month	13	14	15	16	17	18	19	20	21	22	23	24	
Window (+ or – days)		16		16		16		16		16		16	
**Study Eye Determination**													
**Randomization**													
**Pegcetacoplan administration or Sham Injection** [T]		x		x		x		x		x			
**Postinjection Assessment** [P]		x		x		x		x		x			
**Follow-Up Call** [Q]													
**Concomitant Medication/ Concomitant Ocular Procedures** [R]		x		x		x		x		x		x	x
**Adverse Events**		x		x		x		x		x		x	x

Abbreviations: BCVA=best corrected visual acuity; CNV=choroidal neovascularization; DCFP=digital color fundus photography; eCRF=electronic case report form; FAF=fundus autofluorescence; FFA=fundus fluorescein angiography; FRI=Functional Reading Independence index; IOP=intraocular pressure; LL BCVA= low luminance best corrected visual acuity; MNREAD=Minnesota Low-Vision Reading Test; NEI VFG-25=National Eye Institute Visual Functioning Questionnaire 25-item Version; NIR=near infrared reflectance; OCT-A=optical coherence tomography angiography; SD-OCT=spectral domain optical coherence tomography; SE=study eye; Term=termination; VEGF=vascular endothelial growth factor.

Note: All ocular assessments are to be performed for both eyes unless annotated with 'SE' (study eye) in the above schedule. All assessments should be performed on the same day. All study visits should be scheduled and projected based on the Day 1 visit date.

A. For subjects that discontinue the study early, the early termination assessments should be performed after a minimum of 30 days have passed from the last dosing visit. If a subject reports for a scheduled visit and decides to terminate early prior to dosing, then the visit should be considered the early termination visit and all early termination procedures should be performed. At Month 24, all subjects should be offered entry into an open-label study.

B. At Day 1 (Visit 2), confirm subject eligibility through reviewing the inclusion/ exclusion criteria and receive confirmation of eligibility from the reading center.

C. Significant medical/ surgical history from the previous 5 years. Anti-VEGF treatments (fellow eye) and invasive ocular procedures performed within the past 5 years and while on study should be recorded. Any history of tobacco use should be recorded.

D. Obtain prior to fluorescein angiography and before study drug administration.

E. At screening, serum pregnancy should be performed for women of childbearing potential. If positive, subject is not eligible to continue in the study.

F. Beginning at Day 1, perform the urine pregnancy test for women of childbearing potential at each treatment visit. If positive, perform a serum pregnancy test. If serum test is positive, study drug should not be administered and an early term visit should be completed.

G. Only subjects that sign the separate consent for the clinical repository will have these samples collected. A 14-mL whole-blood sample will be collected at the specified time points.

H. Blood pressure, respiratory rate, heart rate, and temperature. On dosing days, vital signs should be taken pre- dose.

I. Height and weight should be measured at screening.

J. Perform assessments prior to dilating the eyes.

K. In select countries, the MNREAD or Radner Reading Charts should be done during the study visit, prior to dilating the eyes. It should be performed monocularly first, then binocularly.

L. Microperimetry assessments will be performed post dilation. Data will be forwarded to the reading center.

M. To be administered by the masked site staff prior to any other assessments performed on that day. In-clinic assessments on the digital application should be completed after completion of all functional tests and quality of life measures prior to dilating the eyes.

N. At select sites, and for those subjects who decide to participate, the digital applications will be completed on an electronic device and will consist of assessments for visual function and reading speed. Subjects will receive training at Day 1 on the use of the digital applications and the electronic device and will perform the tests using the application in the clinic at Month 1, Month 2, Month 3, Month 6, Month 12, Month 18, and Month 24. Beginning at Day 1, subjects will complete the functional assessments weekly at home. Subjects should be instructed to complete the assessments on the same day each week and at approximately the same time of day each week, if possible.
O. FAF and SD-OCT images, near infrared reflectance, fluorescein angiograms, and fundus photographs will be performed for the study eye only on days where 'SE' is specified and for both eyes at all other visits as specified in the above schedule and will be sent to the reading center for evaluation (consult the reading center manual for specifics on image capture, processing, and transmission). Images should be captured prior to dosing on dosing days. If a subject misses a study visit or images cannot be obtained at a specific visit, study staff should make every effort to obtain images at the next scheduled visit. If new active CNV is suspected, SD-OCT, FFA, and OCT-A (selected sites) images should be collected and sent to the reading center for analysis.
P. Postinjection assessments should be performed within 5 minutes after dosing by the unmasked physician or study staff and should include a gross assessment of vision (finger-counting, hand motion, then light perception when applicable). If subject passes gross vision test, the subject may leave the site. If subject fails gross vision test, the tonometry should be performed. IOP should be ≤30 mm Hg in order for the subject to leave the site. If necessary, antiglaucomatous medication can be given to lower the IOP. If IOP is >30 mm Hg, assessments will continue every approximately 30 minutes from the previous measurement until the subject passes the gross vision test and IOP is ≤30 mm Hg. Note: if the study eye is treated with a ranibizumab or aflibercept injection during the same visit as the study treatment (pegcetacoplan or sham), the treatment with ranibizumab or aflibercept must be performed first. The pre- and post- anti-VEGF IOP values must be measured and recorded on the eCRF.
Q. Starting at Day 1, study subjects will be contacted by study site staff within 4 ± 2 days after each study treatment visit (through Month 4) to collect any information on any safety concerns, decrease in vision, eye pain, unusual ocular events, or any new ocular symptoms in the study eye. If the investigator determines that there are any safety concerns, a follow-up visit should be scheduled as soon as possible.
R. Record concomitant medications (ie, prescription and over-the-counter medications) used by the patient within 30 days of screening and throughout the subject's participation in the study.
S. In addition to the time points indicated on the study schedule, OCT-A should be performed at the time of any suspected new active CNV. If new active CNV is confirmed in the study eye, OCT-A should be repeated every 2 months for the study eye.
T. Administration of pegcetacoplan or sham can be done on separate days from the assessment visit if both days fall within the visit window. Administration of study treatment (pegcetacoplan or sham) can be done on a separate day from the assessment visit if both days fall within the visit window. If this occurs on the randomization visit, then the administration of pegcetacoplan or sham should be done within 3 days of randomization and after approval from the medical monitor. When study treatment administration is on a day other than a study visit, then the only assessment that must be done on the day of study treatment administration is preinjection IOP. During the COVID-19 pandemic, the following flexibility is allowed for IP administration: –16 days to + 30 days after medical monitor approval.

1.2. Sample of SAS Code

```
* The SAS codes in this section are shown as examples
* Some modifications (such as variables used in the model statement)
* may require further changes to reflect the methods specified in the
* SAP and analysis data structure
* e.g., change trtan to trtpn to reflect analysis w/ the ITT set;
```

1.2.1. Mixed Effect Model for Repeated Measure

```
*----------------------------------------------------
* Read Analysis data
*----------------------------------------------------
* In this example, repeated measures are available at
* avisitn = 0.5 (baseline), 2 (Month 2),
*   6 (Month 6), and 12 (Month 12)
*-----------------------------------------------------;

data ga;
 set adam.adga;
 if paramcd = 'SEGAORG' and
  avisitn in (0.5, 2, 6, 12) and
  ANL01FL = 'Y';

  * trtan (actual treatment[number])
  * 1 = APL-2 Monthly
  * 2 = APL-2 Every Other Month
  * 3 = pooled Sham group;
 if trtan in (4,5) then trtan = 3;

 * trt01 are trt02 are used in multiple imputation
 * trt01 = 1 if Sham group
 * trt02 = 1 if APL2 Monthly group
 * trt01 = trt02 = 0 for APL2 EOM group;
 if trtan = 3 then trt01= 1; else trt01 = 0;
  if trtan = 1 then trt02= 1; else trt02 = 0;

 * avisitc will be used in PROC TRANSPOSE;
 if avisitn = 0.5 then avisitc = '00'; * Baseline;
 if avisitn = 2 then avisitc = '02'; * Month 2;
 if avisitn = 6 then avisitc = '06'; * Month 6;
 if avisitn = 12 then avisitc = '12'; * Month 12;
run;

********************************;
*** MAIN ANALYSIS using MMRM ***;
********************************;

proc mixed data=ga method=reml covtest empirical;
  where avisitn NE 0.5; * exclude rows for baseline;
  * cnv (CNV in the fellow eye)
  * 0 = No
  * 1 = Yes;
  *base_cat (baseline lesion size (<7.5 vs. ≥ 7.5);
  class trtan cnv avisitn usubjid base_cat;
  model chg = trtan cnv base_cat avisitn trtan*avisitn base_cat*avisitn
  /cl;
```

```
 repeated avisitn / subject=usubjid type=un r;
 lsmeans trtan*avisitn/pdiff cl e alpha=.05;
 ods output diffs= diff1 LSMeans= LSMean1;
run;
```

1.2.2. Multiple Imputation

```
********************************;
*** RESHAPE DATA FOR PROC MI ***;
********************************;

proc sort data= ga;
 by usubjid cnv trta trtan trt01 trt02 param paramcd base base_cat avisitc;
run;

proc transpose data= ga out= ga_t(drop= _NAME_ _LABEL_) prefix=V;
 by usubjid cnv trta trtan trt01 trt02 param paramcd base base_cat;
 id avisitc;
 var aval;
run;

* Examine the missing patterns of the data;
proc mi data=ga_t nimpute=0;
 var cnv trtan v00 -- v12;
 ods output missPattern=pattern;
run;

*****************************************;
*** MCMC (impute nonmonotone missing) ***;
*****************************************;

* Below statements invoke MCMC procedure and specify IMPUTE=MONOTONE
 to turn the arbitrary missing patterns to monotone missing patterns
 under missing at random (MAR) assumption;

proc mi data=ga_t out=ga_t_mono seed= 12135541 nimpute=1000;
 mcmc chain=multiple impute= monotone displayinit
      initial=em(itprint);
 var cnv trt01 trt02 v00 -- v12;
run;

* Examine the missing patterns of the data;
proc mi data=ga_t_mono nimpute=0;
 var cnv trt01 trt02 v00 -- v12;
 ods output missPattern=pattern;
run;

*******************************************************************;
*** Control-based pattern imputation(impute monotone missing) ***;
*******************************************************************;
data ga_t_mono2;
      set ga_t_mono;
      **Identify the subjects in the active groups we want to keep
  imputed as MAR;
      **DSCREASN is just an example;
      if trtan in (1,2) and DCSREASN = 1 then mar = 1;
      else mar = 0;
```

```
      run;

**First impute general MAR based on regression method for all subjects to get
the true MAR;
proc mi data=ga_t_mono2 out=ga_step1 nimpute=1 seed=1284054;
 by _Imputation_;
 class trtan;
 monotone reg(v00 -- v12/details);
 var cnv trtan v00 -- v12;
run;

**Identify and keep the data from the subjects in the active group that
should remain as a MAR;
data ga_step2;
      set ga_step1;
      if Mar = 1;
      run;

**Update the dataset and keep the MAR imputed data for the subjects in the
active group that should remain as a MAR, all others remain to be imputed
with control based imputation;
data ga_t_mono3;
      update ga_t_mono2 ga_step2;
      by _Imputation_ subjid;
      run;

**Fill out the remaining missing where missing data should be imputed based
on the controls;
proc mi data=ga_t_mono3 out=ga_t_cbp nimpute=1 seed=1284054;
 by _imputation_;
 class trtan;
 var cnv v00 -- v12;
 monotone reg(/details);
 mnar model(v02 -- v12 / modelobs= (trtan='3'));
run;

**General control based (all monotone missing);
proc mi data=ga_t_mono out=ga_t_cbp2 nimpute=1 seed=1284054;
 by _imputation_;
 class trtan;
 var cnv v00 -- v12;
 monotone reg(/details);
 mnar model(v02 -- v12 / modelobs= (trtan='3'));
run;

*******************************************************************;
*** Delta-Adjusted Pattern Imputation (impute monotone missing) ***;
*******************************************************************;
**First identify the subjects in the active treatment groups that we want to
apply the shift parameter if they have monotone missing data;
data ga_t_mono2;
      set ga_t_mono;

      **This is just an example of a reason that we do not want to
  apply the shift parameter. The actual reasons are in the text;
      if DCSREAS1 = "Death" then flag = 1;
      else flag = 0;
```

```
      if trtan in (1,2) and flag = 0 then adjustthis = 1;
      else adjustthis = 0;
      run;

proc mi data=ga_t_mono2 out=ga_t_tip6 nimpute=1 seed=9484353;
 by _imputation_;
 class trtan adjustthis;
 var cnv trtan v00 -- v12;
 monotone reg(/details);
 mnar adjust(v02 / shift=0.12 adjustobs=(adjustthis = '1' ))
  adjust(v06 / shift=0.12 adjustobs=(adjustthis = '1' ))
  adjust(v12 / shift=0.12 adjustobs=(adjustthis = '1' ));
run;
*****************************************************;
*** SAS macro to run MIANALYZE on outputs from MMRM *;
*****************************************************;

%macro mi_results(in, lsm, dif);

* After imputation, the data sets are in the wide format in which
 different variables (V00--V12) represent the outcome measured at
 different occasions. Before analyzing using PROC MIXED,
 the data sets need to be converted into long format in which one
 variable represents all outcome with different values of AVISITN
 differentiating different occasions;

proc transpose data= &in
    out= ga_mi (rename= (_NAME_ = AVISITN COL1 = AVAL));
 by _imputation_ usubjid trta trtan trt01 trt02 param paramcd base base_cat;
 var V00 -- V12;
run;

data ga_mi;
 set ga_mi;
 label AVISITN = 'AVISITN';
  chg = aval - base;
  * baseline record is not used in MMRM;
 if AVISITN = 'V00' then delete;
run;

proc sort; by _imputation_; run;

proc mixed data=ga_mi method=reml empirical;
 by _imputation_;
 class trtan cnv avisitn usubjid base_cat;
 model chg=trtan cnv base_cat avisitn trtan*avisitn base_cat*avisitn /cl;
 repeated avisitn / subject=usubjid type=UN;
 lsmeans trtan*avisitn / pdiff cl alpha=.05;
 ods output diffs= diffs lsmeans= lsmeans;
run;

* In the final step, the analysis results obtained from PROC MIXED
  procedure are combined into a single estimation with standard error
  using PROC MIANALYZE;

proc sort data=lsmeans;
```

```
 by avisitn trtan _imputation_;
run;
proc mianalyze parms=lsmeans;
 by avisitn trtan;
 modeleffects trtan*avisitn;
 ods output ParameterEstimates=&lsm;
run;

proc sort data=diffs (where= (_trtan= 3 and (avisitn = _avisitn)));
 by avisitn trtan _trtan _imputation_;
run;

proc mianalyze parms=diffs;
 by avisitn trtan _trtan;
 modeleffects trtan*avisitn;
 ods output ParameterEstimates=&dif;
run;

proc sql; *** LSMEANS using Multiple Imputation;
  select Parm as Effect, AVISITN, TRTAN, Estimate, StdErr, DF,
    tValue, Probt, LCLMean as L95, UCLMean as U95
 from &lsm;

  select Parm as Effect, AVISITN, TRTAN, _TRTAN, Estimate, StdErr, DF,
    tValue, Probt, LCLMean as L95, UCLMean as U95
 from &dif;
quit;
%mend;

%mi_results(in= ga_t_mcmc, lsm= lsm_mcmc, dif=dif_mcmc);
%mi_results(in= ga_t_reg, lsm= lsm_reg, dif=dif_reg );
%mi_results(in= ga_t_cbp, lsm= lsm_cbp, dif=dif_cbp );
%mi_results(in= ga_t_tip6, lsm= lsm_tip6, dif=dif_tip6);
```

1.2.3. Rate of Change Models

```
*********************************;
*** Rate of Change analyses ***;

* In this example, repeated measures are available at
* avisitn = 0 (baseline), 2 (Month 2), 4 (Month 4)
*   6 (Month 6), 8 (Month 8), 10 (Month 10) and 12 (Month 12)
*
* AVAL is the actual GA lesion measurement at the corresponding visit * for
the study eye from ADGA.
* SGALESAT is the GA lesion size (categorical) at baseline
* FCNVAT is the fellow eye CNV status at baseline
* TR01PG2N is the pooled treatment group (1 = PM, 2 = PEOM, 3 = Sham)
* T is equivalent to AVISITN
*
* The data is read in from ADGA
*********************************;


proc mixed data=ga method=reml covtest empirical;
```

```
    class subjid TR01PG2N FCNVAT SGALESAT t;
    model aval = SGALESAT TR01PG2N avisitn TR01PG2N * avisitn
          SGALESAT*avisitn FCNVAT/cl solution;
    repeated t /type=un sub=subjid r ;

    **Month 6;
    estimate 'PM Month 0 to 6 slope' TR01PG2N*avisitn 6 0 0
          avisitn 6 SGALESAT*avisitn 3 3 /cl;
    estimate 'PEOM Month 0 to 6 slope' TR01PG2N*avisitn 0 6 0
          avisitn 6 SGALESAT*avisitn 3 3 /cl;
    estimate 'Sham Month 0 to 6 slope' TR01PG2N*avisitn 0 0 6
          avisitn 6 SGALESAT*avisitn 3 3 /cl;

    estimate 'Month 0 to Month 6 Difference in slope PM - Sham'
          TR01PG2N*avisitn 6 0 -6 /cl;
    estimate 'Month 0 to Month 6 Difference in slope PEOM - Sham'
          TR01PG2N*avisitn 0 6 -6 /cl;
    estimate 'Month 0 to Month 6 Difference in slope PM - PEOM'
          TR01PG2N*avisitn 6 -6 0 /cl;

    **Month 12;
    estimate 'PM Month 6 to 12 slope' TR01PG2N*avisitn 6 0 0
          avisitn 6 SGALESAT*avisitn 3 3 /cl;
    estimate 'PEOM Month 6 to 12 slope' TR01PG2N*avisitn 0 6 0
          avisitn 6 SGALESAT*avisitn 3 3 /cl;
    estimate 'Sham Month 6 to 12 slope' TR01PG2N*avisitn 0 0 6
          avisitn 6 SGALESAT*avisitn 3 3 /cl;

    estimate 'Month 6 to 12 Difference in slope PM - Sham'
          TR01PG2N*avisitn 6 0 -6 /cl;
    estimate 'Month 6 to 12 Difference in slope PEOM - Sham'
          TR01PG2N*avisitn 0 6 -6 /cl;
    estimate 'Month 6 to 12 Difference in slope PM - PEOM'
          TR01PG2N*avisitn 6 -6 0 /cl;

    ods output diffs= diff2 LSMeans= LSMean2 CovParms = COV2
          estimates = estimates2;

    **Month 0 to 12;
    estimate 'PM Month 0 to 12 slope' TR01PG2N*avisitn 12 0 0
          avisitn 12 SGALESAT*avisitn 6 6 /cl;
    estimate 'PEOM Month 0 to 12 slope' TR01PG2N*avisitn 0 12 0
          avisitn 12 SGALESAT*avisitn 6 6 /cl;
    estimate 'Sham Month 0 to 12 slope' TR01PG2N*avisitn 0 0 12
          avisitn 12 SGALESAT*avisitn 6 6 /cl;

    estimate 'Month 0 to Month 12 Difference in slope PM - Sham'
          TR01PG2N*avisitn 12 0 -12 /cl;
    estimate 'Month 0 to Month 12 Difference in slope PEOM - Sham'
          TR01PG2N*avisitn 0 12 -12 /cl;
    estimate 'Month 0 to Month 12 Difference in slope PM - PEOM'
          TR01PG2N*avisitn 12 -12 0 /cl;

run;
```

```
* For the piecewise model, a knot at Month 6 is added to the dataset and
included in the model to allow for different slopes for the two 6 month
periods;

data ga2;
      set ga;

      k = 6;
      if avisitn <= k then avisitnspl1 = 0;
      if avisitn > k then avisitnspl1 = avisitn - k;
run;

proc mixed data=ga2 method=reml covtest empirical;
      class subjid TR01PG2N FCNVAT SGALESAT t;
      model aval = SGALESAT TR01PG2N avisitn avisitnspl1 TR01PG2N *
            avisitn TR01PG2N*avisitnspl1 SGALESAT*avisitn
            SGALESAT*avisitnspl1 FCNVAT/cl ;
      repeated t /type=un sub=subjid r ;

      **Month 6;
      estimate 'PM Month 0 to 6 slope' TR01PG2N*avisitn 6 0 0
            avisitn 6 SGALESAT*avisitn 3 3 /cl;
      estimate 'PEOM Month 0 to 6 slope' TR01PG2N*avisitn 0 6 0
            avisitn 6 SGALESAT*avisitn 3 3 /cl;
      estimate 'Sham Month 0 to 6 slope' TR01PG2N*avisitn 0 0 6
            avisitn 6 SGALESAT*avisitn 3 3 /cl;

      estimate 'Month 0 to Month 6 Difference in slope PM - Sham'
            TR01PG2N*avisitn 6 0 -6 /cl;
      estimate 'Month 0 to Month 6 Difference in slope PEOM - Sham'
            TR01PG2N*avisitn 0 6 -6 /cl;
      estimate 'Month 0 to Month 6 Difference in slope PM - PEOM'
            TR01PG2N*avisitn 6 -6 0 /cl;

      **Month 12;
      estimate 'PM Month 6 to 12 slope' TR01PG2N*avisitn 6 0 0
            avisitn 6 SGALESAT*avisitn 3 3 TR01PG2N*avisitnspl1 6 0 0
            avisitnspl1 6 SGALESAT*avisitnspl1 3 3 /cl;
      estimate 'PEOM Month 6 to 12 slope' TR01PG2N*avisitn 0 6 0
            avisitn 6 SGALESAT*avisitn 3 3 TR01PG2N*avisitnspl1 0 6 0
            avisitnspl1 6 SGALESAT*avisitnspl1 3 3 /cl;
      estimate 'Sham Month 6 to 12 slope' TR01PG2N*avisitn 0 0 6
            avisitn 6 SGALESAT*avisitn 3 3 TR01PG2N*avisitnspl1 0 0 6
            avisitnspl1 6 SGALESAT*avisitnspl1 3 3 /cl;

      estimate 'Month 6 to 12 Difference in slope PM - Sham'
            TR01PG2N*avisitn 6 0 -6 TR01PG2N*avisitnspl1 6 0 -6/cl;
      estimate 'Month 6 to 12 Difference in slope PEOM - Sham'
            TR01PG2N*avisitn 0 6 -6 TR01PG2N*avisitnspl1 0 6 -6/cl;
      estimate 'Month 6 to 12 Difference in slope PM - PEOM'
            TR01PG2N*avisitn 6 -6 0 TR01PG2N*avisitnspl1 6 -6 0/cl;

      ods output diffs= diff2 LSMeans= LSMean2 CovParms = COV2
            estimates = estimates2;
run;
```

1.2.4. Sample Code for Maximum Reading Speed and Critical Print Size

Please see below regarding the uncorrected and corrected reading speed calculation, and Critical Print Size: Flag = 1 MNREAD , 2 = Radner .

```
if flag = 1 then do;
   if (time  = 0 or notdone = 1) then urs = 0;
   /** "Not Done" indicates the print size was not attempted due to vision or
that the sentence was attempted but could not be read **/
   else if time > 0 then urs = 600/time;
   if urs > 300 then urs = 300;
    /** Calculate Corrected Reading Speed **/
   if (time = 0 or numerr >= 10) or (notdone = 1) then crs = 0;
   else if numerr >=0 and time > 0 then crs = 60*(10-numerr)/time;
   else if (numerr = 0 or time = 0) then crs = urs;
   if crs > 300 then crs = 300;
  end;
  if flag = 2 then do;
   if (time  = 0 or notdone = 1) then urs = 0;
   else if time > 0 then urs =  840/time;
   if urs > 300 then urs = 300;
   if (time = 0 or numerr >= 14) or (notdone = 1) then crs = 0;
   else if numerr >=0 and time  > 0 then crs = 60*(14-numerr)/time;
   else if (numerr = 0 or time = 0) then crs = urs;
   if crs > 300 then crs = 300;
end;
Critical Print Size:
if flag = 1 and n(aval,dist) =2 and dist ne 40 then aval = aval +
round(log10(40/dist),.01); /** Apply correction for viewing distance ne 40
**/
if flag = 2 and n(aval,dist) =2 and dist not in (32 40) then aval = aval -
0.1 + round(log10(40/dist),.01);
```
