# Supplementary material for: Pegcetacoplan Treatment and Consensus Features of Geographic Atrophy Over 24 Months
Source: JAMA Ophthalmol. 2024 May 9;142(6):548–58. doi: 10.1001/jamaophthalmol.2024.1269 (PMC11082756; doi:10.1001/jamaophthalmol.2024.1269)
Supplement: Supplement 2. — eFigure 1. Segmentation of ETDRS regions from SD-OCT imaging eFigure 2. CONSORT flow diagram eFigure 3. SD-OCT biomarkers of GA at baseline eFigure 4. Difference in least squares mean growth of GA features substratified by the ETDRS region eFigure 5. Least squares mean change in GA features eFigure 6. Example of participants with slow GA growth (top) and with fast GA growth (bottom) over treatment period eFigure 7. Sensitivity analyses eTable 1. Analysis of change in area from baseline at 12, 18, and 24 months post baseline by ETDRS region eTable 2. Least squares mean change in best-corrected visual acuity from baseline at 12, 18, and 24 months post-baseline eTable 3. Spearman Correlation of Change from Baseline between GA SD-OCT feature and BCVA at Month 24 eTable 4. Change in area from baseline at 12, 18, and 24 months post baseline – Sensitivity analyses eMethods [file jamaophthalmol-e241269-s002.pdf]

Supplemental Online Content

Fu DJ, Bagga P, Naik G, et al. Pegcetacoplan treatment and consensus features of geographic atrophy over 24 months. *JAMA Ophthalmol*. Published online May 9, 2024.
doi:10.1001/jamaophthalmol.2024.1269

eFigure 1. Segmentation of ETDRS regions from SD-OCT imaging

eFigure 2. CONSORT flow diagram

eFigure 3. SD-OCT biomarkers of GA at baseline

eFigure 4. Difference in least squares mean growth of GA features substratified by the ETDRS region

eFigure 5. Least squares mean change in GA features

eFigure 6. Example of participants with slow GA growth (top) and with fast GA growth (bottom) over treatment period

eFigure 7. Sensitivity analyses

eTable 1. Analysis of change in area from baseline at 12, 18, and 24 months post baseline by ETDRS region

eTable 2. Least squares mean change in best-corrected visual acuity from baseline at 12, 18, and 24 months post-baseline

eTable 3. Supplementary Table 3. Spearman Correlation of Change from Baseline between GA SD-OCT feature and BCVA at Month 24

eTable 4. Change in area from baseline at 12, 18, and 24 months post baseline – Sensitivity analyses

eMethods

This supplemental material has been provided by the authors to give readers additional information about their work.

© 2024 Fu DJ et al. *JAMA Ophthalmology.*

SUPPLEMENTARY FIGURE LEGENDS

eFigure 1. Segmentation of ETDRS regions from SD-OCT imaging

Manual central foveal point annotation permitted interpolation of a given voxel's localisation in relation to the fovea and thereby consideration of retinal regions divided up into the Early Treatment Diabetic Retinopathy Study (ETDRS) grid of three concentric rings: the 1 mm diameter ETDRS central foveal region); the inner ETDRS ring of 3 mm diameter; and the outer perifoveal ETDRS ring with 6 mm diameter.

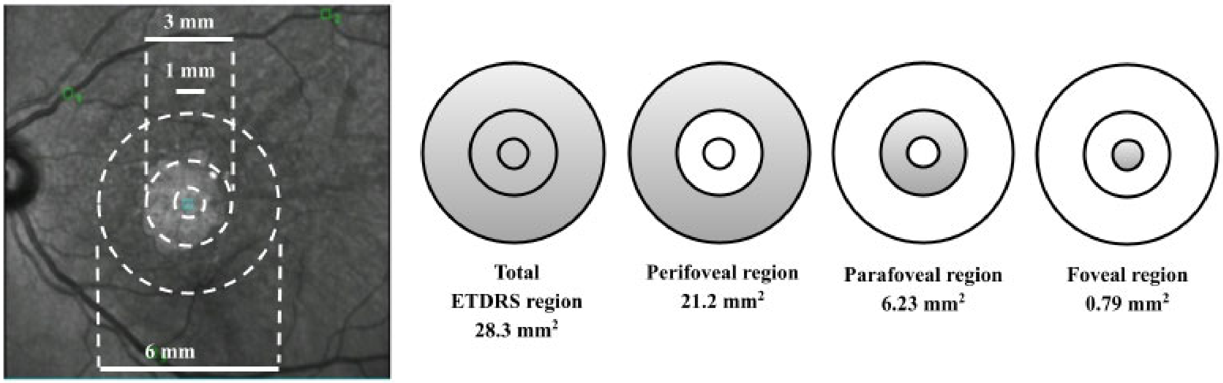

© 2024 Fu DJ et al. *JAMA Ophthalmology.*

eFigure 2. CONSORT flow diagram.

OAKS and DERBY were similar 24-month, phase 3, multicenter, randomized, double-masked, sham-controlled studies, which enrolled participants at 110 and 122 clinical sites worldwide, respectively. Those who did not consent or did not meet eligibility criteria were excluded from the study, and the remaining 1258 subjects were randomized 2:2:1:1 to pegcetacoplan monthly (PM), pegcetacoplan every other month (PEOM), sham treatment monthly (SM) and sham treatment every other month (SEOM). This study only considered participants who did met the following criteria: OCT imaging carried out on Heidelberg Spectralis system; OCTs with 25 B-scans or more per volume; OCT at baseline and at least 1 scan post-baseline; and at least 1 injection of pegceptacoplan.

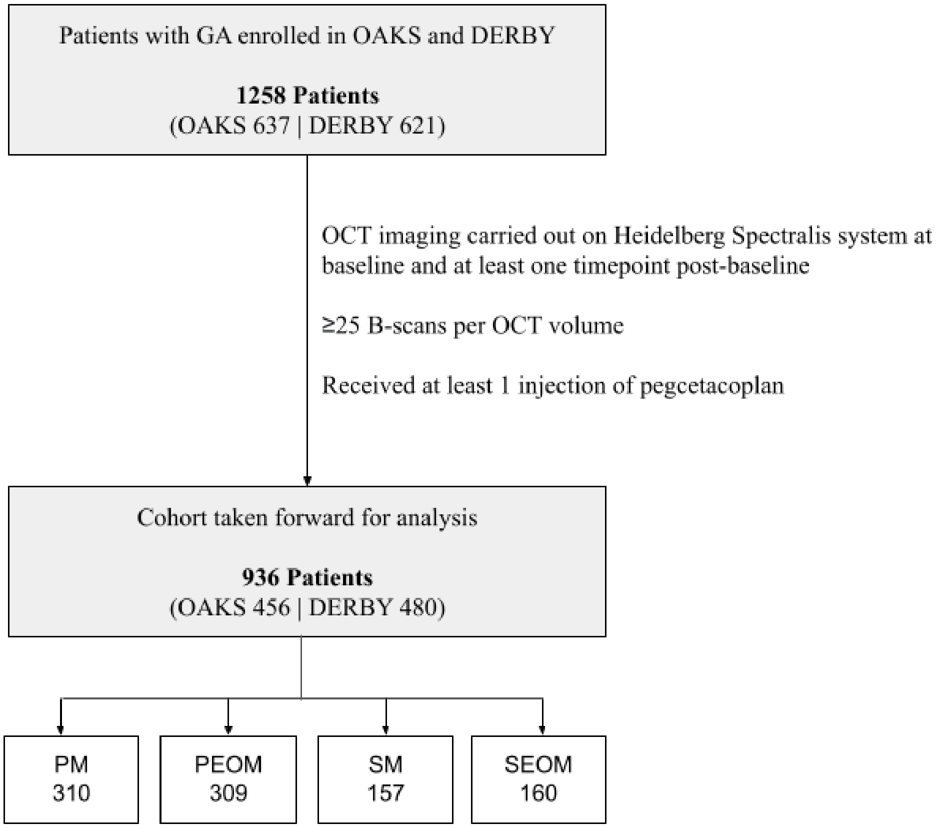

© 2024 Fu DJ et al. *JAMA Ophthalmology.*

eFigure 3. SD-OCT biomarkers of GA at baseline.

Boxplots of GA features segmented from SD-OCT included RPE-loss and outer retinal atrophy (RORA; red), RPE-loss (yellow), hypertransmission (HTD; green), photoreceptor degeneration (PRD; dark-blue), PRD in isolation (PRD [isolated]; light-blue) in mm^2 SD-OCT. Area of each GA features shown within (a) total ETDRS (early treatment diabetic retinopathy study); (b) foveal; (c) parafoveal; and (d) perifoveal regions. Mean area value expressed as absolute value in mm2 (left y-axis) and percentage occupancy of given ETDRS regions (right y-axis).

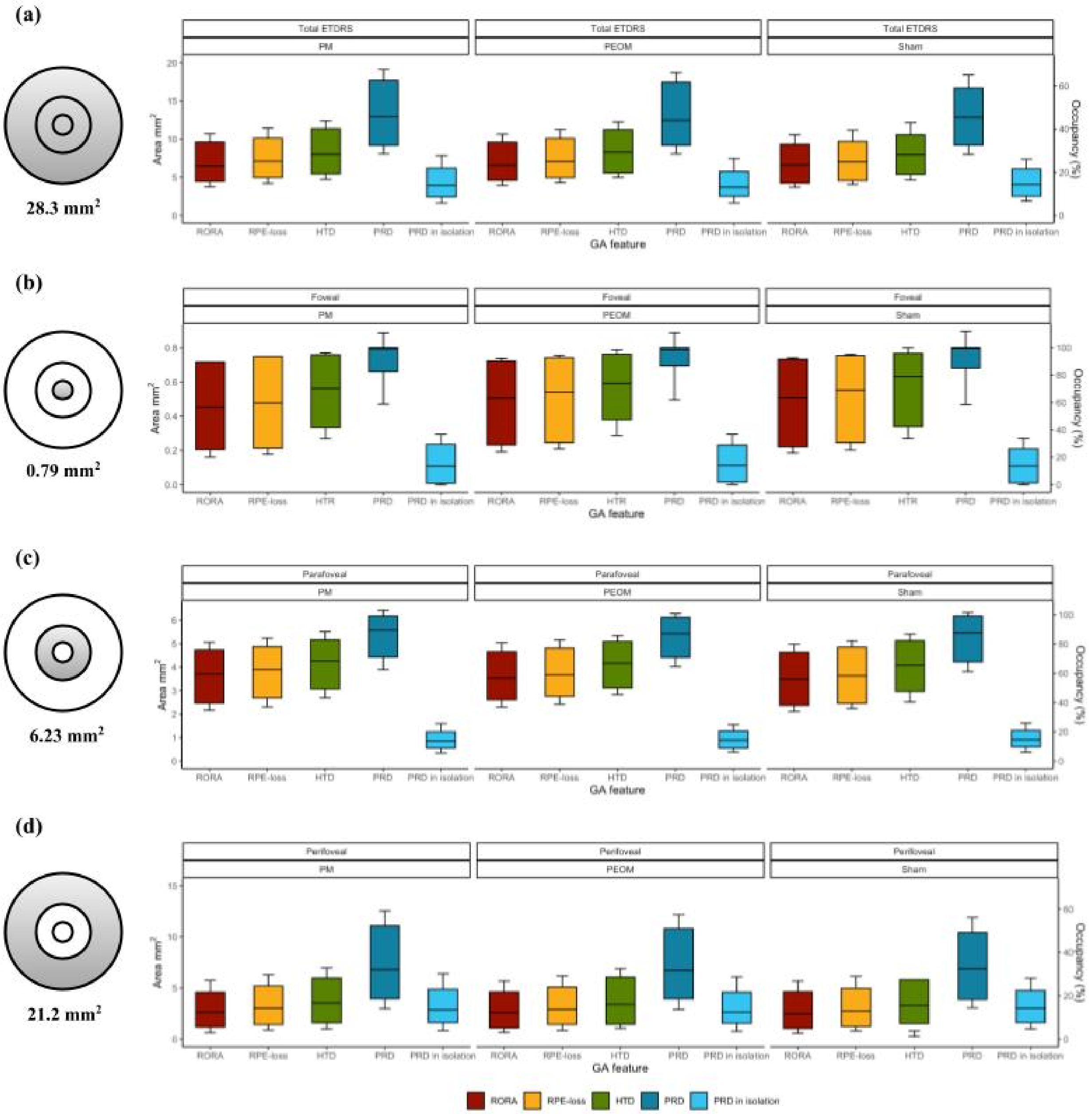

© 2024 Fu DJ et al. *JAMA Ophthalmology.*

eFigure 4. Difference in least squares mean growth of GA features substratified by the ETDRS region.

Differences between treatment groups (PEOM and pooled-sham, blue; PM and pooled-sham, red) in least square (LS) mean change in area size (mm2) of GA features from baseline. SD-OCT GA features considered are RPE-loss and outer retinal atrophy (RORA), RPE-loss, hypertransmission, photoreceptor degeneration (PRD), PRD in isolation in study eyes. Changes within (a) foveal; (b) parafoveal; and (c) perifoveal regions are shown. Graph showing LS means and standard error treatment group and month post baseline, which were estimated from a mixed effect model with a random intercept at the level of the participant that included the following as cross-level interactions: treatment, presence of CNV in the fellow eye (Yes or No); Baseline GA lesion area (< 7.5 mm2 or >= 7.5 mm2); Baseline area of SD-OCT GA feature; analysis visit; treatment x analysis visit; and baseline SD-OCT GA feature x analysis visit.baseline. PM = Pegceptacoplan monthly; PEOM = Pegceptacoplan every other month.

© 2024 Fu DJ et al. *JAMA Ophthalmology.*

(a)

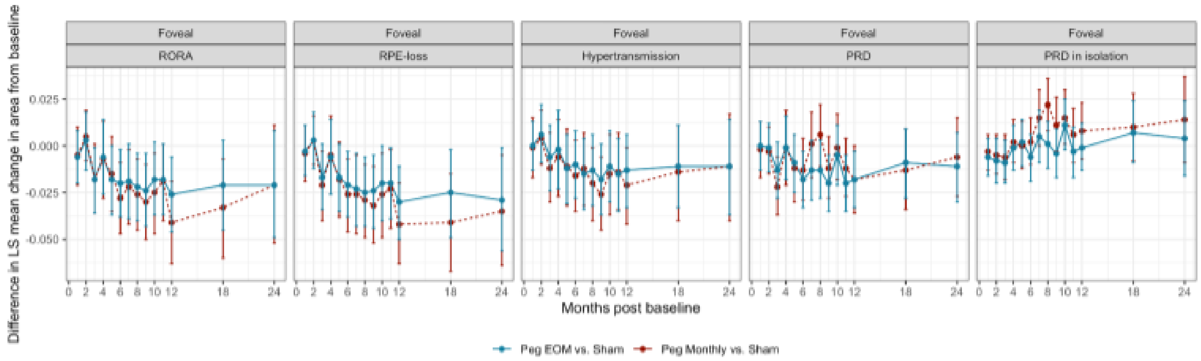

(b)

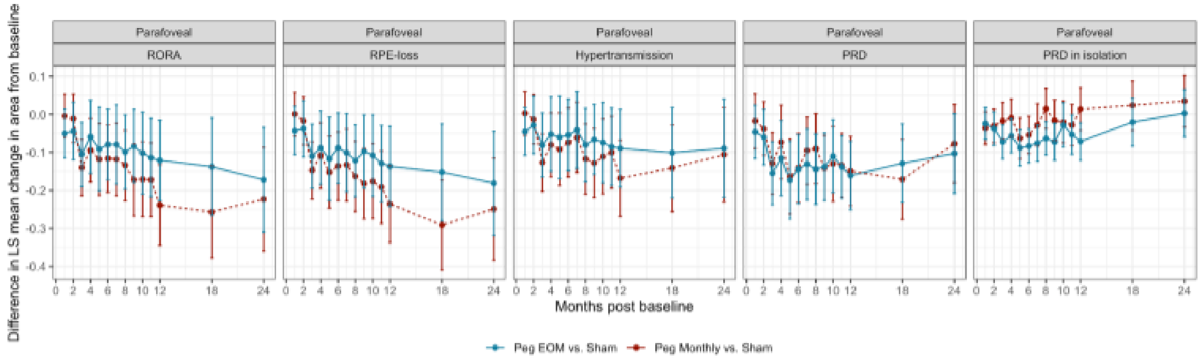

(c)

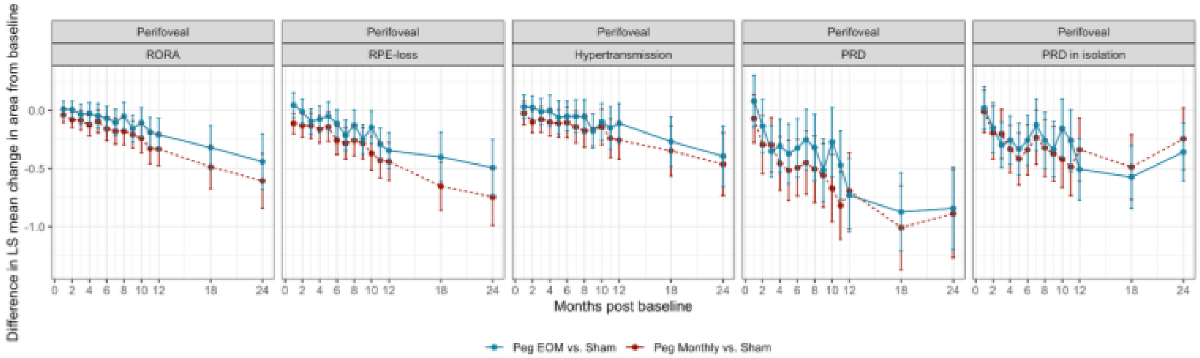

© 2024 Fu DJ et al. *JAMA Ophthalmology.*

eFigure 5. Least squares mean change in GA features.

Graph depicting the least square (LS) mean change in area size (mm2) of RPE-loss and outer retinal atrophy (RORA), RPE-loss, hypertransmission, photoreceptor degeneration (PRD), PRD in isolation in study eyes. Changes in GA feature were sub-stratified by relative location within macula: (a) total ETDRS regions; (b) foveal; (c) parafoveal; and (d) perifoveal regions. Graph showing least squares (LS) means and standard error treatment group and month post baseline, which were estimated from a mixed effect model with a random intercept at the level of the participant that included the following as cross-level interactions: treatment, presence of CNV in the fellow eye (Yes or No); Baseline GA lesion area (< 7.5 mm2 or >= 7.5 mm2); Baseline SD-OCT GA feature; analysis visit; treatment x analysis visit; and baseline SD-OCT GA feature x analysis visit.baseline. PM = Pegceptacoplan monthly; PEOM = Pegceptacoplan every other month.

© 2024 Fu DJ et al. *JAMA Ophthalmology.*

(a)

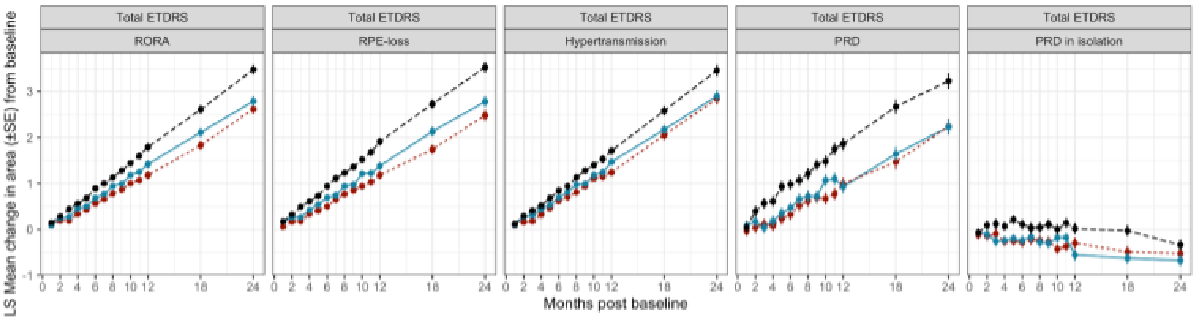

(b)

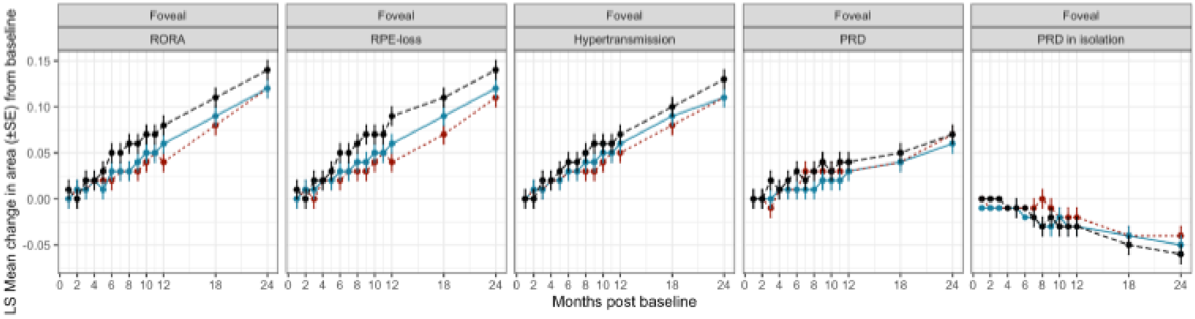

(c)

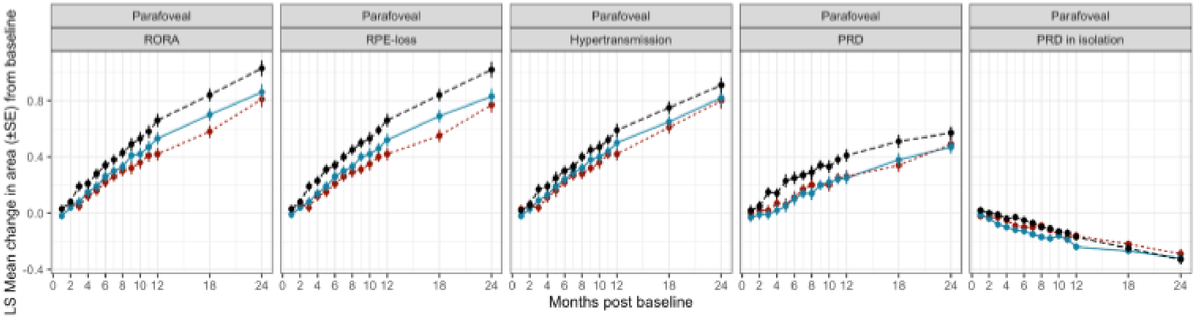

(d)

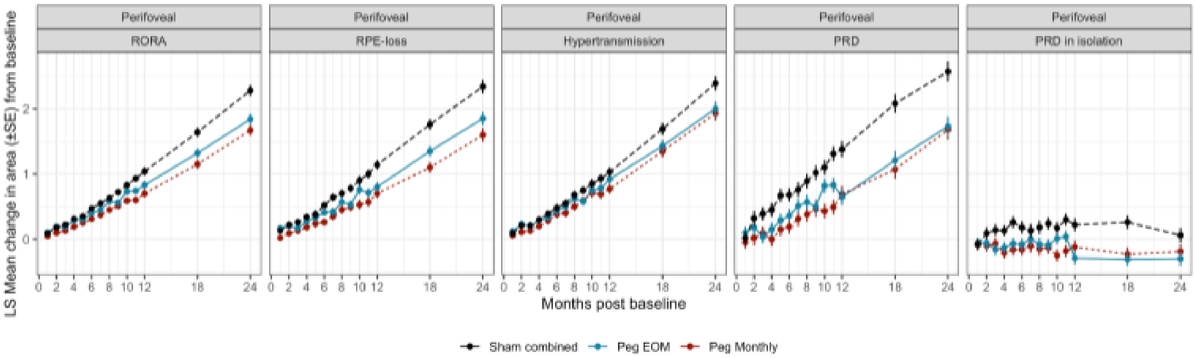

© 2024 Fu DJ et al. *JAMA Ophthalmology.*

eFigure 6. Example of participants with slow GA growth (top) and with fast GA growth (bottom) over treatment period.

Slow GA growth rate

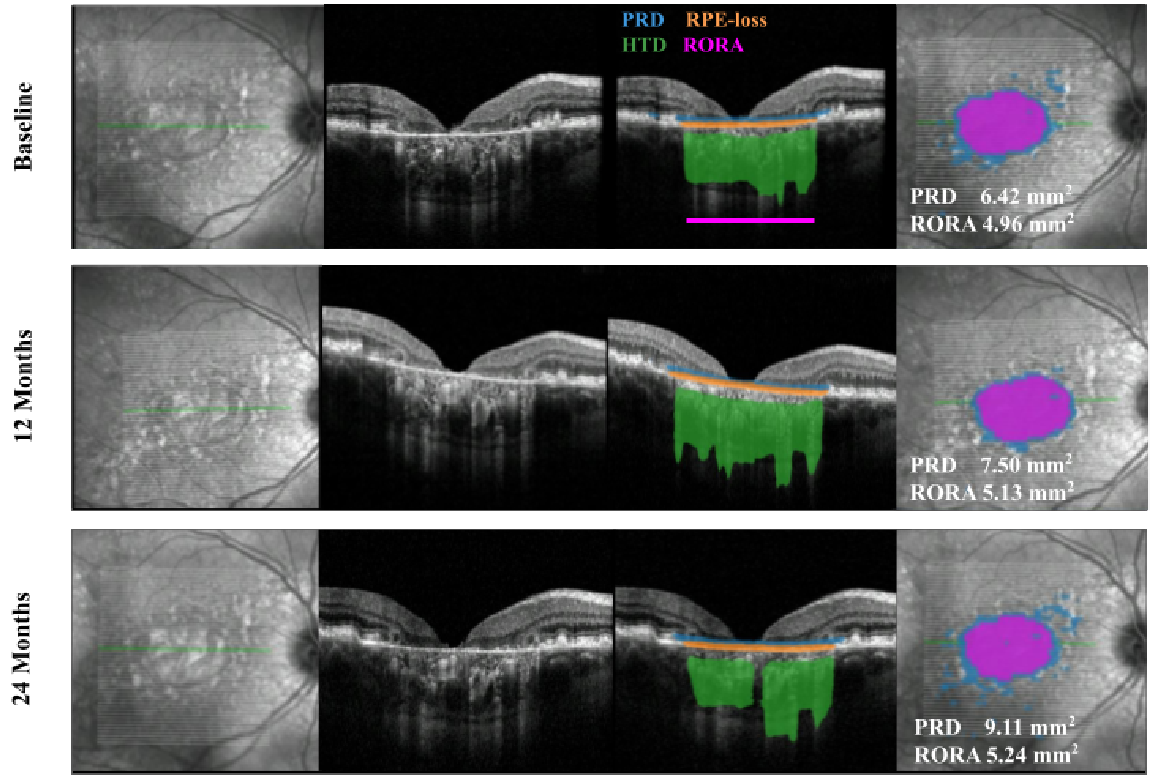

Fast GA growth rate

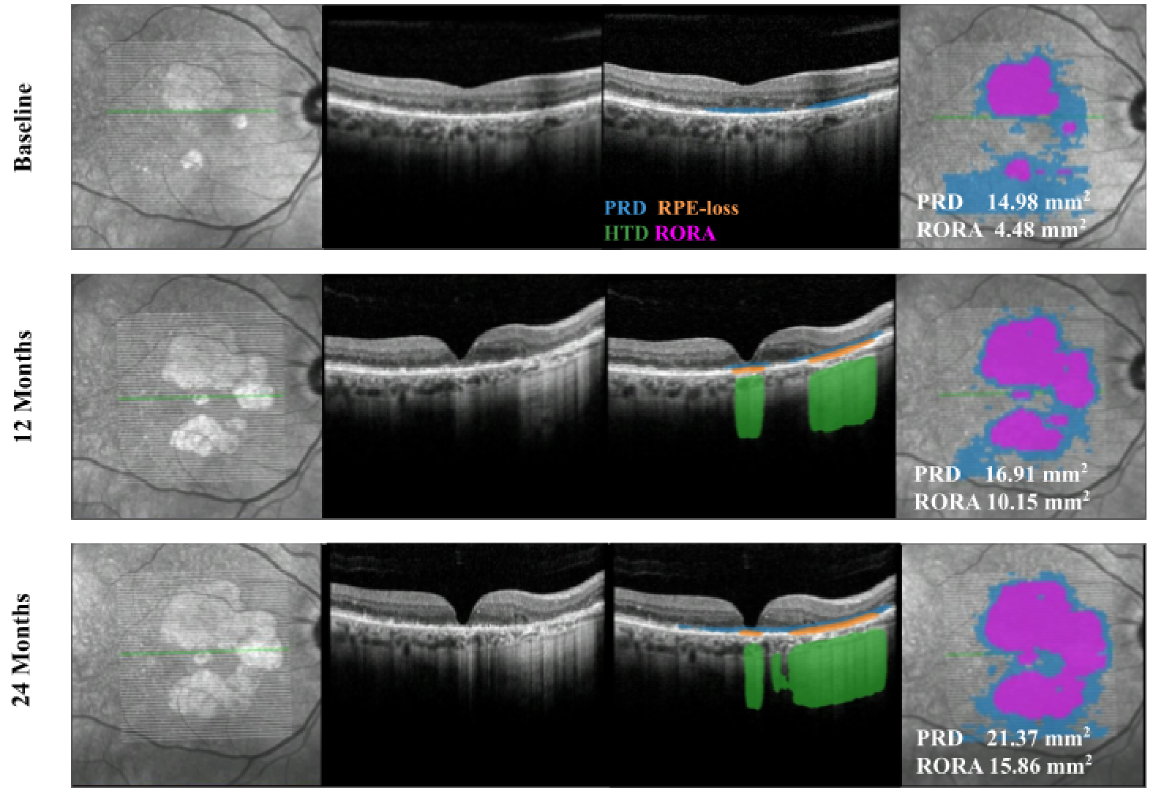

© 2024 Fu DJ et al. *JAMA Ophthalmology.*

eFigure 7. Sensitivity analyses.

Differences between treatment groups (PEOM and pooled-sham, blue; PM and pooled-sham, red) in mean area size (mm2) change of GA features from baseline. SD-OCT GA features considered are RPE-loss and outer retinal atrophy (RORA), RPE-loss, hypertransmission, photoreceptor degeneration (PRD), PRD in isolation in study eyes. Changes within total ETDRS regions were considered. Graph showing means and standard error treatment group and month post baseline using all available observed values without mixed-model effects. PM = Pegceptacoplan monthly; PEOM = Pegceptacoplan every other month.

© 2024 Fu DJ et al. *JAMA Ophthalmology.*

(a)

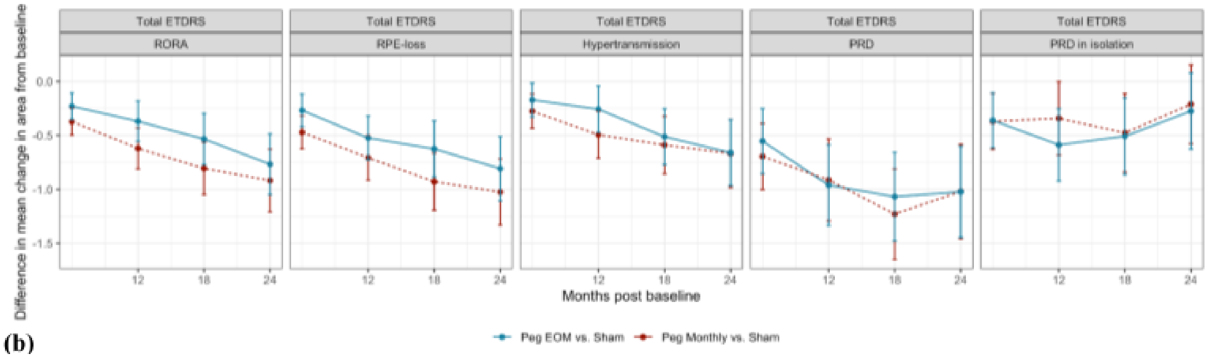

(b)

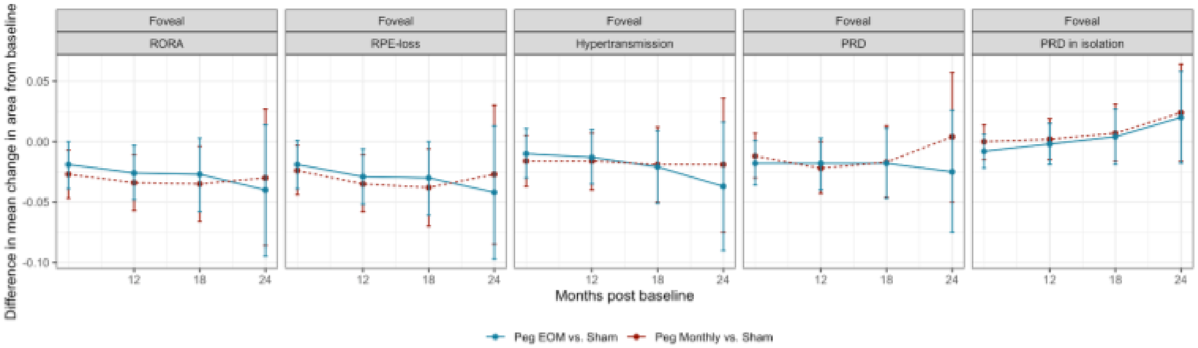

(c)

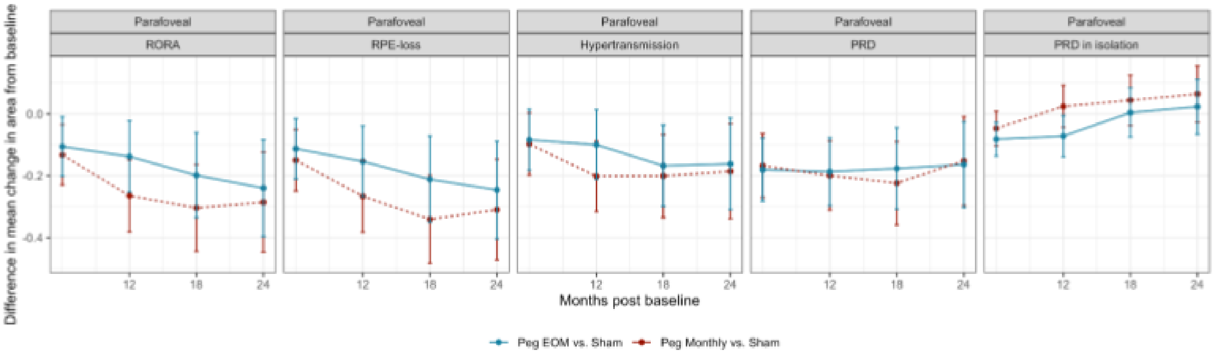

(d)

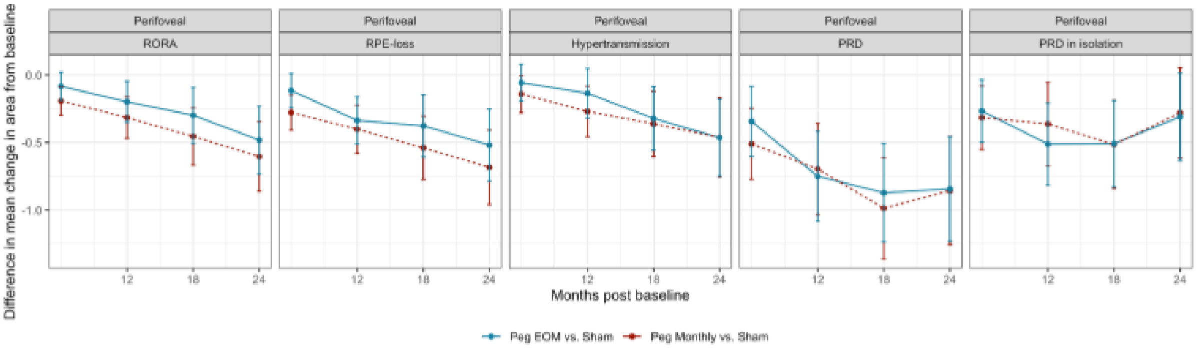

© 2024 Fu DJ et al. *JAMA Ophthalmology.*

eTable 1. Analysis of change in area from baseline at 12, 18, and 24 months post baseline by ETDRS region.

Table shows least square (LS) mean change of area, standard error (SE), absolute and percentage difference between treatment groups and 95% confidence interval (CI). Change in GA feature were sub-stratified by relative location within macula: foveal; parafoveal; and perifoveal regions. P-values shown for comparison of pegceptacoplan monthly (PM), pegceptacoplan every other month (PEOM), and pooled-shamsham-pooled when considering: RPE and outer retinal atrophy (RORA); RPE-loss; hypertransmission; photoreceptor degeneration (PRD); PRD in isolation.

Analysis of change in area (mm^2) from baseline to month 24 with MMRM model within central ETDRS foveal region (1 mm diameter; 0.79 mm^2)

	PM	P-value (PM vs. Pooled-Sham)	P-value (PM vs. PEOM)	PEOM	P-value (PEOM vs. Pooled-Sham)	Pooled-Sham
**RORA (mm^2)**						
LS Mean (SE)	0.12 (0.01)			0.12 (0.01)		0.14 (0.01)
Difference (95% CI) in LS Mean (vs Pooled-Sham)	-0.02 (-0.05, 0.01)	.19		-0.02 (-0.05, 0.01)	.16	
Percentage Difference (vs Pooled-Sham)	-14.90			-14.80		
Difference (95% CI) in LS Mean (vs PEOM)	-0.00 (-0.03, 0.03)		.99			
Percentage Difference (vs PEOM)	-0.20					
**RPE-loss (mm^2)**						
LS Mean (SE)	0.11 (0.01)			0.12 (0.01)		0.14 (0.01)
Difference (95% CI) in LS Mean (vs Pooled-Sham)	-0.03 (-0.06, -0.00)	.02		-0.03 (-0.06, -0.00)	.041	
Percentage Difference (vs Pooled-Sham)	-23.80			-19.80		

© 2024 Fu DJ et al. *JAMA Ophthalmology.*

Difference (95% CI) in LS Mean (vs PEOM)	-0.01 (-0.04, 0.03)		.71			
Percentage Difference (vs PEOM)	-5.10					
**Hypertransmission (mm^2)**						
LS Mean (SE)	0.11 (0.01)			0.11 (0.01)		0.13 (0.01)
Difference (95% CI) in LS Mean (vs Pooled-Sham)	-0.01 (-0.04, 0.02)	.43		-0.01 (-0.04, 0.01)	.37	
Percentage Difference (vs Pooled-Sham)	-9.10			-9.20		
Difference (95% CI) in LS Mean (vs PEOM)	0.00 (-0.03, 0.03)		.99			
Percentage Difference (vs PEOM)	0.10					
**Photoreceptor degeneration (PRD; mm^2)**						
LS Mean (SE)	0.07 (0.01)			0.06 (0.01)		07 (0.01)
Difference (95% CI) in LS Mean (vs Pooled-Sham)	-0.01 (-0.03, 0.01)	.56		-0.01 (-0.03, 0.01)	.23	
Percentage Difference (vs Pooled-Sham)	-8.50			-15.30		
Difference (95% CI) in LS Mean (vs PEOM)	0.01 (-0.02, 0.03)		.67			
Percentage Difference (vs PEOM)	8.00					
**PRD in isolation (mm^2)**						
LS Mean (SE)	-0.04 (0.01)			-0.05 (0.01)		.06 (0.01)
Difference (95% CI) in LS Mean (vs Pooled-Sham)	0.01 (-0.01, 0.04)	.23		0.00 (-0.02, 0.02)	.69	

© 2024 Fu DJ et al. *JAMA Ophthalmology.*

	PM	P-value (PM vs. Pooled-Sham)	P-value (PM vs. PEOM)	PEOM	P-value (PEOM vs. Pooled-Sham)	Pooled-Sham
Percentage Difference (vs Pooled-Sham)	-25.20			-7.40		
Difference (95% CI) in LS Mean (vs PEOM)	0.01 (-0.01, 0.03)		.38			
Percentage Difference (vs PEOM)	-19.20					

Parafoveal ETDRS region (6.23 mm^2) from baseline to month 24

	PM	P-value (PM vs. Pooled-Sham)	P-value (PM vs. PEOM)	PEOM	P-value (PEOM vs. Pooled-Sham)	Pooled-Sham
**RORA (mm^2)**						
LS Mean (SE)	0.81 (0.05)			0.86 (0.05)		1.03 (0.05)
Difference (95% CI) in LS Mean (vs Pooled-Sham)	0.22 (-0.36, -0.09)	.001		-0.17 (-0.31, -0.03)	.02	
Percentage Difference (vs Pooled-Sham)	-21.60			-16.60		
Difference (95% CI) in LS Mean (vs PEOM)	-0.05 (-0.19, 0.09)		.48			
Percentage Difference (vs PEOM)	-6.00					
**RPE-loss (mm^2)**						
LS Mean (SE)	0.77 (0.05)			0.83 (0.05)		1.02 (0.05)
Difference (95% CI) in LS Mean (vs Pooled-Sham)	0.25 (-0.38, -0.12)	<.001		-0.18 (-0.32, -0.04)	.009	
Percentage Difference (vs Pooled-Sham)	-24.60			-17.80		
Difference (95% CI) in LS Mean (vs PEOM)	-0.07 (-0.21, 0.07)		.34			

© 2024 Fu DJ et al. *JAMA Ophthalmology.*

Percentage Difference (vs PEOM)	-8.20					
**Hypertransmission (mm^2)**						
LS Mean (SE)	0.80 (0.05)			0.82 (0.05)		0.91 (0.04)
Difference (95% CI) in LS Mean (vs Pooled-Sham)	-0.11 (-0.23, 0.02)	.09		-0.09 (-0.22, 0.04)	.17	
Percentage Difference (vs Pooled-Sham)	-11.60			-9.80		
Difference (95% CI) in LS Mean (vs PEOM)	-0.02 (-0.15, 0.11)		.81			
Percentage Difference (vs PEOM)	-2.00					
**Photoreceptor degeneration (PRD; mm^2)**						
LS Mean (SE)	0.49 (0.04)			0.47 (0.04)		0.57 (0.04)
Difference (95% CI) in LS Mean (vs Pooled-Sham)	-0.08 (-0.18, 0.03)	.14		-0.10 (-0.21, 0.00)	.05	
Percentage Difference (vs Pooled-Sham)	-13.60			-18.10		
Difference (95% CI) in LS Mean (vs PEOM)	0.03 (-0.08, 0.13)		.64			
Percentage Difference (vs PEOM)	5.50					
**PRD in isolation (mm^2)**						
LS Mean (SE)	-0.29 (0.03)			-0.32 (0.02)		-0.33 (0.02)
Difference (95% CI) in LS Mean (vs Pooled-Sham)	0.03 (-0.03, 0.10)	.31		0.00 (-0.06, 0.07)	.92	
Percentage Difference (vs Pooled-Sham)	-10.50			-1.00		

© 2024 Fu DJ et al. *JAMA Ophthalmology.*

Difference (95% CI) in LS Mean (vs PEOM)	0.03 (-0.03, 0.10)		.35			
Percentage Difference (vs PEOM)	-9.60					

Perifoveal ETDRS region (21.2 mm^2) from baseline to month 24

	PM	P-value (PM vs. Pooled-Sham)	P-value (PM vs. PEOM)	PEOM	P-value (PEOM vs. Pooled-Sham)	Pooled-Sham
**RORA (mm^2)**						
LS Mean (SE)	1.67 (0.07)			1.84 (0.08)		2.28 (0.09)
Difference (95% CI) in LS Mean (vs Pooled-Sham)	-0.61 (-0.84, -0.37)	<.0001		-0.44 (-0.68, -0.20)	<.001	
Percentage Difference (vs Pooled-Sham)	-26.70			-19.40		
Difference (95% CI) in LS Mean (vs PEOM)	-0.17 (-0.37, 0.04)		.12			
Percentage Difference (vs PEOM)	-9.00					
**RPE-loss (mm^2)**						
LS Mean (SE)	1.60 (0.09)			1.85 (0.08)		2.34 (0.09)
Difference (95% CI) in LS Mean (vs Pooled-Sham)	-0.75 (-0.99, -0.50)	<.0001		-0.49 (-0.74, -0.25)	<.0001	
Percentage Difference (vs Pooled-Sham)	-31.80			-21.00		
Difference (95% CI) in LS Mean (vs PEOM)	-0.25 (-0.49, -0.02)		.035			
Percentage Difference (vs PEOM)	-13.70					

© 2024 Fu DJ et al. *JAMA Ophthalmology.*

**Hypertransmission (mm²)**						
LS Mean (SE)	1.93 (0.10)			2.00 (0.09)		2.39 (0.10)
Difference (95% CI) in LS Mean (vs Pooled-Sham)	-0.46 (-0.73, -0.19)	<.001		-0.40 (-0.66, -0.13)	.003	
Percentage Difference (vs Pooled-Sham)	-19.40			-16.50		
Difference (95% CI) in LS Mean (vs PEOM)	-0.07 (-0.32, 0.19)		.60			
Percentage Difference (vs PEOM)	-3.40					
**Photoreceptor degeneration (PRD; mm²)**						
LS Mean (SE)	1.68 (0.14)			1.73 (0.12)		2.57 (0.14)
Difference (95% CI) in LS Mean (vs Pooled-Sham)	-0.89 (-1.27, -0.51)	<.0001		-0.84 (-1.20, -0.49)	<.0001	
Percentage Difference (vs Pooled-Sham)	-34.60			-32.80		
Difference (95% CI) in LS Mean (vs PEOM)	-0.05 (-0.40, 0.31)		.80			
Percentage Difference (vs PEOM)	-2.60					
**PRD in isolation (mm²)**						
LS Mean (SE)	-0.19 (0.10)			-0.30 (0.09)		0.06 (0.10)
Difference (95% CI) in LS Mean (vs Pooled-Sham)	-0.25 (-0.51, 0.02)	.07		-0.36 (-0.61, -0.11)	<.001	
Percentage Difference (vs Pooled-Sham)	-422.70			-617.80		

© 2024 Fu DJ et al. *JAMA Ophthalmology.*

Difference (95% CI) in LS Mean (vs PEOM)	0.11 (-0.13, 0.36)	.37
Percentage Difference (vs PEOM)	-37.70	

eTable 2. Least squares mean change in best-corrected visual acuity from baseline at 12, 18, and 24 months post-baseline.

Table shows least square (LS) mean change of area, standard error (SE), absolute and percentage difference between treatment groups and 95% confidence interval (CI). P-values shown for comparison of pegceptacoplan monthly (PM), pegceptacoplan every other month (PEOM), and pooled-sham.

Analysis of change in BCVA (ETDRS Letters) from baseline to month 24 with MMRM model						
	PM	P-value (PM vs. Pooled-Sham)	P-value (PM vs. PEOM)	PEOM	P-value (PEOM vs. Pooled-Sham)	Pooled-Sham
**Month 12**						
LS Mean (SE)	-3.02 (0.55)			-4.51 (0.64)		-2.75 (0.64)
Difference (95% CI) in LS Mean (vs Pooled-Sham)	-0.28 (-1.92, 1.37)	.74		-1.77 (-3.53, 0.00)	.50	
Percentage Difference (vs Pooled-Sham)	10.0			64.3		
Difference (95% CI) in LS Mean (vs PEOM)	1.49 (-0.16, 3.14)		.08			
Percentage Difference (vs PEOM)	-33.0					
**Month 18**						
LS Mean (SE)	-5.32 (0.74)			-6.16 (0.76)		-5.67 (0.72)
Difference (95% CI) in LS Mean (vs Pooled-Sham)	0.34 (-1.66, 2.35)	.74		-0.50 (-2.54, 1.55)	.63	
Percentage Difference (vs Pooled-Sham)	-6.1			8.8		

© 2024 Fu DJ et al. *JAMA Ophthalmology.*

Difference (95% CI) in LS Mean (vs PEOM)	0.84 (-1.24, 2.92)		.43			
Percentage Difference (vs PEOM)	-13.6					
**Month 24**						
LS Mean (SE)	-7.65 (0.82)			-8.94 (0.85)		-7.54 (0.86)
Difference (95% CI) in LS Mean (vs Pooled-Sham)	-0.11 (-2.41, 2.20)	.93		-1.40 (-3.76, 0.96)	.24	
Percentage Difference (vs Pooled-Sham)	1.4			18.6		
Difference (95% CI) in LS Mean (vs PEOM)	1.30 (-1.01, 3.61)		.27			
Percentage Difference (vs PEOM)	-14.5					

eTable 3. Supplementary Table 3. Spearman Correlation of Change from Baseline between GA SD-OCT feature and BCVA at Month 24.

Spearman correlation analysis of change from baseline to month 24 in BCVA (best-corrected visual acuity) letters and SD-OCT features of GA area within the 6 mm diameter ETDRS region. Presented is Spearman partial correlation adjusted for baseline SD-OCT GA feature area, baseline BCVA score, and treatment group. GA features considered were RORA, RPE-loss, hypertransmission, PRD, and PRD in isolation.

		PM	PEOM	Pooled-Sham	Overall
**RORA**					
	Correlation	-0.04	-0.22	-0.18	-0.15
	P-value	.52	<.001	.005	<.001
**RPE-loss**					
	Correlation	-0.05	-0.21	-0.19	-0.15
	P-value	.46	.001	.003	<.001
**Hypertransmission**					

Correlation	-0.08	-0.19	-0.22	-0.17
P-value	.21	.003	<.001	<.001
**Photoreceptor degeneration (PRD)**				
Correlation	0.02	-0.08	-0.16	-0.07
P-value	.74	.19	.01	.05
**PRD in isolation**				
Correlation	0.11	0.08	0.04	0.07
P-value	.10	.22	.58	.05

eTable 4. Change in area from baseline at 12, 18, and 24 months post baseline – Sensitivity analyses.

Table shows least squares mean change of area, standard error (SE), absolute and percentage difference between treatment groups and 95% confidence interval (CI). ANOVA model used with all available observed values without any covariate adjustment. P-values shown for comparison of pegceptacoplan monthly (PM), pegceptacoplan every other month (PEOM), and pooled-sham.

**Change in area (mm^2) of RORA from baseline through month 24 with observed data only**					
	PM	P-value (PM vs. Pooled Sham)	PEOM	P-value (PEOM vs. Pooled Sham)	Pooled Sham
**Number of Subjects Included in the Model**	**240**		**255**		**251**
**Change in RORA (mm^2) at month 12**					
LS Mean (SE)	1.19 (0.07)		1.44 (0.07)		1.81 (0.07)
Difference (95% CI) in LS Mean (vs Pooled-Sham)	-0.62 (-0.81, -0.43)	<.001	-0.37 (-0.56, -0.18)	<.001	
Percentage Difference (vs Pooled-Sham)	-34.2		-20.4		
**Change in RORA (mm^2) at month 18**	**228**		**249**		**243**
LS Mean (SE)	1.83 (0.09)		2.10 (0.08)		2.63 (0.09)
Difference (95% CI) in LS Mean (vs Pooled-Sham)	-0.81 (-1.05, -0.56)	<.001	-0.53 (-0.77, -0.30)	<.001	
Percentage Difference (vs Pooled-Sham)	-30.6		-20.3		
**Change in RORA (mm^2) at month 24**	**217**		**245**		**250**
LS Mean (SE)	2.61 (0.11)		2.7629 (0.10165)		3.53(0.10)
Difference (95% CI) in LS Mean (vs Pooled-Sham)	-0.92 (-1.21, -0.63)	<.001	-0.7668 (-1.0477, -0.4860)	<.001	
Percentage Difference (vs Pooled-Sham)	-26.0		-21.7		

© 2024 Fu DJ et al. *JAMA Ophthalmology.*

RPE-loss

	PM	P-value (PM vs. Pooled Sham)	PEOM	P-value (PEOM vs. Pooled Sham)	Pooled Sham
**Number of Subjects Included in the Model**	**240**		**255**		**251**
**Change in RPE-loss (mm²) at month 12**					
LS Mean (SE)	1.22 (0.075)		1.40 (0.07)		1.92 (0.07)
Difference (95% CI) in LS Mean (vs Pooled-Sham)	-0.71 (-0.91, -0.50)	<.001	-0.52 (-0.73, -0.32)	<.001	
Percentage Difference (vs Pooled-Sham)	-36.7		-27.2		
**Change in RPE-loss (mm²) at month 18**	**228**		**249**		**243**
LS Mean (SE)	1.81 (0.09)		2.11 (0.09)		2.74 (0.09)
Difference (95% CI) in LS Mean (vs Pooled-Sham)	-0.93 (-1.19, -0.66)	<.001	-0.63 (-0.89, -0.37)	<.001	
Percentage Difference (vs Pooled-Sham)	-33.9		-22.8		
**Change in RPE-loss (mm²) at month 24**	**217**		**245**		**250**
LS Mean (SE)	2.54 (0.11)		2.76 (0.11)		3.56 (0.11)
Difference (95% CI) in LS Mean (vs Pooled-Sham)	-1.03 (-1.33, -0.71)	<.001	-0.81 (-1.11, -0.51)	<.001	
Percentage Difference (vs Pooled-Sham)	-28.8		-22.7		

Hypertransmission

	PM	P-value (PM vs. Pooled Sham)	PEOM	P-value (PEOM vs. Pooled Sham)	Pooled Sham
**Number of Subjects Included in the Model**	**240**		**255**		**251**
**Change in Hypertransmission (mm²) at month 12**					
LS Mean (SE)	1.25 (0.08)		1.48 (0.08)		1.74 (0.08)
Difference (95% CI) in LS Mean (vs Pooled-Sham)	-0.49 (-0.71, -0.28)	<.001	-0.26 (-0.47, -0.04)	.03	
Percentage Difference (vs Pooled-Sham)	-28.4		-14.8		
**Change in Hypertransmission (mm²) at month 18**	**228**		**249**		**243**
LS Mean (SE)	2.03 (0.10)		2.11(0.09)		2.62(0.09)
Difference (95% CI) in LS Mean (vs Pooled-Sham)	-0.59 (-0.85, -0.32)	<.001	-0.51 (-0.77, -0.25)	<.001	
Percentage Difference (vs Pooled-Sham)	-22.5		-19.5		
**Change in Hypertransmission (mm²) at month 24**	**217**		**245**		**250**

© 2024 Fu DJ et al. *JAMA Ophthalmology.*

LS Mean (SE)	2.82 (0.12)		2.83 (0.11)		3.49 (0.11)
Difference (95% CI) in LS Mean (vs Pooled-Sham)	-0.67 (-0.98, -0.35)	<.001	-0.66 (-0.97, -0.35)	<.001	
Percentage Difference (vs Pooled-Sham)	-19.1		-18.9		

Photoreceptor degeneration (PRD)

	PM	P-value (PM vs. Pooled Sham)	PEOM	P-value (PEOM vs. Pooled Sham)	Pooled Sham
**Number of Subjects Included in the Model**	**240**		**255**		**251**
**Change in PRD (mm^2) at month 12**					
LS Mean (SE)	1.00 (0.14)		0.95 (0.13)		1.91 (0.14)
Difference (95% CI) in LS Mean (vs Pooled-Sham)	-0.91 (-1.29, -0.53)	<.001	-0.96 (-1.34, -0.59)	.02	
Percentage Difference (vs Pooled-Sham)	-47.8		-50.3		
**Change in PRD (mm^2) at month 18**	**228**		**249**		**243**
LS Mean (SE)	1.45 (0.15)		1.62 (0.15)		2.68 (0.15)
Difference (95% CI) in LS Mean (vs Pooled-Sham)	-1.23 (-1.65, -0.81)	<.001	-1.07 (-1.48, -0.66)	<.001	
Percentage Difference (vs Pooled-Sham)	-45.8		-39.8		
**Change in PRD (mm^2) at month 24**	**217**		**245**		**250**
LS Mean (SE)	2.23 (0.16)		2.23 (0.15)		3.25 (0.15)
Difference (95% CI) in LS Mean (vs Pooled-Sham)	-1.02 (-1.46, -0.58)	<.001	-1.02 (-1.44, -0.60)	<.001	
Percentage Difference (vs Pooled-Sham)	-31.3		-31.5		

PRD in isolation

	PM	P-value (PM vs. Pooled Sham)	PEOM	P-value (PEOM vs. Pooled Sham)	Pooled Sham
**Number of Subjects Included in the Model**	**240**		**255**		**251**
**Change in PRD in isolation (mm^2) at month 12**					
LS Mean (SE)	-0.32 (0.12)		-0.57 (0.12)		0.02 (0.12)
Difference (95% CI) in LS Mean (vs Pooled-Sham)	-0.34 (-0.68, -0.001)	.04	-0.59(-0.92, -0.25)	<.001	
Percentage Difference (vs Pooled-Sham)	-1604.6		-2758.9		
**Change in PRD in isolation (mm^2) at month 18**	**288**		**249**		**243**
LS Mean (SE)	-0.55 (0.13)		-0.58 (0.13)		-0.07 (0.13)

© 2024 Fu DJ et al. *JAMA Ophthalmology.*

Difference (95% CI) in LS Mean (vs Pooled-Sham)	-0.48 (-0.84, -0.11)	.01	-0.51 (-0.86, -0.15)	<.001	
Percentage Difference (vs Pooled-Sham)	667.4		712.0		
**Change in PRD in isolation (mm²) at month 24**	**217**		**245**		**250**
LS Mean (SE)	-0.56 (0.14)		-0.63 (0.13)		-0.35 (0.13)
Difference (95% CI) in LS Mean (vs Pooled-Sham)	-0.21 (-0.58, 0.15)	.26	-0.27 (-0.63, 0.08)	.13	
Percentage Difference (vs Pooled-Sham)	60.1		78.4		

© 2024 Fu DJ et al. *JAMA Ophthalmology.*

eMethods

Study design and cohort selection

Key inclusion criteria were: 60 years of age or older; best-corrected visual acuity (BCVA) ≥24 letter score on Early Treatment Diabetic Retinopathy Study (ETDRS) charts; total GA area between 2.5 - 17.5 mm^2 with - if multifocal - at least one lesion ≥1.25 mm^2 on FAF imaging. Key study eye exclusion criteria included: GA secondary to a condition other than AMD and any history or presence of active choroidal neovascularization (CNV), including any evidence of RPE tears or neovascularization elsewhere. Participants Patients were randomly assigned 2:2:1:1 to receive 15 mg per 0.1 mL intravitreal injection pegcetacoplan monthly (PM) or every other month (PEOM), or sham injection monthly (SM) or every other month (SEOM), for 24 months.

All participants with SD-OCT volumes acquired using Heidelberg Spectralis OCT and HRA (Heidelberg Engineering, Heidelberg, Germany) and with 25 or more B-scans covering 6x6x2 mm^3 were taken forward for analysis, resulting in 10,688 volumes of 936 eyes (310 PM; 309 PEOM; 619 pegceptacoplan pooled; 157 SM; 160 SEOM; 317 pooled-sham) (**Supplementary Figure 1**). In cases of multiple SD-OCT volume scans for a given eye at a given timepoint, the volume closest to 49 B-scans was used.

Image analysis workflow

Deep-learning models were applied across each B-scan of every SD-OCT volume scan to extract each of the CAM-defined morphological features that define GA (RPE-loss, PRD, and hypertransmission). Resultant voxel-level binary labels were used to assess the presence or absence of each feature per vertical column (A-scan) and were subsequently projected onto the corresponding pixel of a paired en face near-infrared fundus photo. Spatial localisation of RPE-loss, PRD, and hypertransmission were considered collectively to extrapolate the additional features: PRD in isolation (PRD without overlapping RPE-loss or hypertransmission) and RORA (RPE-loss and Outer Retinal Atrophy). RORA was defined as regions of overlapping RPE-loss, PRD, and hypertransmission; and can thus be considered as a continuous variable that encompasses both incomplete RORA and complete RORA).[12,36]

To enable longitudinal comparison of SD-OCT time-series from each study participant over 24 months of follow-up and account for inconsistencies in image capture of the macular region, a deep-learning model for foveal localisation developed by our research team was also applied to guide the accurate positioning of the circular, 6 mm diameter Early Treatment of Diabetic Retinopathy Study (ETDRS) grid. Quality control by manual inspection of foveal center localisation and ETDRS grid positioning was undertaken by expert Reading Centre graders. Areas of each feature could thus be summarized by retinal regions

© 2024 Fu DJ et al. *JAMA Ophthalmology.*

divided up into the ETDRS grid of three concentric rings: the 1 mm diameter ETDRS central foveal region; the inner ETDRS annulus of 3 mm outer diameter and 1 mm inner diameter; and outer perifoveal ETDRS annulus with 6 mm diameter outer diameter and 3 mm inner diameter.[37]

For FAF images, GA was determined from FAF images by manually delineating regions of hypo-autofluorescence by a minimum of two certified readers with independent manual measurements of features of the GA lesions and measured values served as primary endpoints for the DERBY and OAKS studies. The imaging field size with FAF was 30 x 30 degrees (10 mm x 10 mm) and thus captures a greater field than with a macular SD-OCT (6 mm x 6 mm). Total area of GA features between FAF and SD-OCT are therefore not directly comparable.

Study Outcomes

Normal-luminance best-corrected visual acuity scores were assessed by ETDRS charts.

Statistical analysis

A mixed-effect model for repeated measures (MMRM) was used to analyze the change from baseline to month 24 in the total area of GA lesion(s), with a random intercept at the level of the participant that included the following as cross-level interactions: treatment, presence of CNV in the fellow eye (Yes or No); Baseline GA lesion area (< 7.5 mm^2 or >= 7.5 mm^2); Baseline SD-OCT GA feature; analysis visit; treatment x analysis visit; and baseline SD-OCT GA feature x analysis visit.

An MMRM was carried out for change from baseline in BCVA measured as ETDRS letter score, with a random intercept at the level of the participant that included the following as cross-level interactions: treatment, Baseline GA Lesion Area (< 7.5 mm2 or >= 7.5 mm2); Baseline BCVA Score; Analysis Visit; Baseline Presence of CNV in the fellow eye (Yes or No); Analysis Visit x Treatment; Baseline BCVA Score; Analysis Visit. PM = Pegceptacoplan monthly; PEOM = Pegceptacoplan every other month.

Spearman partial correlations adjusted for baseline GA SD-OCT feature, baseline NL-BCVA score, and treatment group were used to analyze the correlation between change in BCVA from baseline to month 24 for the mITT cohort and change from baseline to Month 24 in GA features on SD-OCT. GA features considered were RORA, RPE-loss, hypertransmission, PRD, and PRD in isolation.

Analysis of variance (ANOVA) was used to compare the three treatment groups (PM, PEOM, sham pooled) at various timepoints (Month 6, Month 12, Month 18, and Month 24) of GA lesion(s) without any covariate adjustment. GA features considered were RORA, RPE-loss, hypertransmission, PRD, and PRD in isolation.

© 2024 Fu DJ et al. *JAMA Ophthalmology.*

OAKS & DERBY PROTOCOL

Available as part of registration on ClinicalTrials.gov, NCT03525613 (OAKS; https://storage.googleapis.com/ctgov2-large-docs/13/NCT03525613/Prot_000.pdf) and NCT03525600 (DERBY; https://storage.googleapis.com/ctgov2-large-docs/00/NCT03525600/Prot_000.pdf), as well as, in Supplementary Materials of primary outcome publication: Heier JS, Lad EM, Holz FG, et al. Pegcetacoplan for the treatment of geographic atrophy secondary to age-related macular degeneration (OAKS and DERBY): two multicentre, randomized, double-masked, sham-controlled, phase 3 trials. Lancet. 2023;402(10411):1434-1448.

STATISTICAL ANALYSIS PLAN

Published as part of registration on ClinicalTrials.gov, NCT03525613 (OAKS; https://storage.googleapis.com/ctgov2-large-docs/13/NCT03525613/SAP_001.pdf) and NCT03525600 (DERBY; https://storage.googleapis.com/ctgov2-large-docs/00/NCT03525600/SAP_001.pdf),

© 2024 Fu DJ et al. *JAMA Ophthalmology.*
