# Supplementary material for: Pegcetacoplan Treatment and Consensus Features of Geographic Atrophy Over 24 Months
Source: JAMA Ophthalmol. 2024 May 9;142(6):548–58. doi: 10.1001/jamaophthalmol.2024.1269 (PMC11082756; doi:10.1001/jamaophthalmol.2024.1269)
Supplement: Supplement 3. — Data sharing statement [file jamaophthalmol-e241269-s003.pdf]

Data Sharing Statement

Fu. Pegcetacoplan Treatment and Consensus Features of Geographic Atrophy Over 24 Months. *JAMA Ophthalmol*. Published May 09, 2024. doi:10.1001/jamaophthalmol.2024.1269

Data
Data available: No

Additional Information
Explanation for why data not available: Proprietary clinical trial data to Apellis plc from the Phase III DERBY and OAKS Studies.
